# Atroposelective desymmetrization of 2-arylresorcinols via Tsuji-Trost allylation

**DOI:** 10.1038/s42004-023-00839-z

**Published:** 2023-02-25

**Authors:** Sangji Kim, Aram Kim, Chanhee Lee, Junsoo Moon, Eun Jeong Hong, Duck-Hyung Lee, Yongseok Kwon

**Affiliations:** 1 School of Pharmacy, Sungkyunkwan University, Suwon, 16419 Republic of Korea; 2 Department of Chemistry, Sogang University, Seoul, 04107 Republic of Korea

**Keywords:** Synthetic chemistry methodology, Asymmetric catalysis, Asymmetric synthesis

## Abstract

Palladium-catalyzed asymmetric allylic alkylation has proven to be a powerful method for the preparation of a wide variety of chiral molecules. However, the catalytic and atroposelective allylic alkylation is still rare and challenging, especially for biaryl substrates. Herein, we report the palladium-catalyzed desymmetric and atroposelective allylation, in which the palladium complex with a chiral phosphoramidite ligand enables desymmetrization of nucleophilic 2-arylresorcinols in a highly enantioselective manner. With the aid of the secondary kinetic resolution effect, a wide variety of substrates containing a hydroxymethyl group at the bottom aromatic ring are able to provide *O*-allylated products up to 98:2 er. Computational studies show an accessible quadrant of the allylpalladium complex and provide three plausible transition states with intra- or intermolecular hydrogen bonding. The energetically favorable transition state is in good agreement with the observed enantioselectivity and suggests that the catalytic reaction would proceed with an intramolecular hydrogen bond.

## Abstract (web summary)

Desymmetrization of symmetric biaryls is an efficient but underexplored way to approach axially chiral molecules. Here, the authors report the atroposelective desymmetrization of 2- arylresorcinols via chiral palladium complex-catalyzed allylic alkylation.

## Introduction

**Fig. 1:**
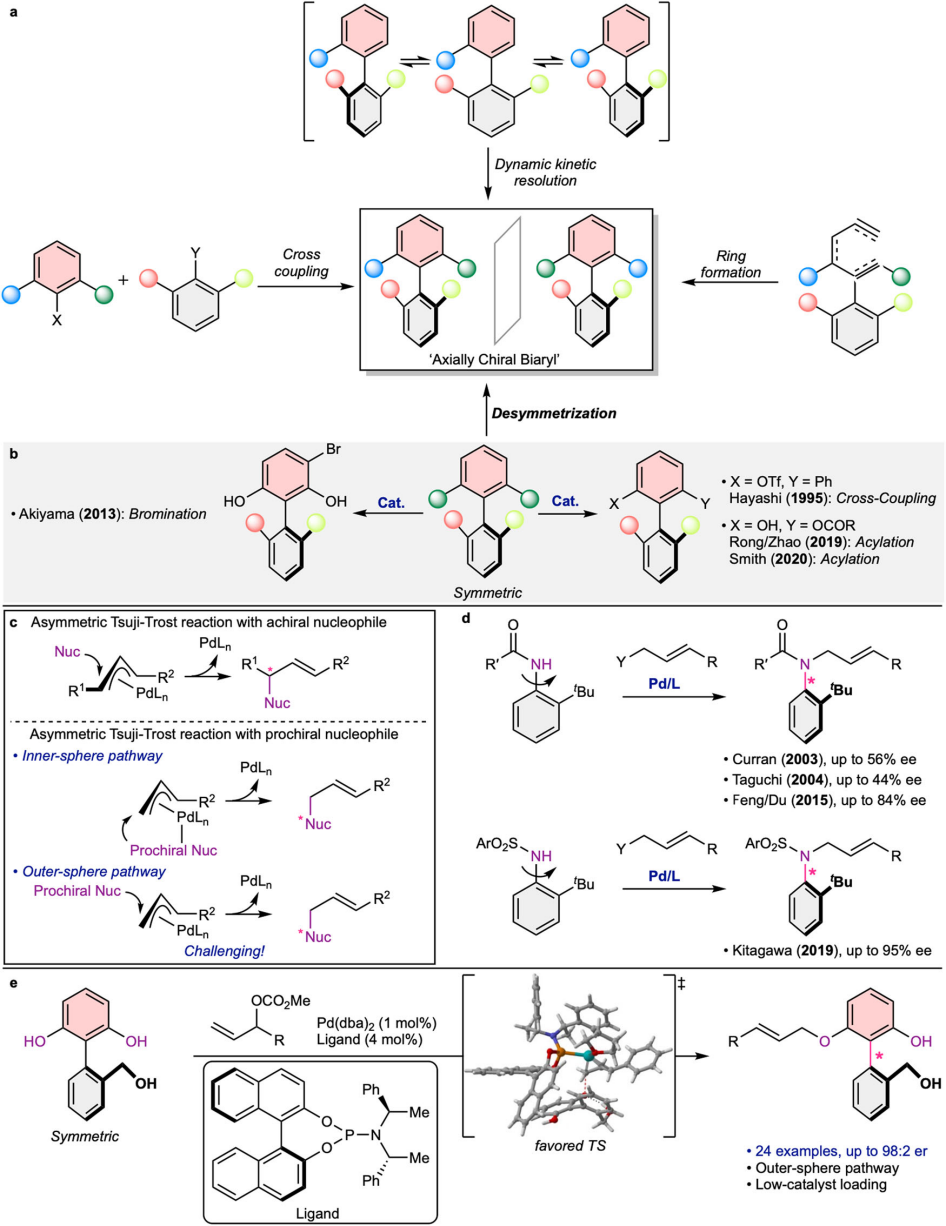
Strategies on the asymmetric synthesis of axially chiral biaryls and the catalytic asymmetric Tsuji-Trost reactions. **a** Strategies on the asymmetric synthesis of axially chiral biaryls. **b** Representative examples for atroposelective desymmetrization of biaryls. **c** Catalytic asymmetric Tsuji-Trost reactions. **d** Atroposelective allylations of anilides by dynamic kinetic resolution. **e** Palladium-catalyzed atroposelective desymmetric allylation (this work).

The development of catalytic and enantioselective syntheses of axially chiral biaryls has been extensively explored^1–7^ because it provides a highly efficient and selective route to access natural products^8,9^, biologically active compounds^10–12^, and chiral catalysts^13–15^. Strategies to control a stereogenic axis are generally classified into several categories^16–23^, such as direct cross-coupling, dynamic kinetic resolution, ring formation, and desymmetrization (Fig. 1a)^24–31^. As each strategy inherently possesses its own strengths and weaknesses, they are necessarily complementary to each other depending on a target molecule. Thus, the diversification of methodologies employing various strategies is highly demanding to expand accessible axially chiral molecules. Among the strategies, desymmetrization of configurationally stable and symmetric biaryls can provide an alternative and efficient way to approach axially chiral molecules (Fig. 1b)^32–44^. However, compared to other strategies, a limited number of reactions have been reported, which represents the current cutting edge of this type of reaction^33–44^.

Palladium-catalyzed asymmetric allylic alkylation, also known as Tsuji-Trost allylation, is a powerful method to form a C–C bond or C–heteroatom bond with high enantioselectivities^45–48^. In this reaction, a π-allyl-Pd complex with a chiral ligand is formed, in which a nucleophile generally approaches an allyl group in the opposite direction of the Pd atom (Fig. 1c). In this regard, asymmetric reactions essentially have focused on the control of a stereogenic center that is newly generated on an allyl group^46–48^, while investigation of the stereogenicity on a nucleophile is relatively underexplored^49–58^. When a hard nucleophile is employed, it can be bound to the palladium center and then transferred to the allyl group (inner-sphere pathway). In this scenario, the orientation and conformation of a prochiral nucleophile should be limited and affected by the chiral palladium complex^49^. However, a soft prochiral nucleophile directly attacks to the allyl group from the outside of the catalytic complex (outer-sphere pathway) which should be challenging to develop new asymmetric methodology^50^. For these reasons, this type of reactions has restrictively been applied to control the stereogenic axis. For example, nonbiaryl anilides were initially investigated by dynamic kinetic resolution (Fig. 1d) by Taguchi^55^ and Curran^56^ in the early 2000s. Even though the nucleophilic nitrogen atom itself consists of the stereogenic axis, moderate enantioselectivities were observed. Further efforts have been made by Feng/Du^57^ and Kitagawa^58^ to enhance enantioselectivities around the C–N bond. However, to the best of our knowledge, Pd-catalyzed allylic alkylation of a biaryl substrate has not been developed. Furthermore, this type of reaction has not been explored with atroposelective desymmetrization despite its long and powerful history.

At the outset of our work, we hypothesized that a chiral palladium complex could atroposelectively desymmetrize symmetric biaryls by distinguishing two prochiral heteroatom nucleophiles. Given the previous literature^46–48^, the major challenge to realize atroposelective allylic alkylation probably lies in (1) the opposite and remote arrangement of a chiral ligand with prochiral heteroatom nucleophiles and (2) the possible multiple orientations of the prochiral nucleophile when intermolecularly approaching the allylpalladium complex. To overcome these challenges, we envisioned that a catalytic complex should provide an extensive chiral environment around the π-allyl-Pd center to limit the orientation of the nucleophile. Furthermore, an additional functional group on the other side of the aromatic ring would be desirable to make favorable interactions. Herein, we report a highly atroposelective Pd-catalyzed allylation, in which achiral 2-arylresorcinols are desymmetrized by distinguishing two symmetric phenolic hydroxyl groups (Fig. 1e).

## Results and discussion

### Reaction optimizations

To test our hypothesis, we designed a substrate, **1a**, containing resorcinol at the top and *ortho*-benzylalcohol at the bottom (Table 1)^34^. We initially envisioned that the bottom hydroxyl group would form a desirable intra- or intermolecular hydrogen bond for the catalytic and enantioselective reaction. With methyl cinnamyl carbonate (**2a**), preliminary chiral ligand screening was performed (Table 1, entries 1–4), in which desired product **3aa** was obtained along with disubstituted product **4aa**. Among the tested ligands, phosphoramidite ligand^59^ (**L4**) was found to be the most effective at affording **3aa** in 44% yield and 91:9 er (Table 1, entry 4 vs. entries 1–3). Based on the results from the preliminary experiments, we performed a thorough investigation with a series of phosphoramidite ligands (**L5**–**L13**), as summarized in Table 1, entries 5–13. Interestingly, when the binaphthyl group was substituted with a biphenyl group (**L5**), enantioselectivity was retained to a degree. This result suggested that the point chirality of the catalysts would play an important role in the observed selectivity. However, further modifications of the ligands did not improve the enantioselectivities (Table 1, entries 6–13), which suggested that all component of **L4** would involve to generate enantioselective environment in good harmony with the allylpalladium complex. We were pleased to find that enantioselectivity was enhanced to 93:7 er, when the reaction was performed at −20 °C (Table 1, entry 14). When the branched carbonate (**2a′**) was employed, the enantioselectivity slightly increased to 94:6 er (Table 1, entry 15). After the exhaustive optimization of various reaction parameters (see Supplementary Table 1−5), we were able to establish the optimized reaction conditions (1.5 equiv of **2a′**, 1 mol% of Pd(dba)_2_, 4 mol% of **L4**, THF,−20 °C) to provide the desired product in 49% yield and 97:3 er (Table 1, entry 16).

**Table 1:** Reaction optimizations^a^.


Entry	2a / 2a′	Ligand	Temp (°C)	Time (h)	Yield^b^ of 3aa (%)	Enantiomeric ratio^c^ (er)	Yield^b^ of 4aa (%)
1	**2a**	**L1**	0	48	30	49:51	6
2	**2a**	**L2**	0	48	<5	n.d.^d^	n.d.^d^
3	**2a**	**L3**	0	48	25	35:65	3
4	**2a**	**L4**	0	2	44	91:9	18
5	**2a**	**L5**	0	3	18	89:11	10
6	**2a**	**L6**	0	48	11	67:33	n.d.^d^
7	**2a**	**L7**	0	1.5	42	41:59	26
8	**2a**	**L8**	0	48	11	57:43	n.d.^d^
9	**2a**	**L9**	0	3	32	57:43	11
10	**2a**	**L10**	0	0.5	27	58:42	17
11	**2a**	**L11**	0	0.5	49	61:39	23
12	**2a**	**L12**	0	11	38	53:47	26
13	**2a**	**L13**	0	0.5	59	44:56	12
14	**2a**	**L4**	−20	3	55	93:7	23
15	**2a′**	**L4**	−20	3	55	94:6	22
16^e^	**2a′**	**L4**	−20	10	49	97:3	42

^a^Unless otherwise noted, the reactions were carried out with **1a** (0.10 mmol, 1.0 equiv), **2a** or **2a′** (0.10 mmol, 1.0 equiv), Pd(dba)_2_ (0.005 mmol, 0.05 equiv), **L1**−**L13** (0.02 mmol, 0.2 equiv), THF (0.5 mL, 0.2 M).

^b^Isolated yields.

^c^Enantiomeric ratios were determined by chiral-phase high-performance liquid chromatography analysis.

^d^Not determined.

^e^1 mol% of Pd(dba)_2_, 4 mol% of **L4**, and 1.5 equiv of **2a′** were employed.

### Substrate scope

**Fig. 2:**
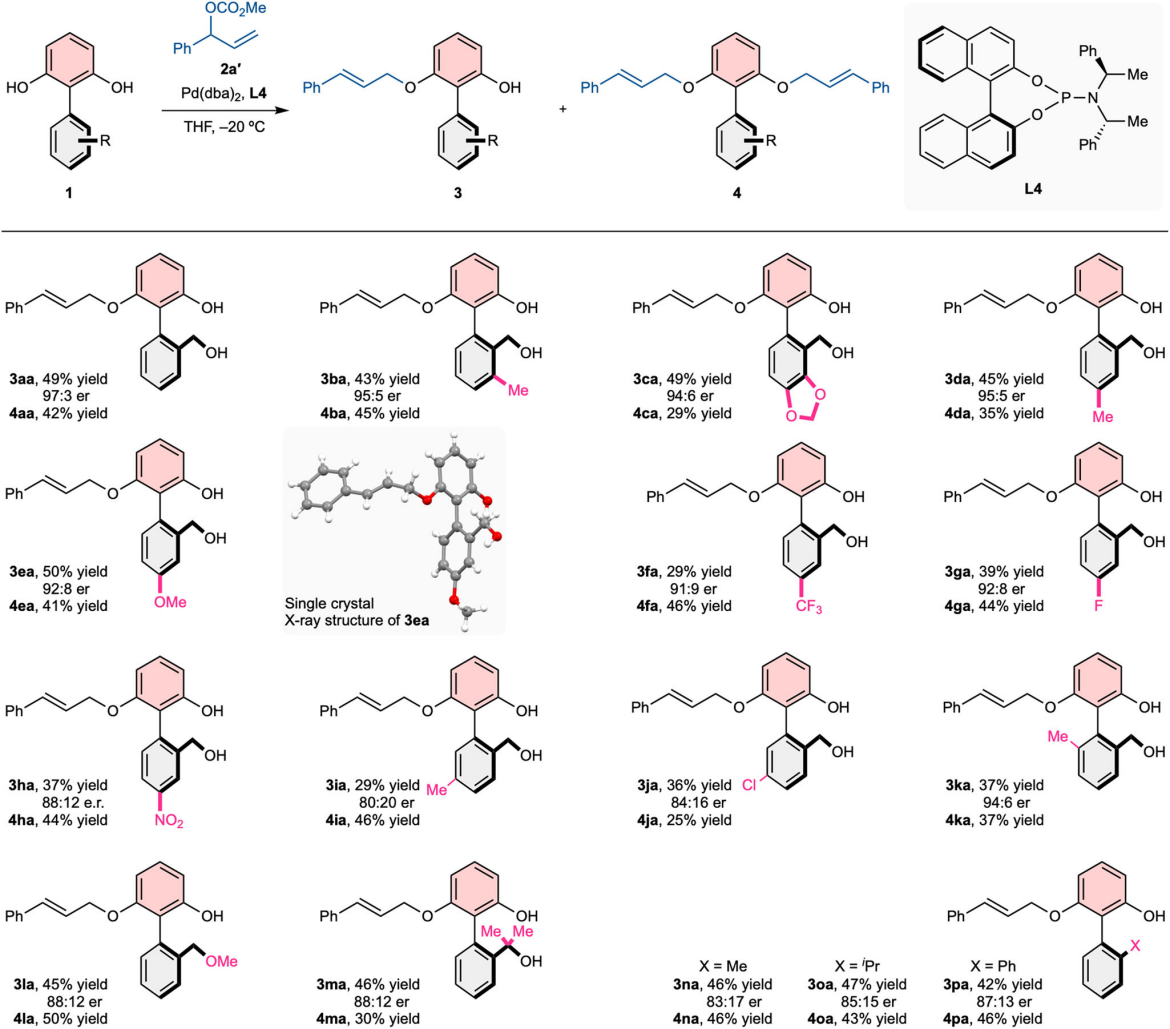
Substrate scope of bottom aromatic ring. Unless otherwise noted, the reactions were carried out with **1** (0.10 mmol, 1.0 equiv), **2a′** (0.15 mmol, 1.5 equiv), Pd(dba)_2_ (0.001 mmol, 0.01 equiv), **L4** (0.004 mmol, 0.04 equiv), THF (0.5 mL, 0.2 M). Isolated yields. Enantiomeric ratios were determined by chiral-phase high-performance liquid chromatography analysis.

**Fig. 3:**
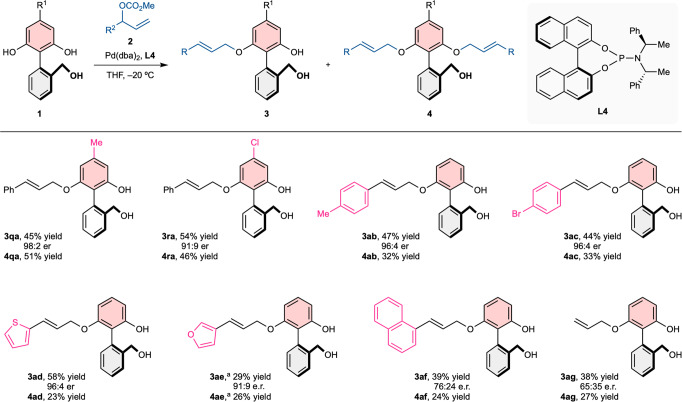
Substrate scope of top aromatic ring and allyl carbonate. Unless otherwise noted, the reactions were carried out with **1** (0.10 mmol, 1.0 equiv), **2a′** (0.15 mmol, 1.5 equiv), Pd(dba)_2_ (0.001 mmol, 0.01 equiv), **L4** (0.004 mmol, 0.04 equiv), THF (0.5 mL, 0.2 M). Isolated yields. Enantiomeric ratios were determined by chiral-phase high-performance liquid chromatography analysis. ^a^0.05 equiv of Pd(dba)_2_ and 0.20 equiv of **L4** were employed.

Next, we explored the substrate scope under the optimized reaction conditions, as summarized in Fig. 2 and Fig. 3 (for detailed procedures, NMR spectra and HPLC chromatograms, see Supplementary Methods and Supplementary Data 2 and 3). In general, the modifications of bottom aromatic ring in 2-arylresorcinols are highly tolerable to give the desired products with high atroposelectivity (Fig. 2). The reaction of the substrate (**1b**) bearing a methyl group afforded the desired product (**3ba**) with 95:5 er. The substrate containing 1,3-dioxolane was reacted with **2a′** to provide the allylated product (**3ca**) with 94:6 er. The introductions of electron-donating groups such as methyl and methoxy group at the *para*-position of the stereogenic axis were tolerable to afford **3da** with 95:5 er and **3ea** with 92:8 er, respectively. When the reactions were performed with the substrates substituted with electron-withdrawing groups, the desired product were obtained in good enantioselectivity (**3fa**, 91:9 er; **3ga**, 92:8 er; **3ha**, 88:12 er), albeit with slightly lower yield. However, the substrates that were substituted at the *para* position of the hydroxymethyl group showed lower selectivities (**3ia**, 80:20 er; **3ja**, 84:16 er), presumably due to the unfavorable interactions between the substituent and catalytic complex. It was observed that the substitution at the *ortho* position to the stereogenic axis was tolerable to give the desired product (**3ka**) in 94:6 er. Notably, substrates (**1l**−**1p**) containing different functional groups instead of the hydroxymethyl group at the bottom aromatic ring were found to be tolerable to a degree. For example, the substrate containing a methoxymethyl or 2-hydroxyisopropyl group was reacted in the optimized reaction conditions to give the allylated products (**3ha** or **3ia**) in 88:12 er. Also, the reactions of the substrates with a methyl, isopropyl, or phenyl group provided the desired products with good enantioselectivity (**3na**, methyl-, 83:17 er; **3oa**, isopropyl-, 85:15 er; **3pa**, phenyl-, 87:13 er). These results suggest that the observed enantioselectivity would originate from repulsive interactions between the catalytic complex and substrate, and the hydroxyl group in **1a** would facilitate the asymmetric transformation. Next, further modifications on the top aromatic ring and allyl carbonate were performed, which showed high compatibility of our methodology (Fig. 3). When substrates were substituted with a methyl or bromide at the top aromatic ring, they provided **3qa** with 98:2 er and **3ra** with 91:9 er, respectively. The reaction of **1a** with **2b** and **2c** in which the cinnamyl group was substituted with a methyl or bromide group provided the desired products with 96:4 er. While thiophene and furan instead of the phenyl group of **2a′** were tolerable to afford **3ad** in 96:4 er and **3ae** in 91:9 er, the reaction with naphthalene-substituted carbonates (**2f**) gave lower enantioselectivity (76:24 er). The nonsubstituted allyl carbonate (**2g**) was found not to be compatible with our methodology. The absolute configuration of **3ea** was determined by X-ray crystallography (see Supplementary Data 1, Supplementary Fig. 1, and Supplementary Table 6−13).

### Reaction profile and secondary kinetic resolution effects

**Fig. 4:**
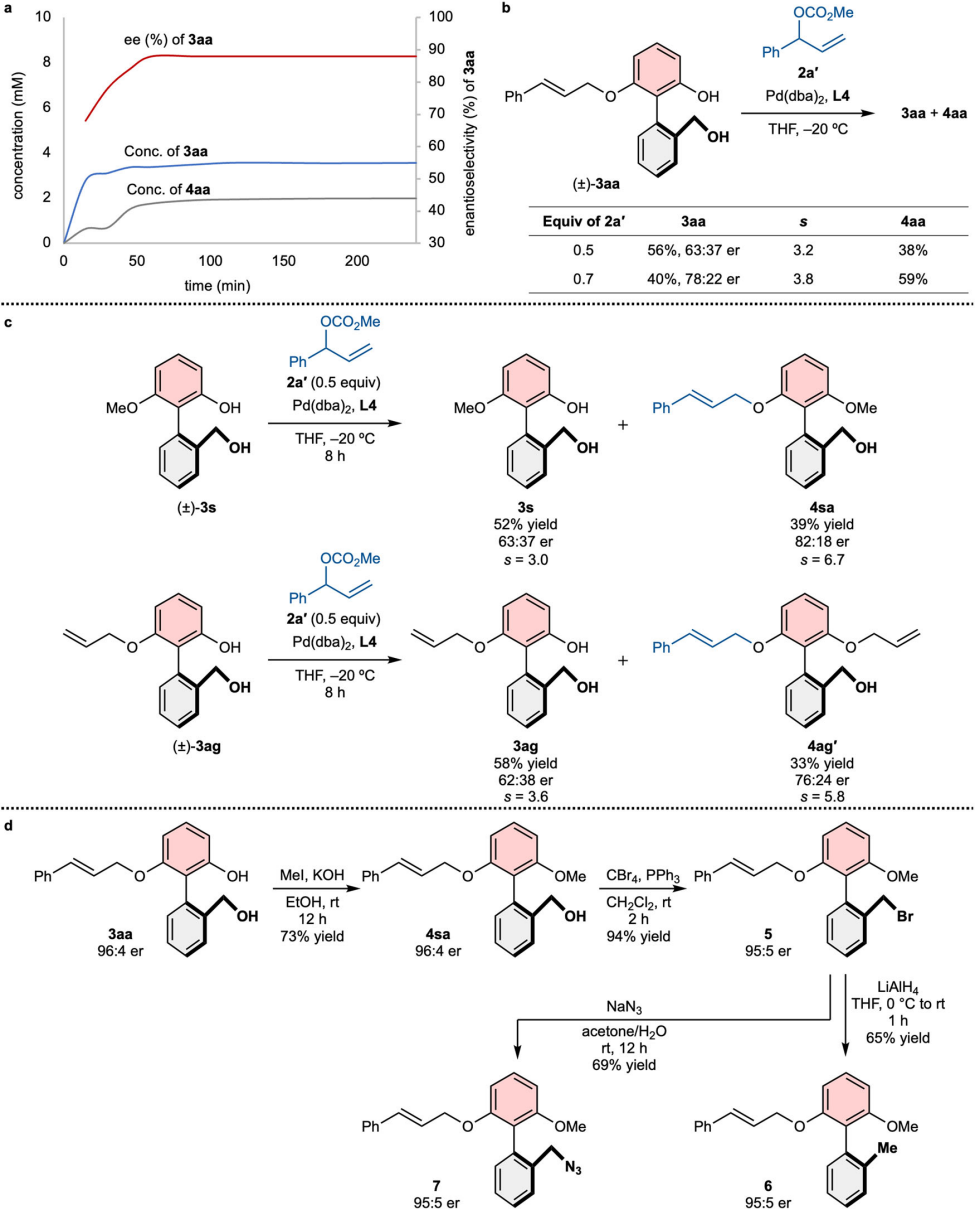
Reaction profile, kinetic resolution effects, and further transformations. **a** Reaction conversion and product distributions. **b** Secondary kinetic resolution effects of **3aa**. **c** Atroposelective allylation via kinetic resolution. **d** Further transformations.

Because difunctionalized products can be formed in desymmetrization, the moderate chemical yields of monofunctionalized products have been observed despite high overall yields^38^. Furthermore, because the formation of difunctionalized products is related to secondary kinetic resolution effect, careful investigations are required. To explore this issue more aggressively, the changes in **3aa** and **4aa** were examined in the reaction mixture (Fig. 4a). Interestingly, the desired product was quickly formed within 15 min, and the total amount of **3aa** was mostly unchanged. Instead, the amount of the diallylated product (**4aa**) and enantioselectivity of **3aa** increased until 45 min. This result suggests that the substrate (**1a**) and monosubstituted product (**3aa**) are allylated at a similar rate, and favorable secondary kinetic resolution is involved. Indeed, when the racemic mixture of **3aa** was reacted with 0.7 equiv of **2a′** under the optimized reaction conditions, the same atropisomer of **3aa** remained at 78:22 er (Fig. 4b). Because the fast initial rate could lead to an uncontrolled reaction in terms of product distribution and selectivity, we tried several milder reaction conditions, including lower concentrations, catalytic loading, and temperature, to decrease the reaction rate. However, these efforts were found to be unfruitful (see Supplementary Table 1−5).

### Kinetic resolution and further transformation

Inspired by secondary kinetic resolution effect, we envisioned atroposelective kinetic resolution of mono-substituted biaryls (Fig. 4c). Two racemic mixtures of **3s** and **3ag** substituted with a methyl or allyl group, respectively, were reacted with 0.5 equiv of **2a′** under the optimized reaction conditions to give chiral difunctionalized products with moderate enantioselectivities (**4sa**, 39% yield, 82:18 er; **4ag′**, 33% yield, 76:24 er). In these reactions, the substrates were recovered in non-racemic, but lower enantioselectivities (**3** **s**, 52% yield, 63:37 er; **3ag**, 58% yield, 62:38 er). These results suggest that atroposelective kinetic resolution would be feasible based on asymmetric Tsuji-Trost reaction.

In order to demonstrate the practicality of our method, the monofunctionalized product was further transformed. Even though the hydroxyl group at the bottom aromatic ring is required to achieve high atroposelectivity in this reaction, this hydroxyl group can be easily transformed to other functionalities which would be additional advantage of our methodology (Fig. 4d). Because the phenolic OH is highly reactive, selective methylation was initially conducted to provide the methylated product (**4sa**) in 73% yield and 96:4 er. Then, the hydroxyl group at the bottom aromatic ring was brominated to afford **5** in 94% yield and 95:5 er, which could be converted to many different functional groups. For example, the bromomethyl compound was reduced with LiAlH_4_ to give **6** in 95:5 er and underwent a substitution reaction with NaN_3_ to furnish **7** without loss of enantioselectivity. We believe that the azide **7** can be further transformed to a variety of atropisomeric amine compounds.

### Computational studies

**Fig. 5:**
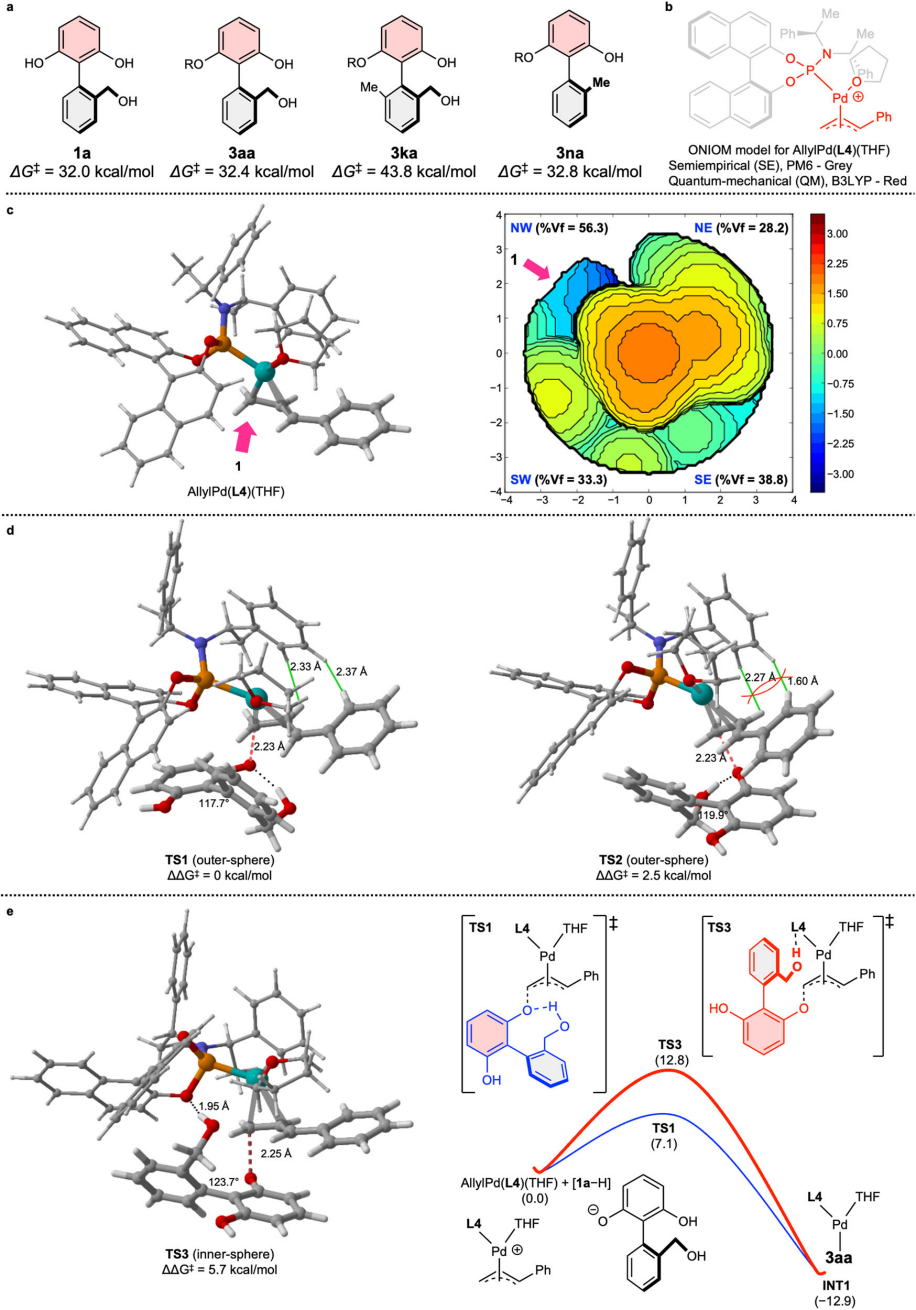
Computational studies. **a** Computed barriers to rotation about the C–C bond. **b** ONIOM model for AllylPd(**L4**)(THF). **c** Optimized geometry of the AllylPd(**L4**)(THF) complex and steric map. **d** Transition state structures of the outer-sphere models and computed relative energy profiles. **e** A transition state structure of the inner-sphere model and computed relative energy profiles.

To investigate the configurational stability of the products, we conducted computational calculations on the rotational barriers of **1a**, **3aa**, **3ka**, and **3na** as shown in Fig. 5a (See Supplementary Table 14−17 in Supplementary Data 4)^60^. Geometries/frequencies were computed at the B3LYP/6-31+G(d,p) level of theory, and the single point energies were calculated at the M06-2X/6-311++G(2d,3p) level of theory. In our calculations, the substrate and allylated products were expected to have a sufficiently high rotational barrier to lock their stereo-configurations at the reaction temperature. In particular, **3ka**, which contained another substituent at the *ortho* position to the stereogenic axis, showed a much higher rotational barrier (43.8 kcal/mol).

To further elucidate the origin of the observed enantioselectivity, we conducted preliminary computational studies on this transformation (See Supplementary Table 18 in Supplementary Data 4)^60^. Because of the bulky chiral ligand, the two-layer quantum-mechanical (QM)/semiempirical (SE) ONIOM model^61–63^ was applied to the palladium complexes. Heteroatoms in ligands, palladium atom, cinnamyl group, and **1a** were designated to the QM layer which was treated with B3LYP/6-31G(d) (C, H, O, N, P)/LANL2DZ (ECP Pd). All carbon and hydrogen atoms in ligands were designated to the SE layer which was treated with PM6 (Fig. 5b). Single-point energies of these optimized structures were calculated using M06-2X/6-311++G(2d,3p) (C, H, O, N, P)/SDD (ECP Pd) for the QM layer and PM6 for the SE layer with the inclusion of solvation energy corrections (SMD, tetrahydrofuran). Based on the optimized geometry of the π-allyl palladium complex (AllylPd(**L4**)(THF)), the steric effects of the ligands were quantitatively assessed using the steric map produced by the SambVca 2.1 tool^64^. The results clearly showed an accessible quadrant between the BINOL of **L4** and the cinnamyl group (Fig. 5c). With two enantiomers of anionic **1a**, possible transition states (**TS1**, **TS2**, and **TS3**) were obtained (Fig. 5d and Fig. 5e). In our calculations, **TS1**, which can afford the observed enantiomer, is more energetically favorable than **TS2** by 2.5 kcal/mol. In **TS2**, the bottom aromatic ring of 2-arylresorcinol pointed to the binaphthyl group in **L4**, which would make a slight turn clockwise around the Pd-P bond. We believe that this inevitable turn would cause unfavorable steric interaction (indicated as red lines in Fig. 5d) between the cinnamyl group and the phenyl group of **L4**. The noncovalent interaction (NCI) plots also showed this unfavorable interaction in **TS2** (See Supplementary Figure 2 in Supplementary Data 4)^65^. Additionally, these results suggested that the intramolecular hydrogen bond would stabilize the partially eclipsed conformation of 2-arylresorcinol in **TS1** and **TS2**. This effect would further improve enantioselectivity of **3aa** up to 98:2 er, compared to that of the non-hydroxymethyl substrates such as **3la**, **3na**−**3pa**. Interestingly, because the *para* position to the hydroxymethyl group oriented to the ligands in **TS1**, the substitution at this position could lead to unfavorable steric repulsion, which was in agreement with the observed result in **3ia** and **3ja**. Because the hydroxymethyl group could form an intermolecular hydrogen bond with the BINOL group of **L4**, the inductive model (**TS3**) was considered. However, this transition state (**TS3**) was energetically unfavorable by 5.7 kcal/mol compared to **TS1** (Fig. 5e).

## Conclusion

In conclusion, an efficient strategy for the highly atroposelective palladium-catalyzed desymmetrization of 2-arylresorcinols has been established. The chiral palladium complex with a phosphoramidite ligand smoothly induces the desymmetric allylic *O*-alkylation reaction with excellent enantioselectivities up to 98:2 er. Our calculations reveal that the hydroxymethyl group at the bottom aromatic ring forms an intramolecular hydrogen bond and facilitates the catalytic reaction. The transition states of this transformation have been obtained by computational calculations, which have provided insight into the origin of enantioselectivity. Given the importance of catalytic and atroposelective reactions, this unique and efficient methodology will encourage further efforts in this field.

## Methods

### General procedure for atroposelective allylation

In an oven dried reaction tube equipped with a magnetic stirring bar, were premixed Pd(dba)_2_ (0.6 mg, 0.001 mmol, 0.01 equiv), **L4** (2.2 mg, 0.004 mmol, 0.04 equiv), and THF (0.2 mL). After 10 min, **2** (0.15 mmol, 1.5 equiv) in THF (0.3 mL) was added, and the mixture was stirred for 10 min. Then, **1** (0.10 mmol, 1 equiv) was added and the vial was sealed with a Teflon cap and further secured with Parafilm M^Ⓡ^. The reaction mixture was left to stir for 10–240 h at –20 °C. After that, the crude material was purified by flash column chromatography using an eluent of 9–33% EtOAc/Hx to provide the desired product **3**. The enantioselectivity was determined by chiral HPLC.

## Supplementary information

Supplementary Information

Description of Additional Supplementary Files

Supplementary Data 1

Supplementary Data 2

Supplementary Data 3

Supplementary Data 4

## Data Availability

Detailed experimental procedures and characterizations of new compounds are available in Supplementary Information. The X-ray crystallographic coordinates for structures reported in this Article have been provided as Supplementary Data 1 and deposited at the Cambridge Crystallographic Data Centre (CCDC), under deposition numbers CCDC 701796. These data can be obtained free of charge from The Cambridge Crystallographic Data Centre via http://www.ccdc.cam.ac.uk/data_request/cif. ^1^H and ^13^C NMR spectra and HPLC chromatograms can be found in the Supplementary Data 2 and 3, respectively. Computational chemistry details are available in Supplementary Data 4. Reprints and permissions information is available online at www.nature.com/reprints. Correspondence and requests for materials should be addressed to Y.K.
