# Supplementary material for: Atroposelective desymmetrization of 2-arylresorcinols via Tsuji-Trost allylation
Source: Commun Chem. 2023 Feb 25;6:42. doi: 10.1038/s42004-023-00839-z (PMC9968306; doi:10.1038/s42004-023-00839-z)
Supplement: Supplementary file 1 — Supplementary Information [file 42004_2023_839_MOESM1_ESM.pdf]

Supplementary Information

Atroposelective Desymmetrization of 2-Arylresorcinols via Tsuji-Trost Allylation

Sangji Kim,[1,†] Aram Kim,[2,†] Chanhee Lee,[1] Junsoo Moon,[1] Eun Jeong Hong,[2] Duck-Hyung Lee,[2] Yongseok Kwon[1,*]

[1]School of Pharmacy, Sungkyunkwan University, Suwon 16419, Republic of Korea

[2]Department of Chemistry, Sogang University, Seoul 04107, Republic of Korea

[†]These authors contributed equally. *Email: y.kwon@skku.edu

1. Materials and Methods S2
2. Synthesis of Substrates 1 .... S4
2.1. Synthetic Route A for Substrate 1 .... S4
2.2. Synthetic Route B for Substrate 1 .... S15
2.3. Characterization of 1 .... S22
3. Reaction Optimizations and Procedures S30
3.1. Ligand Screening S30
3.2. Catalyst Screening S32
3.3. Allyl Electrophiles Screening S33
3.4. Temperature and Additive Screening S34
3.5. Equivalent Screening S35
4. Reaction Procedures for 3 and 4 .... S36
5. Characterization of Products S37
5.1. Characterization of Products 3 .... S37
5.2. Characterization of Products 4 .... S50
6. Kinetic Resolution Study S62
6.1. Synthesis of Substrates 3ag and 3s S62
6.2. Kinetic Resolution of 3ag S63
6.3. Kinetic Resolution of 3s S65
7. Further Transformations S67
8. Experiment for X-ray Crystallography S71
9. Supplementary References S79

Supplementary Methods

1 Materials and Methods

Room temperature is defined as 21–23 °C. All reagents including chiral ligand were purchased from commercial suppliers and used without further purification, unless otherwise noted. All solvents were purchased from commercial suppliers and used without further purification, unless otherwise noted.

Routine ^{1}H NMR spectra were recorded on Varian 400 MHz and Bruker 400 MHz spectrometers at ambient temperature unless otherwise stated. All NMR solvents were purchased from Cambridge Isotope Laboratories and used without further purification. Chloroform-*d*, methanol-d_4 and dimethylsulfoxide-d_6 was stored at ambient temperature. Spectra were processed using MestReNova 6.0.2 using the automatic phasing and polynomial baseline correction capabilities. Splitting was determined using the automatic multiplet analysis function with manual intervention as necessary. Spectral data are reported as follows: chemical shift (multiplicity [singlet (s), broad singlet (brs), doublet (d), triplet (t), quartet (q), pentet (p), multiplet (m), doublet of doublets (dd), doublet of doublet of doublets (ddd), doublet of triplet of doublets (dtd), doublet of doublet of doublet of doublets (dddd), doublet of triplets (dt), triplet of doublets (td), etc.], coupling constant, integration). Chemical shifts are reported in ppm (δ) and coupling constants are reported in Hz. ^{1}H Resonances are referenced to solvent residual peaks for $CDCl_3$ (7.26 ppm), CD_3OD (3.31 ppm) or $(CD_3)_2SO$ (2.50 ppm).[1] Routine ^{13}C NMR spectra were recorded on Varian 400 MHz or Bruker 400 MHz spectrometers with protons fully decoupled. ^{13}C Resonances are reported in ppm relative to solvent residual peaks for $CDCl_3$ (77.16 ppm), CD_3OD (49.00 ppm) or $(CD_3)_2SO$ (39.52 ppm).[1] Note: Small deviations in chemical shifts may be observed depending on the concentration of NMR samples.

Infrared spectra were recorded on a JASCO FT/IR-4600 spectrometer and ν_{max} are partially reported in cm^{-1}. High-resolution mass spectra were acquired on a Thermo-Fischer Scientific LTQ Orbitrap XL instrument or a JEOL JMS-700 instrument with an ESI or EI mode. Analytical thin-layer chromatography was performed using 60 Å Silica Gel F_{254} pre-coated plates (0.25 mm thickness). TLC plates were visualized by irradiation with a UV lamp. Normal-phase column chromatography was performed using 60 Å Silica Gel (32–62 micron) with an appropriate mobile phase composition and gradient. Optical rotations were recorded on a JASCO P-2000 at the sodium D-line (589 nm) using a cell of 1 dm path length. Concentration values are reported in units of g/100 mL. Normal-phase high-performance liquid chromatography was performed using an Agilent 1260 series instrument equipped with a diode array detector and columns (chiral supports) from Daicel Chemical Industries.

Abbreviation

DME	1,2-Dimethoxyethane
DMSO	Dimethyl sulfoxide
DIPEA	*N*,*N*-Diisopropylethylamine
EI	Electron ionization
ESI	Electrospray ionization
EtOAc	Ethyl acetate
HPLC	High-performance liquid chromatography
HRMS	High-resolution mass spectrometry
Hx	Hexanes
rt	Room temperature
MOM	Methoxy methyl
M.S.	Molecular sieves
TBAF	*t*-Butylammonium fluoride
TBS	*t*-Butyldimethylsilyl
THF	Tetrahydrofuran
TLC	Thin-layer chromatography

2 Synthesis of Substrates 1

Substrates were synthesized via two routes (Route A and B) as shown below.

Route A

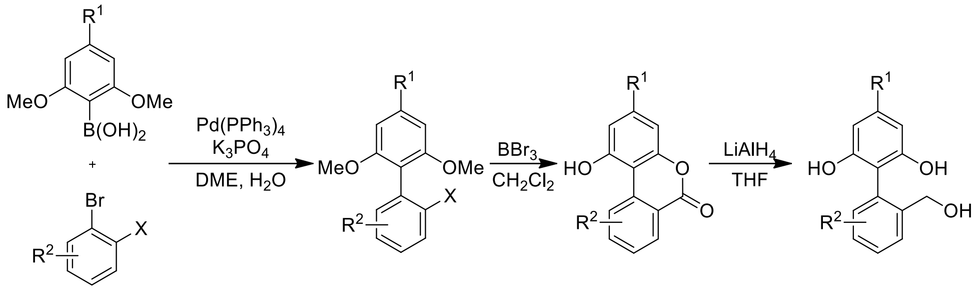

Route B

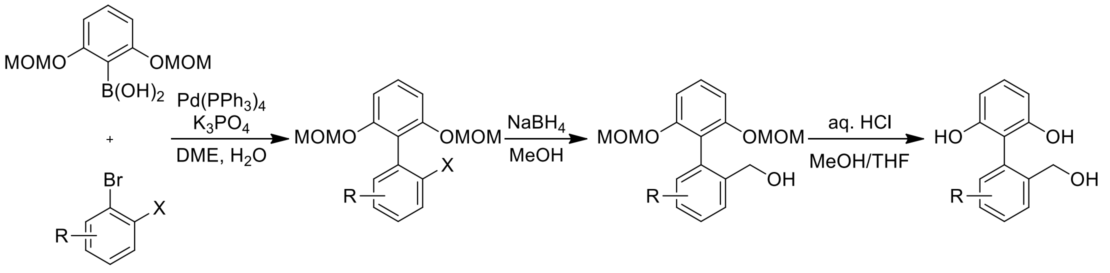

2.1 Synthetic Route A for Substrate 1

2.1.1 Synthesis of 2,6-Dimethoxybiphenyl (S1–9)

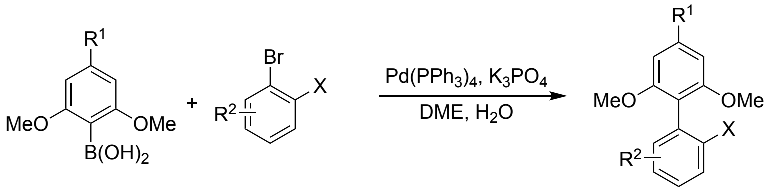

S1–3, **S8**, and **S9** (X = CO_2Me)
S4 (X = CHO)
S5 (X = Me)
S6 (X = ^{i}Pr)
S7 (X = Ph)

Procedure 1: To a round-bottom flask equipped with a magnetic stirring bar, 2,6-dimethoxybenzeneboronic acid (1.2 equiv), arylhalide (1.0 equiv), $Pd(PPh_3)_4$ (0.15 equiv) and K_3PO_4 (3.0 equiv) were added and subsequently dissolved in DME (0.1 M) and H_2O (0.3 M). The reaction was heated to 95 °C and allowed to stir for 3 h. The reaction was diluted with EtOAc, transferred to a separatory funnel and quenched with H_2O. The organic and aqueous layers were separated and the aqueous layer was extracted an additional two times with EtOAc, dried with anhydrous $MgSO_4$, filtered and concentrated *in vacuo*. The crude material was then purified by flash chromatography to afford the desired materials.

Characterization of 2,6-Dimethoxybiphenyl (S1–9)

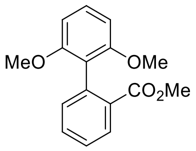

Methyl 2',6'-Dimethoxy-[1,1'-biphenyl]-2-carboxylate (S1) was synthesized by following Procedure 1. The crude material was purified by normal-phase column chromatography using an eluent of 17% EtOAc/Hx to provide **S1** (755 mg, 99%). The spectral data were identical with those previously reported.[2]

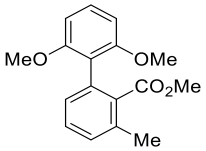

Methyl 2',6'-Dimethoxy-3-methyl-[1,1'-biphenyl]-2-carboxylate (S2) was synthesized by following Procedure 1. The crude material was purified by normal-phase column chromatography using an eluent of 17% EtOAc/Hx to provide **S2** (494 mg, 79%).

^{1}H NMR (400 MHz, $CDCl_3$) δ 7.38 (t, *J* = 7.6 Hz, 1H), 7.28 (t, *J* = 8.0 Hz, 1H), 7.22 (d, *J* = 7.6 Hz, 1H), 7.15 (d, *J* = 7.6 Hz, 1H), 6.63 (d, *J* = 8.4 Hz, 2H), 3.73 (s, 6H), 3.52 (s, 3H), 2.45 (s, 3H).
^{13}C NMR (100 MHz, $CDCl_3$) δ 169.5, 157.6 (2C), 135.9, 133.9, 133.5, 129.5, 129.4, 129.2, 129.1, 118.6, 104.0 (2C), 56.0 (2C), 51.2, 20.5.
IR (FT-ATR, cm^{-1}, CH_2Cl_2) ν_{max} 2945, 2836, 1724, 1588, 1471, 1432, 1249, 1187, 1172, 1106, 1075, 1062, 1035, 960, 872, 826, 781, 749, 732, 702, 607
HRMS: Exact mass calculated for $[C_{17}H_{18}O_4+H]^+$ requires *m*/*z* = 309.1103, found *m*/*z* = 309.1097 (ESI+).

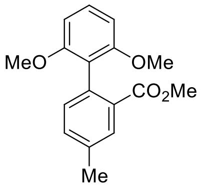

Methyl 2',6'-Dimethoxy-4-methyl-[1,1'-biphenyl]-2-carboxylate (S3) was synthesized by following Procedure 1. The crude material was purified by normal-phase column chromatography using an eluent of 33% EtOAc/Hx to provide **S3** (542 mg, 87%).

^{1}H NMR (400 MHz, $CDCl_3$) δ 7.76 (s, 1H), 7.35 (d, *J* = 7.7 Hz, 2H), 7.30–7.24 (m, 3H), 7.21 (d, *J* = 7.8 Hz, 1H), 6.63 (d, *J* = 8.3 Hz, 2H), 3.70 (s, 6H), 3.62 (s, 3H), 2.41 (s, 3H).
^{13}C NMR (100 MHz, $CDCl_3$) δ 168.4, 157.2 (2C), 136.8, 132.4, 132.2, 132.1, 131.4, 130.4, 128.7, 119.0, 104.1 (2C), 56.0 (2C), 51.7, 21.3.
IR (FT-ATR, cm^{-1}, CH_2Cl_2) ν_{max} 2947, 2836, 1729, 1590, 1508 1470, 1433, 1296, 1241, 1203, 1145, 1108, 1086, 1037, 1004, 977, 893, 829, 793, 782, 734, 543.

HRMS: Exact mass calculated for $[C_{17}H_{18}O_4+H]^+$ requires m/z = 287.1283, found m/z = 287.1278 (ESI+).

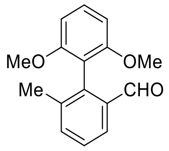

2',6'-Dimethoxy-6-methyl-[1,1'-biphenyl]-2-carbaldehyde (S4) was synthesized by following Procedure 1. The crude material was purified by normal-phase column chromatography using an eluent of 13% EtOAc/Hx to provide **S4** (1.33 g, 72%).

^{1}H NMR (400 MHz, $CDCl_3$) δ 9.65 (s, 1H), 7.85 (d, J = 7.7 Hz, 1H), 7.53 (d, J = 7.4 Hz, 1H), 7.38 (t, J = 8.4 Hz, 2H), 6.67 (d, J = 8.4 Hz, 2H), 3.70 (d, J = 1.4 Hz, 6H), 2.07 (s, 3H).
^{13}C NMR (100 MHz, $CDCl_3$) δ 193.7, 157.8 (2C), 138.7, 138.5, 135.4, 134.5, 130.1, 127.6, 124.3, 113.2, 103.8 (2C), 55.9 (2C), 19.5.
IR (FT-ATR, cm^{-1}, CH_2Cl_2) ν_{max} 2939, 2837, 2744, 1687, 1587, 1470, 1432, 1388, 1299, 1284, 1247, 1172, 1104, 1033, 999, 921, 781, 747, 726, 701, 610, 567, 542, 527.
HRMS: Exact mass calculated for $[C_{16}H_{16}O_3+H]^+$ requires m/z = 287.1283 found m/z = 287.1278 (ESI+).

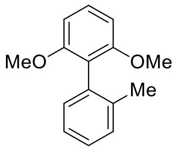

Methyl 2',6'-Dimethoxy-[1,1'-biphenyl]-2-carboxylate (S5) was synthesized by following Procedure 1. The crude material was purified by normal-phase column chromatography using an eluent of 14% EtOAc/Hx to provide **S5** (2.68 g, 100%). The spectral data were identical with those previously reported.[3]

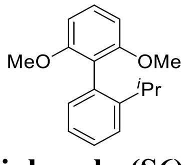

2'-Isopropyl-2,6-dimethoxy-1,1'-biphenyl (S6) was synthesized by following Procedure 1. The crude material was purified by normal-phase column chromatography using an eluent of 10% EtOAc/Hx to provide **S6** (180 mg, 38%). The spectral data were identical with those previously reported.[3]

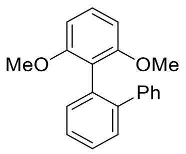

2,6-Dimethoxy-1,1':2',1''-terphenyl (S7) was synthesized by following Procedure 1. The crude material was purified by normal-phase column chromatography using an eluent of 10% EtOAc/Hx to provide **S7** (538 mg, 100%). The spectral data were identical with those previously reported.[3]

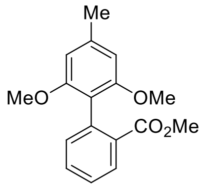

Methyl 2',6'-Dimethoxy-4'-methyl-[1,1'-biphenyl]-2-carboxylate (S8) was synthesized by following Procedure 1. The crude material was purified by normal-phase column chromatography using an eluent of 33% EtOAc/Hx to provide **S8** (741 mg, 100%). The spectral data were identical with those previously reported.[4]

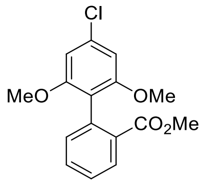

Methyl 4'-Chloro-2',6'-dimethoxy-[1,1'-biphenyl]-2-carboxylate (S9) was synthesized by following Procedure 1. The crude material was purified by normal-phase column chromatography using an eluent of 33% EtOAc/Hx to provide **S9** (399 mg, 92%).

^{1}H NMR (400 MHz, $CDCl_3$) δ 7.96 (d, J = 7.8 Hz, 1H), 7.53 (t, J = 7.4 Hz, 1H), 7.40 (t, J = 7.8 Hz, 1H), 7.27 (s, 1H), 6.64 (d, J = 2.2 Hz, 2H), 3.69 (s, 6H), 3.66 (s, 3H).
^{13}C NMR (100 MHz, $CDCl_3$) δ 167.9, 157.4 (2C), 134.3, 134.2, 132.5, 131.49, 131.45, 129.9, 127.4, 117.6, 105.1 (2C), 56.1 (2C), 51.8.
IR (FT-ATR, cm^{-1}, CH_2Cl_2) ν_{max} 2948, 2836, 1724, 1582, 1454, 1441, 1401, 1288, 1278, 1258, 1221 1190, 1119, 1085, 1046, 1003, 965, 920, 866, 815, 770, 733, 711, 659, 592, 538, 482.
HRMS: Exact mass calculated for $[C_{16}H_{15}ClO_4+Na]^+$ requires m/z = 329.0557, found m/z = 392.0551 (ESI+).

2.1.2 Synthesis of 2',6'-Dimethoxy-6-methyl-[1,1'-biphenyl]-2-carboxylic Acid (S10)

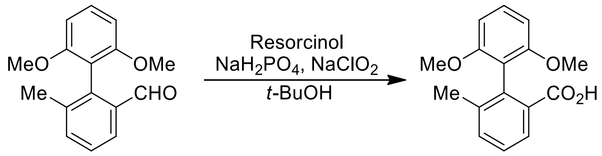

S4 **S10**

2',6'-Dimethoxy-6-methyl-[1,1'-biphenyl]-2-carboxylic Acid (S10) To a round-bottom flask equipped with a magnetic stirring bar, **S4** (1.0 equiv), resorcinol (1.3 equiv) were added and subsequently dissolved in *t*-BuOH (0.05 M) through gentle heating to 70 °C. The solution was cooled to rt and a solution of NaH_2PO_4 (3.0 equiv) and $NaClO_2$ (6.0 equiv) in deionized H_2O was added dropsied to the flask over a period of 10 min. The reaction was allowed to stir for overnight. The reaction was diluted with EtOAc, transferred to a separatory funnel and quenched with H_2O. The organic and aqueous layers were separated and the aqueous layer was extracted an additional two times with EtOAc, dried with anhydrous $MgSO_4$, filtered and concentrated *in vacuo*. The crude material was purified by normal-phase column chromatography using an eluent of 25% Hx/EtOAc to provide **S10** (1.20 g, 87%).

^{1}H NMR (400 MHz, $CDCl_3$) δ 7.85 (d, J = 7.2 Hz, 1H), 7.46 (d, J = 7.7 Hz, 1H), 7.32 (t, J = 8.2 Hz, 2H), 6.63 (d, J = 8.3 Hz, 2H), 3.69 (s, 6H), 2.03 (s, 3H).
^{13}C NMR (100 MHz, $CDCl_3$) δ 172.7, 157.1 (2C), 138.8, 135.4, 134.1, 130.7, 129.0, 128.2, 127.1, 117.3, 104.2 (2C), 56.0 (2C), 20.1.
IR (FT-ATR, cm^{-1}, CH_2Cl_2) ν_{max} 2937, 2837, 1690, 1589, 1470, 1432, 1284, 1248, 1182, 1154, 1106, 1035, 1003, 970, 908, 846, 783, 763, 732, 697, 645, 620, 592, 543, 436.
HRMS: Exact mass calculated for $[C_{16}H_{16}O_4+H]^+$ requires *m/z* = 287.1283, found *m/z* = 287.1278 (ESI+).

2.1.3 Synthetic of Lactones (S11–13)

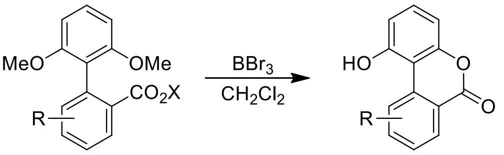

Procedure 2: To a round-bottom flask equipped with a magnetic stirring bar, **S1**, **S2** or **S10** (1.0 equiv) was added and subsequently dissolved in CH_2Cl_2 (0.08 M). The reaction was cooled to −78 °C and added boron tribromide (3.0 equiv) dropwised to the flask. Then the reaction was allowed to stir for 3 h. The reaction was diluted with CH_2Cl_2 and quenched with a saturated aqueous $NaHCO_3$. Transferred to a separatory funnel, the organic and aqueous layers were separated, extracted with CH_2Cl_2 and aqueous HCl, dried with anhydrous $MgSO_4$, filtered and concentrated *in vacuo*. The crude material was then purified by flash chromatography to afford the desired materials.

Characterization of Lactones (S11–13)

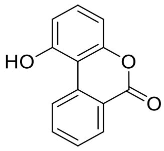

1-Hydroxy-6*H*-benzo[*c*]chromen-6-one (S11) was synthesized by following Procedure 2. The crude material was purified by normal-phase column chromatography using an eluent of 33% EtOAc/Hx to provide **S11** (1.51 g, 98%). The spectral data were identical with those previously reported.[5]

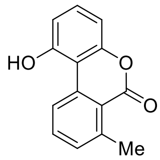

1-Hydroxy-7-methyl-6*H*-benzo[*c*]chromen-6-one (S12) was synthesized by following Procedure 2. The crude material was purified by normal-phase column chromatography using an eluent of 33% EtOAc/Hx to provide **S12** (287 mg, 91%).

^{1}H NMR (400 MHz, DMSO-*d₆*) δ 9.14 (d, *J* = 8.0 Hz, 1H), 7.75 (t, *J* = 7.9 Hz, 1H), 7.44 (d, *J* = 7.4 Hz, 1H), 7.30 (t, *J* = 8.2 Hz, 1H), 6.88 (d, *J* = 8.1 Hz, 1H), 6.80 (d, *J* = 8.1 Hz, 1H), 2.76 (s, 3H).
^{13}C NMR (100 MHz, DMSO-d_6) δ 159.6, 156.5, 152.2, 142.5, 136.1, 134.1, 131.3, 130.0, 125.2, 118.5, 111.9, 107.3, 106.3, 23.8.
IR (FT-ATR, cm^{-1}, CH_2Cl_2) ν_{max} 3267, 3124, 2925, 1698, 1618, 1598, 1506, 1479, 1467, 1440, 1403, 1380, 1358, 1308, 1292, 1249, 1218, 1186, 1130, 1087, 1056, 1029, 806, 790, 774, 723, 688, 646, 596, 583, 532, 506, 494, 461
HRMS: Exact mass calculated for $[C_{14}H_{10}O_3-H]^-$ requires *m*/*z* = 225.0552, found *m*/*z* = 225.0557 (ESI−).

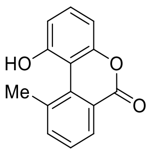

1-Hydroxy-10-methyl-6*H*-benzo[*c*]chromen-6-one (S13) was synthesized by following Procedure 2. The crude material was purified by normal-phase column chromatography using an eluent of 33% EtOAc/Hx to provide **S13** (707 mg, 77%).

^{1}H NMR (400 MHz, DMSO-d_6) δ 10.73 (s, 1H), 8.03 (d, *J* = 7.6 Hz, 1H), 7.71 (d, *J* = 7.4 Hz, 1H), 7.52 (t, *J* = 7.5 Hz, 1H), 7.35 (t, *J* = 8.1 Hz, 1H), 6.86 (t, *J* = 9.0 Hz, 2H), 2.45 (s, 3H).
^{13}C NMR (100 MHz, DMSO-d_6) δ 161.1, 155.1, 151.5, 137.5, 136.7, 132.9, 130.2, 127.6, 126.5, 122.7, 112.0, 107.5, 106.7, 23.2.
IR (FT-ATR, cm^{-1}, CH_2Cl_2) ν_{max} 3322, 2925, 2852, 1699, 1591, 1494, 1477, 1459, 1433, 1378, 1344, 1277, 1226, 1176, 1128, 1094, 1075, 1052, 1004, 971, 899, 851, 787, 763, 727, 642, 586, 533, 511, 464, 455, 433
HRMS: Exact mass calculated for $[C_{14}H_{10}O_3+H]^+$ requires *m*/*z* = 225.0552, found *m*/*z* = 225.0557 (ESI+).

2.1.4 Synthesis of 1-((*tert*-butyldimethylsilyl)oxy)-6*H*-benzo[*c*]chromen-6-one (S14)

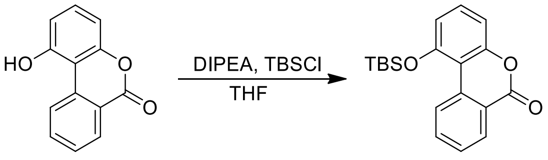

S11 **S14**

1-((*tert*-Butyldimethylsilyl)oxy)-6*H*-benzo[*c*]chromen-6-one (S14) To a round-bottom flask equipped with a magnetic stirring bar and a refluxing condenser, **S11** (1.0 equiv) and *N*,*N*-diisopropylethylamine (3.0 equiv) were added and subsequently dissolved in THF (0.07 M). To the reaction mixture, TBSCl (1.1 equiv) was added and the reaction was allowed to stir for 2 h at rt. After removing the solvent *in vacuo*, the residue was diluted with EtOAc, quenched with a saturated aqueous NH_4Cl and transferred to a separatory funnel. The organic and aqueous layers were separated and the aqueous layer was extracted with EtOAc. The combined organic layers were dried with anhydrous $MgSO_4$, filtered and concentrated *in vacuo*. The crude material was purified by normal-phase column chromatography using an eluent of 13% EtOAc/Hx to provide **S14** (782 mg, 94%).

^{1}H NMR (400 MHz, $CDCl_3$) δ 9.08 (d, J = 8.4 Hz, 1H), 8.42 (d, J = 7.9 Hz, 1H), 7.76 (dd, J = 11.3, 4.3 Hz, 1H), 7.55 (t, J = 7.6 Hz, 1H), 7.29 (t, J = 8.2 Hz, 1H), 7.01 (d, J = 8.2 Hz, 1H), 6.81 (d, J = 8.2 Hz, 1H), 1.05 (s, 9H), 0.36 (s, 6H).
^{13}C NMR (100 MHz, $CDCl_3$) δ 161.5, 154.5, 152.9, 134.8, 134.3, 130.3, 129.5, 128.2, 127.2, 121.1, 115.8, 110.8, 110.4, 26.2 (3C), 18.8, −3.5 (2C).
IR (FT-ATR, cm^{-1}, CH_2Cl_2) ν_{max} 2962, 2927, 2884, 2858, 1722, 1604, 1591, 1492, 1470, 1455, 1429, 1392, 1312, 1302, 1289, 1257, 1240, 1224, 1121, 1093, 1056, 1033, 1003, 874, 843, 826, 813, 789, 742, 713, 681, 665, 639, 557.
HRMS: Exact mass calculated for $[C_{19}H_{23}O_3Si+Na]^+$ requires m/z = 349.1236, found m/z = 349.1230 (ESI+).

2.1.5 Synthesis of 6-((*tert*-Butyldimethylsilyl)oxy)-2'-(2-hydroxypropan-2-yl)-[1,1'-biphenyl]-2-ol (S15)

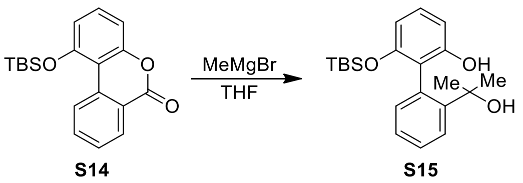

6-((*tert*-Butyldimethylsilyl)oxy)-2'-(2-hydroxypropan-2-yl)-[1,1'-biphenyl]-2-ol (S15) To a round-bottom flask equipped with a magnetic stirring bar and a refluxing condenser, **S14** (1.0 equiv) was added and subsequently dissolved in THF (0.5 M). To the reaction mixture, MeMgBr (3.0 equiv, 3 M in ether) was added at 0 °C and the reaction was allowed to stir for overnight at rt. After removing the solvent *in vacuo*, the residue was diluted with EtOAc, quenched with a saturated aqueous NH_4Cl and transferred to a separatory funnel. The organic aqueous layers were separated and the aqueous layer was extracted with EtOAc. The combined organic layers were dried with anhydrous $MgSO_4$, filtered and concentrated *in vacuo*. The crude material was purified by normal-phase column chromatography using an eluent of 33% EtOAc/Hx to provide **S15** (46 mg, 85%).

^{1}H NMR (400 MHz, $CDCl_3$) δ 7.63 (d, *J* = 7.8 Hz, 1H), 7.33 (dt, *J* = 22.0, 7.4 Hz, 2H), 7.13 (t, *J* = 8.2 Hz, 1H), 7.03 (d, *J* = 7.4 Hz, 1H), 6.63 (d, *J* = 8.2 Hz, 1H), 6.49 (d, *J* = 8.1 Hz, 1H), 5.14 (s, 1H), 2.80 (s, 1H), 1.49 (d, *J* = 8.0 Hz, 6H), 0.62 (s, 9H), 0.18 (s, 3H), −0.06 (s, 3H).
^{13}C NMR (100 MHz, $CDCl_3$) δ 154.2, 153.2, 148.1, 133.6, 130.7, 129.0, 128.6, 127.7, 126.6, 122.9, 111.2, 109.2, 73.4, 31.1, 30.9, 25.3 (3C), 17.8, −3.7, −4.8.
IR (FT-ATR, cm^{-1}, CH_2Cl_2) ν_{max} 3053, 2929, 2866, 1608, 1580, 1504, 1458, 1386, 1361, 1333, 1296, 1263, 1244, 1180, 1152, 1114, 1097, 1044, 1005, 954, 941, 852, 837, 797, 781, 705, 675, 654, 622, 589, 567, 942, 520, 467.
HRMS: Exact mass calculated for $[C_{21}H_{31}O_3Si{-}H]^-$ requires *m*/*z* = 357.1884, found *m*/*z* = 357.1890 (ESI–).

2.1.6 Synthesis of Substrate 1a, 1b and 1k

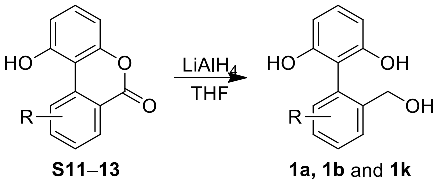

Procedure 3: To a round-bottom flask equipped with a magnetic stirring bar, **S11–13** (1.0 equiv) was added and subsequently dissolved in THF (0.2 M). To a solution $LiAlH_4$ (2.0 equiv) was added at 0 °C. The reaction was allowed to stir for 6 h. The reaction was diluted with EtOAc and quenched with a saturated aqueous HCl. The organic and aqueous layers were separated and the aqueous layer was extracted an additional two times with EtOAc. The combined organic layers were then rinsed with water, dried with anhydrous $MgSO_4$, filtered and concentrated *in vacuo*. The crude material was then purified by flash chromatography to afford the desired materials.

2.1.7 Synthesis of Substrate 1d, 1q and 1r

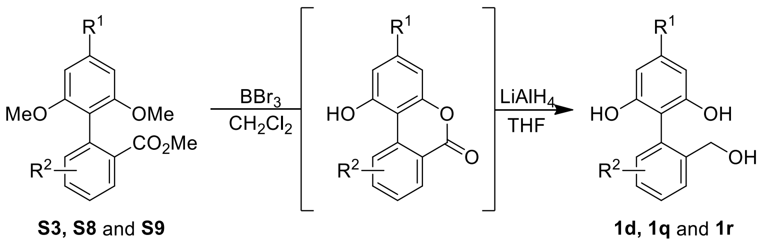

Procedure 4: To a round-bottom flask equipped with a magnetic stirring bar, **S3**, **S8** or **S9** (1.0 equiv) was added and subsequently dissolved in CH_2Cl_2 (0.08 M). The reaction was cooled to −78 °C and added boron tribromide (3.0 equiv) dropwised to the flask. Then the reaction was allowed to stir for 3 h. The reaction was diluted with CH_2Cl_2 and quenched with a saturated aqueous $NaHCO_3$. Transferred to a separatory funnel, the organic and aqueous layers were separated, extracted with CH_2Cl_2 and aqueous HCl, dried with anhydrous $MgSO_4$, filtered and concentrated *in vacuo*. This material was used for the next reaction without further purification.

To a round-bottom flask equipped with a magnetic stirring bar, the crude material (1.0 equiv) was added and subsequently dissolved in THF (0.2 M). To a solution $LiAlH_4$ (2.0 equiv) was added at 0 °C. The reaction was allowed to stir for 6 h. The reaction was diluted with EtOAc and quenched with a saturated aqueous HCl. The organic and aqueous layers were separated and the aqueous layer was extracted an additional two times with EtOAc. The combined organic layers were then rinsed with water, dried with anhydrous $MgSO_4$, filtered and concentrated *in vacuo*. The crude material was then purified by flash chromatography to afford the desired materials.

2.1.8 Synthesis of Substrate 1m

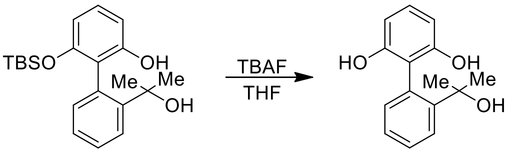

Procedure 5: To a round-bottom flask equipped with a magnetic stirring bar and a refluxing condenser, **S15** (1.0 equiv) was added and subsequently dissolved in THF (0.02 M). To the reaction mixture, TBAF (5.0 equiv, 1 M in THF) was added and the reaction was allowed to stir for 1 h at rt. After removing the solvent *in vacuo*, the residue was diluted with EtOAc and transferred to a separatory funnel. The organic and aqueous layers were separated and the aqueous layer was extracted with EtOAc. The combined organic layers were dried with anhydrous $MgSO_4$, filtered and concentrated *in vacuo*. The crude material was then purified by flash chromatography to afford the desired material.

2.1.9 Synthesis of Substrate 1n–p

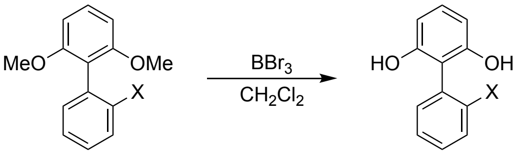

Procedure 6: To a round-bottom flask equipped with a magnetic stirring bar, **S5−7** (1.0 equiv) was added and subsequently dissolved in CH_2Cl_2 (0.08 M). The reaction was cooled to −78 °C and added boron tribromide (3.0 equiv) dropwised to the flask. Then the reaction was allowed to stir for 3 h. The reaction was diluted with CH_2Cl_2 and quenched with a saturated aqueous $NaHCO_3$. Transferred to a separatory funnel, the organic and aqueous layers were separated, extracted with CH_2Cl_2 and aqueous HCl, dried with anhydrous $MgSO_4$, filtered and concentrated *in vacuo*. The crude material was then purified by flash chromatography to afford the desired materials.

2.2 Synthetic Route B for Substrate 1

2.2.1 Synthesis of 2,6-MOM-Protected Dihydroxybiphenyls (S16–23)

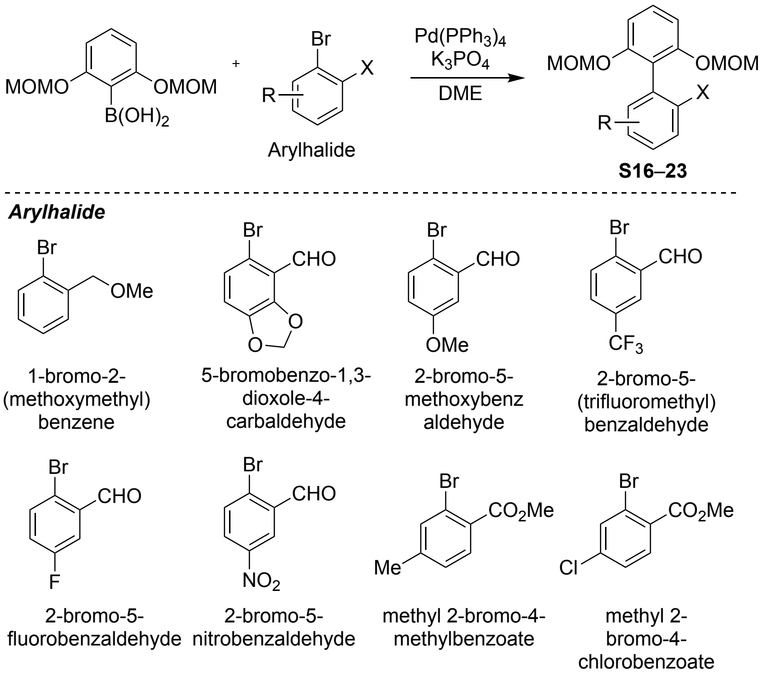

Procedure 7: To a round-bottom flask equipped with a magnetic stirring bar, phenylboronic acid (1.2 equiv), arylhalide (1.0 equiv), $Pd(PPh_3)_4$ (0.15 equiv) and K_3PO_4 (3.0 equiv) were added and subsequently dissolved in DME (0.1 M) and H_2O (0.3 M). The reaction was heated to 95 °C and allowed to stir for 3 h. The reaction was diluted with EtOAc, transferred to a separatory funnel and quenched with H_2O. The organic and aqueous layers were separated and the aqueous layer was extracted an additional two times with EtOAc, dried with anhydrous $MgSO_4$, filtered and concentrated *in vacuo*. The crude material was then purified by flash chromatography to afford the desired material.

Characterization of 2,6-MOM-Protected Dihydroxybiphenyls (S16–23)

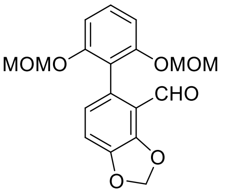

5-(2,6-Bis(methoxymethoxy)phenyl)benzo[*d*][1,3]dioxole-4-carbaldehyde (S16) was synthesized by following Procedure 7. The crude material was purified by normal-phase column chromatography using an eluent of 33% EtOAc/Hx to provide **S16** (574 mg, 95%).

^{1}H NMR (400 MHz, $CDCl_3$) δ 9.68 (s, 1H), 7.32–7.27 (m, 1H), 7.05 (d, *J* = 8.0 Hz, 1H), 6.89 (d, *J* = 8.4 Hz, 2H), 6.78 (d, *J* = 8.0 Hz, 1H), 6.19 (s, 2H), 5.06 (s, 4H), 3.31 (s, 6H).
^{13}C NMR (100 MHz, $CDCl_3$) δ 190.9, 155.8 (2C), 148.1, 147.6, 130.4, 130.0, 124.9, 118.6, 117.0, 112.7, 108.4, 102.8 (2C), 94.7 (2C), 56.3 (2C).
IR (FT-ATR, cm^{-1}, CH_2Cl_2) ν_{max} 2902, 1694, 1623, 1595, 1458, 1440, 1398, 1343, 1310, 1236, 1206, 1152, 1097, 1082, 1036, 922, 899, 822, 788, 745, 704, 672, 633, 605, 544, 522.
HRMS: Exact mass calculated for $[C_{18}H_{19}O_7+Na]^+$ requires *m*/*z* = 369.0950, found *m*/*z* = 369.0944 (ESI+).

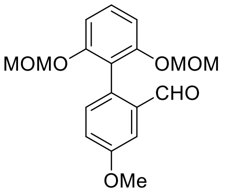

4-Methoxy-2',6'-bis(methoxymethoxy)-[1,1'-biphenyl]-2-carbaldehyde (S17) was synthesized by following Procedure 7. The crude material was purified by normal-phase column chromatography using an eluent of 33% EtOAc/Hx to provide **S17** (555 mg, 97%).

^{1}H NMR (400 MHz, $CDCl_3$) δ 9.76 (s, 1H), 7.52 (s, 1H), 7.29 (dd, *J* = 17.6, 8.5 Hz, 2H), 7.21 (dd, *J* = 8.5, 2.4 Hz, 1H), 6.91 (d, *J* = 8.4 Hz, 2H), 5.04 (s, 4H), 3.90 (s, 3H), 3.28 (s, 6H).
^{13}C NMR (100 MHz, $CDCl_3$) δ 192.7, 159.1, 155.8 (2C), 135.3, 133.5, 131.1, 130.0, 121.3, 117.1, 109.1, 108.6 (2C), 94.8 (2C), 56.3 (2C), 55.6.
IR (FT-ATR, cm^{-1}, CH_2Cl_2) ν_{max} 2956, 2845, 1692, 1596, 1504, 1464, 1441, 1397, 1312, 1269, 1247, 1224, 1204, 1154, 1097, 1081, 1039, 922, 897, 834, 789, 730
HRMS: Exact mass calculated for $[C_{18}H_{21}O_6+H]^+$ requires *m*/*z* = 355.1158, found *m*/*z* = 355.1152 (ESI+).

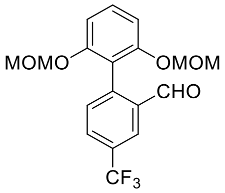

2',6'-Bis(methoxymethoxy)-4-(trifluoromethyl)-[1,1'-biphenyl]-2-carbaldehyde (S18) was synthesized by following Procedure 7. The crude material was purified by normal-phase column chromatography using an eluent of 17% EtOAc/Hx to provide **S18** (148 mg, 100%).

^{1}H NMR (400 MHz, $CDCl_3$) δ 9.82 (s, 1H), 8.29 (s, 1H), 7.86 (d, *J* = 8.0 Hz, 1H), 7.50 (d, *J* = 8.1 Hz, 1H), 7.36 (t, *J* = 8.4 Hz, 1H), 6.94 (d, *J* = 8.4 Hz, 2H), 5.06 (s, 4H), 3.29 (s, 6H).
^{13}C NMR (100 MHz, $CDCl_3$) δ 191.5, 155.4 (2C), 145.5, 145.0, 141.8, 134.8, 133.2, 131.0, 130.1 (d, *J* = 33.3 Hz), 129.5 (q, *J* = 3.3 Hz), 123.9 (d, *J* = 271.1 Hz), 123.8 (d, *J* = 3.7 Hz), 115.7, 108.4 (2C), 94.7 (2C), 56.4 (2C).
IR (FT-ATR, cm^{-1}, CH_2Cl_2) ν_{max} 2904, 1700, 1616, 1596, 1465, 1442, 1401, 1331, 1243, 1154, 1124, 1097, 1074, 1032, 919, 895, 843, 788, 732, 700, 669, 650, 634, 583, 541, 444, 412.
HRMS: Exact mass calculated for $[C_{18}H_{18}F_3O_5+Na]^+$ requires *m*/*z* = 393.0926, found *m*/*z* = 393.0920 (ESI+).

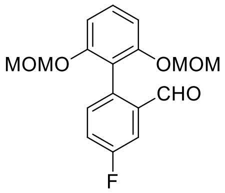

4-Fluoro-2',6'-bis(methoxymethoxy)-[1,1'-biphenyl]-2-carbaldehyde (S19) was synthesized by following Procedure 7. The crude material was purified by normal-phase column chromatography using an eluent of 33% EtOAc/Hx to provide **S19** (631 mg, 100%).

^{1}H NMR (400 MHz, $CDCl_3$) δ 9.73 (d, *J* = 3.2 Hz, 1H), 7.69 (d, *J* = 8.9 Hz, 1H), 7.33 (dd, *J* = 11.0, 4.5 Hz, 3H), 6.91 (d, *J* = 8.4 Hz, 2H), 5.05 (s, 4H), 3.28 (s, 6H).
^{13}C NMR (100 MHz, $CDCl_3$) δ 191.7, 162.2 (d, *J* = 247.6 Hz), 155.6 (2C), 136.2 (d, *J* = 7.3 Hz), 134.2 (d, *J* = 7.8 Hz), 130.4 (2C), 120.7 (d, *J* = 21.9 Hz), 116.3, 112.9 (d, *J* = 22.9 Hz), 108.6 (2C), 94.8 (2C), 56.3 (2C).
IR (FT-ATR, cm^{-1}, CH_2Cl_2) ν_{max} 2902, 2849, 1691, 1596, 1500, 1463, 1441, 1398, 1294, 1258, 1246, 1204, 1150, 1097, 1081, 1030, 920, 894, 831, 787, 733, 663, 573, 534, 452, 407
HRMS: Exact mass calculated for $[C_{17}H_{18}FO_5+Na]^+$ requires *m*/*z* = 343.0958, found *m*/*z* = 343.0952 (ESI+).

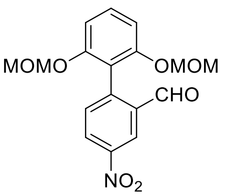

2',6'-Bis(methoxymethoxy)-4-nitro-[1,1'-biphenyl]-2-carbaldehyde (S20) was synthesized by following Procedure 7. The crude material was purified by normal-phase column chromatography using an eluent of 33% EtOAc/Hx to provide **S20** (683 mg, 95%).

^{1}H NMR (400 MHz, $CDCl_3$) δ 9.81 (s, 1H), 8.84 (d, *J* = 2.1 Hz, 1H), 8.45 (dd, *J* = 8.5, 2.4 Hz, 2H), 7.57 (d, *J* = 8.5 Hz, 2H), 7.38 (t, *J* = 8.4 Hz, 1H), 6.95 (d, *J* = 8.5 Hz, 2H), 5.06 (s, 4H), 3.29 (s, 6H).
^{13}C NMR (100 MHz, $CDCl_3$) δ 190.5, 155.2 (2C), 147.5, 144.4, 135.4, 133.9, 131.4, 127.0, 121.9, 114.8, 108.3 (2C), 94.7 (2C), 56.4 (2C).
IR (FT-ATR, cm^{-1}, CH_2Cl_2) ν_{max} 2956, 2851, 1697, 1599, 1524, 1465, 1440 1400, 1345, 1308, 1248, 1204, 1184, 1154, 1098, 1080, 1038, 939, 919, 894, 847, 819, 787, 752, 735, 706, 665, 646, 568, 522, 448.
HRMS: Exact mass calculated for $[C_{17}H_{17}NO_7+Na]^+$ requires *m/z* = 370.0903, found *m/z* = 370.0898 (ESI+).

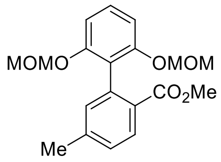

Methyl 2',6'-bis(methoxymethoxy)-5-methyl-[1,1'-biphenyl]-2-carboxylate (S21) was synthesized by following Procedure 7. The crude material was purified by normal-phase column chromatography using an eluent of 33% EtOAc/Hx to provide **S21** (527 mg, 72%).

^{1}H NMR (400 MHz, $CDCl_3$) δ 7.90 (d, *J* = 7.9 Hz, 1H), 7.22 (t, *J* = 9.4 Hz, 2H), 7.11 (s, 1H), 6.87 (d, *J* = 8.3 Hz, 2H), 5.02 (dd, *J* = 6.6, 0.9 Hz, 2H), 4.97 (dd, *J* = 6.6, 0.9 Hz, 2H), 3.62–3.60 (m, 3H), 3.29–3.27 (m, 6H), 2.40 (s, 3H).
^{13}C NMR (100 MHz, $CDCl_3$) δ 167.8, 154.8 (2C), 141.7, 135.7, 132.9, 130.0, 128.6, 128.4, 127.9, 122.3, 108.9 (2C), 94.8 (2C), 55.8 (2C), 51.5, 21.5.
IR (FT-ATR, cm^{-1}, CH_2Cl_2) ν_{max} 2951, 2827, 1729, 1599, 1463, 1436, 1401, 1285, 1252, 1191, 1152, 1099, 1040, 922, 837, 783, 735, 713, 667.
HRMS: Exact mass calculated for $[C_{19}H_{23}O_6+Na]^+$ requires *m/z* = 369.1314, found *m/z* = 369.1309 (ESI+).

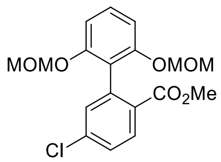

Methyl 5-chloro-2',6'-bis(methoxymethoxy)-[1,1'-biphenyl]-2-carboxylate (S22) was synthesized by following Procedure 7. The crude material was purified by normal-phase column chromatography using an eluent of 33% EtOAc/Hx to provide **S22** (402 mg, 55%).

^{1}H NMR (400 MHz, $CDCl_3$) δ 7.93 (d, J = 8.4 Hz, 1H), 7.37 (d, J = 8.4 Hz, 1H), 7.32 (s, 1H), 7.25 (t, J = 8.4 Hz, 1H), 6.86 (d, J = 8.4 Hz, 2H), 5.05 (d, J = 6.7 Hz, 2H), 4.99 (d, J = 6.7 Hz, 2H), 3.62 (s, 3H), 3.30 (s, 6H).
^{13}C NMR (100 MHz, $CDCl_3$) δ 167.1, 154.7(2C), 137.5, 137.4, 132.4, 131.4, 129.9, 129.4, 127.3, 120.5, 108.6 (2C), 94.8 (2C), 56.1 (2C), 51.9.
IR (FT-ATR, cm^{-1}, CH_2Cl_2) ν_{max} 2952, 2827, 1731, 1590, 1562, 1464, 1435, 1401, 1286, 1244, 1203, 1153, 1100, 1039, 922, 902, 838, 781, 739, 708, 669, 575, 504.
HRMS: Exact mass calculated for $[C_{18}H_{19}ClO_6+H]^+$ requires m/z = 367.0948, found m/z = 367.0944 (ESI+).

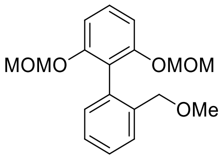

2,6-Bis(methoxymethoxy)-2'-(methoxymethyl)-1,1'-biphenyl (S23) was synthesized by following Procedure 7. The crude material was purified by normal-phase column chromatography using an eluent of 20% EtOAc/Hx to provide **S23** (131 mg, 100%).

^{1}H NMR (400 MHz, $CDCl_3$) δ 7.57 (d, J = 7.5 Hz, 1H), 7.39 (t, J = 7.5 Hz, 1H), 7.35–7.29 (m, 1H), 7.29–7.22 (m, 1H), 7.17 (d, J = 7.5 Hz, 1H), 6.91 (d, J = 8.4 Hz, 2H), 5.02 (q, J = 6.8 Hz, 4H), 4.29 (s, 2H), 3.29 (s, 6H), 3.27 (s, 3H).
^{13}C NMR (100 MHz, $CDCl_3$) δ 155.5 (2C), 137.6, 133.2, 130.8, 129.2, 127.5, 127.0, 126.8, 120.6, 109.1, 94.8 (2C), 72.3 (2C), 58.2, 56.1 (2C).
IR (FT-ATR, cm^{-1}, CH_2Cl_2) ν_{max} 2925, 2824, 1587, 1462, 1401, 1379, 1310, 1244, 1201, 1153, 1097, 1038, 922, 900, 788, 762, 737.
HRMS: Exact mass calculated for $[C_{18}H_{22}O_5+H]^+$ requires m/z = 319.1545, found m/z =319.1543 (ESI+).

2.2.2 Synthesis of Substrate 1c, 1e–h

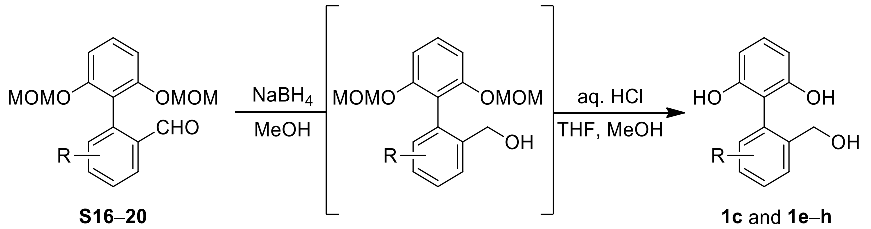

S16–20 **1c** and **1e–h**

Procedure 8: To a reaction tube equipped with a magnetic rring bar, **S16–20** (1.0 equiv) was added and subsequently dissolved in MeOH (0.08 M). $NaBH_4$ (1.2 equiv) was added at 0 °C. The reaction was allowed to stir for 1.5 h. The reaction was diluted with EtOAc and quenched with a saturated aqueous HCl. The organic and aqueous layers were separated and the aqueous layer was extracted an additional two times with EtOAc. The combined organic layers were then rinsed with water, dried with anhydrous $MgSO_4$, filtered and concentrated *in vacuo*. This material was used to next reaction without further purification.

To a round-bottom flask equipped with a magnetic stirring bar and a refluxing condenser, the crude material (1.0 equiv) was added and subsequently dissolved in THF (0.27 M) and MeOH (0.2 M). To a solution aqueous HCl (2.0 M, 4.8 equiv) was added at 0 °C. The reaction was allowed to stir for 3 h. The reaction was diluted with EtOAc and quenched with a saturated aqueous HCl. The organic and aqueous layers were separated and the aqueous layer was extracted with EtOAc, dried with anhydrous $MgSO_4$, filtered and concentrated *in vacuo*. The crude material was then purified by flash chromatography to afford the desired materials.

2.2.3 Synthesis of Substrates 1i and 1j

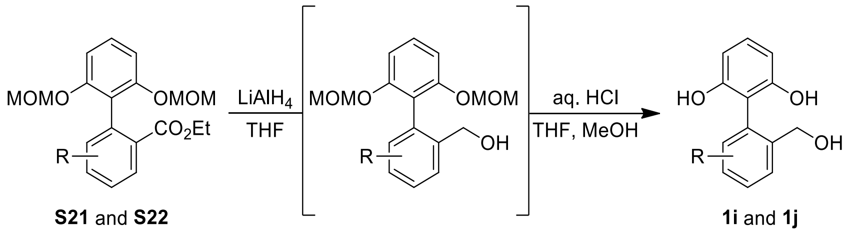

S21 and **S22** **1i** and **1j**

Procedure 9: To a reaction tube equipped with a magnetic stirring bar, **S21** or **S22** (1.0 equiv) was added and subsequently dissolved in THF (0.2 M). To a solution $LiAlH_4$ (2.0 equiv) was added at 0 °C. The reaction was allowed to stir for 6 h. The reaction was diluted with EtOAc and quenched with a saturated aqueous HCl. The organic and aqueous layers were separated and the aqueous layer was extracted an additional two times with EtOAc. The combined organic layers were then rinsed with water, dried with anhydrous $MgSO_4$, filtered and concentrated *in vacuo*. This material was used to next reaction without further purification.

To a round-bottom flask equipped with a magnetic stirring bar and a refluxing condenser, the crude material (1.0 equiv) was added and subsequently dissolved in THF (0.27 M) and MeOH (0.2 M). To a solution aqueous HCl (2.0 M, 4.8 equiv) was added at 0 °C. The reaction was allowed to stir for 3 h. The reaction was diluted with EtOAc and quenched with a saturated aqueous HCl. The organic and aqueous layers were

separated and the aqueous layer was extracted with EtOAc, dried with anhydrous $MgSO_4$, filtered and concentrated *in vacuo*. The crude material was then purified by flash chromatography to afford the desired materials.

2.2.4 Synthesis of Substrate 1l

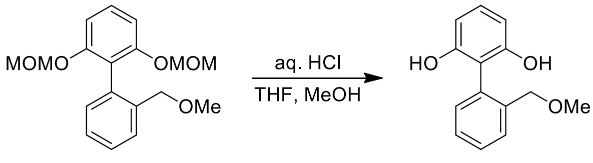

Procedure 10: To a round-bottom flask equipped with a magnetic stirring bar and a refluxing condenser, **S23** (1.0 equiv) was added and subsequently dissolved in THF (0.27 M) and MeOH (0.2 M). To a solution aqueous HCl (2.0 M, 4.8 equiv) was added at 0 °C. The reaction was allowed to stir for 3 h. The reaction was diluted with EtOAc and quenched with a saturated aqueous HCl. The organic and aqueous layers were separated and the aqueous layer was extracted with EtOAc, dried with anhydrous $MgSO_4$, filtered and concentrated *in vacuo*. The crude material was then purified by flash chromatography to afford the desired material.

2.3 Characterization of 1

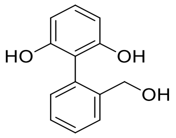

2'-(Hydroxymethyl)-[1,1'-biphenyl]-2,6-diol (1a) was synthesized by following Procedure 3 from **S11**. The crude material was purified by normal-phase column chromatography using an eluent of 33% Hx/EtOAc to provide **1a** (1.24 g, 81%).

^{1}H NMR (400 MHz, CD_3OD) δ 7.57 (d, *J* = 6.9 Hz, 1H), 7.32 (dt, *J* = 22.1, 6.9 Hz, 2H), 7.14 (d, *J* = 6.3 Hz, 1H), 7.00 (t, *J* = 8.1 Hz, 1H), 6.43 (t, *J* = 6.1 Hz, 2H), 4.44 (s, 2H).
^{13}C NMR (100 MHz, CD_3OD) δ 156.5 (2C), 141.7, 134.1, 132.3, 129.8, 128.4, 127.9 (2C), 116.5, 108.1 (2C), 63.4.
IR (FT-ATR, cm^{-1}, CH_2Cl_2) ν_{max} 3502, 3444, 3408, 3326, 3305, 3246, 3214, 3164, 2915, 2855, 1619, 1603, 1587, 1463, 1438, 1309, 1294, 1272, 1197, 1184, 1149, 1033, 1007, 790, 763, 745, 724, 541.
HRMS: Exact mass calculated for $[C_{13}H_{12}O_3-H]^-$ requires *m*/*z* = 215.0708, found *m*/*z* = 215.0714 (ESI−).

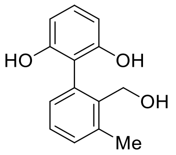

2'-(Hydroxymethyl)-3'-methyl-[1,1'-biphenyl]-2,6-diol (1b) was synthesized by following Procedure 3 from **S12**. The crude material was purified by normal-phase column chromatography using an eluent of 33% Hx/EtOAc to provide **1b** (242 mg, 90%).

^{1}H NMR (400 MHz, CD_3OD) δ 7.26–7.17 (m, 2H), 7.02 (t, *J* = 8.6 Hz, 2H), 6.45 (d, *J* = 8.1 Hz, 2H), 4.46 (s, 2H), 2.50 (s, 3H).
^{13}C NMR (100 MHz, CD_3OD) δ 156.5 (2C), 139.1, 138.8, 136.0, 130.7, 130.3, 129.7, 128.8, 117.7, 108.3 (2C), 61.4, 19.6.
IR (FT-ATR, cm^{-1}, CH_2Cl_2) ν_{max} 3318, 2957, 2924, 2853, 1619, 1589, 1462, 1378, 1295, 1277, 1184, 1277, 1184, 1153, 1006, 966, 791, 763, 744, 732, 651, 564.
HRMS: Exact mass calculated for $[C_{14}H_{14}O_3-H]^-$ requires *m*/*z* = 229.0865, found *m*/*z* = 229.0870 (ESI−).

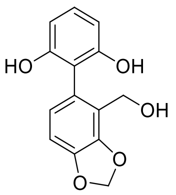

2-(4-(Hydroxymethyl)benzo[*d*][1,3]dioxol-5-yl)benzene-1,3-diol (1c) was synthesized by following Procedure 8 from **S16**. The crude material was purified by normal-phase column chromatography using an eluent of 33% Hx/EtOAc to provide **1c** (189 mg, 50%).

^{1}H NMR (400 MHz, CD_3OD) δ 7.01 (t, *J* = 8.1 Hz, 1H), 6.83 (d, *J* = 8.0 Hz, 1H), 6.66 (d, *J* = 8.0 Hz, 1H), 6.43 (d, *J* = 8.1 Hz, 2H), 6.01 (s, 2H), 4.39 (s, 2H).
^{13}C NMR (100 MHz, CD_3OD) δ 156.9 (2C), 148.2, 148.1, 129.9, 128.9, 125.6, 123.1, 116.1, 108.8, 108.2 (2C), 102.4, 58.5.
IR (FT-ATR, cm^{-1}, CH_2Cl_2) ν_{max} 3280, 2894, 1618, 1585, 1499, 1460, 1449, 1337, 1298, 1246, 1181, 1153, 1098, 1071, 1042, 1005, 954, 931, 862, 814, 789, 734, 702, 677, 644, 607, 582, 544, 498, 477.
HRMS: Exact mass calculated for $[C_{14}H_{12}O_5-H]^-$ requires *m*/*z* = 259.0606, found *m*/*z* = 259.0612 (ESI–).

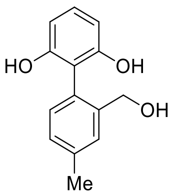

2'-(Hydroxymethyl)-4'-methyl-[1,1'-biphenyl]-2,6-diol (1d) was synthesized by following Procedure 4 from **S3**. The crude material was purified by normal-phase column chromatography using an eluent of 33% Hx/EtOAc to provide **1d** (179 mg, 94%).

^{1}H NMR (400 MHz, CD_3OD) δ 7.39 (s, 1H), 7.12 (d, *J* = 7.7 Hz, 1H), 7.04–6.96 (m, 2H), 6.42 (d, *J* = 8.1 Hz, 2H), 4.40 (s, 2H), 2.39 (s, 3H).
^{13}C NMR (100 MHz, CD_3OD) δ 156.6 (2C), 141.5, 138.1, 132.2, 131.0, 129.6, 128.8, 128.7, 116.4, 108.1 (2C), 63.5, 21.4.
IR (FT-ATR, cm^{-1}, CH_2Cl_2) ν_{max} 3295, 2923, 1612, 1583, 1491, 1459, 1378, 1295, 1268, 1230, 1176, 1152, 1075, 1003, 924, 898, 824, 788, 733, 702, 640, 583, 533, 515, 471, 438.
HRMS: Exact mass calculated for $[C_{14}H_{14}O_3-H]^-$ requires *m*/*z* = 229.0865, found *m*/*z* = 229.0870 (ESI–).

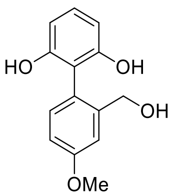

2'-(Hydroxymethyl)-4'-methoxy-[1,1'-biphenyl]-2,6-diol (1e) was synthesized by following Procedure 8 from **S17**. The crude material was purified by normal-phase column chromatography using an eluent of 33% Hx/EtOAc to provide **1e** (283 mg, 72%).

^{1}H NMR (400 MHz, CD_3OD) δ 7.17 (d, *J* = 2.0 Hz, 1H), 7.06 (d, *J* = 8.4 Hz, 1H), 6.99 (t, *J* = 8.1 Hz, 1H), 6.86 (dd, *J* = 8.3, 2.3 Hz, 1H), 6.41 (d, *J* = 8.1 Hz, 2H), 4.41 (s, 2H), 3.83 (s, 3H), 3.35 (s, 2H).
^{13}C NMR (100 MHz, CD_3OD) δ 160.7, 156.8 (2C), 143.3, 133.3, 129.6, 125.7, 116.0, 113.4, 113.1, 108.0 (2C), 63.3, 55.6.
IR (FT-ATR, cm^{-1}, CH_2Cl_2) ν_{max} 3339, 3259, 2925, 2856, 1607, 1493, 1461, 1376, 1296, 1275, 1234, 1159, 1121, 1108, 1077, 1004, 932, 853, 818, 789, 763, 738, 643, 622, 604, 571, 544, 518, 476, 469, 448.
HRMS: Exact mass calculated for $[C_{14}H_{14}O_4{-}H]^-$ requires *m*/*z* = 245.0814, found *m*/*z* = 245.0819 (ESI−).

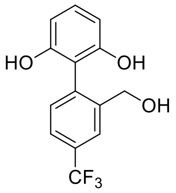

2'-(Hydroxymethyl)-4'-(trifluoromethyl)-[1,1'-biphenyl]-2,6-diol (1f) was synthesized by following Procedure 8 from **S18**. The crude material was purified by normal-phase column chromatography using an eluent of 33% Hx/EtOAc to provide **1f** (108 mg, 56%).

^{1}H NMR (400 MHz, $CDCl_3$) δ 7.81 (s, 1H), 7.66 (d, *J* = 7.9 Hz, 1H), 7.37 (d, *J* = 7.9 Hz, 1H), 7.10 (t, *J* = 8.2 Hz, 1H), 6.48 (d, *J* = 8.2 Hz, 2H), 5.94 (s, 2H), 4.42 (s, 2H).
^{13}C NMR (100 MHz, $CDCl_3$) δ 153.7 (2C), 141.0, 135.4, 132.2, 131.4 (q, *J* = 32.6 Hz), 130.3, 126.8 (q, *J* = 3.4 Hz), 125.7 (q, *J* = 3.3 Hz), 124.0 (q, *J* = 271.4 Hz), 113.7, 108.6 (2C), 63.4.
IR (FT-ATR, cm^{-1}, CH_2Cl_2) ν_{max} 3320, 1706, 1623, 1587, 1461, 1416, 1377, 1329, 1266, 1164, 1121, 1085, 1077, 1005, 907, 888, 839, 789, 732, 689, 665, 644, 610, 584, 429.
HRMS: Exact mass calculated for $[C_{14}H_{11}F_3O_3{-}H]^-$ requires *m*/*z* = 283.0582 found *m*/*z* = 283.0588 (ESI−).

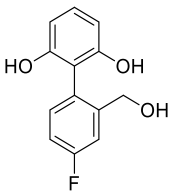

4'-Fluoro-2'-(hydroxymethyl)-[1,1'-biphenyl]-2,6-diol (1g) was synthesized by following Procedure 8 from **S19**. The crude material was purified by normal-phase column chromatography using an eluent of 33% Hx/EtOAc to provide **1g** (359 mg, 94%).

^{1}H NMR (400 MHz, CD_3OD) δ 7.30 (d, *J* = 10.3 Hz, 1H), 7.15–7.09 (m, 1H), 6.99 (dt, *J* = 7.5, 5.2 Hz, 2H), 6.41 (d, *J* = 8.1 Hz, 2H), 4.43 (s, 2H).
^{13}C NMR (100 MHz, CD_3OD) δ 163.9 (d, *J* = 242.9 Hz), 156.7 (2C), 144.9 (d, *J* = 7.0 Hz), 133.9 (d, *J* = 8.0 Hz), 129.62 (d, *J* = 3.3 Hz), 129.59, 115.2, 114.0 (d, *J* = 21.4 Hz), 113.6 (d, *J* = 22.7 Hz), 107.9 (2C), 62.7.
IR (FT-ATR, cm^{-1}, CH_2Cl_2) ν_{max} 3514, 3361, 2923, 1607, 1587, 1488, 1461, 1368, 1312, 1270, 1226, 1179, 1149, 1101, 1078, 1034, 1007, 951, 874, 824, 789, 737, 586, 531, 446.
HRMS: Exact mass calculated for $[C_{13}H_{11}FO_3{-}H]^-$ requires *m*/*z* = 233.0614, found *m*/*z* = 233.0619 (ESI−).

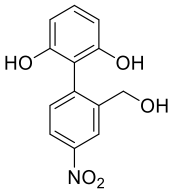

2'-(Hydroxymethyl)-4'-nitro-[1,1'-biphenyl]-2,6-diol (1h) was synthesized by following Procedure 8 from **S20**. The crude material was purified by normal-phase column chromatography using an eluent of 33% Hx/EtOAc to provide **1h** (225 mg, 100%).

^{1}H NMR (400 MHz, CD_3OD) δ 8.48 (s, 1H), 8.12 (d, *J* = 8.4 Hz, 1H), 7.37 (d, *J* = 8.4 Hz, 1H), 7.05 (t, *J* = 8.2 Hz, 1H), 6.43 (d, *J* = 8.2 Hz, 2H), 4.53 (s, 2H).
^{13}C NMR (100 MHz, CD_3OD) δ 156.5 (2C), 148.7, 144.7, 141.7, 133.5, 130.8, 122.0, 121.4, 114.3, 107.8 (2C), 62.1.
IR (FT-ATR, cm^{-1}, CH_2Cl_2) ν_{max} 3371, 2927, 2854, 1619, 1519, 1461, 1347, 1264, 1008, 897, 789, 735, 704.
HRMS: Exact mass calculated for $[C_{13}H_{11}NO_5{-}H]^-$ requires *m*/*z* = 260.0559, found *m*/*z* = 260.0564 (ESI−).

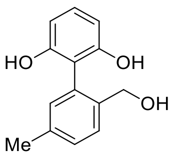

2'-(Hydroxymethyl)-4-methyl-[1,1'-biphenyl]-2,6-diol (1i) was synthesized by following Procedure 9 from **S21**. The crude material was purified by normal-phase column chromatography using an eluent of 33% Hx/EtOAc to provide **1i** (148 mg, 49%).

^{1}H NMR (400 MHz, $CDCl_3$) δ 7.44 (d, J = 7.8 Hz, 1H), 7.25 (d, 1H), 7.14–7.08 (m, 2H), 6.54 (d, J = 8.2 Hz, 2H), 5.76 (s, 2H), 4.34 (s, 2H), 2.37 (s, 3H).
^{13}C NMR (100 MHz, $CDCl_3$) δ 153.8 (2C), 139.6, 137.5, 132.1, 130.8, 130.6, 130.5, 129.8, 114.8, 108.4 (2C), 63.7, 21.3.
IR (FT-ATR, cm^{-1}, CH_2Cl_2) ν_{max} 3320, 1706, 1623, 1587, 1461, 1416, 1377, 1329, 1266, 1164, 1121, 1085, 1077, 1005, 907, 888, 839, 789, 732, 689, 665, 644, 610, 584, 429.
HRMS: Exact mass calculated for $[C_{14}H_{14}O_3{-}H]^-$ requires m/z = 229.0865, found m/z = 229.0870 (ESI−).

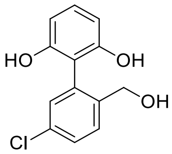

5'-Chloro-2'-(hydroxymethyl)-[1,1'-biphenyl]-2,6-diol (1j) was synthesized by following Procedure 9 from **S22**. The crude material was purified by normal-phase column chromatography using an eluent of 33% Hx/EtOAc to provide **1j** (121 mg, 51%).

^{1}H NMR (400 MHz, CD_3OD) δ 7.55 (d, J = 8.3 Hz, 1H), 7.33 (dd, J = 8.3, 1.9 Hz, 1H), 7.12 (d, J = 1.8 Hz, 1H), 7.02 (t, J = 8.1 Hz, 1H), 6.42 (d, J = 8.1 Hz, 2H), 4.41 (s, 2H).
^{13}C NMR (100 MHz, CD_3OD) δ 156.6 (2C), 140.8, 136.2, 133.0, 132.0, 130.2, 128.9, 128.1, 115.1, 107.9 (2C), 62.6.
IR (FT-ATR, cm^{-1}, CH_2Cl_2) ν_{max} 3287, 1619, 1592, 1566, 1459, 1394, 1298, 1265, 1176, 1153, 1194, 1003, 888, 851, 824, 789, 732, 701, 657, 563, 530, 491.
HRMS: Exact mass calculated for $[C_{13}H_{12}ClO_3{-}H]^-$ requires m/z = 249.0318, found m/z = 249.0324 (ESI−).

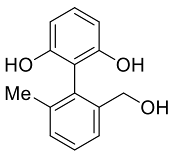

2'-(Hydroxymethyl)-6'-methyl-[1,1'-biphenyl]-2,6-diol (1k) was synthesized by following Procedure 3 from **S13**. The crude material was purified by normal-phase column chromatography using an eluent of 33% Hx/EtOAc to provide **1k** (463 mg, 70%).

^{1}H NMR (400 MHz, CD_3OD) δ 7.39 (d, *J* = 7.4 Hz, 1H), 7.26 (t, *J* = 7.5 Hz, 1H), 7.19 (d, *J* = 7.3 Hz, 1H), 7.03 (t, *J* = 8.1 Hz, 1H), 6.45 (d, *J* = 8.1 Hz, 2H), 4.35 (s, 2H), 2.04 (s, 3H).
^{13}C NMR (100 MHz, CD_3OD) δ 156.2 (2C), 141.6, 138.9, 133.5, 129.8, 129.5, 128.4, 125.1, 115.0, 108.1 (2C), 63.7, 20.1.
IR (FT-ATR, cm^{-1}, CH_2Cl_2) ν_{max} 3334, 2923, 1620, 1581, 1504, 1458, 1379, 1301, 1266, 1174, 1150, 1059, 1036, 1003, 905, 785, 760, 731, 702, 645, 579, 526, 492.
HRMS: Exact mass calculated for $[C_{14}H_{14}O_3{-}H]^-$ requires *m*/*z* = 229.0862, found *m*/*z* = 229.0868 (ESI−).

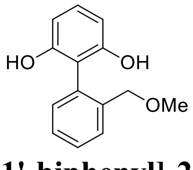

2'-(Hydroxymethyl)-3'-methyl-[1,1'-biphenyl]-2,6-diol (1l) was synthesized by following Procedure 10 from **S23**. The crude material was purified by normal-phase column chromatography using an eluent of 33% Hx/EtOAc to provide **1l** (93 mg, 70%). The spectral data were identical with those previously reported.[6]

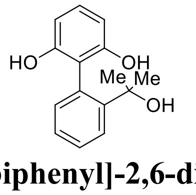

2'-(2-Hydroxypropan-2-yl)-[1,1'-biphenyl]-2,6-diol (1m) was synthesized by following Procedure 5 from **S15**. The crude material was purified by normal-phase column chromatography using an eluent of 33% Hx/EtOAc to provide **1m** (135 mg, 99%).

^{1}H NMR (400 MHz, CD_3OD) δ 7.79 (d, *J* = 8.0 Hz, 1H), 7.32 (t, *J* = 7.6 Hz, 1H), 7.25 (t, *J* = 7.4 Hz, 1H), 7.04–6.94 (m, 2H), 6.40 (d, *J* = 8.1 Hz, 2H), 1.41 (s, 6H).
^{13}C NMR (100 MHz, CD_3OD) δ 156.5 (2C), 149.0, 134.1, 133.4, 129.6, 128.4, 127.8, 127.1, 120.5, 108.0 (2C), 74.1, 30.7 (2C).
IR (FT-ATR, cm^{-1}, CH_2Cl_2) ν_{max} 3500, 3329, 3064, 2966, 2925, 2854, 1619, 1582, 1505, 1460, 1438, 1366, 1312, 1275, 1176, 1152, 1117, 1072, 1049, 1006, 944, 857, 790, 761, 735, 702, 666, 585, 566, 526, 483, 449, 412.
HRMS: Exact mass calculated for $[C_{15}H_{16}O_3{-}H]^-$ requires *m*/*z* = 243.1019, found *m*/*z* = 243.1029 (ESI−).

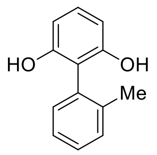

2'-Methyl-[1,1'-biphenyl]-2,6-diol (1n) was synthesized by following Procedure 6 from **S5**. The crude material was purified by normal-phase column chromatography using an eluent of 25% EtOAc/Hx to provide **1n** (421 mg, 89%). The spectral data were identical with those previously reported.[3]

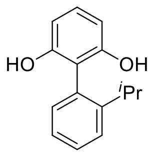

2'-Isopropyl-[1,1'-biphenyl]-2,6-diol (1o) was synthesized by following Procedure 6 from **S6**. The crude material was purified by normal-phase column chromatography using an eluent of 14% EtOAc/Hx to provide **1o** (86 mg, 66%). The spectral data were identical with those previously reported.[3]

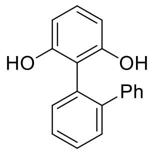

[1,1':2',1''-Terphenyl]-2,6-diol (1p) was synthesized by following Procedure 6 from **S7**. The crude material was purified by normal-phase column chromatography using an eluent of 14% EtOAc/Hx to provide **1p** (94 mg, 74%). The spectral data were identical with those previously reported.[3]

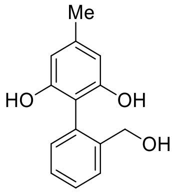

2'-(Hydroxymethyl)-4-methyl-[1,1'-biphenyl]-2,6-diol (1q) was synthesized by following Procedure 4 from **S8**. The crude material was purified by normal-phase column chromatography using an eluent of 33% Hx/EtOAc to provide **1q** (374 mg, 82%).

^{1}H NMR (400 MHz, CD_3OD) δ 7.56 (d, *J* = 7.6 Hz, 1H), 7.31 (dt, *J* = 22.1, 7.4 Hz, 2H), 7.14 (d, *J* = 7.4 Hz, 1H), 6.28 (s, 2H), 4.44 (s, 2H), 2.24 (s, 3H).
^{13}C NMR (100 MHz, CD_3OD) δ 156.2 (2C), 141.8, 140.0, 134.2, 132.5, 128.3, 127.92, 127.87, 113.7, 108.9 (2C), 63.5, 21.5.
IR (FT-ATR, cm^{-1}, CH_2Cl_2) ν_{max} 3354, 1630, 1582, 1417, 1332, 1174, 1041, 987, 824, 761, 589.
HRMS: Exact mass calculated for $[C_{14}H_{14}O_3{-}H]^-$ requires *m*/*z* = 229.0862, found *m*/*z* = 229.0868 (ESI−).

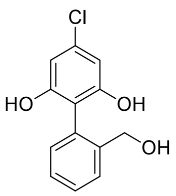

4-Chloro-2'-(hydroxymethyl)-[1,1'-biphenyl]-2,6-diol (1r) was synthesized by following Procedure 4 from **S9**. The crude material was purified by normal-phase column chromatography using an eluent of 33% Hx/EtOAc to provide **1r** (397 mg, 82%).

^{1}H NMR (400 MHz, CD_3OD) δ 7.57 (d, *J* = 7.6 Hz, 1H), 7.35 (t, *J* = 7.5 Hz, 1H), 7.28 (t, *J* = 7.4 Hz, 1H), 7.12 (d, *J* = 7.5 Hz, 1H), 6.44 (s, 2H), 4.44 (s, 2H).
^{13}C NMR (100 MHz, CD_3OD) δ 157.4 (2C), 141.8, 134.6, 133.2, 132.2, 128.6, 127.8, 127.7, 115.3, 108.2 (2C), 63.2.
IR (FT-ATR, cm^{-1}, CH_2Cl_2) ν_{max} 3315, 2956, 2924, 2723, 1614, 1581, 1505, 1483, 1415, 1377, 1321, 1264, 1226, 1163, 1112, 1094, 1034, 1005, 951, 888, 825, 765, 702, 620, 591, 572, 530, 482, 442, 425, 409.
HRMS: Exact mass calculated for $[C_{13}H_{11}ClO_3{-}H]^-$ requires *m*/*z* = 249.0318, found *m*/*z* = 249.0324 (ESI−).

3 Reaction Optimizations and Procedures

3.1 Ligand Screening

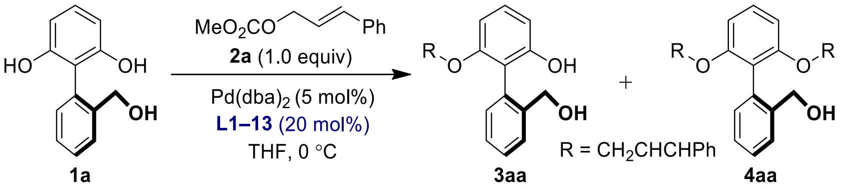

To an oven dried reaction tube equipped with a magnetic stirring bar was premixed $Pd(dba)_2$ (2.9 mg, 0.005 mmol, 0.05 equiv) and **L1**–**13** (0.02 mmol, 0.2 equiv) with THF (0.2 ml) at 0 °C. After 10 min, methyl cinnamyl carbonate, **2a** (19.2 mg, 0.1 mmol, 1 equiv) was added with THF (0.3 ml) and the reaction mixture was left to stir for 10 min at 0 °C. Then, **1a** (21.6 mg, 0.1 mmol, 1 equiv) was added and the reaction mixture was left to stir for 1–48 h at 0 °C. The reaction tube was sealed with a Teflon cap and further secured with Parafilm M®. After that, the crude material was purified by flash column chromatography using an eluent of 33% EtOAc/Hx to provide the desired product. The enantioselectivity was determined by chiral HPLC.

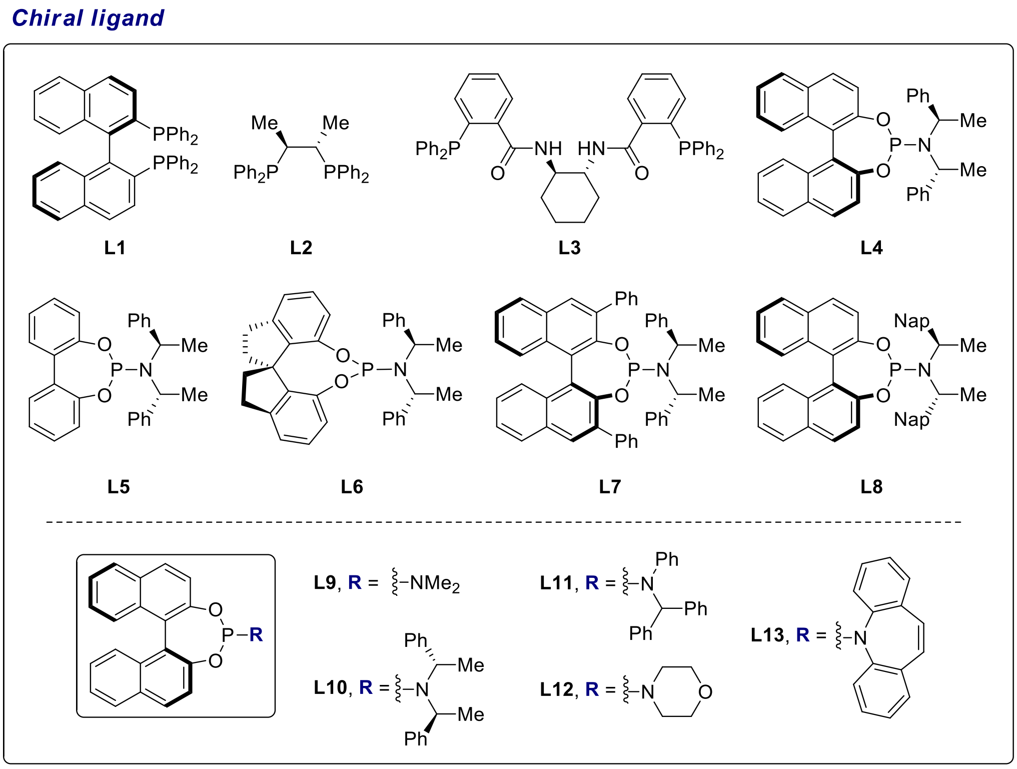

Supplementary Table 1. Ligand screening

Entry	Ligand	Time (h)	**3aa** (%)	**4aa** (%)	er of **3aa**[a]
1	**L1**	48	30	6	49:51
2	**L2**	48	<5	n.d.[b]	n.d.[b]
3	**L3**	48	25	3	35:65
4	**L4**	2	44	18	91:9
5	**L5**	3	18	10	89:11
6	**L6**	48	11	n.d.[b]	67:33
7	**L7**	1.5	42	26	41:59
8	**L8**	48	11	n.d.[b]	57:43
9	**L9**	3	32	11	57:43
10	**L10**	0.5	27	17	58:42
11	**L11**	0.5	49	23	61:39
12	**L12**	11	38	26	53:47
13	**L13**	0.5	59	12	44:56

[a]Enantiomeric ratios were determined by chiral HPLC analysis. [b]Not determined.

3.2 Catalyst Screening

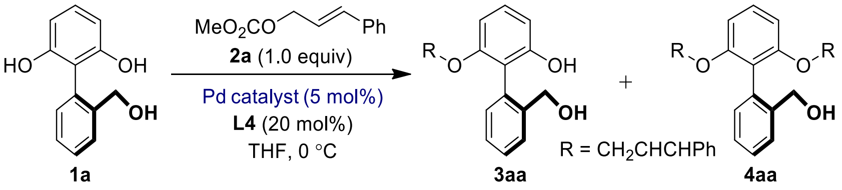

To an oven dried reaction tube equipped with a magnetic stirring bar was premixed Pd catalyst (0.005 mmol, 0.05 equiv) and **L4** (10.8 mg, 0.02 mmol, 0.2 equiv) with THF (0.2 ml) at 0 °C. After 10 min, methyl cinnamyl carbonate, **2a** (19.2 mg, 0.1 mmol, 1 equiv) was added with THF (0.3 ml) and the reaction mixture was left to stir for 10 min at 0 °C. Then, **1a** (21.6 mg, 0.1 mmol, 1 equiv) was added and the reaction mixture was left to stir for 0.5–19 h at 0 °C. The reaction tube was sealed with a Teflon cap and further secured with Parafilm M®. After that, the crude material was purified by flash column chromatography using an eluent of 33% EtOAc/Hx to provide the desired product. The enantioselectivity was determined by chiral HPLC.

Supplementary Table 2. Catalyst screening

Entry	Catalyst	Time (h)	**3aa** (%)	**4aa** (%)	er of **3aa**[a]
1	$Pd(dba)_2$	2	44	18	91:9
2	$Pd_2(dba)_3{\cdot}CHCl_3$	0.5	48	20	88:12
3	$Pd_2(dba)_3$	0.5	55	15	89:11
4	$[PdClAllyl]_2$	19	48	26	87:13
5	$Pd(PPh_3)_4$	19	39	21	58:42

[a]Enantiomeric ratios were determined by chiral HPLC analysis.

3.3 Allyl Electrophiles Screening

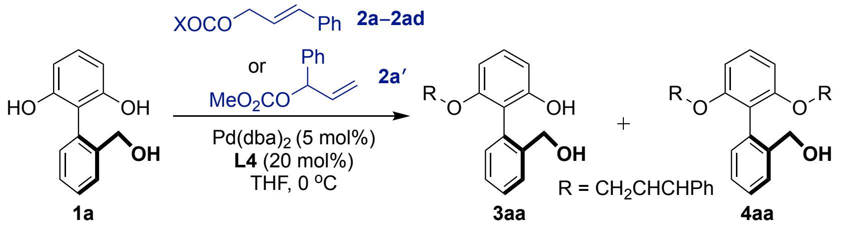

To an oven dried reaction tube equipped with a magnetic stirring bar was premixed $Pd(dba)_2$ (2.9 mg, 0.005 mmol, 0.05 equiv) and **L4** (10.8 mg, 0.02 mmol, 0.2 equiv) with THF (0.2 ml) at 0 °C. After 10 min, **2** (0.1 mmol, 1 equiv) was added with THF (0.3 ml) and the reaction mixture was left to stir for 10 min at 0 °C. Then, **1a** (21.6 mg, 0.1 mmol, 1 equiv) was added and the reaction mixture was left to stir for 2–48 h at 0 °C. The reaction tube was sealed with a Teflon cap and further secured with Parafilm M®. After that, the crude material was purified by flash column chromatography using an eluent of 33% EtOAc/Hx to provide the desired product. The enantioselectivity was determined by chiral HPLC.

Supplementary Table 3. Allyl electrophiles screening

Entry	**2**	Time (h)	**3aa** (%)	**4aa** (%)	er of **3aa**[a]
1	**2a** (X = OMe)	2	44	18	91:9
2	**2ab** (X = O^{i}Pr)	8	46	16	86:14
3	**2ac** (X = OBn)	2	47	26	90:10
4	**2ad** (X = Me)	48	7	21	82:18
5	**2a′**	2	55	22	90:10

[a]Enantiomeric ratios were determined by chiral HPLC analysis.

3.4 Temperature and Additive Screening

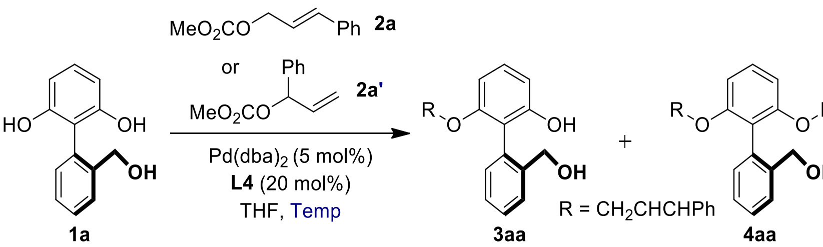

To an oven dried reaction tube equipped with a magnetic stirring bar was premixed $Pd(dba)_2$ (2.9 mg, 0.005 mmol, 0.05 equiv) and **L4** (10.8 mg, 0.02 mmol, 0.2 equiv) with THF (0.2 ml). After 10 min, methyl cinnamyl carbonate, **2a** or **2a′** (19.2 mg, 0.1 mmol, 1 equiv) was added with THF (0.3 ml) and the reaction mixture was left to stir for 10 min. Then, **1a** (21.6 mg, 0.1 mmol, 1 equiv) was added and the reaction mixture was left to stir for 2–10 h. The reaction tube was sealed with a Teflon cap and further secured with Parafilm M®. After that, the crude material was purified by flash column chromatography using an eluent of 33% EtOAc/Hx to provide the desired product. The enantioselectivity was determined by chiral HPLC.

Supplementary Table 4. Temperature and additive screening

Entry	Allyl	Additive	Temp (°C)	Time (h)	**3aa** (%)	**4aa** (%)	er of **3aa**[a]
1	**2a**		0	2	44	18	91:9
2	**2a′**		0	2	55	22	90:10
3	**2a**		–20	3	53	23	93:7
4	**2a′**		–20	3	55	22	94:6
5	**2a′**	4Å M.S.	–20	3	49	13	90:10
6	**2a**		–45	9	27	29	91:9
7	**2a′**		–45	4	43	28	94:6
8	**2a′**	4Å M.S.	–45	4	27	34	93:7
9	**2a′**		–78	10	18	8	82:18

[a]Enantiomeric ratios were determined by chiral HPLC analysis.

3.5 Equivalent Screening

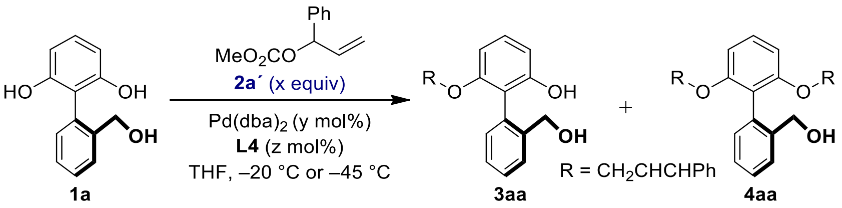

To an oven dried reaction tube equipped with a magnetic stirring bar was premixed $Pd(dba)_2$ (y mol%) and **L4** (z mol%) with THF (0.2 ml) at –20 °C or –45 °C. After 10 min, methyl cinnamyl carbonate, **2a′** (x equiv) was added with THF (0.3 ml) and the reaction mixture was left to stir for 10 min at –20 °C or –45 °C. Then, **1a** (21.6 mg, 0.1 mmol, 1 equiv) was added and the reaction mixture was left to stir for 2–16 h at –20 °C or –45 °C. The reaction tube was sealed with a Teflon cap and further secured with Parafilm M®. After that, the crude material was purified by flash column chromatography using an eluent of 33% EtOAc/Hx to provide the desired product. The enantioselectivity was determined by chiral HPLC.

Supplementary Table 5. Equivalent screening

Entry	x	y	z	Temp (°C)	Time (h)	**3aa** (%)	**4aa** (%)	er of **3aa**[a]
1	1.0	5	20	–20	3	55	22	94:6
2	1.0	5	20	–45	4	43	28	94:6
3	1.2	5	20	–20	2	35	35	94:6
4	1.2	5	20	–45	8	25	41	94:6
5	1.5	5	20	–20	2	47	30	96:4
6	1.5	5	20	–45	16	32	52	95:5
7	1.5	2.5	20	–20	4	41	51	96:4
8	1.5	1	20	–20	10	48	46	96:4
9	1.5	1	10	–20	10	42	51	97:3
10	1.5	1	4	–20	10	49	42	97:3
11	1.5	0.5	2	–20	16	48	34	96:4

[a]Enantiomeric ratios were determined by chiral HPLC analysis.

4 Reaction Procedures for 3 and 4

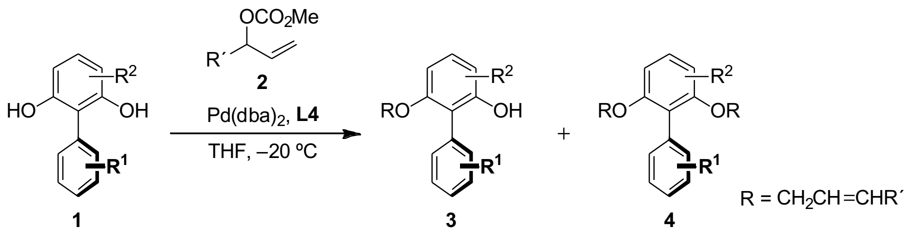

Procedure 11: To an oven dried reaction tube equipped with a magnetic stirring bar was premixed Pd(dba)$_2$ (0.6 mg, 0.001 mmol, 0.01 equiv), **L4** (2.2 mg, 0.004 mmol, 0.04 equiv) with THF (0.2 ml). After 10 min, **2** (0.15 mmol, 1.5 equiv) was added with THF (0.3 ml) and stirring was continued for 10 min. Then, **1** (0.10 mmol, 1 equiv) was added and the reaction mixture was left to stir for 10–240 h at –20 °C. The vial was sealed with a Teflon cap and further secured with Parafilm M®. After that, the crude material was purified by flash column chromatography using an eluent of 9–33% EtOAc/Hx to provide the desired product **3** and **4**. The enantioselectivity was determined by chiral HPLC.

5 Characterization of Products

5.1 Characterization of Products 3

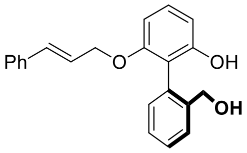

6-(Cinnamyloxy)-2'-(hydroxymethyl)-[1,1'-biphenyl]-2-ol (3aa) was synthesized by following Procedure 11 from **1a**. The crude material was purified by normal-phase column chromatography using an eluent of 9–33% EtOAc/Hx to provide **3aa** (49% yield).

^{1}H NMR (400 MHz, $CDCl_3$) δ 7.64 (dd, *J* = 7.5, 1.4 Hz, 1H), 7.52–7.42 (m, 2H), 7.31–7.27 (m, 5H), 7.25–7.20 (m, 2H), 6.70 (dd, *J* = 8.2, 0.7 Hz, 1H), 6.64 (d, *J* = 8.3 Hz, 1H), 6.42 (d, *J* = 16.0 Hz, 1H), 6.17 (dt, *J* = 16.0, 5.4 Hz, 1H), 4.67–4.57 (m, 2H), 4.44 (q, *J* = 12.0 Hz, 2H).
^{13}C NMR (100 MHz, $CDCl_3$) δ 156.4, 154.1, 140.9, 136.4, 132.6, 131.4, 131.3, 130.0, 129.7, 129.2, 128.8, 128.7 (2C), 128.0, 126.6 (2C), 124.0, 116.6, 109.5, 105.3, 69.4, 63.9.
IR (FT-ATR, cm^{-1}, CH_2Cl_2) ν_{max} 3295, 3059, 3025, 2923, 2853, 1610, 1590, 1494, 1459, 1378, 1304, 1253, 1199, 1158, 1101, 1058, 1006, 968, 785, 761, 732, 692, 608, 578, 540, 492, 437, 425.
HRMS: Exact mass calculated for $[C_{22}H_{20}O_3-H]^-$ requires *m*/*z* = 331.1412, found *m*/*z* = 331.1338 (ESI−).
Optical: $[\alpha]^{20}_{D}$ = +48.9° (*c* = 1.05, $CHCl_3$, 97:3 er)
HPLC (Chiralpak AD-H, 20% ^{i}PrOH/Hx eluent, 1 mL/min, 254 nm): major enantiomer t_R = 13.6 min, minor enantiomer t_R = 27.9 min.

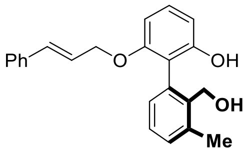

6-(Cinnamyloxy)-2'-(hydroxymethyl)-3'-methyl-[1,1'-biphenyl]-2-ol (3ba) was synthesized by following Procedure 11 from **1b**. The crude material was purified by normal-phase column chromatography using an eluent of 9–33% EtOAc/Hx to provide **3ba** (43% yield).

^{1}H NMR (400 MHz, $CDCl_3$) δ 7.37–7.30 (m, 2H), 7.28 (t, *J* = 6.0 Hz, 5H), 7.23 (d, *J* = 8.2 Hz, 1H), 7.10 (d, *J* = 6.9 Hz, 1H), 6.70 (d, *J* = 8.3 Hz, 1H), 6.65 (d, *J* = 8.3 Hz, 1H), 6.43 (d, *J* = 16.0 Hz, 1H), 6.17 (dt, *J* = 15.9, 5.5 Hz, 1H), 5.03 (s, 1H), 4.62 (qd, *J* = 13.0, 5.5 Hz, 2H), 4.48–4.37 (m, 2H), 2.54 (s, 3H).
^{13}C NMR (100 MHz, $CDCl_3$) δ 156.4, 154.2, 139.5, 138.9, 136.3, 133.1, 132.2, 131.3, 129.5, 128.9, 128.7 (2C), 128.66, 128.1, 126.7 (2C), 123.9, 117.6, 109.6, 105.6, 69.7, 61.1, 19.6.
IR (FT-ATR, cm^{-1}, CH_2Cl_2) ν_{max} 3214, 3058, 3024, 2955, 2923, 2854, 1608, 1591, 1496, 1464, 1451, 1379, 1302, 1261, 1241, 1199, 1100, 1055, 986, 965, 847, 792, 782, 732, 692, 669, 645, 595, 575, 540, 495.

HRMS: Exact mass calculated for $[C_{22}H_{22}O_3-H]^-$ requires m/z = 345.1488, found m/z = 345.1493 (ESI–).
Optical: $[\alpha]^{20}_D$ = +99.0° (c = 0.95, $CHCl_3$, 95:5 er)
HPLC (Chiralpak AD, 40% ^{i}PrOH/Hx eluent, 1 mL/min, 254 nm): major enantiomer t_R = 8.5 min, minor enantiomer t_R = 12.0 min.

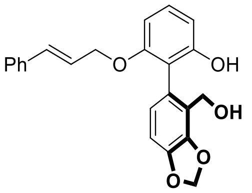

3-(Cinnamyloxy)-2-(4-(hydroxymethyl)benzo[d][1,3]dioxol-5-yl)phenol (3ca) was synthesized by following Procedure 11 from **1c**. The crude material was purified by normal-phase column chromatography using an eluent of 9–33% EtOAc/Hx to provide **3ca** (49% yield).

^{1}H NMR (400 MHz, $CDCl_3$) δ 7.26–7.23 (m, 3H), 7.23–7.18 (m, 2H), 7.18–7.15 (m, 1H), 6.86 (d, J = 7.9 Hz, 1H), 6.71 (d, J = 7.9 Hz, 1H), 6.65 (d, J = 8.2 Hz, 1H), 6.58 (d, J = 8.3 Hz, 1H), 6.46 (d, J = 15.9 Hz, 1H), 6.16 (dt, J = 15.9, 5.7 Hz, 1H), 6.06–6.02 (m, 2H), 5.11 (s, 1H), 4.64–4.52 (m, 2H), 4.35 (q, J = 11.9 Hz, 2H).
^{13}C NMR (100 MHz, $CDCl_3$) δ 156.8, 154.6, 147.9, 136.3, 133.3, 129.6, 128.7 (2C), 128.1, 126.7 (2C), 125.3, 124.1, 124.0, 122.6, 115.8, 109.5, 108.8, 105.4, 101.8, 69.8, 58.2.
IR (FT-ATR, cm^{-1}, CH_2Cl_2) ν_{max} 3261, 2923, 1582, 1497, 1458, 1378, 1330, 1299, 1246, 1160, 1098, 1043, 1022, 989, 964, 932, 861, 811, 784, 731, 692, 643, 609, 564, 539, 496, 477.
HRMS: Exact mass calculated for $[C_{23}H_{20}O_5-H]^-$ requires m/z = 375.1230, found m/z = 375.1238 (ESI–).
Optical: $[\alpha]^{20}_D$ = +87.0° (c = 1.13, $CHCl_3$, 94:6 er)
HPLC (Chiralpak AD-H, 20% ^{i}PrOH/Hx eluent, 1 mL/min, 254 nm): major enantiomer t_R = 12.0 min, minor enantiomer t_R = 24.6 min.

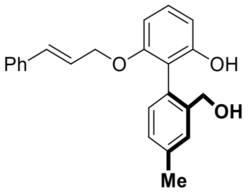

6-(Cinnamyloxy)-2'-(hydroxymethyl)-4'-methyl-[1,1'-biphenyl]-2-ol (3da) was synthesized by following Procedure 11 from **1d**. The crude material was purified by normal-phase column chromatography using an eluent of 9–33% EtOAc/Hx to provide **3da** (45% yield).

^{1}H NMR (400 MHz, $CDCl_3$) δ 7.44 (s, 1H), 7.32–7.20 (m, 7H), 7.15 (d, J = 7.6 Hz, 1H), 6.68 (d, J = 8.3 Hz, 1H), 6.63 (d, J = 8.3 Hz, 1H), 6.44 (d, J = 16.0 Hz, 1H), 6.18 (dt, J = 10.9, 5.2 Hz, 1H), 5.14 (s, 1H), 4.66–4.56 (m, 2H), 4.38 (q, J = 11.9 Hz, 2H), 2.44 (s, 3H).

^{13}**C NMR** (100 MHz, $CDCl_3$) δ 156.6, 154.3, 140.7, 139.1, 136.4, 132.8, 131.1, 130.8, 129.6, 129.5, 128.7 (2C), 128.2, 128.0, 126.7 (2C), 124.1, 116.6, 109.4, 105.3, 69.5, 64.0, 21.4.
IR (FT-ATR, cm^{-1}, CH_2Cl_2) ν_{max} 3263, 3025, 2922, 2853, 1720, 1582, 1458, 1316, 1377, 1304, 1257, 1226, 1185, 1157, 1101, 1057, 1005, 965, 889, 822, 784, 770, 732, 692, 633, 609, 588, 572, 525, 502, 473, 439.
HRMS: Exact mass calculated for $[C_{23}H_{22}O_3–H]^-$ requires *m*/*z* = 345.1488, found *m*/*z* = 345.1496 (ESI–).
Optical: $[\alpha]^{20}_{D}$ = +46.7° (*c* = 1.02, $CHCl_3$, 95:5 er)
HPLC (Chiralpak AD-H, 20% ^{i}PrOH/Hx eluent, 1 mL/min, 254 nm): major enantiomer t_R = 9.7 min, minor enantiomer t_R = 14.4 min.

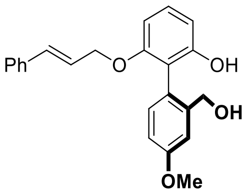

6-(Cinnamyloxy)-2'-(hydroxymethyl)-4'-methoxy-[1,1'-biphenyl]-2-ol (3ea) was synthesized by following Procedure 11 from **1e**. The crude material was purified by normal-phase column chromatography using an eluent of 9–33% EtOAc/Hx to provide **3ea** (50% yield).
^{1}**H NMR** (400 MHz, $CDCl_3$) δ 7.26 (q, *J* = 4.3 Hz, 3H), 7.23 (d, *J* = 4.9 Hz, 1H), 7.22–7.19 (m, 1H), 7.18 (d, *J* = 4.1 Hz, 1H), 7.15 (d, *J* = 8.7 Hz, 1H), 6.95 (dd, *J* = 8.4, 2.7 Hz, 1H), 6.66 (d, *J* = 8.2 Hz, 1H), 6.60 (d, *J* = 8.3 Hz, 1H), 6.43 (d, *J* = 16.0 Hz, 1H), 6.16 (dt, *J* = 16.0, 5.5 Hz, 1H), 5.00 (s, 1H), 4.65–4.54 (m, 2H), 4.37 (q, *J* = 12.4 Hz, 2H), 3.86 (s, 3H).
^{13}**C NMR** (100 MHz, $CDCl_3$) δ 160.2, 156.7, 154.4, 142.5, 136.4, 132.8, 132.4, 129.5, 128.7 (2C), 128.0, 126.6 (2C), 124.1, 122.8, 116.2, 114.8, 114.4, 109.2, 105.2, 69.5, 63.9, 55.5.
IR (FT-ATR, cm^{-1}, CH_2Cl_2) ν_{max} 3510, 3418, 3152, 3023, 2921, 2853, 1603, 1593, 1492, 1457, 1414, 1376, 1357, 1303, 1261, 1231, 1189, 1161, 1108, 1091, 1058, 1041, 1027, 1003, 967, 955, 937, 854, 827, 784, 735, 692, 587, 536.
HRMS: Exact mass calculated for $[C_{23}H_{22}O_4–H]^-$ requires *m*/*z* = 361.1438, found *m*/*z* = 361.1446 (ESI–).
Optical: $[\alpha]^{20}_{D}$ = +38.7° (*c* = 1.06, $CHCl_3$, 92:8 er)
HPLC (Chiralpak AD-H, 20% ^{i}PrOH/Hx eluent, 1 mL/min, 254 nm): major enantiomer t_R = 16.3 min, minor enantiomer t_R = 23.1 min.

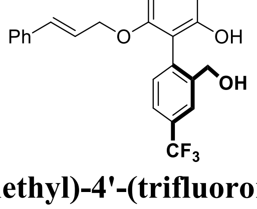

6-(Cinnamyloxy)-2'-(hydroxymethyl)-4'-(trifluoromethyl)-[1,1'-biphenyl]-2-ol (3fa) was synthesized by following Procedure 11 from **1f**. The crude material was purified by normal-phase column chromatography using an eluent of 9–33% EtOAc/Hx to provide **3fa** (29% yield).

^{1}H NMR (400 MHz, $CDCl_3$) δ 7.92 (s, 1H), 7.68 (d, *J* = 7.9 Hz, 1H), 7.39 (d, *J* = 7.9 Hz, 1H), 7.30 (t, *J* = 4.2 Hz, 1H), 7.27 (dd, *J* = 4.5, 2.4 Hz, 4H), 7.26–7.21 (m, 2H), 6.69–6.63 (m, 2H), 6.39 (d, *J* = 16.0 Hz, 1H), 6.16 (dt, *J* = 16.0, 5.4 Hz, 1H), 5.07 (s, 1H), 4.69–4.57 (m, 2H), 4.50 (q, *J* = 12.7 Hz, 2H).
^{13}C NMR (100 MHz, $CDCl_3$) δ 156.4, 153.8, 141.7, 136.2, 132.7, 132.0, 130.9, 130.2, 128.7 (2C), 128.1, 126.6 (2C), 126.2 (q, *J* = 3.0 Hz), 125.1 (q, J = 2.5 Hz), 123.7, 119.5 (q, J = 256.7 Hz), 115.5, 109.7, 105.3, 69.3, 63.3.
IR (FT-ATR, cm^{-1}, CH_2Cl_2) ν_{max} 3246, 3060, 3026, 2924, 2854, 1595, 1579, 1496, 1482, 1448, 1415, 1378, 1319, 1265, 1203, 1169, 1112, 1077, 1031, 1004, 964, 889, 872, 830, 769, 748, 737, 693, 656, 634, 609, 591, 561, 544, 506, 437.
HRMS: Exact mass calculated for $[C_{23}H_{19}F_3O_3–H]^-$ requires *m*/*z* = 399.1208, found *m*/*z* = 399.1213 (ESI–).
Optical: $[\alpha]^{20}_{D}$ = +40.4° (*c* = 0.68, $CHCl_3$, 91:9 er)
HPLC (Chiralpak AD-H, 20% ^{i}PrOH/Hx eluent, 1 mL/min, 254 nm): major enantiomer t_R = 9.5 min, minor enantiomer t_R = 16.0 min.

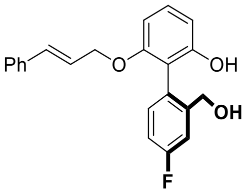

6-(Cinnamyloxy)-4'-fluoro-2'-(hydroxymethyl)-[1,1'-biphenyl]-2-ol (3ga) was synthesized by following Procedure 11 from **1g**. The crude material was purified by normal-phase column chromatography using an eluent of 9–33% EtOAc/Hx to provide **3ga** (39% yield).

^{1}H NMR (400 MHz, $CDCl_3$) δ 7.35 (dd, *J* = 9.4, 2.6 Hz, 1H), 7.29 (dd, *J* = 14.2, 6.5 Hz, 2H), 7.25–7.23 (m, 2H), 7.22 (d, *J* = 1.8 Hz, 1H), 7.21 (d, *J* = 2.6 Hz, 1H), 7.19 (d, *J* = 4.0 Hz, 1H), 7.10 (td, *J* = 8.3, 2.7 Hz, 1H), 6.65 (d, *J* = 7.7 Hz, 1H), 6.60 (d, *J* = 8.3 Hz, 1H), 6.41 (d, *J* = 16.0 Hz, 1H), 6.15 (dt, *J* = 16.0, 5.5 Hz, 1H), 4.91 (s, 1H), 4.67–4.51 (m, 2H), 4.38 (q, *J* = 12.6 Hz, 2H).
^{13}C NMR (100 MHz, $CDCl_3$) δ 164.5, 162.0, 155.4 (d, *J* = 245.6 Hz), 143.6 (d, *J* = 7.2 Hz), 136.3, 133.0 (d, *J* = 8.0 Hz), 132.8, 129.9, 128.7 (2C), 128.1, 126.8 (d, *J* = 3.4 Hz), 126.6 (2C), 124.0, 116.3 (d, *J* = 21.8 Hz), 115.6 (d, *J* = 7.5 Hz), 115.4, 109.4, 105.2, 69.4, 63.3.
IR (FT-ATR, cm^{-1}, CH_2Cl_2) ν_{max} 3301, 3060, 2954, 2923, 2853, 1604, 1587, 1459, 1417, 1378, 1302, 1259, 1223, 1184, 1150, 1099, 1057, 1007, 967, 872, 822, 785, 734, 693, 635, 571, 528, 505, 477, 448.
HRMS: Exact mass calculated for $[C_{22}H_{19}FO_3–H]^-$ requires *m*/*z* = 349.1240, found *m*/*z* = 349.1246 (ESI–).
Optical: $[\alpha]^{20}_{D}$ = +40.6 (*c* = 0.86, $CHCl_3$, 92:8 er)
HPLC (Chiralpak AD-H, 20% ^{i}PrOH/Hx eluent, 1 mL/min, 254 nm): major enantiomer t_R = 11.7 min, minor enantiomer t_R = 21.5 min.

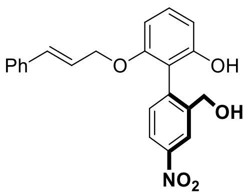

6-(Cinnamyloxy)-2'-(hydroxymethyl)-4'-nitro-[1,1'-biphenyl]-2-ol (3ha) was synthesized by following Procedure 11 from **1h**. The crude material was purified by normal-phase column chromatography using an eluent of 9–33% EtOAc/Hx to provide **3ha** (37% yield).

^{1}H NMR (400 MHz, $CDCl_3$) δ 8.50 (d, *J* = 2.4 Hz, 1H), 8.23 (dd, *J* = 8.4, 2.4 Hz, 1H), 7.43 (d, *J* = 8.4 Hz, 1H), 7.29 (dd, *J* = 10.8, 5.5 Hz, 4H), 7.26–7.20 (m, 2H), 6.65 (t, *J* = 7.8 Hz, 2H), 6.45 (d, *J* = 16.0 Hz, 1H), 6.16 (dt, *J* = 16.0, 5.6 Hz, 1H), 5.21 (s, 1H), 4.62 (dd, *J* = 5.6, 1.1 Hz, 2H), 4.59–4.46 (m, 2H).
^{13}C NMR (100 MHz, $CDCl_3$) δ 156.4, 153.6, 148.2, 142.5, 139.3, 136.1, 133.2, 132.7, 130.6, 128.8 (2C), 128.2, 126.6 (2C), 123.8, 123.7, 122.9, 114.9, 109.7, 105.4, 69.5, 63.0.
IR (FT-ATR, cm^{-1}, CH_2Cl_2) ν_{max} 3319, 2924, 2854, 1609, 1590, 1517, 1459, 1342, 1263, 1185, 1158, 1097, 1054, 1005, 966, 925, 900, 839, 808, 784, 730, 692, 617, 571, 436.
HRMS: Exact mass calculated for $[C_{22}H_{19}NO_5-H]^-$ requires *m*/*z* = 376.1183, found *m*/*z* = 376.1191 (ESI−).
Optical: $[\alpha]^{20}_D$ = +10.2° (*c* = 0.40, $CHCl_3$, 88:12 er)
HPLC (Chiralpak AD-H, 20% ^{i}PrOH/Hx eluent, 1 mL/min, 254 nm): major enantiomer t_R = 15.8 min, minor enantiomer t_R = 34.4 min.

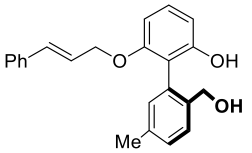

6-(Cinnamyloxy)-2'-(hydroxymethyl)-5'-methyl-[1,1'-biphenyl]-2-ol (3ia) was synthesized by following Procedure 11 from **1i**. The crude material was purified by normal-phase column chromatography using an eluent of 9–33% EtOAc/Hx to provide **3ia** (29% yield).

^{1}H NMR (400 MHz, $CDCl_3$) δ 7.50 (d, *J* = 7.8 Hz, 1H), 7.27 (d, *J* = 2.6 Hz, 2H), 7.24 (m, 3H), 7.21 (dd, *J* = 8.4, 4.4 Hz, 2H), 7.06 (s, 1H), 6.68 (d, *J* = 8.1 Hz, 1H), 6.62 (d, *J* = 8.2 Hz, 1H), 6.42 (d, *J* = 16.0 Hz, 1H), 6.16 (dt, *J* = 16.0, 5.5 Hz, 1H), 5.03 (s, 1H), 4.67–4.55 (m, 2H), 4.44–4.30 (m, 2H), 2.37 (s, 3H).
^{13}C NMR (100 MHz, $CDCl_3$) δ 156.4, 154.2, 138.6, 138.0, 136.4, 132.8, 131.8, 131.3, 130.1, 130.0, 129.6, 128.7 (2C), 128.0, 126.6 (2C), 124.1, 116.8, 109.4, 105.3, 69.5, 63.7, 21.3.
IR (FT-ATR, cm^{-1}, CH_2Cl_2) ν_{max} 3300, 3024, 2922, 2853, 1713, 1610, 1584, 1494, 1456, 1377, 1303, 1257, 1192, 1157, 1131, 1101, 1056, 986, 966, 882, 825, 783, 735, 692, 608, 579, 542, 496, 451.
HRMS: Exact mass calculated for $[C_{23}H_{22}O_3-H]^-$ requires *m*/*z* = 345.1488, found *m*/*z* = 345.1497 (ESI−).
Optical: $[\alpha]^{20}_D$ = +18.0° (*c* = 0.62, $CHCl_3$, 80:20 er)

HPLC (Chiralpak AD, 20% ^{i}PrOH/Hx eluent, 1 mL/min, 254 nm): major enantiomer t_R = 10.4 min, minor enantiomer t_R = 21.1 min.

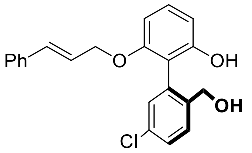

5'-Chloro-6-(cinnamyloxy)-2'-(hydroxymethyl)-[1,1'-biphenyl]-2-ol (3ja) was synthesized by following Procedure 11 from **1j**. The crude material was purified by normal-phase column chromatography using an eluent of 9–33% EtOAc/Hx to provide **3ja** (36% yield).

^{1}H NMR (400 MHz, $CDCl_3$) δ 7.57 (d, *J* = 8.4 Hz, 1H), 7.45 (d, *J* = 8.2 Hz, 1H), 7.32–7.22 (m, 7H), 6.68 (d, *J* = 8.2 Hz, 1H), 6.64 (d, *J* = 8.2 Hz, 1H), 6.46 (d, *J* = 16.3 Hz, 1H), 6.19 (dt, *J* = 10.1, 4.6 Hz, 1H), 5.03 (s, 1H), 4.63 (s, 2H), 4.40 (q, *J* = 12.3 Hz, 2H).
^{13}C NMR (100 MHz, $CDCl_3$) δ 156.4, 154.0, 139.2, 136.3, 134.1, 133.6, 132.8, 131.3, 131.1, 130.1, 129.1, 128.7 (2C), 128.1, 126.7 (2C), 123.9, 115.6, 109.6, 105.3, 69.4, 63.3.
IR (FT-ATR, cm^{-1}, CH_2Cl_2) ν_{max} 3285, 3059, 3025, 2924, 2854, 1608, 1593, 1495, 1458, 1379, 1302, 1250, 1184, 1159, 1096, 1057, 1017, 966, 884, 849, 822, 786, 735, 692, 660, 608, 575, 532, 493, 442, 423.
HRMS: Exact mass calculated for $[C_{22}H_{19}ClO_3{-}H]^-$ requires *m*/*z* = 365.0942, found *m*/*z* = 365.0950 (ESI−).
Optical: $[\alpha]^{20}_D$ = –8.9° (*c* = 0.85, $CHCl_3$, 84:16 er)
HPLC (Chiralpak AD-H, 20% ^{i}PrOH/Hx eluent, 1 mL/min, 254 nm): major enantiomer t_R = 10.6 min, minor enantiomer t_R = 13.2 min.

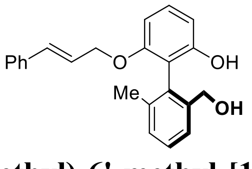

6-(Cinnamyloxy)-2'-(hydroxymethyl)-6'-methyl-[1,1'-biphenyl]-2-ol (3ka) was synthesized by following Procedure 11 from **1k**. The crude material was purified by normal-phase column chromatography using an eluent of 9–33% EtOAc/Hx to provide **3ka** (37% yield).

^{1}H NMR (400 MHz, $CDCl_3$) δ 7.39 (dt, *J* = 15.2, 7.1 Hz, 2H), 7.31 (d, *J* = 8.1 Hz, 1H), 7.26 (t, *J* = 10.5 Hz, 6H), 6.68 (d, *J* = 8.2 Hz, 1H), 6.64 (d, *J* = 8.2 Hz, 1H), 6.38 (d, *J* = 15.9 Hz, 1H), 6.16 (dt, *J* = 10.3, 4.9 Hz, 1H), 5.02 (s, 1H), 4.61 (s, 2H), 4.35 (s, 2H), 2.06 (s, 3H).
^{13}C NMR (100 MHz, $CDCl_3$) δ 156.2, 153.8, 140.7, 138.8, 136.4, 132.3, 130.8, 130.2, 129.7, 129.0, 128.7 (2C), 127.9, 127.0, 126.6 (2C), 124.2, 115.2, 109.3, 105.2, 67.0, 64.4, 20.0.
IR (FT-ATR, cm^{-1}, CH_2Cl_2) ν_{max} 3284, 3060, 3025, 2922, 2853, 1608, 1579, 1495, 1451, 1377, 1303, 1256, 1183, 1157, 1099, 1055, 1003, 987, 964, 906, 842, 801, 782, 729, 691, 607, 581, 557, 525, 492.
HRMS: Exact mass calculated for $[C_{23}H_{22}O_3{-}H]^-$ requires *m*/*z* = 345.1491, found *m*/*z* = 345.1496 (ESI−).

Optical: $[\alpha]^{20}_{D}$ = +21.2° (c = 0.85, $CHCl_3$, 94:6 er)
HPLC (Chiralpak AD, 20% ^{i}PrOH/Hx eluent, 1 mL/min, 254 nm): major enantiomer t_R = 9.0 min, minor enantiomer t_R = 10.9 min.

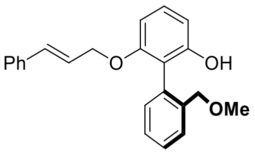

6-(Cinnamyloxy)-2'-(methoxymethyl)-[1,1'-biphenyl]-2-ol (3la) was synthesized by following Procedure 11 from **1l**. The crude material was purified by normal-phase column chromatography using an eluent of 9–33% EtOAc/Hx to provide **3la** (45% yield).

^{1}H NMR (400 MHz, $CDCl_3$) δ 7.60–7.55 (m, 1H), 7.47–7.39 (m, 2H), 7.28 (t, J = 5.3 Hz, 2H), 7.26–7.24 (m, 3H), 7.21 (m, 2H), 6.72–6.68 (m, 1H), 6.61 (d, J = 8.2 Hz, 1H), 6.37 (d, J = 16.0 Hz, 1H), 6.17 (dt, J = 16.0, 5.1 Hz, 1H), 5.60 (s, 1H), 4.67–4.54 (m, 2H), 4.30 (d, J = 11.3 Hz, 1H), 4.20 (d, J = 11.3 Hz, 1H), 3.28 (s, 3H).
^{13}C NMR (100 MHz, $CDCl_3$) 156.6, 154.5, 137.9, 136.6, 132.3, 131.8, 131.7, 129.5, 129.3, 128.7, 128.62 (2C), 128.58, 127.8, 126.5 (2C), 124.6, 117.3, 109.9, 105.0, 73.2, 68.9, 58.6.
IR (FT-ATR, cm^{-1}, CH_2Cl_2) ν_{max} 3319, 3059, 3025, 2924, 1609, 1581, 1495, 1459, 1449, 1378, 1305, 1253, 1185, 1156, 1083, 1058, 1005, 965, 784, 762, 729, 691, 610, 577, 537.
HRMS: Exact mass calculated for $[C_{23}H_{22}O_3{-}H]^-$ requires m/z = 345.1489, found m/z = 345.1492 (ESI−).
Optical: $[\alpha]^{20}_{D}$ = +21.4° (c = 0.93, $CHCl_3$, 88:12 er)
HPLC (Chiralpak AD-H, 20% ^{i}PrOH/Hx eluent, 1 mL/min, 254 nm): major enantiomer t_R = 6.8 min, minor enantiomer t_R = 8.4 min.

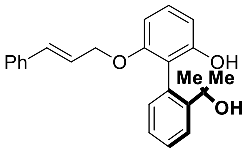

6-(Cinnamyloxy)-2'-(2-hydroxypropan-2-yl)-[1,1'-biphenyl]-2-ol (3ma) was synthesized by following Procedure 11 from **1m**. The crude material was purified by normal-phase column chromatography using an eluent of 9–33% EtOAc/Hx to provide **3ma** (46% yield).

^{1}H NMR (400 MHz, $CDCl_3$) δ 7.70 (d, J = 7.9 Hz, 1H), 7.43 (t, J = 7.6 Hz, 1H), 7.36 (t, J = 7.4 Hz, 1H), 7.31–7.20 (m, 6H), 7.11 (d, J = 7.4 Hz, 1H), 6.67 (d, J = 8.2 Hz, 1H), 6.60 (d, J = 8.3 Hz, 1H), 6.36 (d, J = 16.0 Hz, 1H), 6.18 (dt, J = 15.9, 4.8 Hz, 1H), 4.68–4.58 (m, 2H), 1.53 (s, 3H), 1.48 (s, 3H).
^{13}C NMR (100 MHz, $CDCl_3$) δ 156.3, 154.0, 148.1, 136.6, 133.6, 131.8, 130.2, 129.4, 128.6 (3C), 127.89, 127.85, 126.6, 126.5 (2C), 124.4, 120.4, 109.4, 104.7, 73.6, 68.7, 31.1, 31.0.

IR (FT-ATR, cm^{-1}, CH_2Cl_2) ν_{max} 3303, 3056, 3024, 2969, 2925, 2854, 1607, 1579, 1496, 1459, 1379, 1332, 1306, 1264, 1248, 1185, 1156, 1104, 1058, 1005, 967, 944, 857, 785, 759, 732, 692, 607, 566, 488.
HRMS: Exact mass calculated for $[C_{24}H_{24}O_3{-}H]^-$ requires *m/z* = 359.1647, found *m/z* = 359.1650 (ESI−).
Optical: $[\alpha]^{20}_D$ = +47.4° (*c* = 1.07, $CHCl_3$, 88:12 er)
HPLC (Chiralpak AD-H, 20% ^{i}PrOH/Hx eluent, 1 mL/min, 254 nm): major enantiomer t_R = 7.6 min, minor enantiomer t_R = 13.0 min.

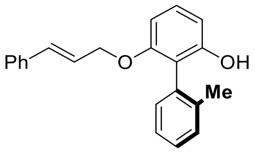

6-(Cinnamyloxy)-2'-methyl-[1,1'-biphenyl]-2-ol (3na) was synthesized by following Procedure 11 from **1n**. The crude material was purified by normal-phase column chromatography using an eluent of 0–14% EtOAc/Hx to provide **3na** (46% yield).

^{1}H NMR (400 MHz, $CDCl_3$) δ 7.37–7.33 (m, 2H), 7.32–7.28 (m, 2H), 7.27 (d, *J* = 3.0 Hz, 1H), 7.23 (ddd, *J* = 5.4, 3.6, 1.4 Hz, 3H), 7.21–7.18 (m, 2H), 6.66 (dd, *J* = 8.2, 0.8 Hz, 1H), 6.59 (dd, *J* = 8.2, 0.6 Hz, 1H), 6.41 (dt, *J* = 16.0, 1.6 Hz, 1H), 6.19 (dt, *J* = 16.0, 5.1 Hz, 1H), 4.62 (dd, *J* = 5.1, 1.6 Hz, 2H), 2.13 (s, 3H).
^{13}C NMR (100 MHz, $CDCl_3$) δ 156.6, 153.8, 138.9, 136.7, 131.8, 131.7, 131.1, 130.8, 129.3, 128.7, 128.67 (2C), 127.8, 126.6, 126.56 (2C), 124.7, 117.2, 108.5, 104.8, 68.9, 19.8.
IR (FT-ATR, cm^{-1}, CH_2Cl_2) ν_{max} 3757, 3529, 1948, 1574, 1450, 1381, 1331, 1257, 1184, 1103, 1061, 968, 733, 694, 451, 432.
HRMS: Exact mass calculated for $[C_{22}H_{20}O_2]$ requires *m/z* = 316.1463, found *m/z* = 316.1459 (EI).
Optical: $[\alpha]^{20}_D$ = +47.1° (c = 0.07, $CHCl_3$, 83:17 er)
HPLC (Chiralpak AD-H, 20% ^{i}PrOH/Hx eluent, 1 mL/min, 254 nm): major enantiomer t_R = 5.8 min, minor enantiomer t_R = 7.6 min.

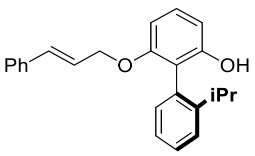

6-(Cinnamyloxy)-2'-isopropyl-[1,1'-biphenyl]-2-ol (3oa) was synthesized by following Procedure 11 from **1o**. The crude material was purified by normal-phase column chromatography using an eluent of 0–10% EtOAc/Hx to provide **3oa** (47% yield).

^{1}H NMR (400 MHz, $CDCl_3$) δ 7.45 (dt, *J* = 15.1, 7.8 Hz, 3H), 7.32–7.27 (m, 3H), 7.25–7.21 (m, 2H), 7.19 (dd, *J* = 8.6, 5.0 Hz, 2H), 6.67 (d, *J* = 8.2 Hz, 1H), 6.59 (d, *J* = 8.2 Hz, 1H), 6.40 (d, *J* = 16.0 Hz, 1H), 6.19 (dt, *J* = 16.0, 5.1 Hz, 1H), 4.62 (d, *J* = 5.0 Hz, 2H), 2.76 (dt, *J* = 13.7, 6.8 Hz, 1H), 1.12 (dd, *J* = 21.4, 6.9 Hz, 6H).

^{13}**C NMR** (100 MHz, $CDCl_3$) δ 156.7, 154.1, 149.7, 136.7, 131.8, 131.2, 130.3, 129.2, 129.15, 128.7 (2C), 127.8, 126.6, 126.5 (2C), 126.3, 124.7, 117.1, 108.3, 104.4, 68.7, 30.5, 24.5, 23.7.
IR (FT-ATR, cm^{-1}, CH_2Cl_2) ν_{max} 3900, 3889, 3883, 3527, 3493, 3464, 3431, 3418, 3406, 3381, 3080, 3059, 3023, 2960, 2927, 2887, 2867, 1612, 1585, 1496, 1458, 1381, 1362, 1329, 1307, 1257, 1202, 1184, 1157, 1104, 1059, 1035, 1005, 967, 784, 762, 731, 689.
HRMS: Exact mass calculated for [$C_{24}H_{24}O_2$] requires *m/z* = 344.1776, found *m/z* = 344.1775 (EI).
Optical: $[\alpha]^{20}_D$ = +32.8° (c = 0.51, $CHCl_3$, 85:15 er)
HPLC (Chiralpak AD-H, 7% ^{i}PrOH/Hx eluent, 1 mL/min, 254 nm): major enantiomer t_R = 8.7 min, minor enantiomer t_R = 16.0 min.

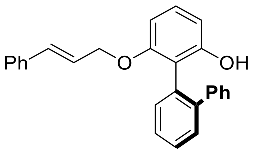

6-(Cinnamyloxy)-[1,1':2',1''-terphenyl]-2-ol (3pa) was synthesized by following Procedure 11 from **1p**. The crude material was purified by normal-phase column chromatography using an eluent of 0–9% EtOAc/Hx to provide **3pa** (42% yield).

^{1}**H NMR** (400 MHz, $CDCl_3$) δ 7.65–7.53 (m, 4H), 7.51–7.45 (m, 1H), 7.43–7.29 (m, 8H), 7.26–7.23 (m, 1H), 7.18 (t, *J* = 8.3 Hz, 1H), 6.64 (dd, *J* = 8.2, 0.8 Hz, 1H), 6.45 (dd, *J* = 11.8, 7.0 Hz, 2H), 6.12 (dt, *J* = 16.0, 5.2 Hz, 1H), 5.01 (s, 1H), 4.55 (ddd, *J* = 13.5, 5.1, 1.6 Hz, 1H), 4.35 (ddd, *J* = 13.5, 5.3, 1.6 Hz, 1H).
^{13}**C NMR** (100 MHz, $CDCl_3$) δ 156.3, 154.0, 143.6, 141.1, 136.7, 131.7, 131.7, 130.8, 129.2, 128.9, 128.8 (2C), 128.7 (2C), 128.1, 127.79, 127.75 (2C), 127.0, 126.6 (2C), 125.0, 117.4, 108.2, 104.4, 68.6, 29.8.
IR (FT-ATR, cm^{-1}, CH_2Cl_2) ν_{max} 3528, 3492, 3430, 3420, 3382, 3354, 3333, 3101, 3080, 3059, 3023, 2955, 2922, 2852, 1613, 1586, 1496, 1458, 1379, 1329, 1308, 1256, 1200, 1183, 1157, 1104, 1061, 1007, 967, 784, 763, 732, 690.
HRMS: Exact mass calculated for [$C_{27}H_{22}O_2$] requires *m/z* = 378.1620, found *m/z* = 378.1617 (EI).
Optical: $[\alpha]^{20}_D$ = +80.8° (c = 0.11, $CHCl_3$, 87:13 er)
HPLC (Chiralpak AD-H, 10% ^{i}PrOH/Hx eluent, 1 mL/min, 254 nm): major enantiomer t_R = 10.5 min, minor enantiomer t_R = 13.9 min.

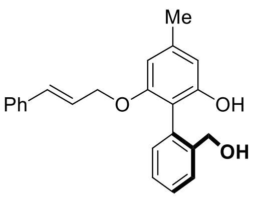

6-(Cinnamyloxy)-2'-(hydroxymethyl)-4-methyl-[1,1'-biphenyl]-2-ol (3qa) was synthesized by following Procedure 11 from **1q**. The crude material was purified by normal-phase column chromatography using an eluent of 9–33% EtOAc/Hx to provide **3qa** (45% yield).

^{1}H NMR (400 MHz, $CDCl_3$) δ 7.62 (d, *J* = 7.3 Hz, 1H), 7.45 (dt, *J* = 15.2, 7.4 Hz, 2H), 7.31–7.21 (m, 6H), 6.53 (s, 1H), 6.46 (s, 1H), 6.41 (d, *J* = 15.9 Hz, 1H), 6.17 (dt, *J* = 15.9, 5.3 Hz, 1H), 5.00 (s, 1H), 4.65–4.54 (m, 2H), 4.43 (q, *J* = 11.9 Hz, 2H), 2.36 (s, 3H).
^{13}C NMR (100 MHz, $CDCl_3$) δ 156.2, 153.8, 141.1, 140.1, 136.4, 132.5, 131.6, 131.5, 130.0, 129.1, 128.71, 128.66 (2C), 128.0, 126.6 (2C), 124.1, 113.8, 110.1, 106.4, 69.4, 64.0, 22.0.
IR (FT-ATR, cm^{-1}, CH_2Cl_2) ν_{max} 3354, 3058, 3026, 2920, 2856, 1615, 1587, 1518, 1483, 1447, 1417, 1379, 1359, 1324, 1281, 1264, 1252, 1221, 1196, 1163, 1083, 1033, 1004, 990, 964, 869, 847, 814, 768, 733, 692, 662, 633, 611, 590, 561, 542, 520, 503, 439.
HRMS: Exact mass calculated for $[C_{23}H_{22}O_3-H]^-$ requires *m*/*z* = 345.1488, found *m*/*z* = 345.1496 (ESI−).
Optical: $[\alpha]^{20}_D$ = +41.9° (*c* = 0.97, $CHCl_3$, 98:2 er)
HPLC (Chiralpak AD-H, 20% ^{i}PrOH/Hx eluent, 1 mL/min, 254 nm): major enantiomer t_R = 18.2 min, minor enantiomer t_R = 27.0 min.

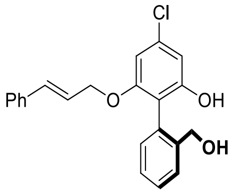

4-Chloro-6-(cinnamyloxy)-2'-(hydroxymethyl)-[1,1'-biphenyl]-2-ol (**3ra**) was synthesized by following Procedure 11 from **1r**. The crude material was purified by normal-phase column chromatography using an eluent of 9–33% EtOAc/Hx to provide **3ra** (54% yield).

^{1}H NMR (400 MHz, CD_3OD) δ 7.60 (d, *J* = 7.6 Hz, 1H), 7.39 (t, *J* = 7.5 Hz, 1H), 7.31 (t, *J* = 7.6 Hz, 1H), 7.26 (d, *J* = 4.5 Hz, 4H), 7.19 (d, *J* = 4.5 Hz, 1H), 7.14 (d, *J* = 7.5 Hz, 1H), 6.64 (s, 1H), 6.59 (s, 1H), 6.39 (d, *J* = 16.2 Hz, 1H), 6.21 (dt, *J* = 16.0, 5.0 Hz, 1H), 4.57 (d, *J* = 4.8 Hz, 2H), 4.44 (s, 2H).
^{13}C NMR (100 MHz, CD_3OD) δ 158.8, 157.2, 141.8, 138.0, 135.1, 133.2, 132.9, 132.2, 129.5 (2C), 128.7, 128.5, 127.7, 127.5, 127.4 (2C), 125.1, 117.0, 110.1, 105.9, 69.9, 63.1.
IR (FT-ATR, cm^{-1}, CH_2Cl_2) ν_{max} 3246, 3060, 3026, 2924, 2854, 1595, 1579, 1496, 1482, 1448, 1415, 1378, 1319, 1265, 1203, 1169, 1112, 1077, 1031, 1004, 964, 889, 872, 830, 769, 748, 737, 693, 656, 634, 609, 591, 561, 544, 506, 437.
HRMS: Exact mass calculated for $[C_{22}H_{19}ClO_3-H]^-$ requires *m*/*z* = 365.0942, found *m*/*z* = 365.0950 (ESI−).
Optical: $[\alpha]^{20}_D$ = −81.7° (*c* = 0.59, $CHCl_3$, 91:9 er)
HPLC (Chiralpak AD-H, 20% ^{i}PrOH/Hx eluent, 1 mL/min, 254 nm): major enantiomer t_R = 15.1 min, minor enantiomer t_R = 21.0 min.

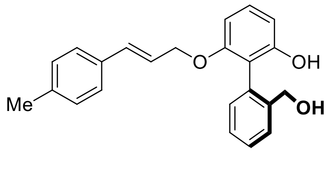

(*E*)-2'-(hydroxymethyl)-6-((3-(*p*-tolyl)allyl)oxy)-[1,1'-biphenyl]-2-ol (3ab) was synthesized by following Procedure 11 from **2b**. The crude material was purified by normal-phase column chromatography using an eluent of 9–33% EtOAc/Hx to provide **3ab** (47% yield).

^{1}H NMR (400 MHz, $CDCl_3$) δ 7.64–7.60 (m, 1H), 7.50–7.40 (m, 2H), 7.27 (dd, *J* = 5.7, 1.2 Hz, 1H), 7.23 (d, *J* = 8.3 Hz, 1H), 7.17 (d, *J* = 8.1 Hz, 2H), 7.09 (d, *J* = 8.0 Hz, 2H), 6.69 (d, *J* = 8.1 Hz, 1H), 6.64 (d, *J* = 8.3 Hz, 1H), 6.40 (d, *J* = 16.0 Hz, 1H), 6.11 (dt, *J* = 15.9, 5.5 Hz, 1H), 5.14 (s, 1H), 4.65–4.55 (m, 2H), 4.42 (q, *J* = 12.1 Hz, 2H), 2.32 (s, 3H).

^{13}C NMR (100 MHz, $CDCl_3$) δ 156.5, 154.2, 140.9, 137.9, 133.6, 132.7, 131.5, 131.3, 129.9, 129.6, 129.4 (2C), 129.2, 128.7, 126.6 (2C), 123.0, 116.7, 109.4, 105.4, 69.6, 63.9, 21.3.

IR (FT-ATR, cm^{-1}, CH_2Cl_2) ν_{max} 3296, 3022, 2921, 1610, 1589, 1512, 1458, 1377, 1305, 1254, 1182, 1158, 1099, 1057, 1005, 968, 831, 784, 760, 731, 701, 608, 560, 505, 432, 418, 410.

HRMS: Exact mass calculated for $[C_{23}H_{22}O_3-H]^-$ requires *m*/*z* = 345.1488, found *m*/*z* = 345.1496 (ESI−).

Optical: $[\alpha]^{20}_D$ = +34.6° (*c* = 1.07, $CHCl_3$, 96:4 er)

HPLC (Chiralpak AD-H, 20% ^{i}PrOH/Hx eluent, 1 mL/min, 254 nm): major enantiomer t_R = 11.9 min, minor enantiomer t_R = 18.0 min.

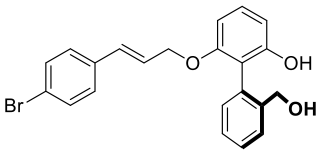

(*E*)-6-((3-(4-bromophenyl)allyl)oxy)-2'-(hydroxymethyl)-[1,1'-biphenyl]-2-ol (3ac) was synthesized by following Procedure 11 from **2c**. The crude material was purified by normal-phase column chromatography using an eluent of 9–33% EtOAc/Hx to provide **3ac** (44% yield).

^{1}H NMR (400 MHz, $CDCl_3$) δ 7.63 (d, J = 7.3 Hz, 1H), 7.51–7.41 (m, 2H), 7.40 (d, J = 8.4 Hz, 2H), 7.28–7.21 (m, 2H), 7.11 (d, J = 8.4 Hz, 2H), 6.71–6.68 (m, 1H), 6.62 (d, J = 8.1 Hz, 1H), 6.32 (d, J = 16.0 Hz, 1H), 6.15 (dt, J = 16.0, 5.2 Hz, 1H), 5.09 (s, 1H), 4.65–4.55 (m, 2H), 4.43 (q, J = 12.1 Hz, 2H).

^{13}C NMR (100 MHz, $CDCl_3$) δ 156.4, 154.2, 140.9, 135.4, 131.8 (2C), 131.4, 131.3, 131.2, 129.9, 129.7, 129.2, 128.8, 128.1 (2C), 124.9, 121.8, 116.7, 109.6, 105.3, 69.2, 63.9.

IR (FT-ATR, cm^{-1}, CH_2Cl_2) ν_{max} 3258, 3057, 2924, 2853, 1720, 1609, 1588, 1486, 1459, 1401, 1376, 1302, 1263, 1183, 1158, 1103, 1058, 1006, 967, 837, 783, 761, 730, 702, 607, 580, 563, 499, 470, 422.

HRMS: Exact mass calculated for $[C_{22}H_{19}BrO_3-H]^-$ requires *m*/*z* = 409.0439, found *m*/*z* = 409.0445 (ESI−).

Optical: $[\alpha]^{20}_D$ = +29.5° (*c* = 1.25, $CHCl_3$, 96:4 er)

HPLC (Chiralpak IC, 10% ^{i}PrOH/Hx eluent, 1 mL/min, 254 nm): major enantiomer t_R = 12.7 min, minor enantiomer t_R = 14.6 min.

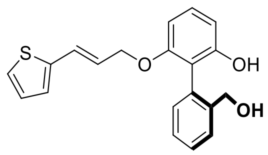

(*E*)-2'-(hydroxymethyl)-6-((3-(thiophen-2-yl)allyl)oxy)-[1,1'-biphenyl]-2-ol (3ad) was synthesized by following Procedure 11 from **2d**. The crude material was purified by normal-phase column chromatography using an eluent of 9–33% EtOAc/Hx to provide **3ad** (58% yield).

^{1}H NMR (400 MHz, $CDCl_3$) δ 7.63 (d, J = 7.2 Hz, 1H), 7.46 (dt, J = 15.1, 7.4 Hz, 2H), 7.25 (dd, J = 13.9, 6.5 Hz, 2H), 7.14 (d, J = 5.0 Hz, 1H), 6.94–6.90 (m, 1H), 6.85 (d, J = 2.8 Hz, 1H), 6.69 (d, J = 8.2 Hz, 1H), 6.62 (d, J = 8.2 Hz, 1H), 6.50 (d, J = 15.7 Hz, 1H), 5.99 (dt, J = 15.7, 5.4 Hz, 1H), 5.13 (s, 1H), 4.62–4.52 (m, 2H), 4.42 (q, J = 12.1 Hz, 2H), 2.24 (s, 1H).
^{13}C NMR (100 MHz, $CDCl_3$) δ 156.4, 154.2, 141.5, 140.8, 131.4, 131.3, 129.9, 129.7, 129.2, 128.8, 127.5, 126.3, 125.8, 124.8, 123.5, 116.7, 109.6, 105.4, 69.1, 63.9.
IR (FT-ATR, cm^{-1}, CH_2Cl_2) ν_{max} 3279, 2923, 2853, 1649, 1609, 1587, 1505, 1459, 1378, 1303, 1253, 1200, 1183, 1158, 1099, 1056, 1040, 1005, 954, 854, 832, 784, 761, 730, 698, 608, 565, 539, 493, 462, 437.
HRMS: Exact mass calculated for $[C_{20}H_{18}O_3S{-}H]^-$ requires *m*/*z* = 337.0896, found *m*/*z* = 337.0903 (ESI−).
Optical: $[\alpha]^{20}_{D}$ = +40.7° (*c* = 1.21, $CHCl_3$, 96:4 er)
HPLC (Chiralpak AD-H, 20% ^{i}PrOH/Hx eluent, 1 mL/min, 254 nm): major enantiomer t_R = 11.0 min, minor enantiomer t_R = 19.8 min.

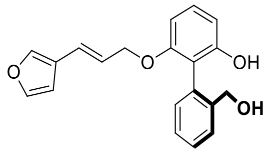

(*E*)-6-((3-(furan-3-yl)allyl)oxy)-2'-(hydroxymethyl)-[1,1'-biphenyl]-2-ol (3ae) was synthesized by following Procedure 11 using 5 mol% of $Pd(dba)_2$ 5 mol% and 20 mol% of **L4** from **2e**. The crude material was purified by normal-phase column chromatography using an eluent of 9–33% EtOAc/Hx to provide **3ae** (29% yield).

^{1}H NMR (400 MHz, $CDCl_3$) δ 7.61 (d, J = 7.1 Hz, 1H), 7.45 (dt, J = 14.4, 7.3 Hz, 2H), 7.33 (s, 2H), 7.28–7.20 (m, 2H), 6.68 (d, J = 8.1 Hz, 1H), 6.62 (d, J = 8.3 Hz, 1H), 6.44 (s, 1H), 6.28 (d, J = 15.8 Hz, 1H), 5.89 (dt, J = 10.5, 5.0 Hz, 1H), 5.22 (s, 1H), 4.61–4.49 (m, 2H), 4.40 (q, J = 12.1 Hz, 2H), 2.36 (s, 1H).
^{13}C NMR (100 MHz, $CDCl_3$) δ 156.5, 154.2, 143.7, 141.0, 140.8, 131.5, 131.3, 130.0, 129.6, 129.1, 128.7, 123.6, 123.5, 122.8, 116.7, 109.5, 107.6, 105.4, 69.5, 63.9.
IR (FT-ATR, cm^{-1}, CH_2Cl_2) ν_{max} 3286, 3059, 3023, 2924, 2854, 1664, 1609, 1588, 1507, 1458, 1373, 1302, 1254, 1184, 1158, 1099, 1056, 1021, 1005, 964, 871, 783, 762, 729, 701, 595, 540, 465, 440.
HRMS: Exact mass calculated for $[C_{20}H_{18}O_4{-}H]^-$ requires *m*/*z* = 321.1125, found *m*/*z* = 321.1132 (ESI−).
Optical: $[\alpha]^{20}_{D}$ = +30.7° (*c* = 0.37, $CHCl_3$, 91:9 er)
HPLC (Chiralpak AD-H, 10% ^{i}PrOH/Hx eluent, 1 mL/min, 254 nm): major enantiomer t_R = 30.9 min, minor enantiomer t_R = 62.9 min.

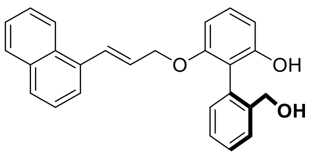

(*E*)-2'-(hydroxymethyl)-6-((3-(naphthalen-1-yl)allyl)oxy)-[1,1'-biphenyl]-2-ol (3af) was synthesized by following Procedure 12 from **2f**. The crude material was purified by normal-phase column chromatography using an eluent of 9–33% EtOAc/Hx to provide **3af** (39% yield).

^{1}H NMR (400 MHz, $CDCl_3$) δ 7.85–7.73 (m, 3H), 7.67 (d, J = 6.4 Hz, 1H), 7.53–7.44 (m, 5H), 7.40 (t, J = 7.7 Hz, 1H), 7.32 (d, J = 8.1 Hz, 1H), 7.30 (d, J = 8.5 Hz, 1H), 7.15 (d, J = 16.1 Hz, 1H), 6.71 (dd, J = 13.5, 8.4 Hz, 2H), 6.23 (dt, J = 16.0, 4.6 Hz, 1H), 5.04 (s, 1H), 4.79–4.66 (m, 2H), 4.53–4.42 (m, 2H).
^{13}C NMR (100 MHz, $CDCl_3$) δ 156.4, 154.2, 141.0, 134.2, 133.7, 131.5, 131.4, 131.2, 129.9, 129.7, 129.2, 129.1, 128.8, 128.6, 128.2, 127.0, 126.1, 125.9, 125.7, 123.9, 123.7, 116.6, 109.5, 105.1, 69.0, 64.0.
IR (FT-ATR, cm^{-1}, CH_2Cl_2) ν_{max} 3273, 3050, 2923, 2853, 1610, 1590, 1508, 1461, 1379, 1347, 1304, 1263, 1183, 1158, 1102, 1058, 1022, 1005, 969, 861, 783, 762, 731, 702, 613, 548, 526, 421.
HRMS: Exact mass calculated for $[C_{26}H_{22}O_3+Na]^+$ requires *m*/*z* = 405.1467, found *m*/*z* = 405.1462 (ESI+).
Optical: $[\alpha]^{20}_{D}$ = +7.6° (*c* = 0.11, $CHCl_3$, 75:25 er)
HPLC (Chiralpak IC, 10% ^{i}PrOH/Hx eluent, 1 mL/min, 254 nm): major enantiomer t_R = 14.9 min, minor enantiomer t_R = 17.3 min.

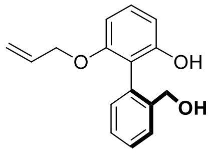

6-(Allyloxy)-2'-(hydroxymethyl)-[1,1'-biphenyl]-2-ol (3ag) was synthesized by following Procedure 11 from **2g**. The crude material was purified by normal-phase column chromatography using an eluent of 9–33% EtOAc/Hx to provide **3ag** (38% yield).

^{1}H NMR (400 MHz, $CDCl_3$) δ 7.61 (d, J = 7.2 Hz, 1H), 7.48–7.40 (m, 2H), 7.27–7.23 (m, 1H), 7.21 (d, J = 8.4 Hz, 1H), 6.68 (d, J = 8.2 Hz, 1H), 6.57 (d, J = 8.3 Hz, 1H), 5.84 (ddd, J = 16.0, 10.3, 4.9 Hz, 1H), 5.13 (s, 1H), 5.09 (d, J = 8.3 Hz, 1H), 4.47–4.43 (m, 2H), 4.43–4.35 (m, 2H).
^{13}C NMR (100 MHz, $CDCl_3$) δ 156.4, 154.1, 140.9, 132.9, 131.4, 131.2, 129.9, 129.6, 129.2, 128.7, 117.4, 116.5, 109.4, 105.0, 69.4, 63.9.
IR (FT-ATR, cm^{-1}, CH_2Cl_2) ν_{max} 3291, 3064, 3021, 2923, 2854, 1647, 1609, 1588, 1506, 1460, 1423, 1378, 1302, 1255, 1225, 1199, 1157, 1114, 1066, 1005, 993, 929, 870, 785, 761, 731, 700, 634, 609, 564, 462, 420.
HRMS: Exact mass calculated for $[C_{16}H_{16}O_3+Na]^+$ requires *m*/*z* = 279.0997, found *m*/*z* = 279.0991 (ESI+).
Optical: $[\alpha]^{20}_{D}$ = +30.2° (c = 0.62, $CHCl_3$, 65:35 er)
HPLC (Chiralpak AD-H, 20% ^{i}PrOH/Hx eluent, 1 mL/min, 254 nm): major enantiomer t_R = 5.7 min, minor enantiomer t_R = 7.5 min.

5.2 Characterization of Products 4

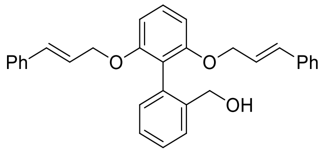

(2',6'-Bis(cinnamyloxy)-[1,1'-biphenyl]-2-yl)methanol (4aa) was synthesized by following Procedure 11 from **1a**. The crude material was purified by normal-phase column chromatography using an eluent of 9–33% EtOAc/Hx to provide **4aa** (42% yield).

^{1}H NMR (400 MHz, $CDCl_3$) δ 7.62 (d, J = 7.2 Hz, 1H), 7.49–7.40 (m, 2H), 7.32 (d, J = 17.8 Hz, 10H), 7.25 (d, J = 6.1 Hz, 2H), 6.76 (d, J = 8.3 Hz, 2H), 6.43 (d, J = 16.0 Hz, 2H), 6.19 (dt, J = 16.0, 5.1 Hz, 2H), 4.64 (d, J = 4.7 Hz, 4H), 4.44 (s, 2H).
^{13}C NMR (100 MHz, $CDCl_3$) δ 156.8 (2C), 139.9, 136.4 (2C), 133.5, 132.3 (2C), 131.4, 129.2, 129.1, 128.6 (4C), 127.90, 127.87 (2C), 127.6, 126.6 (4C), 124.2 (2C), 119.7, 106.8 (2C), 69.4 (2C), 64.3.
IR (FT-ATR, cm^{-1}, CH_2Cl_2) ν_{max} 3438, 3057, 3023, 2923, 2855, 1588, 1495, 1447, 1375, 1297, 1244, 1226, 1180, 1107, 1071, 1044, 1003, 964, 913, 782, 760, 729, 691, 609, 575, 496.
HRMS: Exact mass calculated for $[C_{31}H_{28}O_3+H]^+$ requires m/z = 471.1936, found m/z = 471.1931 (ESI+).

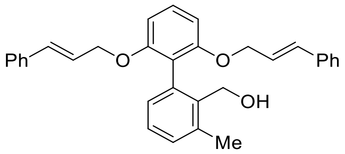

(2',6'-Bis(cinnamyloxy)-3-methyl-[1,1'-biphenyl]-2-yl) methanol (4ba) was synthesized by following Procedure 11 from **1b**. The crude material was purified by normal-phase column chromatography using an eluent of 9–33% EtOAc/Hx to provide **4ba** (45% yield).

^{1}H NMR (400 MHz, $CDCl_3$) δ 7.33–7.25 (m, 11H), 7.23 (d, J = 5.1 Hz, 2H), 7.08 (d, J = 7.0 Hz, 1H), 6.75 (d, J = 8.3 Hz, 2H), 6.42 (d, J = 16.0 Hz, 2H), 6.17 (dt, J = 15.9, 5.3 Hz, 2H), 4.68–4.58 (m, 4H), 4.41 (d, J = 5.9 Hz, 2H), 2.54 (s, 3H).
^{13}C NMR (100 MHz, $CDCl_3$) δ 156.9 (2C), 138.2, 137.9, 136.5 (2C), 134.4, 132.5 (2C), 130.0, 128.99, 128.96, 128.7 (4C), 127.9 (2C), 127.7, 126.6 (4C), 124.2 (2C), 121.0, 107.2 (2C), 69.6 (2C), 61.5, 19.6.
IR (FT-ATR, cm^{-1}, CH_2Cl_2) ν_{max} 3538, 3058, 3024, 2923, 1588, 1494, 1448, 1376, 1334, 1241, 1227, 1198, 1105, 1069, 998, 963, 913, 846, 790, 780, 738, 691, 600, 573, 539, 494.
HRMS: Exact mass calculated for $[C_{32}H_{30}O_3+Na]^+$ requires m/z = 485.2093, found m/z = 485.2087 (ESI+).

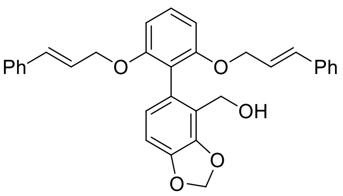

(5-(2,6-Bis(cinnamyloxy)phenyl)benzo[*d*][1,3]dioxol-4-yl)methanol (4ca) was synthesized by following Procedure 11 from **1c**. The crude material was purified by normal-phase column chromatography using an eluent of 9–33% EtOAc/Hx to provide **4ca** (29% yield).

^{1}H NMR (400 MHz, $CDCl_3$) δ 7.29–7.24 (m, 8H), 7.22 (d, *J* = 8.5 Hz, 2H), 7.18 (ddd, *J* = 8.4, 5.8, 2.9 Hz, 2H), 6.83 (d, *J* = 8.0 Hz, 1H), 6.70 (d, *J* = 3.1 Hz, 1H), 6.68 (d, *J* = 3.5 Hz, 1H), 6.44 (d, *J* = 16.0 Hz, 2H), 6.16 (dt, *J* = 16.0, 5.5 Hz, 2H), 6.01 (s, 2H), 4.58 (d, *J* = 5.3 Hz, 4H), 4.34 (d, *J* = 3.8 Hz, 2H).
^{13}C NMR (100 MHz, $CDCl_3$) δ 157.3 (2C), 146.8, 146.6, 136.4 (2C), 132.8 (2C), 129.2, 128.7 (4C), 128.0 (2C), 127.4, 126.6 (4C), 124.4, 124.2 (2C), 122.0, 119.1, 108.0, 107.1 (2C), 101.4, 69.8 (2C), 58.6.
IR (FT-ATR, cm^{-1}, CH_2Cl_2) ν_{max} 3521, 3025, 2883, 1587, 1496, 1449, 1376, 1329, 1245, 1101, 1072, 1043, 999, 965, 932, 861, 809, 780, 731, 691, 631, 609, 587, 561, 536, 497, 478.
HRMS: Exact mass calculated for $[C_{32}H_{28}O_5+Na]^+$ requires *m*/*z* =515.1834, found *m*/*z* = 515.1830 (ESI+).

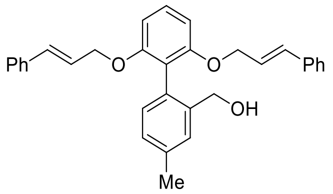

(2',6'-Bis(cinnamyloxy)-4-methyl-[1,1'-biphenyl]-2-yl)methanol (4da) was synthesized by following Procedure 11 from **1d**. The crude material was purified by normal-phase column chromatography using an eluent of 9–33% EtOAc/Hx to provide **4da** (35% yield).

^{1}H NMR (400 MHz, $CDCl_3$) δ 7.38 (s, 1H), 7.31–7.25 (m, 8H), 7.24 (d, *J* = 3.1 Hz, 1H), 7.22 (d, *J* = 2.5 Hz, 1H), 7.20 (dd, *J* = 8.1, 2.9 Hz, 2H), 7.12 (d, *J* = 7.7 Hz, 1H), 6.71 (d, *J* = 8.4 Hz, 2H), 6.41 (d, *J* = 16.0 Hz, 2H), 6.16 (dt, *J* = 16.0, 5.3 Hz, 2H), 4.61 (d, *J* = 4.3 Hz, 4H), 4.35 (s, 2H), 2.43 (s, 3H).
^{13}C NMR (100 MHz, $CDCl_3$) 157.0 (2C), 139.6, 137.4, 136.5, 132.4 (2C), 131.3, 130.5, 130.0, 129.1, 128.6 (4C), 128.5, 127.9 (2C), 126.6 (4C), 126.5 (2C), 124.3 (2C), 106.9 (2C), 69.5 (2C), 64.4, 21.4.
IR (FT-ATR, cm^{-1}, CH_2Cl_2) ν_{max} 3435, 3024, 2920, 2860, 1748, 1589, 1495, 1448, 1375, 1245, 1224, 1180, 1156, 1108, 1071, 1043, 1002, 964, 922, 887, 820, 781, 730, 690, 575, 541, 497, 440.
HRMS: Exact mass calculated for $[C_{32}H_{30}O_3+Na]^+$ requires *m*/*z* = 485.2093, found *m*/*z* = 485.2087 (ESI+).

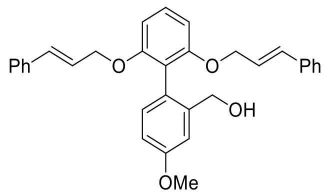

(2',6'-Bis(cinnamyloxy)-4-methoxy-[1,1'-biphenyl]-2-yl)methanol (4ea) was synthesized by following Procedure 11 from **1e**. The crude material was purified by normal-phase column chromatography using an eluent of 9–33% EtOAc/Hx to provide **4ea** (41% yield).

^{1}H NMR (400 MHz, $CDCl_3$) δ 7.29 (d, *J* = 4.2 Hz, 9H), 7.23 (dd, *J* = 8.5, 4.3 Hz, 2H), 7.19–7.14 (m, 2H), 6.95 (d, *J* = 8.4 Hz, 1H), 6.73 (d, *J* = 8.3 Hz, 2H), 6.44 (d, *J* = 16.0 Hz, 2H), 6.19 (dt, *J* = 16.0, 5.2 Hz, 2H), 4.62 (d, *J* = 5.2 Hz, 4H), 4.38 (d, *J* = 4.9 Hz, 2H), 3.88 (s, 3H).
^{13}C NMR (100 MHz, $CDCl_3$) δ 159.2, 157.2 (2C), 141.2, 136.5 (2C), 132.5 (4C), 129.0, 128.7 (4C), 127.9 (2C), 126.6 (4C), 125.5, 124.3 (2C), 119.6, 113.7, 107.0 (2C), 69.6 (2C), 64.4, 55.4.
IR (FT-ATR, cm^{-1}, CH_2Cl_2) ν_{max} 3457, 3025, 2954, 2922, 2854, 1591, 1496, 1450, 1377, 1294, 1264, 1228, 1158, 1108, 1072, 1044, 1001, 965, 858, 817, 782, 732, 692, 562, 496.
HRMS: Exact mass calculated for $[C_{32}H_{30}O_4+Na]^+$ requires *m/z* = 501.2042, found *m/z* = 501.2037 (ESI+).

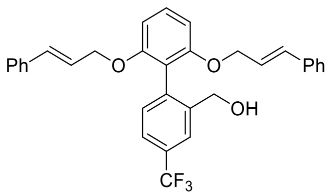

(2',6'-Bis(cinnamyloxy)-4-(trifluoromethyl)-[1,1'-biphenyl]-2-yl)methanol (4fa) was synthesized by following Procedure 11 from **1f**. The crude material was purified by normal-phase column chromatography using an eluent of 9–33% EtOAc/Hx to provide **4fa** (46% yield).

^{1}H NMR (400 MHz, $CDCl_3$) δ 7.89 (s, 1H), 7.63 (d, *J* = 7.8 Hz, 1H), 7.36 (d, *J* = 7.6 Hz, 1H), 7.32 (d, *J* = 8.2 Hz, 1H), 7.26 (dd, *J* = 14.8, 10.8 Hz, 10H), 6.74 (d, *J* = 8.4 Hz, 2H), 6.39 (d, *J* = 16.0 Hz, 2H), 6.17 (dt, *J* = 15.9, 5.2 Hz, 2H), 4.63 (d, *J* = 5.2 Hz, 4H), 4.46 (d, *J* = 6.3 Hz, 2H).
^{13}C NMR (100 MHz, $CDCl_3$) δ 156.6 (2C), 141.0, 137.3 (q, *J* = 1.2 Hz), 136.3, 132.5 (2C), 132.0, 130.0 (q, *J* = 32.3 Hz), 129.9, 128.7 (4C), 128.1 (2C), 126.6 (4C), 125.4 (q, *J* = 3.7 Hz), 124.5 (q, *J* = 271.0 Hz), 124.1 (q, *J* = 3.6 Hz), 123.8 (2C) 118.1, 106.5 (2C), 69.3(2C), 63.5.
IR (FT-ATR, cm^{-1}, CH_2Cl_2) ν_{max} 3442, 3026, 2924, 1618, 1588, 1495, 1464, 1449, 1416, 1376, 1328, 1298, 1246, 1161, 1112, 1076, 1044, 1004, 964, 904, 886, 835, 806, 782, 729, 690, 596, 548, 495.
HRMS: Exact mass calculated for $[C_{32}H_{27}F_3O_3+Na]^+$ requires *m/z* = 539.1810, found *m/z* = 539.1805 (ESI+).

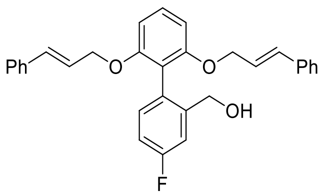

(2',6'-Bis(cinnamyloxy)-4-fluoro-[1,1'-biphenyl]-2-yl)methanol (4ga) was synthesized by following Procedure 11 from **1g**. The crude material was purified by normal-phase column chromatography using an eluent of 9–33% EtOAc/Hx to provide **4ga** (44% yield).

^{1}H NMR (400 MHz, $CDCl_3$) δ 7.3 (t, *J* = 8.8 Hz, 10H), 7.28–7.19 (m, 3H), 7.09 (td, *J* = 8.3, 2.7 Hz, 1H), 6.74 (dd, *J* = 7.7, 2.8 Hz, 2H), 6.45 (dd, *J* = 15.9, 1.5 Hz, 2H), 6.24–6.15 (m, 2H), 4.64 (s, 4H), 4.39 (s, 2H).
^{13}C NMR (100 MHz, $CDCl_3$) δ 162.5 (d, *J* = 245.4 Hz), 156.9 (2C), 142.3 (d, *J* = 7.0 Hz), 136.4 (2C), 132.9 (d, *J* = 8.0 Hz) 132.5 (2C), 129.4, 128.9, 128.7 (4C), 128.0 (2C), 126.6 (4C), 124.1 (2C), 118.5, 115.2 (d, *J* = 21.5 Hz), 114.4 (d, *J* = 21.2 Hz), 106.6 (2C), 69.4 (2C), 63.7.
IR (FT-ATR, cm^{-1}, CH_2Cl_2) ν_{max} 3567, 3056, 3024, 2972, 2926, 2857, 1588, 1495, 1463, 1449, 1377, 1334, 1297, 1248, 1228, 1176, 1111, 1073, 1049, 1003, 966, 858, 806, 782, 758, 730, 692, 605, 565, 495.
HRMS: Exact mass calculated for $[C_{31}H_{27}FO_3+H]^+$ requires *m/z* = 489.1842, found *m/z* = 489.1837 (ESI+).

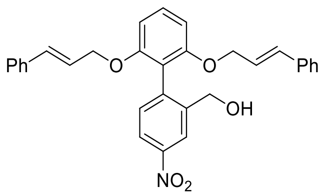

(2',6'-Bis(cinnamyloxy)-4-nitro-[1,1'-biphenyl]-2-yl)methanol (4ha) was synthesized by following Procedure 11 from **1h**. The crude material was purified by normal-phase column chromatography using an eluent of 9–33% EtOAc/Hx to provide **4ha** (44% yield).

^{1}H NMR (400 MHz, $CDCl_3$) δ 8.50 (d, *J* = 2.3 Hz, 1H), 8.20 (dd, *J* = 8.4, 2.4 Hz, 1H), 7.40 (d, *J* = 8.4 Hz, 1H), 7.36 (t, *J* = 8.4 Hz, 1H), 7.31 (d, *J* = 7.6 Hz, 2H), 7.29–7.21 (m, 8H), 6.74 (d, *J* = 8.4 Hz, 2H), 6.45 (d, *J* = 16.0 Hz, 2H), 6.17 (dt, *J* = 16.0, 5.5 Hz, 2H), 4.64 (d, *J* = 4.6 Hz, 4H), 4.50 (s, 2H).
^{13}C NMR (100 MHz, $CDCl_3$) δ 156.4 (2C), 147.7, 142.2, 140.4, 136.1 (2C), 133.0 (2C), 132.6, 130.3, 128.8 (4C), 128.2 (2C), 126.6 (4C), 123.7 (2C), 123.1, 122.1, 117.0, 106.2 (2C), 69.4 (2C), 63.0.
IR (FT-ATR, cm^{-1}, CH_2Cl_2) ν_{max} 3552, 3026, 2923, 2854, 1588, 1518, 1495, 1450, 1376, 1342, 1297, 1264, 1247, 1227, 1184, 1101, 1074, 1043, 1004, 965, 902, 838, 807, 782, 730, 691, 598, 562, 434.
HRMS: Exact mass calculated for $[C_{31}H_{27}NO_5+H]^+$ requires *m/z* = 516.1787, found *m/z* = 516.1781 (ESI+).

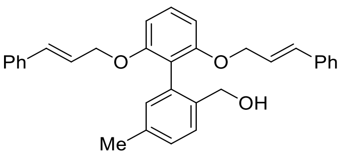

(2',6'-Bis(cinnamyloxy)-5-methyl-[1,1'-biphenyl]-2-yl)methanol (4ia) was synthesized by following Procedure 11 from **1i**. The crude material was purified by normal-phase column chromatography using an eluent of 9–33% EtOAc/Hx to provide **4ia** (46% yield).

^{1}H NMR (400 MHz, $CDCl_3$) δ 7.47 (d, *J* = 7.6 Hz, 1H), 7.28 (m, 10H), 7.23 (m, 2H), 7.07 (s, 1H), 6.73 (d, *J* = 8.3 Hz, 2H), 6.42 (d, *J* = 16.0 Hz, 2H), 6.18 (dt, *J* = 9.5, 4.3 Hz, 2H), 4.63 (d, *J* = 4.2 Hz, 4H), 4.36 (d, *J* = 4.1 Hz, 2H), 2.37 (s, 3H).
^{13}C NMR (100 MHz, $CDCl_3$) δ 156.9 (2C), 137.1, 137.0, 136.5 (2C), 133.5, 132.3 (2C), 132.0, 129.3, 129.1, 128.8, 128.7 (4C), 127.9 (2C), 127.0 (4C), 124.3 (2C), 120.1, 106.9 (2C), 69.4 (2C), 64.2, 21.4.
IR (FT-ATR, cm^{-1}, CH_2Cl_2) ν_{max} 3447, 3024, 2923 2855, 1749, 1586, 1495, 1449, 1376, 1245, 1227, 1179, 1109, 1072, 1047, 1029, 966, 913, 880, 820, 782, 736, 692, 606, 581, 541, 496, 446.
HRMS: Exact mass calculated for $[C_{32}H_{30}O_3+Na]^+$ requires *m/z* = 485.2093, found *m/z* = 485.2087 (ESI+).

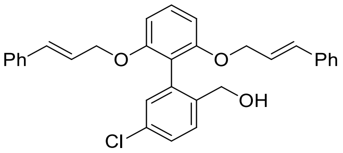

(5-Chloro-2',6'-bis(cinnamyloxy)-[1,1'-biphenyl]-2-yl)methanol (4ja) was synthesized by following Procedure 11 from **1j**. The crude material was purified by normal-phase column chromatography using an eluent of 9–33% EtOAc/Hx to provide **4ja** (25% yield).

^{1}H NMR (400 MHz, $CDCl_3$) δ 7.53 (d, *J* = 8.2 Hz, 1H), 7.40 (d, *J* = 7.9 Hz, 1H), 7.30 (t, *J* = 6.3 Hz, 10H), 7.27–7.22 (m, 2H), 7.09 (s, 1H), 6.73 (d, *J* = 8.3 Hz, 2H), 6.46 (d, *J* = 15.9 Hz, 2H), 6.19 (dt, *J* = 10.9, 5.1 Hz, 2H), 4.64 (d, *J* = 4.2 Hz, 4H), 4.37 (s, 2H).
^{13}C NMR (100 MHz, $CDCl_3$) δ 156.7 (2C), 138.6, 136.4 (2C), 135.2, 133.0, 132.6 (2C), 131.3, 130.3, 129.7, 128.7, 127.98 (4C), 127.95 (2C), 126.6 (4C), 124.0 (2C), 118.2, 106.6 (2C), 69.4 (2C), 63.5.
IR (FT-ATR, cm^{-1}, CH_2Cl_2) ν_{max} 3438, 3026, 2922, 2853, 1725, 1589, 1495, 1449, 1377, 1245, 1227, 1100, 1072, 1046, 1018, 964, 911, 882, 819, 784, 732, 691, 603, 492, 442.
HRMS: Exact mass calculated for $[C_{31}H_{27}ClO_3+Na]^+$ requires *m/z* = 505.1546, found *m/z* = 505.1541 (ESI+).

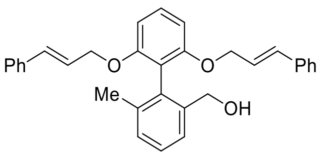

(2',6'-Bis(cinnamyloxy)-6-methyl-[1,1'-biphenyl]-2-yl)methanol (4ka) was synthesized by following Procedure 11 from **1k**. The crude material was purified by normal-phase column chromatography using an eluent of 9–33% EtOAc/Hx to provide **4ka** (37% yield).

^{1}H NMR (400 MHz, $CDCl_3$) δ 7.42 (d, J = 7.5 Hz, 1H), 7.37 (d, J = 7.5 Hz, 1H), 7.33 (d, J = 9.0 Hz, 1H), 7.31–7.25 (m, 9H), 7.25–7.20 (m, 2H), 6.74 (d, J = 8.3 Hz, 2H), 6.39 (d, J = 16.0 Hz, 2H), 6.17 (dt, J = 16.0, 5.1 Hz, 2H), 4.68–4.59 (m, 4H), 4.34 (d, J = 5.7 Hz, 2H), 2.06 (s, 3H).
^{13}C NMR (100 MHz, $CDCl_3$) δ 156.6 (2C), 139.7, 139.3, 137.7, 136.5 (2C), 133.5, 132.1 (2C), 129.34, 129.26, 128.6 (4C), 127.9 (2C), 127.8, 126.6 (4C), 124.3 (2C), 118.3, 106.6 (2C), 69.1 (2C), 64.9, 20.3.
IR (FT-ATR, cm^{-1}, CH_2Cl_2) ν_{max} 3446, 3025, 2922, 2855, 1588, 1495, 1448, 1375, 1296, 1245, 1225, 1179, 1106, 1070, 963, 907, 800, 780, 720, 690, 602, 576, 528, 493.
HRMS: Exact mass calculated for $[C_{32}H_{30}O_3+Na]^+$ requires *m*/*z* = 485.2093, found *m*/*z* = 485.2087 (ESI+).

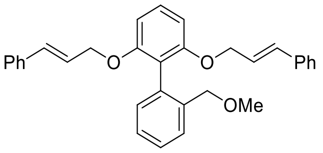

2,6-Bis(cinnamyloxy)-2'-(methoxymethyl)-1,1'-biphenyl (4la) was synthesized by following Procedure 11 from **1l**. The crude material was purified by normal-phase column chromatography using an eluent of 9–33% EtOAc/Hx to provide **4la** (50% yield).

^{1}H NMR (400 MHz, $CDCl_3$) δ 7.61 (d, *J* = 5.0 Hz, 1H), 7.47–7.34 (m, 4H), 7.30 (s, 10H), 6.71 (d, *J* = 7.2 Hz, 2H), 6.43 (d, *J* = 15.9 Hz, 2H), 6.22 (d, *J* = 15.0 Hz, 2H), 4.64 (s, 4H), 4.34 (s, 2H), 3.22 (d, *J* = 1.1 Hz, 3H).
^{13}C NMR (100 MHz, $CDCl_3$) δ 156.9 (2C), 136.70 (2C), 131.69, 131.2 (2C), 129.0, 128.69, 128.64 (4C), 127.8 (2C), 127.42, 127.40, 127.1, 126.9 (4C), 126.5, 124.7 (2C), 118.3, 106.1 (2C), 72.4, 69.0 (2C), 58.0.
IR (FT-ATR, cm^{-1}, CH_2Cl_2) ν_{max} 3057, 3024, 2922, 2855, 2820, 1588, 1495, 1447, 1376, 1333, 1245, 1226, 1198, 1157, 1096, 1046, 1003, 964, 913, 834, 806, 782, 761, 728, 691, 601, 575, 495, 441.
HRMS: Exact mass calculated for $[C_{32}H_{30}O_3+H]^+$ requires *m*/*z* = 463.2273, found *m*/*z* = 463.2268 (ESI+).

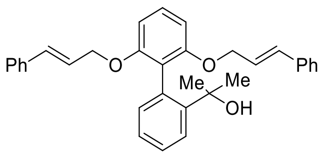

2-(2',6'-Bis(cinnamyloxy)-[1,1'-biphenyl]-2-yl)propan-2-ol (4ma) was synthesized by following Procedure 11 from **1m**. The crude material was purified by normal-phase column chromatography using an eluent of 9–33% EtOAc/Hx to provide **4ma** (30% yield).

^{1}H NMR (400 MHz, $CDCl_3$) δ 7.68 (d, *J* = 7.9 Hz, 1H), 7.41 (t, *J* = 7.6 Hz, 1H), 7.35–7.20 (m, 12H), 7.08 (d, *J* = 7.5 Hz, 1H), 6.69 (d, *J* = 8.3 Hz, 2H), 6.37 (d, *J* = 16.0 Hz, 2H), 6.20 (ddd, *J* = 16.0, 4.9, 4.2 Hz, 2H), 4.71–4.61 (m, 4H), 2.82 (s, 1H), 1.48 (d, *J* = 0.6 Hz, 6H).
^{13}C NMR (100 MHz, $CDCl_3$) δ 156.3 (2C), 147.0, 136.5 (2C), 132.8, 132.3, 131.6 (2C), 128.8, 128.5 (4C), 127.7 (2C), 127.3, 126.7, 126.4 (4C), 125.9, 124.3 (2C), 122.8, 105.8 (2C), 73.4, 68.6 (2C), 31.0 (2C).
IR (FT-ATR, cm^{-1}, CH_2Cl_2) ν_{max} 3567, 3056, 3024, 2972, 2926, 2857, 1588, 1495, 1463, 1449, 1377, 1334, 1297, 1248, 1228, 1111, 1073, 1049, 1003, 966, 858, 806, 782, 758, 730, 692, 605, 565, 495.
HRMS: Exact mass calculated for $[C_{33}H_{32}O_3+Na]^+$ requires *m*/*z* = 499.2249, found *m*/*z* = 499.2244 (ESI+).

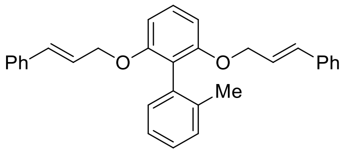

2,6-Bis(cinnamyloxy)-2'-methyl-1,1'-biphenyl (4na) was synthesized by following Procedure 11 from **1n**. The crude material was purified by normal-phase column chromatography using an eluent of 0–14% EtOAc/Hx to provide **4na** (46% yield).

^{1}H NMR (400 MHz, $CDCl_3$) δ 7.31–7.26 (m, 6H), 7.26–7.22 (m, 5H), 7.22–7.16 (m, 4H), 6.68 (d, *J* = 8.3 Hz, 2H), 6.45–6.36 (m, 2H), 6.19 (dt, *J* = 16.0, 5.0 Hz, 2H), 4.62 (d, *J* = 4.8 Hz, 4H), 2.13 (s, 3H).
^{13}C NMR (100 MHz, $CDCl_3$) δ 157.0 (2C), 137.5, 136.8, 134.5, 131.6 (2C), 131.1, 129.6, 128.7 (2C), 128.6 (4C), 127.7 (2C), 127.2, 126.6 (4C), 125.3, 124.9 (2C), 120.8, 106.4 (2C), 69.1 (2C), 20.1.
IR (FT-ATR, cm^{-1}, CH_2Cl_2) ν_{max} 1585, 1458, 1385, 1250, 1111, 968, 733, 694, 455, 432.
HRMS: Exact mass calculated for $[C_{31}H_{28}O_2]$ requires *m*/*z* = 432.2089, found *m*/*z* = 432.2087 (EI).

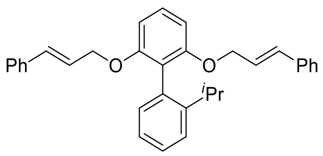

2,6-Bis(cinnamyloxy)-2'-isopropyl-1,1'-biphenyl (4oa) was synthesized by following Procedure 11 from **1o**. The crude material was purified by normal-phase column chromatography using an eluent of 0–10% EtOAc/Hx to provide **4oa** (43% yield).

^{1}H NMR (400 MHz, $CDCl_3$) δ 7.43–7.35 (m, 2H), 7.30–7.27 (m, 2H), 7.25–7.17 (m, 4H), 7.14 (dd, *J* = 7.5, 0.9 Hz, 1H), 6.68 (d, *J* = 8.3 Hz, 2H), 6.39 (dd, *J* = 16.0, 1.5 Hz, 2H), 6.19 (dt, *J* = 16.0, 4.9 Hz, 2H), 4.69–4.58 (m, 4H), 2.76 (hept, *J* = 6.8 Hz, 1H), 1.12 (d, *J* = 6.9 Hz, 6H).
^{13}C NMR (100 MHz, $CDCl_3$) δ 157.2 (2C), 148.1, 136.8 (2C), 133.4, 131.6 (2C), 130.9, 128.6 (4C), 128.5, 127.7 (2C), 127.6, 126.5 (4C), 125.3, 124.9, 124.8 (2C), 120.6, 105.8 (2C), 68.7 (2C), 30.6, 24.0 (2C).
IR (FT-ATR, cm^{-1}, CH_2Cl_2) ν_{max} 3101, 3081, 3058, 3023, 2958, 2925, 2865, 1589, 1496, 1465, 1448, 1377, 1335, 1298, 1247, 1227, 1204, 1179, 1112, 1083, 1072, 1049, 1036, 1004, 965, 783, 757, 728, 690.
HRMS: Exact mass calculated for [$C_{33}H_{32}O_2$] requires *m*/*z* = 460.2402, found *m*/*z* = 460.2400 (EI).

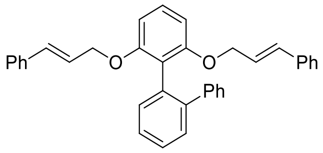

2,6-Bis(cinnamyloxy)-1,1':2',1''-terphenyl (4pa) was synthesized by following Procedure 11 from **1p**. The crude material was purified by normal-phase column chromatography using an eluent of 0–9% EtOAc/Hx to provide **4pa** (46% yield).

^{1}H NMR (400 MHz, $CDCl_3$) δ 7.44–7.40 (m, 2H), 7.40–7.38 (m, 1H), 7.36 (ddd, *J* = 8.5, 3.9, 2.0 Hz, 2H), 7.30–7.27 (m, 3H), 7.25 (s, 1H), 7.20 (ddd, *J* = 5.4, 4.8, 2.6 Hz, 4H), 7.18–7.13 (m, 3H), 7.10 (dd, *J* = 6.1, 4.9 Hz, 2H), 7.08 (d, *J* = 3.5 Hz, 2H), 6.47 (d, *J* = 8.4 Hz, 2H), 6.38 (dt, *J* = 16.0, 1.5 Hz, 2H), 6.08 (dt, *J* = 16.0, 5.1 Hz, 2H), 4.45 (dddd, *J* = 57.6, 13.7, 5.1, 1.6 Hz, 4H).
^{13}C NMR (100 MHz, $CDCl_3$) δ 156.9 (2C), 142.5, 142.4, 136.8 (2C), 133.2, 132.0, 131.6 (2C), 129.5, 129.0 (2C), 128.7 (5C), 127.7 (2C), 127.5, 127.4 (2C), 126.9, 126.6 (4C), 126.4, 125.1 (2C), 120.4, 105.5 (2C), 68.7 (2C).
IR (FT-ATR, cm^{-1}, CH_2Cl_2) ν_{max} 3101, 3080, 3057, 3023, 2923, 2854, 1739, 1589, 1496, 1466, 1449, 1431, 1376, 1299, 1247, 1227, 1113, 1081, 1073, 1050, 1030, 1005, 966, 781, 760, 741, 698, 691.
HRMS: Exact mass calculated for [$C_{36}H_{30}O_2$] requires *m*/*z* = 494.2246, found *m*/*z* = 494.2243 (EI).

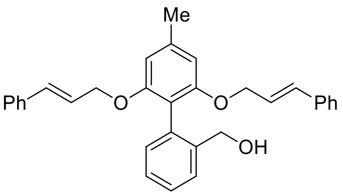

(2',6'-Bis(cinnamyloxy)-4'-methyl-[1,1'-biphenyl]-2-yl)methanol (4qa) was synthesized by following Procedure 11 from **1q**. The crude material was purified by normal-phase column chromatography using an eluent of 9–33% EtOAc/Hx to provide **4qa** (51% yield).

^{1}H NMR (400 MHz, $CDCl_3$) δ 7.60 (d, *J* = 7.4 Hz, 1H), 7.42 (dt, *J* = 14.9, 7.3 Hz, 2H), 7.33–7.22 (m, 12H), 6.58 (s, 2H), 6.42 (d, *J* = 16.0 Hz, 2H), 6.18 (dt, *J* = 16.0, 5.0 Hz, 2H), 4.62 (d, *J* = 4.5 Hz, 4H), 4.43 (s, 2H), 2.43 (s, 3H).
^{13}C NMR (100 MHz, $CDCl_3$) δ 156.6 (2C), 140.0, 139.5, 136.5 (2C), 133.7, 132.2 (2C), 131.6, 129.2, 128.6 (4C), 127.9 (2C), 127.8, 127.6, 126.6 (4C), 124.3 (2C), 117.0, 107.7 (2C), 69.4 (2C), 64.4, 22.3.
IR (FT-ATR, cm^{-1}, CH_2Cl_2) ν_{max} 3445, 3025, 2922, 2858, 1723, 1609, 1577, 1495, 1448, 1415, 1472, 1317, 1265, 1233, 1117, 1073, 1004, 964, 813, 732, 691, 607, 588, 496, 439.
HRMS: Exact mass calculated for $[C_{32}H_{30}O_3+Na]^+$ requires *m*/*z* = 485.2093, found *m*/*z* = 485.2087 (ESI+).

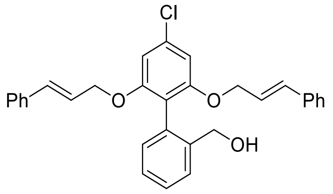

(4'-Chloro-2',6'-bis(cinnamyloxy)-[1,1'-biphenyl]-2-yl)methanol (4ra) was synthesized by following Procedure 11 from **1r**. The crude material was purified by normal-phase column chromatography using an eluent of 9–33% EtOAc/Hx to provide **4ra** (46% yield).

^{1}H NMR (400 MHz, $CDCl_3$) δ 7.60 (d, *J* = 7.5 Hz, 1H), 7.31 (dt, *J* = 21.4, 7.3 Hz, 3H), 7.33–7.25 (m, 8H), 7.25–7.20 (m, 2H), 6.74 (s, 2H), 6.42 (d, *J* = 16.0 Hz, 2H), 6.16 (dt, *J* = 16.0, 5.2 Hz, 2H), 4.61 (d, *J* = 5.1 Hz, 4H), 4.41 (s, 2H).
^{13}C NMR (100 MHz, $CDCl_3$) δ 157.1 (2C), 139.9, 136.3 (2C), 134.6, 132.7 (2C), 132.5, 131.3, 128.9, 128.7 (4C), 128.2, 128.0 (2C), 127.6, 126.6 (4C), 123.5 (2C), 118.1, 107.4 (2C), 69.5 (2C), 64.1.
IR (FT-ATR, cm^{-1}, CH_2Cl_2) ν_{max} 3025, 2922, 2853, 1579, 1495, 1448, 1415, 1376, 1300, 1264, 1211, 1115, 1029, 1003, 964, 890, 873, 844, 814, 768, 733, 690, 606, 589, 559, 494, 440.
HRMS: Exact mass calculated for $[C_{31}H_{27}ClO_3+Na]^+$ requires *m*/*z* = 505.1541, found *m*/*z* = 505.1546 (ESI+).

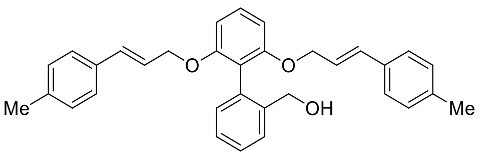

(2',6'-Bis(((*E*)-3-(p-tolyl)allyl)oxy)-[1,1'-biphenyl]-2-yl)methanol (4ab) was synthesized by following Procedure 11 from **2b**. The crude material was purified by normal-phase column chromatography using an eluent of 9–33% EtOAc/Hx to provide **4ab** (32% yield).

^{1}H NMR (400 MHz, $CDCl_3$) δ 7.59 (d, *J* = 7.3 Hz, 1H), 7.46–7.36 (m, 2H), 7.29 (t, *J* = 8.4 Hz, 1H), 7.25 (d, *J* = 6.3 Hz, 1H), 7.18 (d, *J* = 7.9 Hz, 4H), 7.10 (d, *J* = 7.8 Hz, 4H), 6.73 (d, *J* = 8.3 Hz, 2H), 6.39 (d, *J* = 15.9 Hz, 2H), 6.12 (dt, *J* = 15.9, 5.3 Hz, 2H), 4.61 (d, *J* = 5.1 Hz, 4H), 4.40 (s, 2H), 2.33 (s, 6H).
^{13}C NMR (100 MHz, $CDCl_3$) δ 156.9 (2C), 139.9, 137.7 (2C), 133.7, 133.6, 132.4 (2C), 131.4, 129.3 (4C), 129.2, 129.1, 127.9 (2C), 127.6, 126.5 (4C), 123.2 (2C), 119.9, 106.9 (2C), 69.6 (2C), 64.3, 21.3 (2C).
IR (FT-ATR, cm^{-1}, CH_2Cl_2) ν_{max} 3437, 3021, 2953, 2920, 2855, 1744, 1655, 1588, 1512, 1451, 1411, 1377, 1326, 1297, 1245, 1227, 1181, 1103, 1077, 1044, 1018, 1005, 968, 833, 780, 760, 729, 608, 562, 506.
HRMS: Exact mass calculated for $[C_{33}H_{32}O_3+Na]^+$ requires *m/z* = 499.2249, found *m/z* = 499.2243 (ESI+).

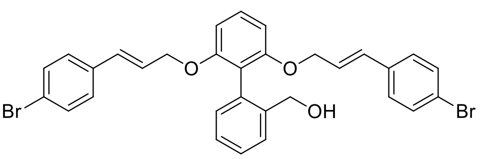

(2',6'-Bis(((*E*)-3-(4-bromophenyl)allyl)oxy)-[1,1'-biphenyl]-2-yl)methanol (4ac) was synthesized by following Procedure 11 from **2c**. The crude material was purified by normal-phase column chromatography using an eluent of 9–33% EtOAc/Hx to provide **4ac** (33% yield).

^{1}H NMR (400 MHz, $CDCl_3$) δ 7.60 (d, *J* = 7.3 Hz, 1H), 7.45 (dd, *J* = 8.1, 7.0 Hz, 1H), 7.40 (d, *J* = 8.3 Hz, 4H), 7.31 (td, *J* = 8.2, 2.0 Hz, 1H), 7.25 (s, 1H), 7.11 (d, *J* = 8.3 Hz, 4H), 6.73 (dd, *J* = 8.4, 1.4 Hz, 2H), 6.32 (d, *J* = 16.0 Hz, 2H), 6.18 (d, *J* = 2.7 Hz, 2H), 4.60 (d, *J* = 3.3 Hz, 4H), 4.41 (s, 2H), 2.33 (s, 1H).
^{13}C NMR (100 MHz, $CDCl_3$) δ 156.8 (2C), 139.9, 135.4 (2C), 133.5, 131.7 (4C), 131.4, 131.0 (2C), 129.3, 129.1, 128.1 (4C), 127.9, 127.6, 125.0 (2C), 121.7 (2C), 119.7, 106.8 (2C), 69.1 (2C), 64.2.
IR (FT-ATR, cm^{-1}, CH_2Cl_2) ν_{max} 3431, 3024, 2922, 2854, 1589, 1487, 1462, 1401, 1376, 1324, 1245, 1228, 1204, 1108, 1093, 1071, 1008, 967, 944, 841, 779, 761, 731, 699, 656, 610, 562, 500.
HRMS: Exact mass calculated for $[C_{31}H_{26}Br_2O_3+Na]^+$ requires *m/z* = 627.0146, found *m/z* = 627.0140 (ESI+).

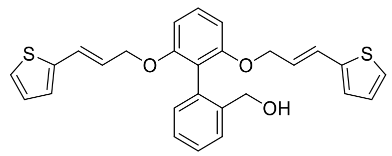

(2',6'-Bis(((*E*)-3-(thiophen-2-yl)allyl)oxy)-[1,1'-biphenyl]-2-yl)methanol (4ad) was synthesized by following Procedure 11 from **2d**. The crude material was purified by normal-phase column chromatography using an eluent of 9–33% EtOAc/Hx to provide **4ad** (23% yield).

^{1}H NMR (400 MHz, $CDCl_3$) δ 7.62–7.58 (m, 1H), 7.48–7.36 (m, 2H), 7.30 (t, *J* = 8.3 Hz, 1H), 7.27–7.21 (m, 1H), 7.13 (d, *J* = 5.0 Hz, 2H), 6.93 (dd, *J* = 5.0, 3.6 Hz, 2H), 6.85 (d, *J* = 3.4 Hz, 2H), 6.72 (d, *J* = 8.4 Hz, 2H), 6.50 (d, *J* = 15.8 Hz, 2H), 6.00 (dt, *J* = 15.7, 5.3 Hz, 2H), 4.58 (dd, *J* = 5.2, 1.3 Hz, 4H), 4.40 (s, 2H).
^{13}C NMR (100 MHz, $CDCl_3$) δ 156.8 (2C), 141.6 (2C), 139.9, 133.5, 131.4, 129.3, 129.1, 128.0, 127.6, 127.5 (2C), 126.2 (2C), 125.5 (2C), 124.7 (2C), 123.7 (2C), 120.0, 107.0 (2C), 69.1 (2C), 64.3.
IR (FT-ATR, cm^{-1}, CH_2Cl_2) ν_{max} 3441, 3066, 3021, 2862, 1648, 1588, 1461, 1450, 1376, 1245, 1228, 1202, 1104, 1080, 1040, 1004, 954, 854, 834, 781, 760, 730, 696, 612, 572, 492.
HRMS: Exact mass calculated for $[C_{27}H_{24}O_3S_2+Na]^+$ requires *m/z* = 483.1065, found *m/z* = 483.1059 (ESI+).

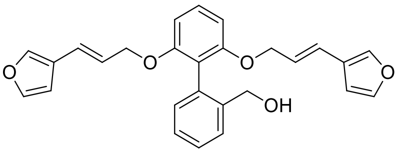

(2',6'-Bis(((*E*)-3-(furan-3-yl)allyl)oxy)-[1,1'-biphenyl]-2-yl)methanol (4ae) was synthesized by following Procedure 11 using 5 mol% of $Pd(dba)_2$ 5 mol% and 20 mol% of **L4** from **2e**. The crude material was purified by normal-phase column chromatography using an eluent of 9–33% EtOAc/Hx to provide **4ae** (26% yield).

^{1}H NMR (400 MHz, $CDCl_3$) δ 7.58 (d, *J* = 7.2 Hz, 1H), 7.44–7.35 (m, 2H), 7.32 (s, 4H), 7.28 (d, *J* = 8.3 Hz, 1H), 7.22 (d, *J* = 7.2 Hz, 1H), 7.09 (s, 1H), 6.72 (d, *J* = 8.3 Hz, 2H), 6.44 (s, 2H), 6.26 (d, *J* = 15.7 Hz, 2H), 5.89 (d, *J* = 15.8 Hz, 2H), 4.55 (s, 4H), 4.38 (s, 2H).
^{13}C NMR (100 MHz, $CDCl_3$) δ 156.9 (2C), 143.7 (2C), 140.9 (2C), 139.9, 133.6, 131.4, 129.21, 129.2, 127.8, 127.5, 123.8 (2C), 123.5 (2C), 122.4 (2C), 119.9, 107.6 (2C), 106.9 (2C), 69.5 (2C), 64.3.
IR (FT-ATR, cm^{-1}, CH_2Cl_2) ν_{max} 3447, 3124, 3023, 2922, 2854, 1664, 1588, 1508, 1452, 1371, 1246, 1228, 1158, 1104, 1072, 1021, 1004, 961, 870, 761, 726, 699, 634, 617, 594, 563, 465, 438.
HRMS: Exact mass calculated for $[C_{27}H_{24}O_5+Na]^+$ requires *m/z* = 451.1521, found *m/z* = 451.1516 (ESI+).

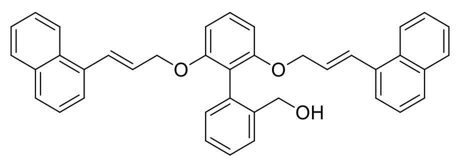

(2',6'-Bis(((*E*)-3-(naphthalen-1-yl)allyl)oxy)-[1,1'-biphenyl]-2-yl)methanol (4af) was synthesized by following Procedure 11 from **2f**. The crude material was purified by normal-phase column chromatography using an eluent of 9–33% EtOAc/Hx to provide **4af** (24% yield).

^{1}H NMR (400 MHz, $CDCl_3$) δ 7.82 (d, *J* = 8.5 Hz, 2H), 7.78 (d, *J* = 7.7 Hz, 2H), 7.75 (d, *J* = 8.5 Hz, 2H), 7.65 (d, *J* = 7.4 Hz, 1H), 7.53–7.44 (m, 7H), 7.41 (dd, *J* = 14.0, 6.1 Hz, 3H), 7.38–7.34 (m, 2H), 7.16 (d, *J* = 15.5 Hz, 2H), 6.81 (d, *J* = 8.3 Hz, 2H), 6.24 (dt, *J* = 8.8, 4.6 Hz, 2H), 4.77–4.73 (m, 4H), 4.48 (d, *J* = 6.2 Hz, 2H).
^{13}C NMR (100 MHz, $CDCl_3$) δ 156.8 (2C), 140.0, 134.3 (2C), 133.68 (2C), 133.65 (2C), 131.4, 131.2, 129.4, 129.2, 128.8 (2C), 128.5 (2C), 128.2 (2C), 128.0, 127.8, 127.1 (2C), 126.1 (2C), 125.9 (2C), 125.6 (2C), 124.0 (2C), 123.7 (2C), 119.6, 106.5 (2C), 69.0 (2C), 64.4.
IR (FT-ATR, cm^{-1}, CH_2Cl_2) ν_{max} 3483, 3044, 2923, 2853, 1715, 1590, 1509, 1467, 1445, 1380, 1348, 1293, 1244, 1200, 1179, 1115, 1043, 1017, 1004, 970, 862, 682, 732, 670, 649, 612, 549, 525, 479, 421.
HRMS: Exact mass calculated for $[C_{39}H_{32}O_3+Na]^+$ requires *m/z* = 571.2249, found *m/z* = 571.2244 (ESI+).

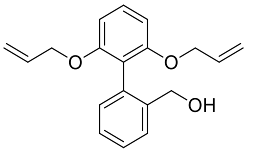

(2',6'-Bis(allyloxy)-[1,1'-biphenyl]-2-yl)methanol (4ag) was synthesized by following Procedure 11 from **2g**. The crude material was purified by normal-phase column chromatography using an eluent of 9–33% EtOAc/Hx to provide **4ag** (27% yield).

^{1}H NMR (400 MHz, $CDCl_3$) δ 7.55 (d, *J* = 7.0 Hz, 1H), 7.37 (tt, *J* = 13.4, 6.8 Hz, 2H), 7.27 (t, *J* = 8.3 Hz, 1H), 7.19 (d, *J* = 6.9 Hz, 1H), 6.66 (d, *J* = 8.4 Hz, 2H), 5.83 (ddt, *J* = 15.3, 9.9, 4.8 Hz, 2H), 5.13 (s, 2H), 5.11–5.07 (m, 2H), 4.45 (d, *J* = 4.6 Hz, 4H), 4.37 (d, *J* = 4.0 Hz, 2H).
^{13}C NMR (100 MHz, $CDCl_3$) δ 156.8 (2C), 139.8, 133.4, 133.0 (2C), 131.3, 129.07, 129.06, 127.9, 127.5, 119.4, 117.2 (2C), 106.4 (2C), 69.4 (2C), 64.3.
IR (FT-ATR, cm^{-1}, CH_2Cl_2) ν_{max} 2923, 1589, 1449, 1228, 1119, 1069, 993, 924, 782, 759, 728.
HRMS: Exact mass calculated for $[C_{19}H_{20}O_3+Na]^+$ requires *m/z* = 319.1310, found *m/z* = 319.1304(ESI+).

6 Kinetic Resolution Study

6.1 Synthesis of Substrates 3ag and 3s

6.1.1. Synthesis of 3ag

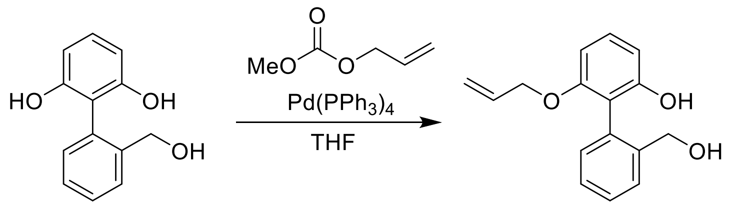

1a (±)-3ag

To a round-bottom flask equipped with a magnetic stirring bar, **1a** (1.0 equiv), methyl allylcarbonate (1.0 equiv) and $Pd(PPh_3)_4$ (0.05 equiv) were added and subsequently dissolved in THF (0.2 M). The reaction was allowed to stir at rt for 1 h. The crude material was purified by normal-phase column chromatography using an eluent of 33% EtOAc/Hx to provide **3ag** (112.1 mg, 22%).

6.1.2. Synthesis of 3s

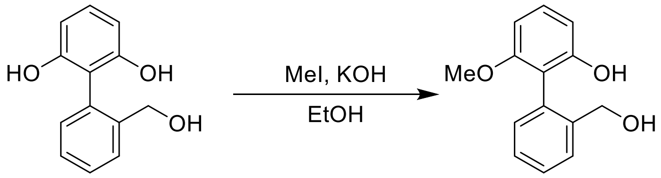

1a (±)-3s

To a round-bottom flask equipped with a magnetic stirring bar, **1a** (1.0 equiv), iodomethane (1.0 equiv) and KOH (1.0 equiv) were added and subsequently dissolved in EtOH (0.1 M). The reaction was heated to 90 °C and allowed to stir for 3 h. After the completion of the reaction, evaporate the EtOH under reduced pressure. Extract the crude mixture with EtOAc and dried with anhydrous $MgSO_4$, filtered and concentrated *in vacuo*. The crude material was purified by normal-phase column chromatography using an eluent of 33% EtOAc/Hx to provide **3s** (139.7 mg, 33%).

^{1}H NMR (400 MHz, $CDCl_3$) δ 7.62–7.57 (m, 1H), 7.49–7.38 (m, 2H), 7.25 (d, J = 6.2 Hz, 1H), 7.24–7.20 (m, 1H), 6.66 (d, J = 8.2 Hz, 1H), 6.57 (d, J = 8.3 Hz, 1H), 4.39 (q, J = 12.1 Hz, 2H), 3.70 (s, 3H).
^{13}C NMR (100 MHz, $CDCl_3$) δ 157.5, 154.1, 140.9, 131.4, 131.3, 129.8, 129.7, 129.2, 128.8, 115.9, 109.2, 103.5, 63.9, 56.1.
IR (FT-ATR, cm^{-1}, CH_2Cl_2) ν_{max} 3696, 3669, 3649, 3640, 3628, 3607, 3588, 3573, 3546, 3469, 2374, 2357, 2342, 2291, 1803, 1783, 1727, 1710, 1695, 1663, 1587, 1568, 1551, 1532, 1516, 1499, 1473, 1446, 1427, 1396, 1037, 1256, 1206, 1159, 1139, 1113, 1085, 1040, 1027, 931, 899, 876, 849, 836, 818, 793, 778, 761, 751, 737, 719, 704, 690, 673, 657, 645, 632.
HRMS: Exact mass calculated for [$C_{14}H_{14}O_3$] requires m/z = 230.0943, found m/z = 230.0938 (EI).

6.2 Kinetic Resolution of 3ag

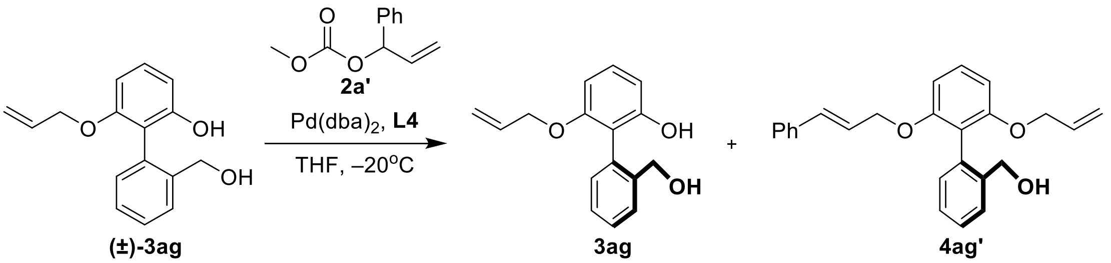

To an oven dried reaction tube equipped with a magnetic stirring bar was premixed $Pd(dba)_2$ (0.6 mg, 0.001 mmol, 0.01 equiv), **L4** (2.2 mg, 0.004 mmol, 0.04 equiv) with THF (0.2 ml). After 10 min, **2** (0.05 mmol, 0.5 equiv) was added with THF (0.3 ml) and stirring was continued for 10 min. Then, **(±)-3ag** (0.10 mmol, 1 equiv) was added and the reaction mixture was left to stir for 8 h at –20 °C. The vial was sealed with a Teflon cap and further secured with Parafilm M®. After that, the crude material was purified by flash column chromatography using an eluent of 9–33% EtOAc/Hx to provide **4ag′**. The enantioselectivity of the remaining **3ag** and the product **4ag′** was determined by chiral HPLC.

6-(Allyloxy)-2'-(hydroxymethyl)-[1,1'-biphenyl]-2-ol (3ag)

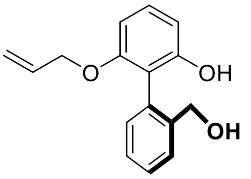

Optical: $[\alpha]^{20}_{D}$ = +20.5° (c = 0.99, $CHCl_3$, 62:38 er)
HPLC (Chiralpak AD-H, 20% ^{i}PrOH/Hx eluent, 1 mL/min, 254 nm): major enantiomer t_R = 6.2 min, minor enantiomer t_R = 8.0 min.

(2'-(Allyloxy)-6'-(cinnamyloxy)-[1,1'-biphenyl]-2-yl)methanol (4ag′)

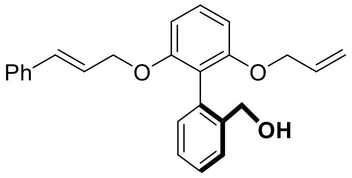

^{1}H NMR (400 MHz, $CDCl_3$) δ 7.56 (dd, J = 11.8, 4.6 Hz, 1H), 7.43–7.31 (m, 2H), 7.31–7.26 (m, 4H), 7.26–7.16 (m, 3H), 6.71 (d, J = 8.3 Hz, 1H), 6.65 (dd, J = 8.3, 5.3 Hz, 1H), 6.39 (d, J = 16.0 Hz, 1H), 6.16 (dt, J = 16.0, 5.2 Hz, 1H), 5.90–5.77 (m, 1H), 5.16–5.10 (m, 1H), 5.10–5.05 (m, 1H), 4.61 (dd, J = 5.2, 1.4 Hz, 2H), 4.49–4.42 (m, 2H), 4.36 (d, J = 7.7 Hz, 2H).
^{13}C NMR (100 MHz, $CDCl_3$) δ 156.9, 156.8, 139.9, 136.5, 133.5, 133.0, 132.3, 131.4, 129.2, 129.11, 129.08, 128.7, 127.9, 127.6, 126.6, 124.3, 119.7, 117.24, 117.19, 106.8, 106.5, 106.4, 69.44, 69.41, 64.3.
IR (FT-ATR, cm^{-1}, CH_2Cl_2) ν_{max} 3696, 3689, 3669, 3649, 3639, 3607, 3574, 2386, 2376, 2358, 2322, 1768, 1744, 1728, 1711, 1695, 1663, 1647, 1569, 1551, 1517, 1457.
HRMS: Exact mass calculated for [$C_{25}H_{24}O_3$] requires m/z = 372.1725, found m/z = 372.1725 (EI).
Optical: $[\alpha]^{20}_{D}$ = +20.4° (c = 1.02, $CHCl_3$, 76:24 er)

HPLC (Chiralpak AD-H, 20% ^{i}PrOH/Hx eluent, 1 mL/min, 254 nm): major enantiomer t_R = 9.0 min, minor enantiomer t_R = 10.0 min.

6.3 Kinetic Resolution of 3s

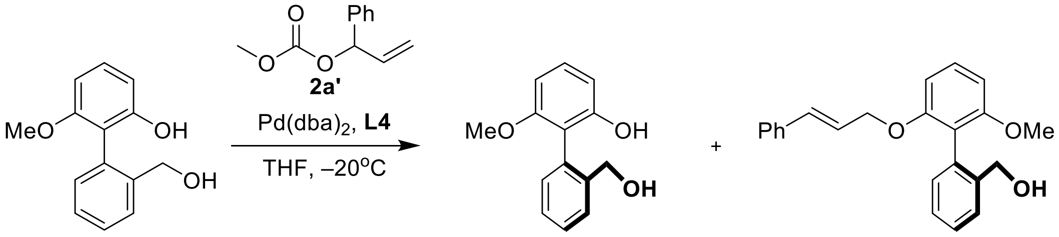

(±)-3s **3s** **4sa**

To an oven dried reaction tube equipped with a magnetic stirring bar was premixed Pd(dba)$_2$ (0.6 mg, 0.001 mmol, 0.01 equiv), **L4** (2.2 mg, 0.004 mmol, 0.04 equiv) with THF (0.2 ml). After 10 min, **2** (0.05 mmol, 0.5 equiv) was added with THF (0.3 ml) and stirring was continued for 10 min. Then, **(±)-3s** (0.10 mmol, 1 equiv) was added and the reaction mixture was left to stir for 8 h at –20 °C. The vial was sealed with a Teflon cap and further secured with Parafilm M®. After that, the crude material was purified by flash column chromatography using an eluent of 9–33% EtOAc/Hx to provide **4sa**. The enantioselectivity of the remaining **3s** and the product **4sa** was determined by chiral HPLC.

2'-(Hydroxymethyl)-6-methoxy-[1,1'-biphenyl]-2-ol (3s)

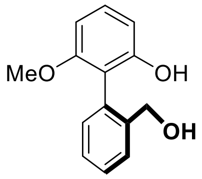

Optical: $[\alpha]^{20}_{D}$ = +37.1° (c = 0.84, $CHCl_3$, 63:37 er)
HPLC (Chiralpak AD-H, 20% ^{i}PrOH/Hx eluent, 1 mL/min, 254 nm): major enantiomer t_R = 6.6 min, minor enantiomer t_R = 8.3 min.

(2'-(Cinnamyloxy)-6'-methoxy-[1,1'-biphenyl]-2-yl)methanol (4sa)

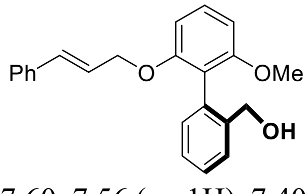

^{1}H NMR (400 MHz, $CDCl_3$) δ 7.60–7.56 (m, 1H), 7.40 (dtd, J = 16.3, 7.3, 1.4 Hz, 2H), 7.33 (d, J = 8.4 Hz, 1H), 7.29 (dd, J = 8.7, 5.6 Hz, 4H), 7.26–7.19 (m, 2H), 6.72 (d, J = 8.3 Hz, 1H), 6.69 (d, J = 8.4 Hz, 1H), 6.39 (d, J = 16.0 Hz, 1H), 6.16 (dt, J = 16.0, 5.2 Hz, 1H), 4.61 (d, J = 5.1 Hz, 2H), 4.38 (d, J = 6.0 Hz, 2H), 3.73 (s, 3H).
^{13}C NMR (100 MHz, $CDCl_3$) δ 157.9, 156.8, 139.9, 136.5, 133.5, 132.3, 131.4, 129.3, 129.1, 128.7 (2C), 128.0, 127.9, 127.7, 126.6 (2C), 124.3, 119.0, 106.6, 104.9, 69.4, 64.3, 56.1.
IR (FT-ATR, cm^{-1}, CH_2Cl_2) ν_{max} 3685, 3664, 3644, 3601, 2367, 2347, 2332, 1723, 1596, 1564, 1476, 1441, 1423, 1388, 1253, 1096, 1004, 857, 834, 813, 768, 742, 724, 691, 669, 656, 639, 619, 607.
HRMS: Exact mass calculated for [$C_{23}H_{22}O_3$] requires m/z = 346.1569, found m/z = 346.1568 (EI).
Optical: $[\alpha]^{20}_{D}$ = +48.9° (c = 1.30, $CHCl_3$, 82:18 er)

HPLC (Chiralpak AD-H, 5% ^{i}PrOH/Hx eluent, 1 mL/min, 254 nm): major enantiomer t_R = 51.3 min, minor enantiomer t_R = 56.8 min.

7 Further Transformations

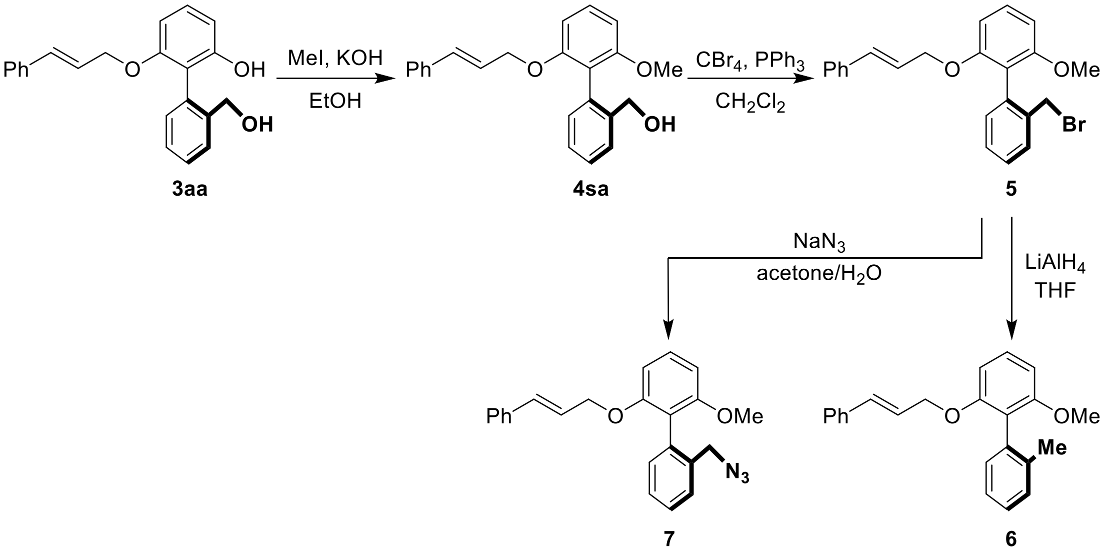

(2'-(Cinnamyloxy)-6'-methoxy-[1,1'-biphenyl]-2-yl)methanol (4sa)

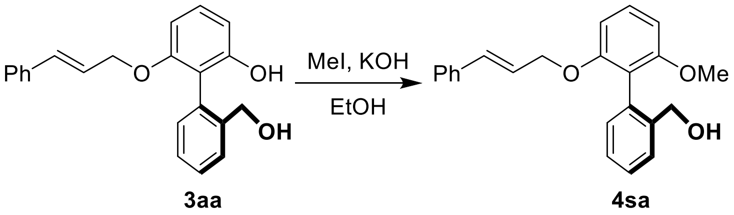

To a round-bottom flask equipped with a magnetic stirring bar, **3aa** (1.0 equiv), iodomethane (1.0 equiv) and KOH (1.0 equiv) were added and subsequently dissolved in EtOH (0.1 M). The reaction was allowed to stir at rt for overnight. After the completion of the reaction, evaporate the EtOH under reduced pressure. Extract the crude mixture with EtOAc and dried with anhydrous $MgSO_4$, filtered and concentrated *in vacuo*. The crude material was purified by normal-phase column chromatography using an eluent of 20% EtOAc/Hx to provide **4sa** (24.0 mg, 73%).

Optical: $[\alpha]^{20}_{D}$ = +26.7° (c = 0.63, $CHCl_3$, 96:4 er)
HPLC (Chiralpak AD-H, 5% ^{i}PrOH/Hx eluent, 1 mL/min, 254 nm): major enantiomer t_R = 51.9 min, minor enantiomer t_R = 57.5 min.

2'-(Bromomethyl)-2-(cinnamyloxy)-6-methoxy-1,1'-biphenyl (5)

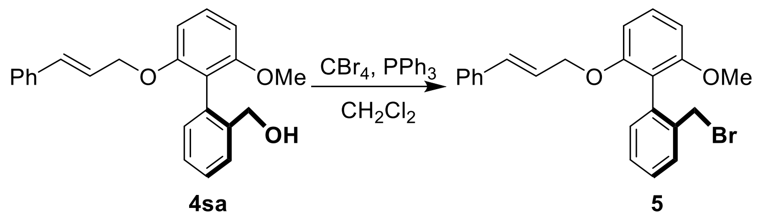

To a round-bottom flask equipped with a magnetic stirring bar, **4sa** (1.0 equiv) in CH_2Cl_2 was added PPh_3 (1.24 equiv) and CBr_4 (1.6 equiv). The reaction mixture was stirred at rt for 2h. After the completion of the reaction, the reaction mixture was quenched with H_2O, extract with CH_2Cl_2, dried with anhydrous $MgSO_4$, filtered and concentrated in vacuo. The crude material was purified by normal-phase column chromatography using an eluent of 20% EtOAc/Hx to provide **5** (25.0 mg, 94%).

^{1}H NMR (400 MHz, $CDCl_3$) δ 7.58–7.54 (m, 1H), 7.40–7.34 (m, 2H), 7.32 (d, J = 8.4 Hz, 1H), 7.28 (d, J = 6.2 Hz, 1H), 7.27–7.26 (m, 2H), 7.26–7.24 (m, 2H), 7.20 (qd, J = 5.4, 2.6 Hz, 2H), 6.71–6.63 (m, 2H), 6.39 (d, J = 16.0 Hz, 1H), 6.19 (dt, J = 16.0, 5.0 Hz, 1H), 4.62 (d, J = 4.3 Hz, 2H), 4.34 (s, 2H), 3.72 (s, 3H).
^{13}C NMR (100 MHz, $CDCl_3$) δ 158.0, 156.9, 136.8, 136.7, 134.7, 131.68, 131.66, 130.2, 129.5, 128.7 (2C), 128.3, 127.9, 127.8, 126.6 (2C), 124.7, 117.9, 105.9, 104.4, 69.0, 56.0, 32.6.
IR (FT-ATR, cm^{-1}, CH_2Cl_2) ν_{max} 3696, 3670, 3649, 3639, 3616, 3607, 3587, 2387, 2375, 2357, 2346, 1803, 1783, 1729, 1695, 1664, 1647, 1596, 1569, 1551, 1517, 1474, 1467, 1456, 1419, 1405, 1388, 1348, 1253, 1206, 1183, 1107, 1064, 1023, 967, 896, 861, 826, 813, 800, 785, 765, 740, 722, 704, 686, 677, 651, 641, 625, 602.
HRMS: Exact mass calculated for [$C_{23}H_{21}BrO_2$] requires m/z = 408.0725, found m/z = 408.0724 (EI).
Optical: $[\alpha]^{20}_D$ = +19.0° (c = 0.92, $CHCl_3$, 95:5 er)
HPLC (Chiralpak OD-H, 5% ^{i}PrOH/Hx eluent, 1 mL/min, 254 nm): major enantiomer t_R = 6.9 min, minor enantiomer t_R = 7.8 min.

2-(Cinnamyloxy)-2'-ethyl-6-methoxy-1,1'-biphenyl (6)

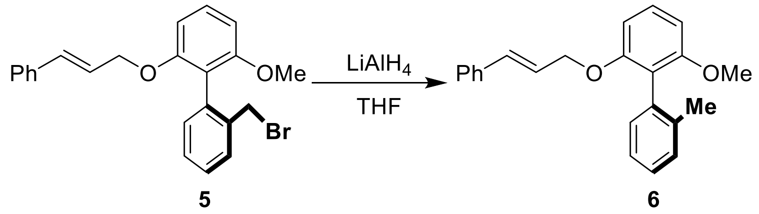

To a round-bottom flask equipped with a magnetic stirring bar, **5** (1.0 equiv) was added and subsequently dissolved in THF (0.4 M). To a solution $LiAlH_4$ (2.3 equiv) was added at 0 °C. The reaction was allowed to stir for 1 h. The reaction was diluted with EtOAc and quenched with a saturated aqueous HCl. The organic and aqueous layers were separated and the aqueous layer was extracted an additional two times with EtOAc. The

combined organic layers were then rinsed with water, dried with anhydrous $MgSO_4$, filtered and concentrated *in vacuo*. The crude material was purified by normal-phase column chromatography using an eluent of 13% EtOAc/Hx to provide **6** (12.9 mg, 65%).

^{1}H NMR (400 MHz, $CDCl_3$) δ 7.31–7.26 (m, 7H), 7.22 (ddd, *J* = 17.1, 10.3, 4.6 Hz, 3H), 6.72–6.64 (m, 2H), 6.40 (d, *J* = 16.0 Hz, 1H), 6.19 (dt, *J* = 16.0, 5.0 Hz, 1H), 4.62 (d, *J* = 4.9 Hz, 2H), 3.73 (s, 3H), 2.11 (s, 3H).
^{13}C NMR (100 MHz, $CDCl_3$) δ 158.0, 156.9, 137.5, 136.8, 134.4, 131.5, 131.0, 129.6, 128.7, 128.6 (2C), 127.7, 127.3, 126.5 (2C), 125.3, 124.9, 119.9, 106.1, 104.4, 69.0, 56.0, 20.0.
IR (FT-ATR, cm^{-1}, CH_2Cl_2) ν_{max} 3685, 3663, 2402, 2371, 2364, 2329, 1799, 1754, 1740, 1722, 1703, 1690, 1659, 1641, 1597, 1563, 1546, 1523, 1512, 1476, 1462, 1442, 1423, 1400, 1380, 1252, 1104, 1078, 971, 924, 915, 865, 823, 789, 766, 739, 730, 705, 695, 671, 657, 637, 625, 610.
HRMS: Exact mass calculated for [$C_{23}H_{22}O_2$] requires *m/z* = 330.162, found *m/z* = 330.1617 (EI).
Optical: $[\alpha]^{20}_D$ = +0.4° (*c* = 1.26, $CHCl_3$, 95:5 er)
HPLC (Chiralpak AD-H, 1% ^{i}PrOH/Hx eluent, 1 mL/min, 254 nm): major enantiomer t_R = 10.3 min, minor enantiomer t_R = 12.7 min.

2'-(Azidomethyl)-2-(cinnamyloxy)-6-methoxy-1,1'-biphenyl (7)

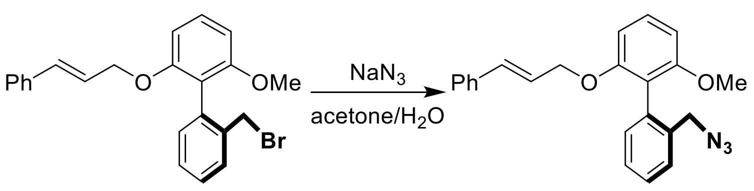

5 **7**

To a round-bottom flask equipped with a magnetic stirring bar, **5** (1.0 equiv) was added and subsequently dissolved in acetone (0.18 M) and H_2O (0.06M). To a solution NaN_3 (2.0 equiv) was added at rt. The reaction was allowed to stir for 12 h. The reaction was diluted with H_2O and extract with EtOAc. The organic and aqueous layers were separated and the aqueous layer was extracted an additional two times with EtOAc, dried with anhydrous $MgSO_4$, filtered and concentrated *in vacuo*. The crude material was purified by normal-phase column chromatography using an eluent of 33% EtOAc/Hx to provide **7** (10.7 mg, 69%).

^{1}H NMR (400 MHz, $CDCl_3$) δ 7.50–7.46 (m, 1H), 7.43–7.35 (m, 2H), 7.31 (d, *J* = 8.4 Hz, 1H), 7.30–7.26 (m, 3H), 7.25–7.18 (m, 3H), 6.67 (dd, *J* = 11.4, 8.4 Hz, 2H), 6.39 (d, *J* = 16.0 Hz, 1H), 6.18 (dt, *J* = 16.0, 5.1 Hz, 1H), 4.61 (dd, *J* = 5.1, 1.5 Hz, 2H), 4.22–4.05 (m, 2H), 3.71 (s, 3H).
δ 7.63 (d, *J* = 6.8 Hz, 1H), 7.51–7.40 (m, 2H), 7.24 (m, 7H), 6.69 (d, *J* = 8.0 Hz, 1H), 6.63 (d, *J* = 8.2 Hz, 1H), 6.40 (d, *J* = 15.7 Hz, 1H), 6.16 (dt, *J* = 15.7, 5.1 Hz, 1H), 4.97 (s, 1H), 4.61 (s, 2H), 4.42 (q, *J* = 11.9 Hz, 2H), 2.17(s, 1H).

^{13}C NMR (100 MHz, $CDCl_3$) δ 157.9, 156.9, 136.7, 135.0, 134.3, 131.7, 131.7, 129.5, 128.6 (2C), 128.5, 127.9, 127.8, 127.7, 126.6 (2C), 124.6, 118.0, 105.8, 104.2, 68.9, 55.9, 52.9.
IR (FT-ATR, cm^{-1}, CH_2Cl_2) ν_{max} 3685, 3662, 3643, 3634, 3581, 3397, 3235, 2415, 2383, 2365, 2352, 2340, 2330, 2315, 2107, 1799, 1691, 1660, 1596, 1546, 1526, 1475, 1442, 1385, 1254, 1199, 1106, 1076, 1049, 1009, 974, 872, 852, 834, 814, 792, 766, 742, 723, 713, 700, 676, 654, 644, 632, 614, 606.
HRMS: Exact mass calculated for [$C_{23}H_{21}N_3O_2$] requires *m/z* = 371.1634, found *m/z* = 371.1631 (EI).
Optical: $[\alpha]^{20}_{D}$ = +15.3° (*c* = 0.43, $CHCl_3$, 95:5 er)
HPLC (Chiralpak AD-H, 1% ^{i}PrOH/Hx eluent, 1 mL/min, 254 nm): major enantiomer t_R = 19.2 min, minor enantiomer t_R = 21.8 min.

8 Experiment for X-ray Crystallography

A colorless plate-like specimen of $C_{23}H_{22}O_4$, approximate dimensions 0.010 mm x 0.090 mm x 0.150 mm, was used for the X-ray crystallographic analysis. The X-ray intensity data were measured (λ = 0.71073 Å). Crystallographic data, atomic coordinates and selective bond lengths of **3ea** are listed in **Supplementary Table 6−13**. The full numbering scheme of compound **3ea** can be found in the full details of the X-ray structure determination (CIF), which is included as Supporting Information. CCDC number 2190478 (**3ea**) contains the supplementary crystallographic data for this paper. These data can be obtained free of charge from The Cambridge Crystallographic Data Center via www.ccdc.cam.ac.uk/data_request/cif.

The X-ray crystallography measurement was performed using the XRD at the National Research Facilities and Equipment Center (NanoBioEnergy Materials Center) at Ewha Womans University.

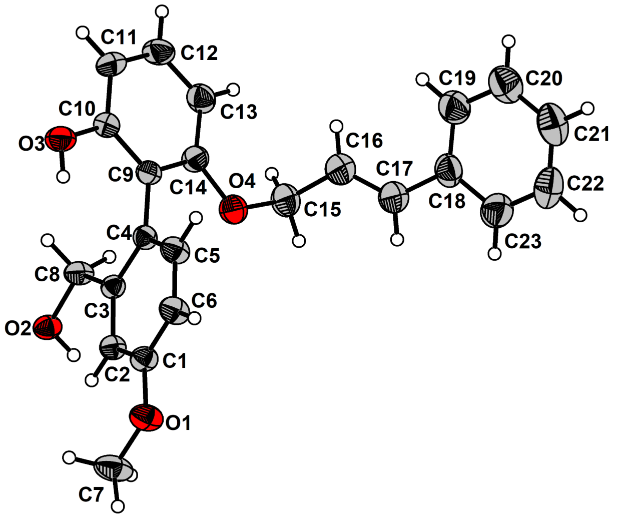

Supplementary Figure 1. Single crystal X-ray structure of **3ea**

Supplementary Table 6. Data collection details for 3ea.

Axis	dx/mm	2θ/°	ω/°	φ/°	χ/°	Width/°	Frames	Time/s	Wavelength/Å	Voltage/kV	Current/mA	Temperature/K
Omega	60.641	18.54	-174.46	153.00	54.74	1.00	206	10.00	0.71073	50	30.0	n/a
Omega	60.641	27.81	-165.19	0.00	54.74	1.00	206	10.00	0.71073	50	30.0	n/a
Omega	60.641	18.54	-174.46	-105.00	54.74	1.00	206	10.00	0.71073	50	30.0	n/a
Omega	60.641	18.54	-174.46	102.00	54.74	1.00	206	10.00	0.71073	50	30.0	n/a
Omega	60.641	18.54	-174.46	0.00	54.74	1.00	206	10.00	0.71073	50	30.0	n/a
Omega	60.641	18.54	-174.46	-156.00	54.74	1.00	206	10.00	0.71073	50	30.0	n/a
Phi	60.641	0.00	0.00	0.00	54.74	360.00	1	108.00	0.71073	50	30.0	n/a

A total of 1237 frames were collected. The total exposure time was 3.46 hours. The frames were integrated with the Bruker SAINT software package using a narrow-frame algorithm. The integration of the data using a monoclinic unit cell yielded a total of 32007 reflections to a maximum θ angle of 28.30° (0.75 Å resolution), of which 4909 were independent (average redundancy 6.520, completeness = 99.8%, R_{int} = 9.24%, R_{sig} = 7.51%) and 2310 (47.06%) were greater than 2σ(F^2). The final cell constants of a = 8.0339(9) Å, b = 7.2593(8) Å, c = 17.3541(19) Å, β = 102.387(4)°, volume = 988.54(19) $Å^3$, are based upon the refinement of the XYZ-centroids of 4540 reflections above 20 σ(I) with 4.806° < 2θ < 42.54°. Data were corrected for absorption effects using the Multi-Scan method (SADABS). The ratio of minimum to maximum apparent transmission was 0.898. The calculated minimum and maximum transmission coefficients (based on crystal size) are 0.9880 and 0.9990.

The structure was solved and refined using the Bruker SHELXTL Software Package, using the space group P 1 21 1, with Z = 2 for the formula unit, $C_{23}H_{22}O_4$. The final anisotropic full-matrix least-squares refinement on F^2 with 247 variables converged at R1 = 5.56%, for the observed data and wR2 = 18.89% for all data. The goodness-of-fit was 1.048. The largest peak in the final difference electron density synthesis was 0.246 $e^-/Å^3$ and the largest hole was -0.296 $e^-/Å^3$ with an RMS deviation of 0.044 $e^-/Å^3$. On the basis of the final model, the calculated density was 1.218 g/cm^3 and F(000), 384 e^-.

Supplementary Table 7. Sample and crystal data for 3ea.

**Chemical formula**	$C_{23}H_{22}O_4$	
**Formula weight**	362.40 g/mol	
**Temperature**	296(2) K	
**Wavelength**	0.71073 Å	
**Crystal size**	0.010 x 0.090 x 0.150 mm	
**Crystal habit**	colorless plate	
**Crystal system**	monoclinic	
**Space group**	$P\ 2_1$	
**Unit cell dimensions**	a = 8.0339(9) Å	α = 90°
	b = 7.2593(8) Å	β = 102.387(4)°
	c = 17.3541(19) Å	γ = 90°
**Volume**	988.54(19) $Å^3$	
**Z**	2	
**Density (calculated)**	1.218 g/cm^3	
**Absorption coefficient**	0.083 mm^{-1}	
**F(000)**	384	

Supplementary Table 8. Data collection and structure refinement for 3ea.

**Theta range for data collection**	2.40 to 28.30°
**Index ranges**	-10≤h≤10, -9≤k≤9, -23≤l≤23
**Reflections collected**	32007
**Independent reflections**	4909 [R(int) = 0.0924]
**Coverage of independent reflections**	99.8%
**Absorption correction**	Multi-Scan
**Max. and min. transmission**	0.9990 and 0.9880
**Structure solution technique**	direct methods
**Structure solution program**	SHELXT 2018/2 (Sheldrick, 2018)
**Refinement method**	Full-matrix least-squares on F^2
**Refinement program**	SHELXL-2018/3 (Sheldrick, 2018)
**Function minimized**	$\Sigma\ w(F_o^2 - F_c^2)^2$

**Data / restraints / parameters**	4909 / 2 / 247	
**Goodness-of-fit on F^2**	1.048	
**Final R indices**	2310 data; I>2σ(I)	R_1 = 0.0556, wR_2 = 0.1315
	all data	R_1 = 0.1603, wR_2 = 0.1889
**Weighting scheme**	$w=1/[\sigma^2(F_o^2)+(0.0761P)^2+0.1444P]$ where $P=(F_o^2+2F_c^2)/3$	
**Absolute structure parameter**	0.0(9)	
**Extinction coefficient**	0.0660(100)	
**Largest diff. peak and hole**	0.246 and -0.296 eÅ^{-3}	
**R.M.S. deviation from mean**	0.044 eÅ^{-3}	

Supplementary Table 9. Atomic coordinates and equivalent isotropic atomic displacement parameters (Å^2) for 3ea.

U(eq) is defined as one third of the trace of the orthogonalized U_{ij} tensor.

	x/a	y/b	z/c	U(eq)
C1	0.1963(6)	0.3340(7)	0.1138(3)	0.0512(13)
C2	0.3443(5)	0.2910(7)	0.0892(3)	0.0473(12)
C3	0.4900(5)	0.3960(6)	0.1146(3)	0.0428(11)
C4	0.4896(6)	0.5435(7)	0.1653(3)	0.0443(11)
C5	0.3374(6)	0.5859(8)	0.1879(3)	0.0583(14)
C6	0.1918(6)	0.4822(8)	0.1622(3)	0.0601(15)
C7	0.0469(8)	0.0858(10)	0.0393(4)	0.085(2)
C8	0.6520(6)	0.3519(7)	0.0878(3)	0.0531(12)
C9	0.6487(6)	0.6519(7)	0.1968(3)	0.0469(12)
C10	0.6831(6)	0.8145(7)	0.1615(3)	0.0527(12)
C11	0.8297(7)	0.9163(8)	0.1921(3)	0.0656(15)
C12	0.9413(7)	0.8538(9)	0.2585(3)	0.0695(16)
C13	0.9114(6)	0.6921(9)	0.2950(3)	0.0641(15)
C14	0.7658(6)	0.5912(7)	0.2630(3)	0.0549(13)
C15	0.8321(7)	0.3531(10)	0.3638(3)	0.0727(16)
C16	0.7987(8)	0.4478(10)	0.4348(3)	0.0796(18)
C17	0.7188(8)	0.3758(10)	0.4856(3)	0.0798(18)
C18	0.6749(8)	0.4632(11)	0.5551(3)	0.0745(17)
C19	0.7295(8)	0.6351(11)	0.5820(4)	0.086(2)

	x/a	y/b	z/c	U(eq)
C20	0.6841(9)	0.7123(14)	0.6460(4)	0.098(2)
C21	0.5793(10)	0.6216(14)	0.6847(4)	0.097(2)
C22	0.5191(9)	0.4518(14)	0.6595(4)	0.097(2)
C23	0.5674(9)	0.3720(12)	0.5951(4)	0.092(2)
O1	0.0473(4)	0.2366(6)	0.0916(2)	0.0673(11)
O2	0.6402(4)	0.1967(5)	0.03562(18)	0.0523(9)
O3	0.5706(5)	0.8709(6)	0.0960(2)	0.0678(10)
O4	0.7252(5)	0.4256(5)	0.2925(2)	0.0688(11)

Supplementary Table 10. Bond lengths (Å) for 3ea.

C1-C6	1.370(7)	C1-O1	1.372(5)
C1-C2	1.383(6)	C2-C3	1.388(6)
C2-H2A	0.93	C3-C4	1.387(6)
C3-C8	1.508(6)	C4-C5	1.396(7)
C4-C9	1.500(6)	C5-C6	1.382(7)
C5-H5	0.93	C6-H6	0.93
C7-O1	1.422(7)	C7-H7A	0.96
C7-H7B	0.96	C7-H7C	0.96
C8-O2	1.436(6)	C8-H8A	0.97
C8-H8B	0.97	C9-C10	1.386(7)
C9-C14	1.390(7)	C10-O3	1.355(6)
C10-C11	1.395(7)	C11-C12	1.377(8)
C11-H11	0.93	C12-C13	1.378(8)
C12-H12	0.93	C13-C14	1.391(7)
C13-H13	0.93	C14-O4	1.374(6)
C15-O4	1.446(6)	C15-C16	1.485(8)
C15-H15A	0.97	C15-H15B	0.97
C16-C17	1.306(8)	C16-H16	0.93
C17-C18	1.470(9)	C17-H17	0.93
C18-C19	1.371(9)	C18-C23	1.387(9)
C19-C20	1.361(9)	C19-H19	0.93
C20-C21	1.355(10)	C20-H20	0.93
C21-C22	1.362(12)	C21-H21	0.93
C22-C23	1.385(10)	C22-H22	0.93
C23-H23	0.93	O2-H2	0.82
O3-H3	0.82		

Supplementary Table 11. Bond angles (°) for 3ea.

C6-C1-O1	116.1(4)	C6-C1-C2	120.3(4)
O1-C1-C2	123.6(5)	C1-C2-C3	120.0(4)
C1-C2-H2A	120.0	C3-C2-H2A	120.0
C4-C3-C2	120.6(4)	C4-C3-C8	118.7(4)
C2-C3-C8	120.7(4)	C3-C4-C5	118.0(4)
C3-C4-C9	121.2(4)	C5-C4-C9	120.8(4)
C6-C5-C4	121.4(5)	C6-C5-H5	119.3
C4-C5-H5	119.3	C1-C6-C5	119.6(5)
C1-C6-H6	120.2	C5-C6-H6	120.2
O1-C7-H7A	109.5	O1-C7-H7B	109.5
H7A-C7-H7B	109.5	O1-C7-H7C	109.5
H7A-C7-H7C	109.5	H7B-C7-H7C	109.5
O2-C8-C3	114.6(4)	O2-C8-H8A	108.6
C3-C8-H8A	108.6	O2-C8-H8B	108.6
C3-C8-H8B	108.6	H8A-C8-H8B	107.6
C10-C9-C14	118.2(4)	C10-C9-C4	121.4(4)
C14-C9-C4	120.5(4)	O3-C10-C9	117.9(4)
O3-C10-C11	121.1(5)	C9-C10-C11	121.0(5)
C12-C11-C10	119.3(5)	C12-C11-H11	120.4
C10-C11-H11	120.4	C11-C12-C13	121.2(5)
C11-C12-H12	119.4	C13-C12-H12	119.4
C12-C13-C14	118.7(5)	C12-C13-H13	120.7
C14-C13-H13	120.7	O4-C14-C9	114.4(4)
O4-C14-C13	124.0(5)	C9-C14-C13	121.6(5)
O4-C15-C16	111.2(5)	O4-C15-H15A	109.4
C16-C15-H15A	109.4	O4-C15-H15B	109.4
C16-C15-H15B	109.4	H15A-C15-H15B	108.0
C17-C16-C15	125.2(7)	C17-C16-H16	117.4
C15-C16-H16	117.4	C16-C17-C18	128.3(7)
C16-C17-H17	115.9	C18-C17-H17	115.9
C19-C18-C23	116.8(7)	C19-C18-C17	123.9(6)
C23-C18-C17	119.3(7)	C20-C19-C18	122.1(7)
C20-C19-H19	119.0	C18-C19-H19	119.0
C21-C20-C19	120.5(9)	C21-C20-H20	119.7
C19-C20-H20	119.7	C20-C21-C22	119.7(8)
C20-C21-H21	120.1	C22-C21-H21	120.1

C21-C22-C23	119.7(8)	C21-C22-H22	120.1
C23-C22-H22	120.1	C22-C23-C18	121.1(8)
C22-C23-H23	119.5	C18-C23-H23	119.5
C1-O1-C7	117.3(4)	C8-O2-H2	109.5
C10-O3-H3	109.5	C14-O4-C15	119.6(4)

Supplementary Table 12. Anisotropic atomic displacement parameters (Å^2) for 3ea.

The anisotropic atomic displacement factor exponent takes the form: $-2\pi^2[h^2 a^{*2} U_{11} + ... + 2 h k a^* b^* U_{12}]$

	U_{11}	U_{22}	U_{33}	U_{23}	U_{13}	U_{12}
C1	0.038(2)	0.058(3)	0.059(3)	-0.005(3)	0.012(2)	-0.006(2)
C2	0.045(3)	0.045(3)	0.054(3)	-0.005(2)	0.015(2)	0.001(2)
C3	0.038(2)	0.044(3)	0.045(2)	0.001(2)	0.0085(19)	-0.002(2)
C4	0.042(2)	0.047(3)	0.043(2)	-0.002(2)	0.009(2)	-0.001(2)
C5	0.050(3)	0.064(4)	0.061(3)	-0.013(3)	0.014(2)	-0.002(3)
C6	0.048(3)	0.068(4)	0.067(3)	-0.017(3)	0.019(3)	-0.001(3)
C7	0.060(4)	0.090(5)	0.110(5)	-0.043(4)	0.029(3)	-0.025(3)
C8	0.046(2)	0.048(3)	0.068(3)	-0.013(3)	0.019(2)	-0.006(2)
C9	0.046(2)	0.047(3)	0.048(3)	-0.005(2)	0.011(2)	-0.003(2)
C10	0.054(3)	0.053(3)	0.050(3)	-0.004(3)	0.009(2)	-0.006(2)
C11	0.064(3)	0.059(4)	0.073(3)	0.001(3)	0.013(3)	-0.021(3)
C12	0.053(3)	0.076(4)	0.076(4)	-0.010(3)	0.007(3)	-0.020(3)
C13	0.054(3)	0.078(4)	0.057(3)	-0.002(3)	0.004(2)	-0.004(3)
C14	0.056(3)	0.053(3)	0.057(3)	-0.001(3)	0.015(3)	-0.003(3)
C15	0.074(4)	0.080(4)	0.061(3)	0.006(3)	0.006(3)	0.006(3)
C16	0.084(4)	0.085(4)	0.068(4)	0.003(4)	0.010(3)	-0.011(4)
C17	0.086(4)	0.082(4)	0.067(4)	0.007(4)	0.006(3)	-0.009(4)
C18	0.071(4)	0.096(5)	0.054(3)	0.004(4)	0.007(3)	-0.002(4)
C19	0.078(4)	0.105(6)	0.074(4)	-0.004(4)	0.013(3)	-0.012(4)
C20	0.092(5)	0.119(6)	0.083(5)	-0.015(5)	0.020(4)	0.002(5)
C21	0.082(5)	0.141(8)	0.068(4)	0.000(5)	0.012(4)	0.022(5)
C22	0.091(5)	0.127(7)	0.075(5)	0.029(5)	0.023(4)	0.011(5)
C23	0.095(5)	0.097(5)	0.083(4)	0.012(4)	0.013(4)	-0.008(4)
O1	0.0446(19)	0.078(3)	0.084(3)	-0.025(2)	0.0234(18)	-0.0117(18)
O2	0.0515(18)	0.0436(19)	0.065(2)	-0.0068(18)	0.0199(16)	-0.0018(16)

	U_{11}	U_{22}	U_{33}	U_{23}	U_{13}	U_{12}
O3	0.068(2)	0.060(2)	0.068(2)	0.003(2)	-0.0021(19)	-0.0159(19)
O4	0.073(2)	0.070(3)	0.056(2)	0.009(2)	-0.0040(17)	-0.006(2)

Supplementary Table 13. Hydrogen atomic coordinates and isotropic atomic displacement parameters (Å^2) for 3ea.

	x/a	y/b	z/c	U(eq)
H2A	0.3461	0.1916	0.0555	0.057
H5	0.3339	0.6860	0.2210	0.07
H6	0.0914	0.5130	0.1776	0.072
H7A	0.0753	0.1289	-0.0086	0.127
H7B	-0.0643	0.0305	0.0275	0.127
H7C	0.1293	-0.0039	0.0637	0.127
H8A	0.6851	0.4592	0.0613	0.064
H8B	0.7416	0.3284	0.1339	0.064
H11	0.8518	1.0251	0.1678	0.079
H12	1.0386	0.9220	0.2793	0.083
H13	0.9871	0.6513	0.3400	0.077
H15A	0.9508	0.3689	0.3616	0.087
H15B	0.8106	0.2223	0.3676	0.087
H16	0.8378	0.5682	0.4438	0.096
H17	0.6854	0.2536	0.4767	0.096
H19	0.7997	0.7010	0.5558	0.103
H20	0.7254	0.8282	0.6633	0.117
H21	0.5486	0.6751	0.7283	0.117
H22	0.4459	0.3896	0.6854	0.117
H23	0.5270	0.2554	0.5785	0.111
H2	0.6151	0.1044	0.0579	0.078
H3	0.4938	0.7949	0.0845	0.102

9 Supplementary References

1. Fulmer, G. R., *et al.* NMR Chemical Shifts of Trace Impurities: Common Laboratory Solvents, Organics, and Gases in Deuterated Solvents Relevant to the Organometallic Chemist. *Organometallics* **29**, 2176−2179 (2010).

2. Demchuk, O. M., Kapłon, K., Mazur, L., Strzelecka, D., Pietrusiewicz, K. M. Readily available catalysts for demanding Suzuki–Miyaura couplings under mild conditions. *Tetrahedron* **72**, 6668−6677 (2016).

3. Munday, E. S., *et al.* Isothiourea-Catalyzed Atropselective Acylation of Biaryl Phenols via Sequential Desymmetrization/Kinetic Resolution. *Angew. Chem. Int. Ed.* **59**, 7897−7905 (2020).

4. Bovy, P. R., Collins, J. T., Olins, G. M., McMahon, E. G., Hutton, W. C. Conformationally restricted polysubstituted biphenyl derivatives with angiotensin II receptors antagonist properties. *J. Med. Chem.* **34**, 2410−2414 (1991).

5. Li, Y., Ding, Y.-J., Wang, J.-Y., Su, Y.-M., Wang, X.-S. Pd-Catalyzed C–H Lactonization for Expedient Synthesis of Biaryl Lactones and Total Synthesis of Cannabinol. *Org. Lett.* **15**, 2574−2577 (2013).

6. Mori, K., Ichikawa, Y., Kobayashi, M., Shibata, Y., Yamanaka, M., Akiyama, T. Enantioselective Synthesis of Multisubstituted Biaryl Skeleton by Chiral Phosphoric Acid Catalyzed Desymmetrization/Kinetic Resolution Sequence. *J. Am. Chem. Soc.* **135**, 3964−3970 (2013).
