# Supplementary material for: Atroposelective desymmetrization of 2-arylresorcinols via Tsuji-Trost allylation
Source: Commun Chem. 2023 Feb 25;6:42. doi: 10.1038/s42004-023-00839-z (PMC9968306; doi:10.1038/s42004-023-00839-z)
Supplement: Supplementary file 2 — Description of Additional Supplementary Files [file 42004_2023_839_MOESM2_ESM.pdf]

Description of Additional Supplementary Files

File name: Supplementary Data 1

Description: X-ray Crystallographic Data for 3ea

File name: Supplementary Data 2

Description: Copies of 1H and 13C NMR Spectra

File name: Supplementary Data 3

Description: Copies of HPLC Chromatograms

File name: Supplementary Data 4

Description: Computational Chemistry Data
