# Supplementary material for: Atroposelective desymmetrization of 2-arylresorcinols via Tsuji-Trost allylation
Source: Commun Chem. 2023 Feb 25;6:42. doi: 10.1038/s42004-023-00839-z (PMC9968306; doi:10.1038/s42004-023-00839-z)
Supplement: Supplementary file 4 — Supplementary Data 2 [file 42004_2023_839_MOESM4_ESM.pdf]

Copies of ^{1}H and ^{13}C NMR Spectra

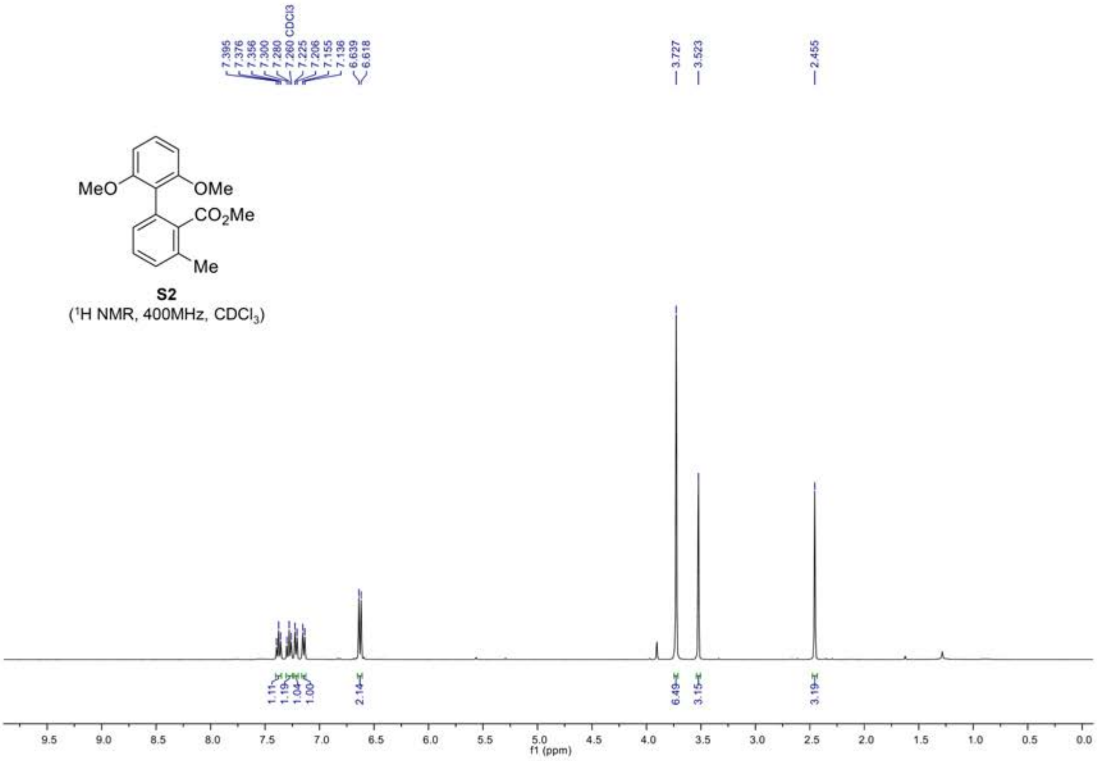

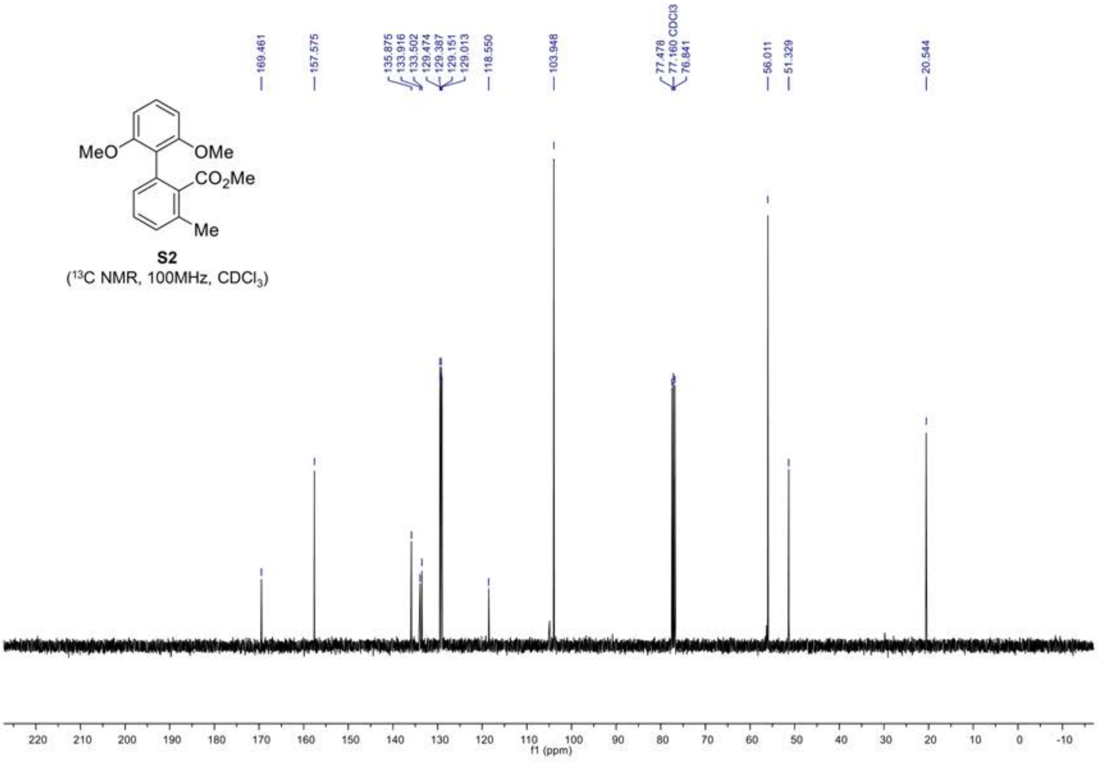

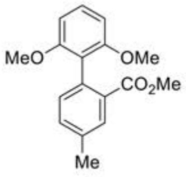

S3
(^{1}H NMR, 400MHz, $CDCl_3$)

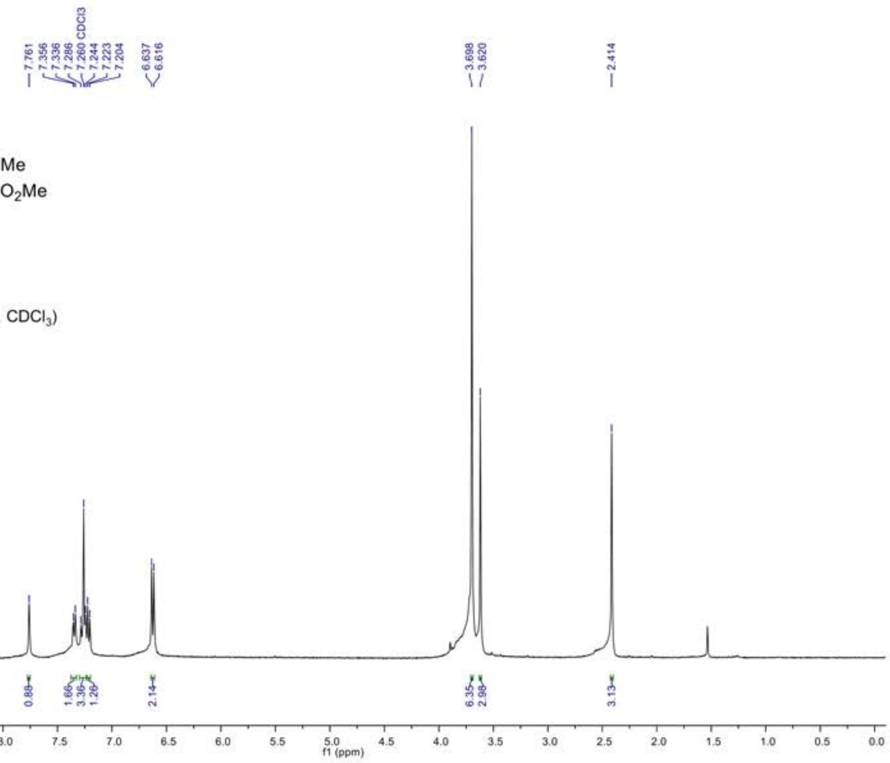

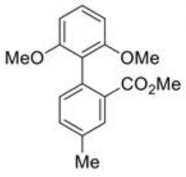

S3
(^{13}C NMR, 100MHz, $CDCl_3$)

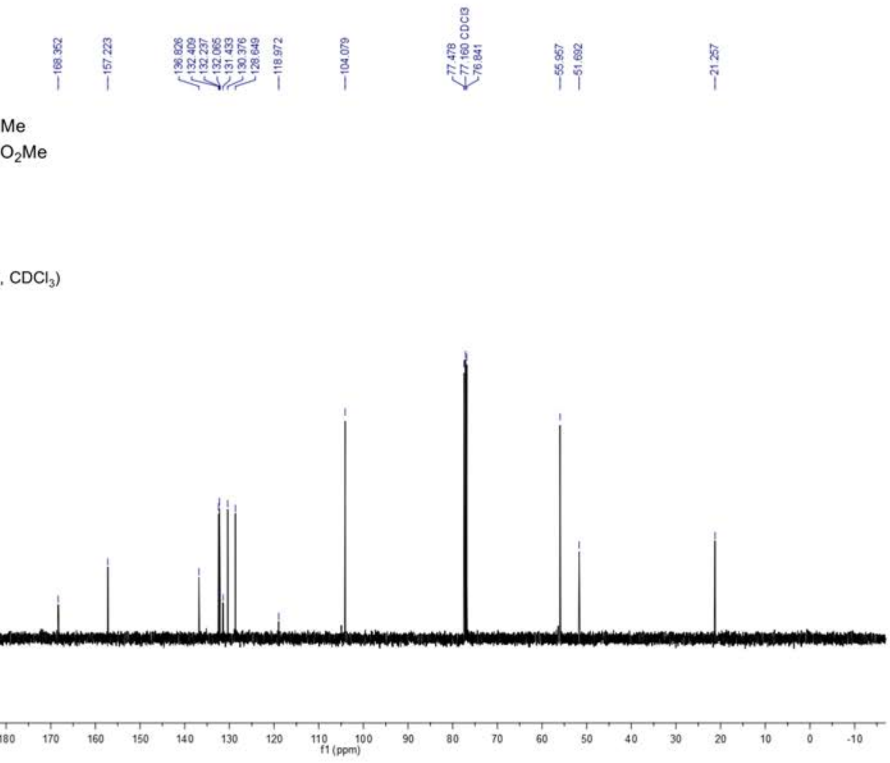

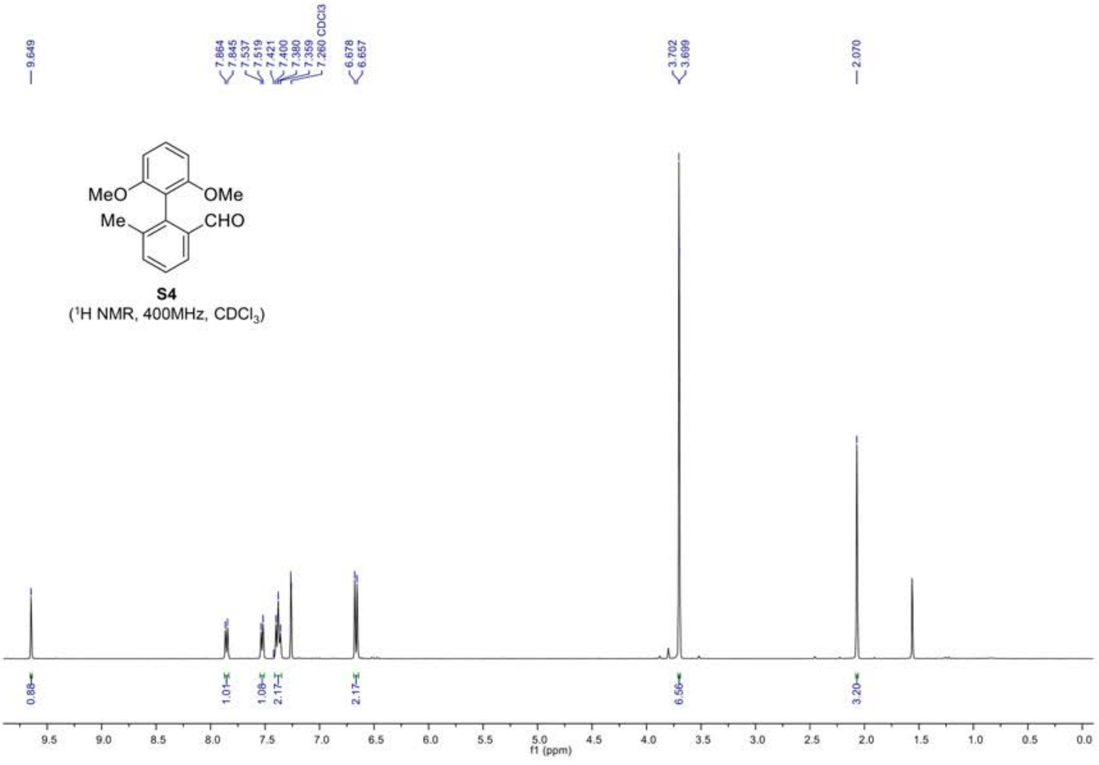
9.649
7.864
7.845
7.537
7.519
7.421
7.400
7.380
7.359
7.260 CDCl3
6.678
6.657
3.702
3.699
2.070
MeO
OMe
Me
CHO
S4
(¹H NMR, 400MHz, CDCl₃)
0.88
1.01
1.08
2.17
2.17
6.56
3.20
f1 (ppm)

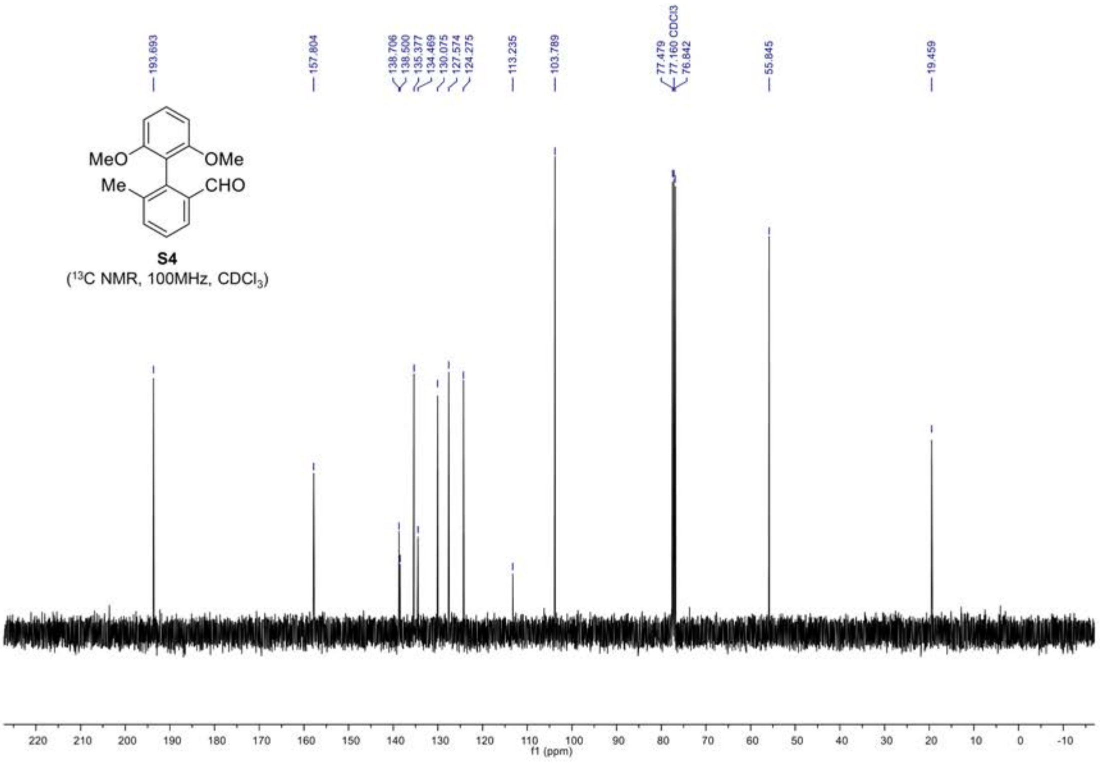
193.693
157.804
138.706
138.500
135.377
134.469
130.075
127.574
124.275
113.235
103.789
77.479
77.160 CDCl3
76.842
55.845
19.459
MeO
OMe
Me
CHO
S4
(¹³C NMR, 100MHz, CDCl₃)
f1 (ppm)

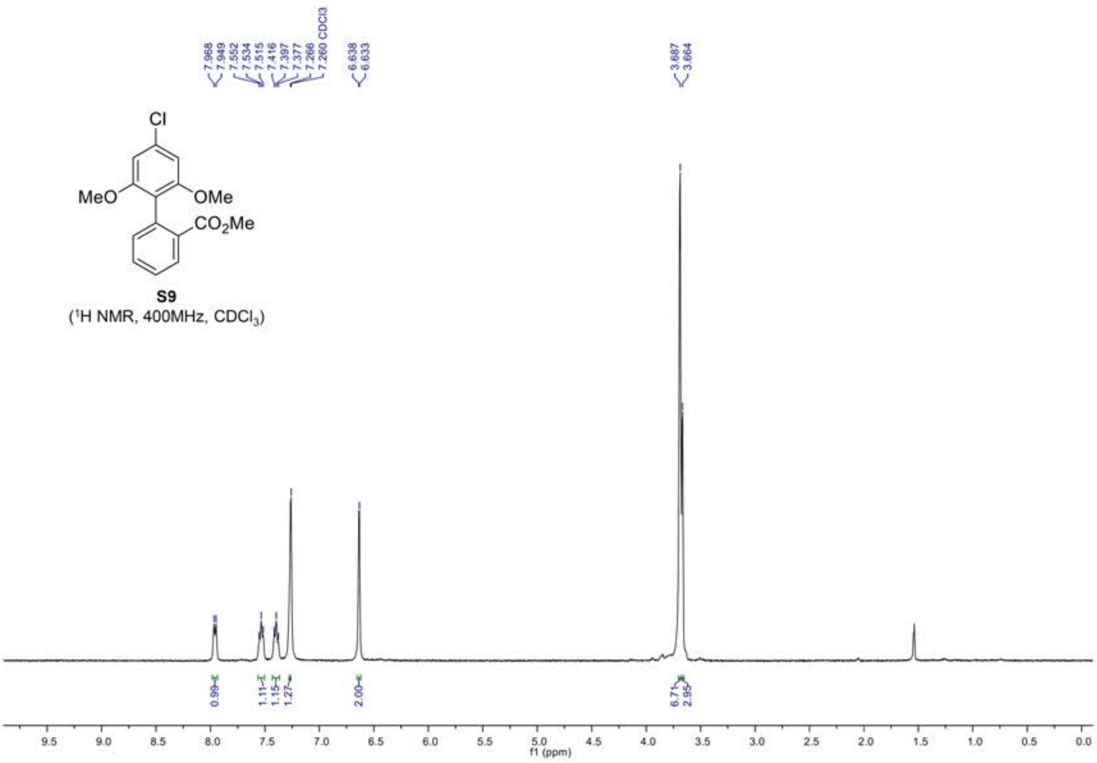

S9
(^{1}H NMR, 400MHz, $CDCl_3$)

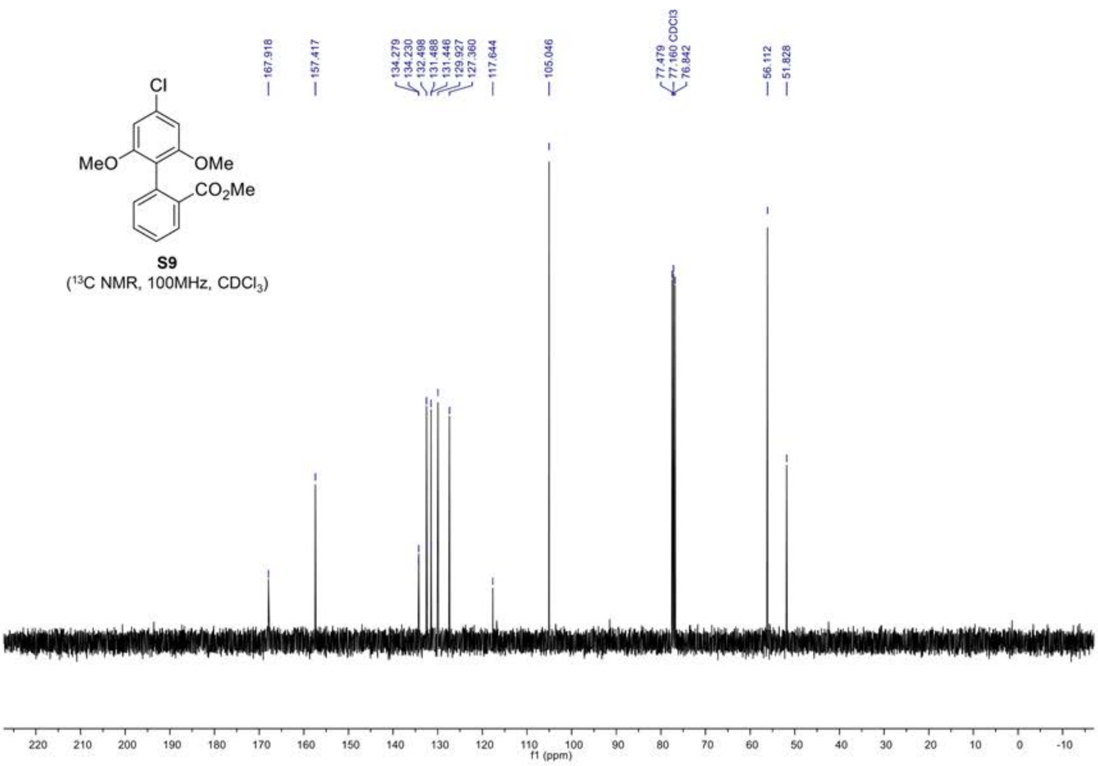

S9
(^{13}C NMR, 100MHz, $CDCl_3$)

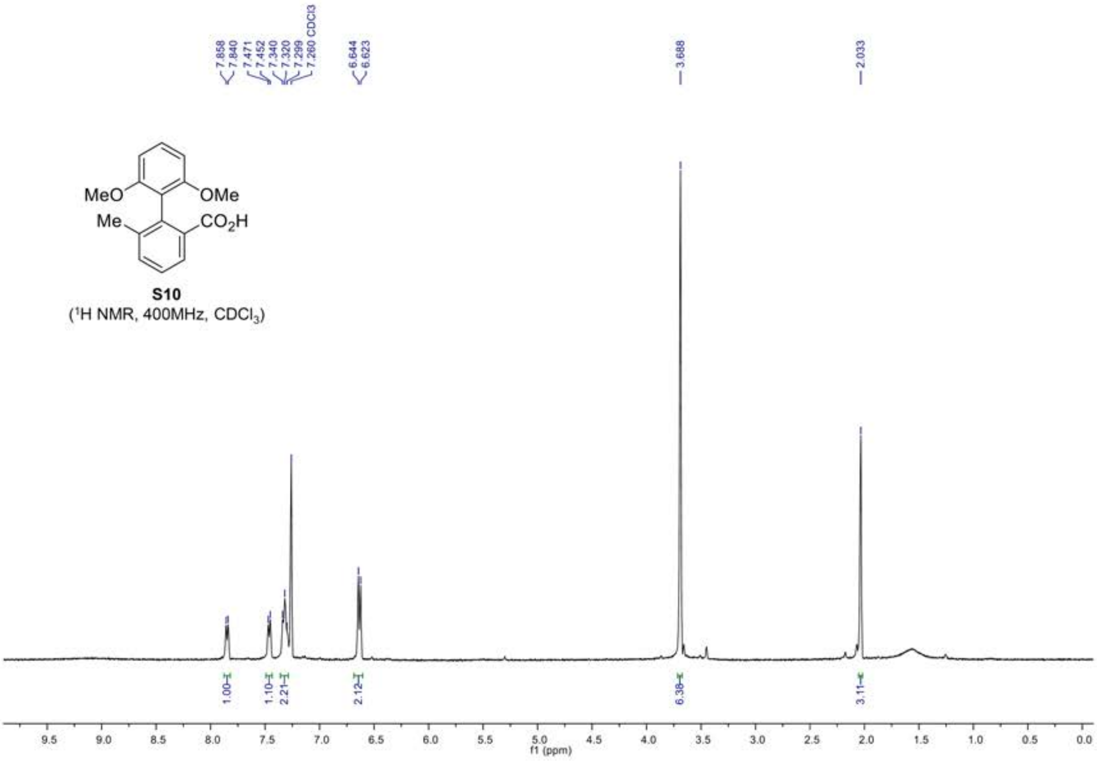
7.858
7.840
7.471
7.452
7.340
7.320
7.299
7.260 CDCl3
6.644
6.623
3.688
2.033
MeO
OMe
Me
CO2H
S10
(1H NMR, 400MHz, CDCl3)
1.00
1.10
2.21
2.12
6.38
3.11
f1 (ppm)

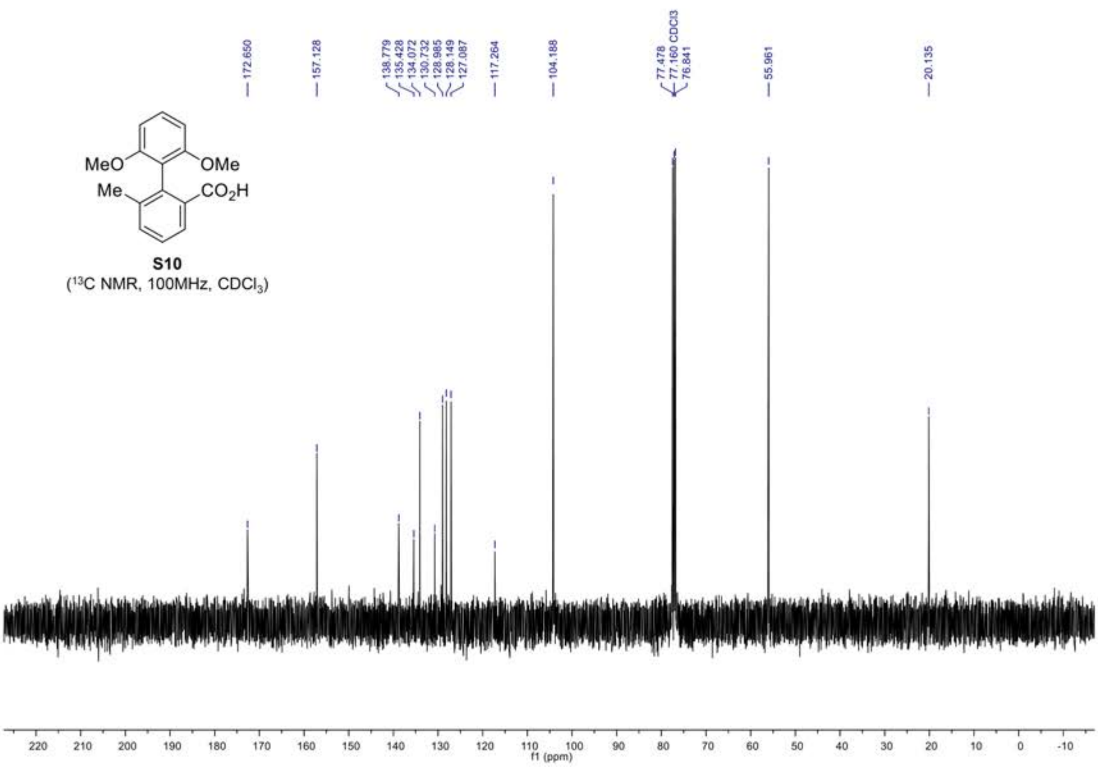
172.650
157.128
138.779
135.428
134.072
130.732
128.985
128.149
127.087
117.264
104.188
77.478
77.160 CDCl3
76.841
55.961
20.135
MeO
OMe
Me
CO2H
S10
(13C NMR, 100MHz, CDCl3)
f1 (ppm)

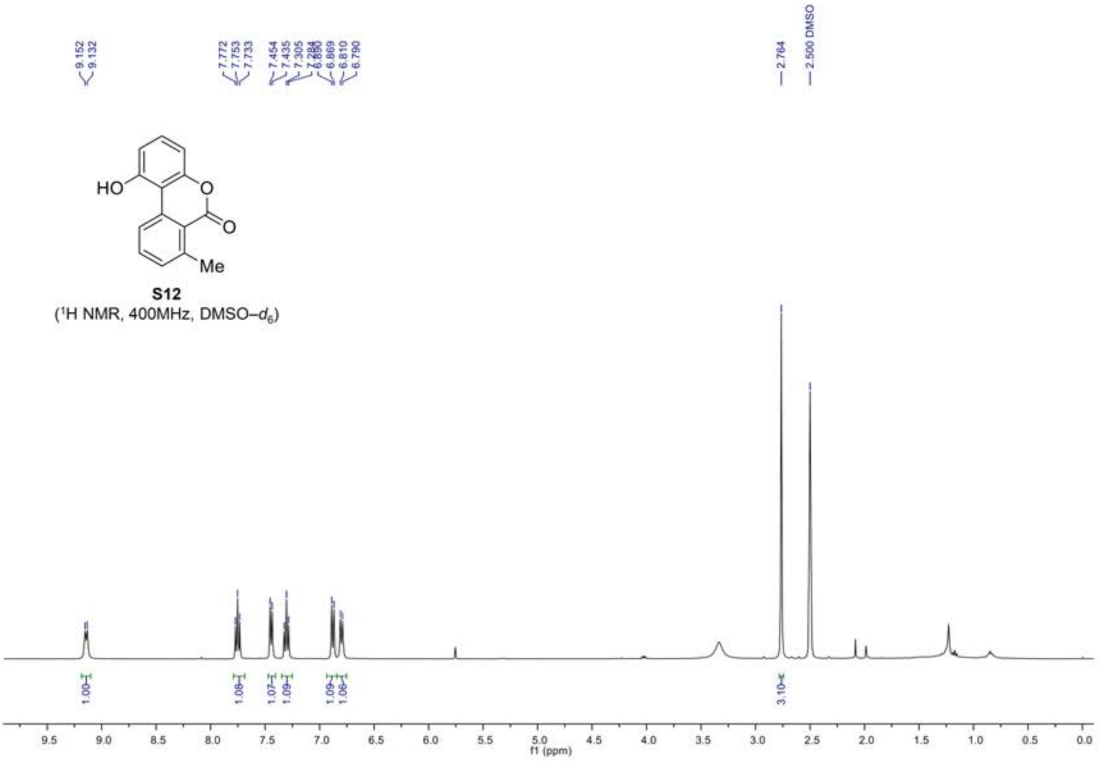
9.152
9.132
7.772
7.753
7.733
7.454
7.435
7.305
6.890
6.869
6.810
6.790
2.764
2.500 DMSO
HO
O
O
Me
S12
(^{1}H NMR, 400MHz, DMSO–d_6)
1.00
1.08
1.07
1.09
1.09
1.06
3.10
f1 (ppm)

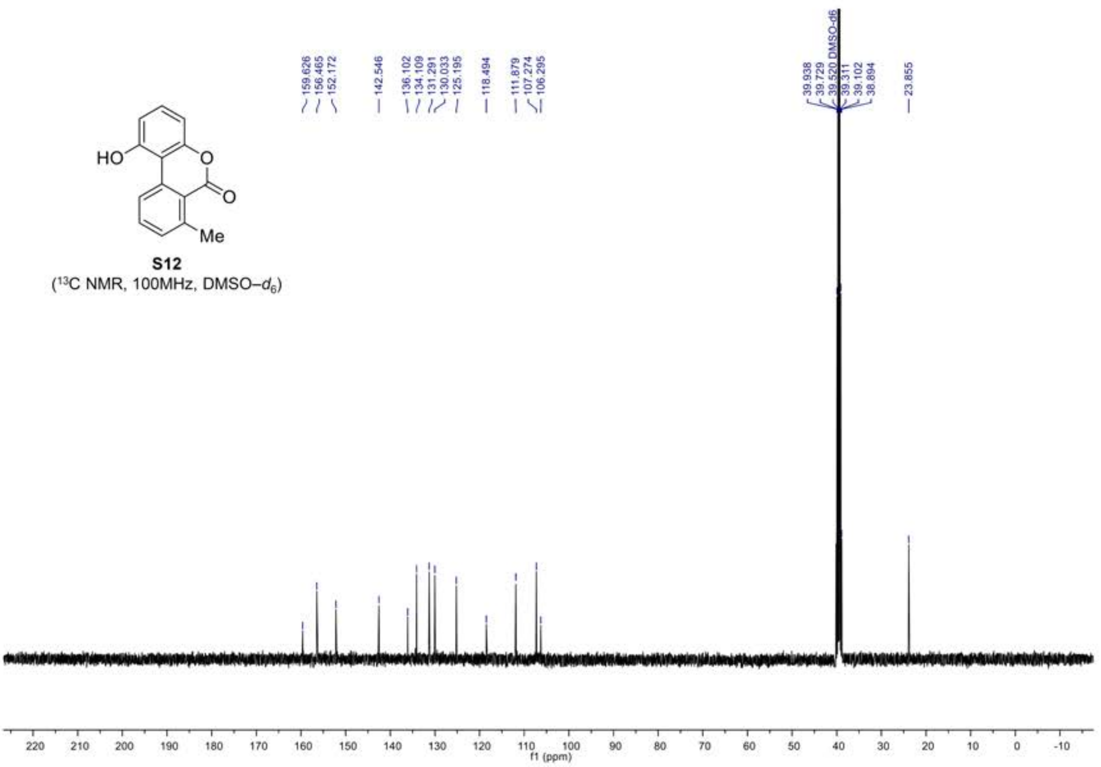
159.626
156.465
152.172
142.546
136.102
134.109
131.291
130.033
125.195
118.494
111.879
107.274
106.295
39.938
39.729
39.520 DMSO-d6
39.311
39.102
38.894
23.855
HO
O
O
Me
S12
(^{13}C NMR, 100MHz, DMSO–d_6)
f1 (ppm)

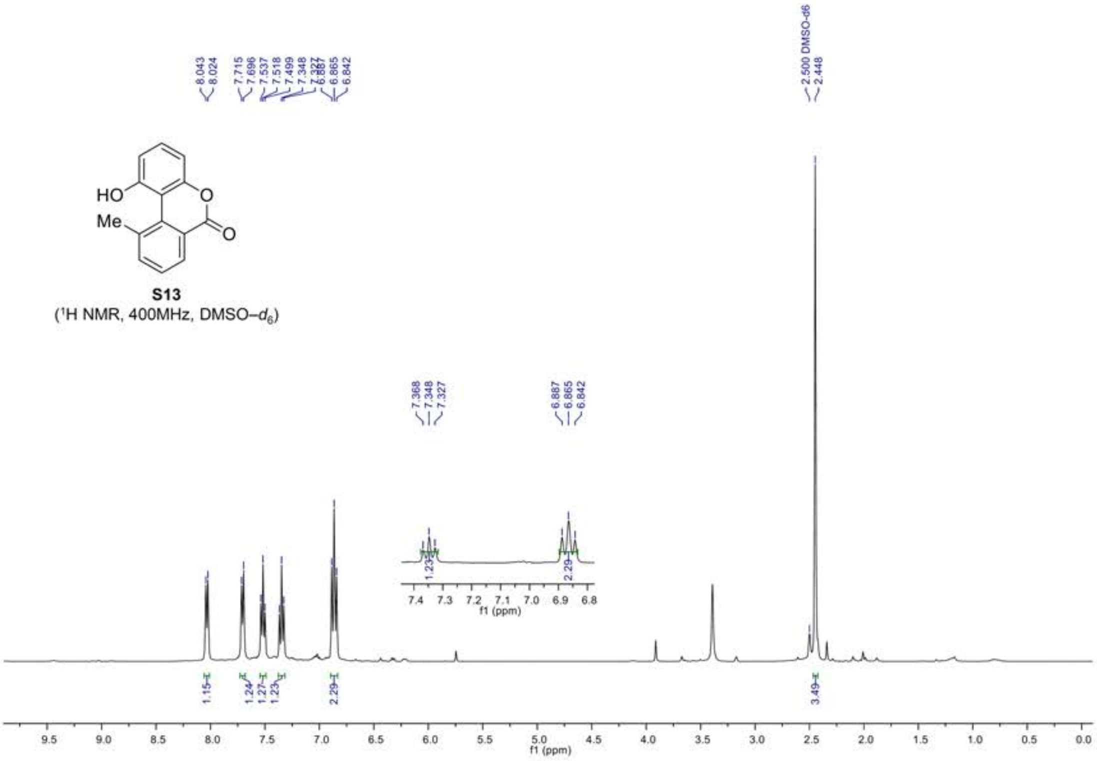

S13
(^{1}H NMR, 400MHz, DMSO–d_6)

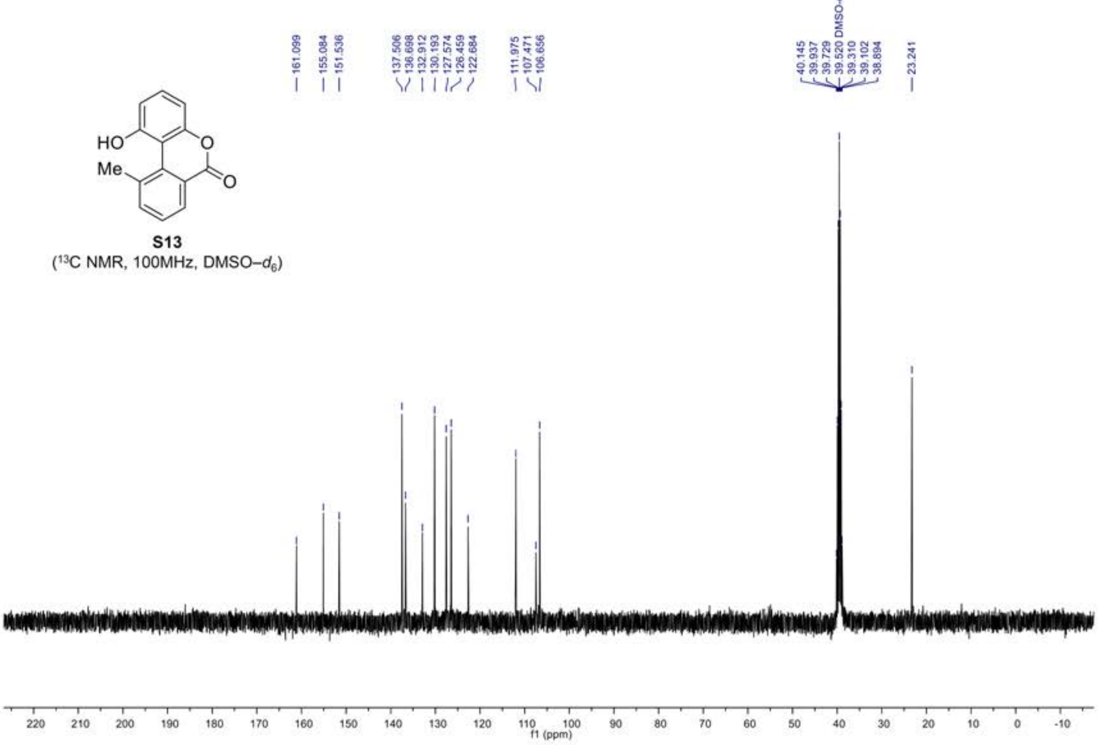

S13
(^{13}C NMR, 100MHz, DMSO–d_6)

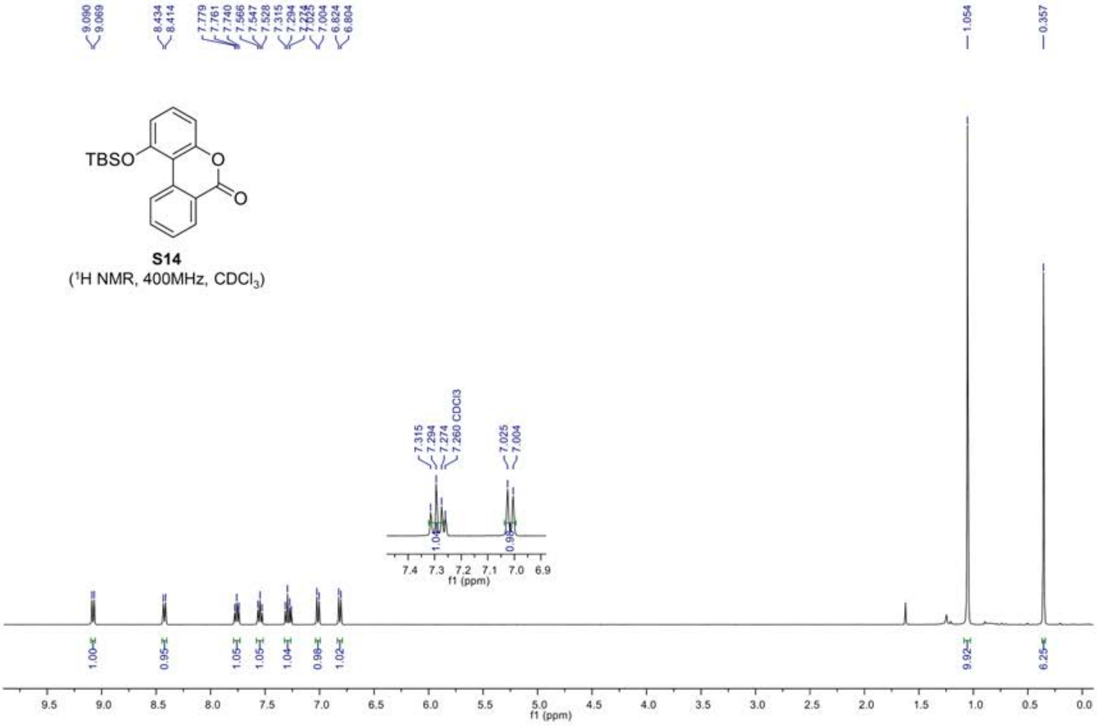

TBSO
O
O
S14
(1H NMR, 400MHz, CDCl3)
9.090
9.069
8.434
8.414
7.779
7.761
7.740
7.566
7.547
7.528
7.315
7.294
7.004
6.824
6.804
1.054
0.357
7.315
7.294
7.274
7.260 CDCl3
7.025
7.004
7.4 7.3 7.2 7.1 7.0 6.9
f1 (ppm)
1.00
0.95
1.05
1.05
1.04
0.98
1.02
9.92
6.25
9.5 9.0 8.5 8.0 7.5 7.0 6.5 6.0 5.5 5.0 4.5 4.0 3.5 3.0 2.5 2.0 1.5 1.0 0.5 0.0
f1 (ppm)

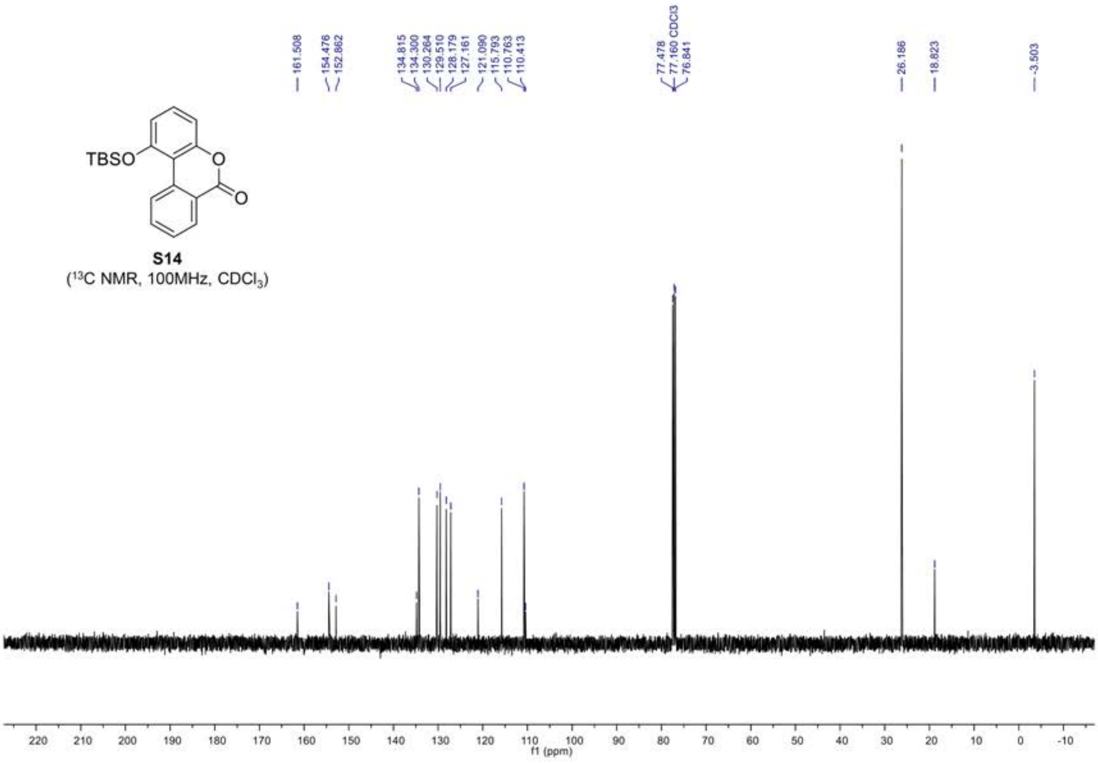

TBSO
O
O
S14
(13C NMR, 100MHz, CDCl3)
161.508
154.476
152.862
134.815
134.300
130.264
129.510
128.179
127.161
121.090
115.793
110.763
110.413
77.478
77.160 CDCl3
76.841
26.186
18.823
-3.503
220 210 200 190 180 170 160 150 140 130 120 110 100 90 80 70 60 50 40 30 20 10 0 -10
f1 (ppm)

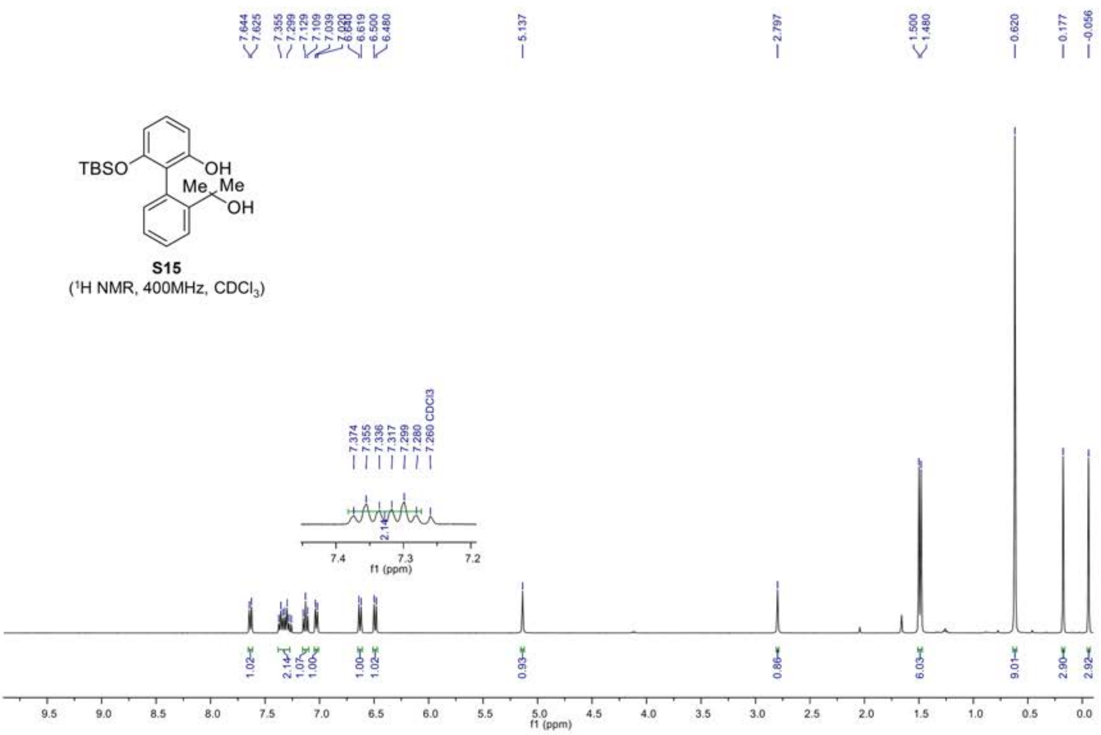
7.644
7.625
7.355
7.299
7.129
7.109
7.039
6.619
6.500
6.480
5.137
2.797
1.500
1.480
0.620
0.177
-0.056
TBSO
OH
Me
Me
OH
S15
(1H NMR, 400MHz, CDCl3)
7.374
7.355
7.336
7.317
7.299
7.280
7.260 CDCl3
2.14
7.4
7.3
7.2
f1 (ppm)
1.02
2.14
1.07
1.00
1.00
1.02
0.93
0.86
6.03
9.01
2.90
2.92
9.5
9.0
8.5
8.0
7.5
7.0
6.5
6.0
5.5
5.0
4.5
4.0
3.5
3.0
2.5
2.0
1.5
1.0
0.5
0.0
f1 (ppm)

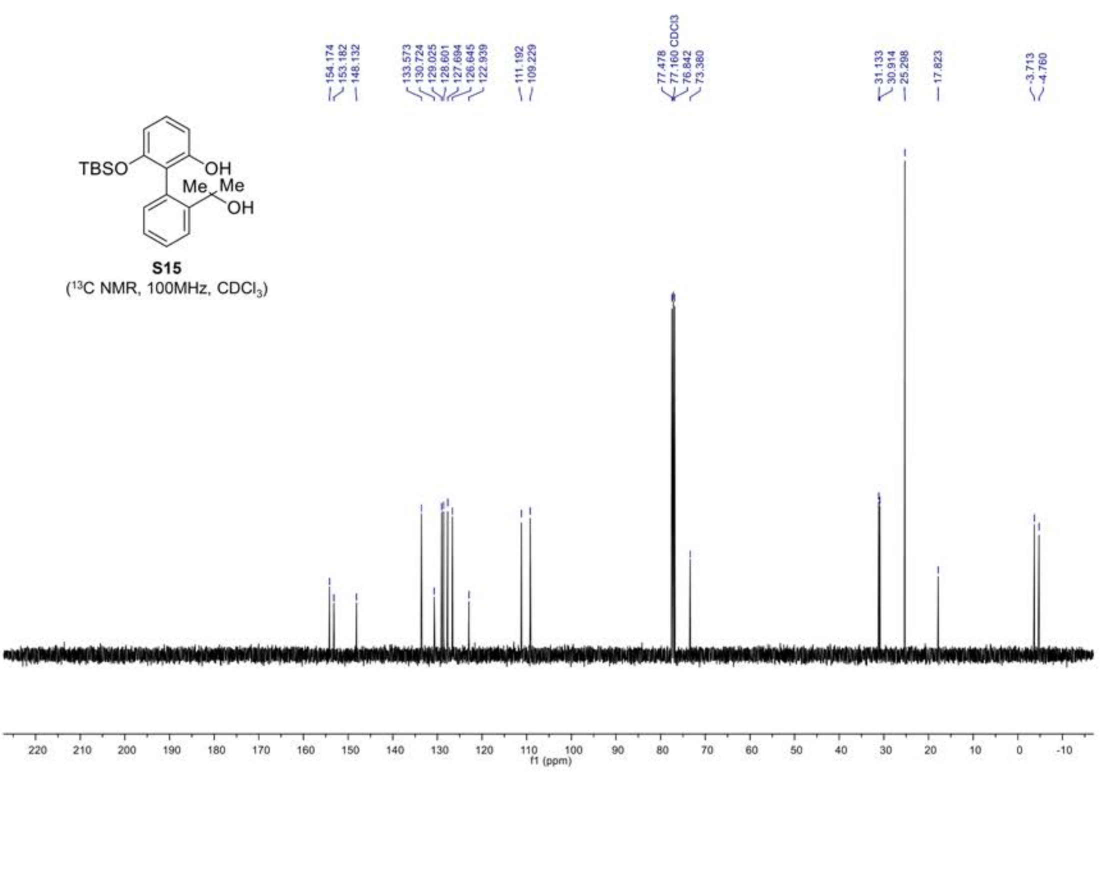
154.174
153.182
148.132
133.573
130.724
129.025
128.601
127.694
126.645
122.939
111.192
109.229
77.478
77.160 CDCl3
76.842
73.380
31.133
30.914
25.298
17.823
-3.713
-4.760
TBSO
OH
Me
Me
OH
S15
(13C NMR, 100MHz, CDCl3)
220
210
200
190
180
170
160
150
140
130
120
110
100
90
80
70
60
50
40
30
20
10
0
-10
f1 (ppm)

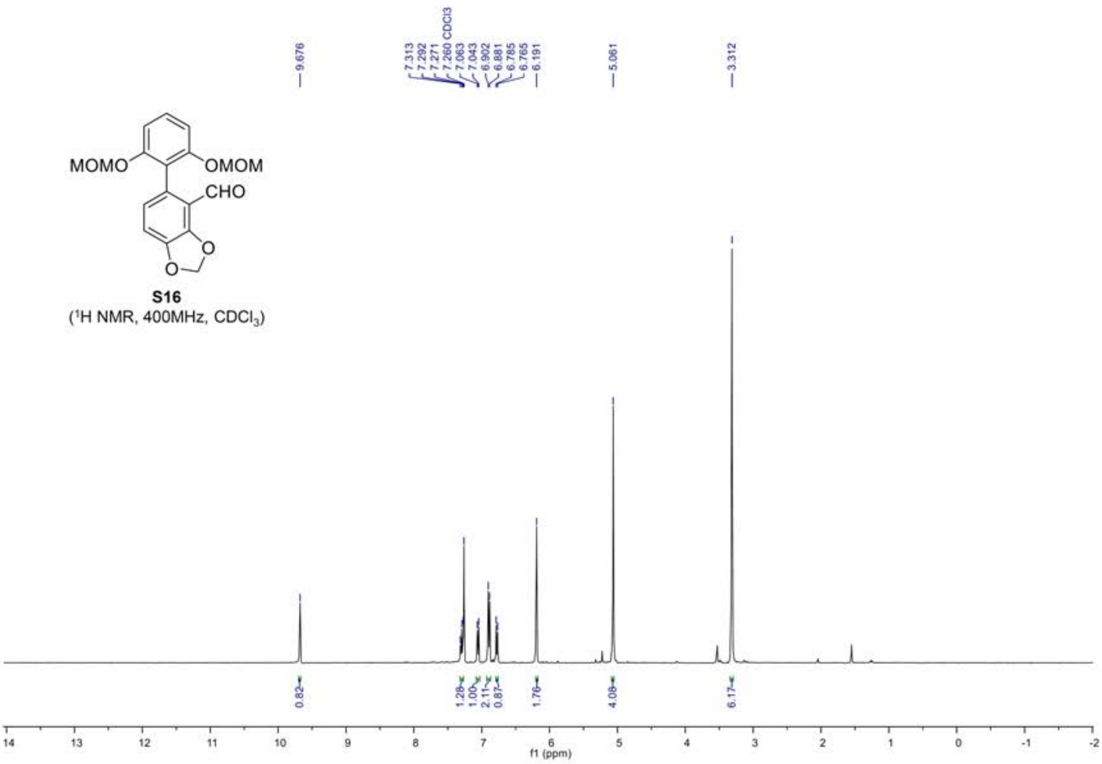

S16
(^{1}H NMR, 400MHz, $CDCl_3$)

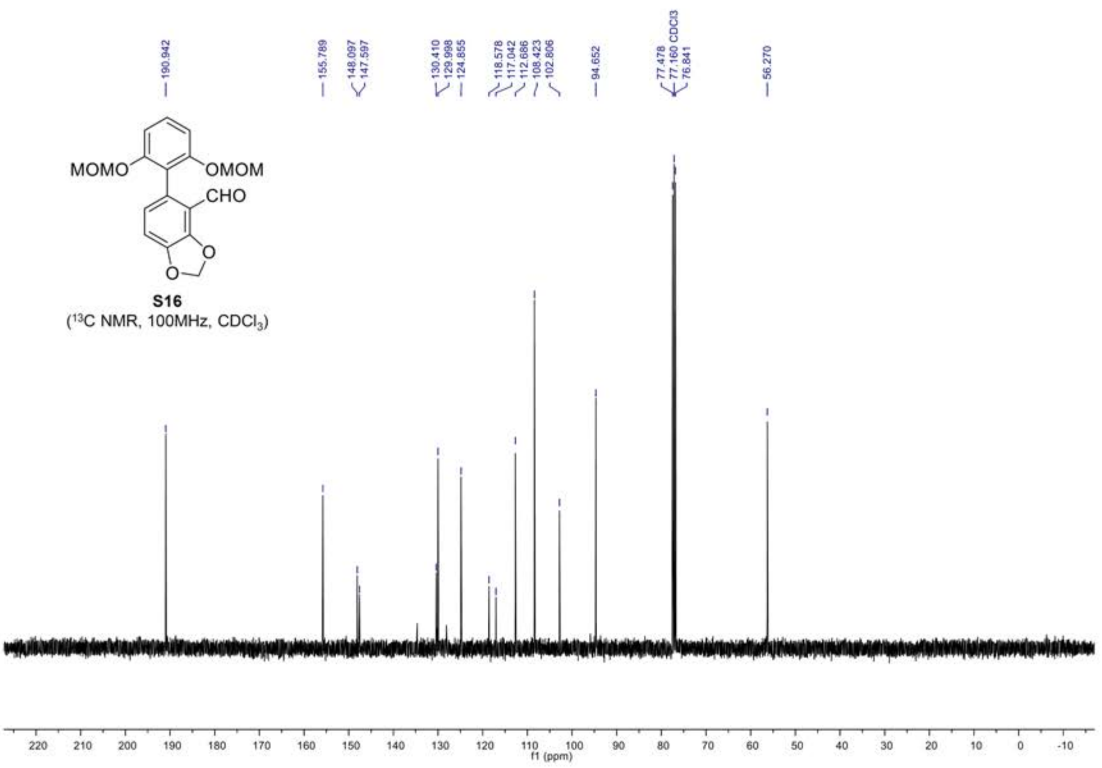

S16
(^{13}C NMR, 100MHz, $CDCl_3$)

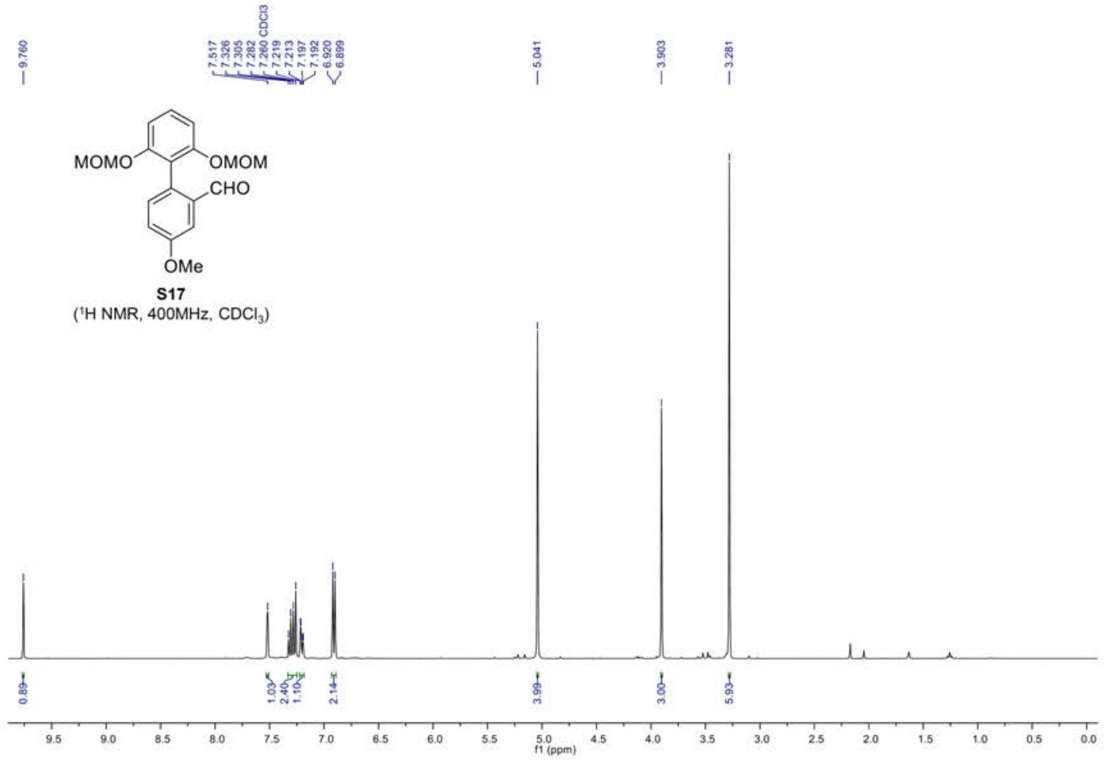
9.760
7.517
7.326
7.305
7.282
7.260 CDCl3
7.219
7.213
7.197
7.192
6.920
6.899
5.041
3.903
3.281
MOMO
OMOM
CHO
OMe
S17
(1H NMR, 400MHz, CDCl3)
0.89
1.03
2.40
1.10
2.14
3.99
3.00
5.93
f1 (ppm)

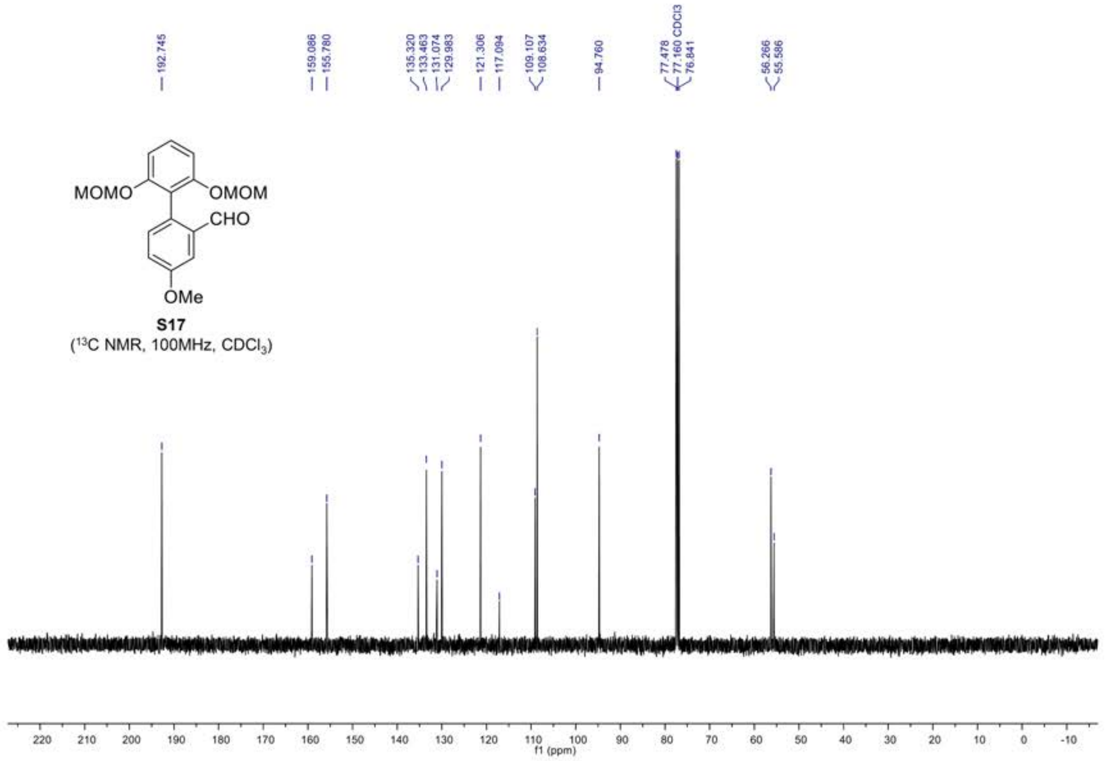
192.745
159.086
155.780
135.320
133.463
131.074
129.983
121.306
117.094
109.107
108.634
94.760
77.478
77.160 CDCl3
76.841
56.266
55.586
MOMO
OMOM
CHO
OMe
S17
(13C NMR, 100MHz, CDCl3)
f1 (ppm)

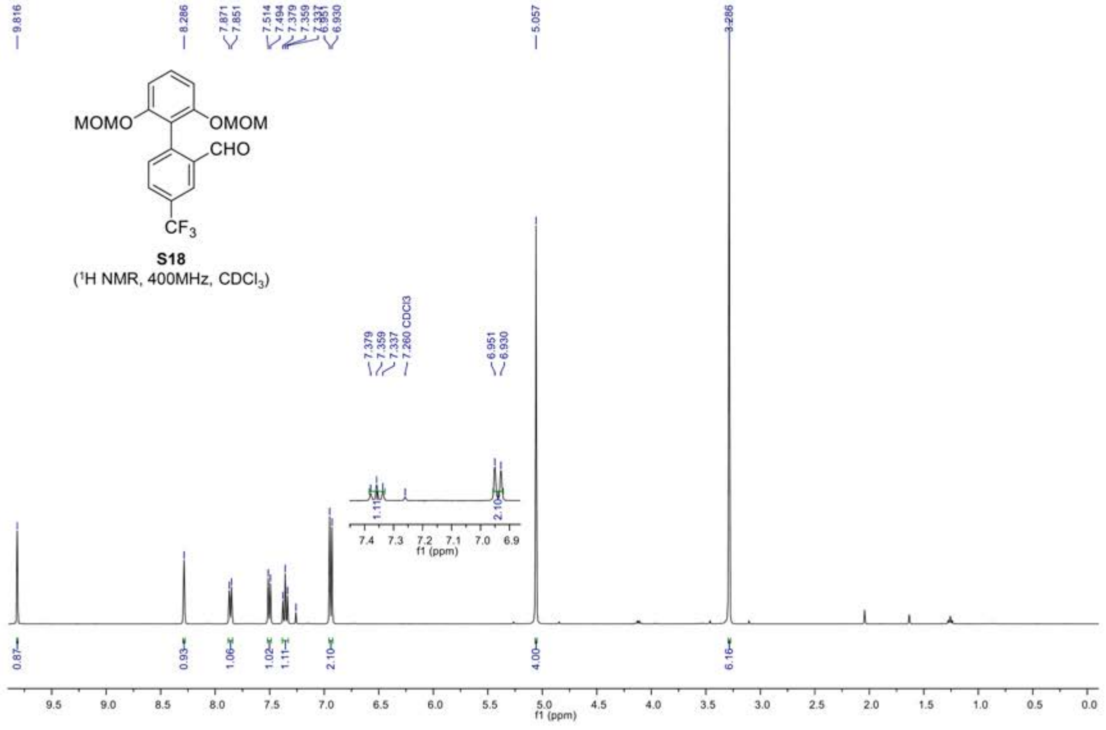

S18
(^{1}H NMR, 400MHz, CDCl$_3$)

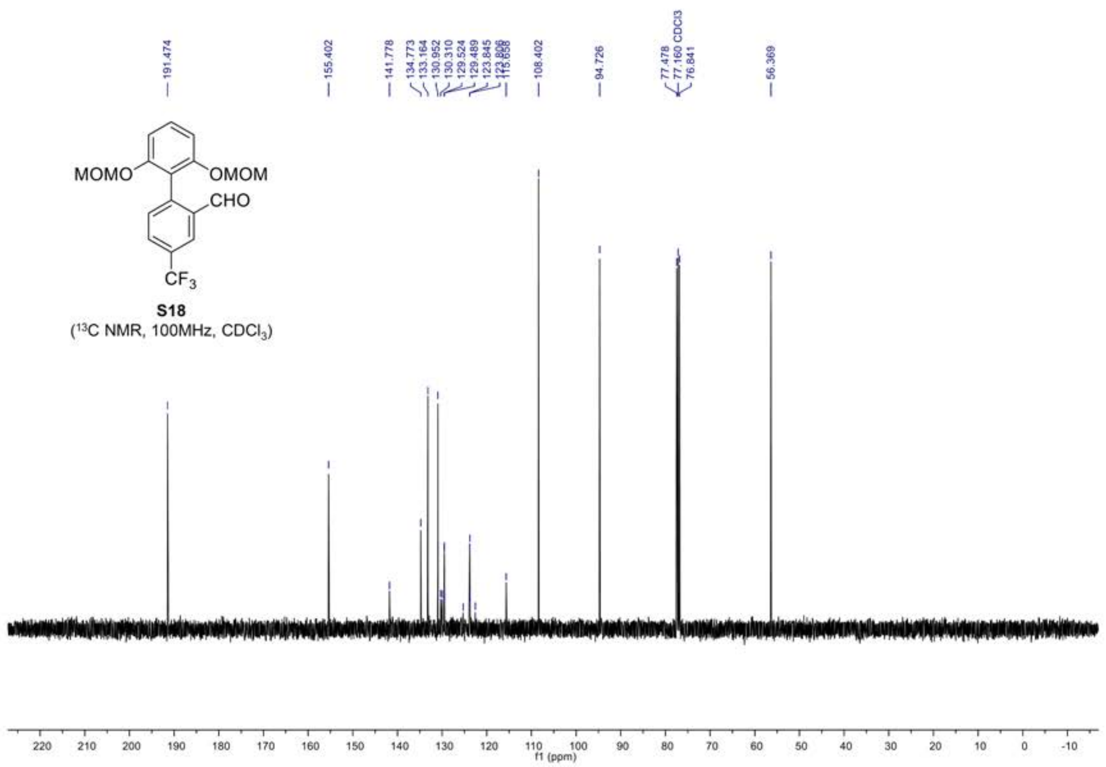

S18
(^{13}C NMR, 100MHz, CDCl$_3$)

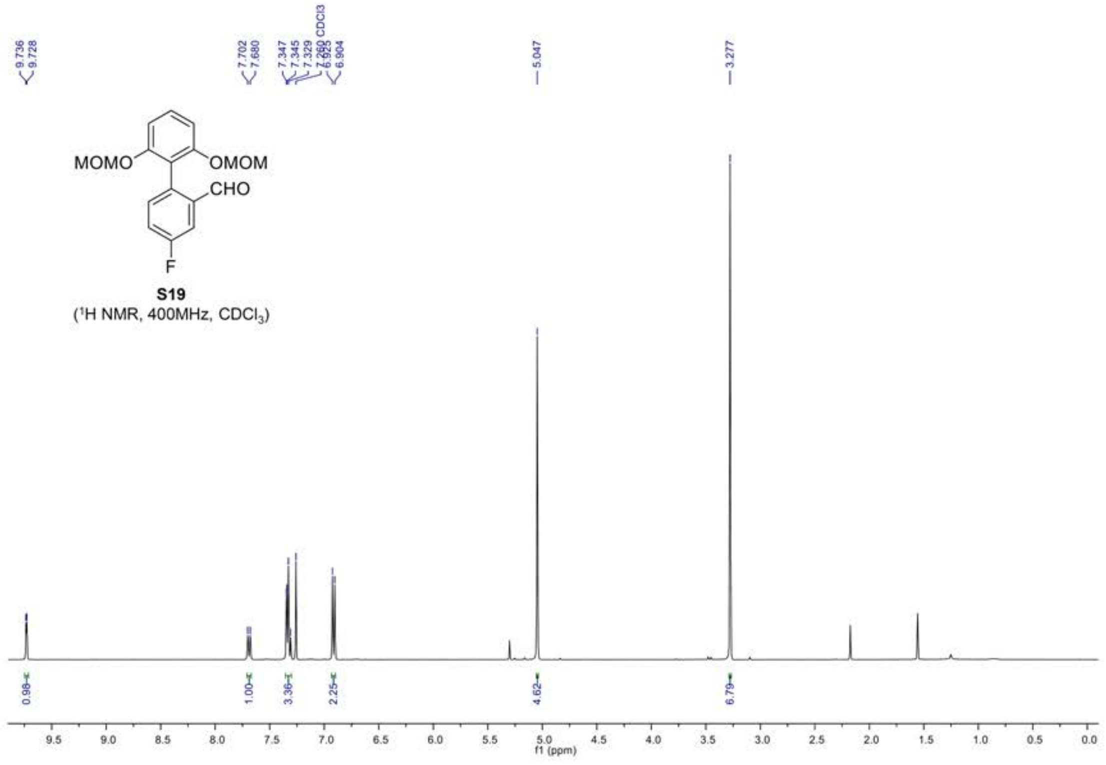
9.736
9.728
7.702
7.680
7.347
7.345
7.329
6.904
5.047
3.277
MOMO
OMOM
CHO
F
S19
(¹H NMR, 400MHz, CDCl₃)
0.98
1.00
3.36
2.25
4.62
6.79
f1 (ppm)

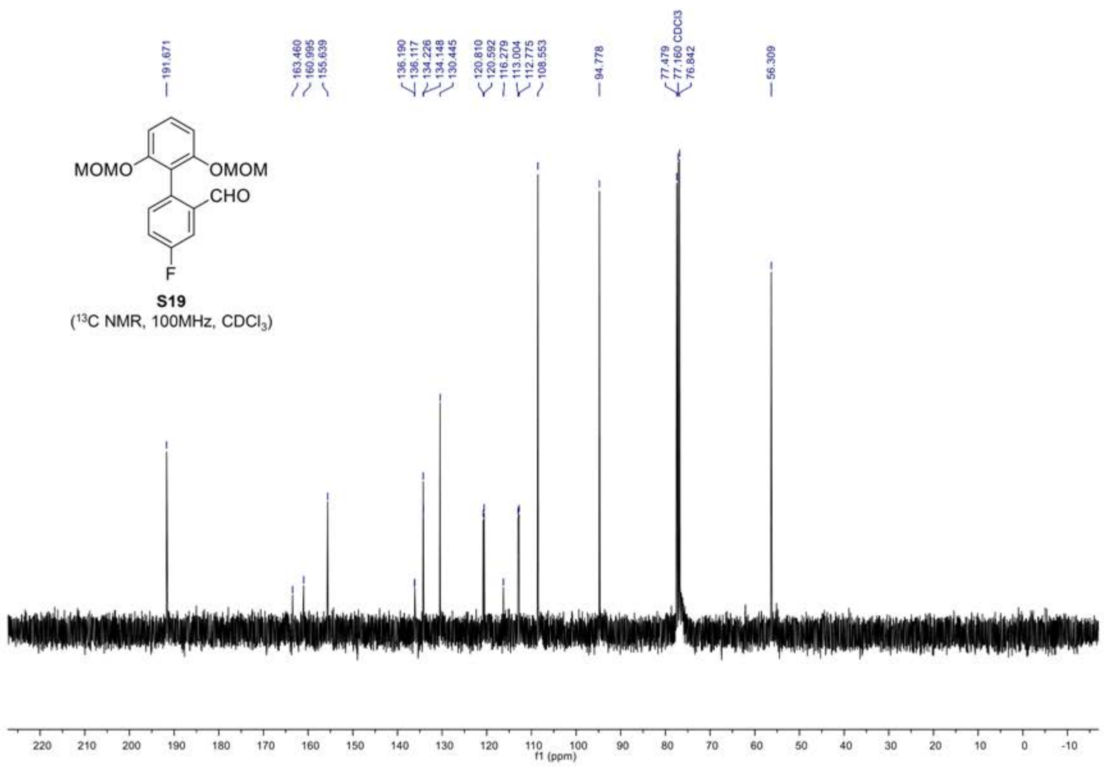
191.671
163.460
160.995
155.639
136.190
136.117
134.226
134.148
130.445
120.810
120.592
116.279
113.004
112.775
108.553
94.778
77.479
77.160 CDCl3
76.842
56.309
MOMO
OMOM
CHO
F
S19
(¹³C NMR, 100MHz, CDCl₃)
f1 (ppm)

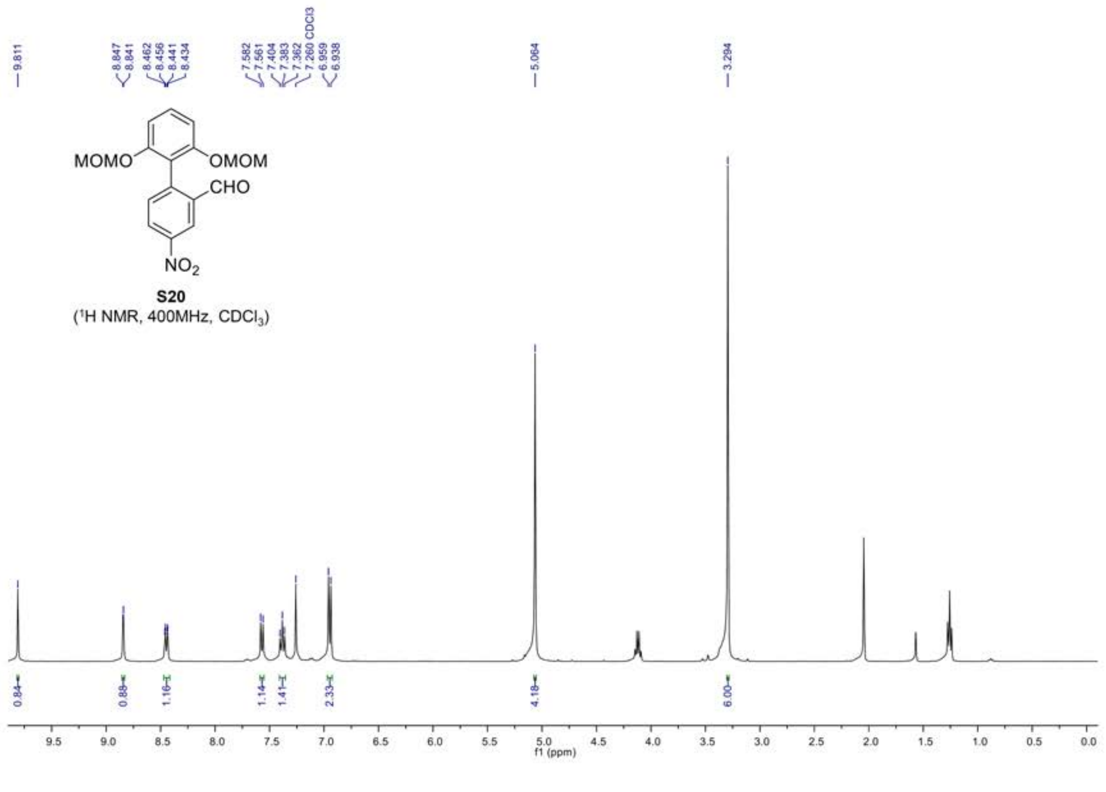
9.811
8.847
8.841
8.462
8.456
8.441
8.434
7.582
7.561
7.404
7.383
7.362
7.260 CDCl3
6.959
6.938
5.064
3.294
MOMO
OMOM
CHO
NO_2
S20
(^{1}H NMR, 400MHz, $CDCl_3$)
0.84
0.88
1.16
1.14
1.41
2.33
4.18
6.00
f1 (ppm)

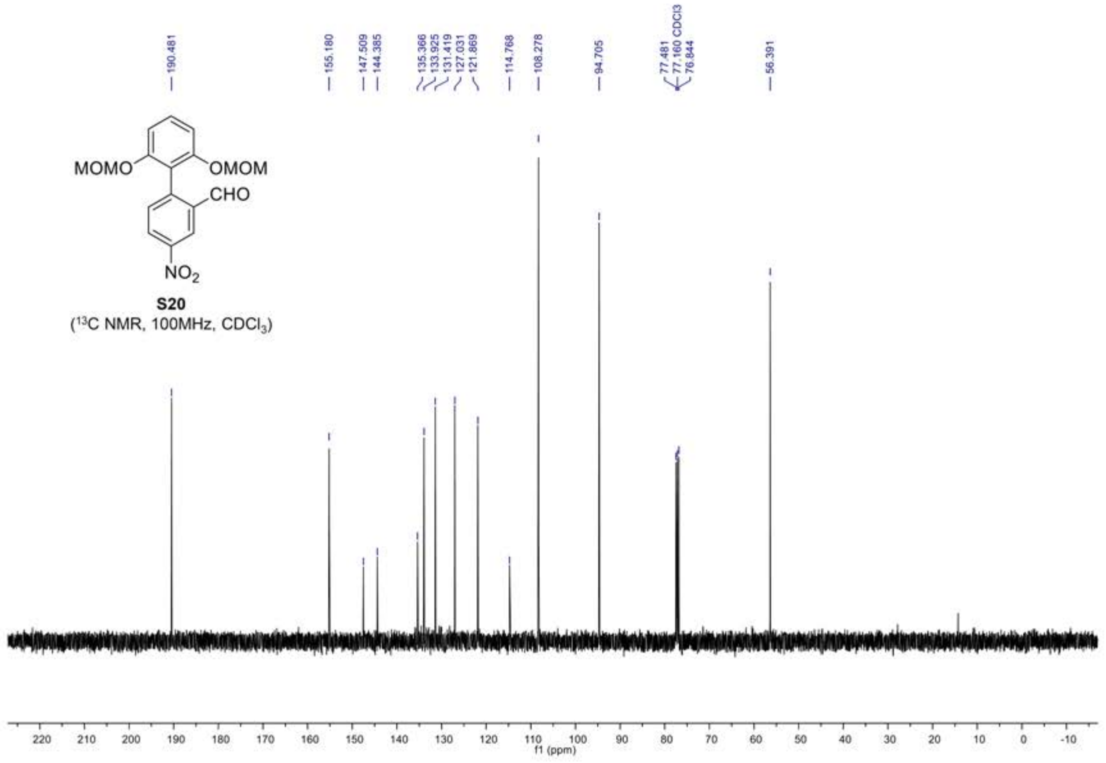
190.481
155.180
147.509
144.385
135.366
133.925
131.419
127.031
121.869
114.768
108.278
94.705
77.481
77.160 CDCl3
76.844
56.391
MOMO
OMOM
CHO
NO_2
S20
(^{13}C NMR, 100MHz, $CDCl_3$)
f1 (ppm)

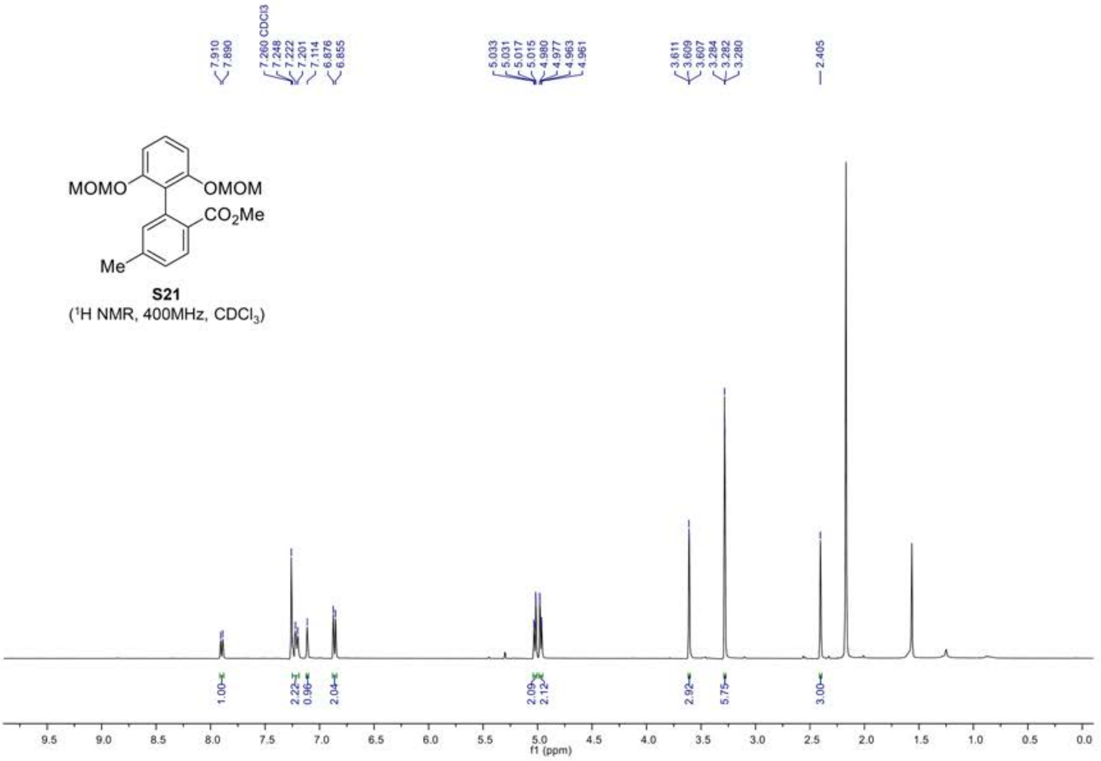
7.910
7.890
7.260 CDCl3
7.248
7.222
7.201
7.114
6.876
6.855
5.033
5.031
5.017
5.015
4.980
4.977
4.963
4.961
3.611
3.609
3.607
3.284
3.282
3.280
2.405
MOMO
OMOM
CO2Me
Me
S21
(1H NMR, 400MHz, CDCl3)
1.00
2.22
0.96
2.04
2.09
2.12
2.92
5.75
3.00
f1 (ppm)

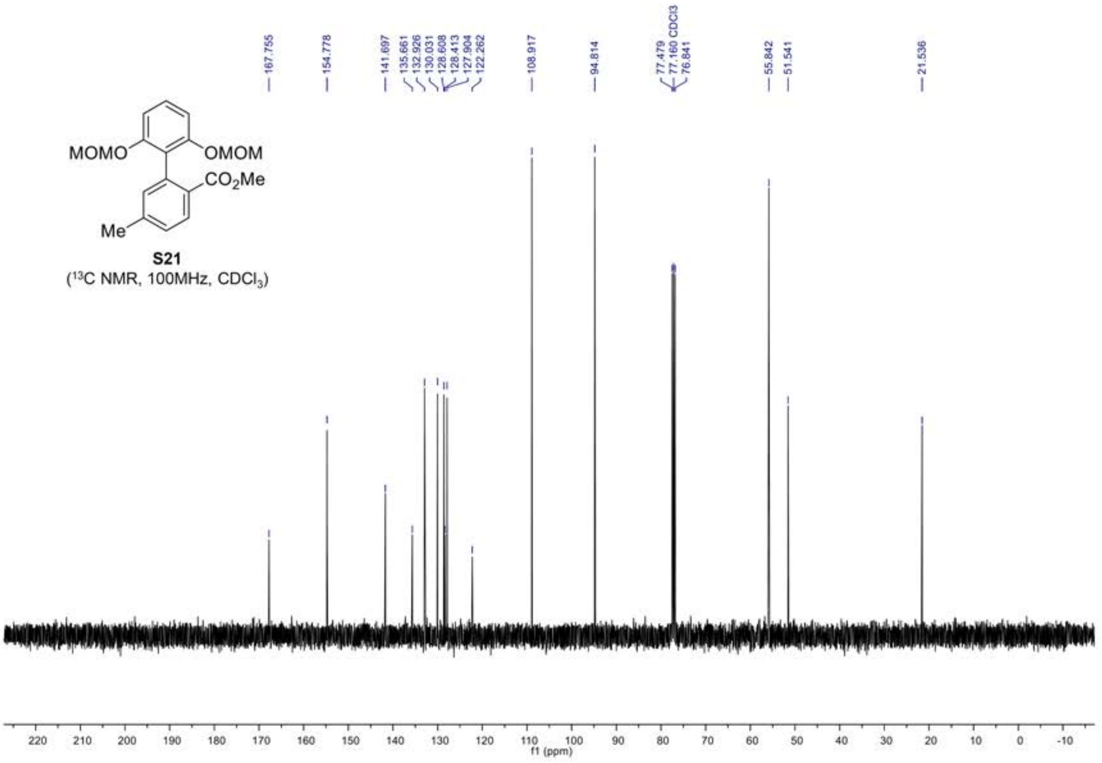
167.755
154.778
141.697
135.661
132.926
130.031
128.608
128.413
127.904
122.262
108.917
94.814
77.479
77.160 CDCl3
76.841
55.842
51.541
21.536
MOMO
OMOM
CO2Me
Me
S21
(13C NMR, 100MHz, CDCl3)
f1 (ppm)

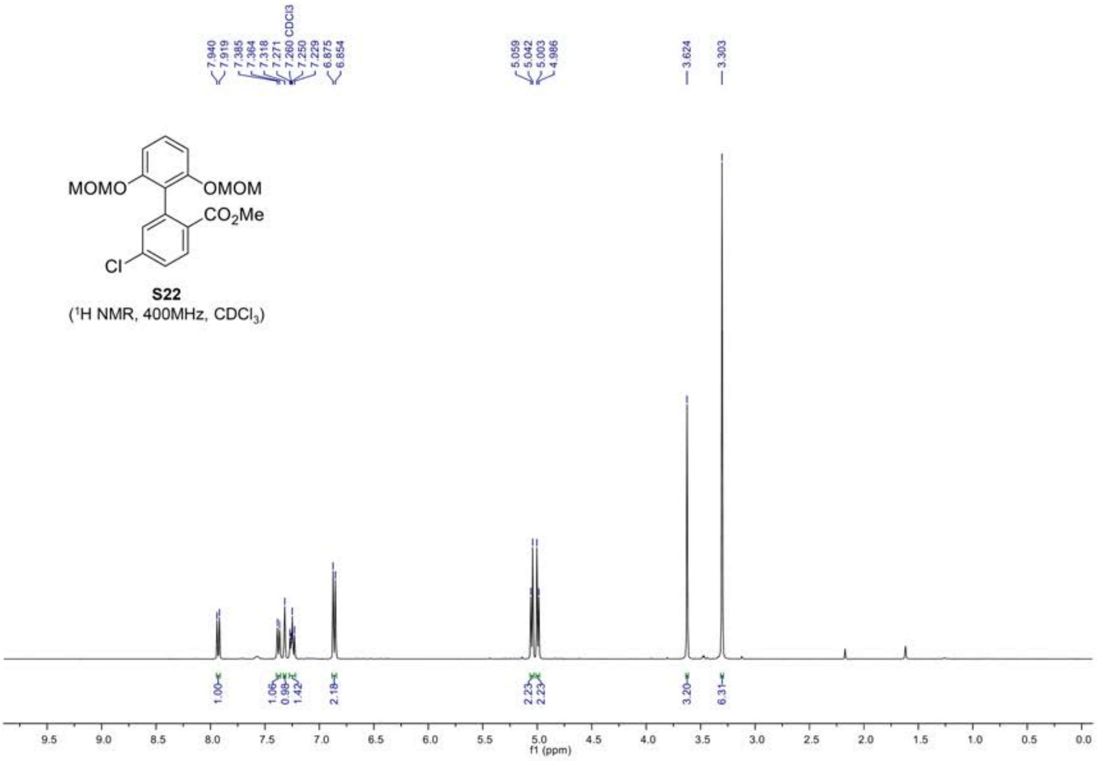

S22
(^1H NMR, 400MHz, $CDCl_3$)

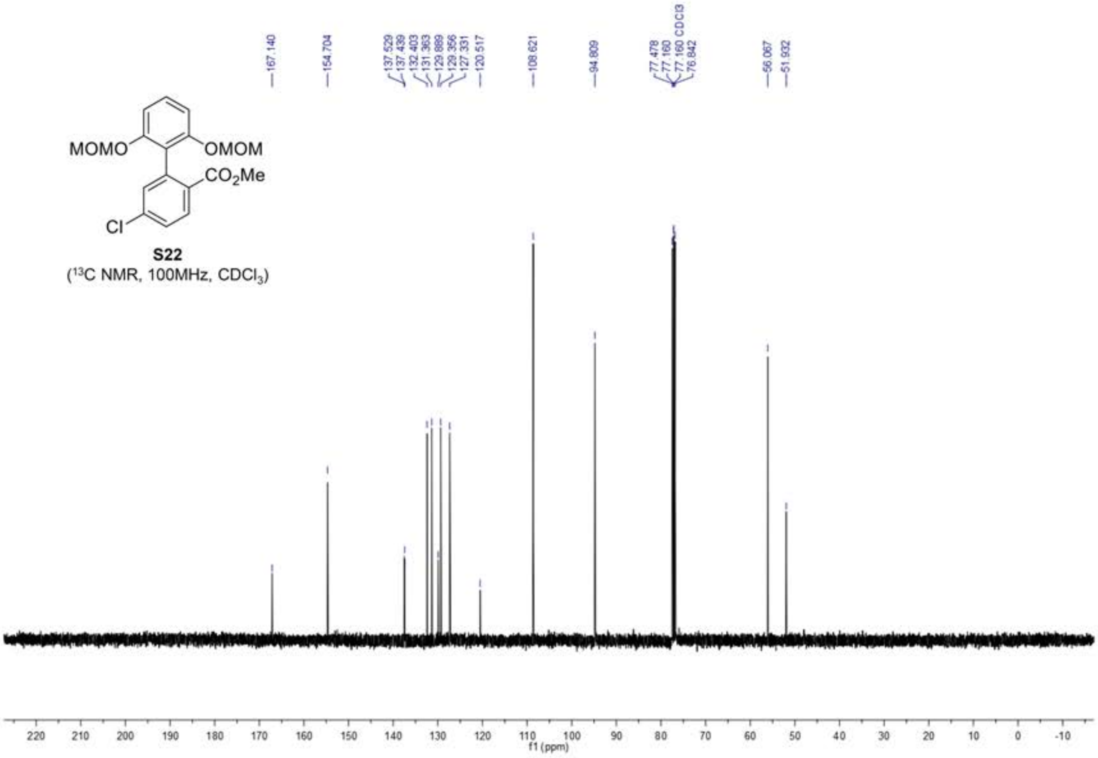

S22
(^{13}C NMR, 100MHz, $CDCl_3$)

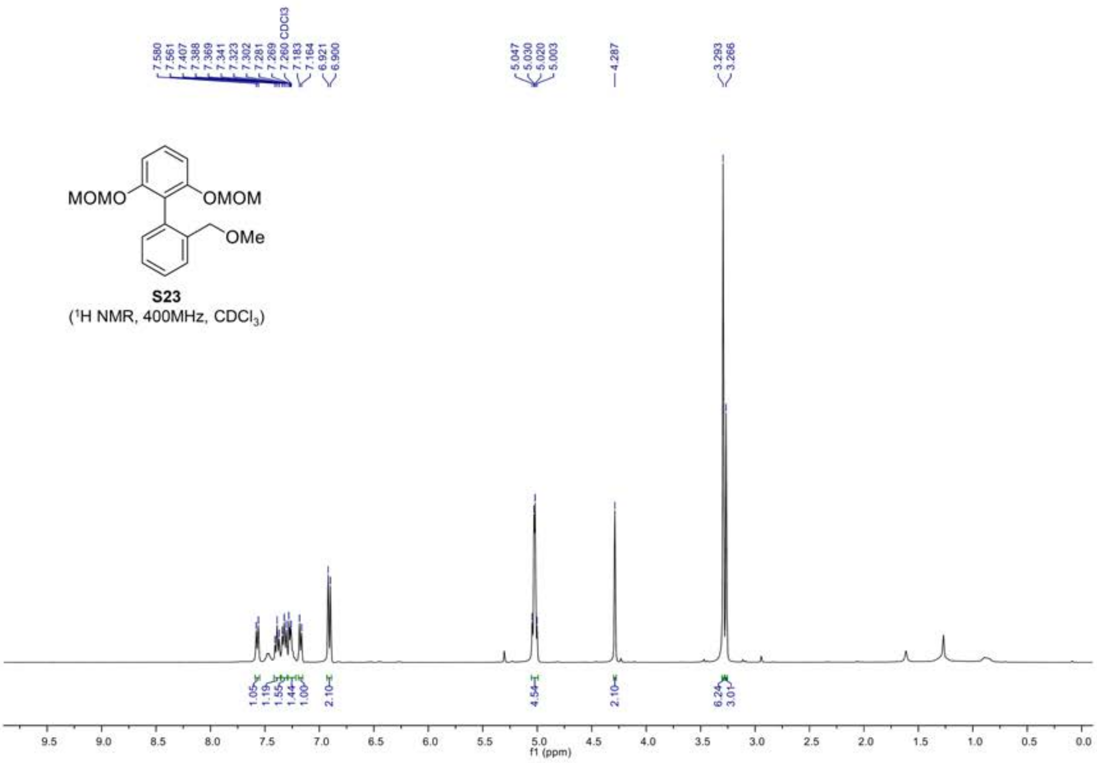

S23
(^{1}H NMR, 400MHz, $CDCl_3$)

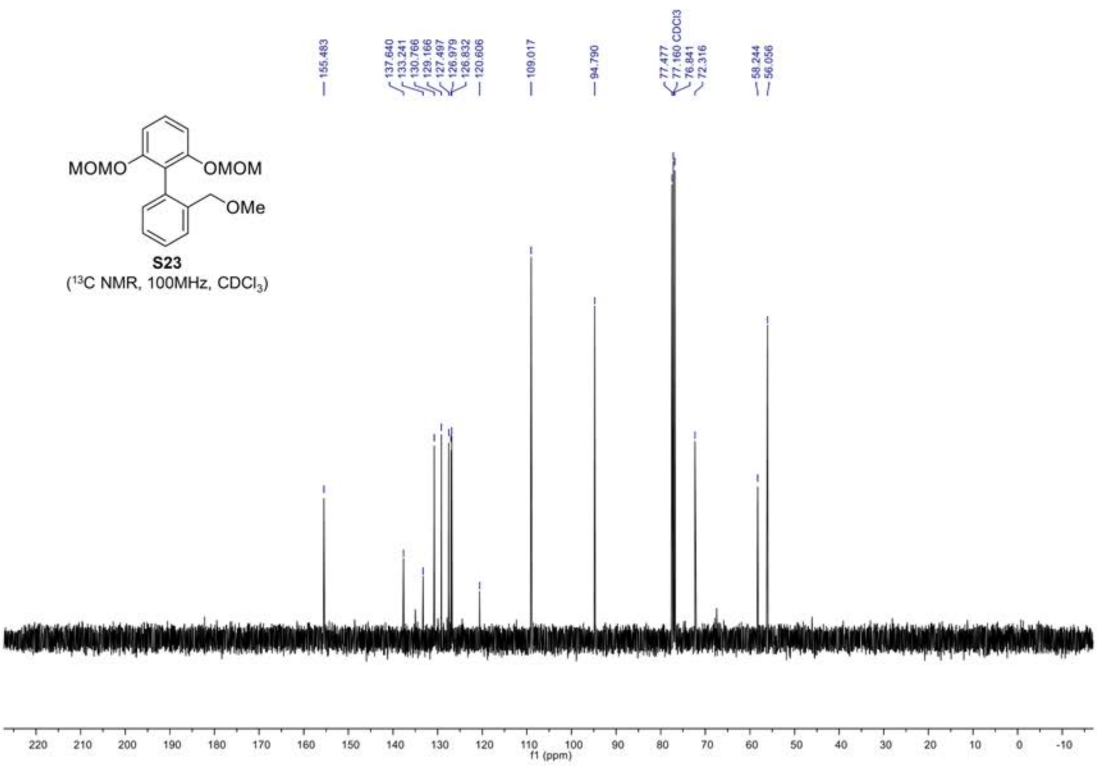

S23
(^{13}C NMR, 100MHz, $CDCl_3$)

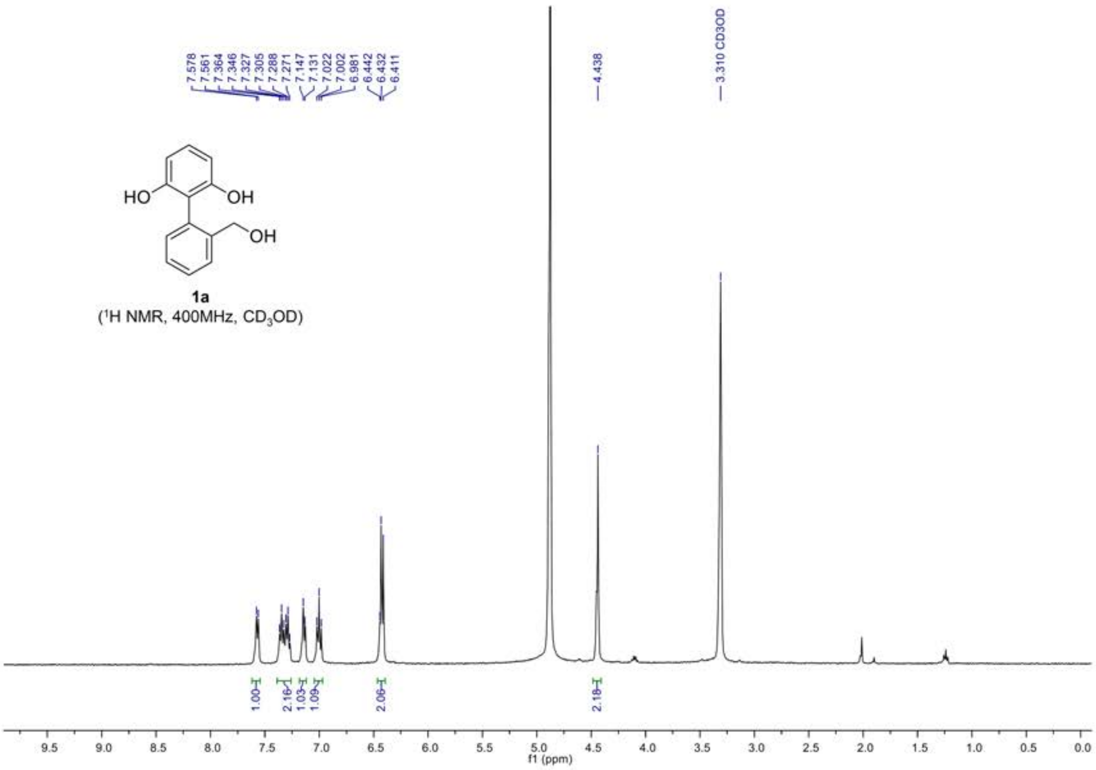
7.578
7.561
7.364
7.346
7.327
7.305
7.288
7.271
7.147
7.131
7.022
7.002
6.981
6.442
6.432
6.411
4.438
3.310 CD3OD
HO
OH
OH
1a
(1H NMR, 400MHz, CD3OD)
1.00
2.16
1.03
1.09
2.06
2.18
f1 (ppm)

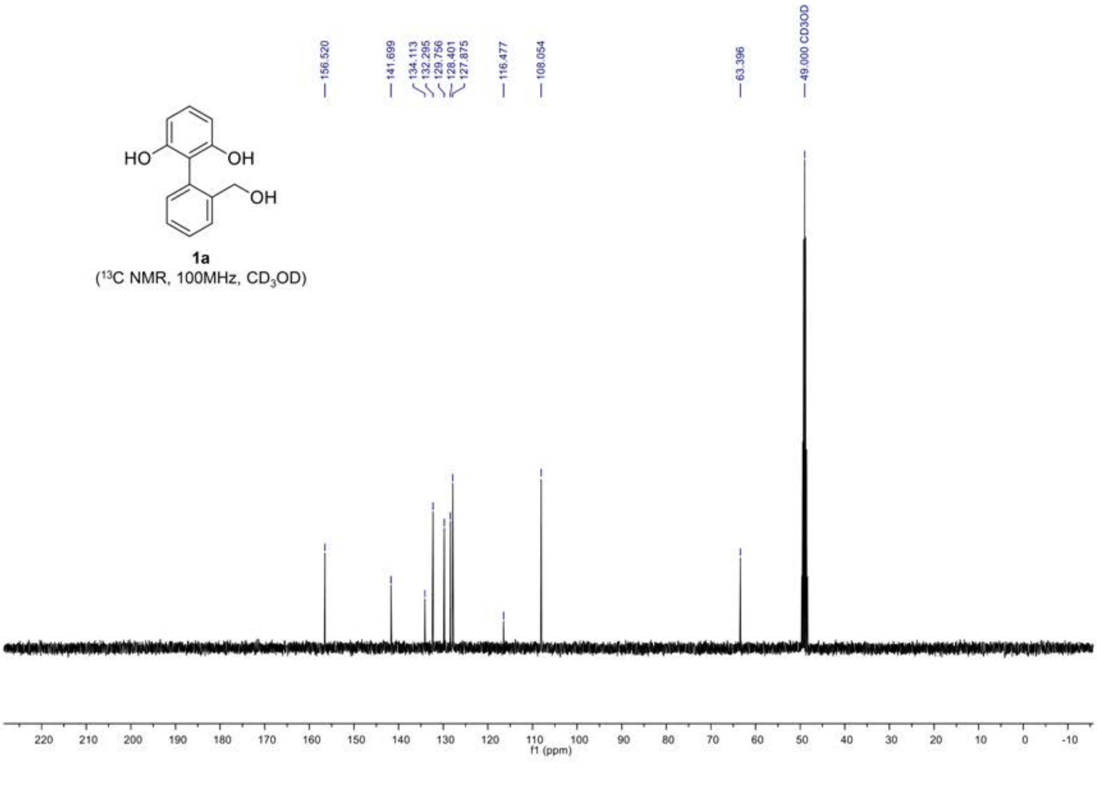
156.520
141.699
134.113
132.295
129.756
128.401
127.875
116.477
108.054
63.396
49.000 CD3OD
HO
OH
OH
1a
(13C NMR, 100MHz, CD3OD)
f1 (ppm)

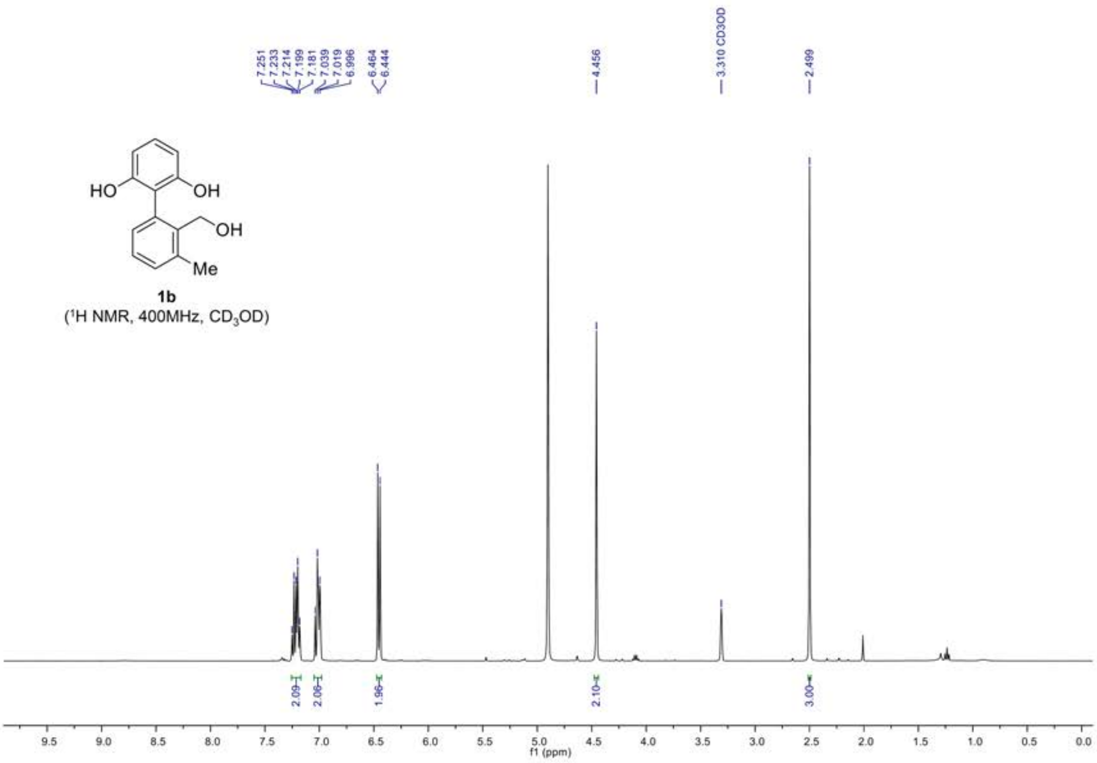

1b
(^{1}H NMR, 400MHz, CD_3OD)

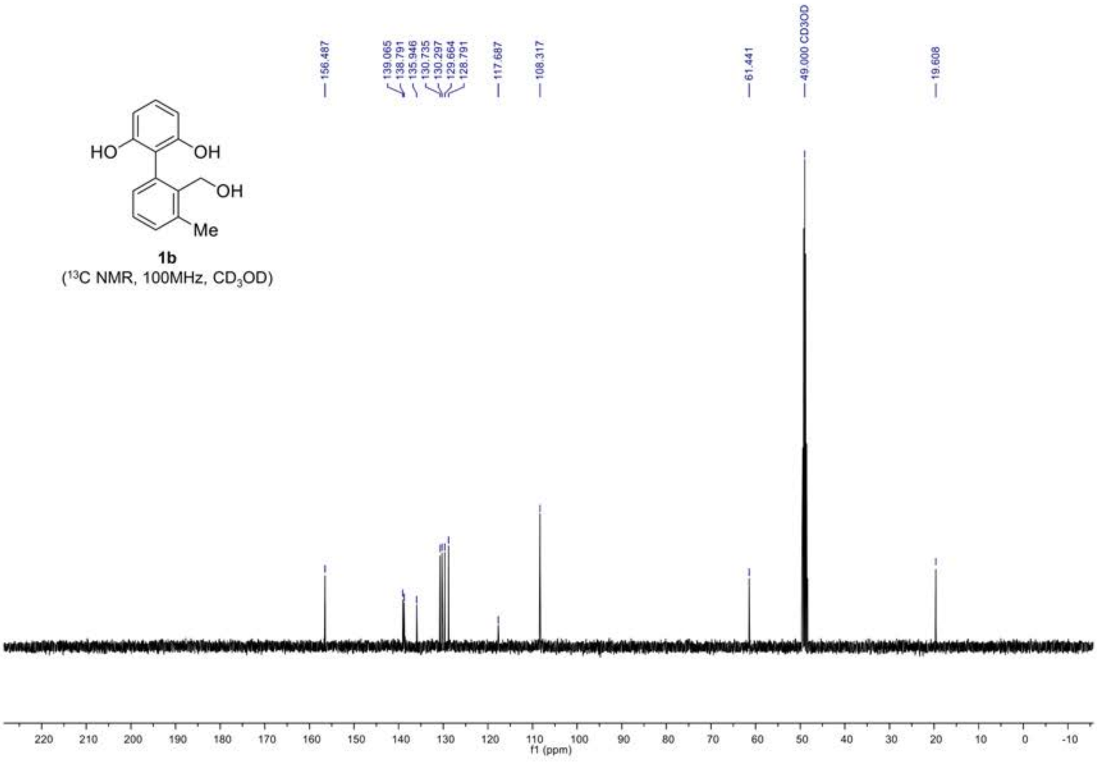

1b
(^{13}C NMR, 100MHz, CD_3OD)

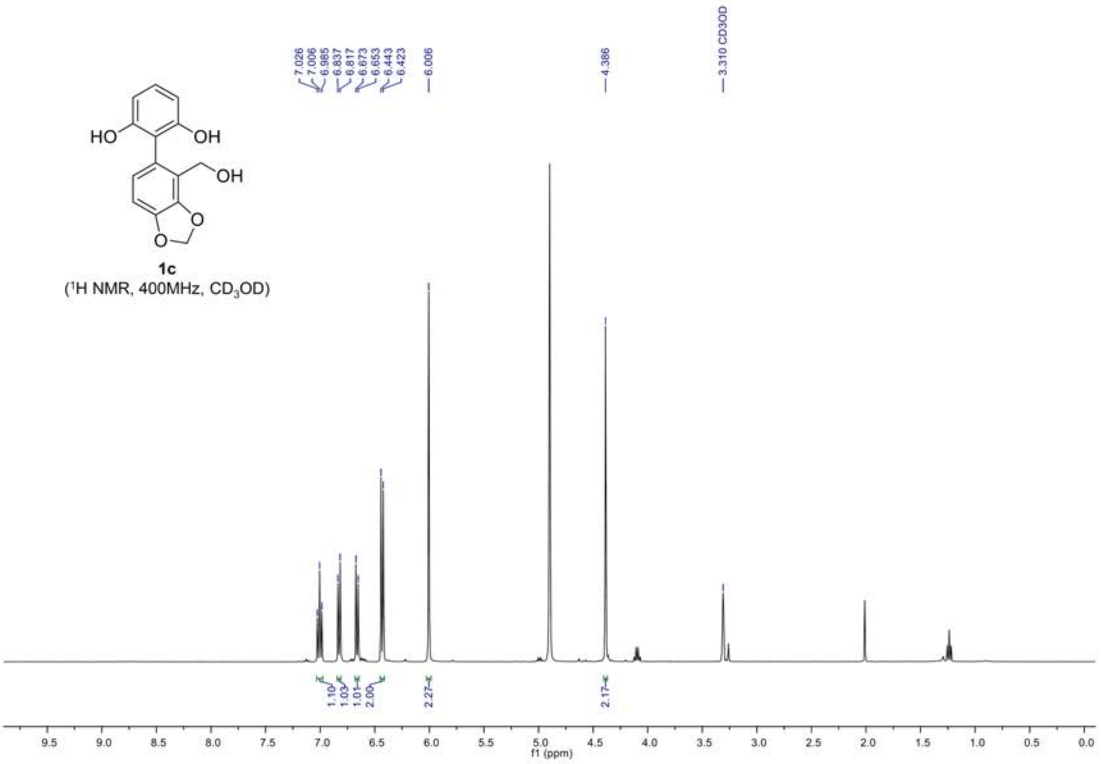

1c
(^{1}H NMR, 400MHz, CD_3OD)

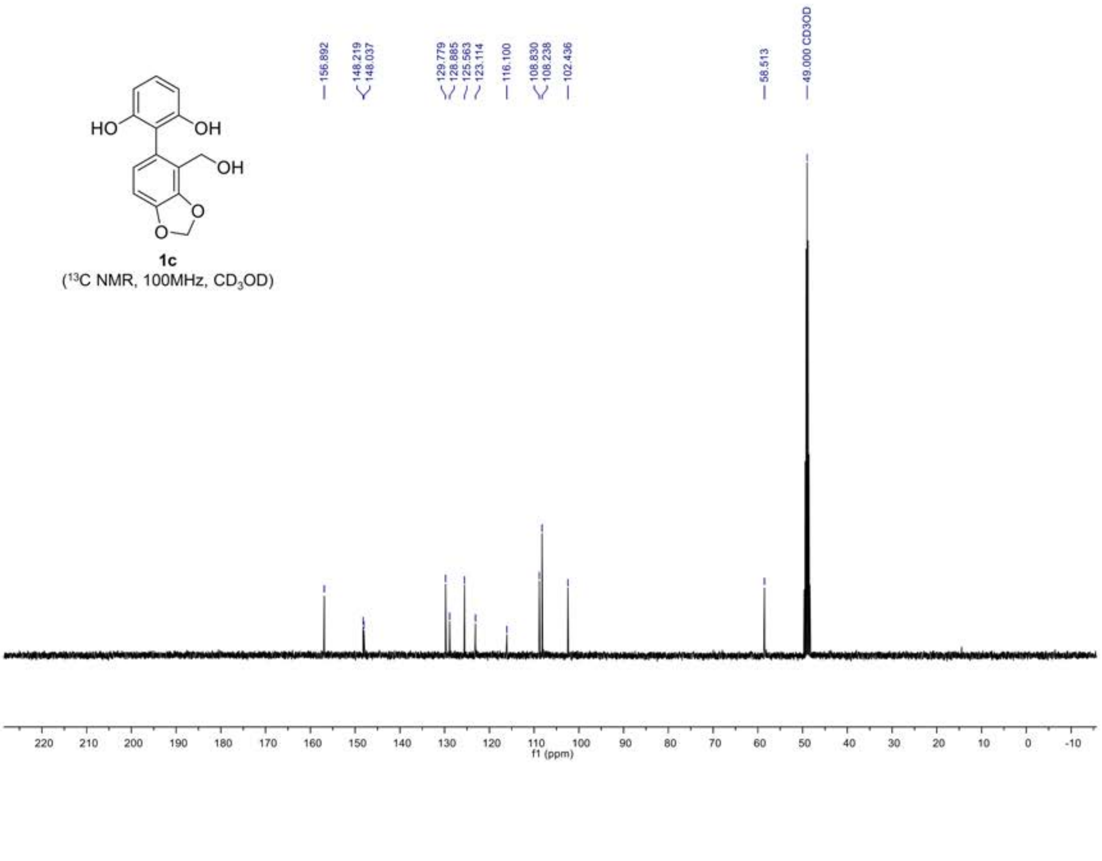

1c
(^{13}C NMR, 100MHz, CD_3OD)

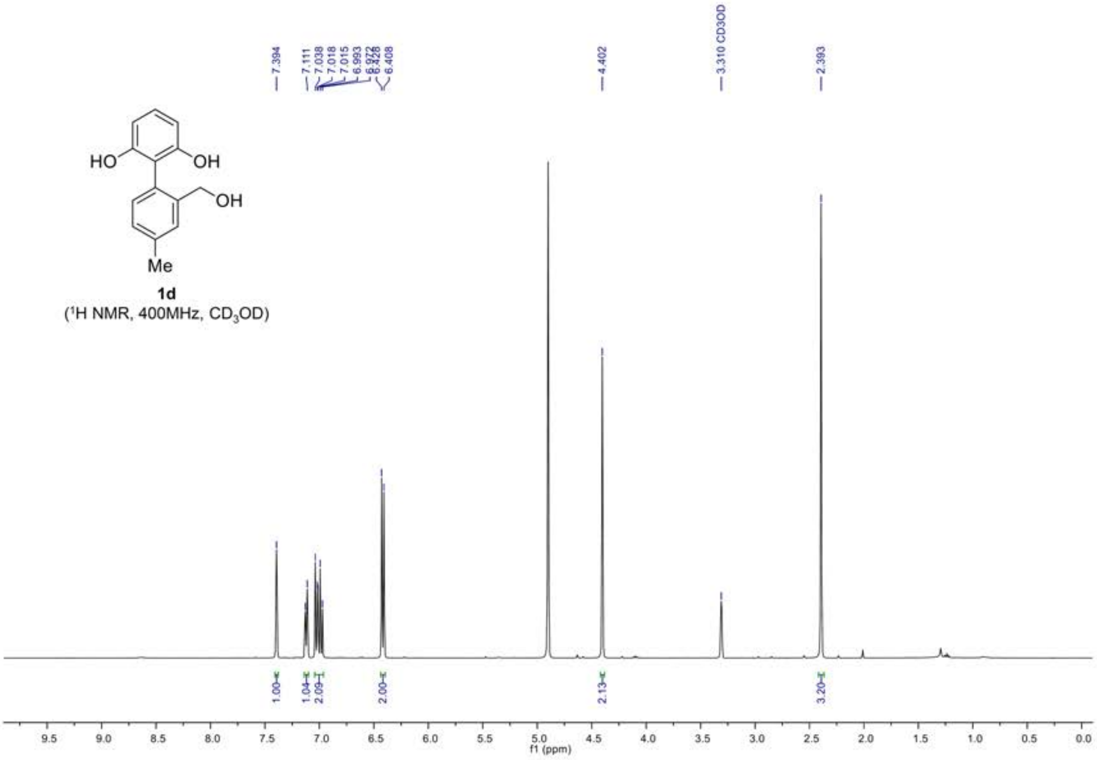

1d
(^{1}H NMR, 400MHz, CD_3OD)

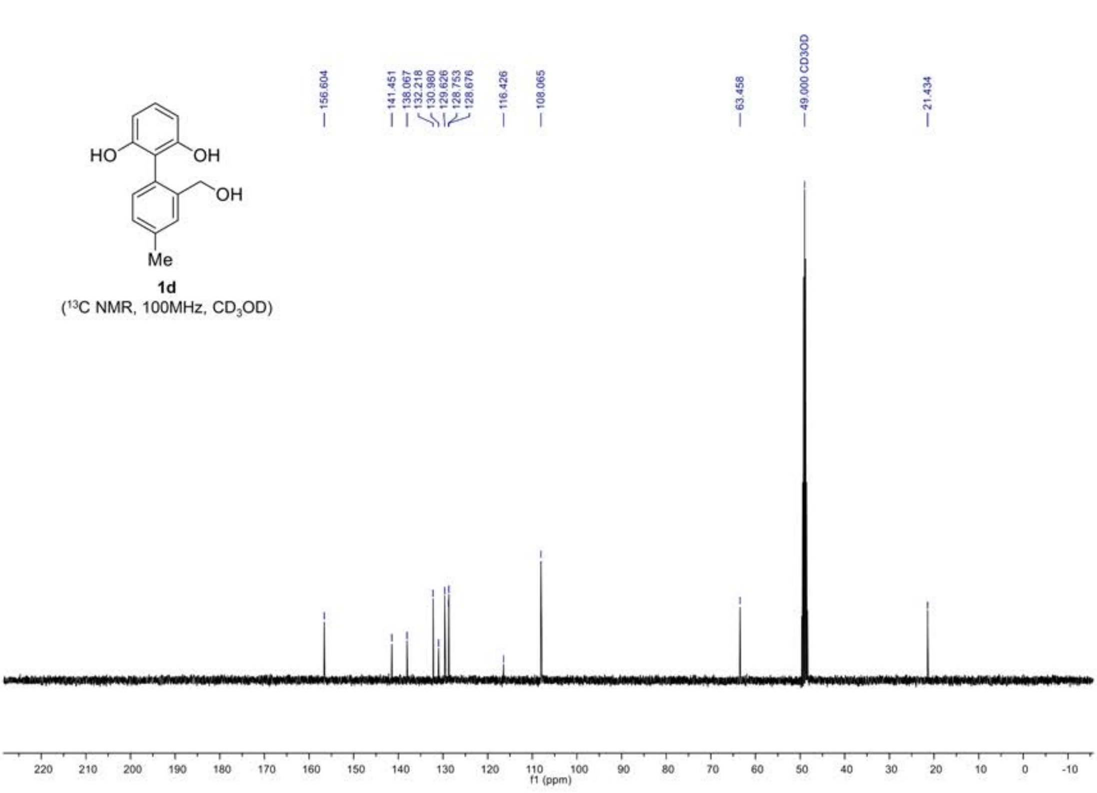

1d
(^{13}C NMR, 100MHz, CD_3OD)

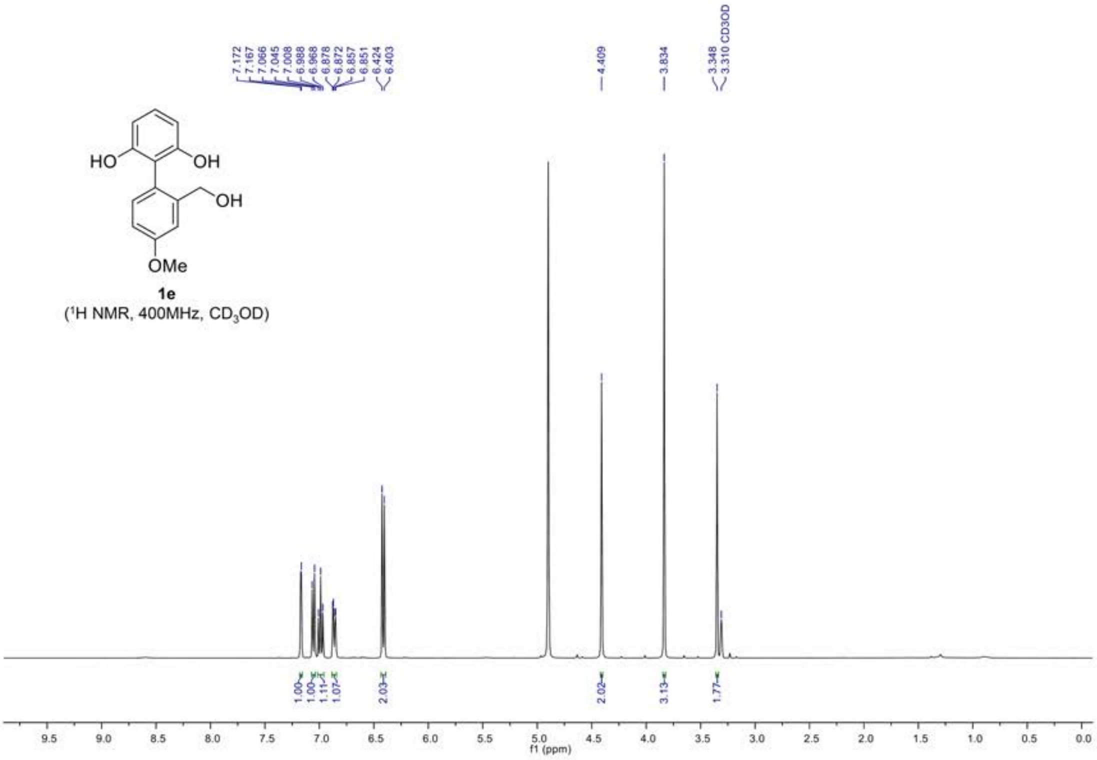

1e
(^{1}H NMR, 400MHz, CD_3OD)

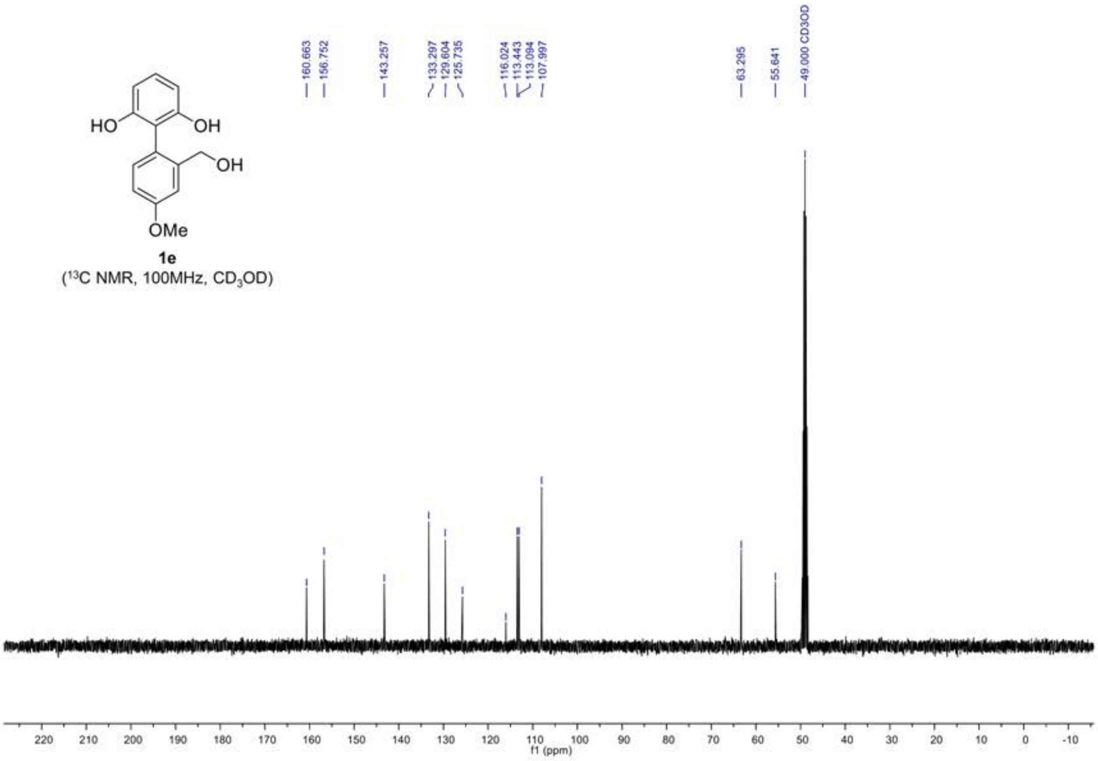

1e
(^{13}C NMR, 100MHz, CD_3OD)

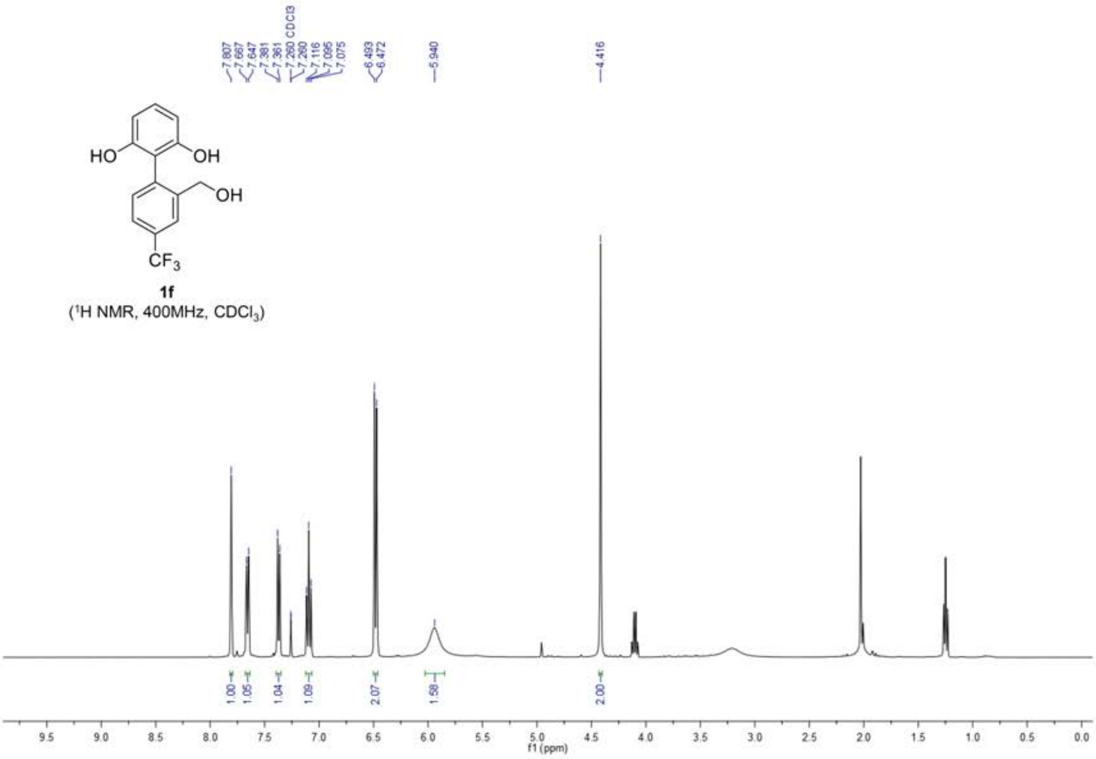

1f
(^{1}H NMR, 400MHz, $CDCl_3$)

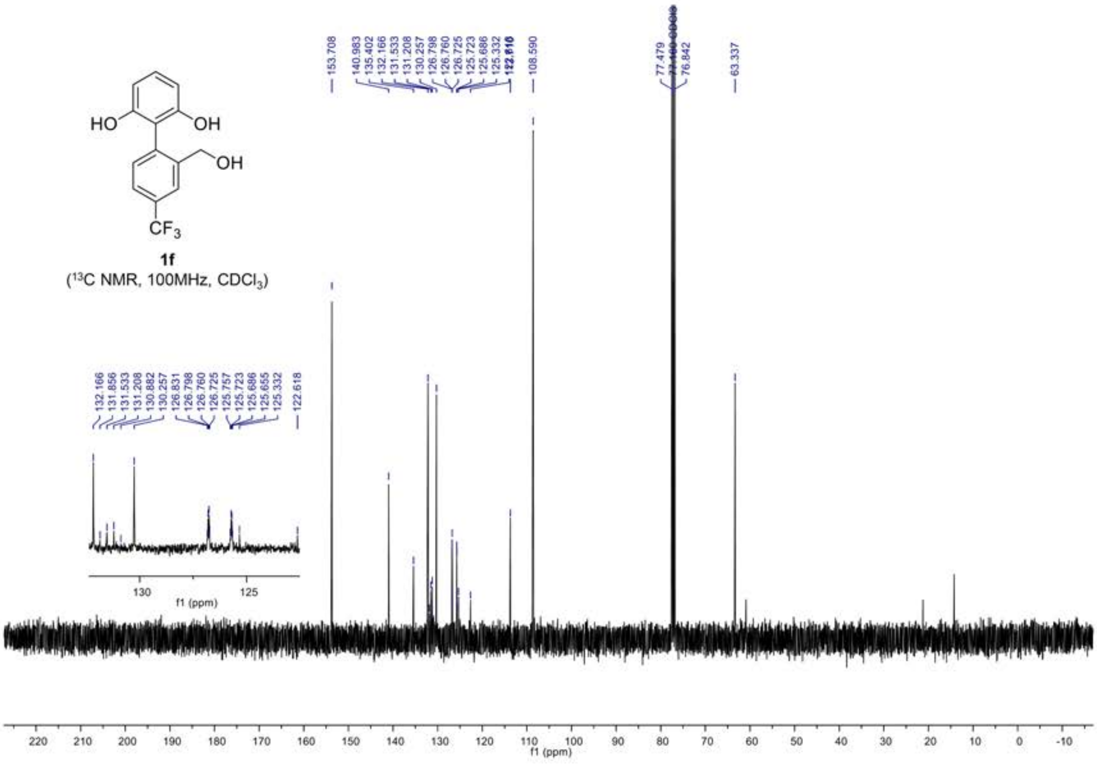

1f
(^{13}C NMR, 100MHz, $CDCl_3$)

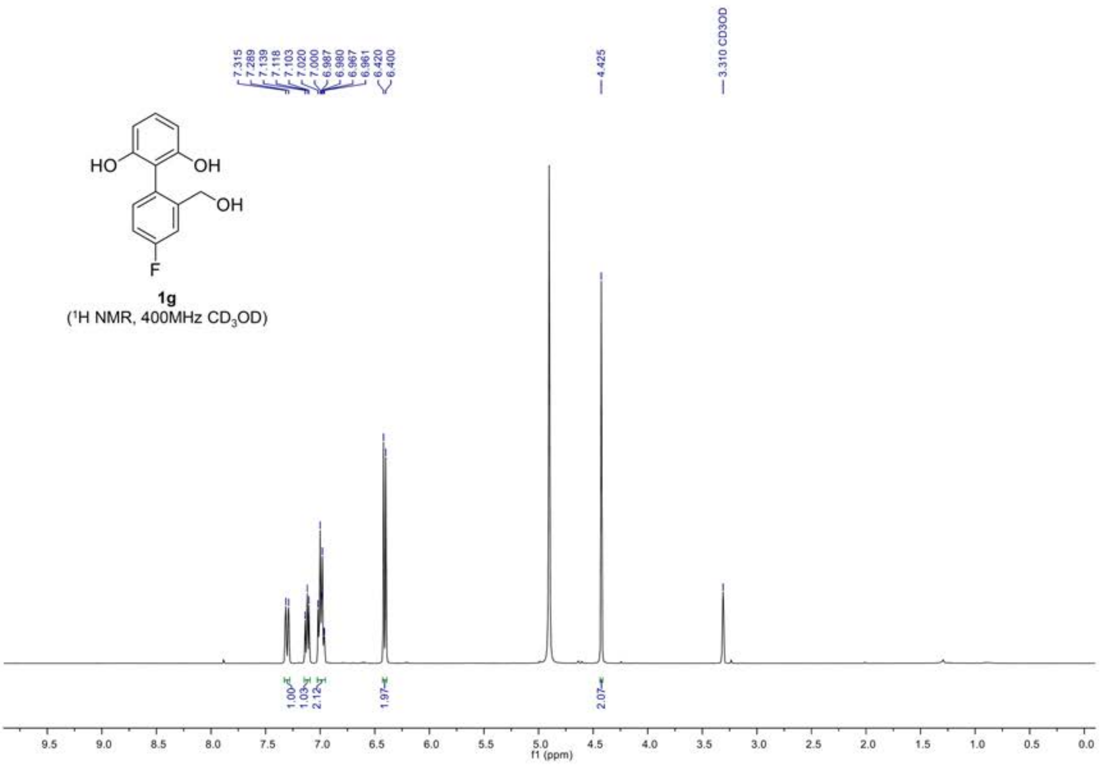

1g
(^{1}H NMR, 400MHz CD_3OD)

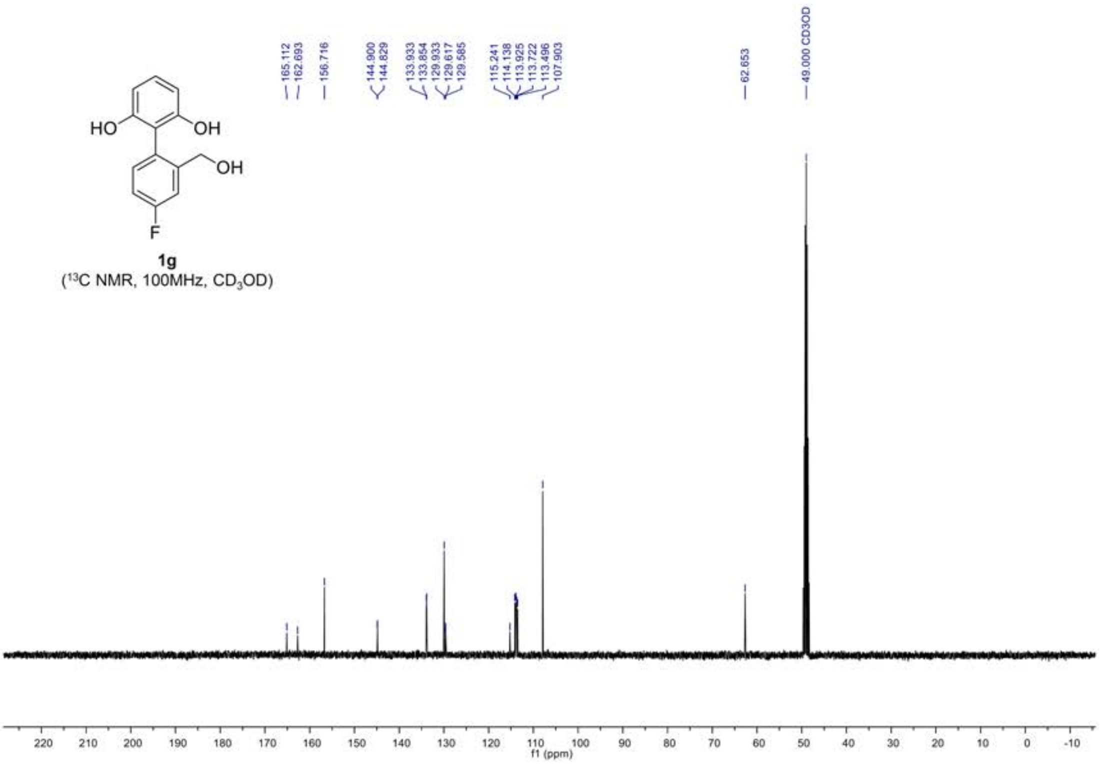

1g
(^{13}C NMR, 100MHz, CD_3OD)

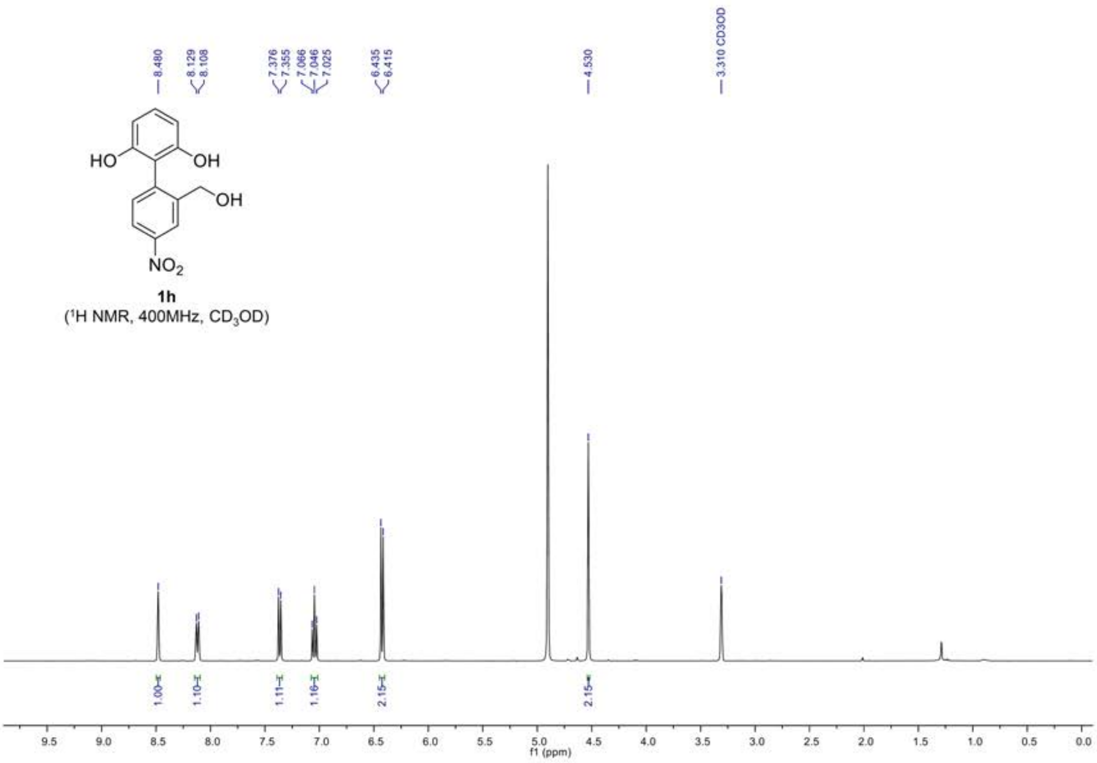
8.480
8.129
8.108
7.376
7.355
7.066
7.046
7.025
6.435
6.415
4.530
3.310 CD3OD
HO
OH
OH
NO2
1h
(1H NMR, 400MHz, CD3OD)
1.00
1.10
1.11
1.16
2.15
2.15
9.5 9.0 8.5 8.0 7.5 7.0 6.5 6.0 5.5 5.0 4.5 4.0 3.5 3.0 2.5 2.0 1.5 1.0 0.5 0.0
f1 (ppm)

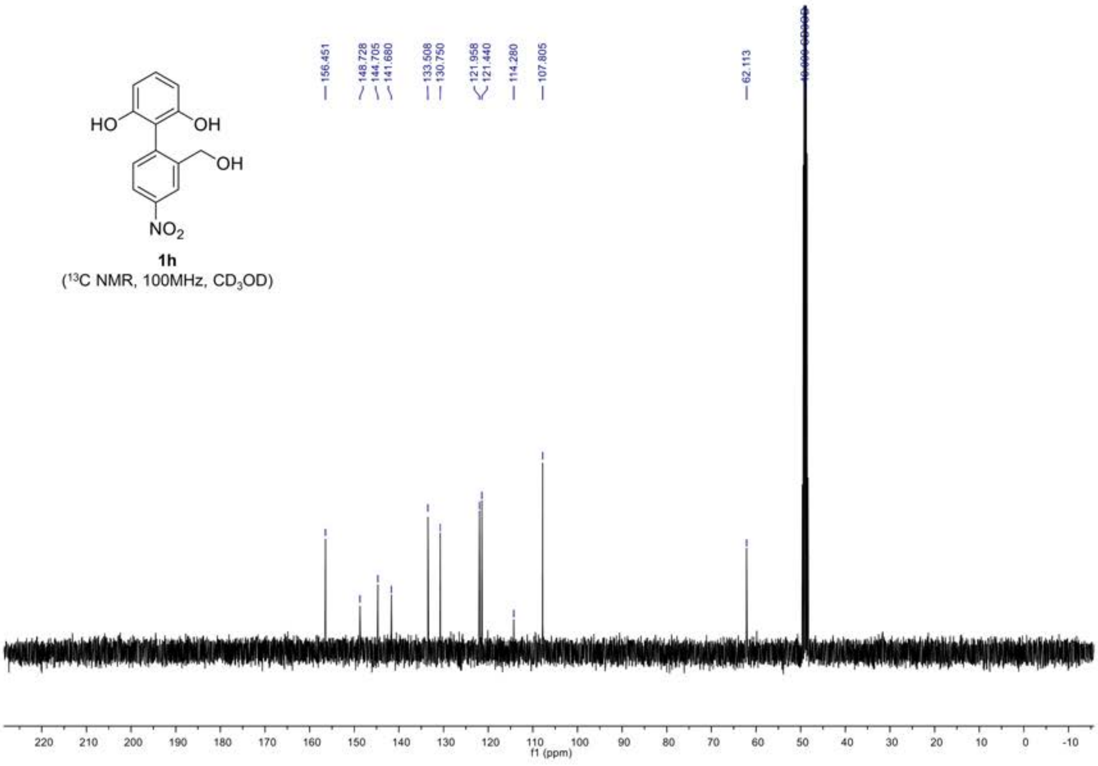
156.451
148.728
144.705
141.680
133.508
130.750
121.958
121.440
114.280
107.805
62.113
HO
OH
OH
NO2
1h
(13C NMR, 100MHz, CD3OD)
220 210 200 190 180 170 160 150 140 130 120 110 100 90 80 70 60 50 40 30 20 10 0 -10
f1 (ppm)

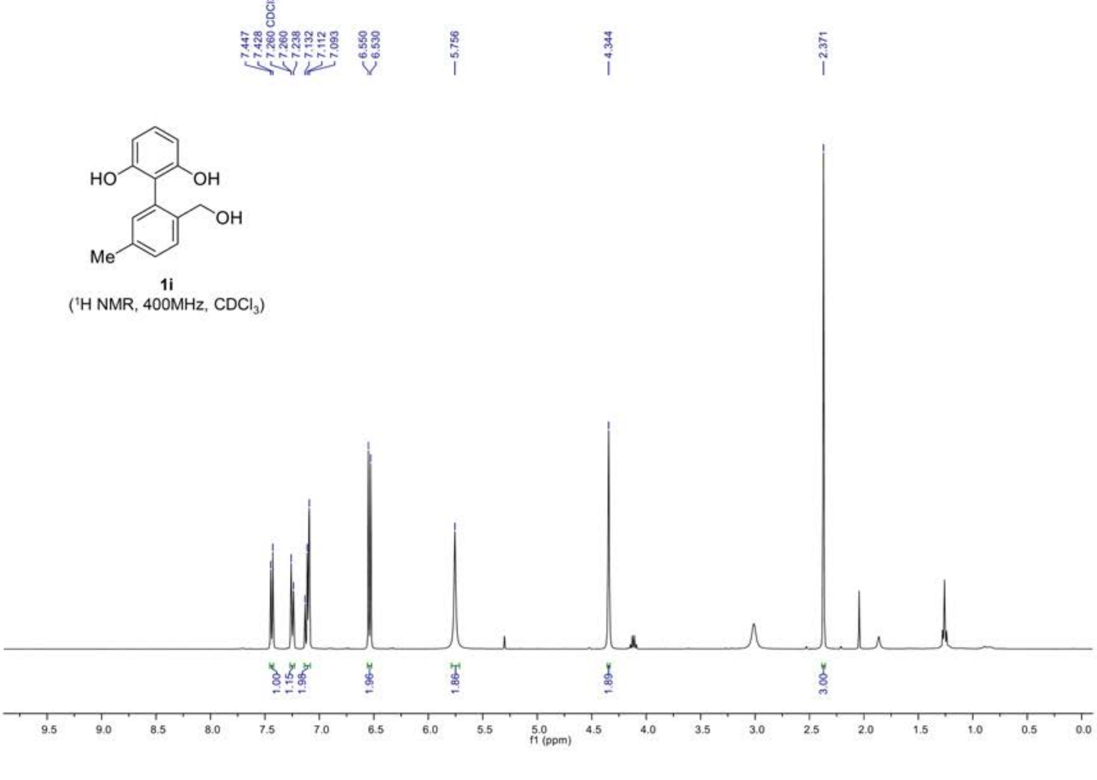

1i
(^{1}H NMR, 400MHz, $CDCl_3$)

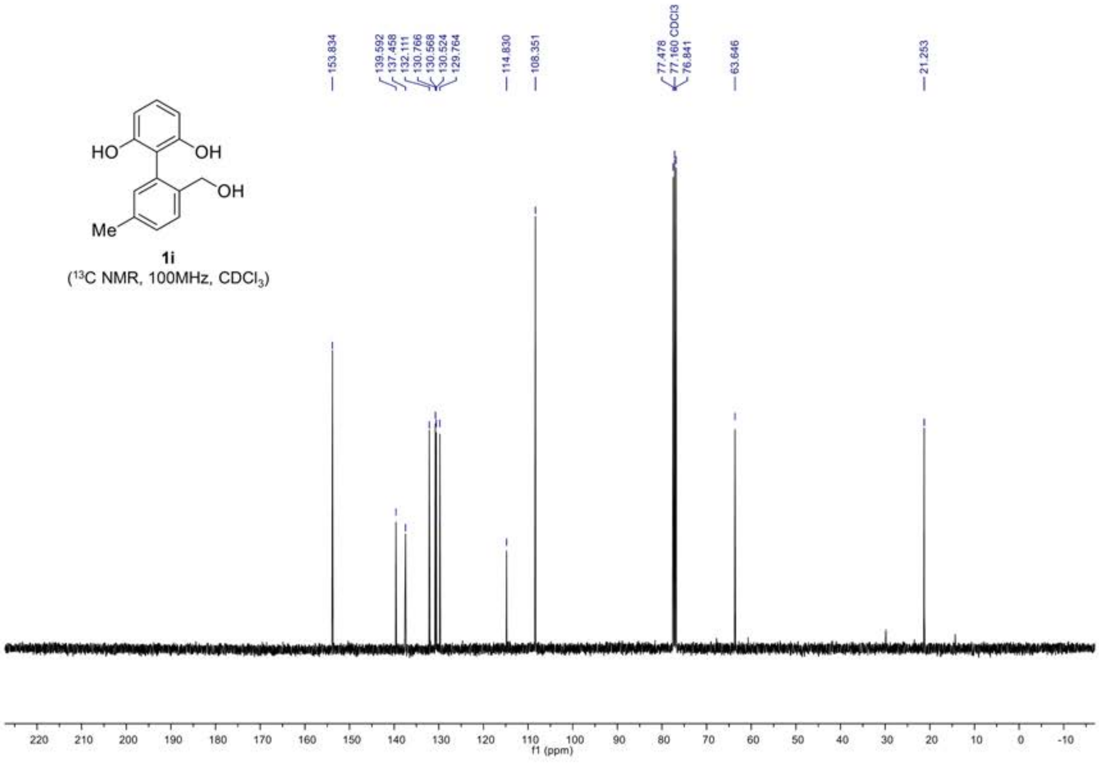

1i
(^{13}C NMR, 100MHz, $CDCl_3$)

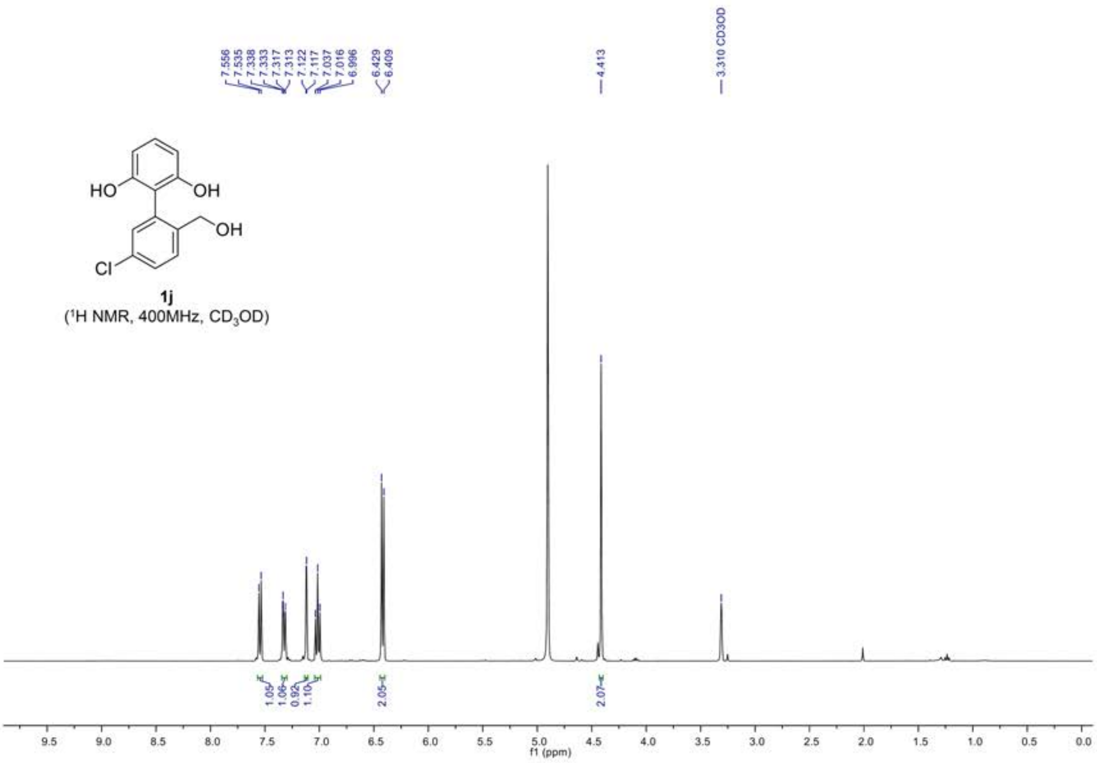

1j
(^{1}H NMR, 400MHz, CD_3OD)

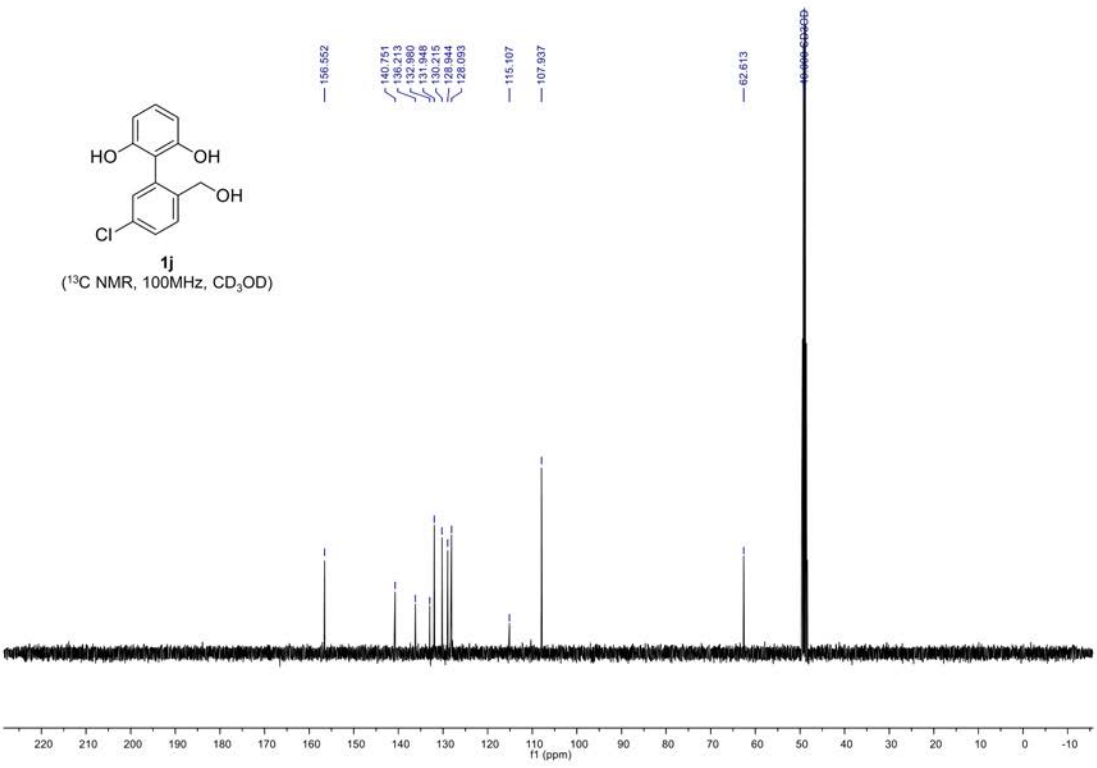

1j
(^{13}C NMR, 100MHz, CD_3OD)

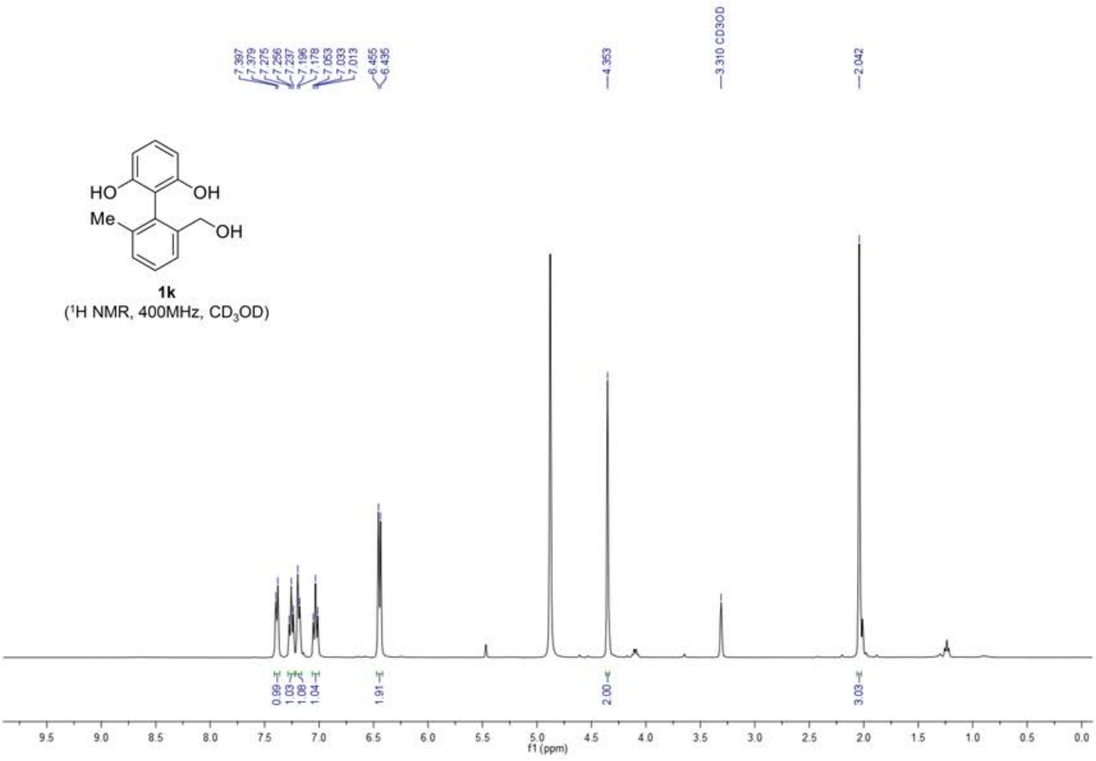

1k
(^{1}H NMR, 400MHz, CD_3OD)

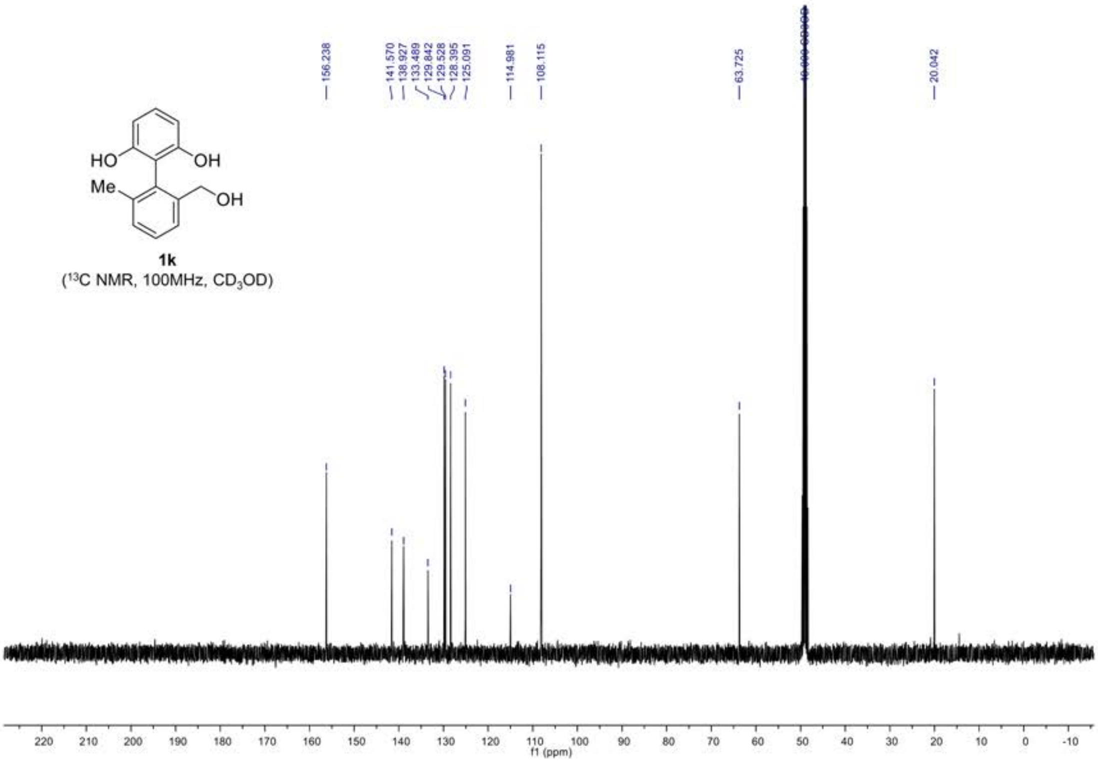

1k
(^{13}C NMR, 100MHz, CD_3OD)

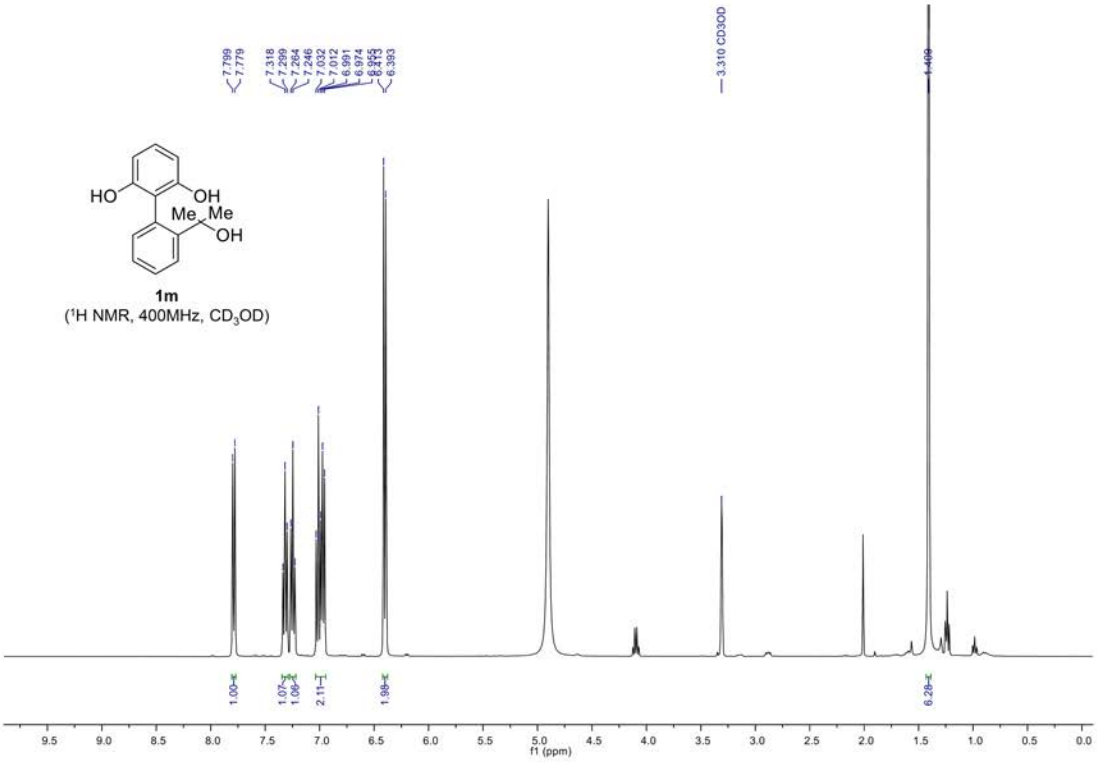
HO
OH
Me
Me
OH
1m
(1H NMR, 400MHz, CD3OD)
7.799
7.779
7.318
7.299
7.264
7.246
7.032
7.012
6.991
6.974
6.413
6.393
3.310 CD3OD
1.409
1.00
1.07
1.06
2.11
1.98
6.28
f1 (ppm)

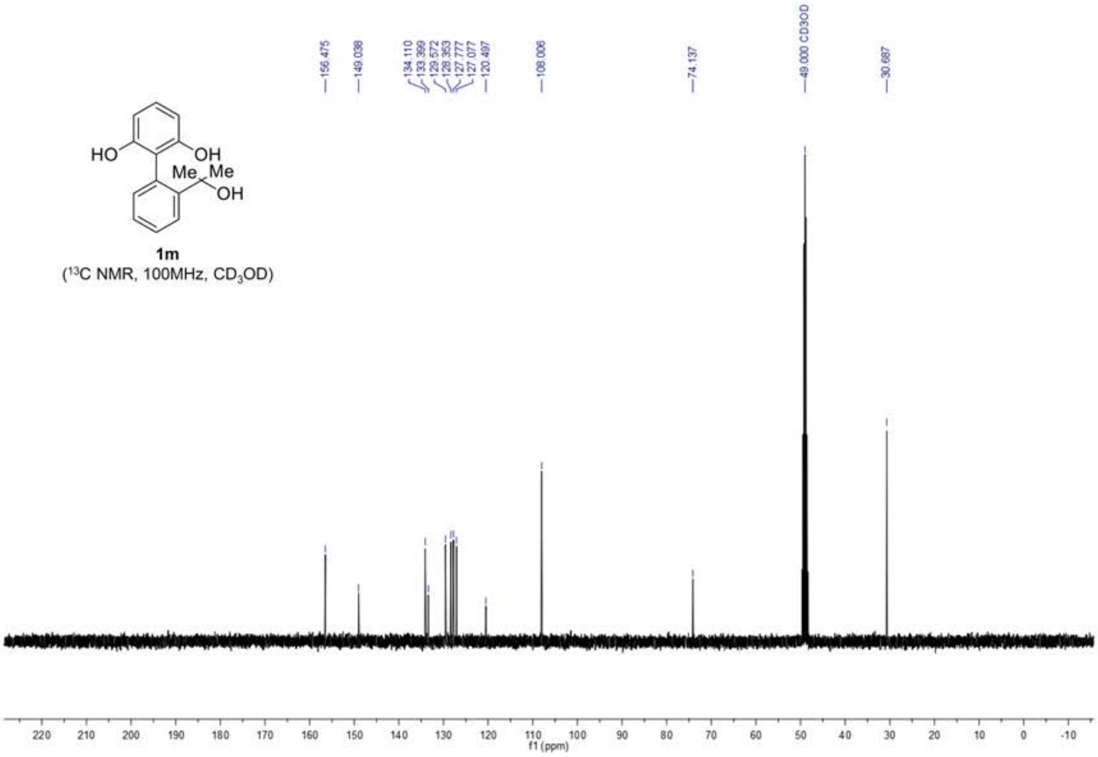
HO
OH
Me
Me
OH
1m
(13C NMR, 100MHz, CD3OD)
156.475
149.038
134.110
133.399
129.572
128.353
127.777
127.077
120.497
108.006
74.137
49.000 CD3OD
30.687
f1 (ppm)

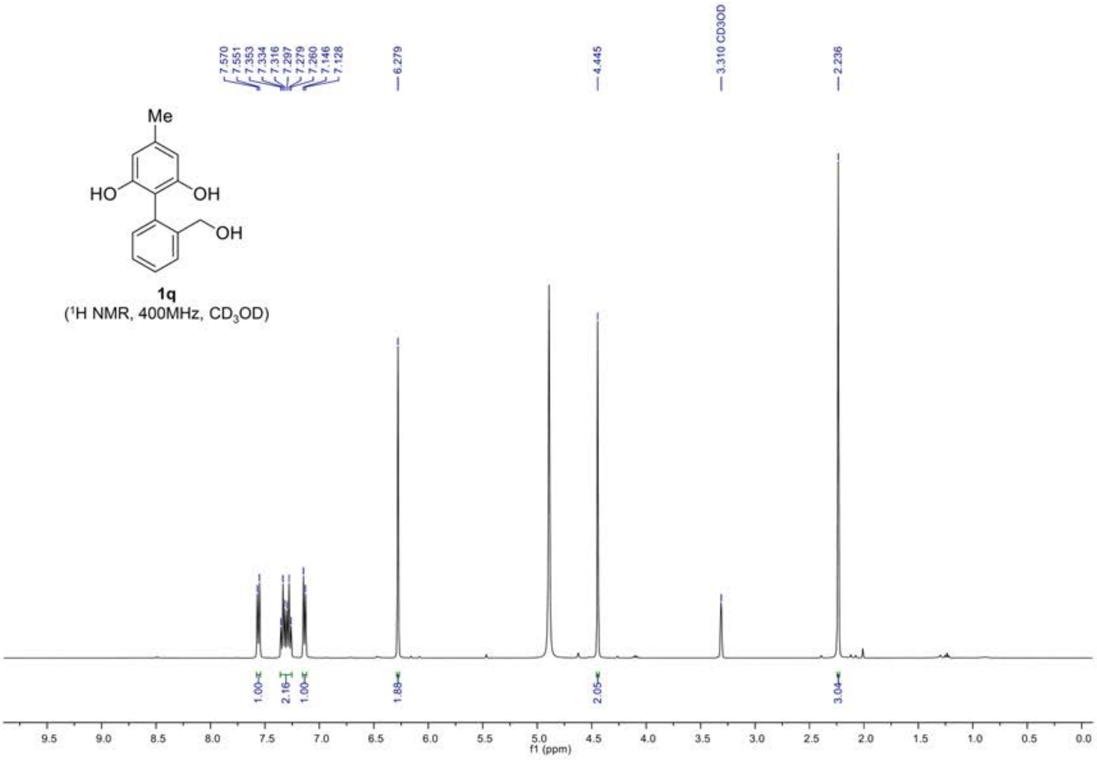

1q
(^{1}H NMR, 400MHz, CD_3OD)

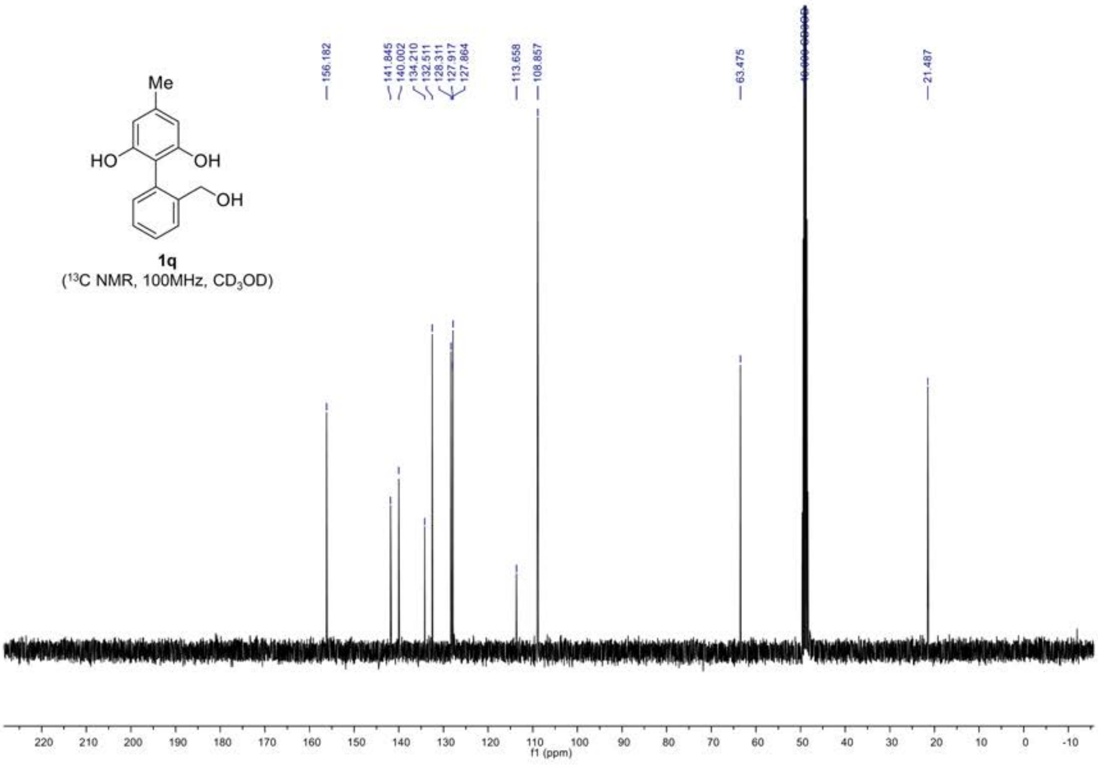

1q
(^{13}C NMR, 100MHz, CD_3OD)

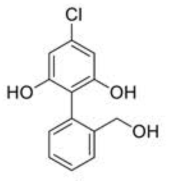

1r
(^{1}H NMR, 400MHz, CD_3OD)

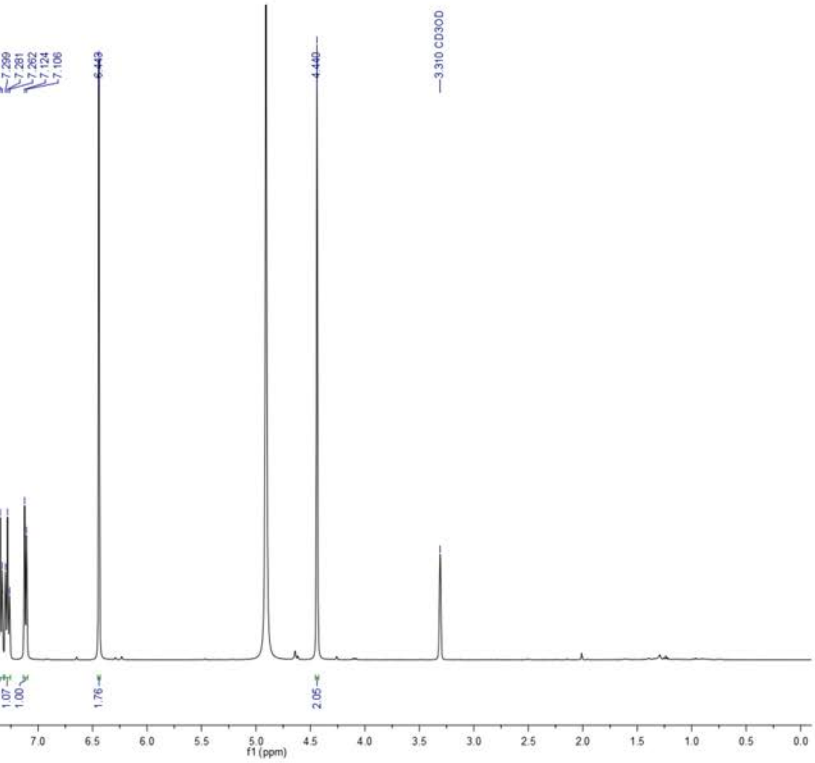

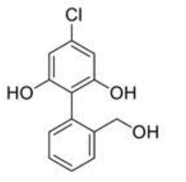

1r
(^{13}C NMR, 100MHz, CD_3OD)

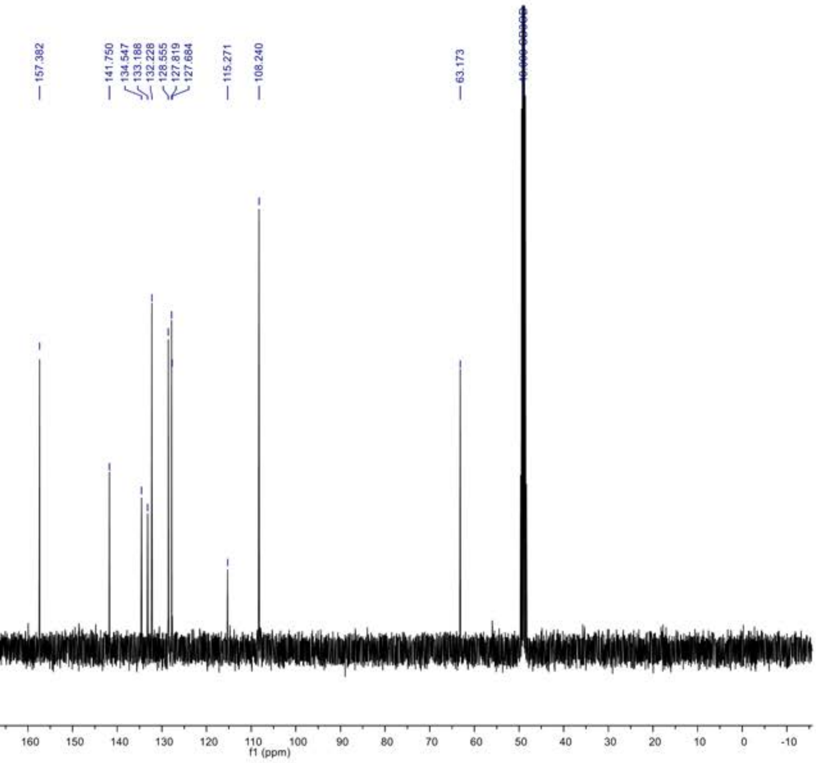

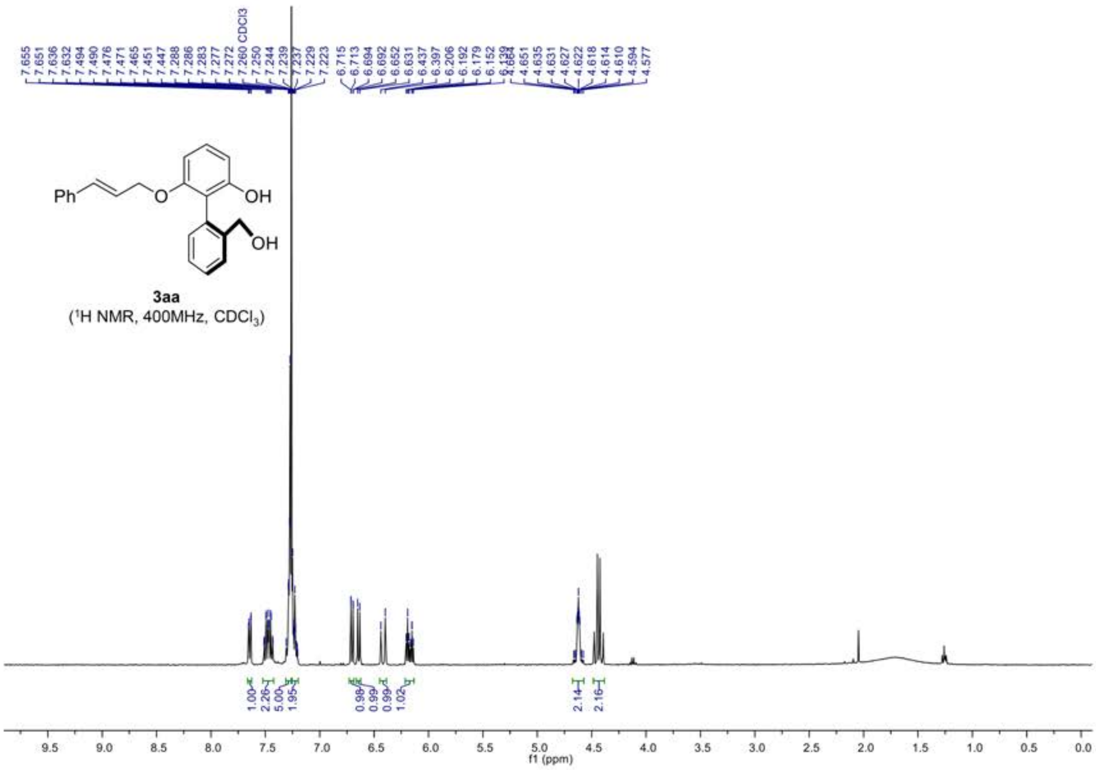

3aa
(^{1}H NMR, 400MHz, CDCl$_3$)

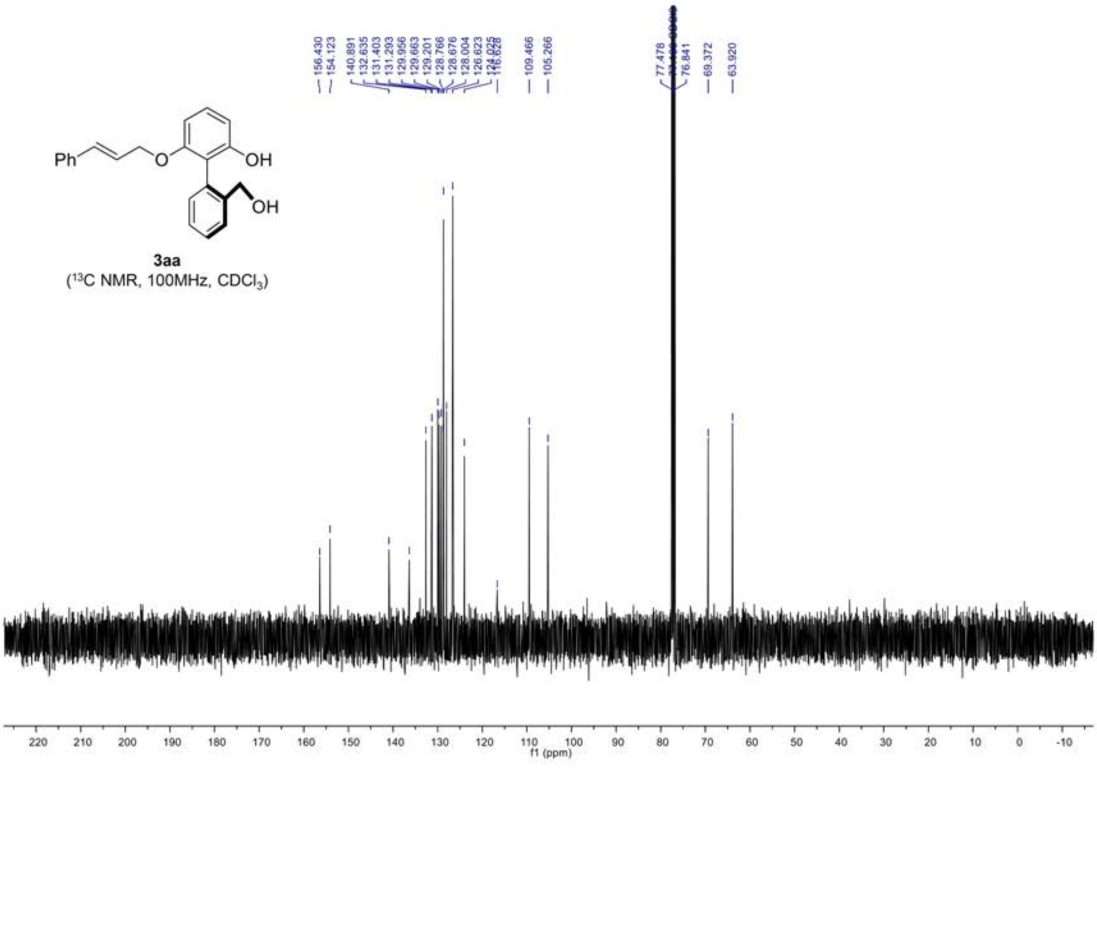

3aa
(^{13}C NMR, 100MHz, CDCl$_3$)

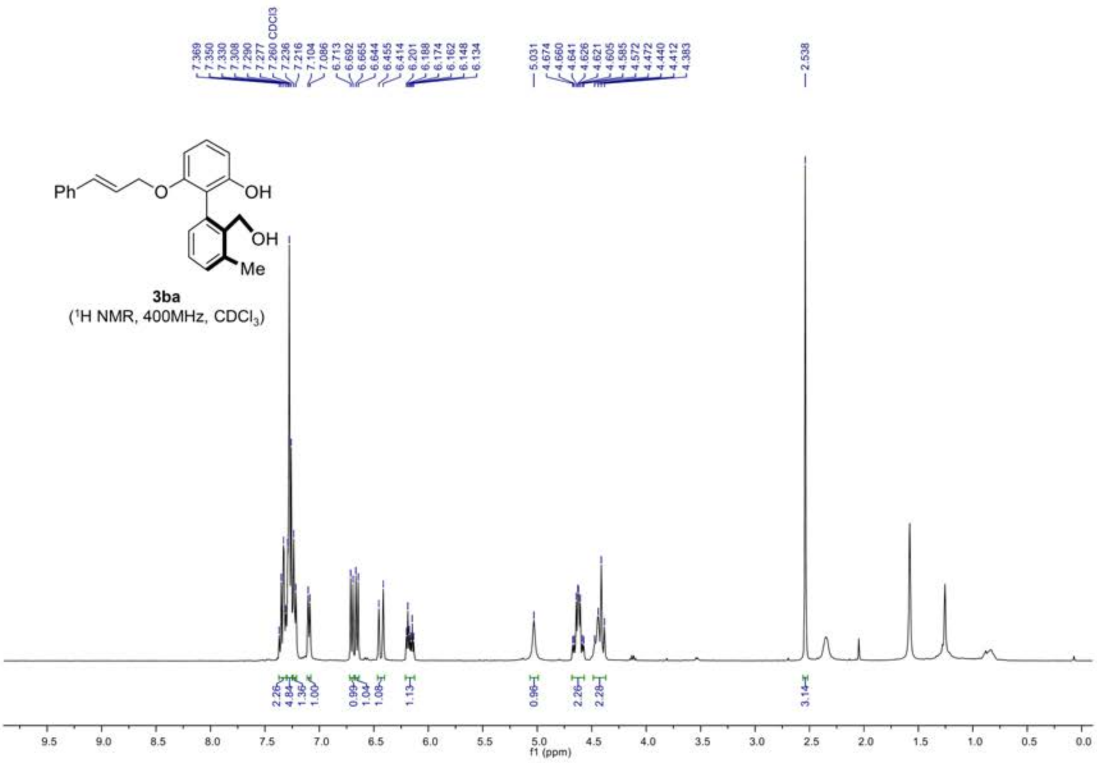

3ba
(^{1}H NMR, 400MHz, $CDCl_3$)

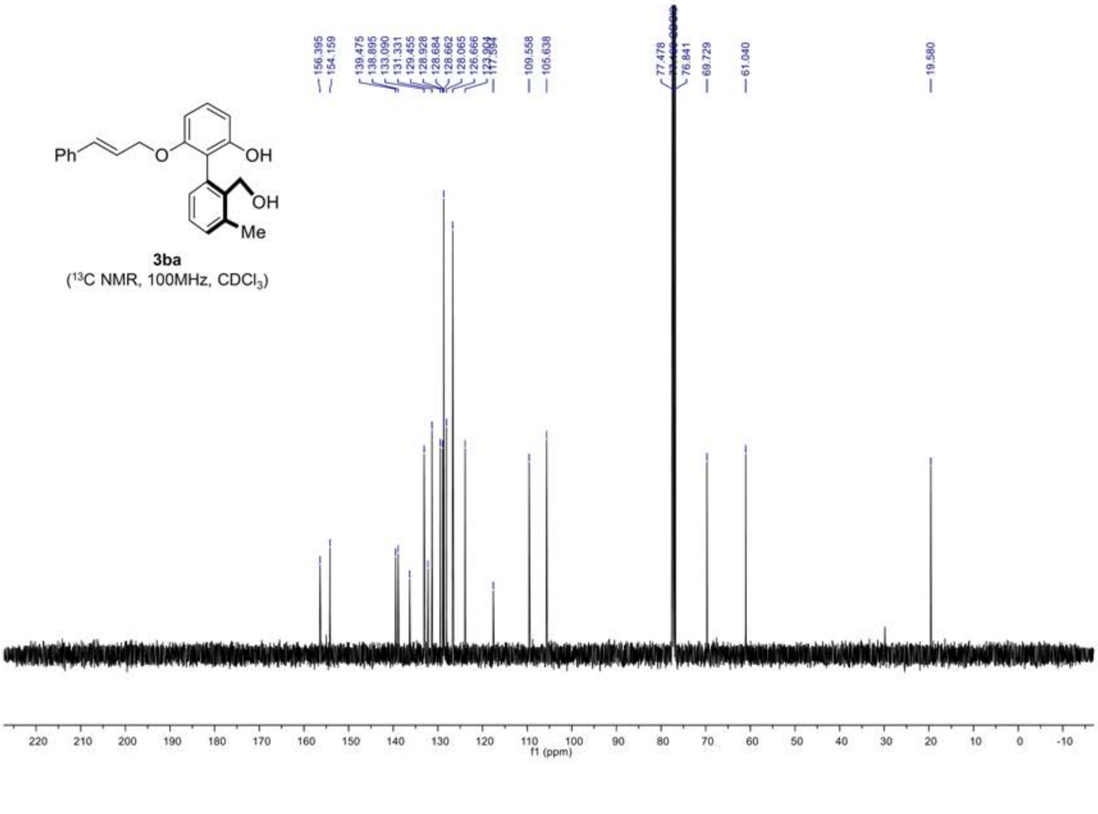

3ba
(^{13}C NMR, 100MHz, $CDCl_3$)

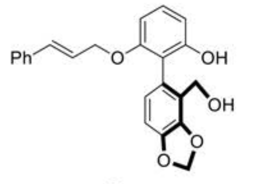

3ca
(^{1}H NMR, 400MHz, $CDCl_3$)

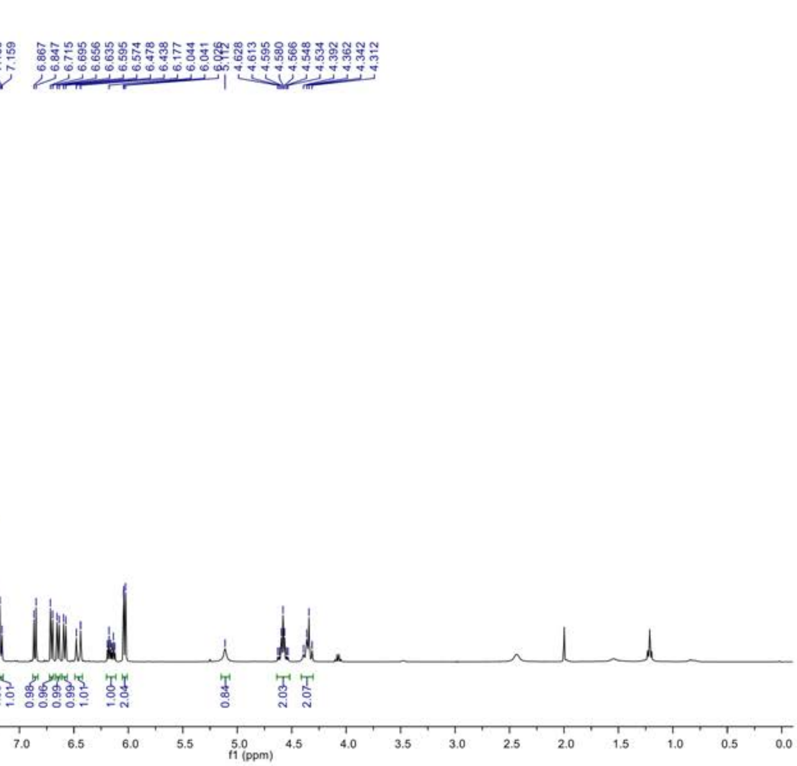

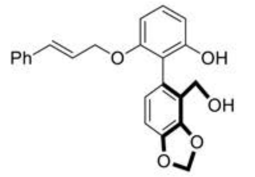

3ca
(^{13}C NMR, 100MHz, $CDCl_3$)

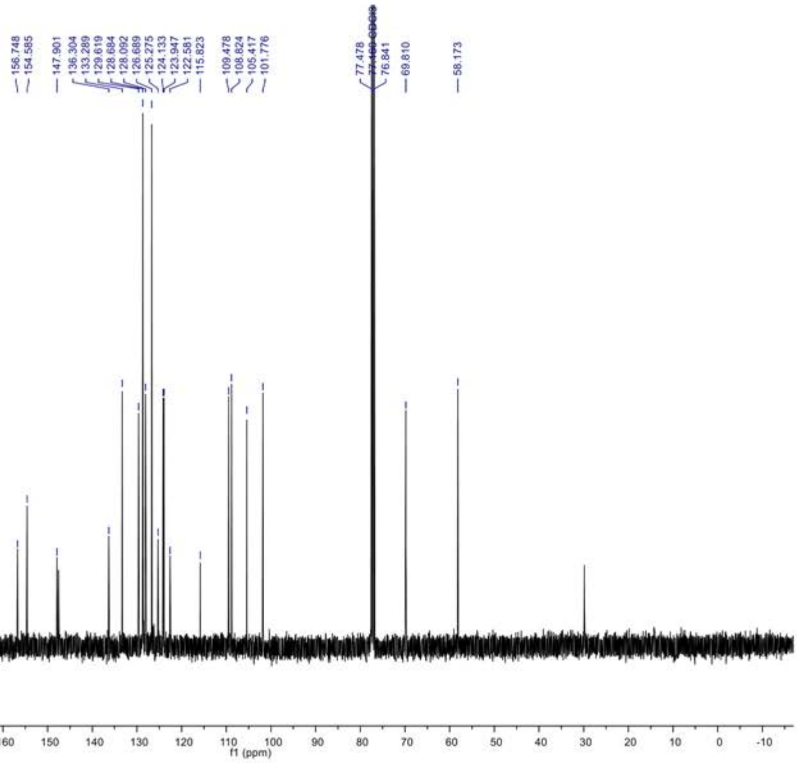

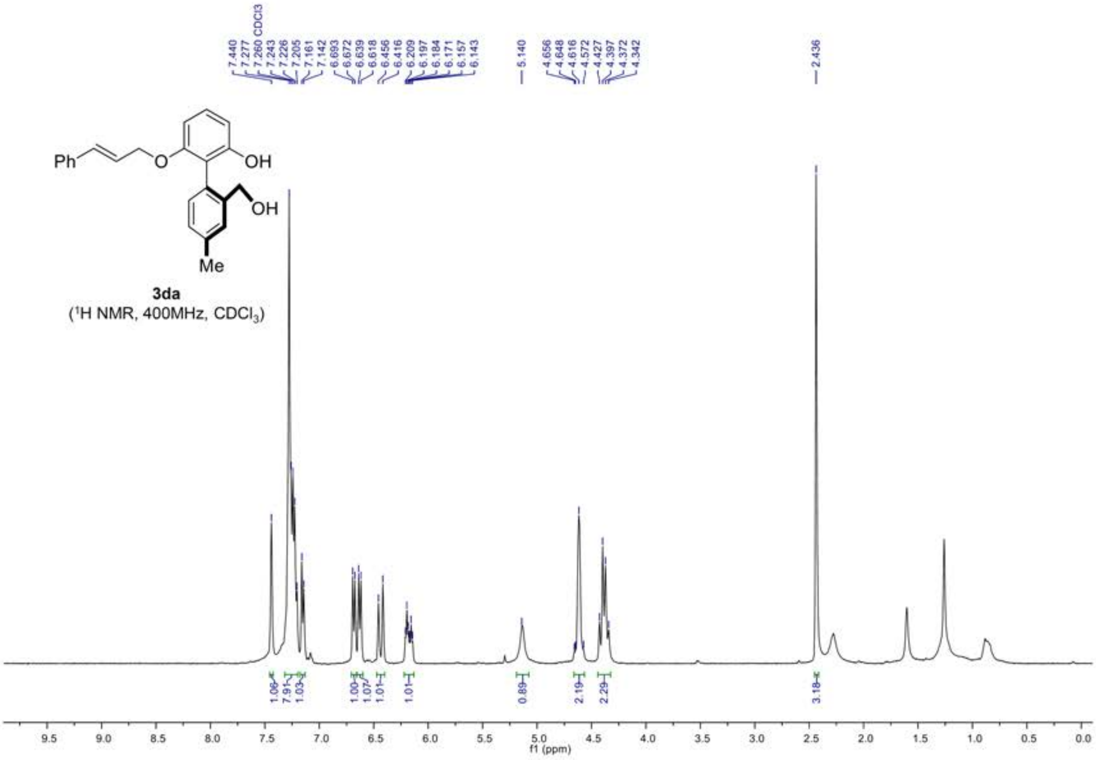

Ph
O
OH
OH
Me
3da
(1H NMR, 400MHz, CDCl3)

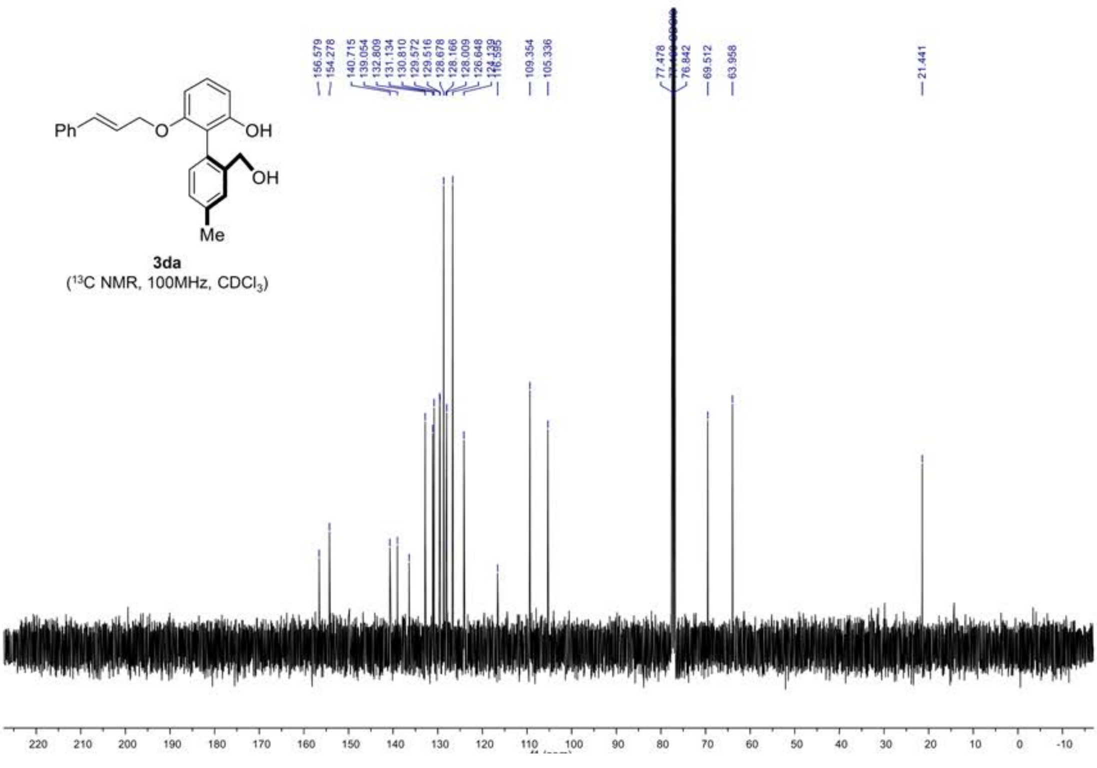

Ph
O
OH
OH
Me
3da
(13C NMR, 100MHz, CDCl3)

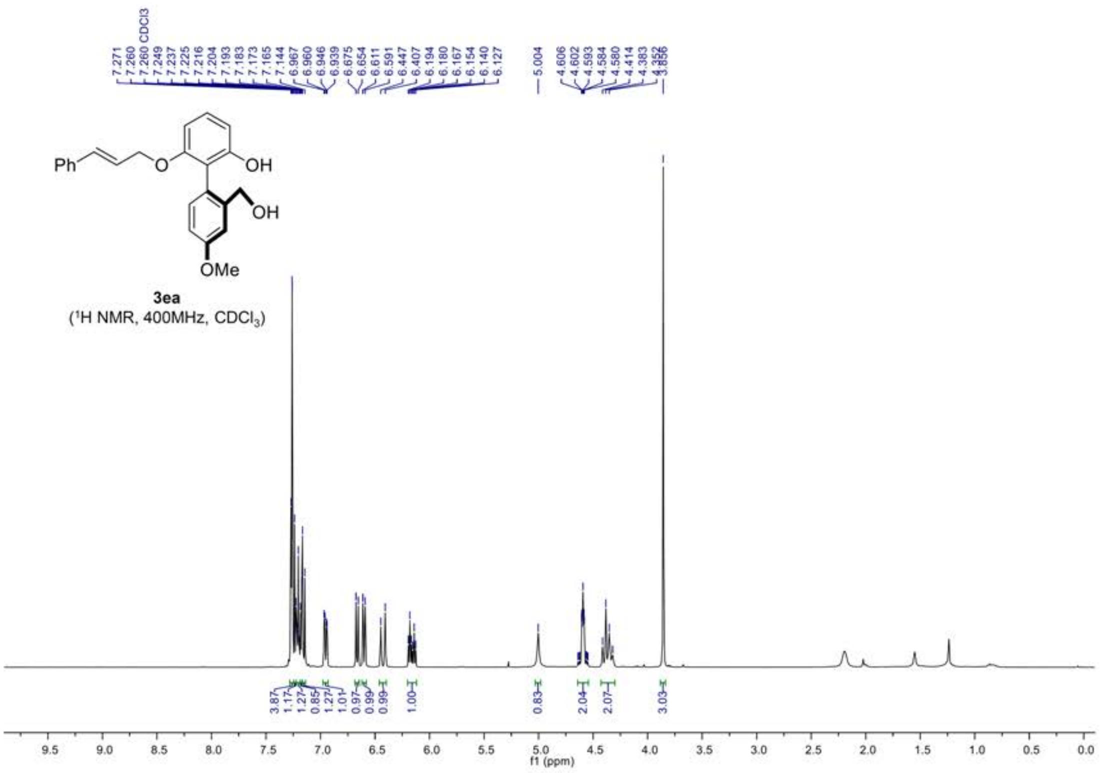

3ea
(^{1}H NMR, 400MHz, $CDCl_3$)

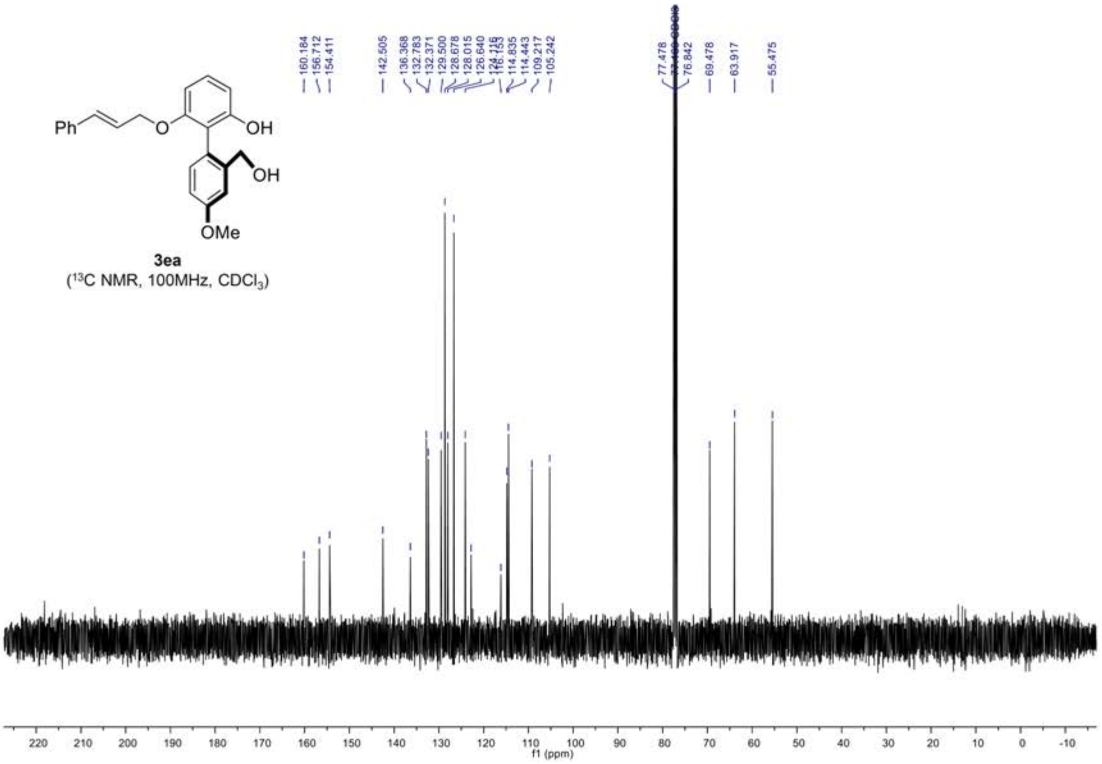

3ea
(^{13}C NMR, 100MHz, $CDCl_3$)

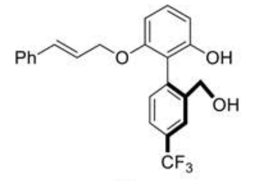

3fa

(^{1}H NMR, 400MHz, $CDCl_3$)

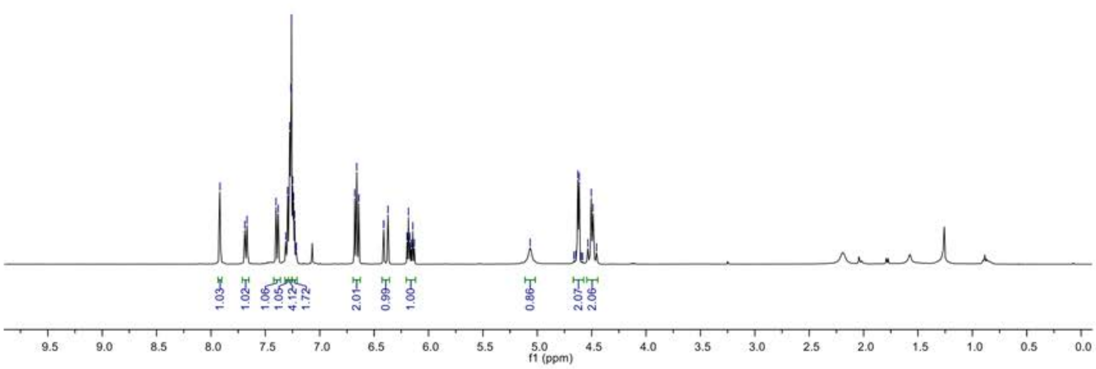

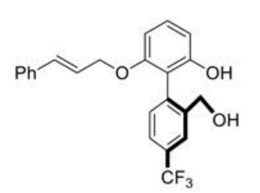

3fa

(^{13}C NMR, 100MHz, $CDCl_3$)

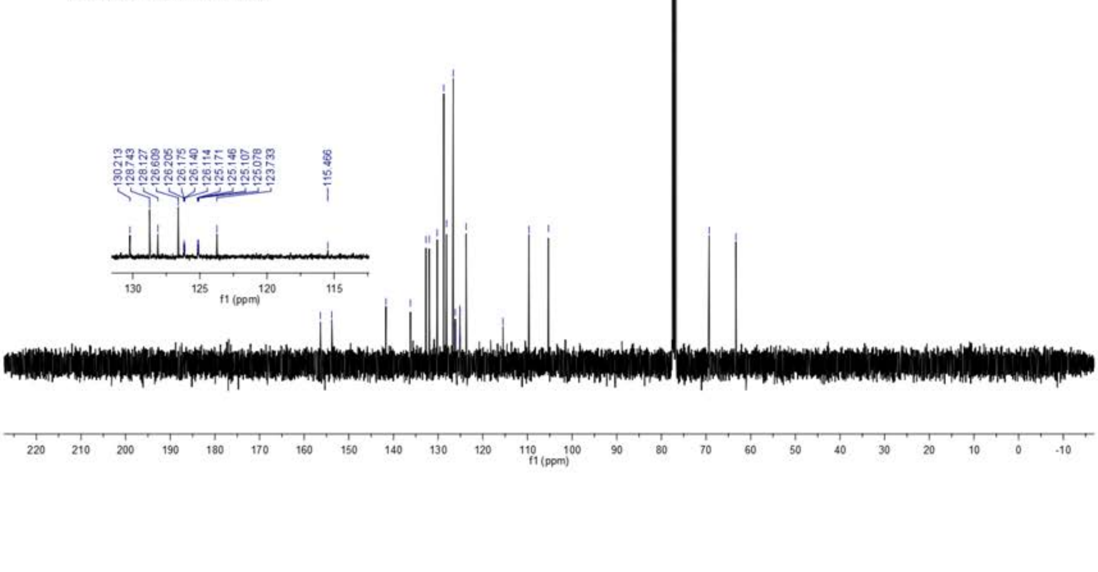

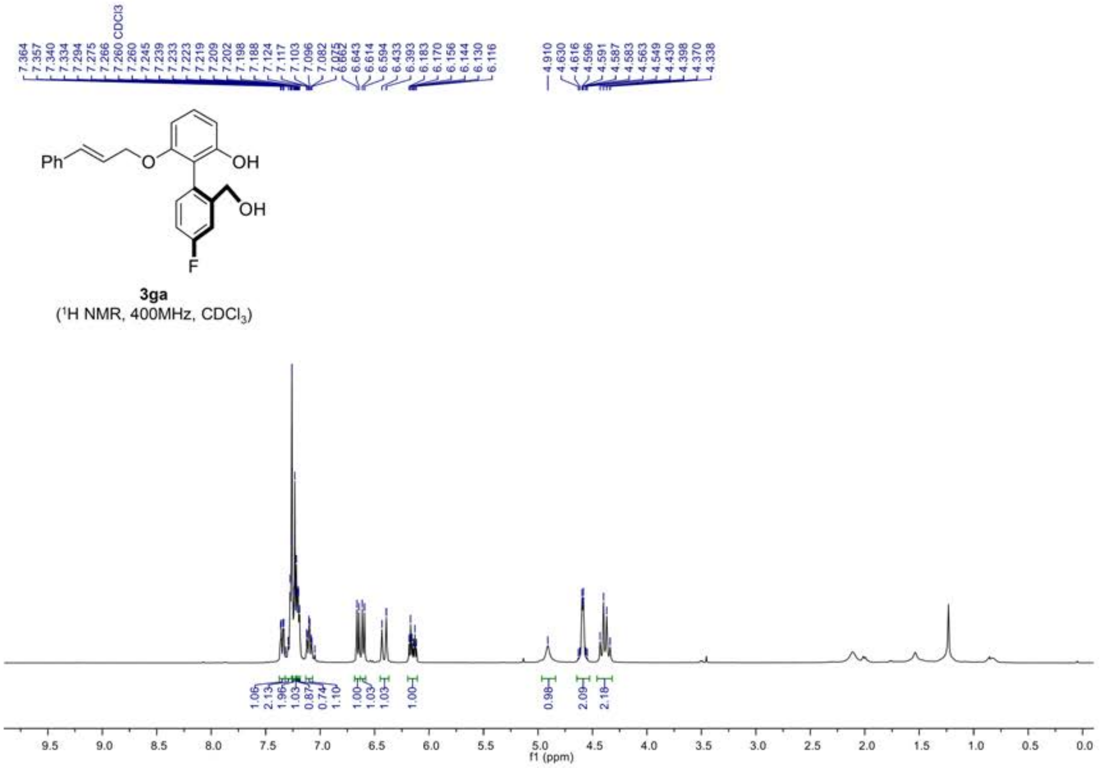

3ga
(^{1}H NMR, 400MHz, $CDCl_3$)

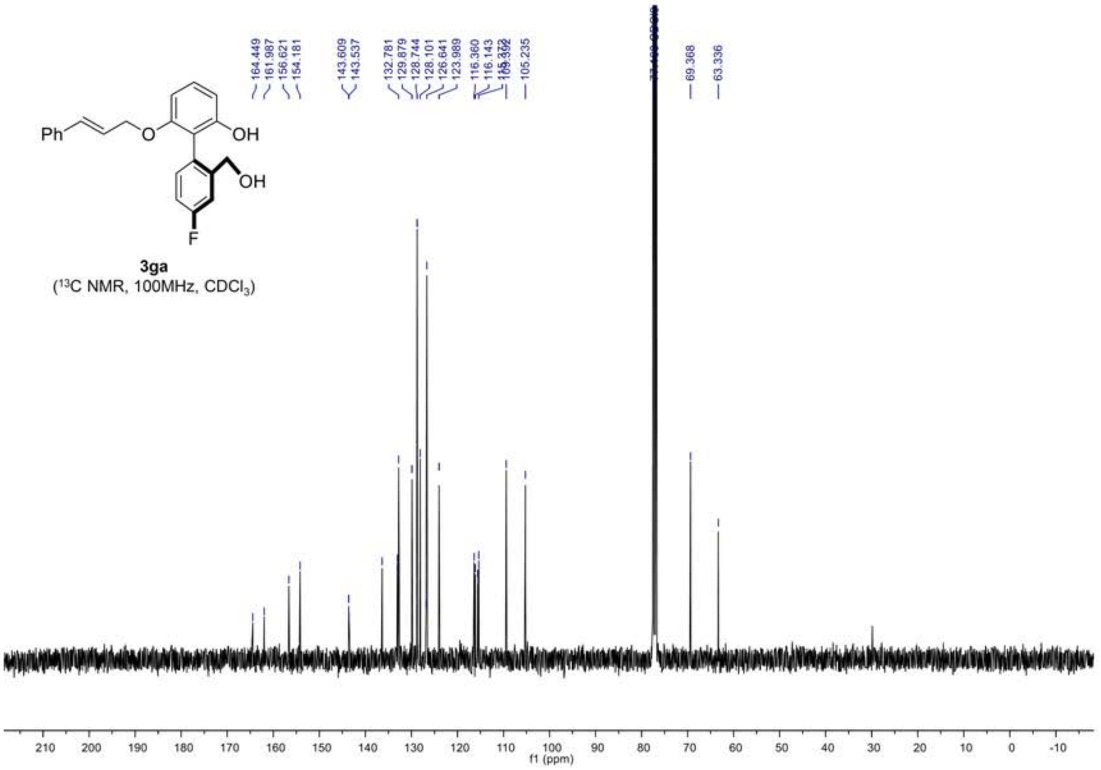

3ga
(^{13}C NMR, 100MHz, $CDCl_3$)

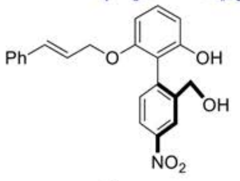

3ha
(^{1}H NMR, 400MHz, $CDCl_3$)

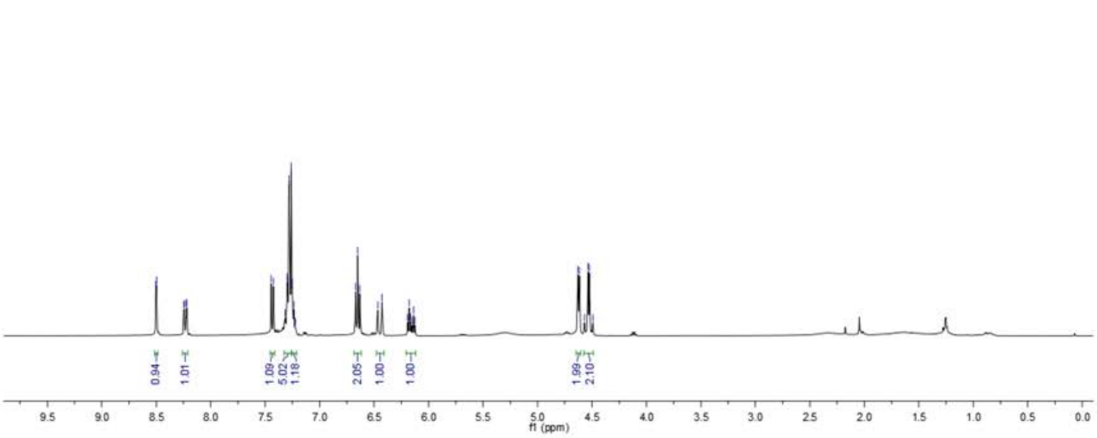

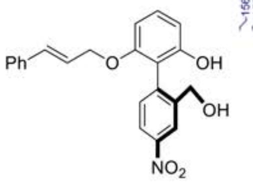

3ha
(^{13}C NMR, 100MHz, $CDCl_3$)

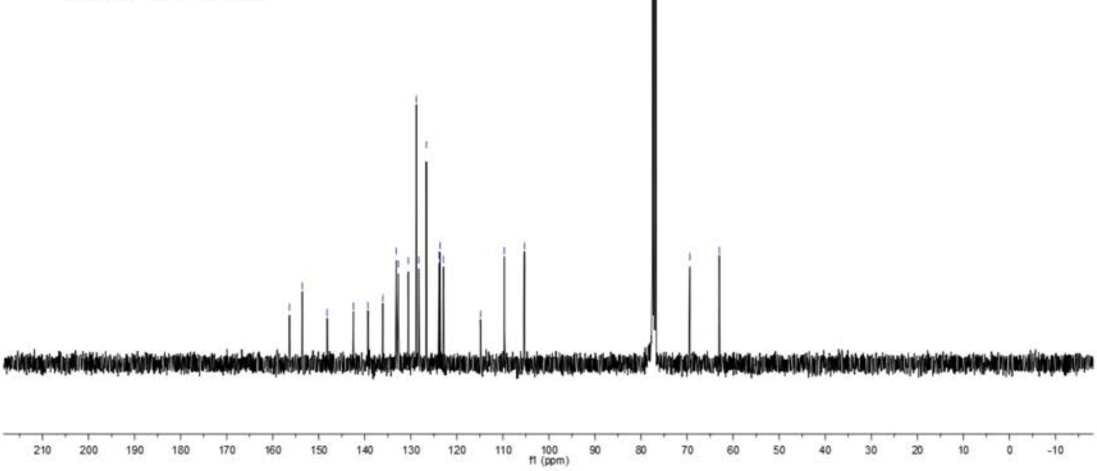

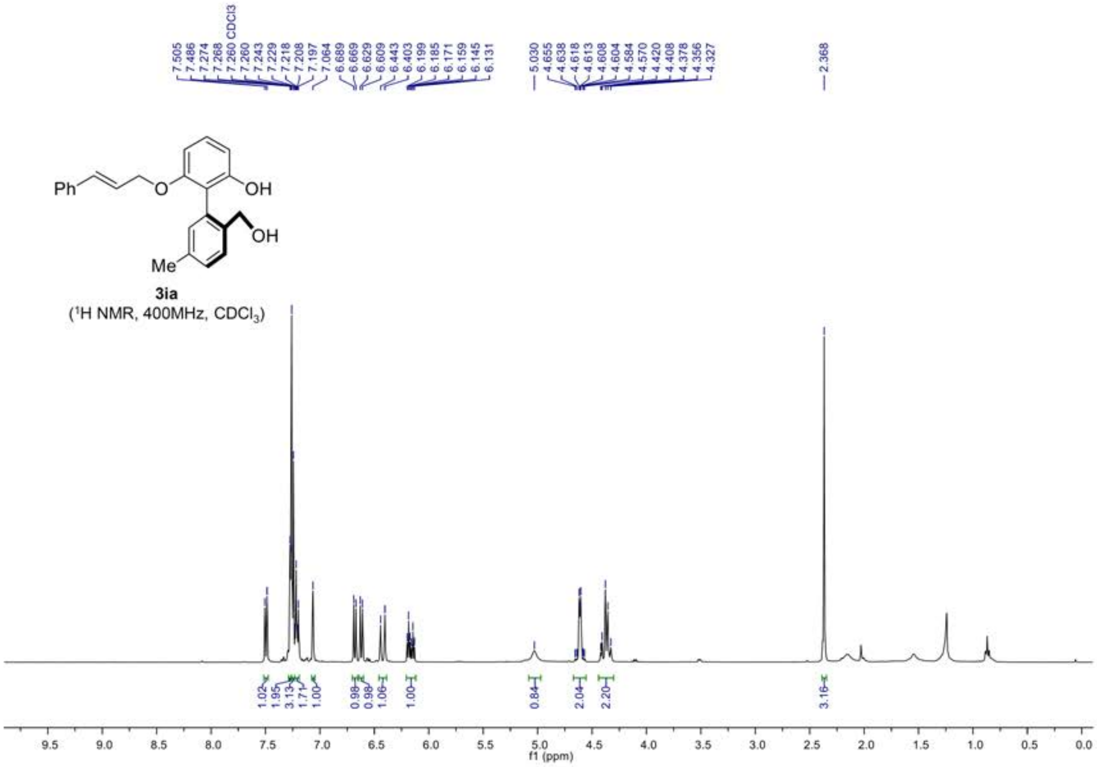

3ia
(^{1}H NMR, 400MHz, $CDCl_3$)

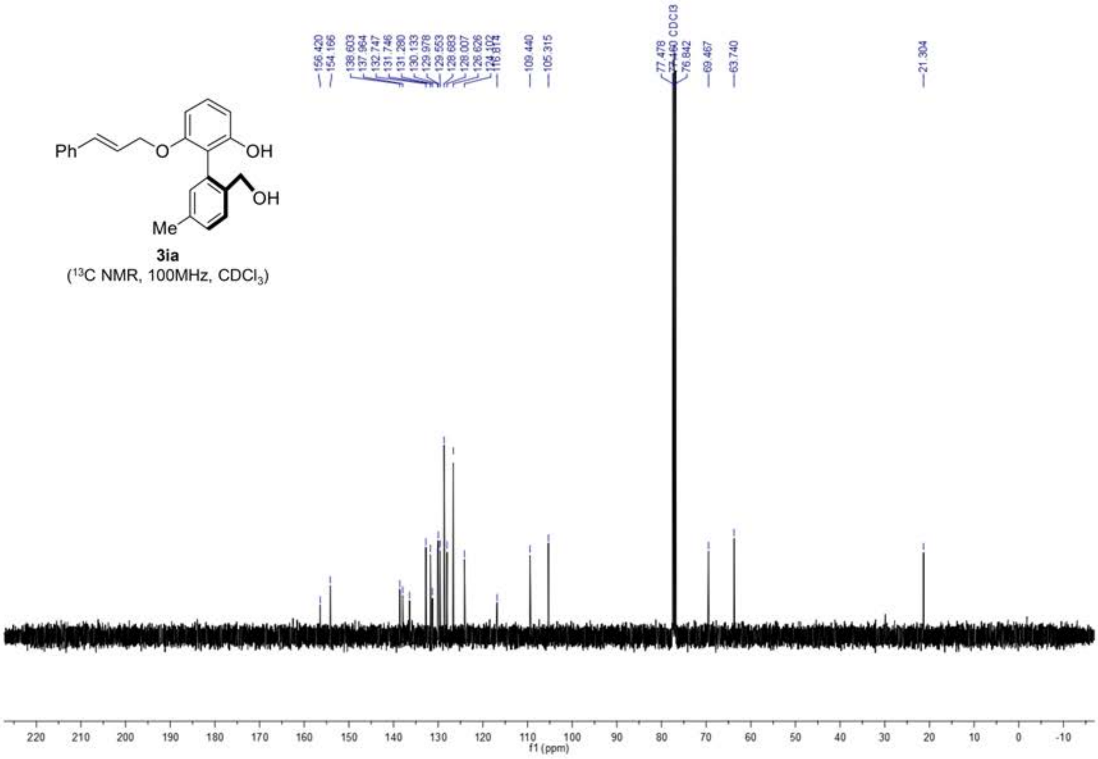

3ia
(^{13}C NMR, 100MHz, $CDCl_3$)

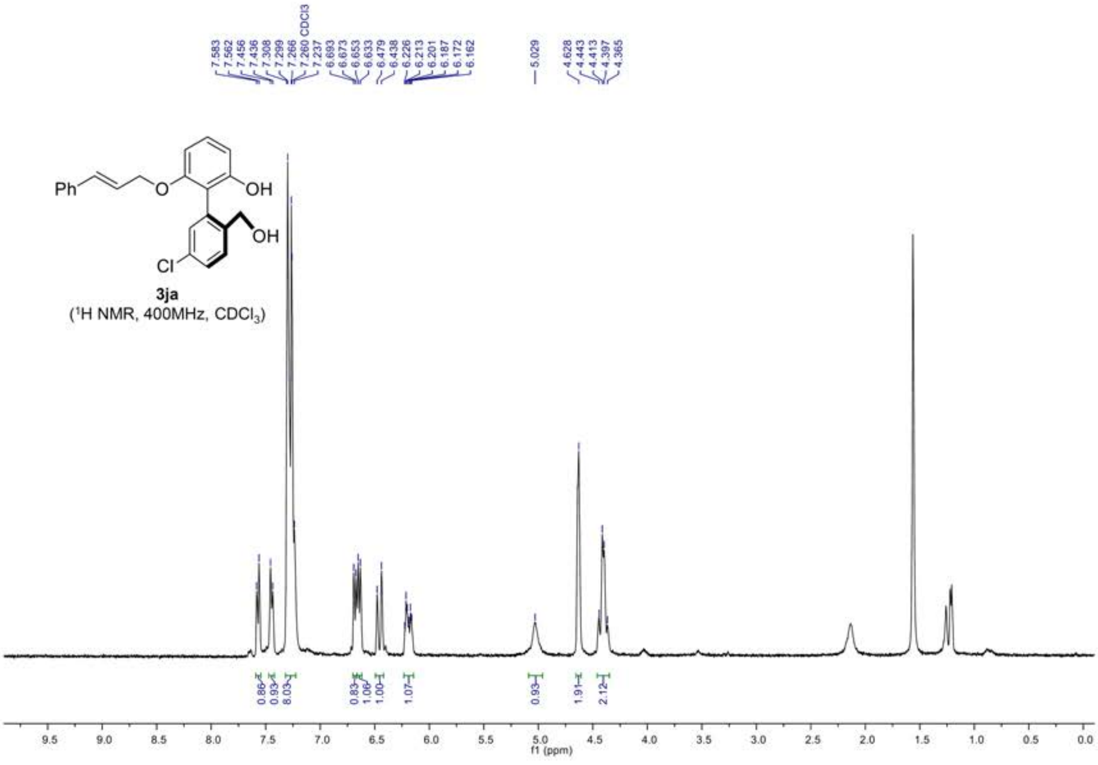

3ja
(^{1}H NMR, 400MHz, $CDCl_3$)

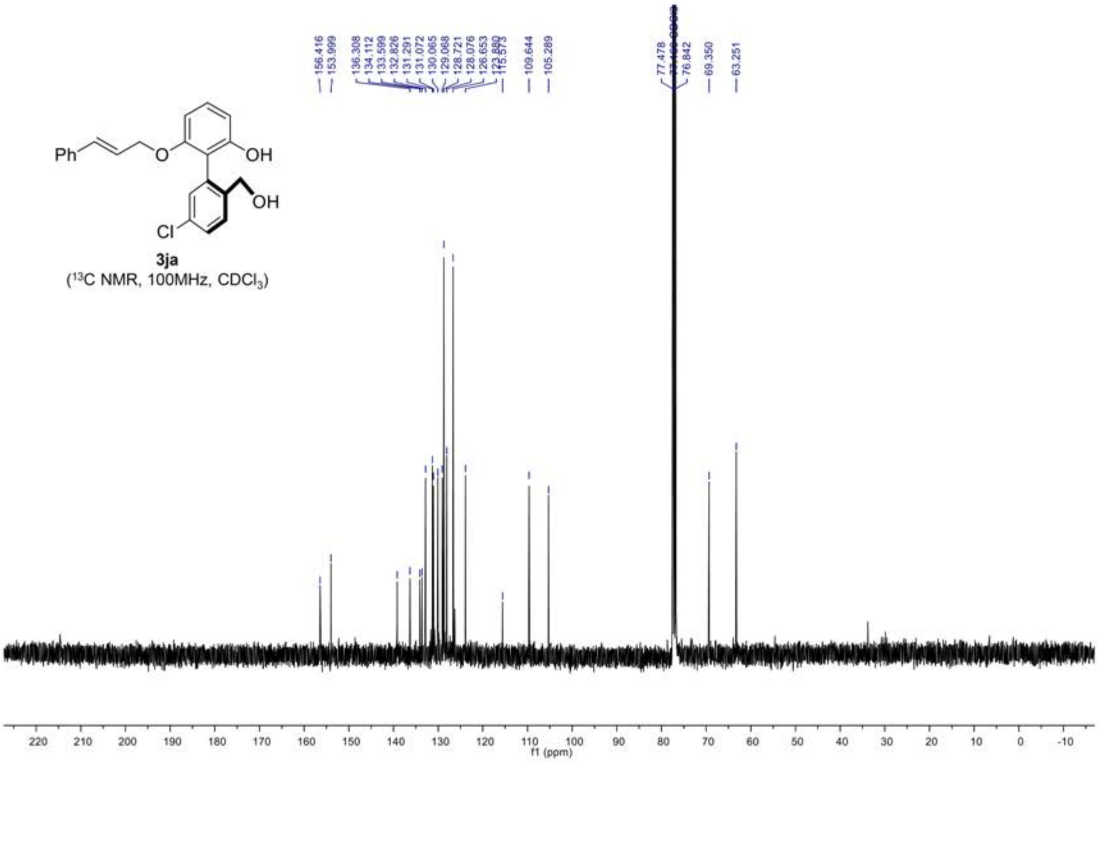

3ja
(^{13}C NMR, 100MHz, $CDCl_3$)

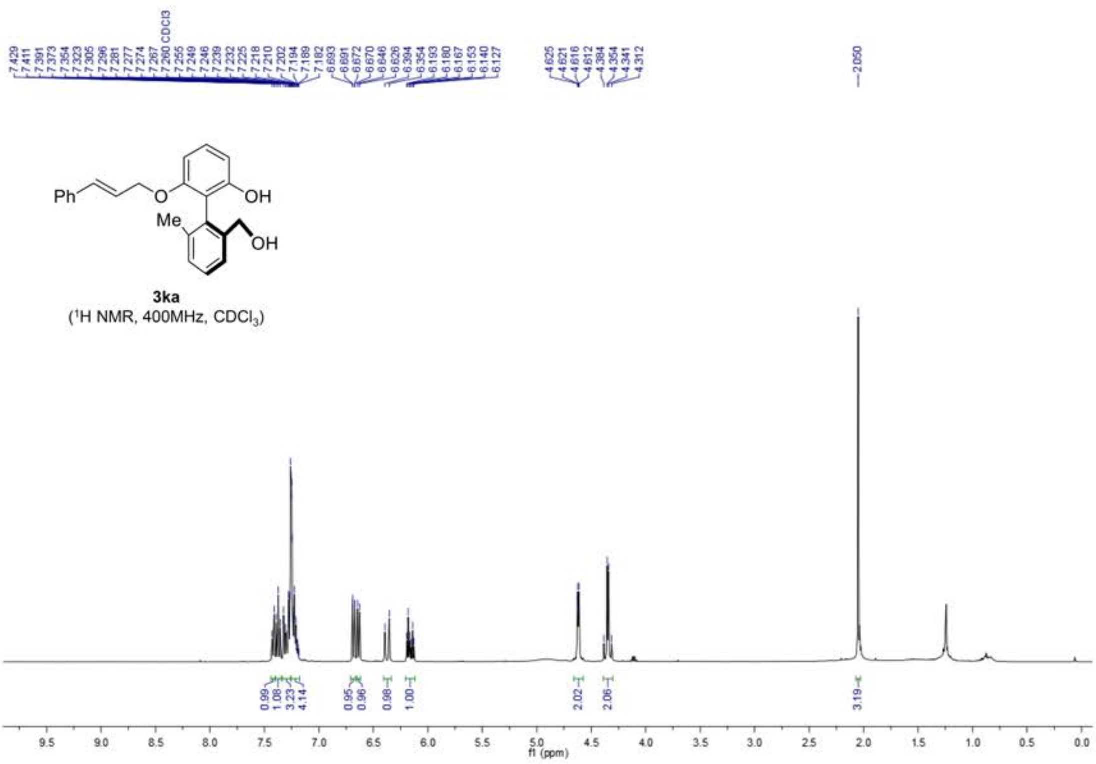

3ka
(^{1}H NMR, 400MHz, $CDCl_3$)

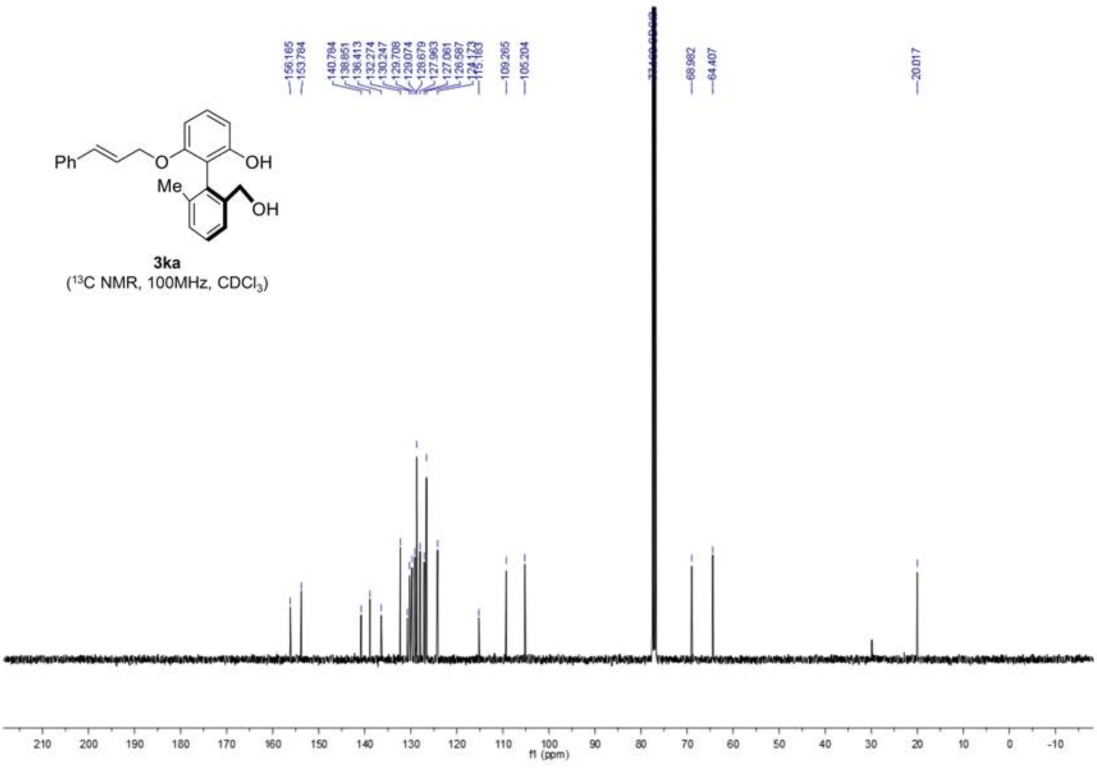

3ka
(^{13}C NMR, 100MHz, $CDCl_3$)

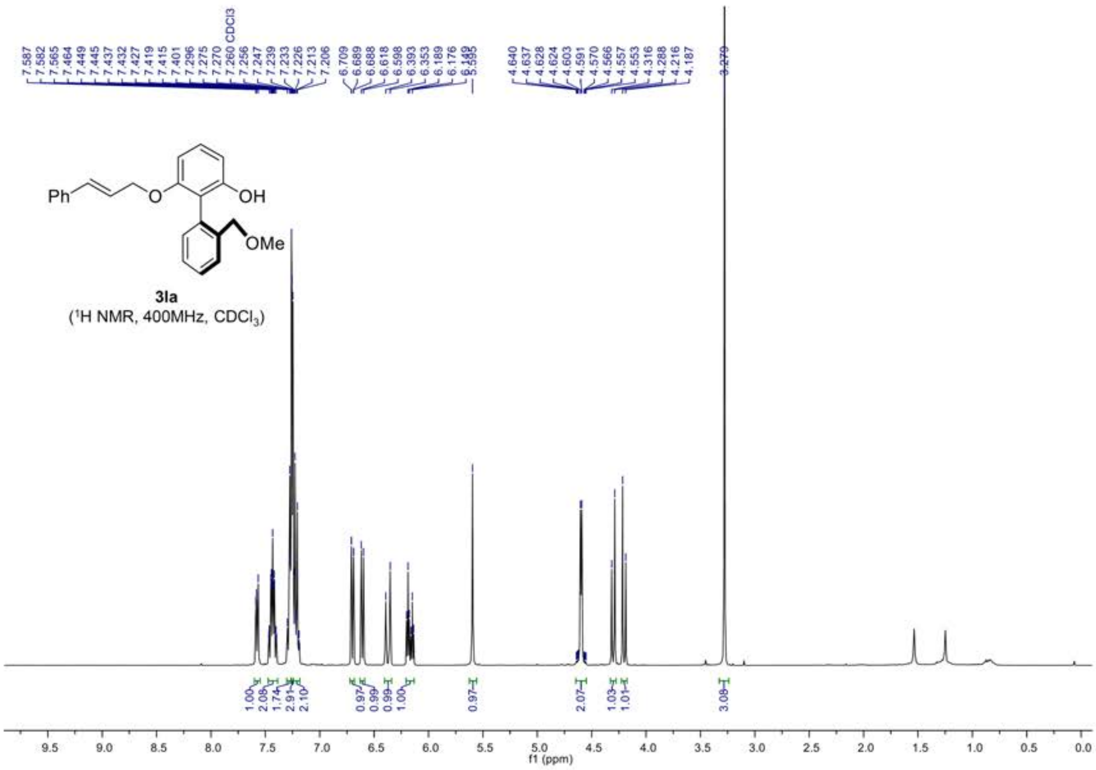
3la
(1H NMR, 400MHz, CDCl3)
f1 (ppm)

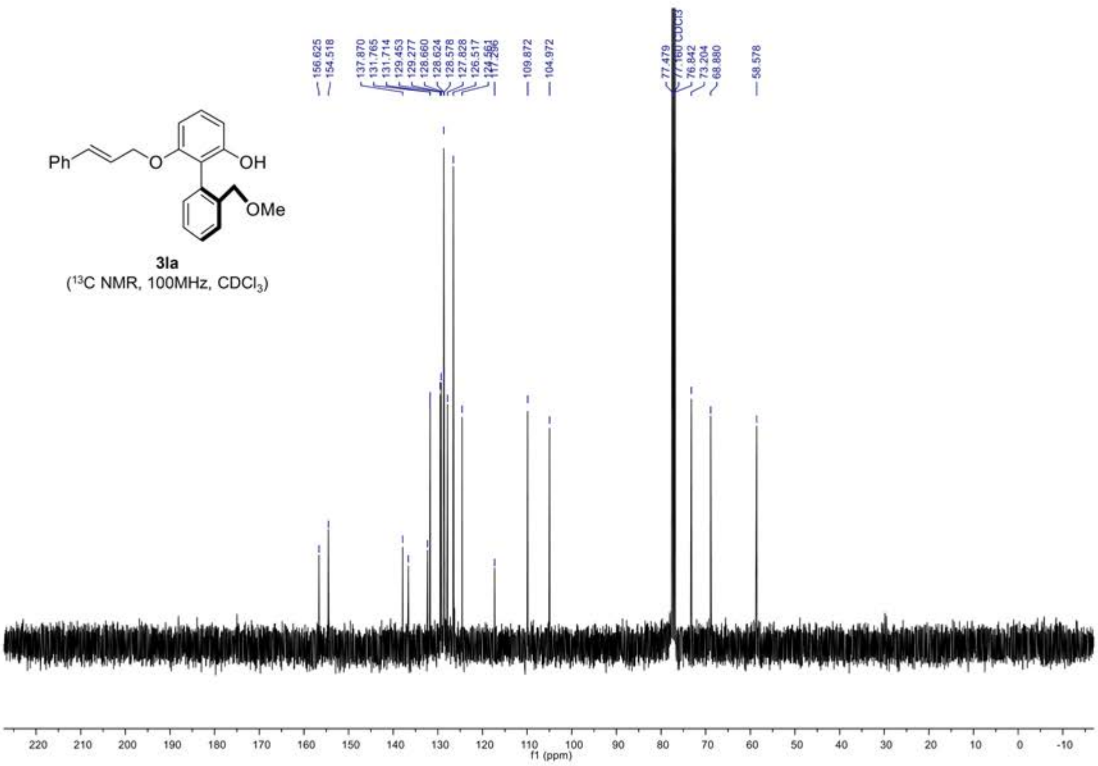
3la
(13C NMR, 100MHz, CDCl3)
f1 (ppm)

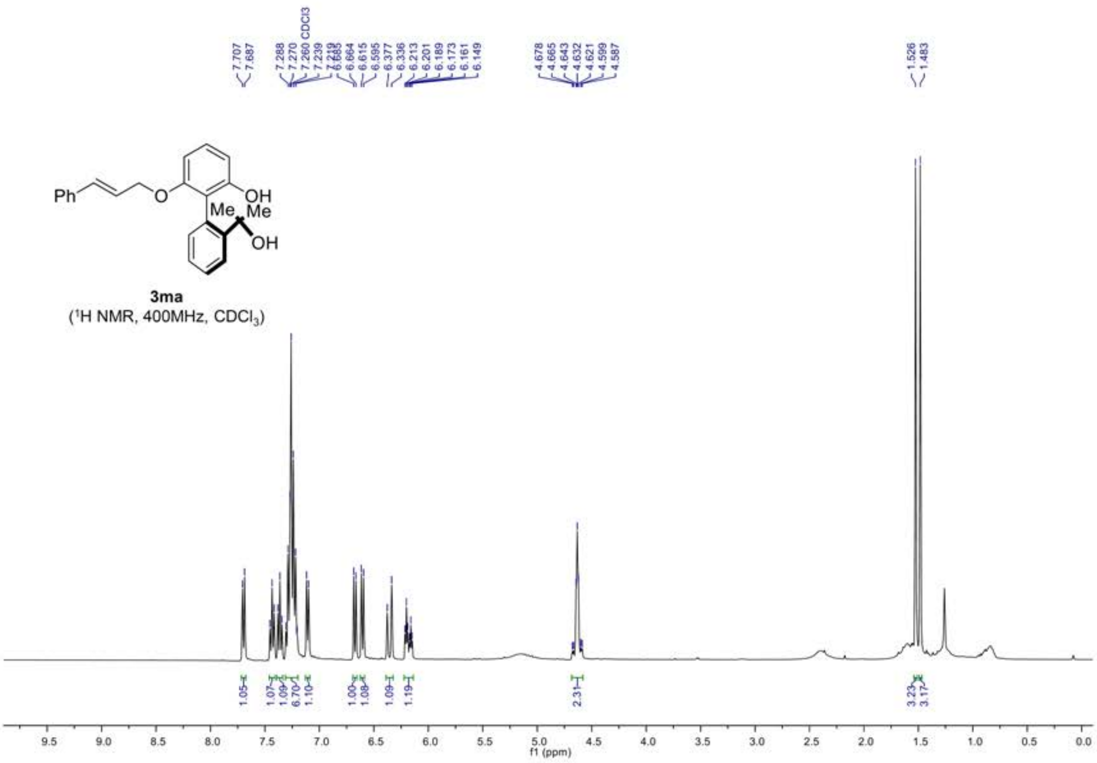

3ma
(^{1}H NMR, 400MHz, $CDCl_3$)

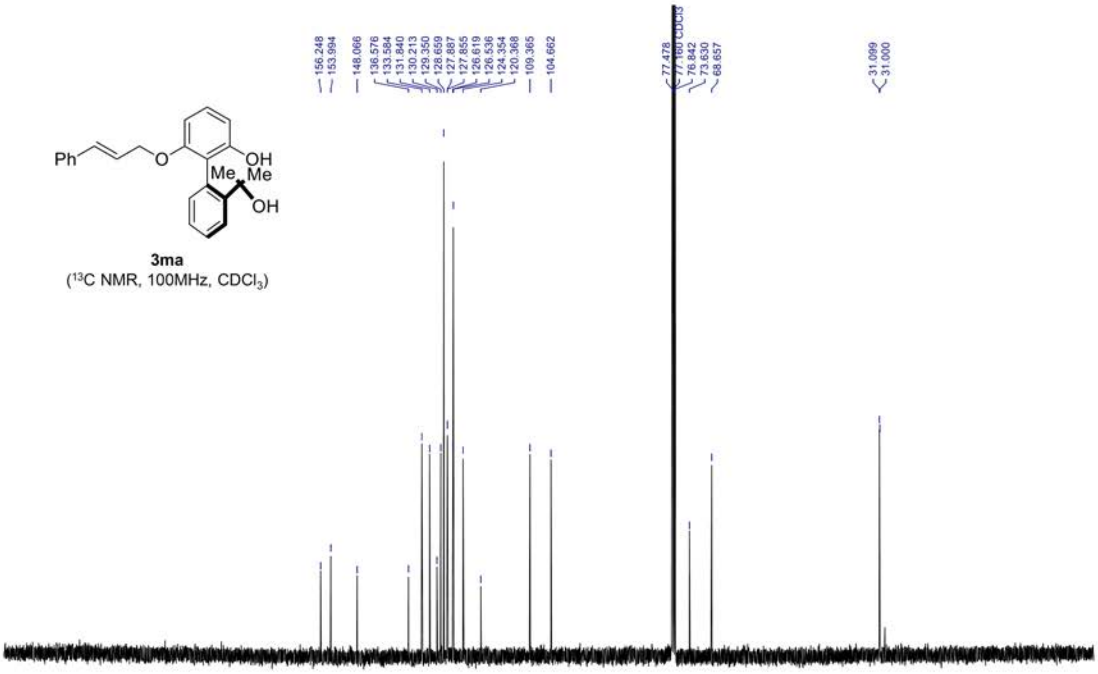

3ma
(^{13}C NMR, 100MHz, $CDCl_3$)

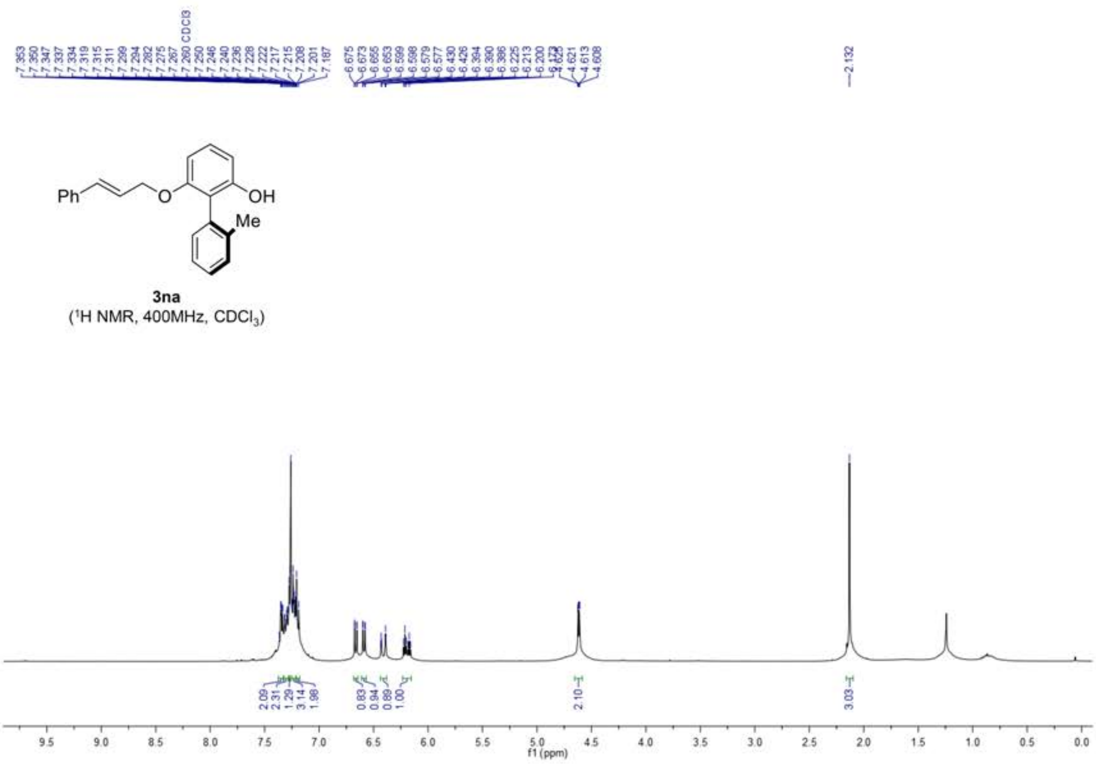

3na
(^{1}H NMR, 400MHz, $CDCl_3$)

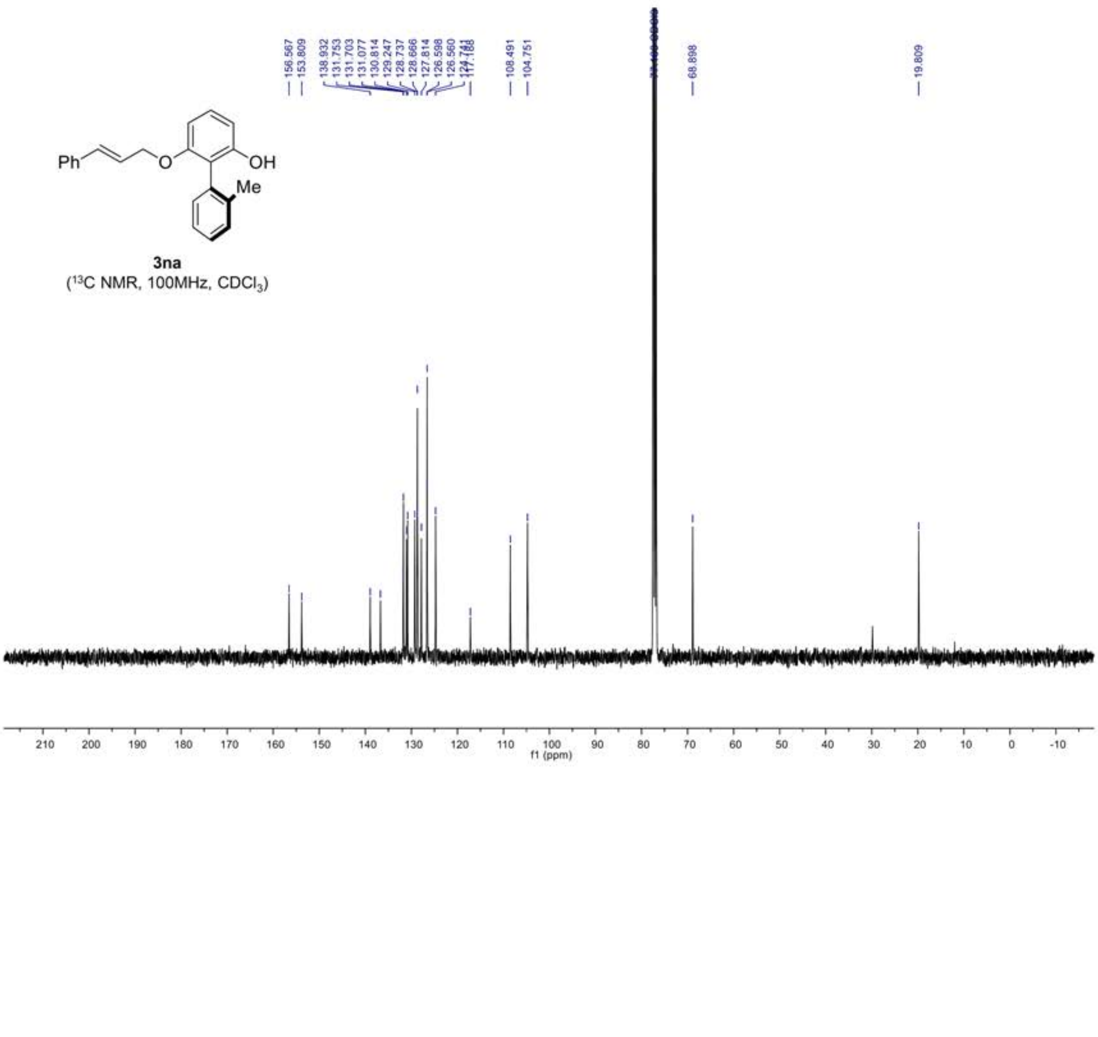

3na
(^{13}C NMR, 100MHz, $CDCl_3$)

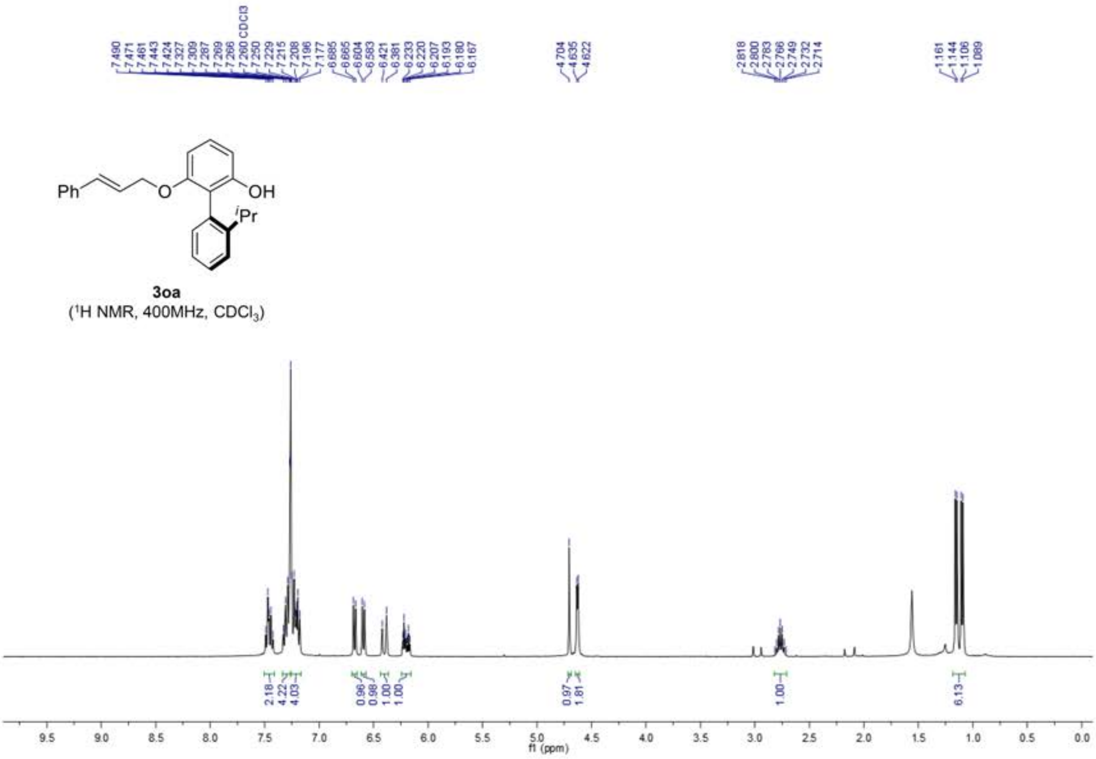

3oa
(^{1}H NMR, 400MHz, CDCl$_3$)

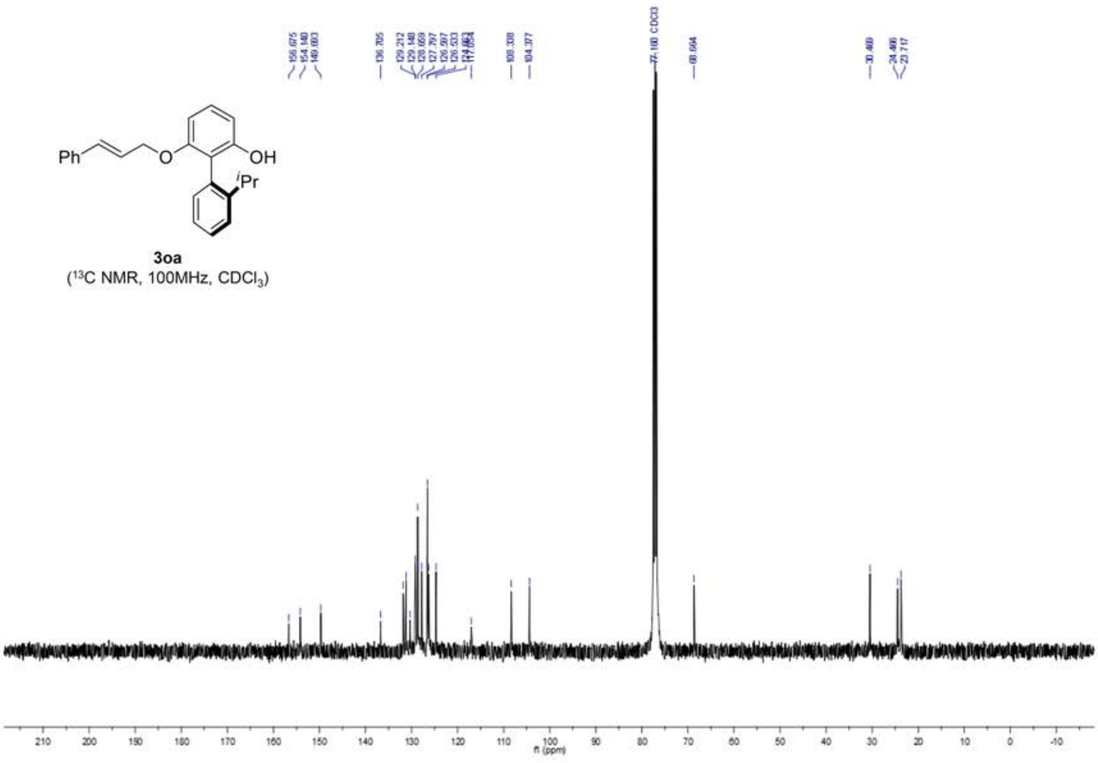

3oa
(^{13}C NMR, 100MHz, CDCl$_3$)

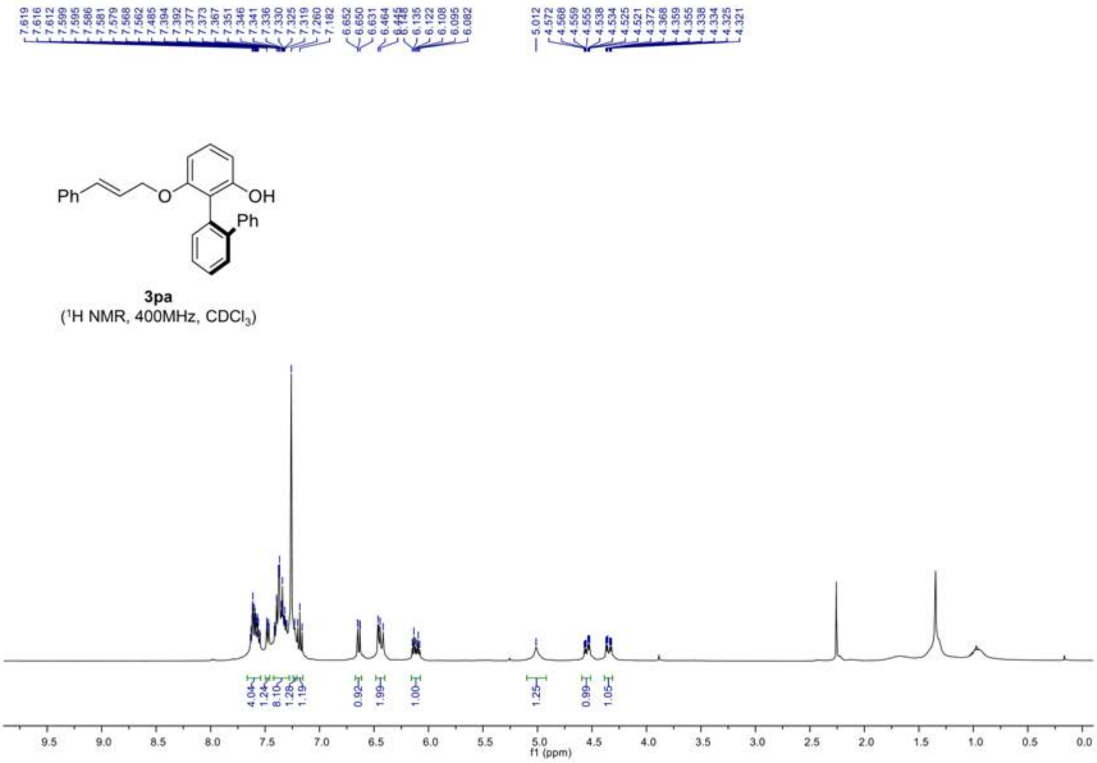

3pa
(^{1}H NMR, 400MHz, $CDCl_3$)

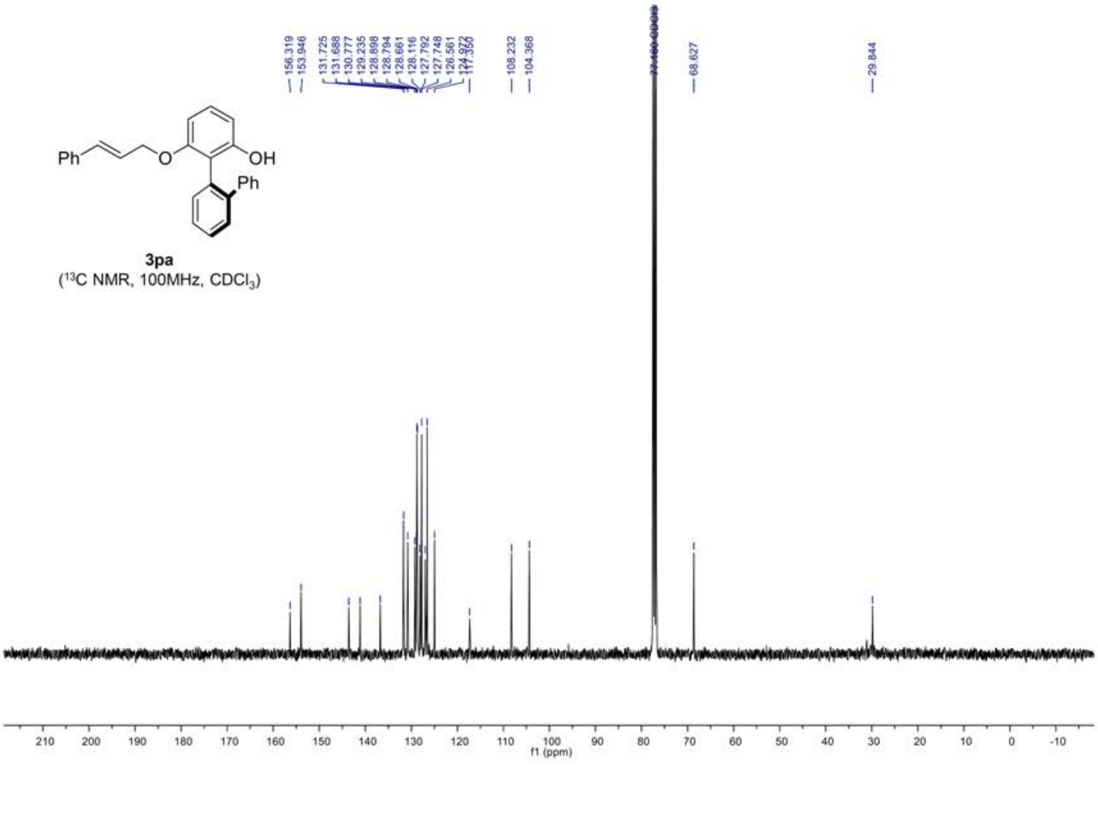

3pa
(^{13}C NMR, 100MHz, $CDCl_3$)

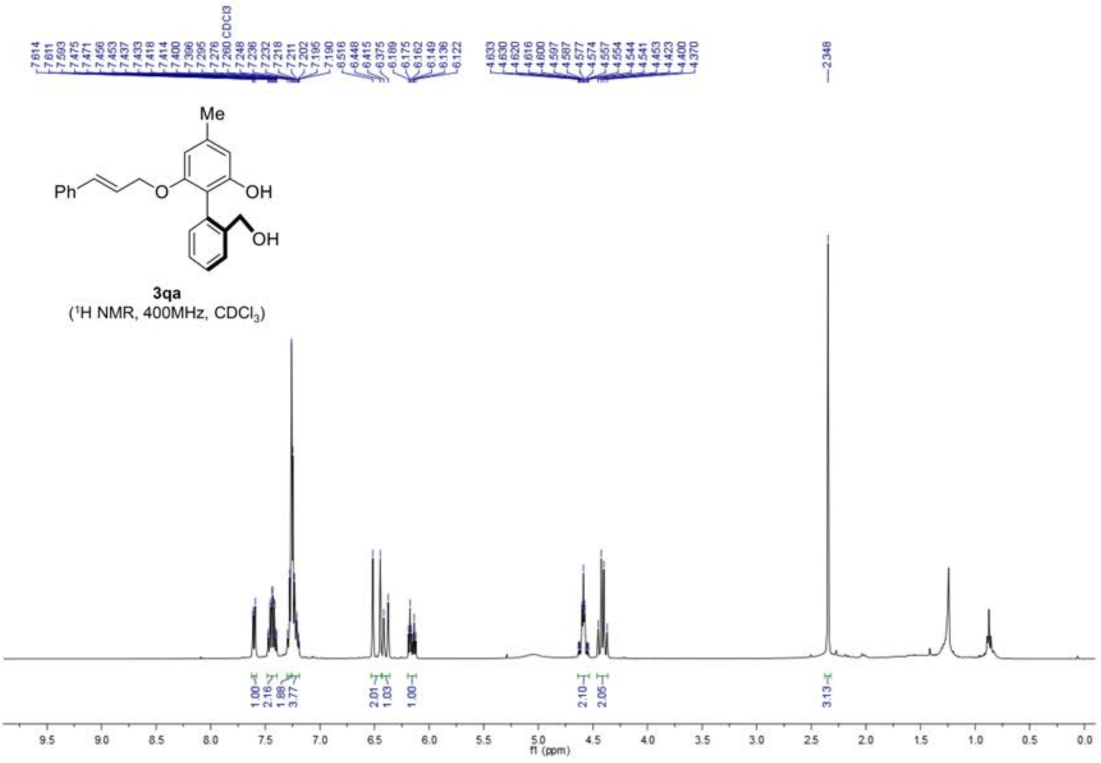
Me
Ph
O
OH
OH
3qa
(¹H NMR, 400MHz, CDCl3)
f1 (ppm)

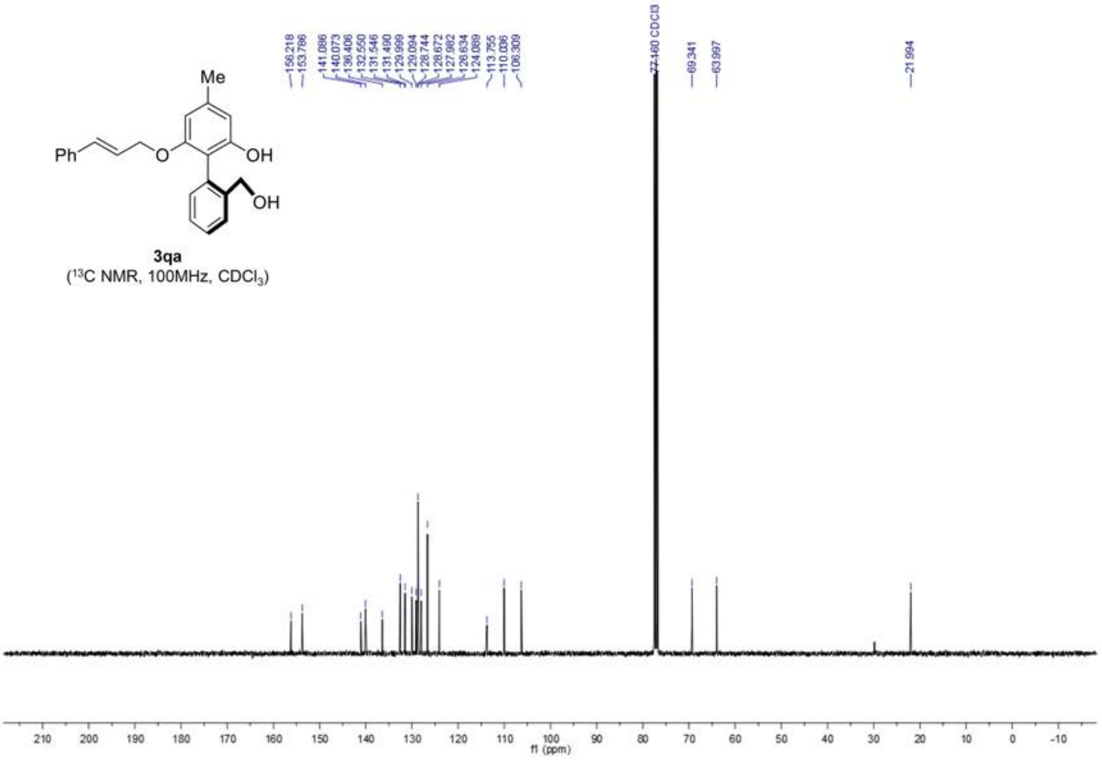
Me
Ph
O
OH
OH
3qa
(¹³C NMR, 100MHz, CDCl3)
f1 (ppm)

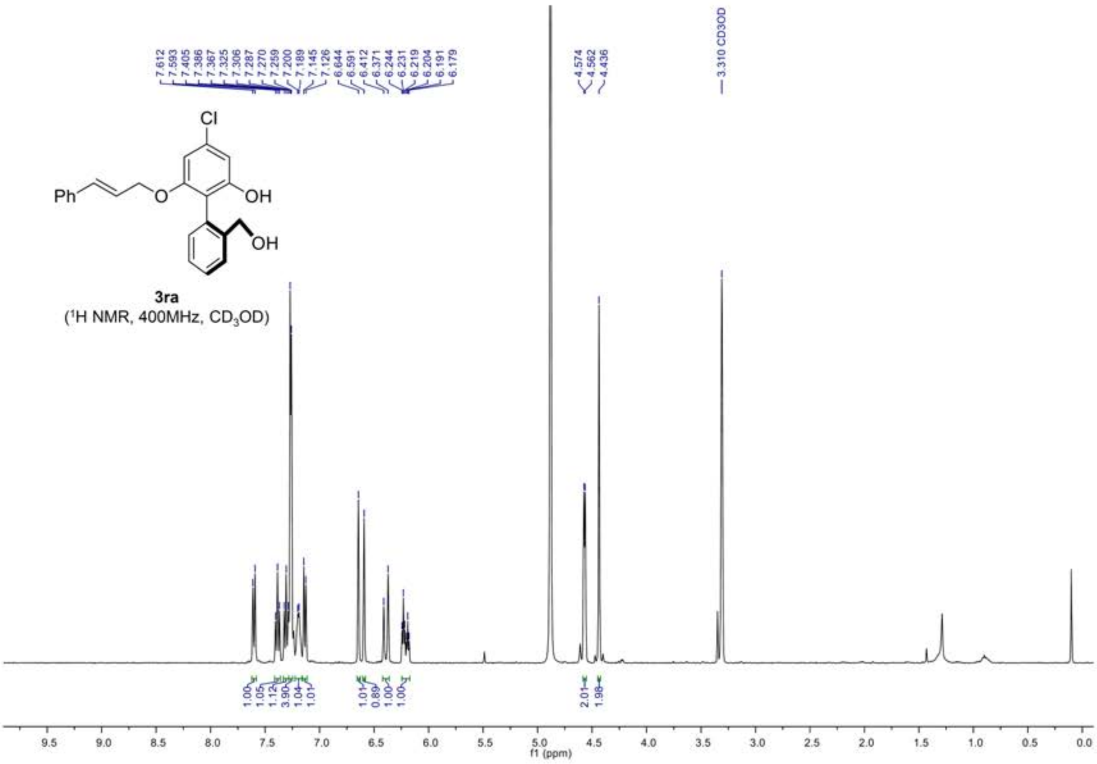
3ra
(¹H NMR, 400MHz, CD3OD)
3.310 CD3OD
f1 (ppm)

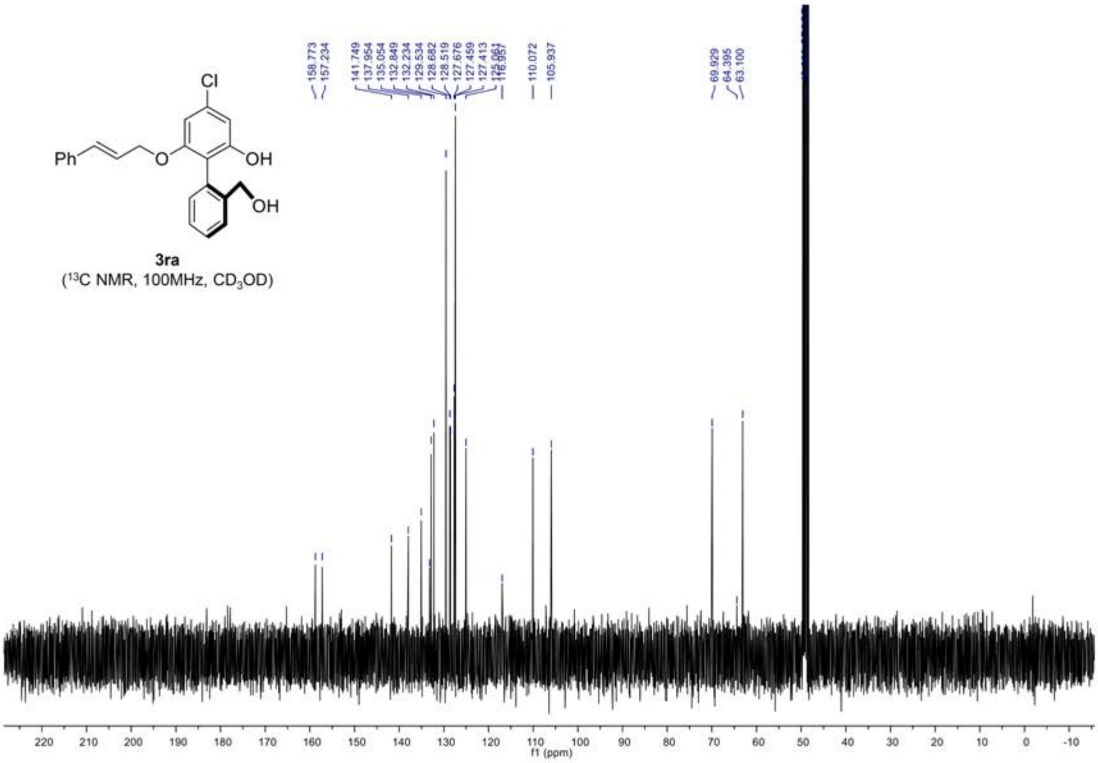
3ra
(¹³C NMR, 100MHz, CD3OD)
f1 (ppm)

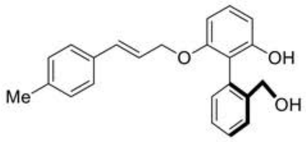

3ab
(^{1}H NMR, 400MHz, $CDCl_3$)

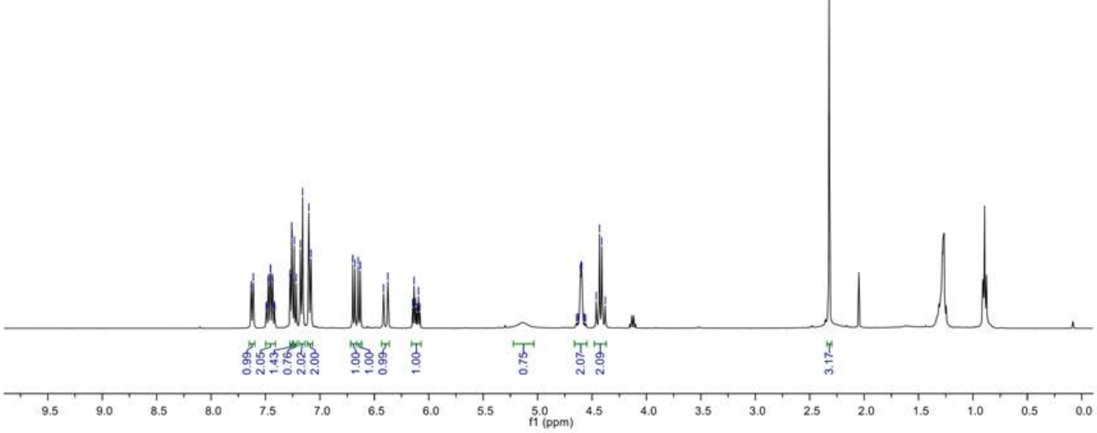

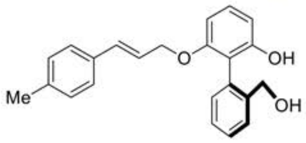

3ab
(^{13}C NMR, 100MHz, $CDCl_3$)

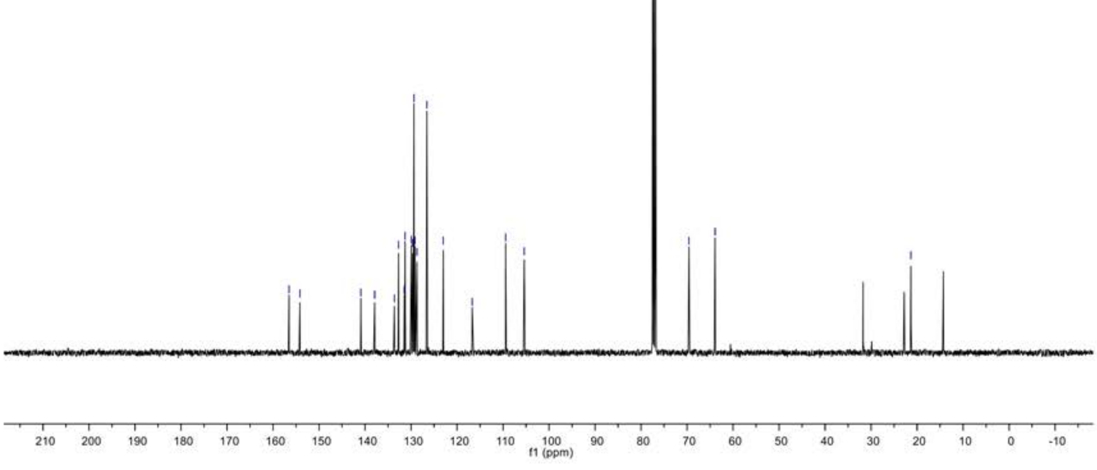

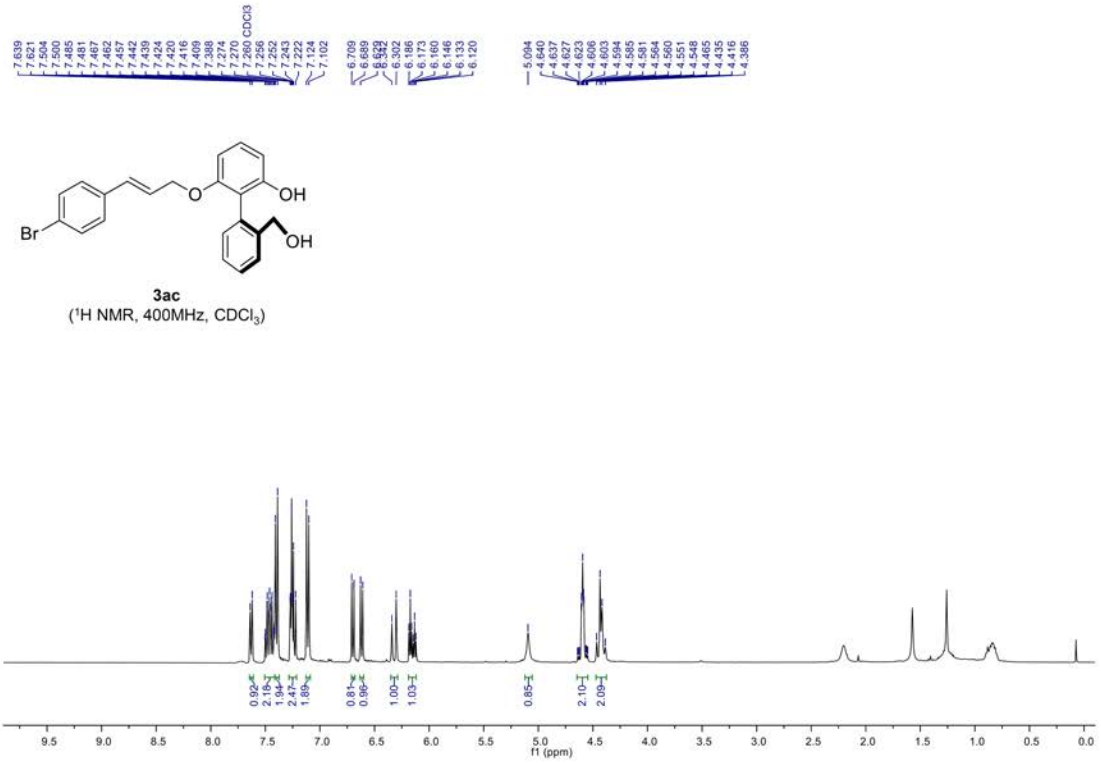

3ac
(^{1}H NMR, 400MHz, $CDCl_3$)

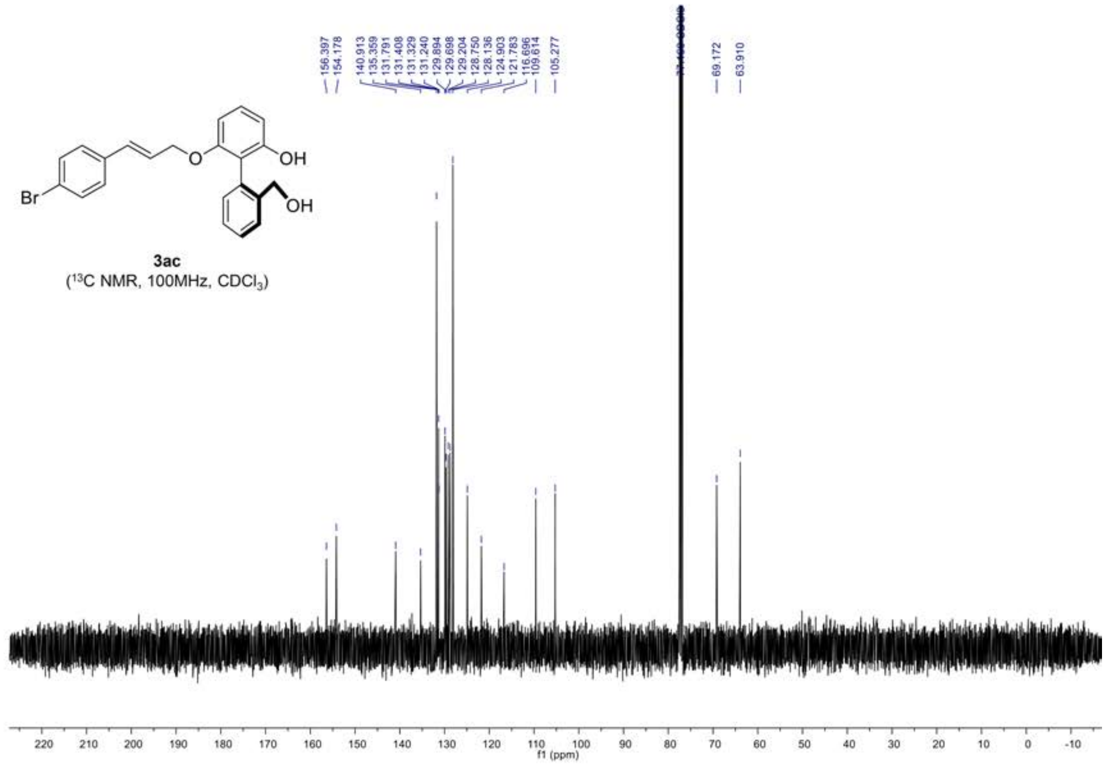

3ac
(^{13}C NMR, 100MHz, $CDCl_3$)

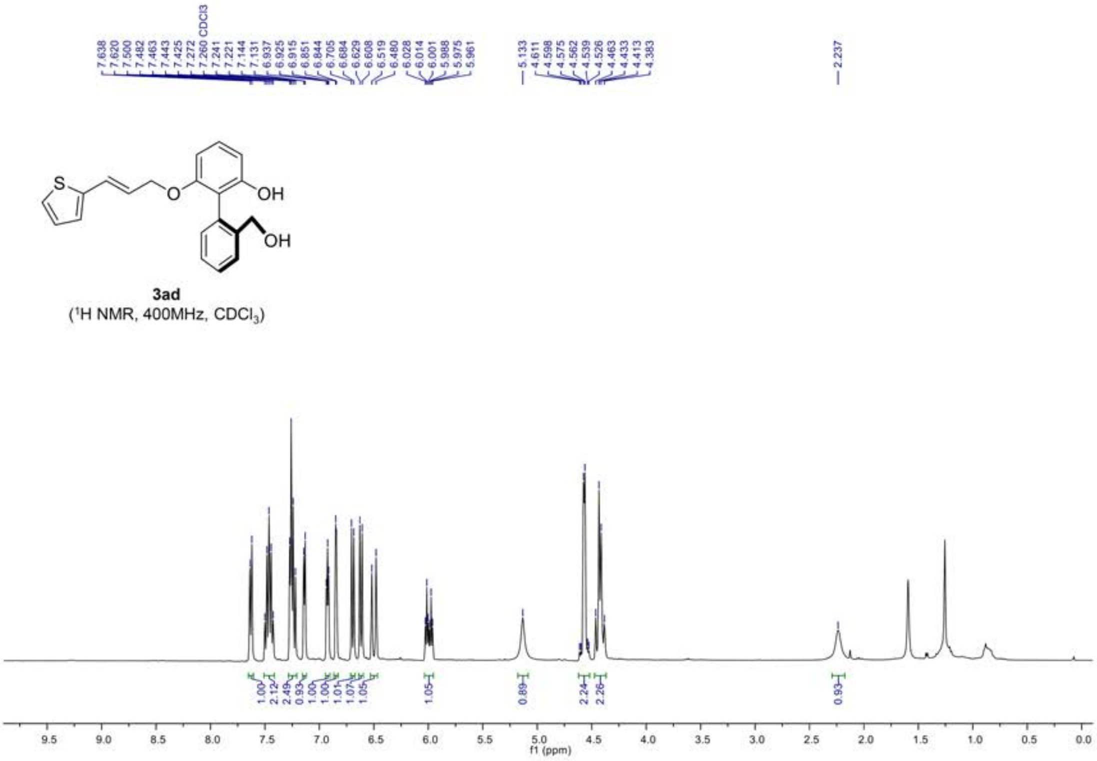

7.638 7.620 7.500 7.482 7.463 7.443 7.425 7.272 7.260 CDCl3 7.241 7.221 7.144 7.131 6.937 6.925 6.915 6.851 6.844 6.705 6.684 6.629 6.608 6.519 6.480 6.028 6.014 6.001 5.988 5.975 5.961
5.133 4.611 4.598 4.575 4.562 4.539 4.526 4.463 4.433 4.413 4.383
2.237
S
O
OH
OH
3ad
(^{1}H NMR, 400MHz, $CDCl_3$)
1.00 2.12 2.49 0.93 1.00 1.00 1.01 1.07 1.05 1.05 0.89 2.24 2.26 0.93
9.5 9.0 8.5 8.0 7.5 7.0 6.5 6.0 5.5 5.0 4.5 4.0 3.5 3.0 2.5 2.0 1.5 1.0 0.5 0.0
f1 (ppm)

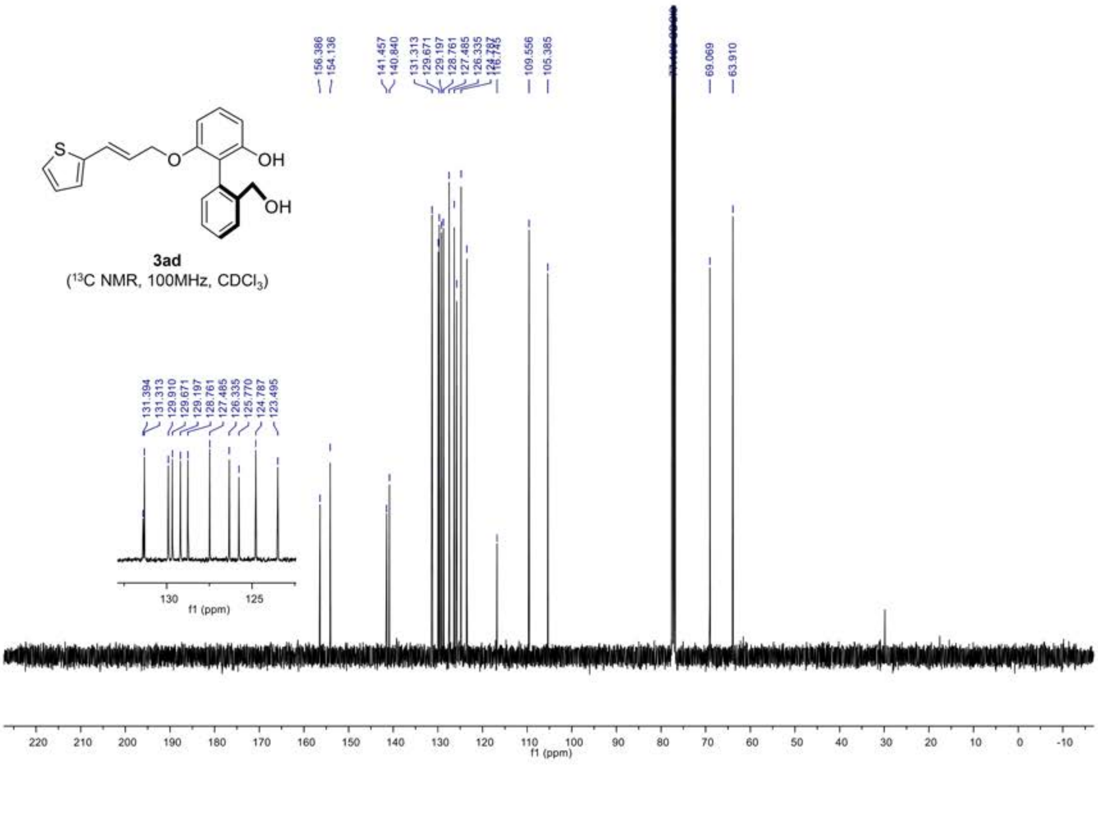

156.386 154.136
141.457 140.840
131.313 129.671 129.197 128.761 127.485 126.335
109.556 105.385
69.069 63.910
S
O
OH
OH
3ad
(^{13}C NMR, 100MHz, $CDCl_3$)
131.394 131.313 129.910 129.671 129.197 128.761 127.485 126.335 125.770 124.787 123.495
130 125
f1 (ppm)
220 210 200 190 180 170 160 150 140 130 120 110 100 90 80 70 60 50 40 30 20 10 0 -10
f1 (ppm)

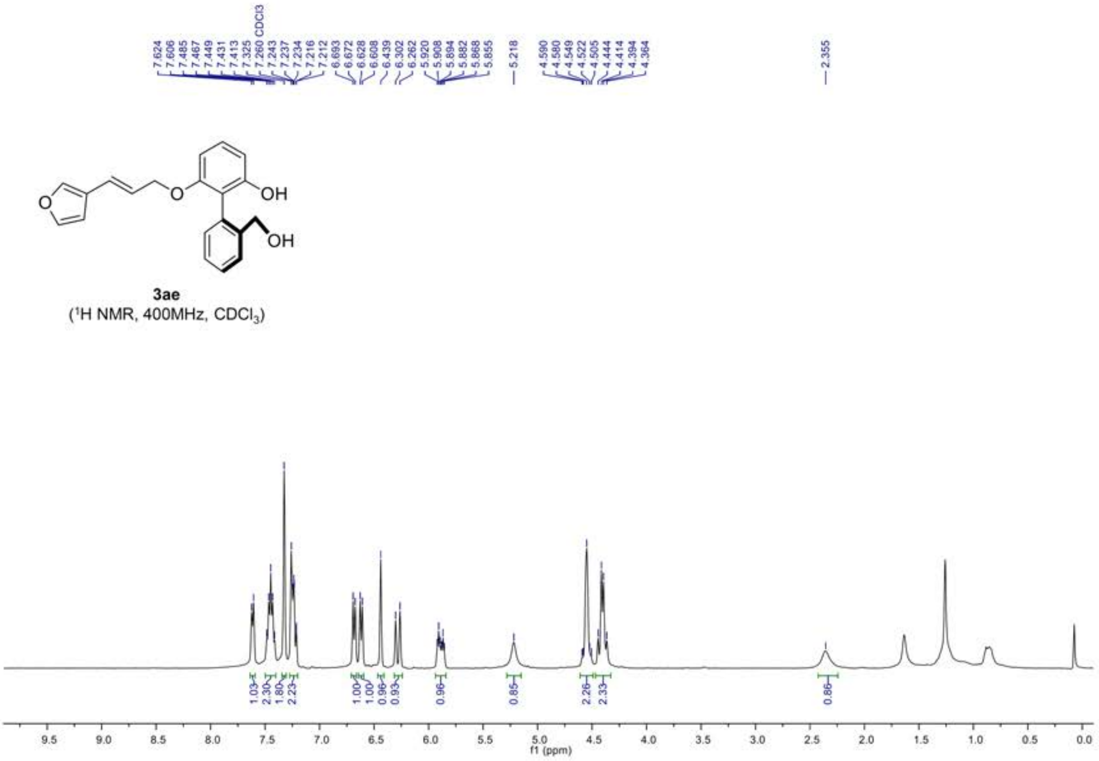

3ae
(^{1}H NMR, 400MHz, $CDCl_3$)

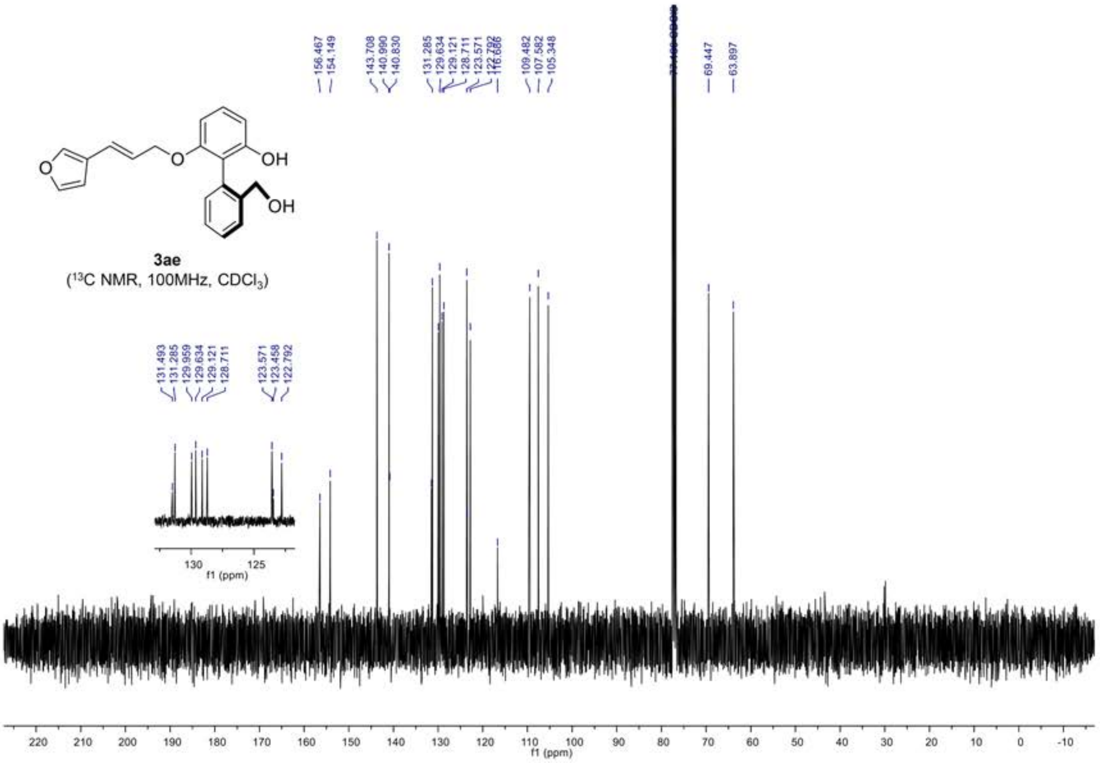

3ae
(^{13}C NMR, 100MHz, $CDCl_3$)

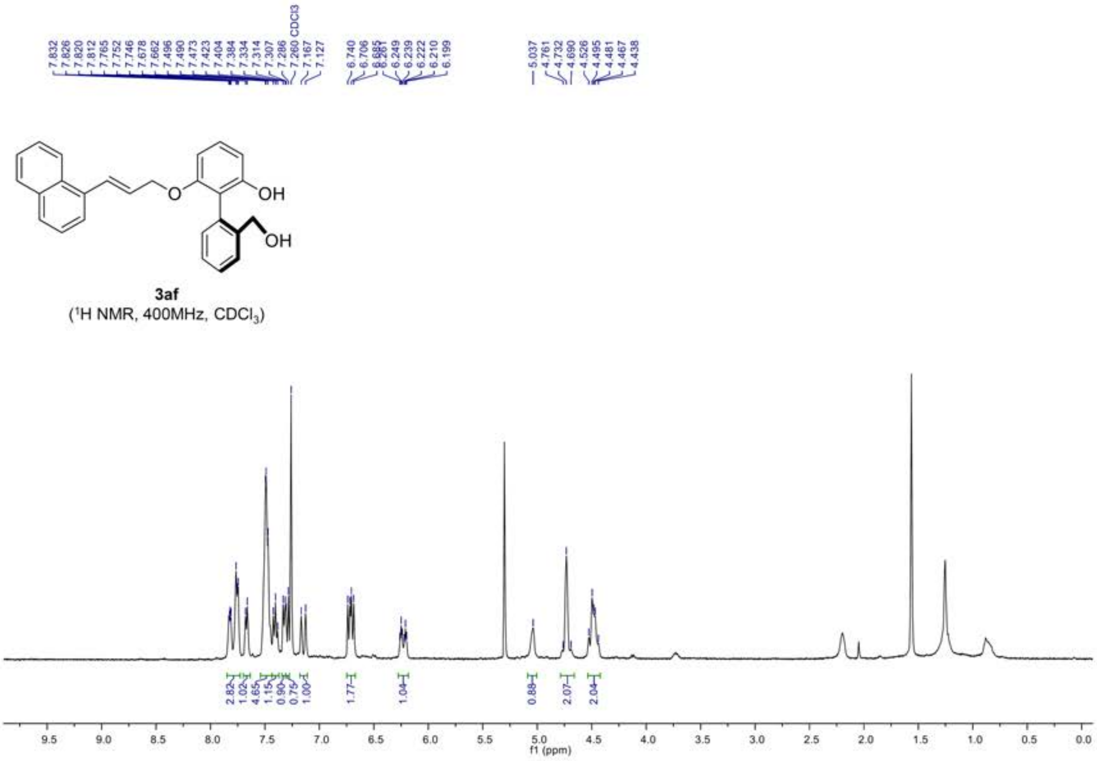

3af
(^{1}H NMR, 400MHz, $CDCl_3$)

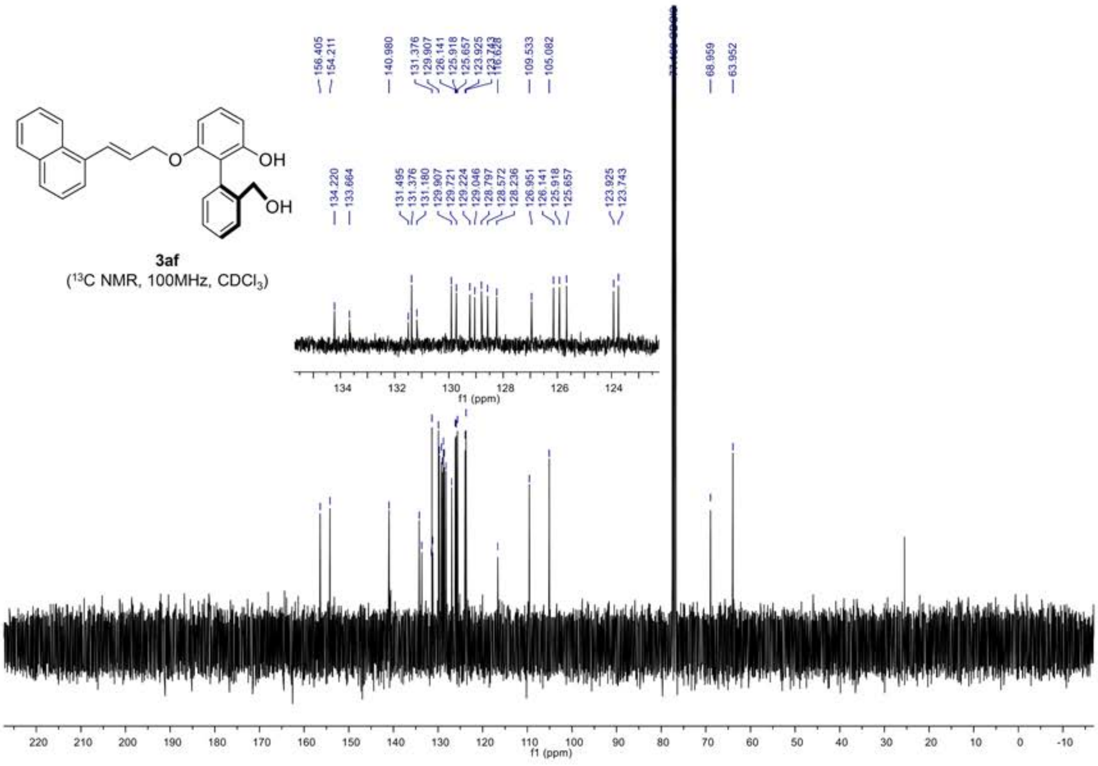

3af
(^{13}C NMR, 100MHz, $CDCl_3$)

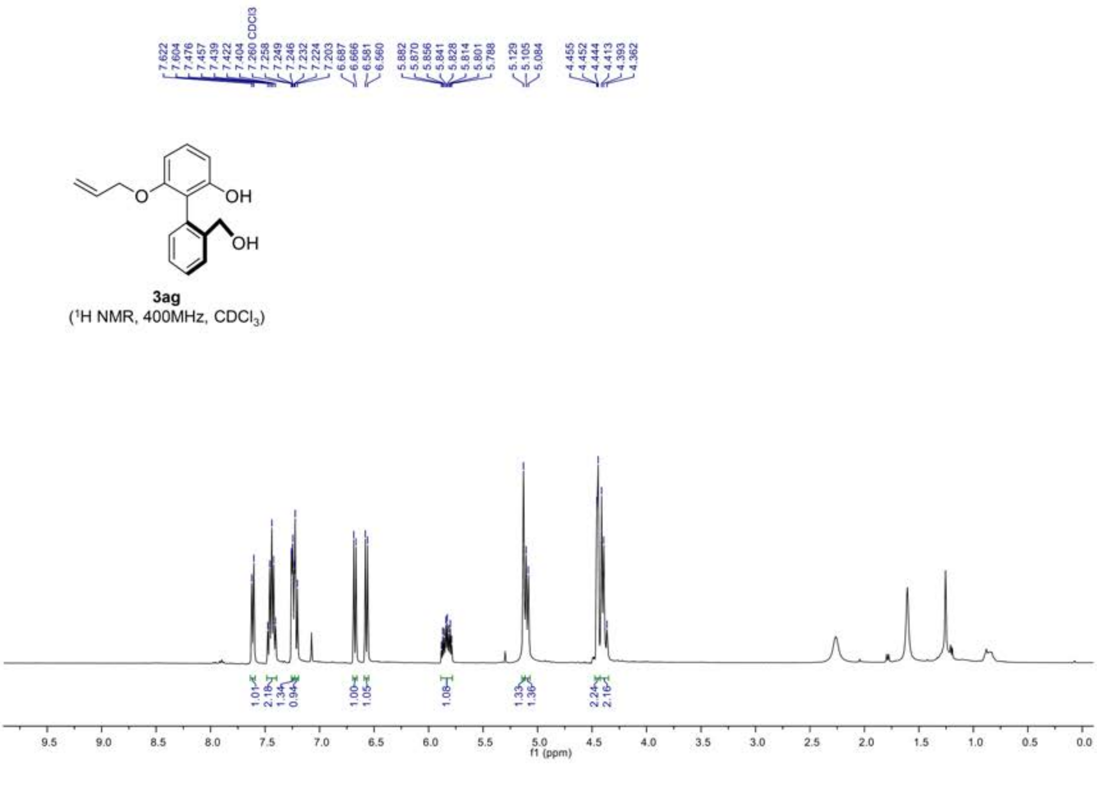
3ag
(¹H NMR, 400MHz, CDCl₃)
OH
OH
O
f1 (ppm)

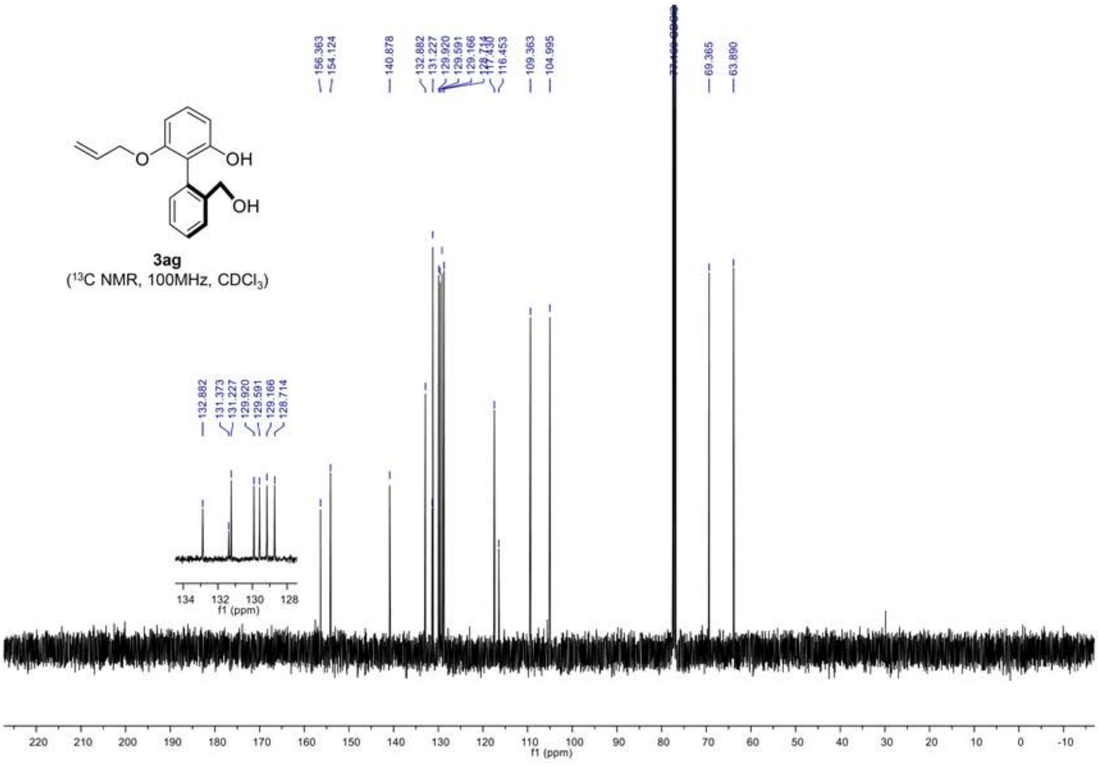
3ag
(¹³C NMR, 100MHz, CDCl₃)
OH
OH
O
f1 (ppm)

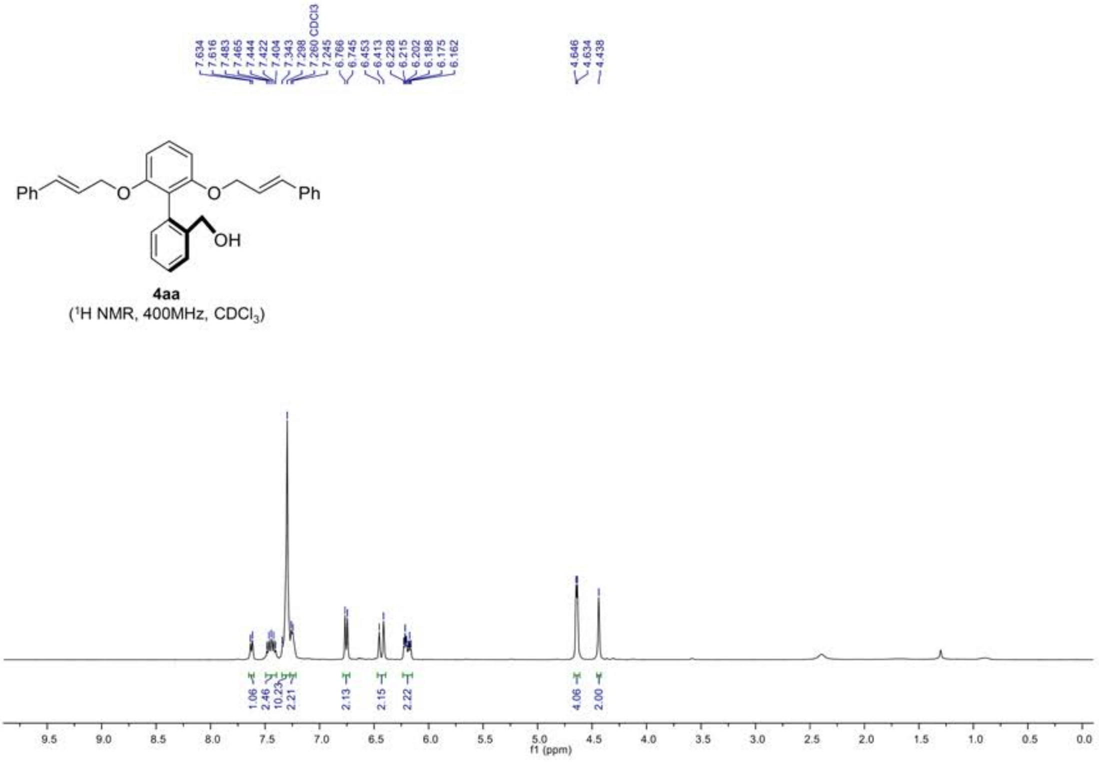

4aa
(^{1}H NMR, 400MHz, $CDCl_3$)

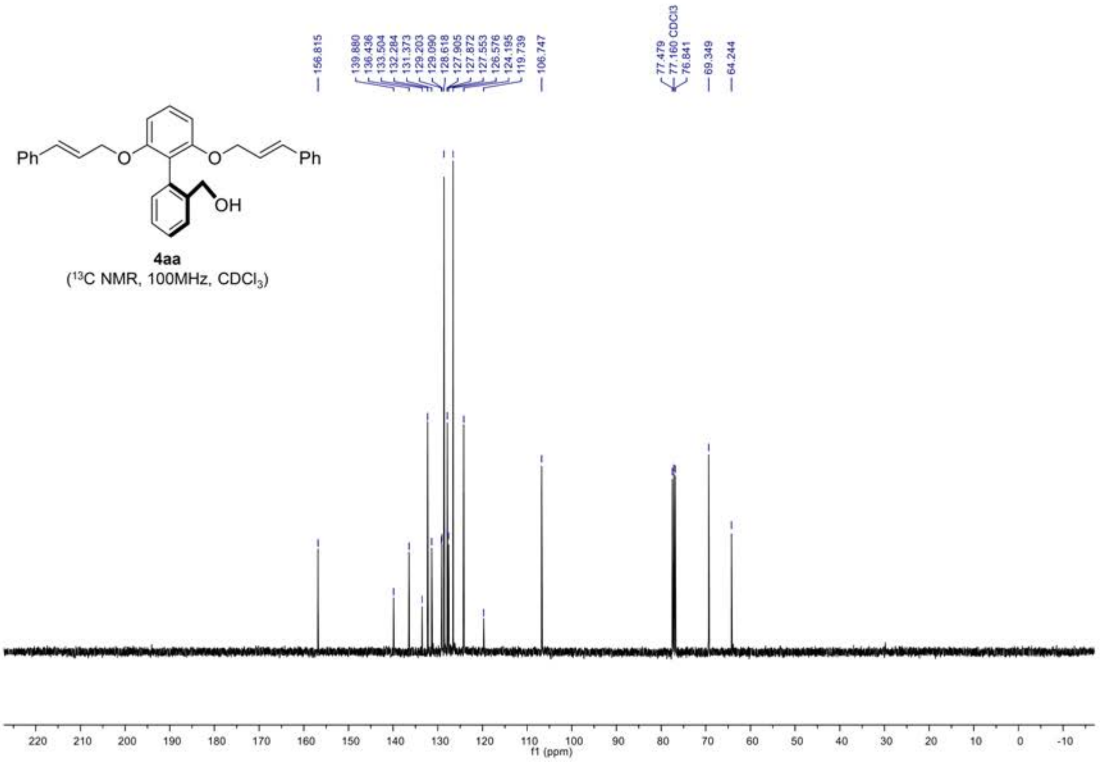

4aa
(^{13}C NMR, 100MHz, $CDCl_3$)

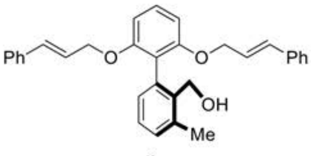

4ba
(^{1}H NMR, 400MHz, $CDCl_3$)

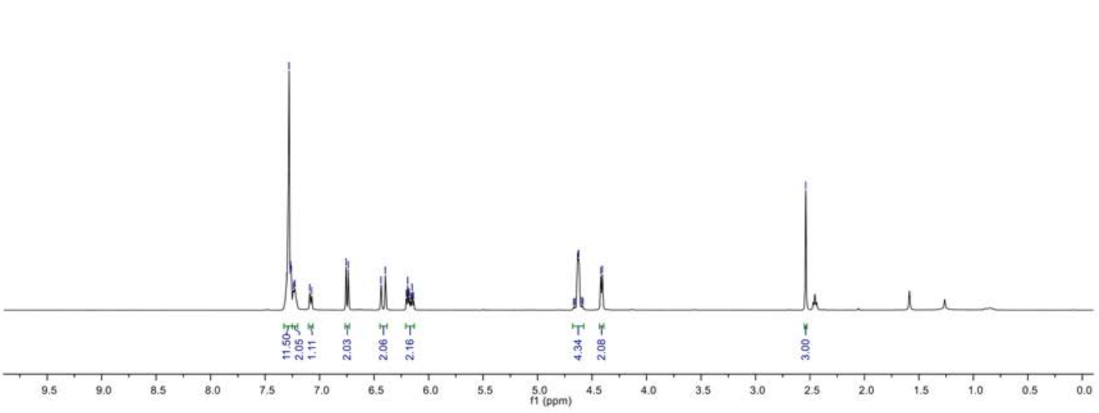

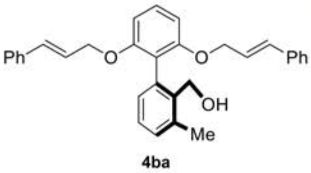

4ba
(^{13}C NMR, 100MHz, $CDCl_3$)

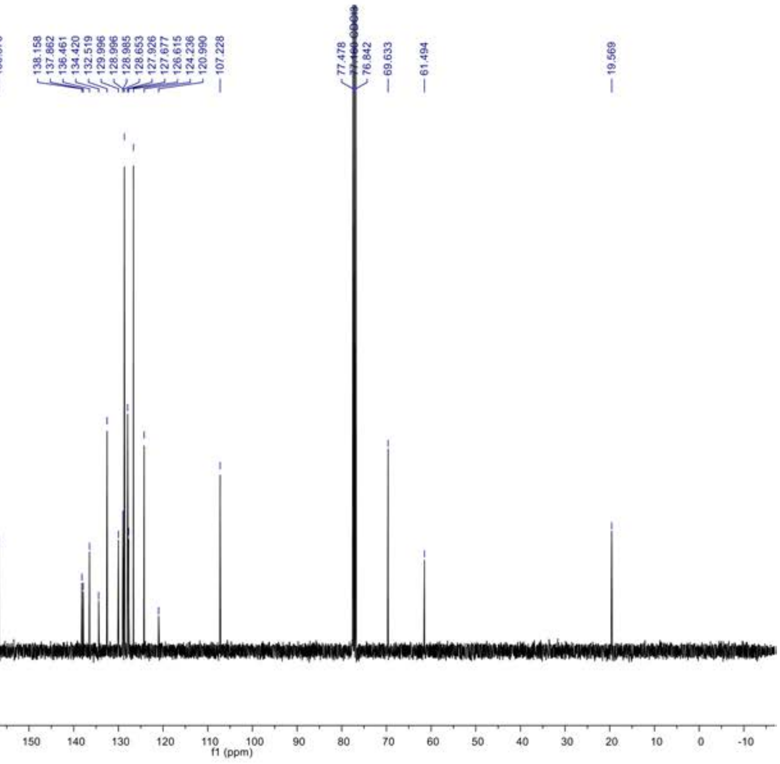

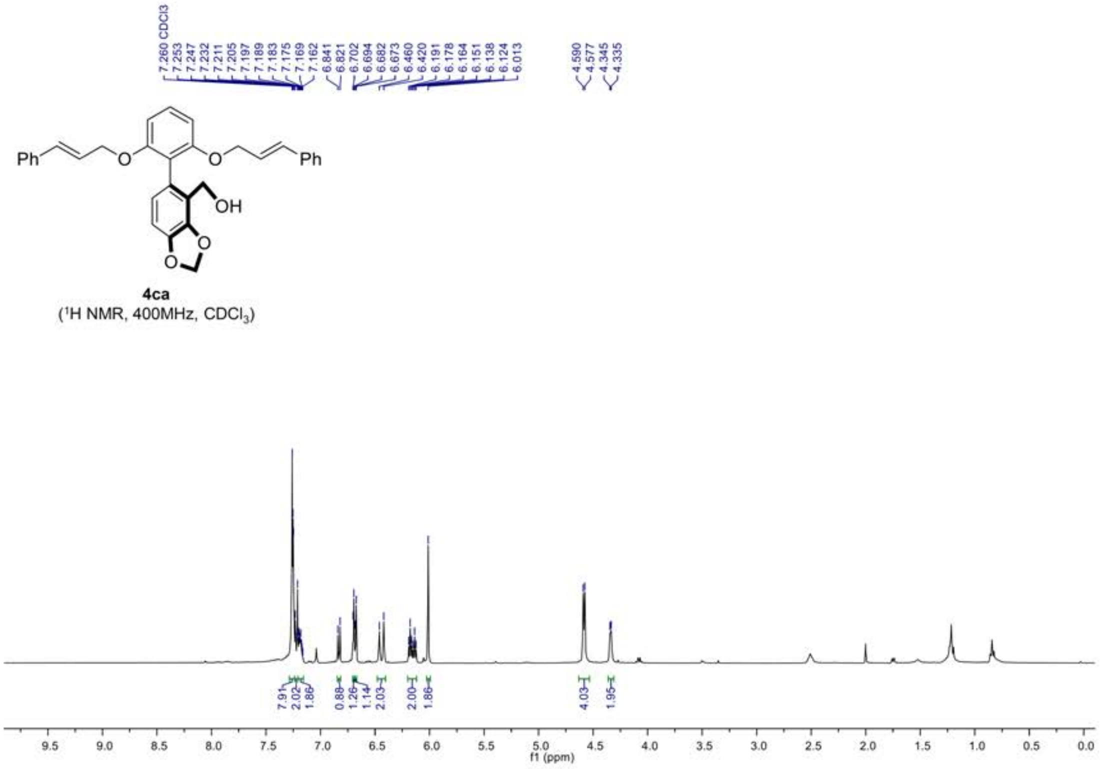

4ca
(^{1}H NMR, 400MHz, $CDCl_3$)

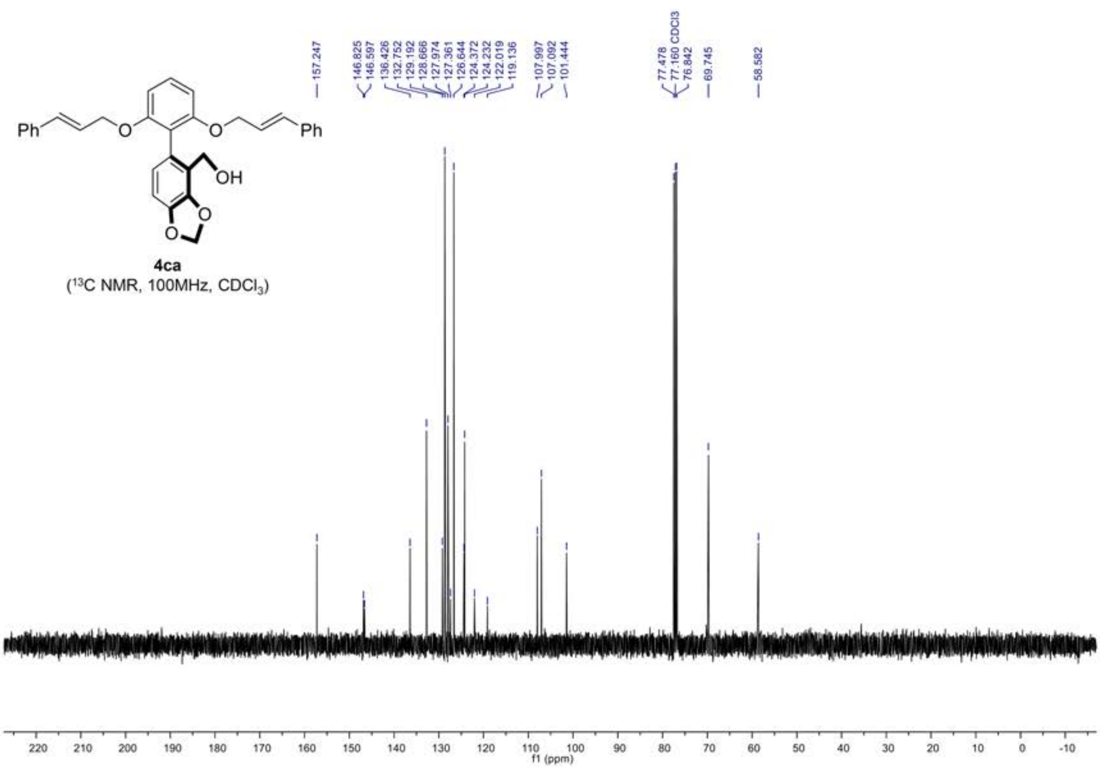

4ca
(^{13}C NMR, 100MHz, $CDCl_3$)

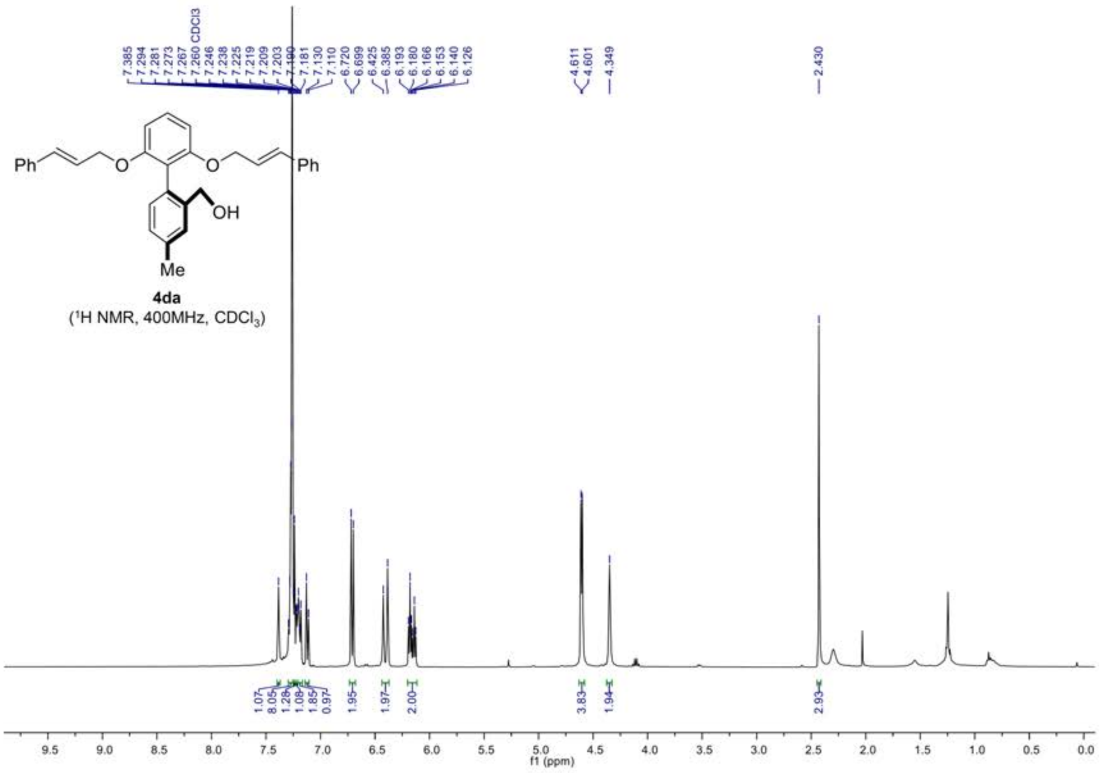

4da
(^{1}H NMR, 400MHz, $CDCl_3$)

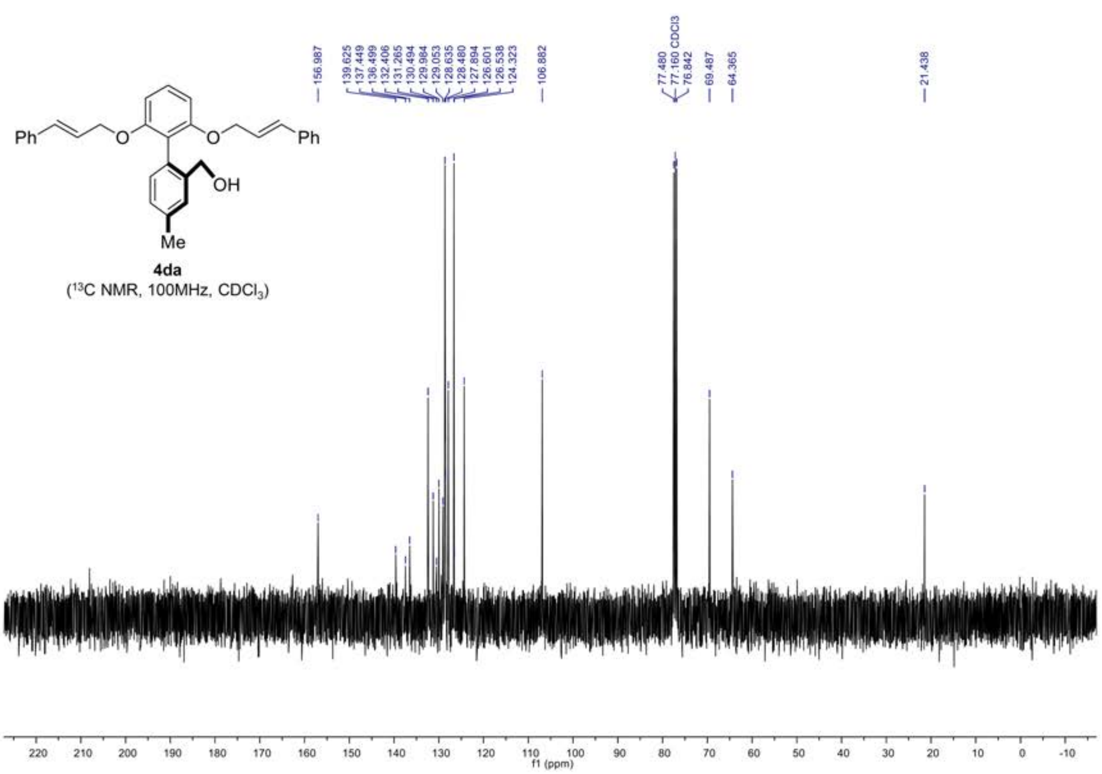

4da
(^{13}C NMR, 100MHz, $CDCl_3$)

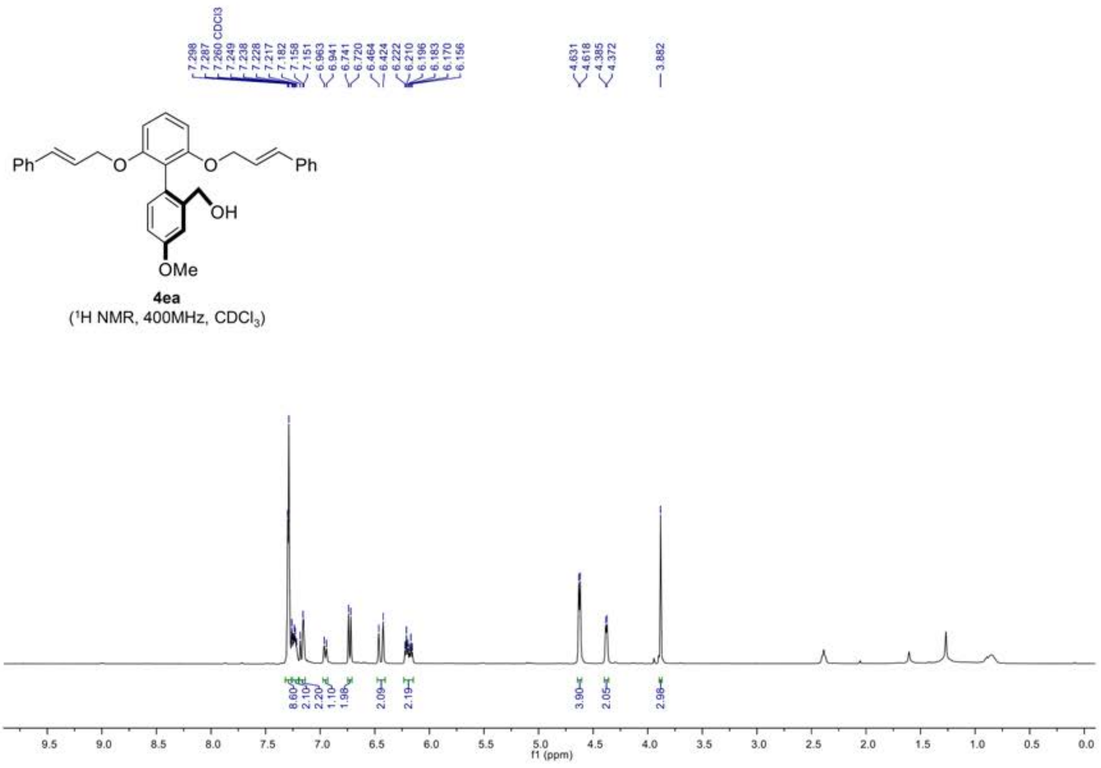

4ea
(^{1}H NMR, 400MHz, $CDCl_3$)

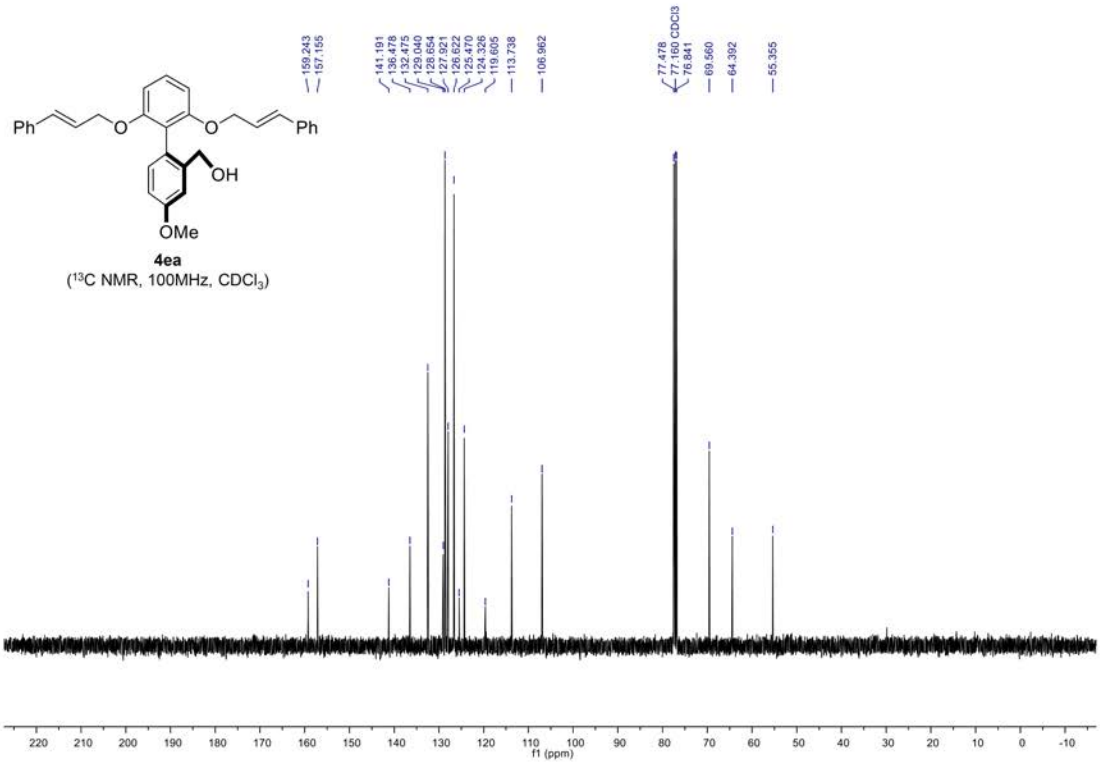

4ea
(^{13}C NMR, 100MHz, $CDCl_3$)

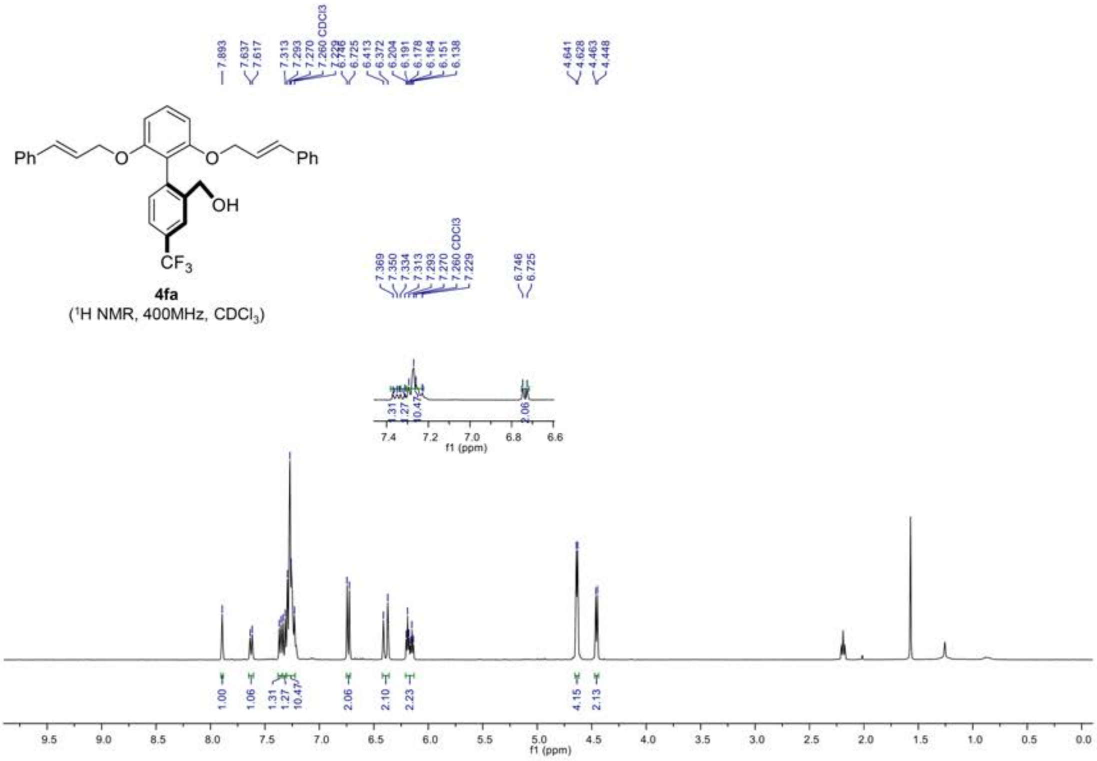

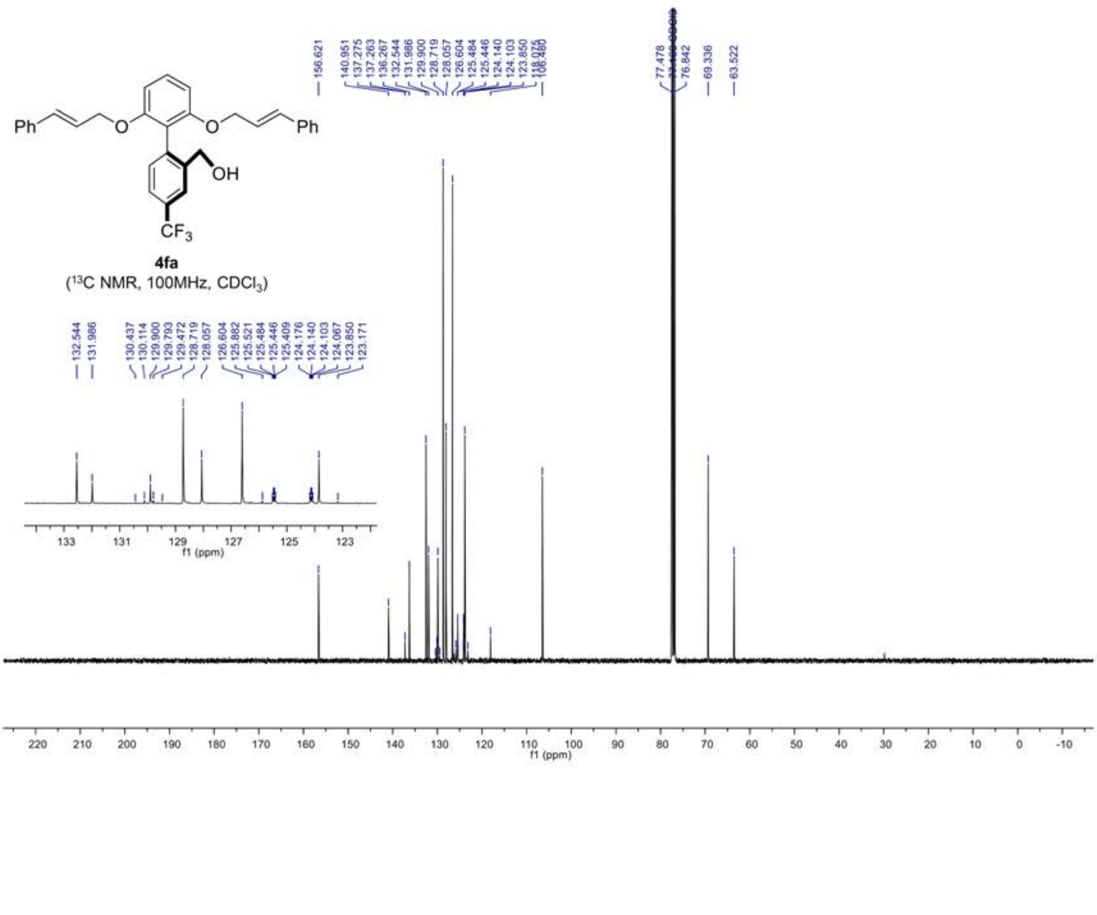

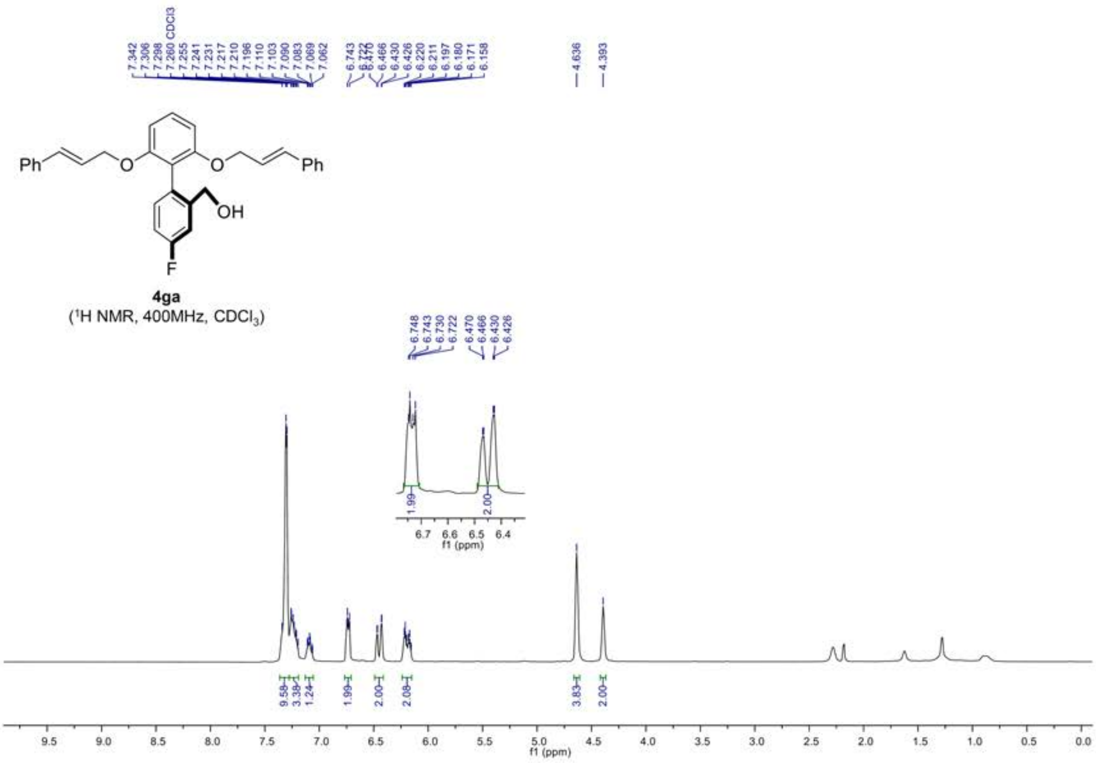

4ga
(^{1}H NMR, 400MHz, $CDCl_3$)

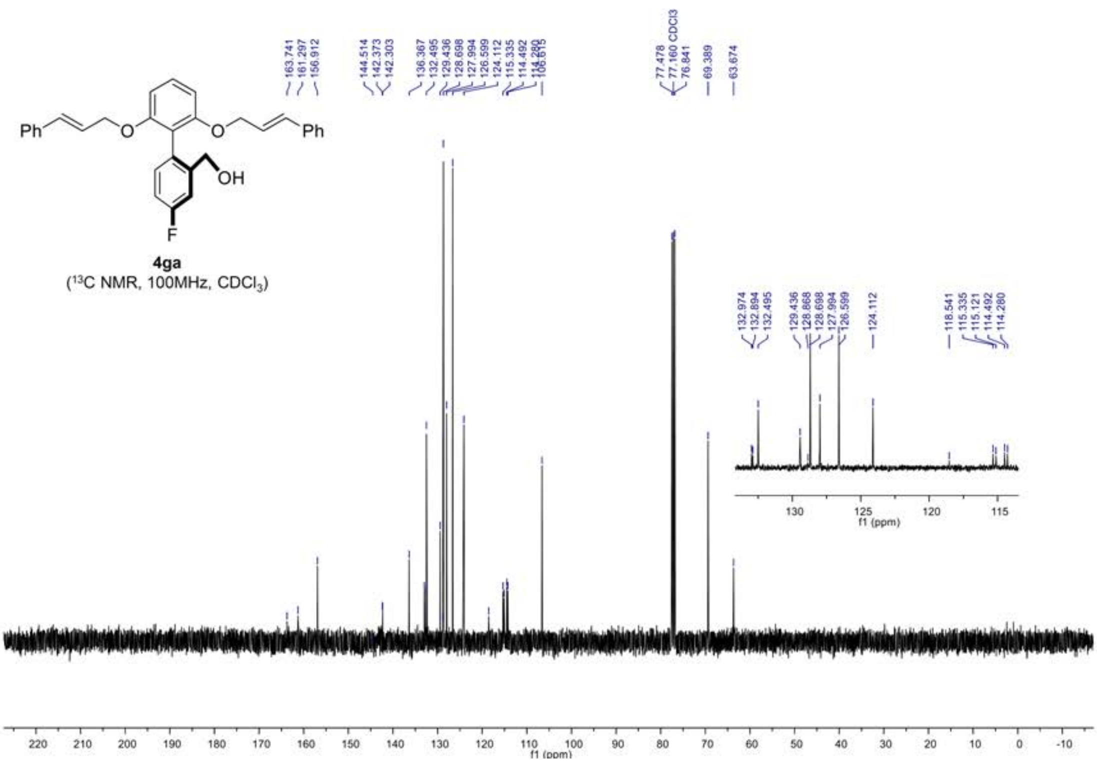

4ga
(^{13}C NMR, 100MHz, $CDCl_3$)

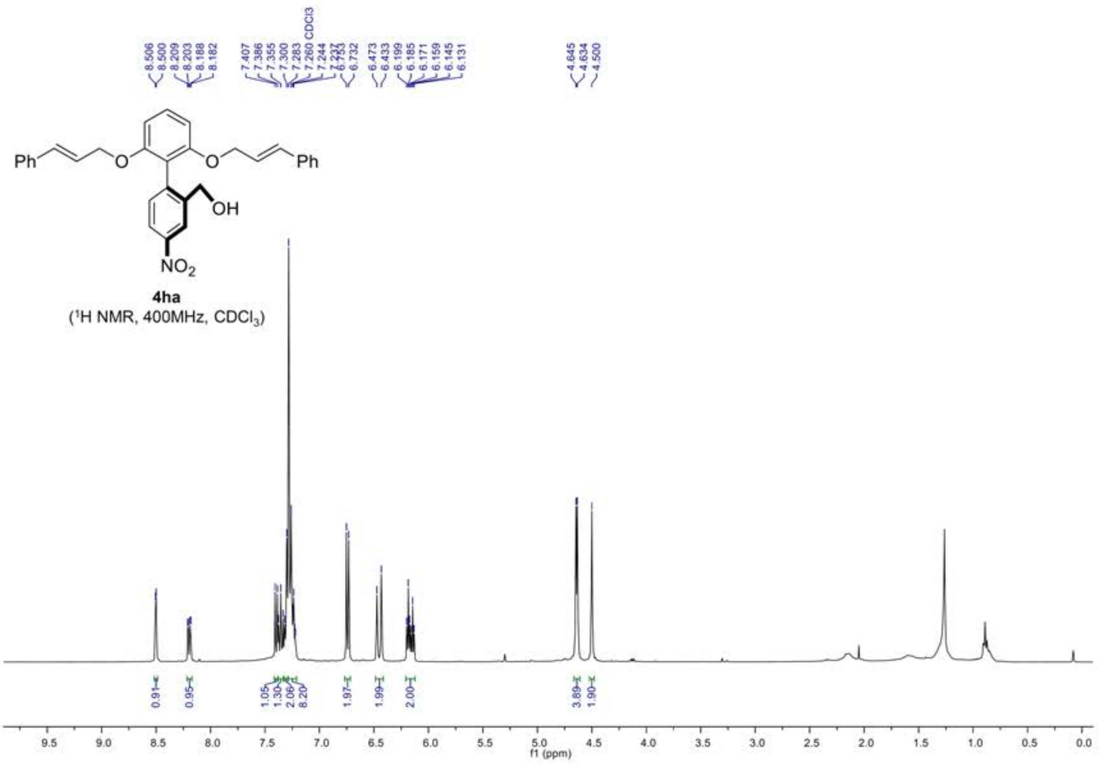

4ha
(^{1}H NMR, 400MHz, $CDCl_3$)

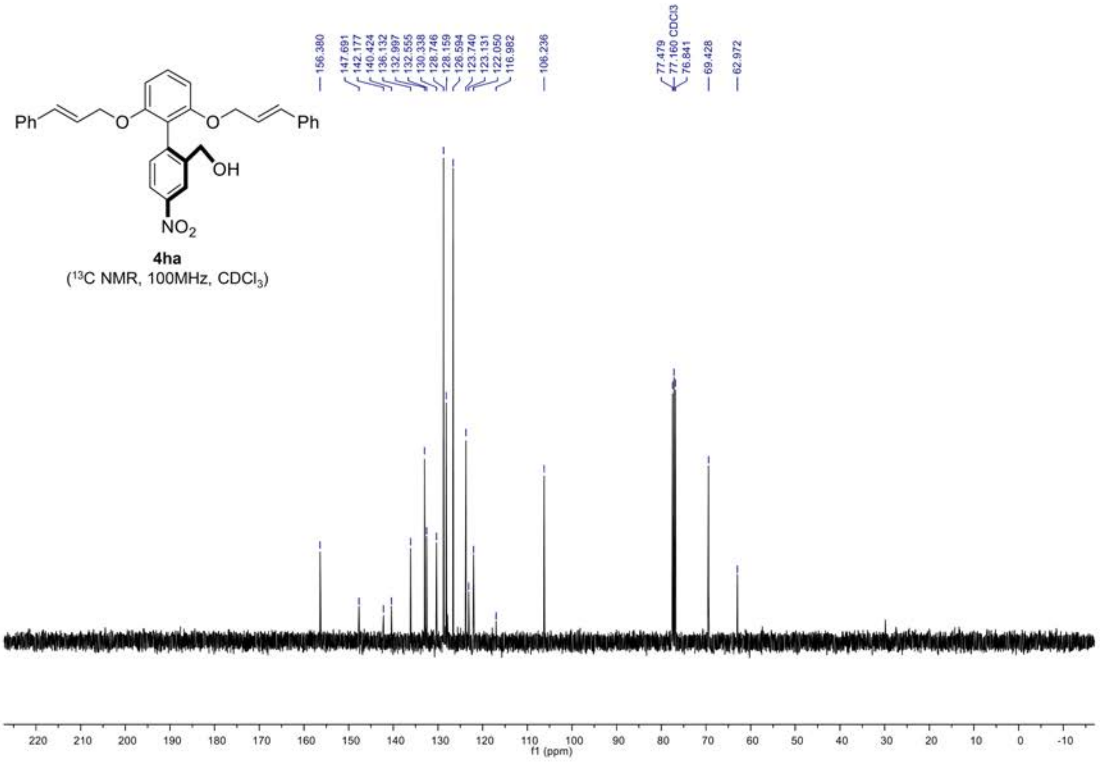

4ha
(^{13}C NMR, 100MHz, $CDCl_3$)

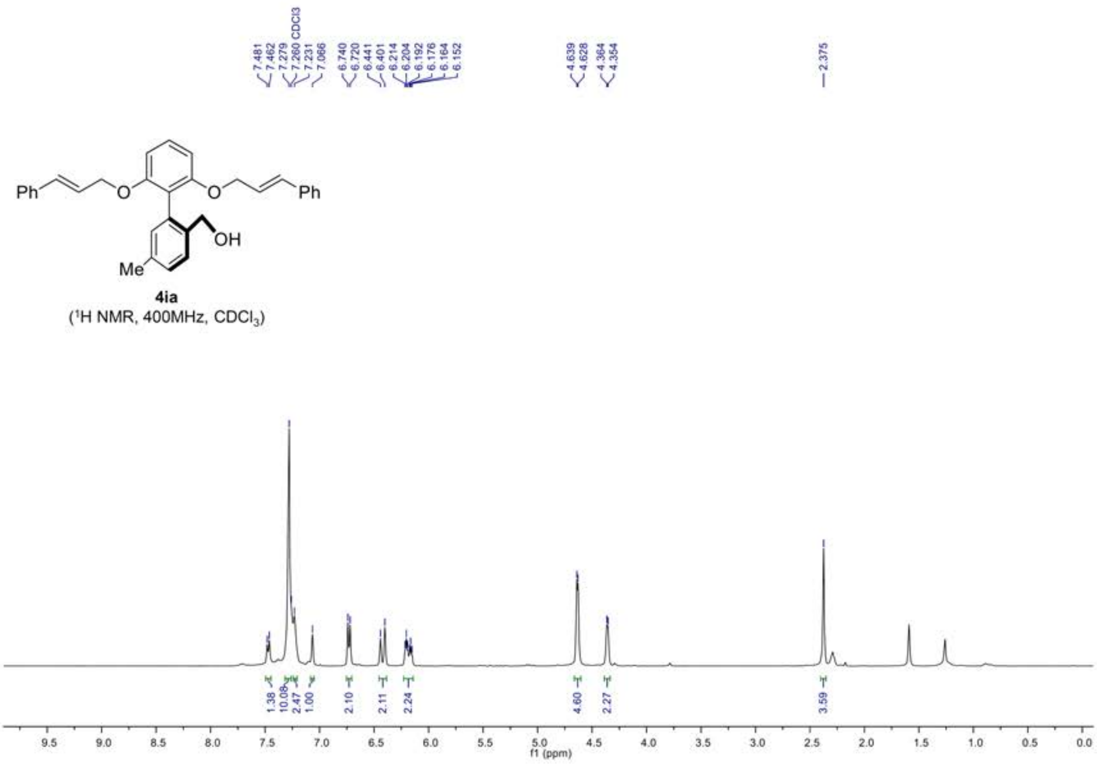

4ia
(^{1}H NMR, 400MHz, $CDCl_3$)

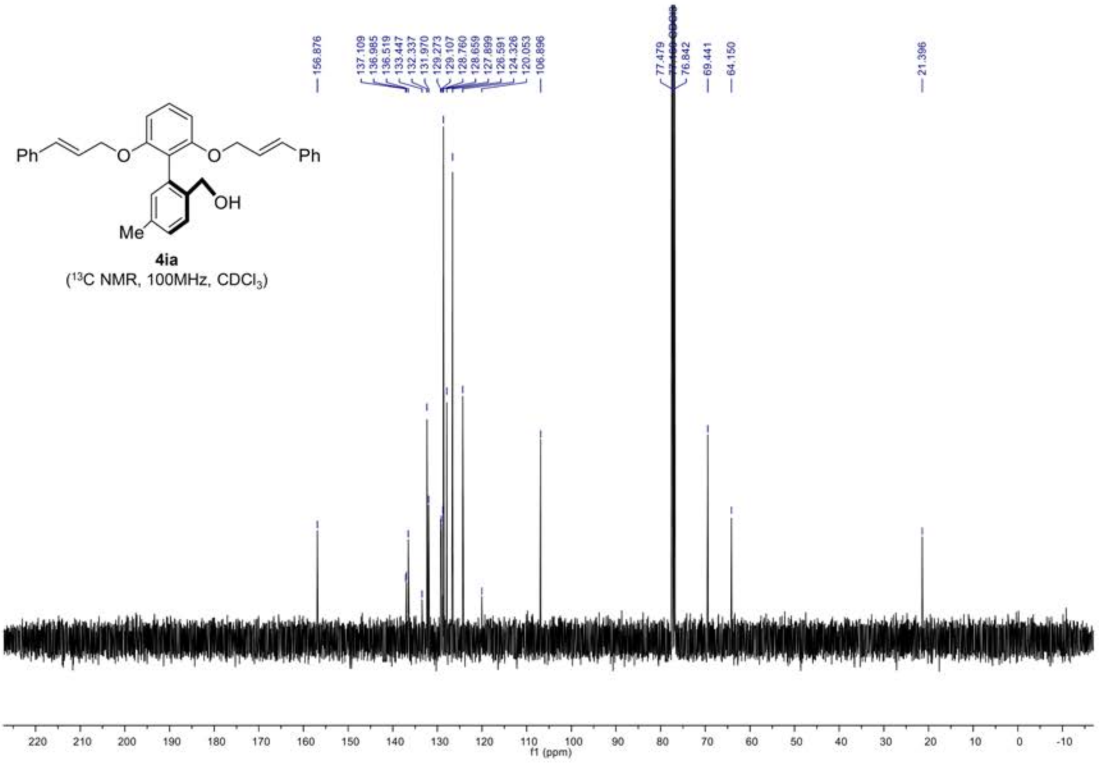

4ia
(^{13}C NMR, 100MHz, $CDCl_3$)

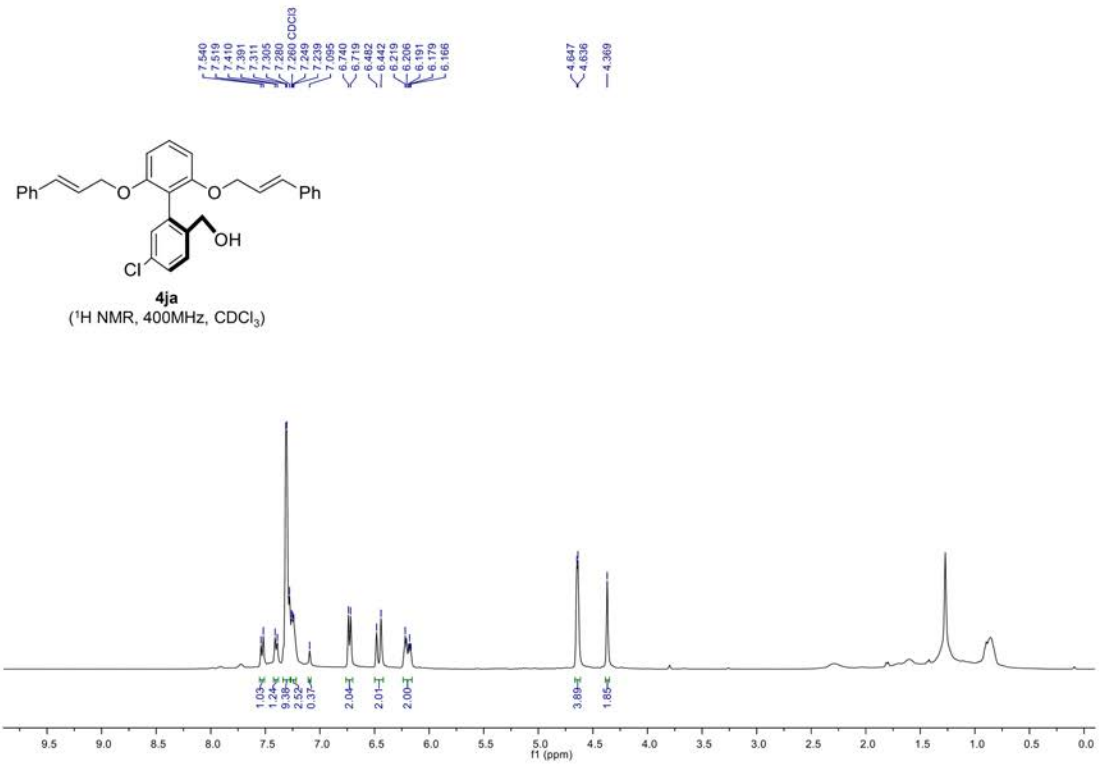

4ja
(^{1}H NMR, 400MHz, $CDCl_3$)

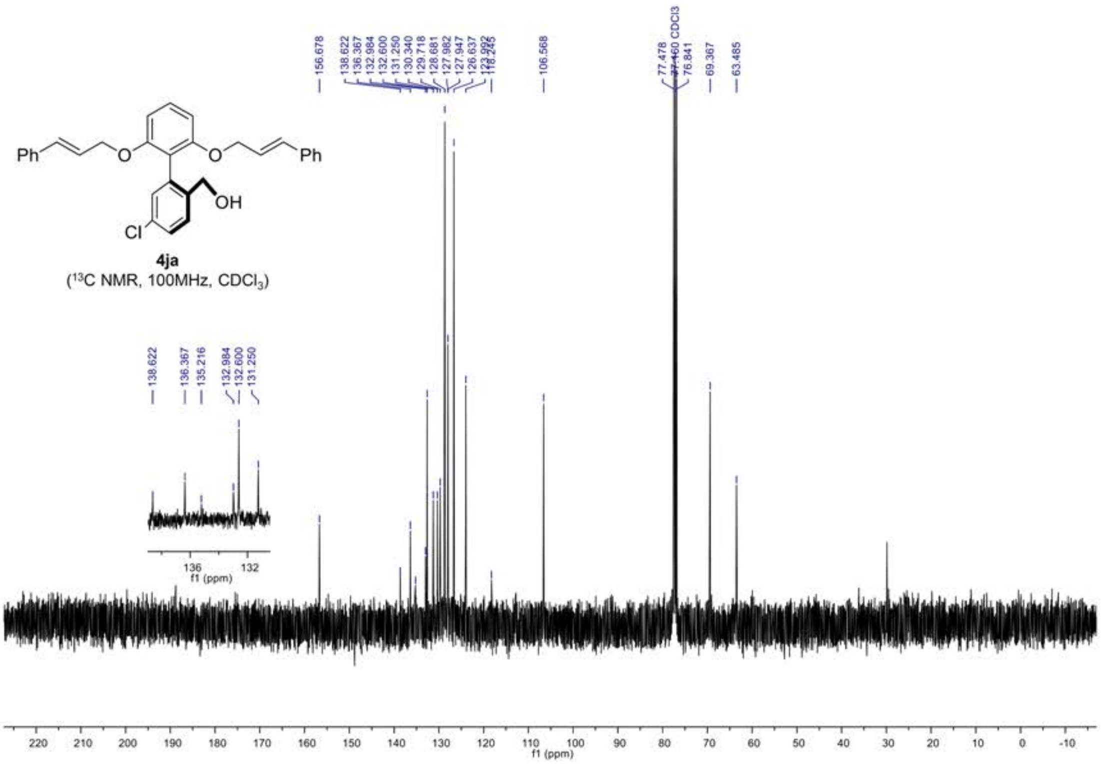

4ja
(^{13}C NMR, 100MHz, $CDCl_3$)

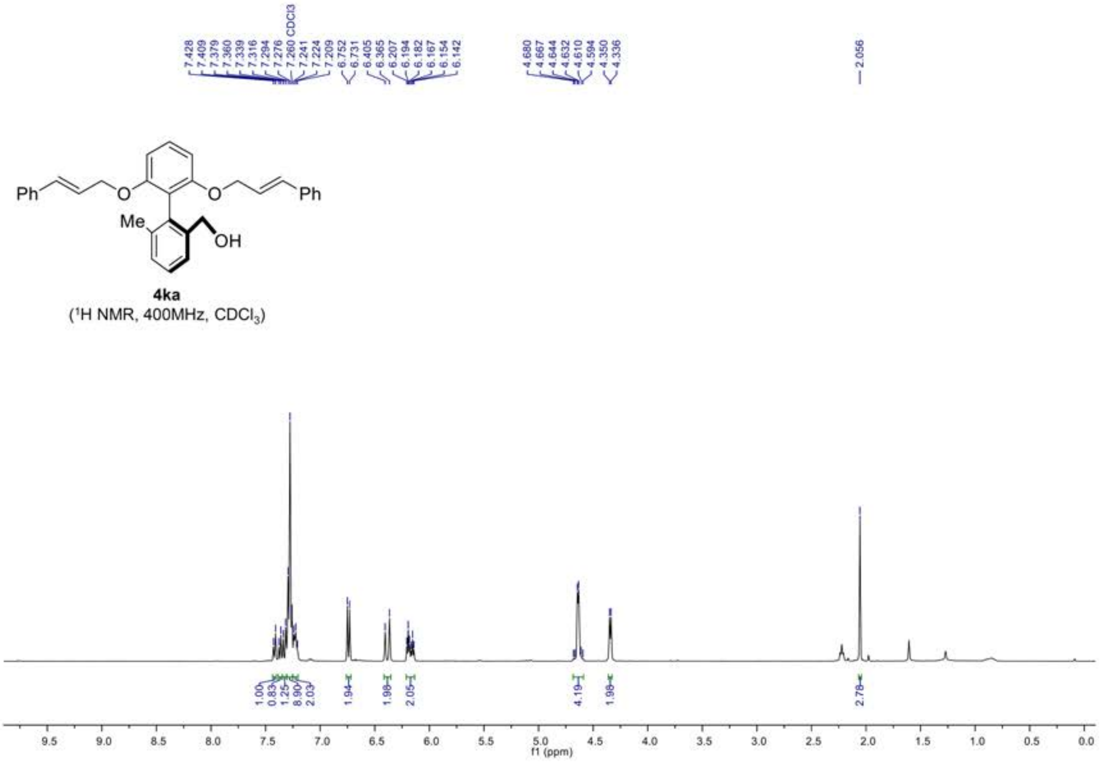

4ka
(^{1}H NMR, 400MHz, $CDCl_3$)

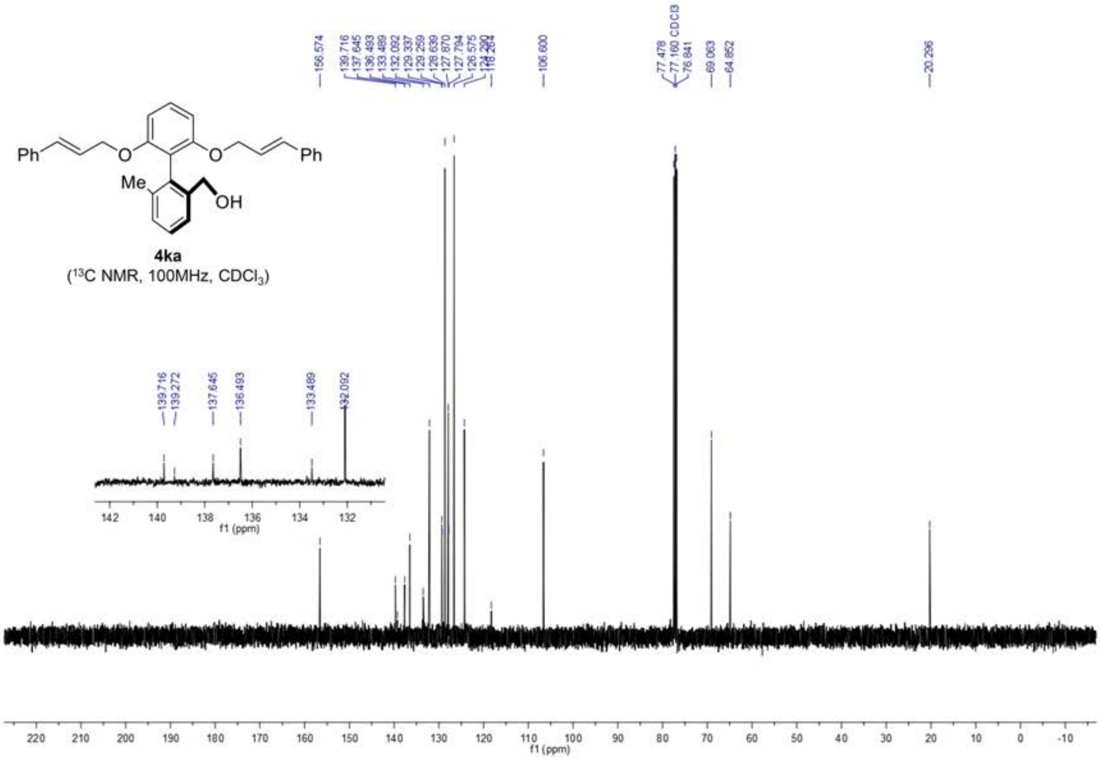

4ka
(^{13}C NMR, 100MHz, $CDCl_3$)

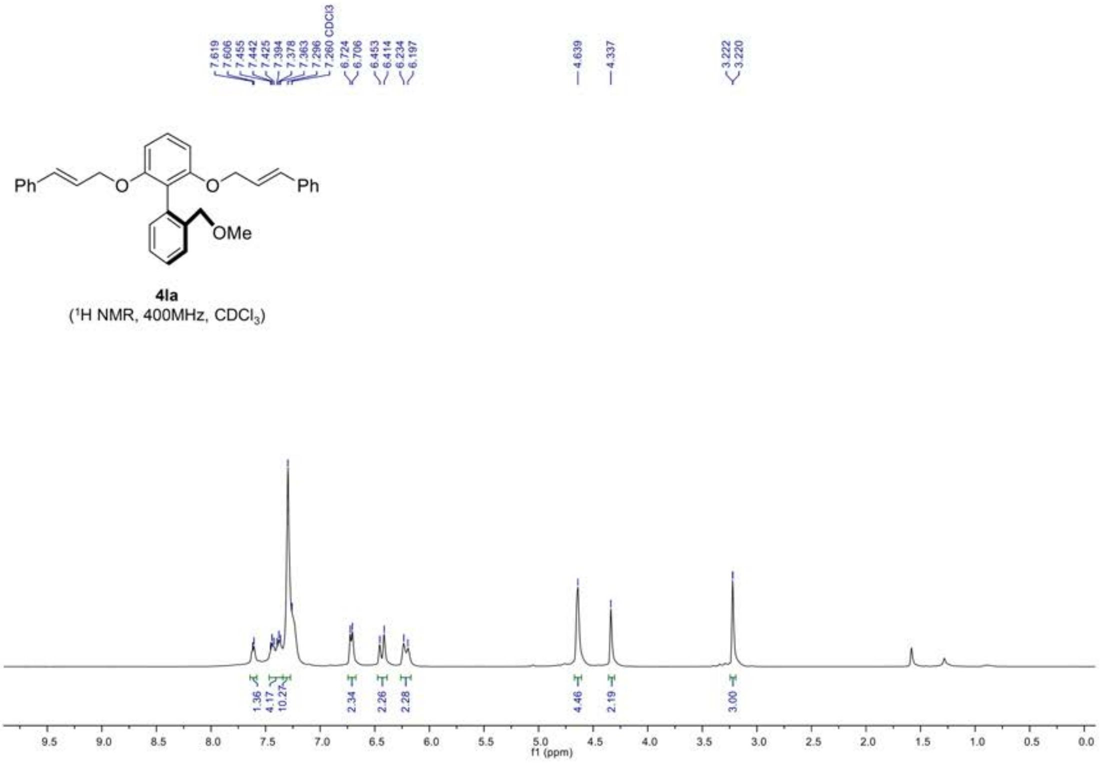

4la
(^{1}H NMR, 400MHz, $CDCl_3$)

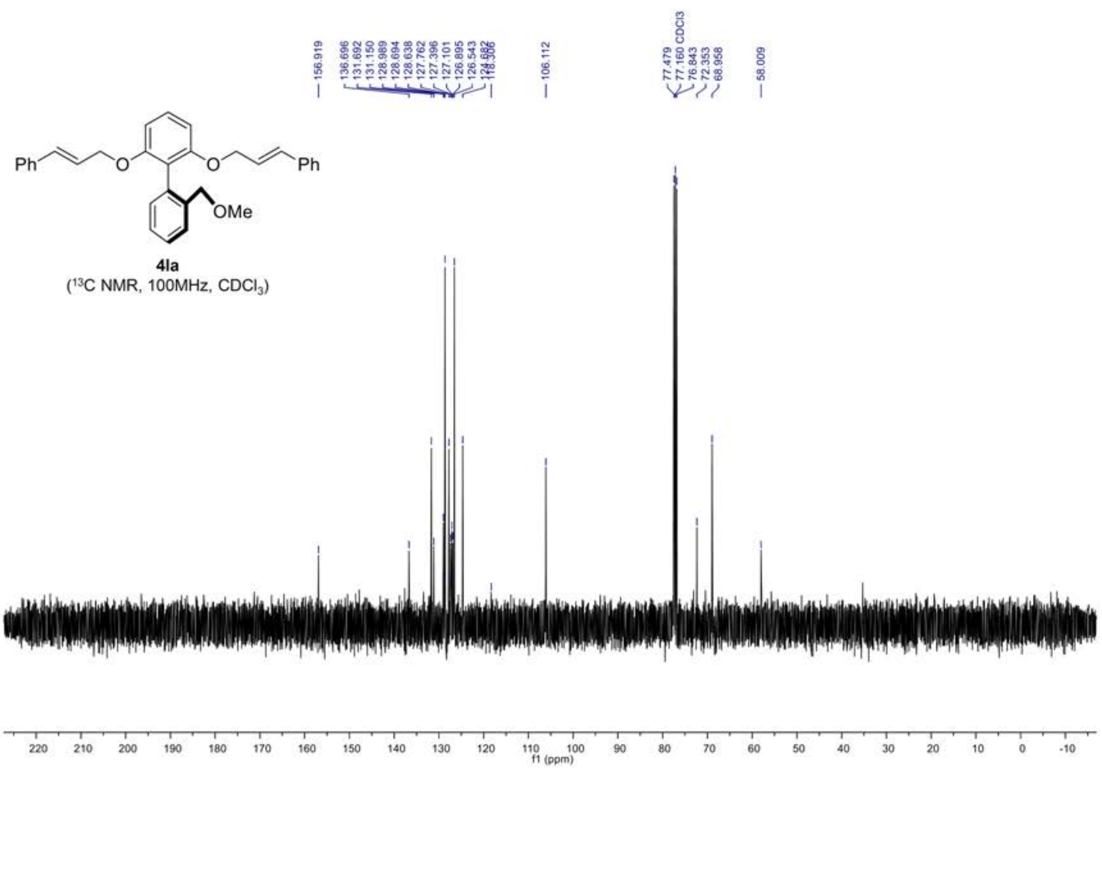

4la
(^{13}C NMR, 100MHz, $CDCl_3$)

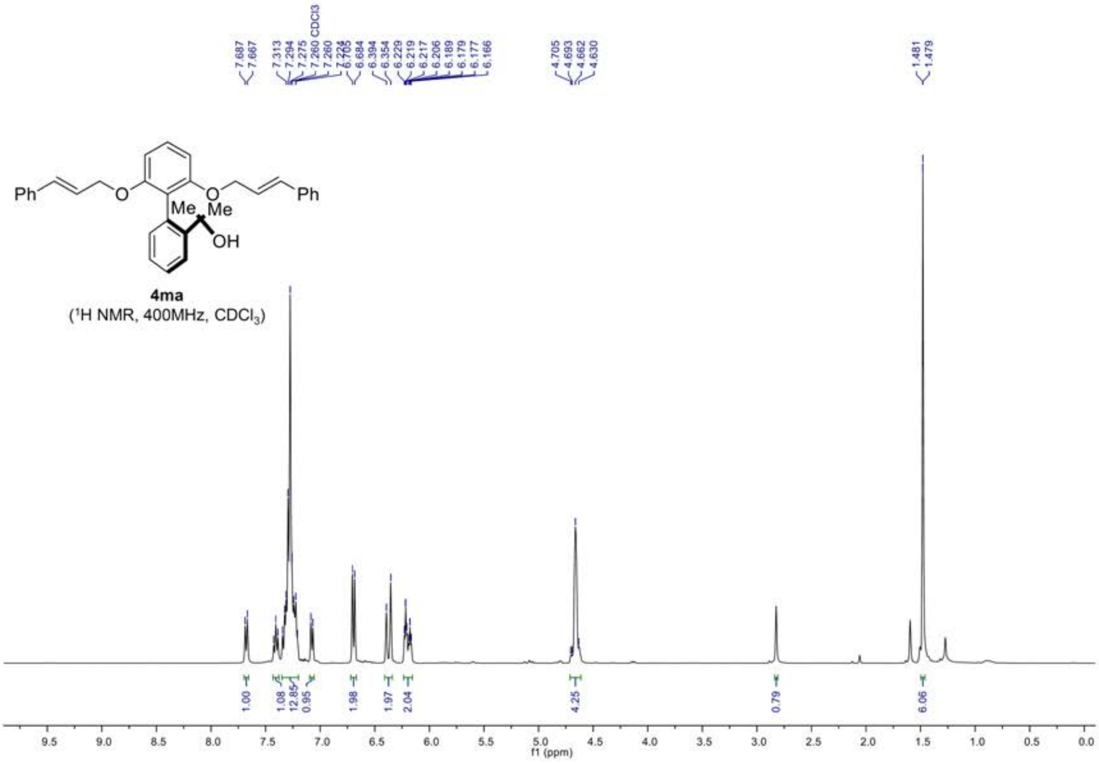
7.687
7.667
7.313
7.294
7.275
7.260 CDCl3
7.260
6.705
6.684
6.394
6.354
6.229
6.219
6.217
6.206
6.189
6.179
6.177
6.166
4.705
4.693
4.662
4.630
1.481
1.479
Ph
O
O
Ph
Me
Me
OH
4ma
(¹H NMR, 400MHz, CDCl3)
1.00
1.08
12.85
0.95
1.98
1.97
2.04
4.25
0.79
6.06
f1 (ppm)

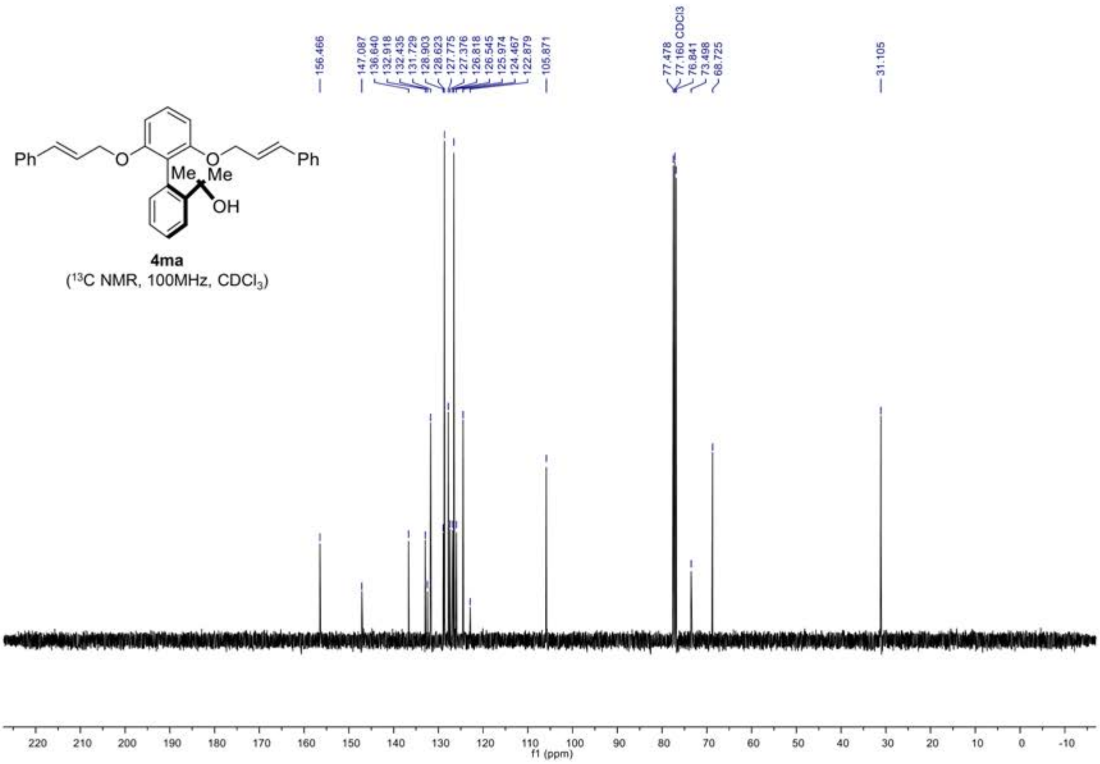
156.466
147.087
136.640
132.918
132.435
131.729
128.903
128.623
127.775
127.376
126.818
126.545
125.974
124.467
122.879
105.871
77.478
77.160 CDCl3
76.841
73.498
68.725
31.105
Ph
O
O
Ph
Me
Me
OH
4ma
(¹³C NMR, 100MHz, CDCl3)
f1 (ppm)

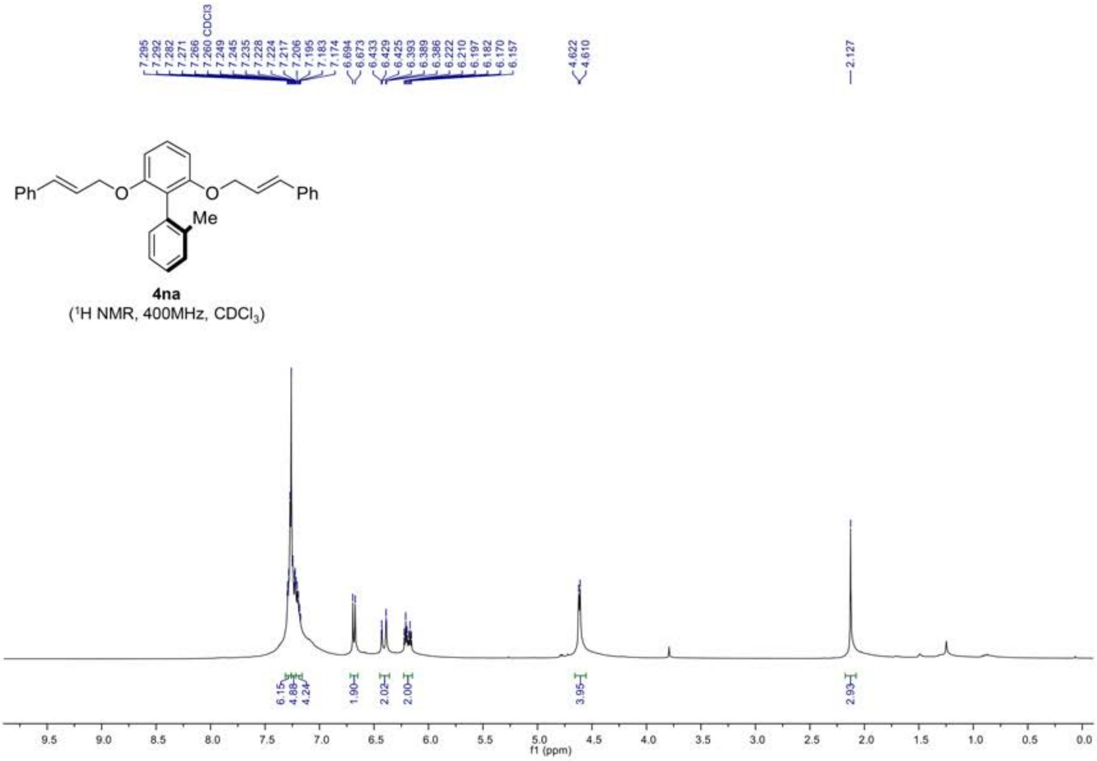

4na
(^{1}H NMR, 400MHz, $CDCl_3$)

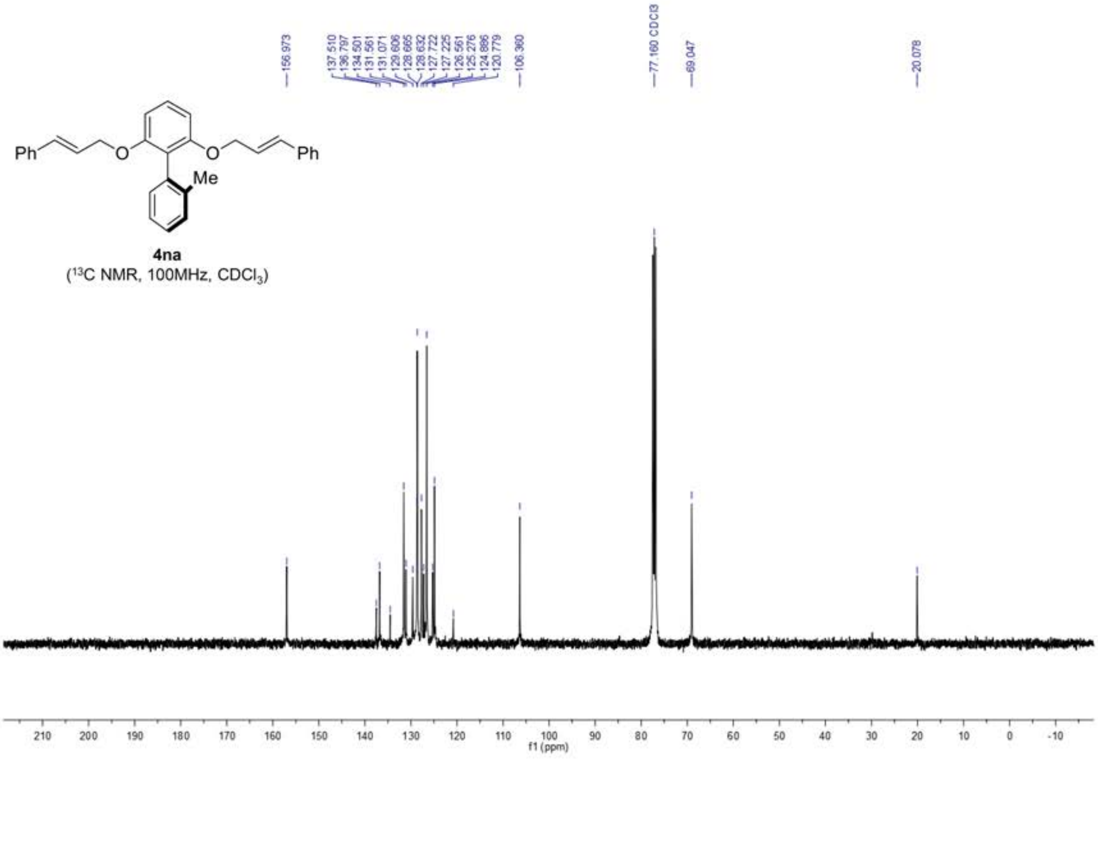

4na
(^{13}C NMR, 100MHz, $CDCl_3$)

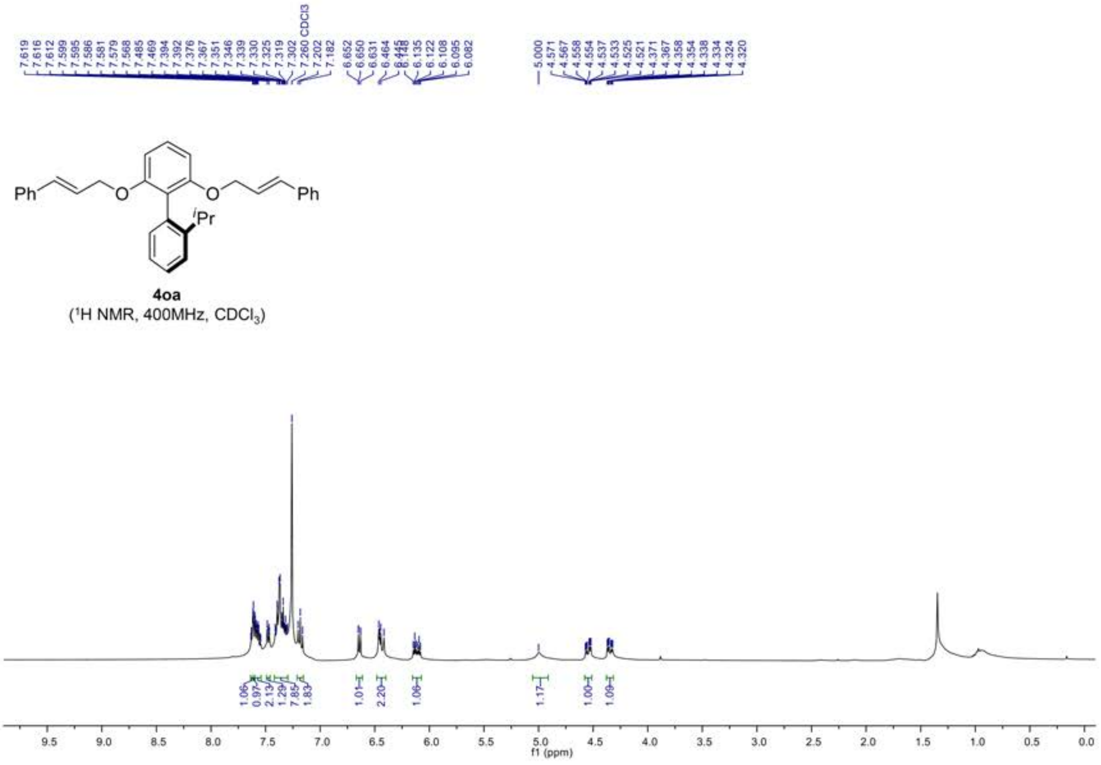

4oa
(^{1}H NMR, 400MHz, $CDCl_3$)

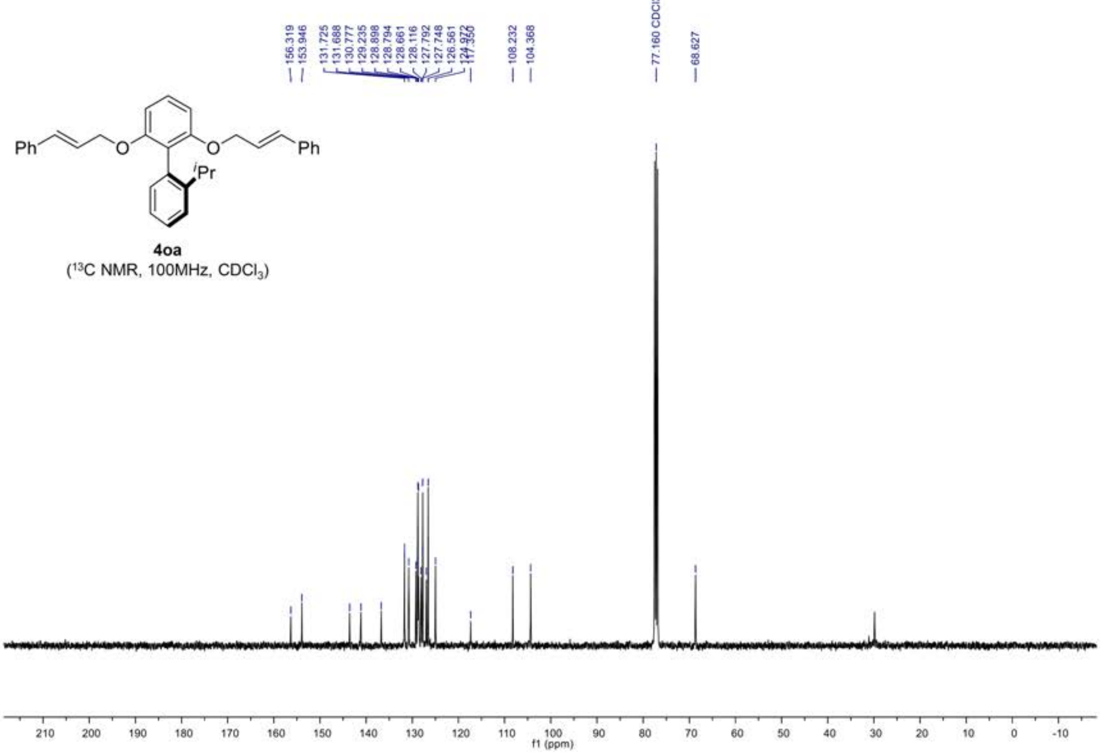

4oa
(^{13}C NMR, 100MHz, $CDCl_3$)

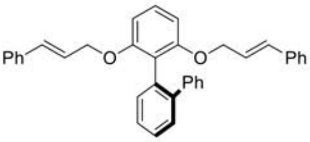

4pa
(^{1}H NMR, 400MHz, $CDCl_3$)

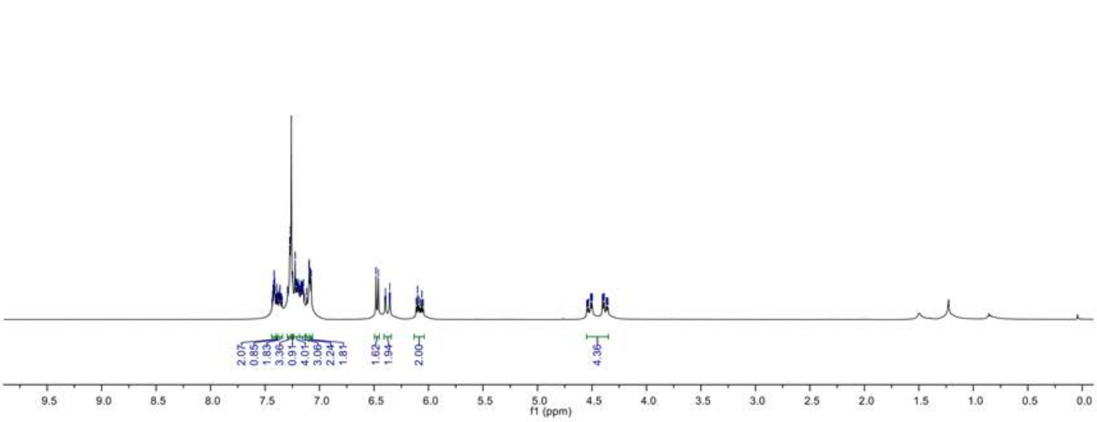

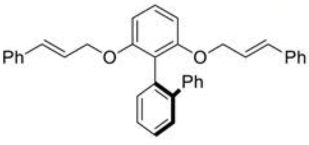

4pa
(^{13}C NMR, 100MHz, $CDCl_3$)

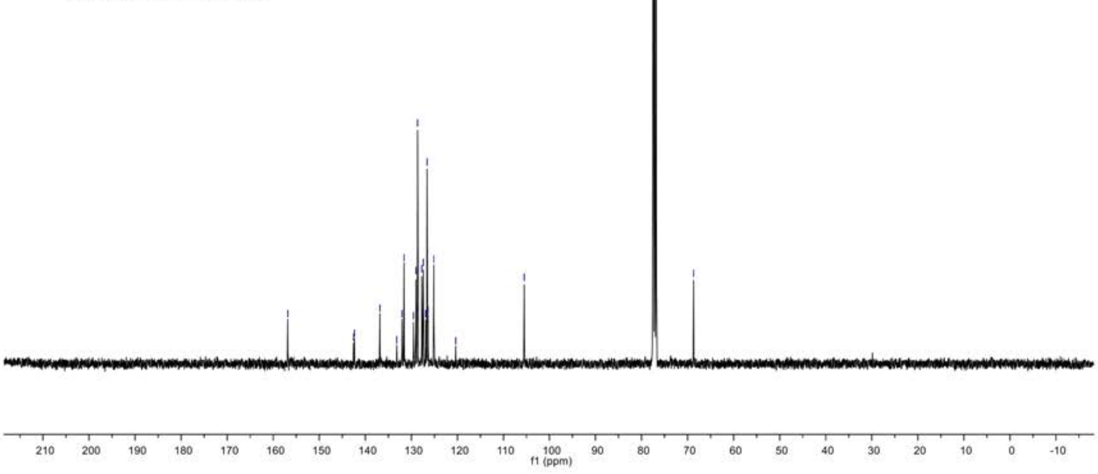

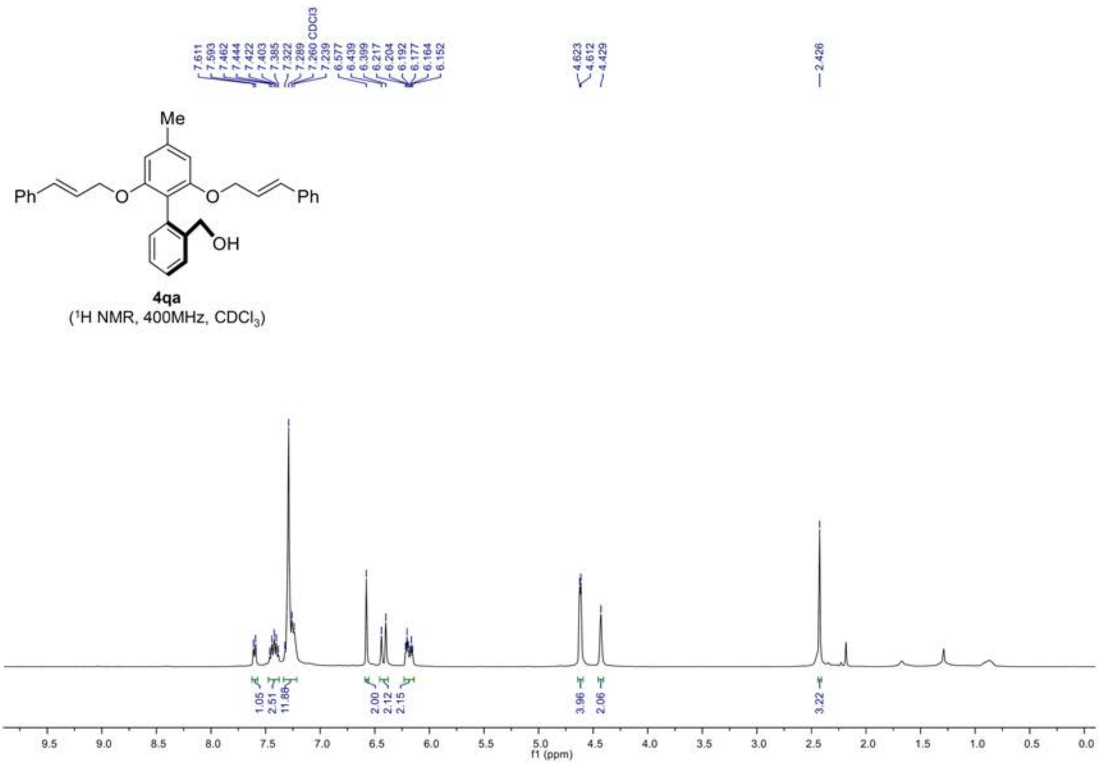

4qa
(^{1}H NMR, 400MHz, $CDCl_3$)

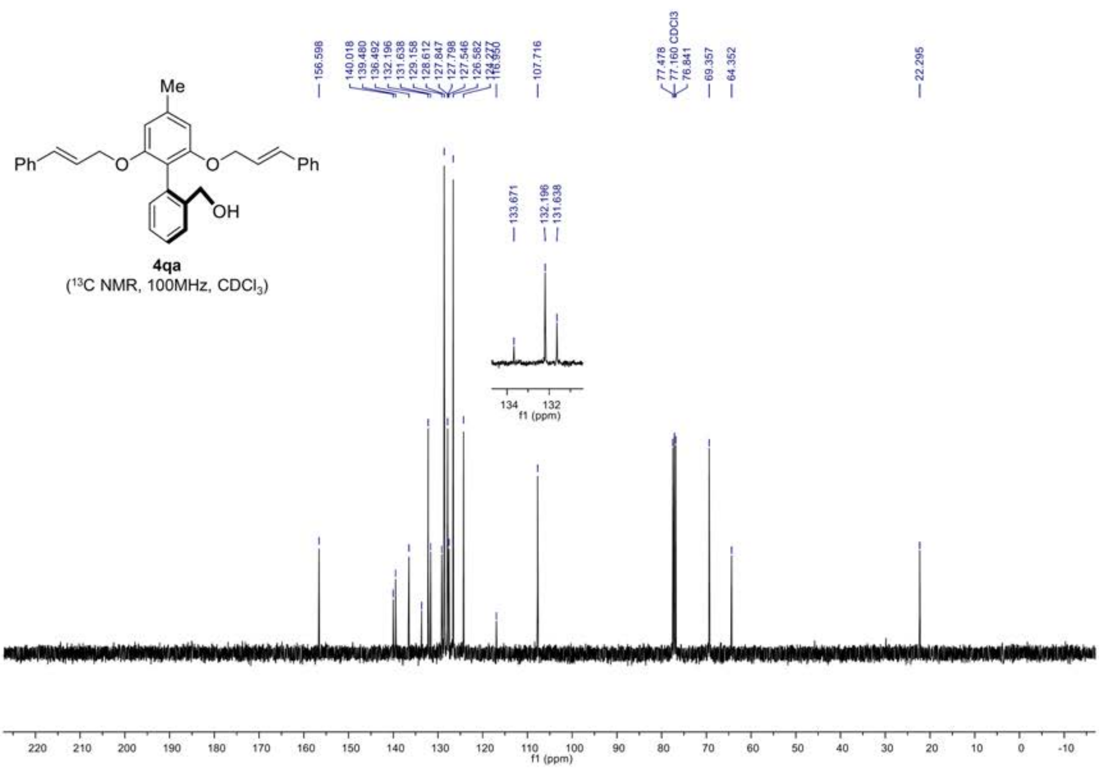

4qa
(^{13}C NMR, 100MHz, $CDCl_3$)

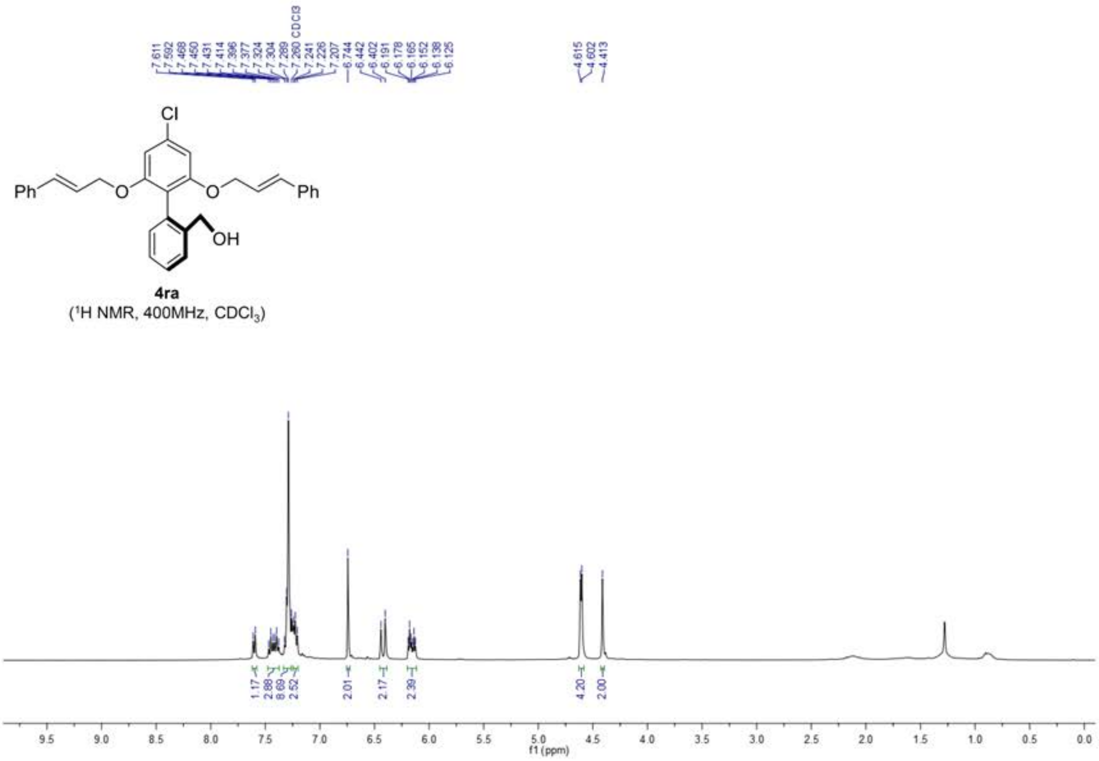

4ra
(^{1}H NMR, 400MHz, $CDCl_3$)

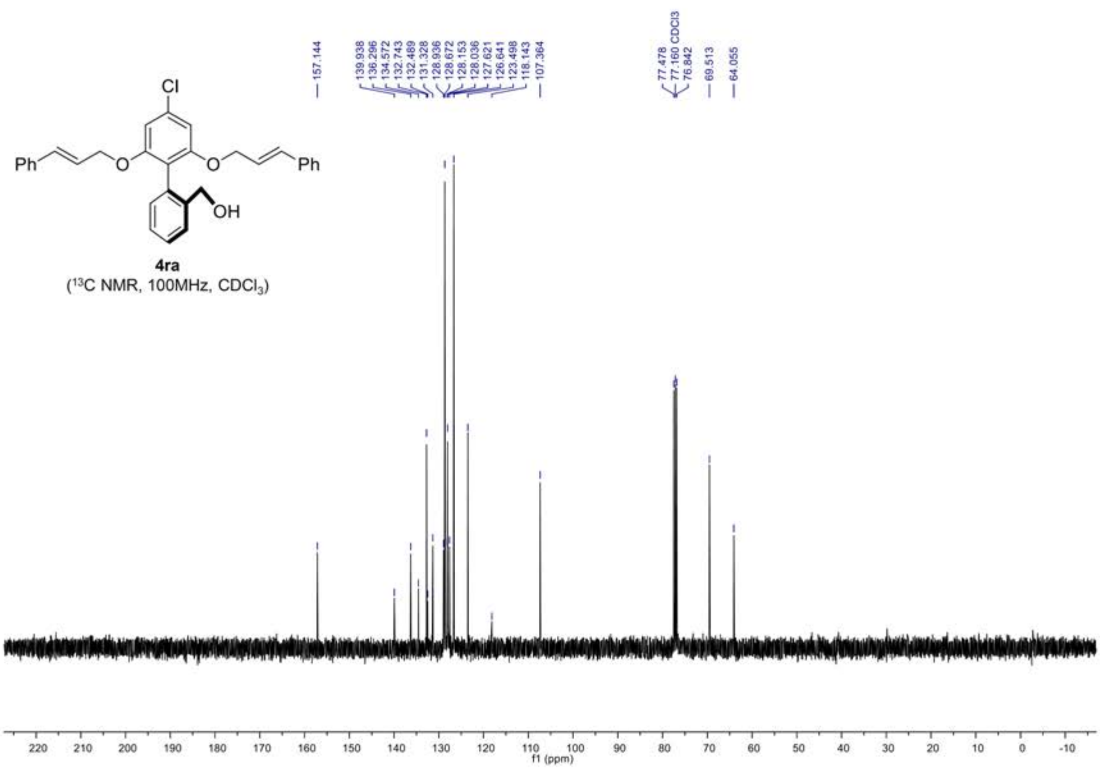

4ra
(^{13}C NMR, 100MHz, $CDCl_3$)

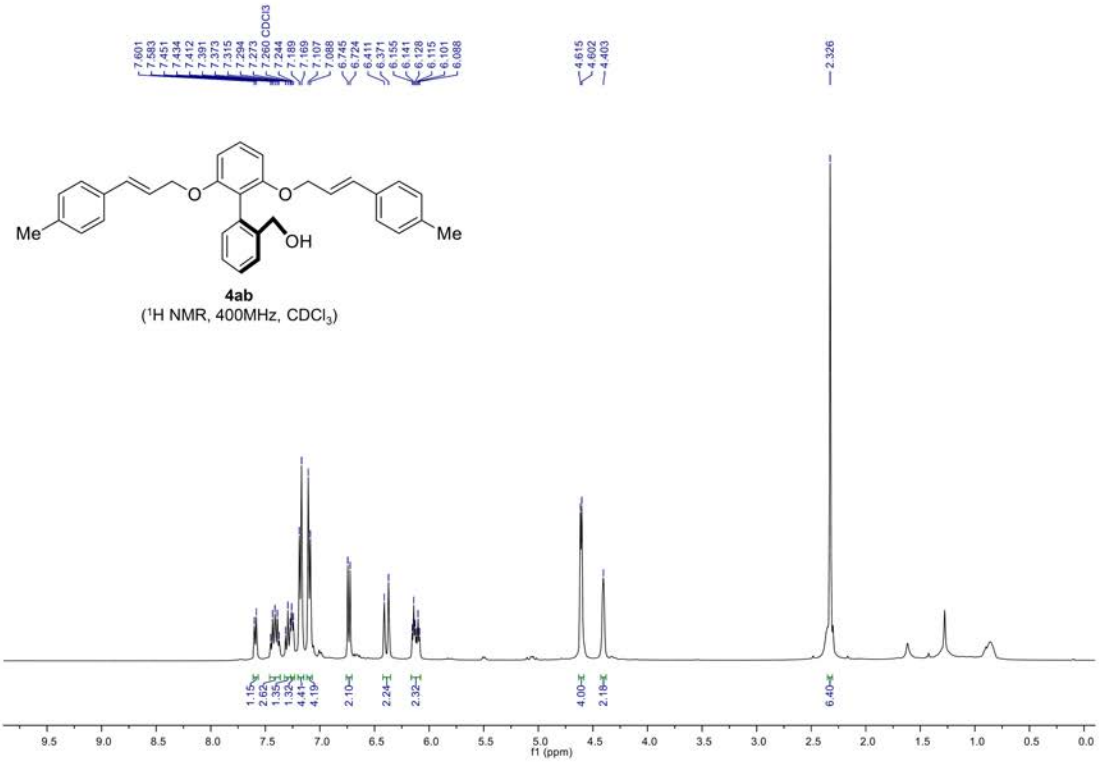
7.601 7.583 7.451 7.434 7.412 7.391 7.373 7.315 7.294 7.273 7.260 CDCl3 7.244 7.189 7.169 7.107 7.088 6.745 6.724 6.411 6.371 6.155 6.141 6.128 6.115 6.101 6.088
4.615 4.602 4.403
2.326
Me
O
O
Me
OH
4ab
(1H NMR, 400MHz, CDCl3)
1.15 2.62 1.35 1.32 4.41 4.19 2.10 2.24 2.32 4.00 2.18 6.40
9.5 9.0 8.5 8.0 7.5 7.0 6.5 6.0 5.5 5.0 4.5 4.0 3.5 3.0 2.5 2.0 1.5 1.0 0.5 0.0
f1 (ppm)

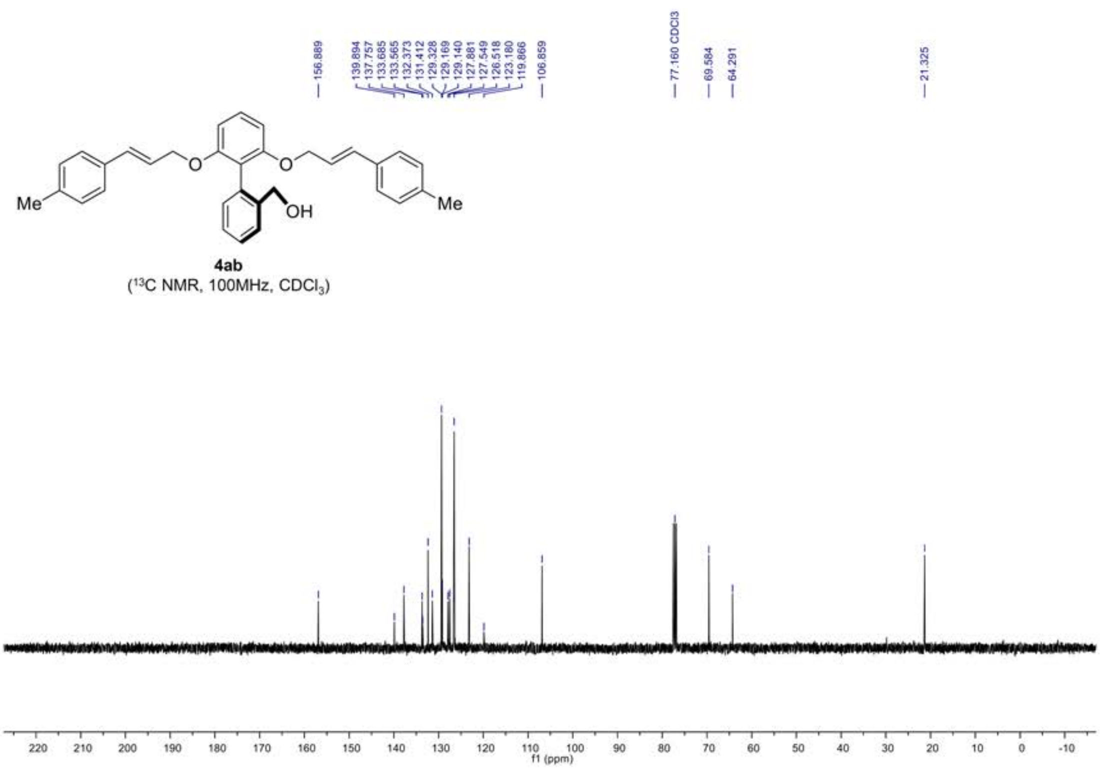
156.889
139.894 137.757 133.685 133.565 132.373 131.412 129.328 129.169 129.140 127.881 127.549 126.518 123.180 119.866
106.859
77.160 CDCl3
69.584
64.291
21.325
Me
O
O
Me
OH
4ab
(13C NMR, 100MHz, CDCl3)
220 210 200 190 180 170 160 150 140 130 120 110 100 90 80 70 60 50 40 30 20 10 0 -10
f1 (ppm)

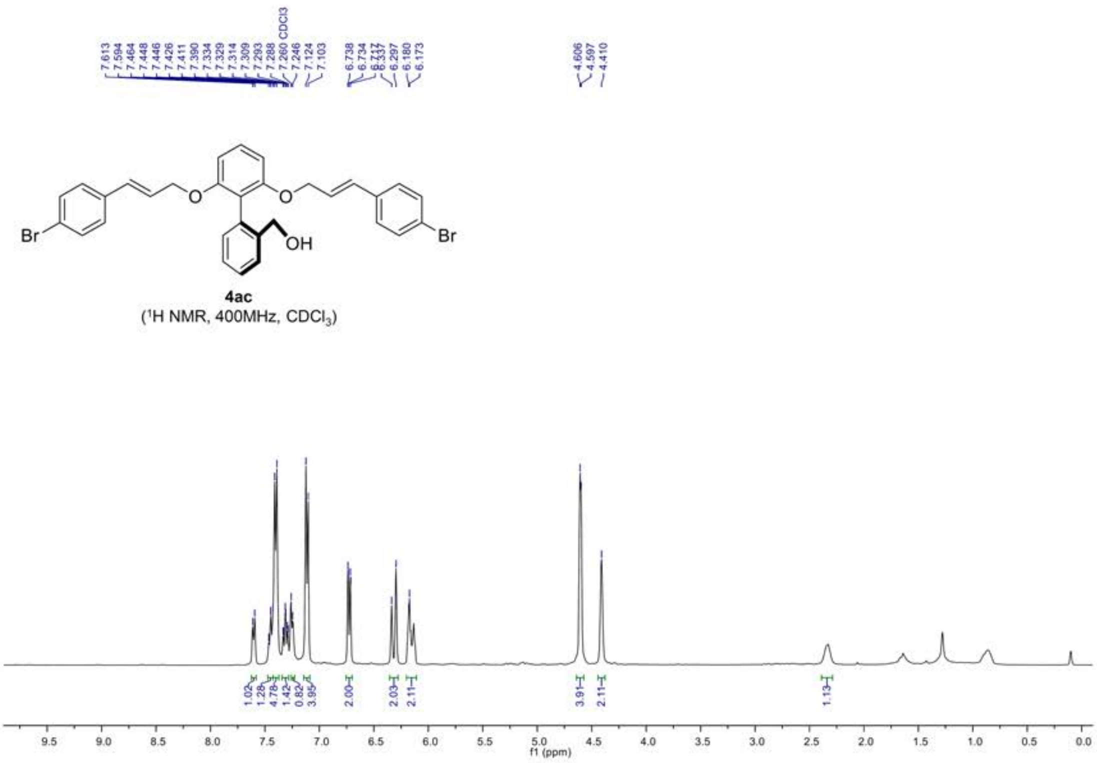

4ac
(^{1}H NMR, 400MHz, $CDCl_3$)

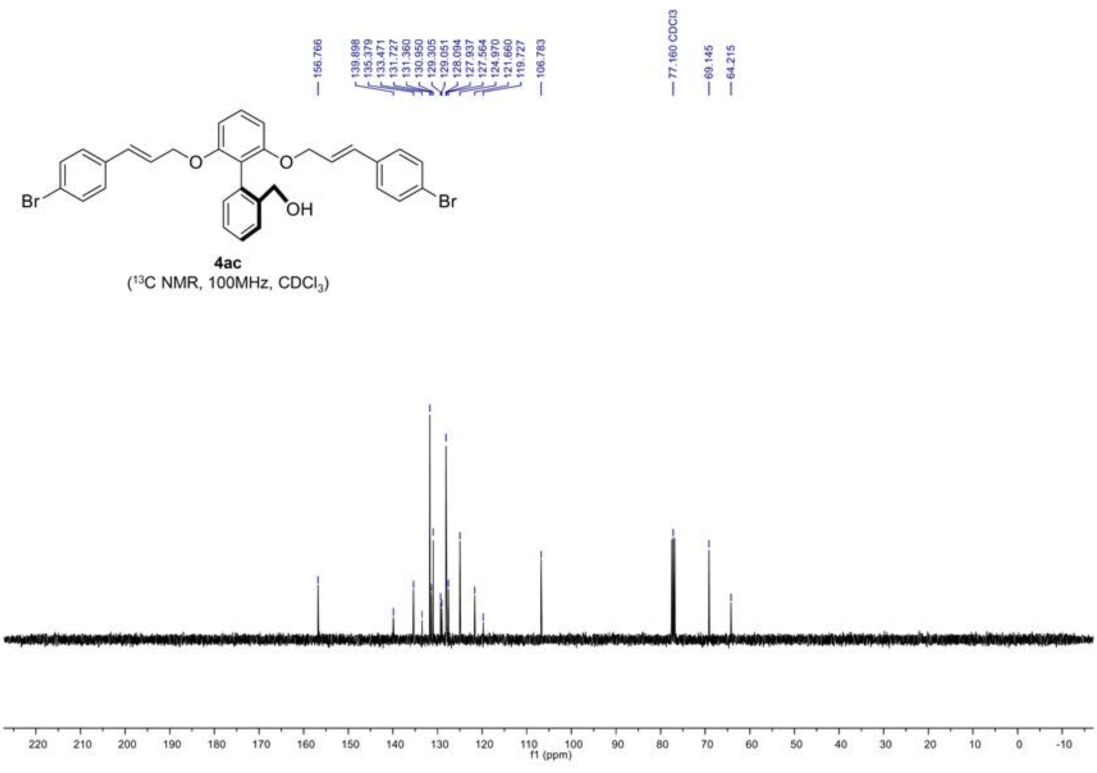

4ac
(^{13}C NMR, 100MHz, $CDCl_3$)

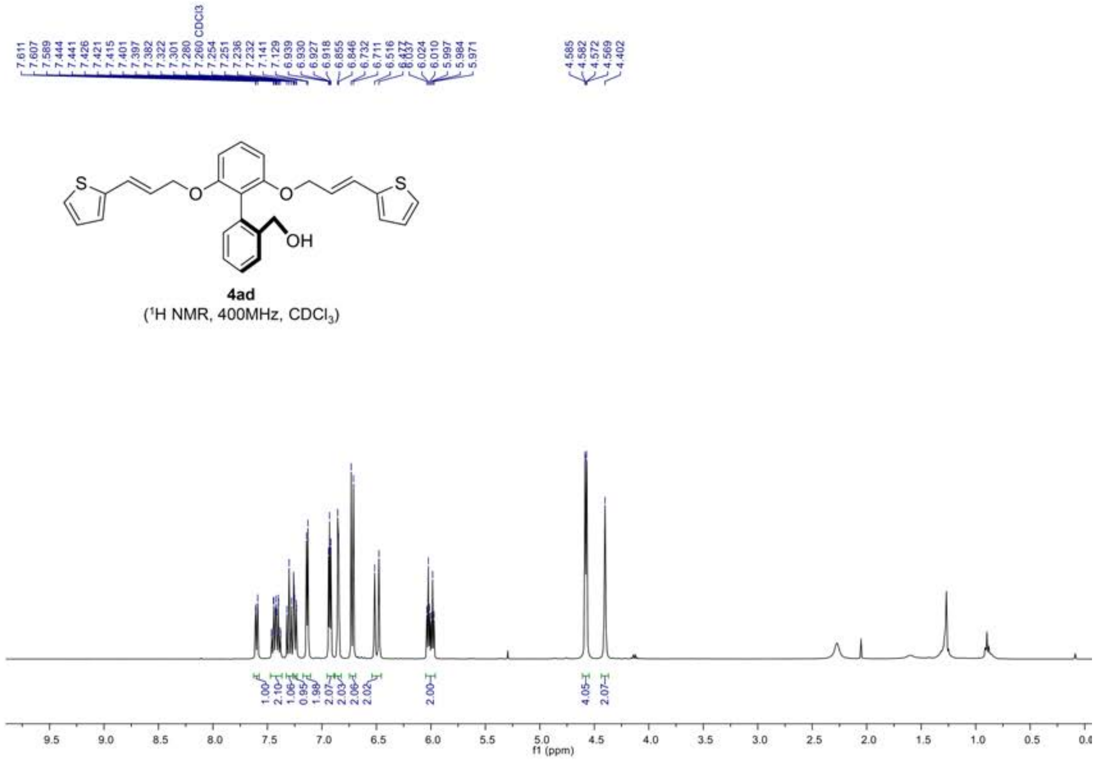

4ad
(^{1}H NMR, 400MHz, $CDCl_3$)

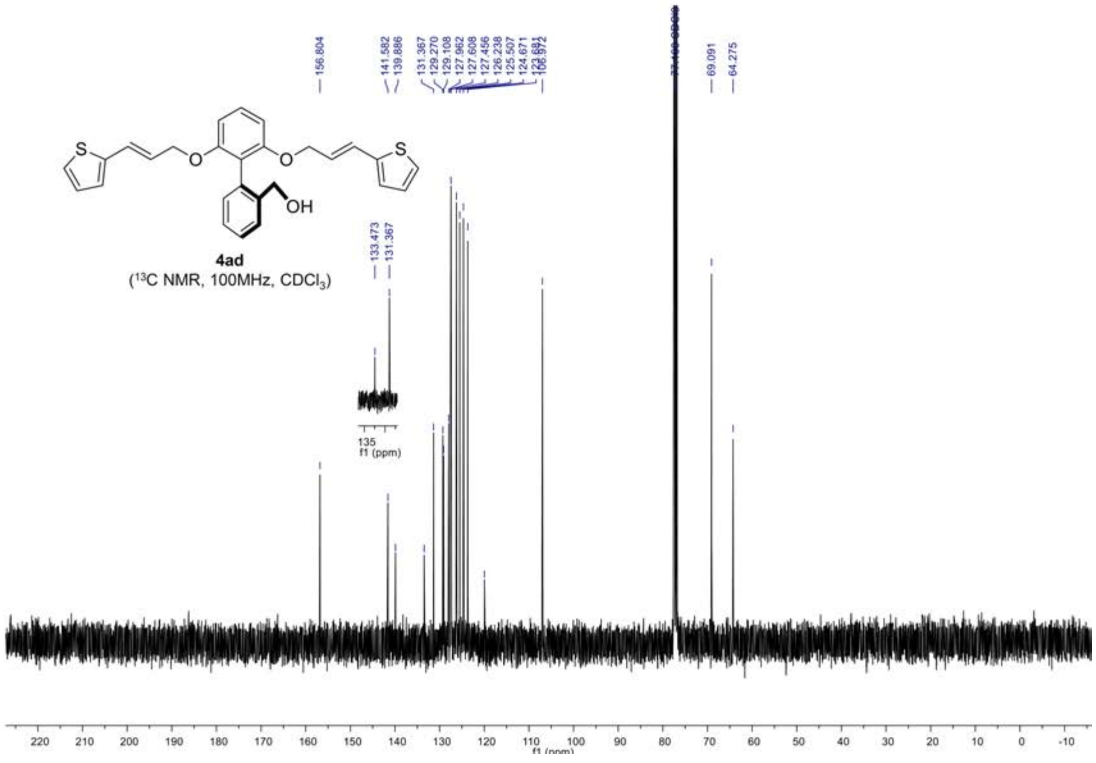

4ad
(^{13}C NMR, 100MHz, $CDCl_3$)

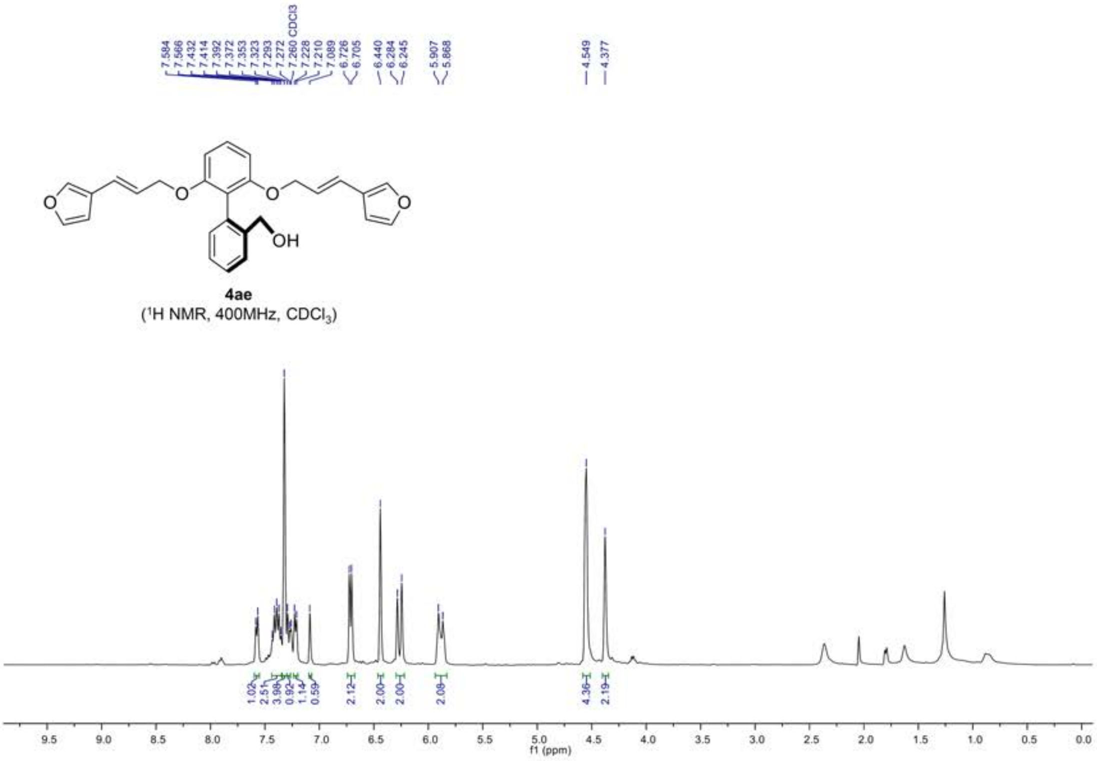

4ae
(^{1}H NMR, 400MHz, $CDCl_3$)

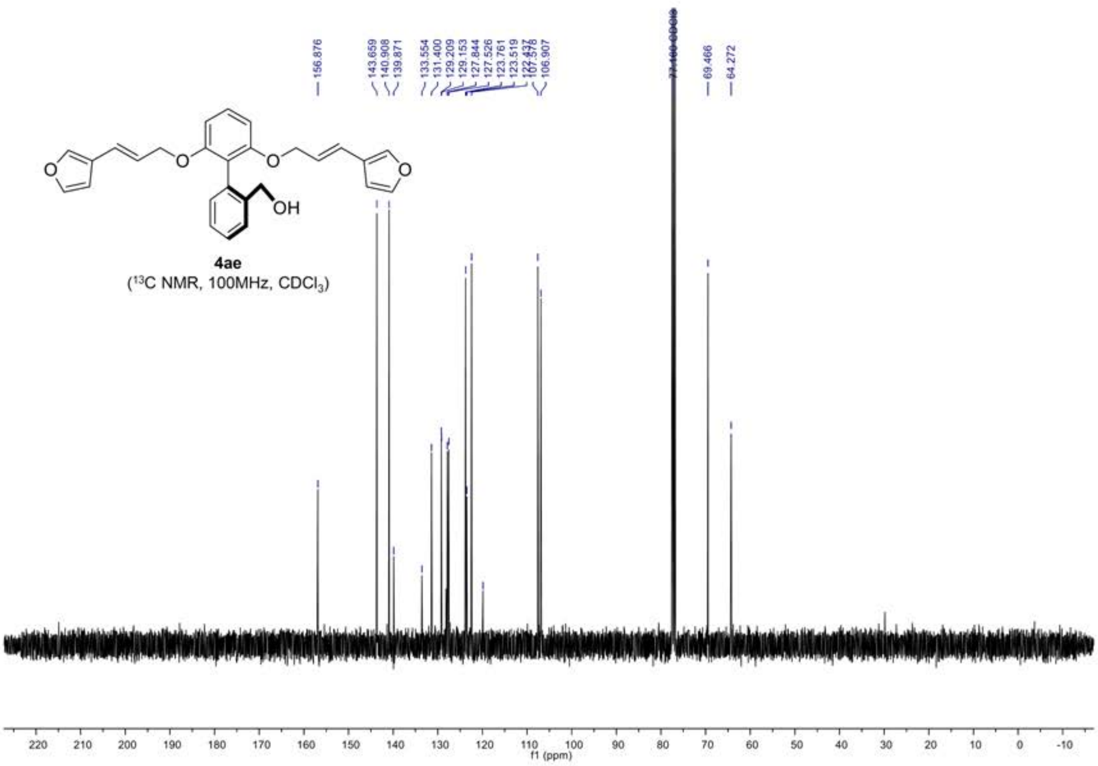

4ae
(^{13}C NMR, 100MHz, $CDCl_3$)

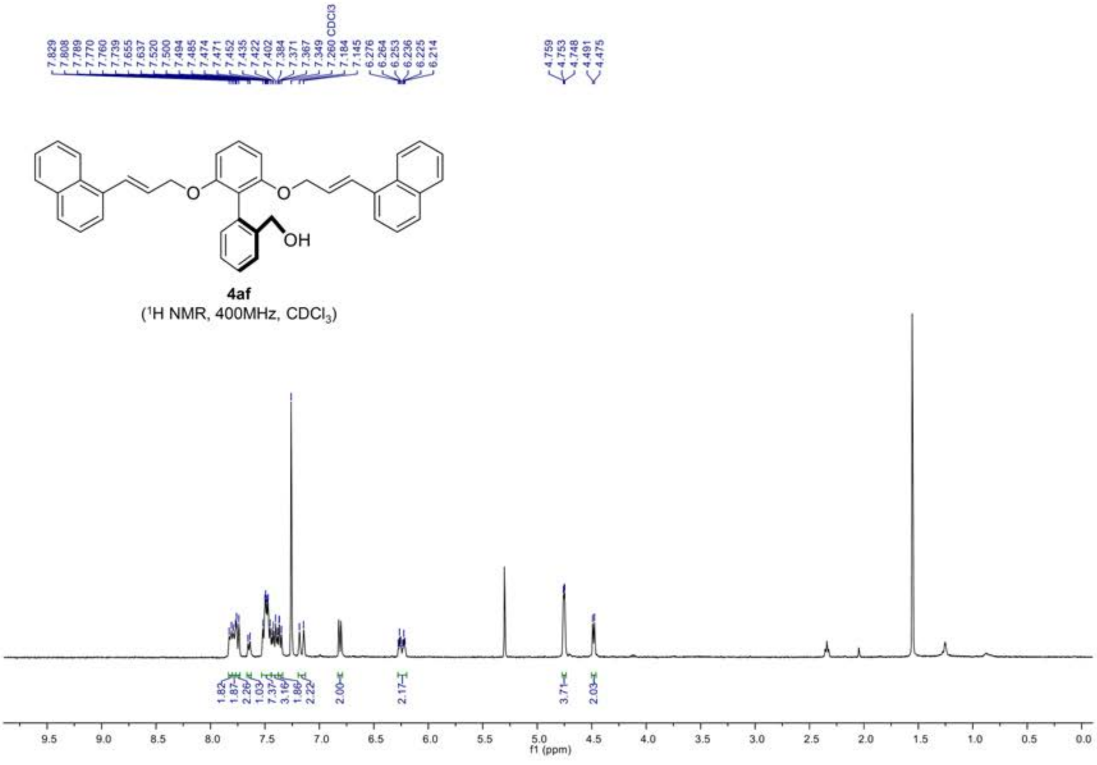

4af
(^{1}H NMR, 400MHz, $CDCl_3$)

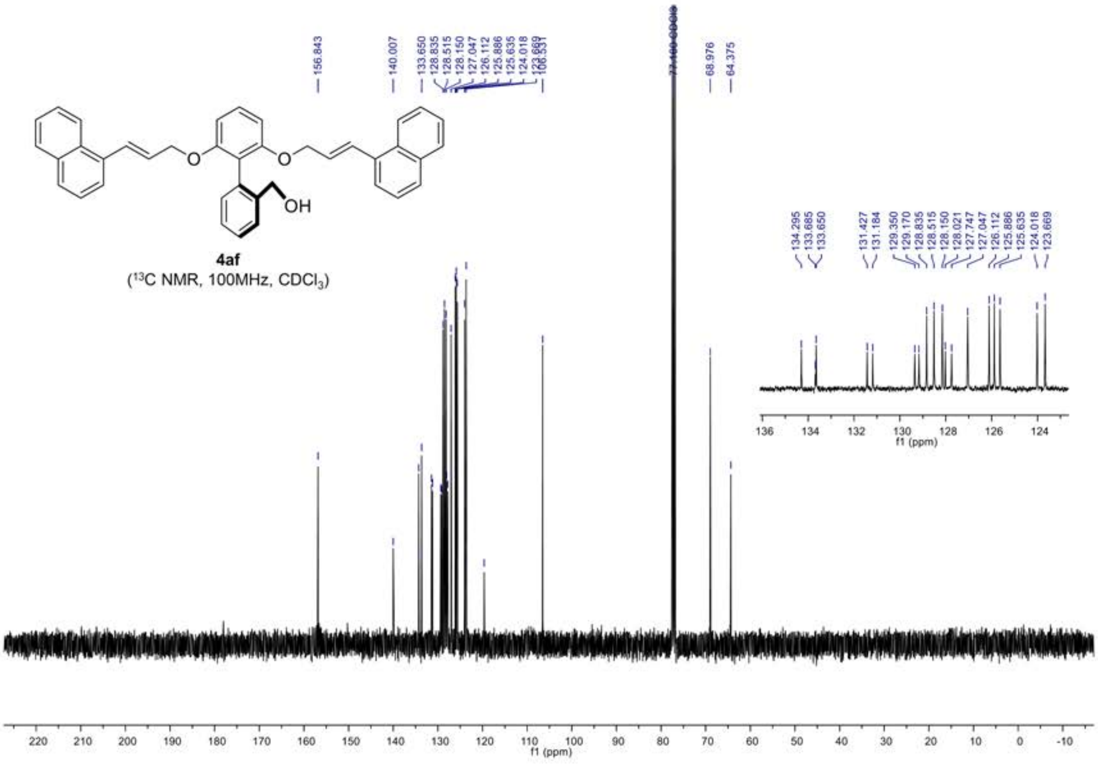

4af
(^{13}C NMR, 100MHz, $CDCl_3$)

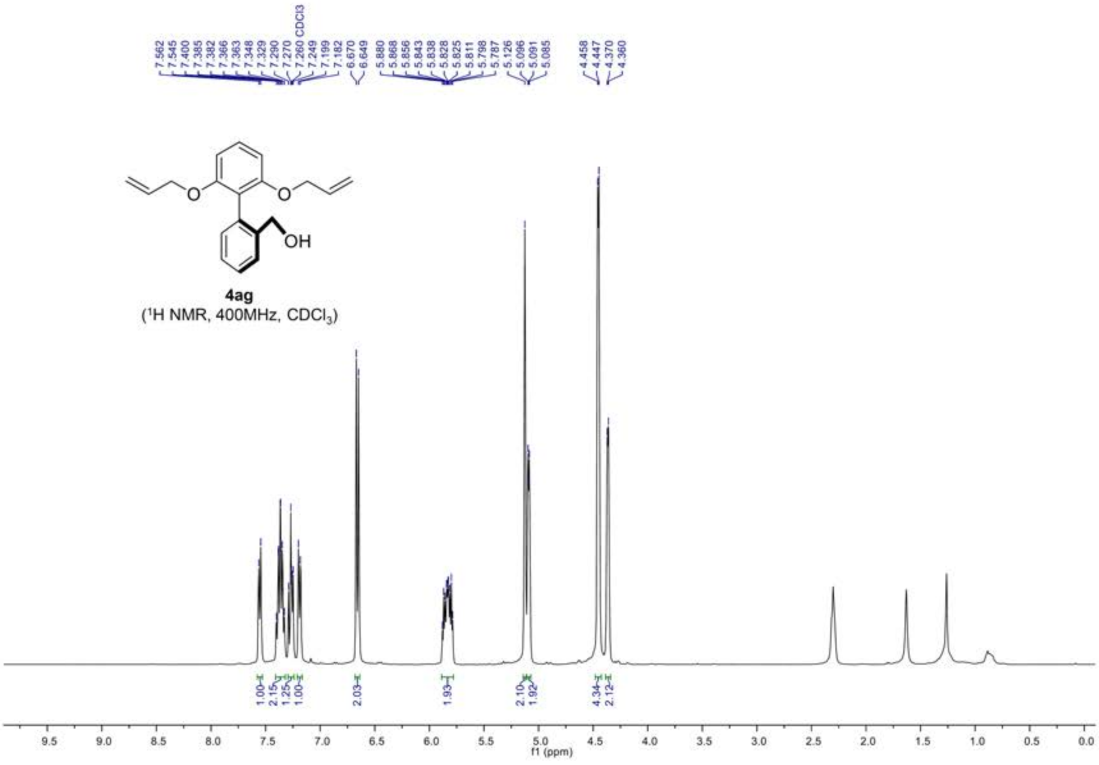
4ag
(^{1}H NMR, 400MHz, $CDCl_3$)
OH
O
f1 (ppm)

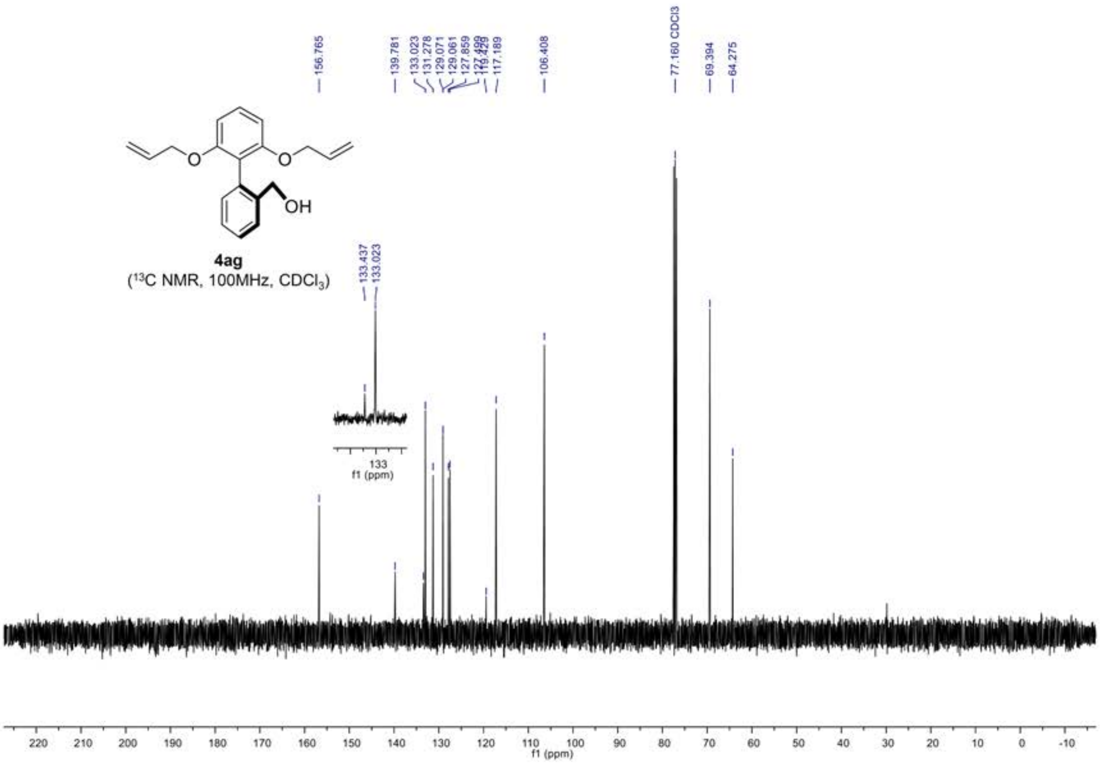
4ag
(^{13}C NMR, 100MHz, $CDCl_3$)
OH
O
156.765
139.781
133.023
131.278
129.071
129.061
127.859
117.189
106.408
77.160 CDCl3
69.394
64.275
133.437
133.023
f1 (ppm)

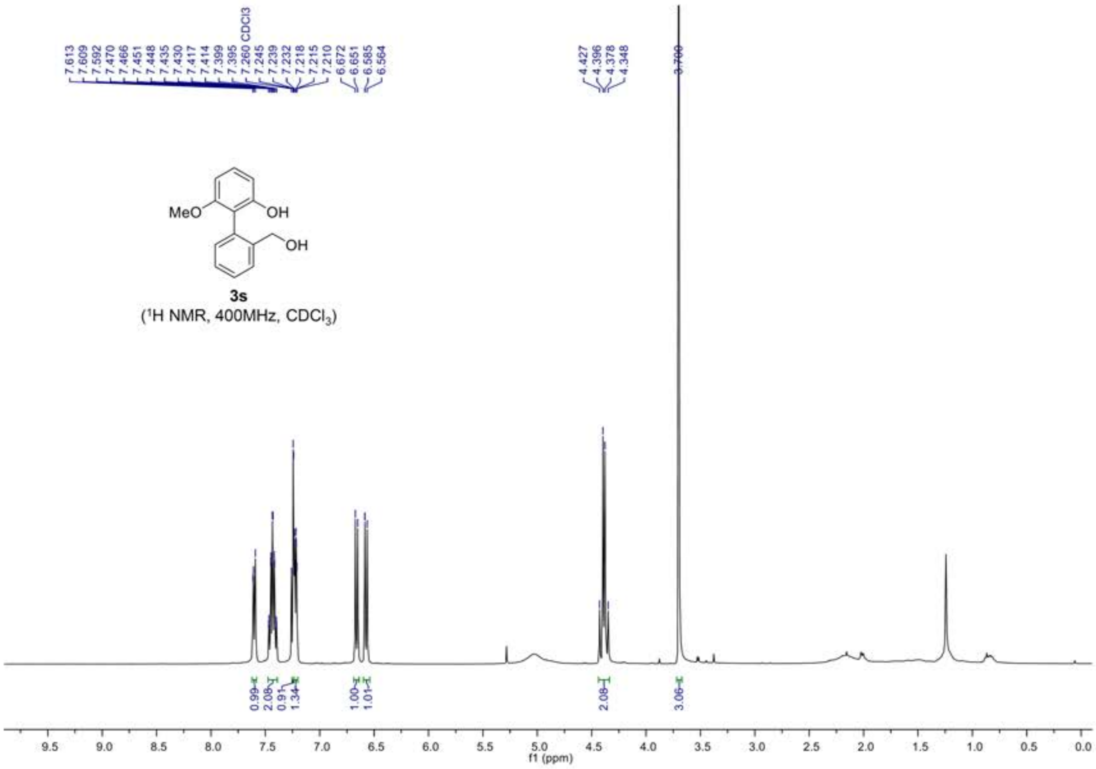

3s
(^{1}H NMR, 400MHz, $CDCl_3$)

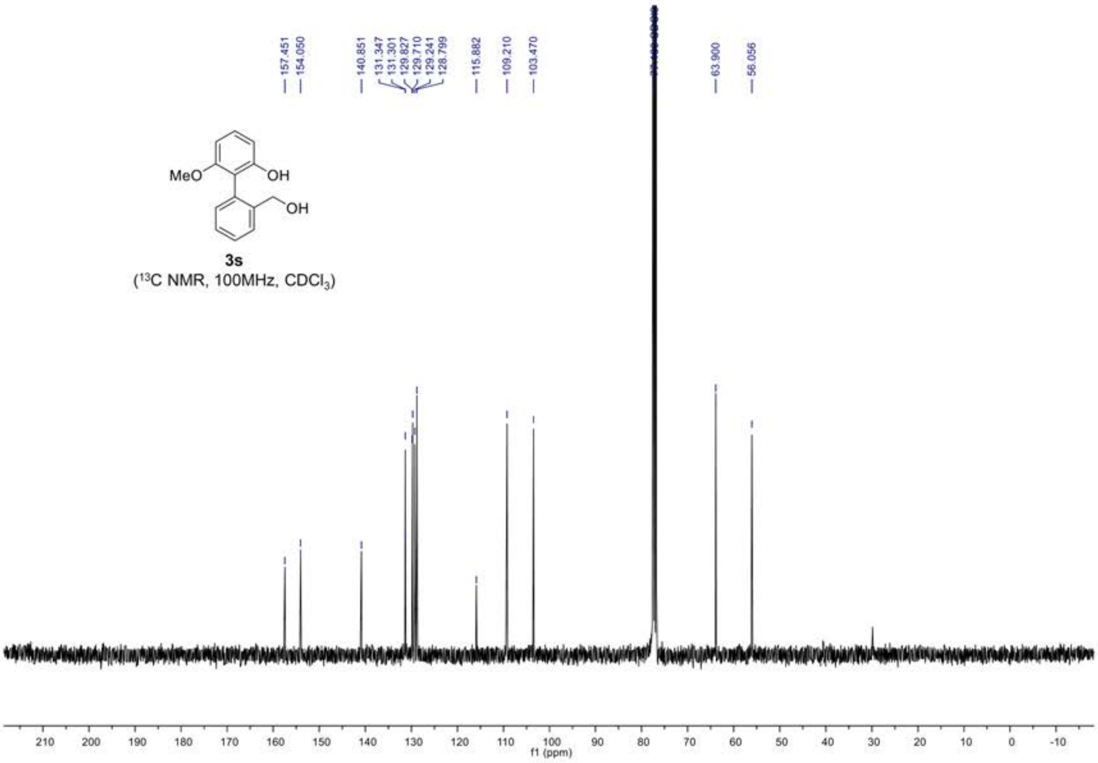

3s
(^{13}C NMR, 100MHz, $CDCl_3$)

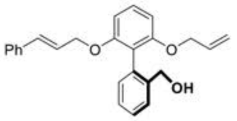

4ag′
(^{1}H NMR, 400MHz, $CDCl_3$)

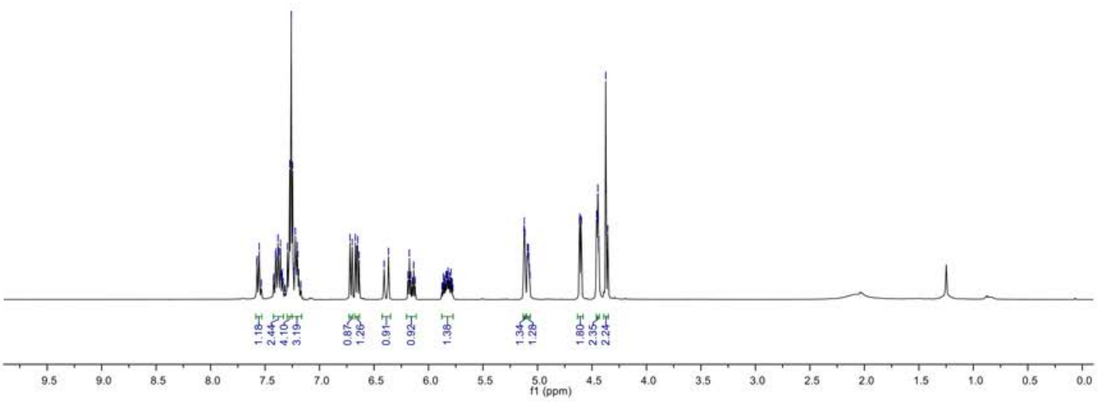

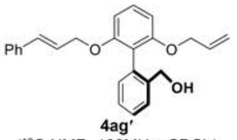

4ag′
(^{13}C NMR, 100MHz, $CDCl_3$)

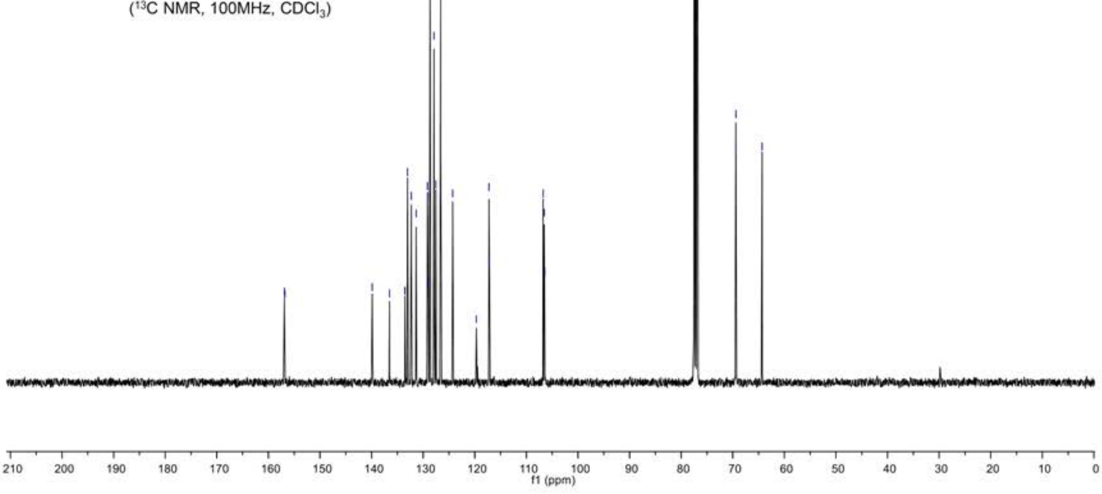

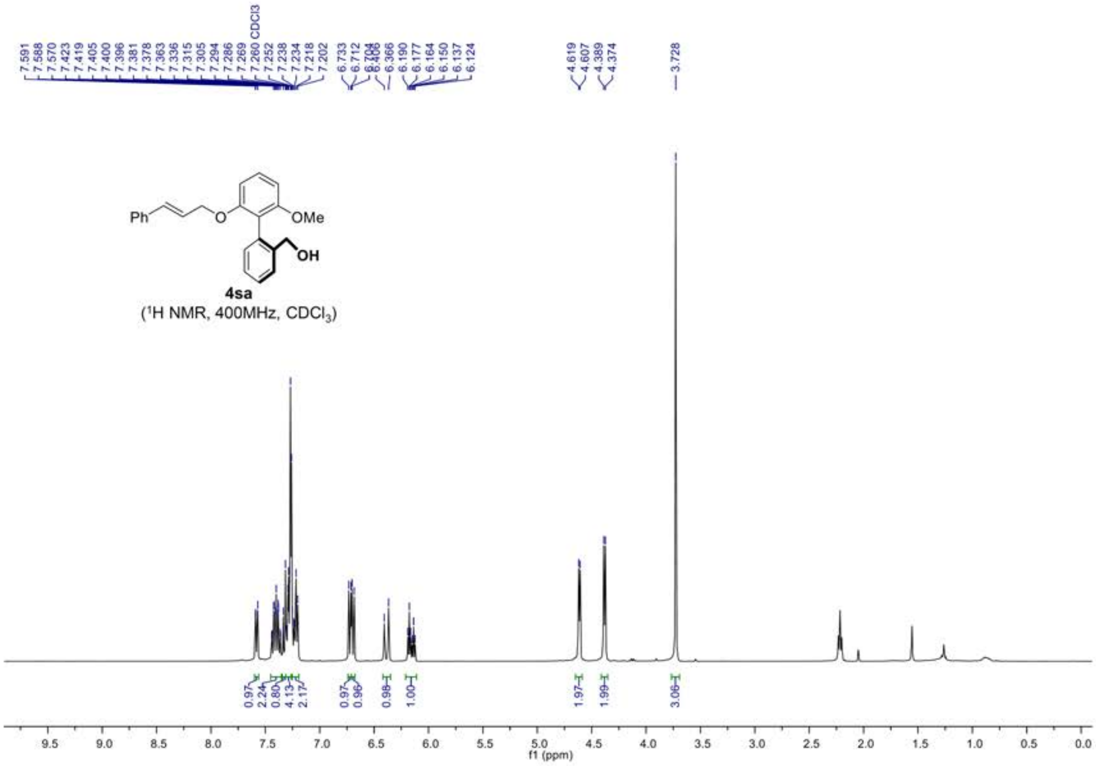

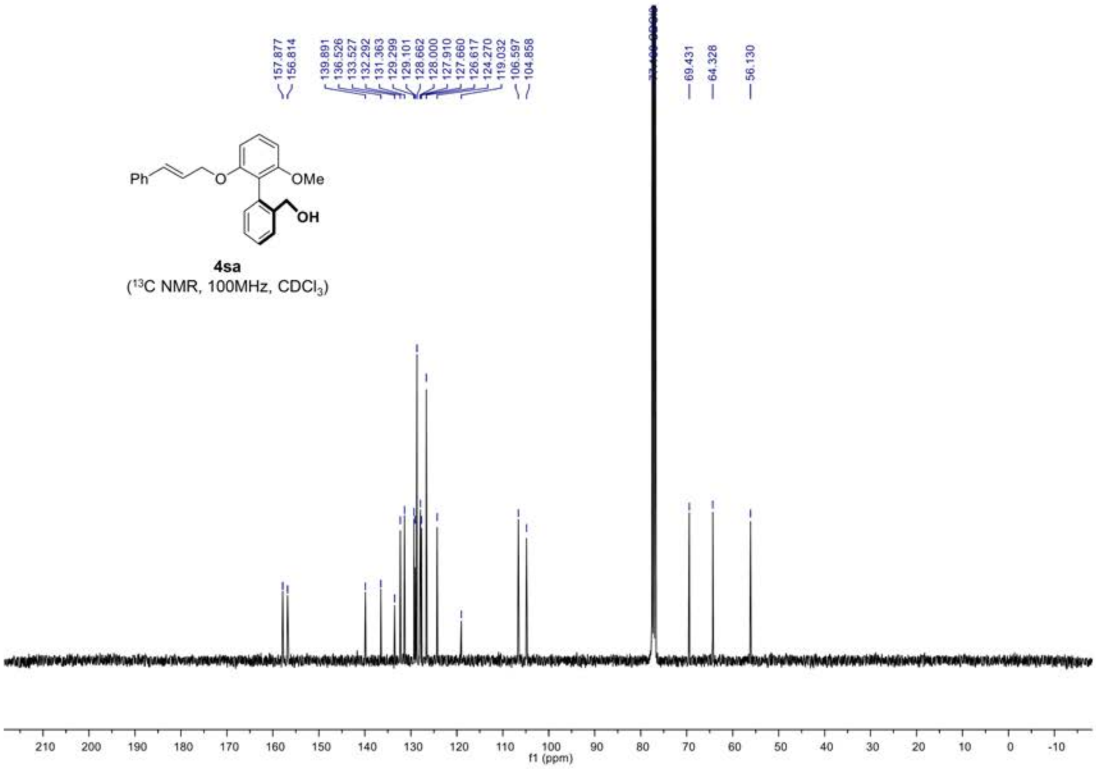

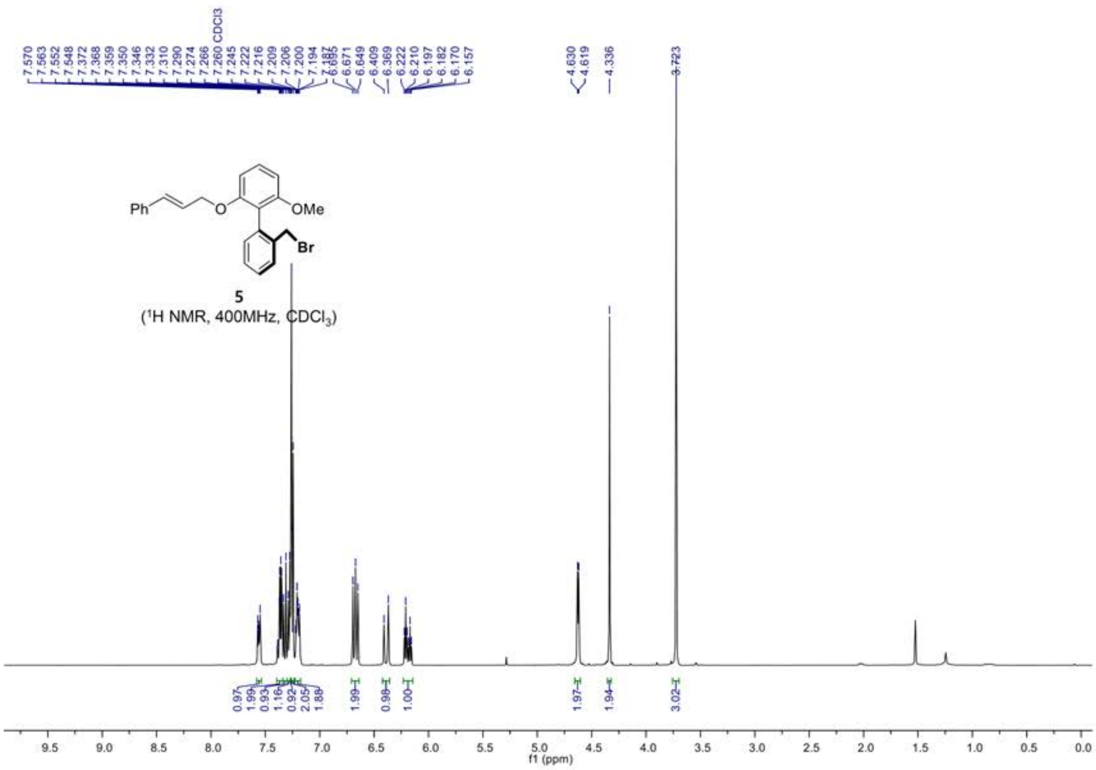
Ph
OMe
Br
5
(1H NMR, 400MHz, CDCl3)
f1 (ppm)

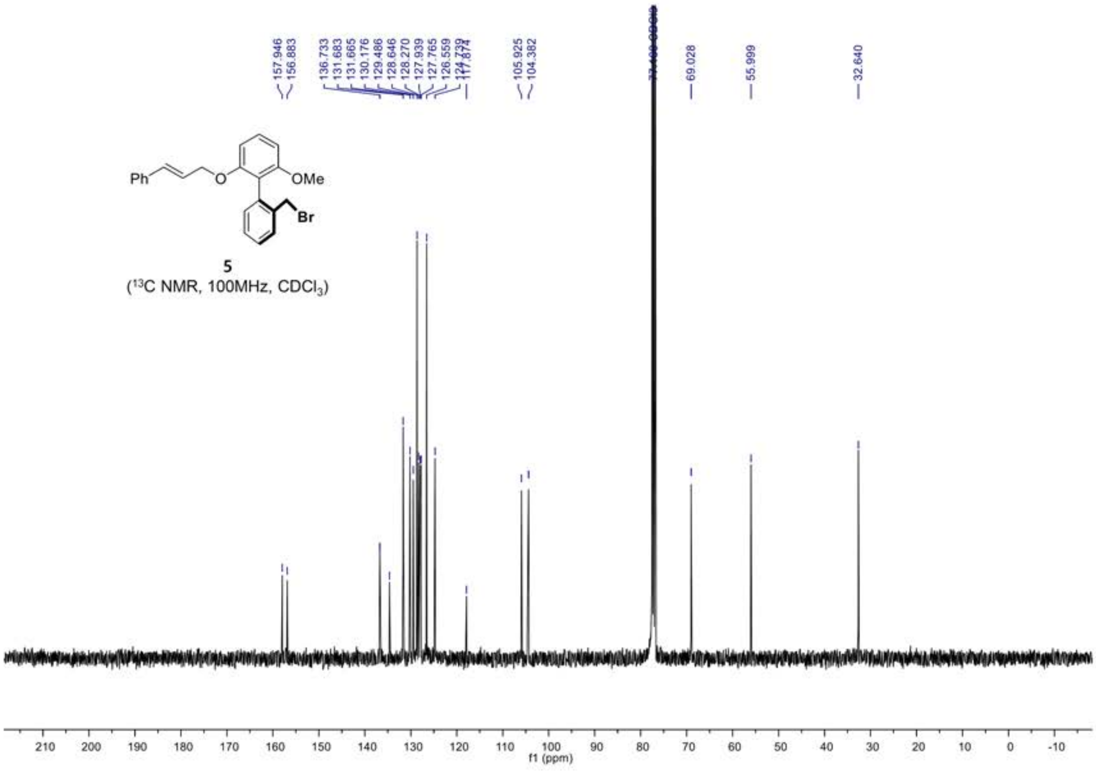
Ph
OMe
Br
5
(13C NMR, 100MHz, CDCl3)
f1 (ppm)

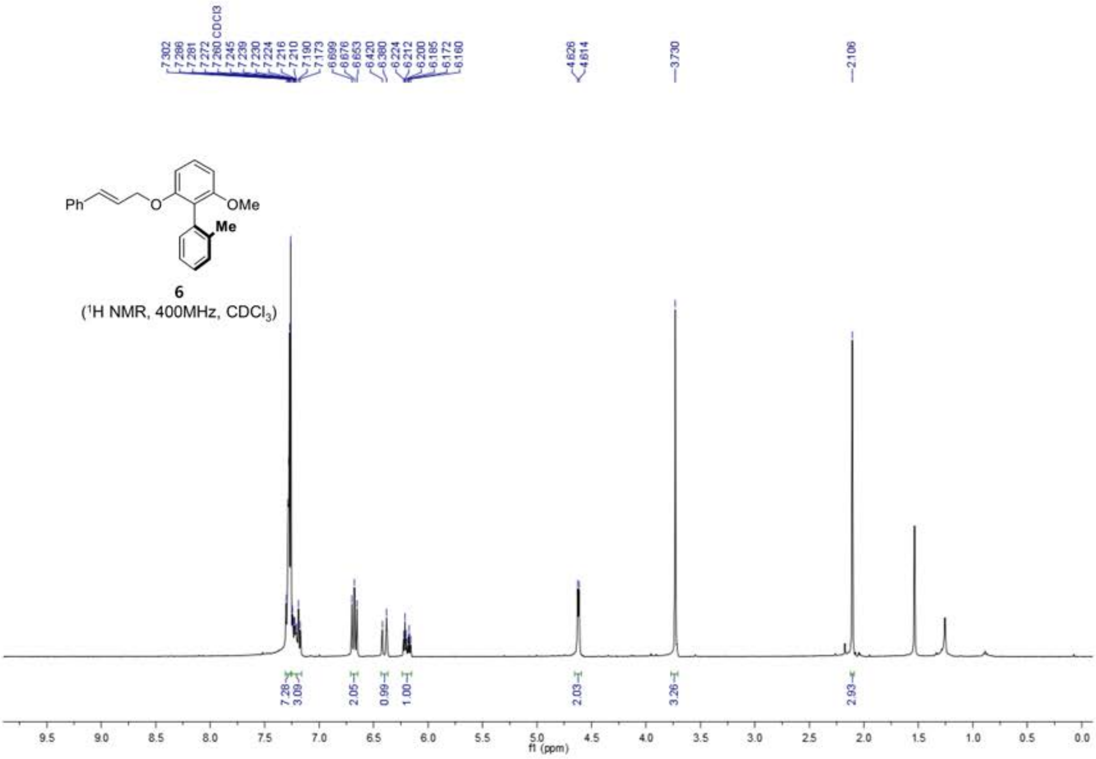
Ph
OMe
Me
6
(1H NMR, 400MHz, CDCl3)
f1 (ppm)

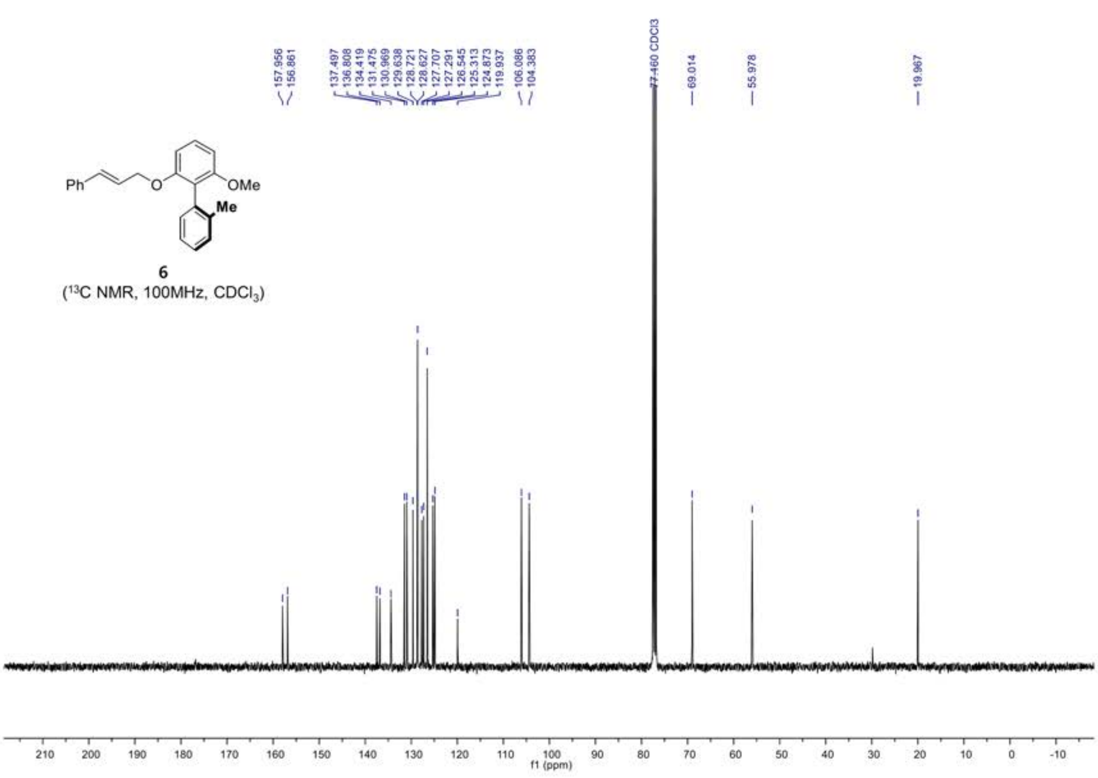
Ph
OMe
Me
6
(13C NMR, 100MHz, CDCl3)
f1 (ppm)

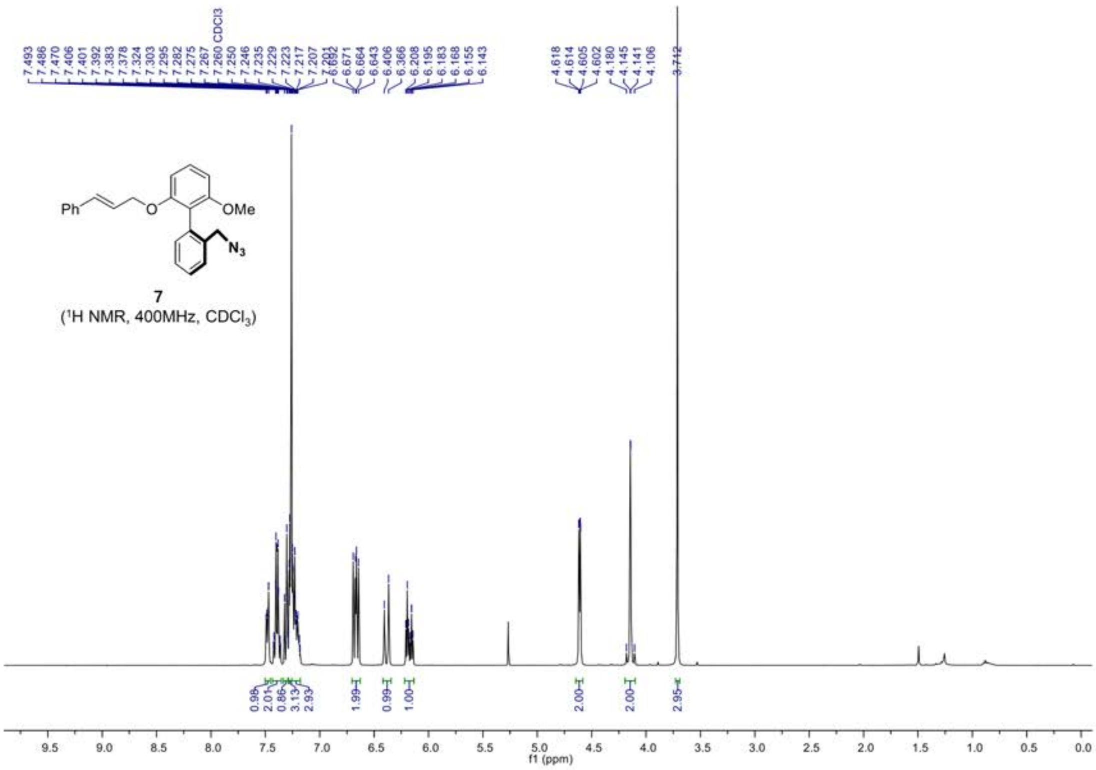

7
(^{1}H NMR, 400MHz, $CDCl_3$)

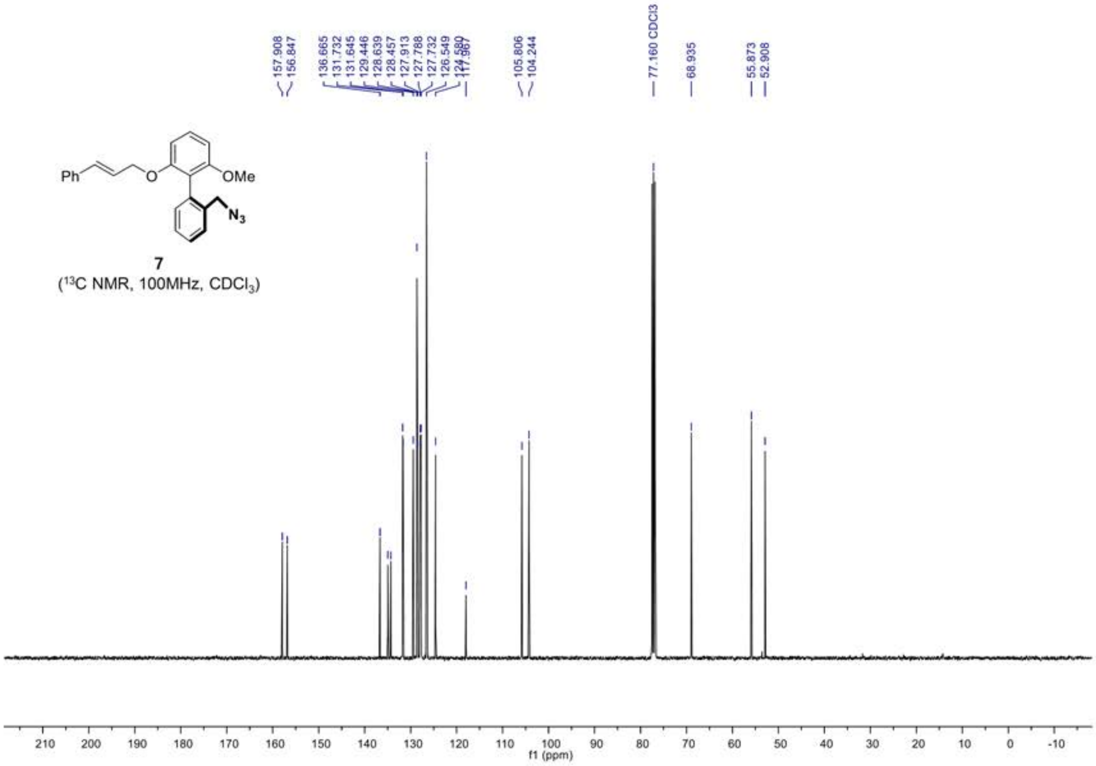

7
(^{13}C NMR, 100MHz, $CDCl_3$)
