# Supplementary material for: Atroposelective desymmetrization of 2-arylresorcinols via Tsuji-Trost allylation
Source: Commun Chem. 2023 Feb 25;6:42. doi: 10.1038/s42004-023-00839-z (PMC9968306; doi:10.1038/s42004-023-00839-z)
Supplement: Supplementary file 5 — Supplementary Data 3 [file 42004_2023_839_MOESM5_ESM.pdf]

Copies of HPLC Chromatograms

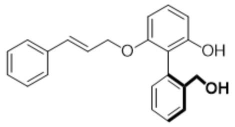

3aa

HPLC conditions:
Chiralpak AD-H, 20% ^{i}PrOH/Hex eluent 1.0 mL/min, 254 nm

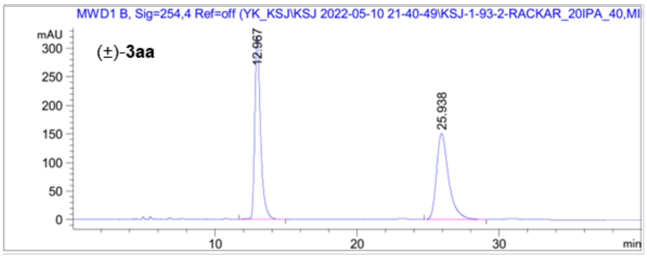

Peak #	RetTime [min]	Type	Width [min]	Area [mAU*s]	Height [mAU]	Area %
1	12.967	VB R	0.4214	9021.14551	319.49481	50.2991
2	25.938	BB	0.8819	8913.86035	150.73836	49.7009
Totals :				1.79350e4	470.23317	

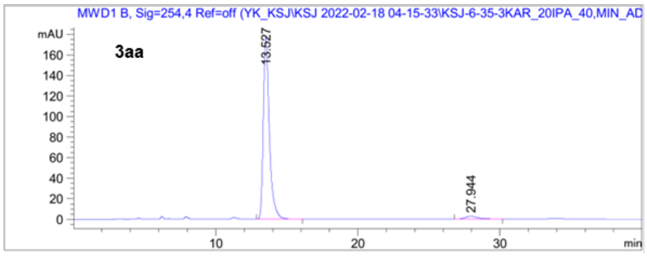

Peak #	RetTime [min]	Type	Width [min]	Area [mAU*s]	Height [mAU]	Area %
1	13.527	BB	0.4419	5239.77246	177.77667	96.9389
2	27.944	BB	0.7602	165.46173	2.64355	3.0611
Totals :				5405.23419	180.42022	

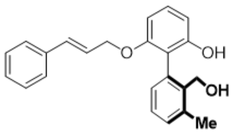

3ba

HPLC conditions:
Chiralpak AD, 40% ^{i}PrOH/Hex eluent 1.0 mL/min, 254 nm

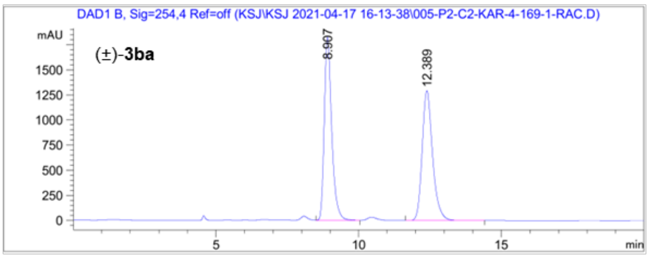

Peak #	RetTime [min]	Type	Width [min]	Area [mAU*s]	Height [mAU]	Area %
1	8.907	VV	0.2718	3.28034e4	1823.87451	49.8702
2	12.389	BB	0.3871	3.29741e4	1294.92188	50.1298
Totals :				6.57775e4	3118.79639	

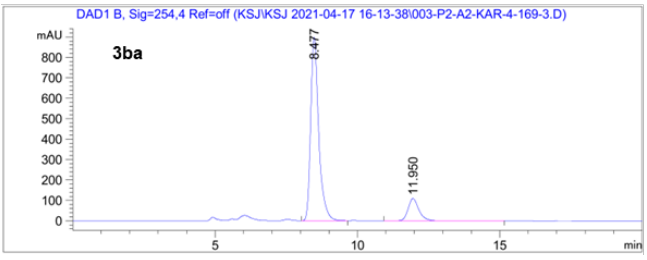

Peak #	RetTime [min]	Type	Width [min]	Area [mAU*s]	Height [mAU]	Area %
1	8.477	BB	0.2925	1.75682e4	897.16504	85.8044
2	11.950	BB	0.4019	2906.51807	109.41774	14.1956
Totals :				2.04748e4	1006.58278	

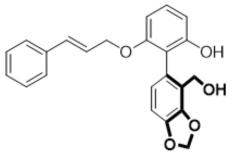

3ca

HPLC conditions:
Chiralpak AD-H, 20% ^{i}PrOH/Hex eluent 1.0 mL/min, 254 nm

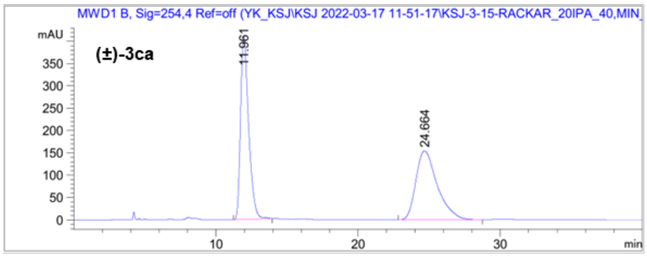

Peak #	RetTime [min]	Type	Width [min]	Area [mAU*s]	Height [mAU]	Area %
1	11.961	BB	0.5962	1.62173e4	409.30511	50.1247
2	24.664	BB	1.5723	1.61365e4	154.27214	49.8753
Totals :				3.23538e4	563.57726	

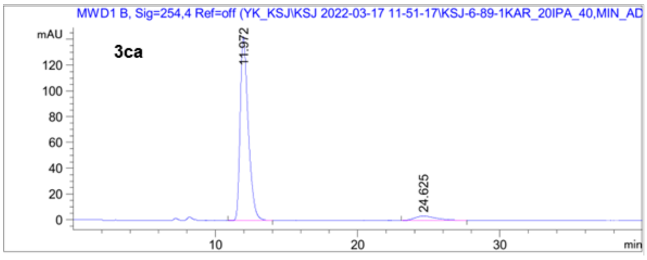

Peak #	RetTime [min]	Type	Width [min]	Area [mAU*s]	Height [mAU]	Area %
1	11.972	BB	0.5933	5640.98096	142.65625	94.1871
2	24.625	BB	1.2308	348.13937	3.33247	5.8129
Totals :				5989.12033	145.98872	

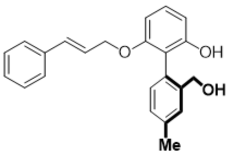

3da

HPLC conditions:
Chiralpak AD-H, 20% ^{i}PrOH/Hex eluent 1.0 mL/min, 254 nm

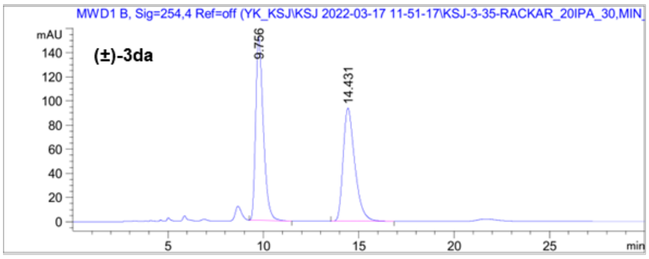

Peak #	RetTime [min]	Type	Width [min]	Area [mAU*s]	Height [mAU]	Area %
1	9.756	BB	0.4242	4247.14258	151.91594	51.5525
2	14.431	BB	0.6444	3991.33252	93.80895	48.4475
Totals :				8238.47510	245.72489	

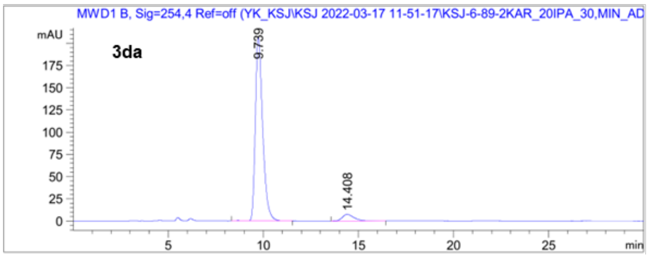

Peak #	RetTime [min]	Type	Width [min]	Area [mAU*s]	Height [mAU]	Area %
1	9.739	VB R	0.4069	5555.76025	207.12422	94.6451
2	14.408	BB	0.6340	314.33517	7.45324	5.3549
Totals :				5870.09543	214.57746	

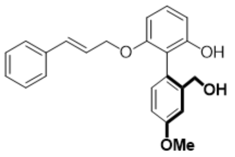

3ea

HPLC conditions:
Chiralpak AD-H, 20% ^{i}PrOH/Hex eluent 1.0 mL/min, 254 nm

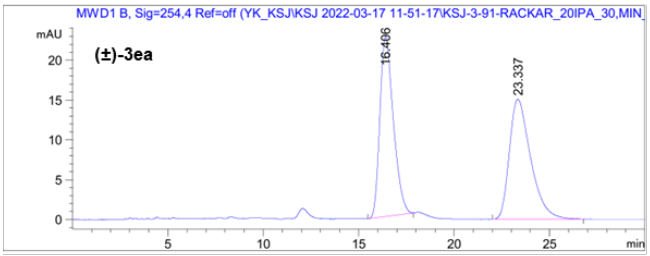

Peak #	RetTime [min]	Type	Width [min]	Area [mAU*s]	Height [mAU]	Area %
1	16.406	BB	0.7363	1107.93970	22.58404	48.7738
2	23.337	BB	1.0703	1163.64612	15.04526	51.2262
Totals :				2271.58582	37.62931	

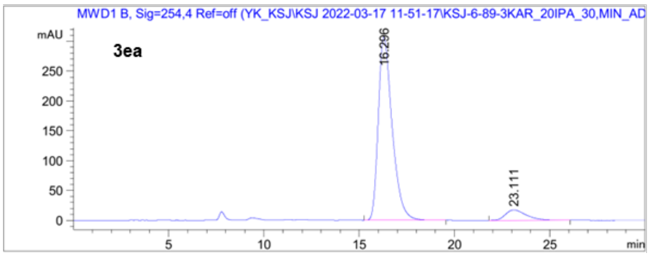

Peak #	RetTime [min]	Type	Width [min]	Area [mAU*s]	Height [mAU]	Area %
1	16.296	BB	0.7668	1.55553e4	307.19278	92.2423
2	23.111	BB	1.0757	1308.23035	17.42958	7.7577
Totals :				1.68635e4	324.62236	

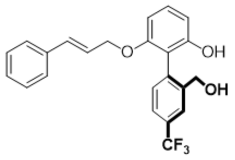

3fa

HPLC conditions:
Chiralpak AD-H, 20% ^{i}PrOH/Hex eluent 1.0 mL/min, 254 nm

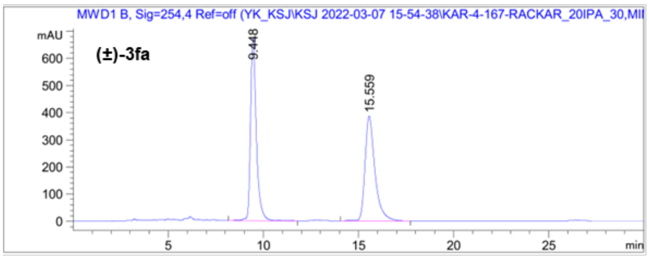

Peak #	RetTime [min]	Type	Width [min]	Area [mAU*s]	Height [mAU]	Area %
1	9.448	VV R	0.3141	1.42335e4	674.75195	50.3959
2	15.559	VB R	0.5437	1.40098e4	385.79034	49.6041
Totals :				2.82433e4	1060.54230	

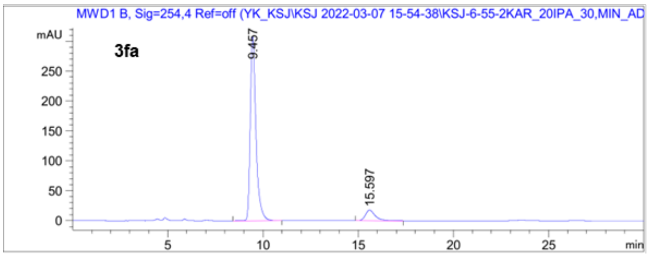

Peak #	RetTime [min]	Type	Width [min]	Area [mAU*s]	Height [mAU]	Area %
1	9.457	BB	0.3108	6343.89795	307.40265	91.0773
2	15.597	BB	0.5299	621.49963	17.51854	8.9227
Totals :				6965.39758	324.92119	

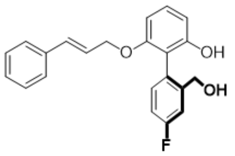

3ga

HPLC conditions:
Chiralpak AD-H, 20% ^{i}PrOH/Hex eluent 1.0 mL/min, 254 nm

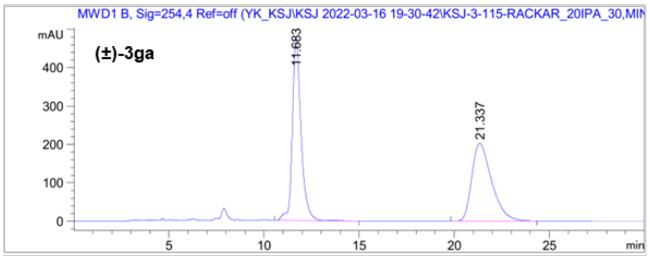

Peak #	RetTime [min]	Type	Width [min]	Area [mAU*s]	Height [mAU]	Area %
1	11.683	BV R	0.4793	1.53100e4	478.52414	51.2433
2	21.337	VB R	1.0883	1.45671e4	202.25385	48.7567
Totals :				2.98771e4	680.77798	

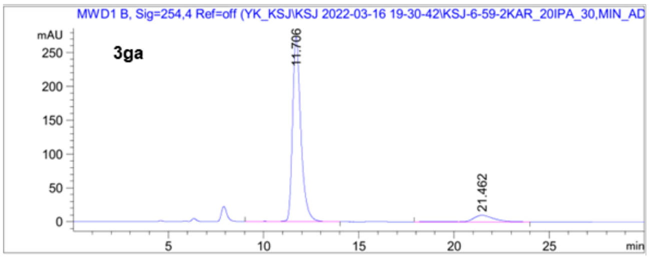

Peak #	RetTime [min]	Type	Width [min]	Area [mAU*s]	Height [mAU]	Area %
1	11.706	VV R	0.4618	8319.99902	272.82510	92.0602
2	21.462	VV R	1.0426	717.56384	9.66052	7.9398
Totals :				9037.56287	282.48562	

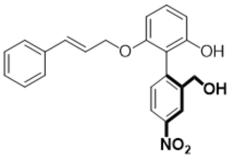

3ha

HPLC conditions:
Chiralpak AD-H, 20% ^{i}PrOH/Hex eluent 1.0 mL/min, 254 nm

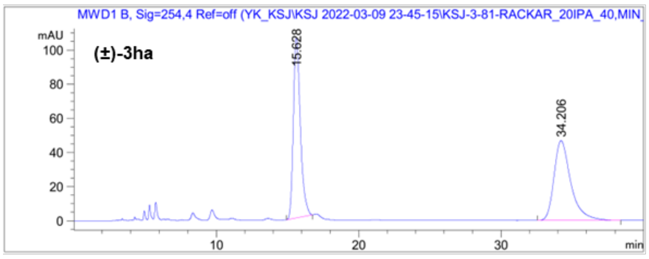

Peak #	RetTime [min]	Type	Width [min]	Area [mAU*s]	Height [mAU]	Area %
1	15.628	BB	0.5202	3603.62012	105.56782	48.6096
2	34.206	BB	1.2046	3809.77808	46.65738	51.3904
Totals :				7413.39819	152.22520	

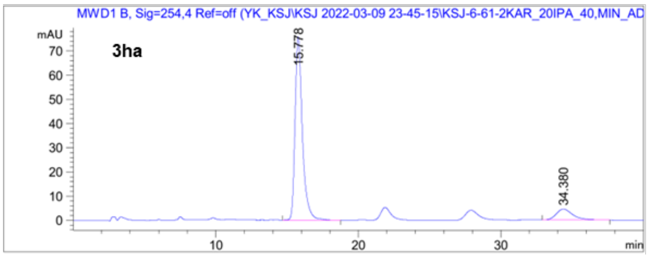

Peak #	RetTime [min]	Type	Width [min]	Area [mAU*s]	Height [mAU]	Area %
1	15.778	BB	0.5361	2702.53687	75.76680	88.1024
2	34.380	BB	1.1614	364.95895	4.51341	11.8976
Totals :				3067.49582	80.28021	

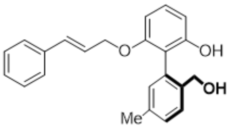

3ia

HPLC conditions:
Chiralpak AD, 20% ^{i}PrOH/Hex eluent 1.0 mL/min, 254 nm

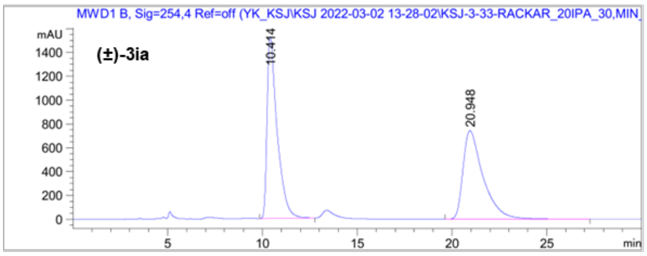

Peak #	RetTime [min]	Type	Width [min]	Area [mAU*s]	Height [mAU]	Area %
1	10.414	BB	0.5328	5.62888e4	1524.46252	50.7635
2	20.948	BB	1.0849	5.45956e4	741.61603	49.2365
Totals :				1.10884e5	2266.07855	

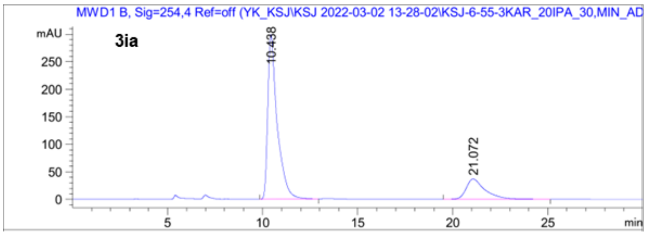

Peak #	RetTime [min]	Type	Width [min]	Area [mAU*s]	Height [mAU]	Area %
1	10.438	BB	0.4966	1.03185e4	298.99313	80.1581
2	21.072	BB	0.9947	2554.17627	36.93917	19.8419
Totals :				1.28727e4	335.93231	

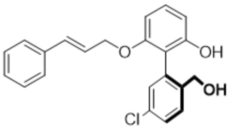

3ja

HPLC conditions:
Chiralpak AD-H, 20% ^{i}PrOH/Hex eluent 1.0 mL/min, 254 nm

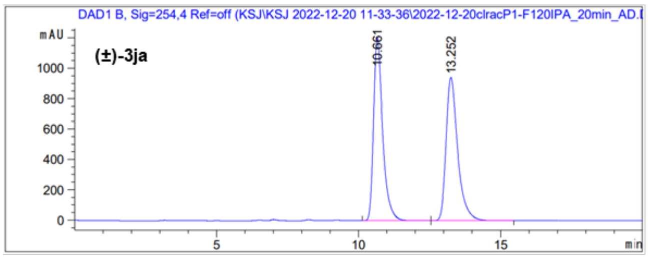

Peak #	RetTime [min]	Type	Width [min]	Area [mAU*s]	Height [mAU]	Area %
1	10.661	BB	0.3356	2.68326e4	1205.84363	49.9932
2	13.252	BB	0.4277	2.68399e4	943.96545	50.0068
Totals :				5.36725e4	2149.80908	

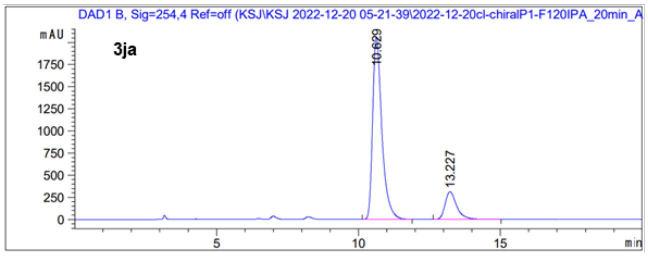

Peak #	RetTime [min]	Type	Width [min]	Area [mAU*s]	Height [mAU]	Area %
1	10.629	BB	0.3419	4.65114e4	2056.13672	84.0699
2	13.227	BB	0.4242	8813.25000	313.28601	15.9301
Totals :				5.53247e4	2369.42273	

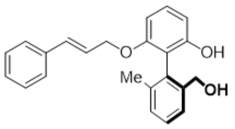

3ka

HPLC conditions:
Chiralpak AD, 20% ^{i}PrOH/Hex eluent 1.0 mL/min, 254 nm

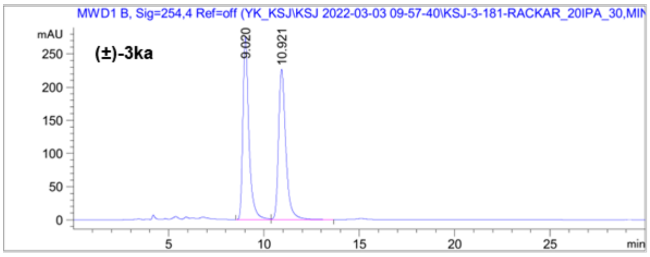

Peak #	RetTime [min]	Type	Width [min]	Area [mAU*s]	Height [mAU]	Area %
1	9.020	BV	0.3298	6093.57861	275.71628	50.4142
2	10.921	VB	0.3988	5993.45068	226.40630	49.5858
Totals :				1.20870e4	502.12257	

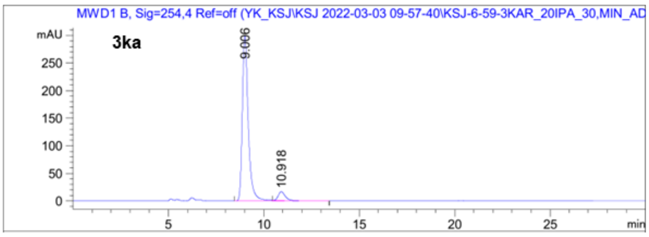

Peak #	RetTime [min]	Type	Width [min]	Area [mAU*s]	Height [mAU]	Area %
1	9.006	BV R	0.3268	6488.41553	299.48651	93.8524
2	10.918	VB E	0.3965	425.01001	16.17825	6.1476
Totals :				6913.42554	315.66476	

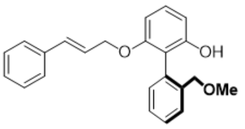

3la

HPLC conditions:
Chiralpak AD-H, 20% ^{i}PrOH/Hex eluent 1.0 mL/min, 254 nm

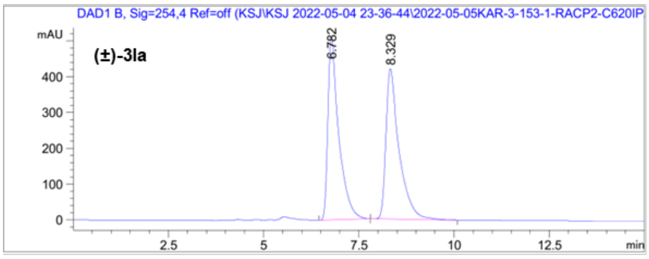

Peak #	RetTime [min]	Type	Width [min]	Area [mAU*s]	Height [mAU]	Area %
1	6.782	BB	0.2817	1.00852e4	512.21265	49.9616
2	8.329	BB	0.3440	1.01007e4	420.82169	50.0384
Totals :				2.01859e4	933.03433	

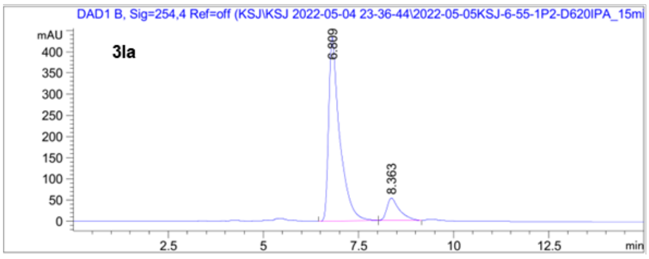

Peak #	RetTime [min]	Type	Width [min]	Area [mAU*s]	Height [mAU]	Area %
1	6.809	BB	0.2869	8728.98047	433.57666	88.0133
2	8.363	BB	0.3304	1188.81921	52.46060	11.9867
Totals :				9917.79968	486.03726	

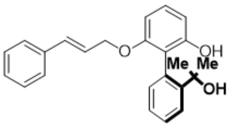

3ma

HPLC conditions:
Chiralpak AD-H, 20% ^{i}PrOH/Hex eluent 1.0 mL/min, 254 nm

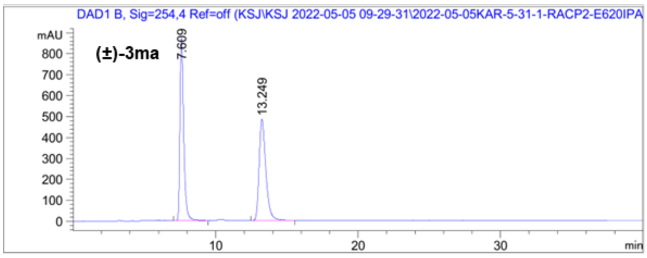

Peak #	RetTime [min]	Type	Width [min]	Area [mAU*s]	Height [mAU]	Area %
1	7.609	BB	0.2783	1.60548e4	874.10455	50.4110
2	13.249	BB	0.4951	1.57931e4	483.49255	49.5890
Totals :				3.18479e4	1357.59711	

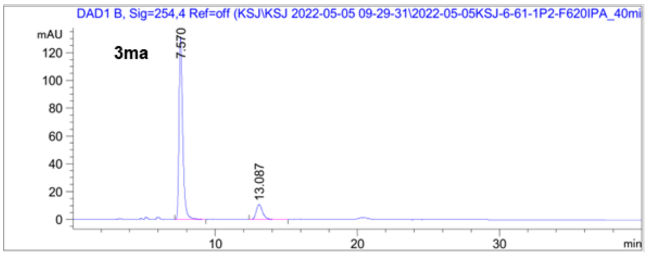

Peak #	RetTime [min]	Type	Width [min]	Area [mAU*s]	Height [mAU]	Area %
1	7.570	BB	0.2761	2389.90454	131.47975	87.6969
2	13.087	BB	0.4702	335.28366	10.74138	12.3031
Totals :				2725.18820	142.22114	

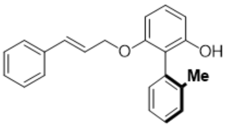

3na

HPLC conditions:
Chiralpak AD-H, 20% ^{i}PrOH/Hex eluent 1.0 mL/min, 254 nm

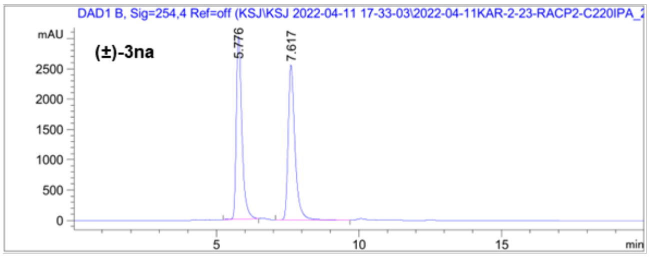

Peak #	RetTime [min]	Type	Width [min]	Area [mAU*s]	Height [mAU]	Area %
1	5.776	VB R	0.2075	4.12447e4	3011.06201	48.9596
2	7.617	BB	0.2542	4.29976e4	2556.54297	51.0404
Totals :				8.42423e4	5567.60498	

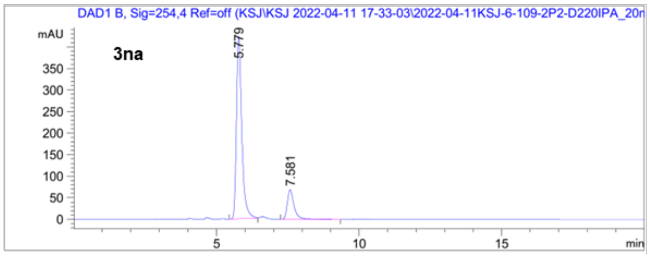

Peak #	RetTime [min]	Type	Width [min]	Area [mAU*s]	Height [mAU]	Area %
1	5.779	BB	0.1948	5469.80225	423.18732	82.8255
2	7.581	BB	0.2456	1134.20496	69.07980	17.1745
Totals :				6604.00720	492.26712	

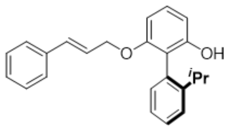

3oa

HPLC conditions:
Chiralpak AD-H, 7% ^{i}PrOH/Hex eluent 1.0 mL/min, 254 nm

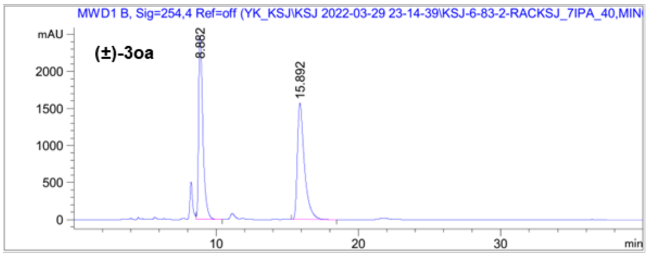

Peak #	RetTime [min]	Type	Width [min]	Area [mAU*s]	Height [mAU]	Area %
1	8.882	VB	0.3125	5.13554e4	2470.68726	48.9259
2	15.892	BB	0.5031	5.36103e4	1575.16638	51.0741
Totals :				1.04966e5	4045.85364	

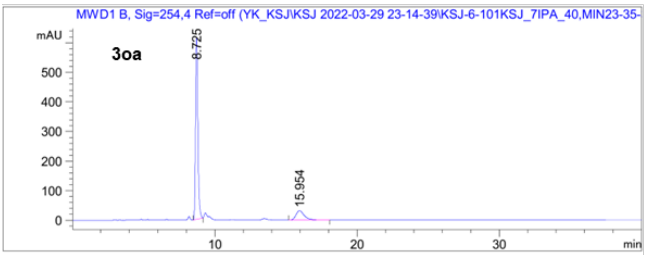

Peak #	RetTime [min]	Type	Width [min]	Area [mAU*s]	Height [mAU]	Area %
1	8.725	BB	0.1740	6951.40332	614.60876	84.7040
2	15.954	BB	0.5976	1255.29761	31.58689	15.2960
Totals :				8206.70093	646.19565	

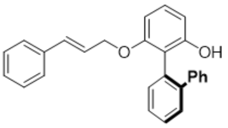

3pa

HPLC conditions:
Chiralpak AD-H, 10% ^{i}PrOH/Hex eluent 1.0 mL/min, 254 nm

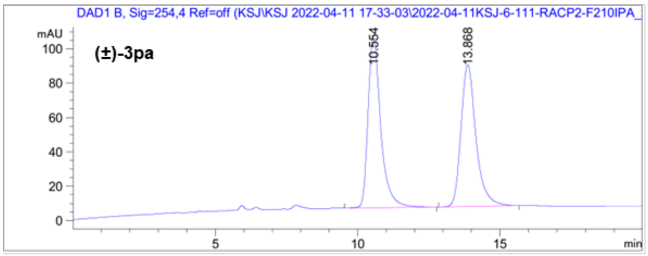

Peak #	RetTime [min]	Type	Width [min]	Area [mAU*s]	Height [mAU]	Area %
1	10.554	BB	0.4646	3090.72144	99.98606	50.5402
2	13.868	BB	0.5555	3024.64868	82.53792	49.4598
Totals :				6115.37012	182.52398	

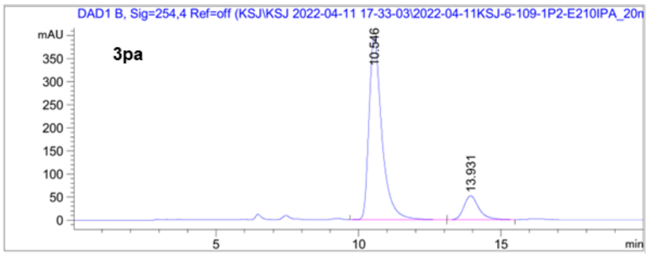

Peak #	RetTime [min]	Type	Width [min]	Area [mAU*s]	Height [mAU]	Area %
1	10.546	BB	0.4661	1.23587e4	396.03085	86.7775
2	13.931	BB	0.5525	1883.12634	51.75786	13.2225
Totals :				1.42418e4	447.78872	

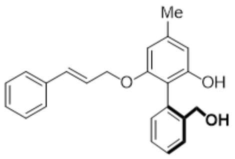

3qa

HPLC conditions:
Chiralpak AD-H, 20% ^{i}PrOH/Hex eluent 1.0 mL/min, 254 nm

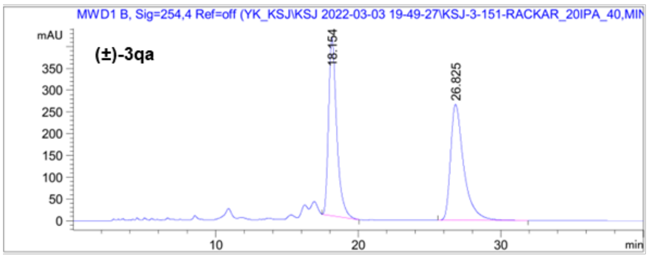

Peak #	RetTime [min]	Type	Width [min]	Area [mAU*s]	Height [mAU]	Area %
1	18.154	BB	0.6015	1.62663e4	409.46597	48.7447
2	26.825	BB	0.9565	1.71041e4	265.87854	51.2553
Totals :				3.33704e4	675.34451	

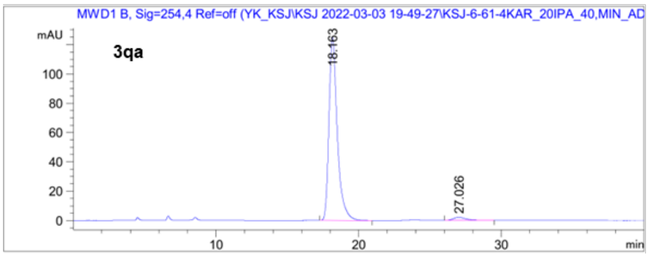

Peak #	RetTime [min]	Type	Width [min]	Area [mAU*s]	Height [mAU]	Area %
1	18.163	BB	0.6090	5066.22510	124.95633	97.5241
2	27.026	BB	0.7274	128.61652	2.07993	2.4759
Totals :				5194.84161	127.03626	

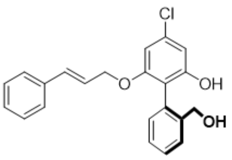

3ra

HPLC conditions:
Chiralpak AD-H, 20% ^{i}PrOH/Hex eluent 1.0 mL/min, 254 nm

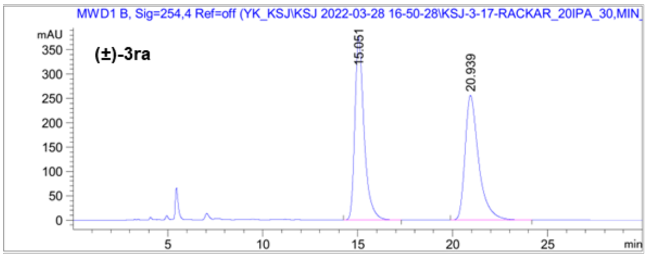

Peak #	RetTime [min]	Type	Width [min]	Area [mAU*s]	Height [mAU]	Area %
1	15.051	BB	0.5089	1.27254e4	375.97147	49.9900
2	20.939	BB	0.7465	1.27305e4	255.87117	50.0100
Totals :				2.54559e4	631.84264	

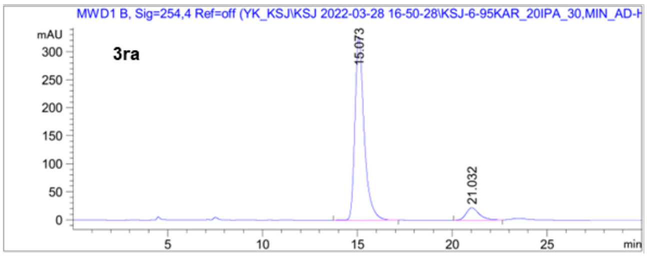

Peak #	RetTime [min]	Type	Width [min]	Area [mAU*s]	Height [mAU]	Area %
1	15.073	BB	0.5082	1.10501e4	327.07220	91.4617
2	21.032	BB	0.7073	1031.57483	21.83207	8.5383
Totals :				1.20817e4	348.90427	

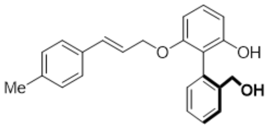

3ab

HPLC conditions:
Chiralpak AD-H, 20% ^{i}PrOH/Hex eluent 1.0 mL/min, 254 nm

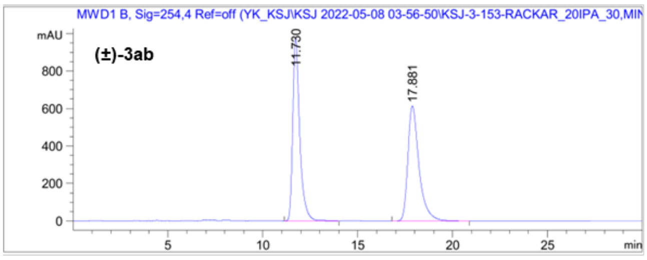

Peak #	RetTime [min]	Type	Width [min]	Area [mAU*s]	Height [mAU]	Area %
1	11.730	BV R	0.3812	2.49893e4	978.51331	49.9488
2	17.881	BB	0.6104	2.50405e4	613.19727	50.0512
Totals :				5.00297e4	1591.71057	

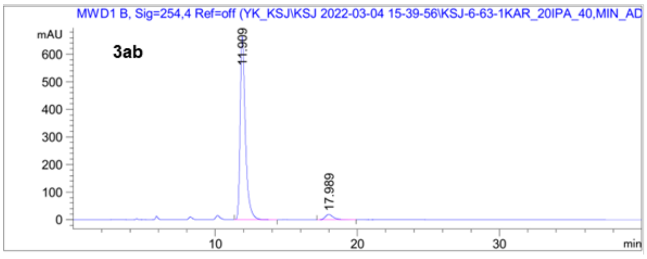

Peak #	RetTime [min]	Type	Width [min]	Area [mAU*s]	Height [mAU]	Area %
1	11.909	BB	0.3825	1.69705e4	663.33667	95.7237
2	17.989	BB	0.5987	758.12329	19.11675	4.2763
Totals :				1.77286e4	682.45342	

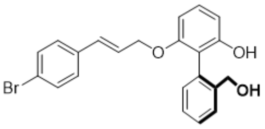

3ac

HPLC conditions:
Chiralpak IC, 10% ^{i}PrOH/Hex eluent 1.0 mL/min, 254 nm

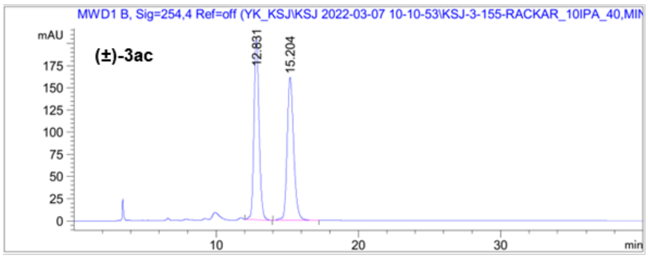

Peak #	RetTime [min]	Type	Width [min]	Area [mAU*s]	Height [mAU]	Area %
1	12.831	BB	0.3815	5101.55225	205.61623	49.8488
2	15.204	BB	0.4900	5132.50488	160.94841	50.1512
Totals :				1.02341e4	366.56464	

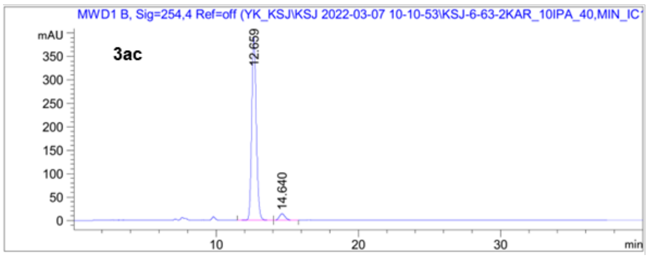

Peak #	RetTime [min]	Type	Width [min]	Area [mAU*s]	Height [mAU]	Area %
1	12.659	BB	0.3482	8756.62988	389.58270	95.8284
2	14.640	BB	0.4288	381.19745	13.69174	4.1716
Totals :				9137.82733	403.27444	

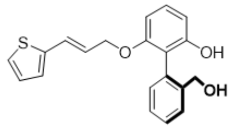

3ad

HPLC conditions:
Chiralpak AD-H, 20% ^{i}PrOH/Hex eluent 1.0 mL/min, 254 nm

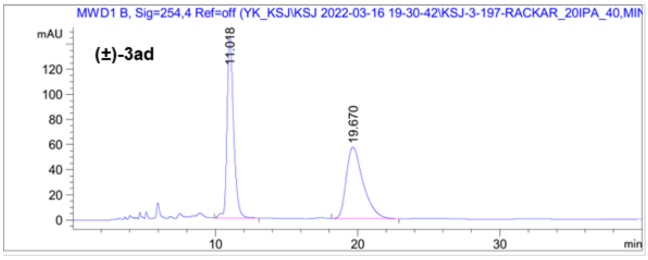

Peak #	RetTime [min]	Type	Width [min]	Area [mAU*s]	Height [mAU]	Area %
1	11.018	VB R	0.4817	4643.29541	144.97279	49.8125
2	19.670	BB	1.2218	4678.25488	56.98533	50.1875
Totals :				9321.55029	201.95813	

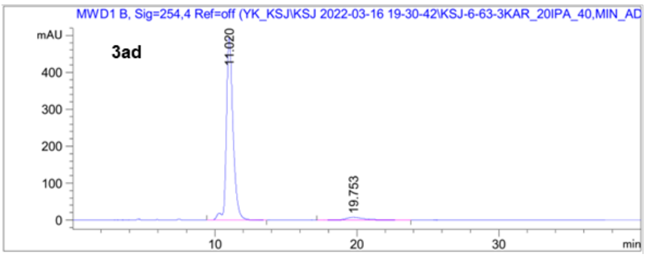

Peak #	RetTime [min]	Type	Width [min]	Area [mAU*s]	Height [mAU]	Area %
1	11.020	VB R	0.4928	1.61360e4	494.39917	96.0922
2	19.753	BB	1.2928	656.21436	7.50017	3.9078
Totals :				1.67922e4	501.89934	

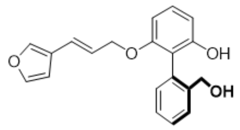

3ae

HPLC conditions:
Chiralpak AD-H, 10% ^{i}PrOH/Hex eluent 1.0 mL/min, 254 nm

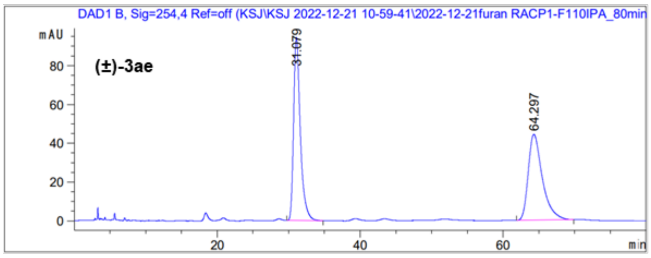

Peak #	RetTime [min]	Type	Width [min]	Area [mAU*s]	Height [mAU]	Area %
1	31.079	BB	0.9808	6172.13770	94.40088	50.4052
2	64.297	BB	1.8594	6072.90381	44.24305	49.5948
Totals :				1.22450e4	138.64393	

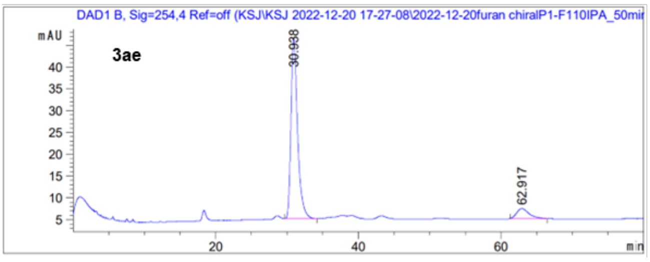

Peak #	RetTime [min]	Type	Width [min]	Area [mAU*s]	Height [mAU]	Area %
1	30.938	BB	0.9666	2663.58325	41.40271	90.6983
2	62.917	BB	1.3951	273.16708	2.30422	9.3017
Totals :				2936.75034	43.70692	

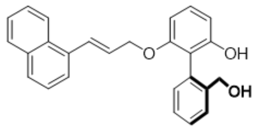

3af

HPLC conditions:
Chiralpak IC, 10% ^{i}PrOH/Hex eluent 1.0 mL/min, 320 nm

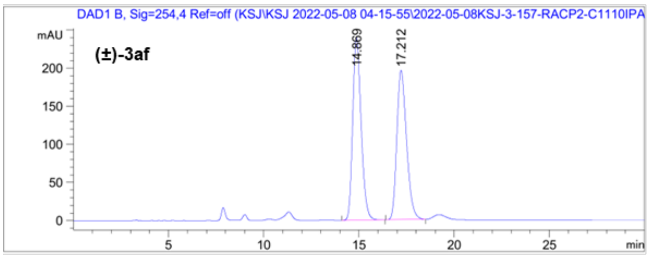

Peak #	RetTime [min]	Type	Width [min]	Area [mAU*s]	Height [mAU]	Area %
1	14.869	BB	0.4734	7346.28613	239.77858	51.4317
2	17.212	BB	0.5474	6937.28125	195.78465	48.5683
Totals :				1.42836e4	435.56323	

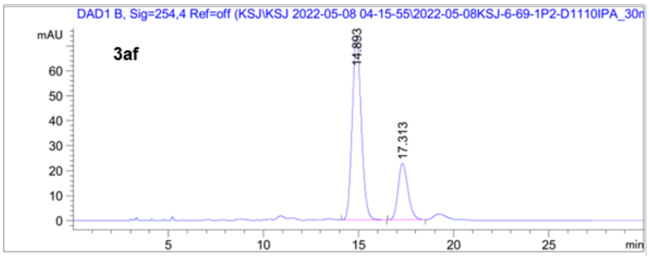

Peak #	RetTime [min]	Type	Width [min]	Area [mAU*s]	Height [mAU]	Area %
1	14.893	BB	0.5129	2440.75366	73.21312	74.9883
2	17.313	BB	0.5529	814.09094	22.67403	25.0117
Totals :				3254.84460	95.88715	

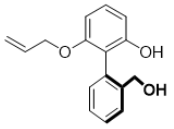

3ag

HPLC conditions:
Chiralpak AD-H, 20% ^{i}PrOH/Hex eluent 1.0 mL/min, 254 nm

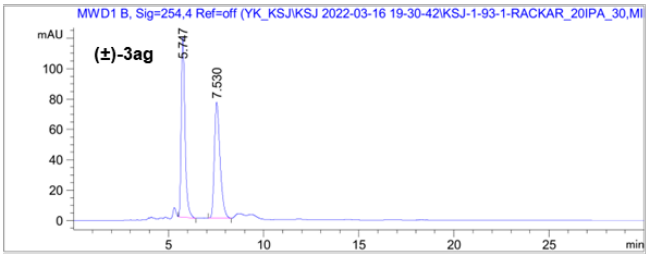

Peak #	RetTime [min]	Type	Width [min]	Area [mAU*s]	Height [mAU]	Area %
1	5.747	BB	0.2052	1620.55493	118.76287	50.4162
2	7.530	BB	0.3154	1593.79651	76.40486	49.5838
Totals :				3214.35144	195.16773	

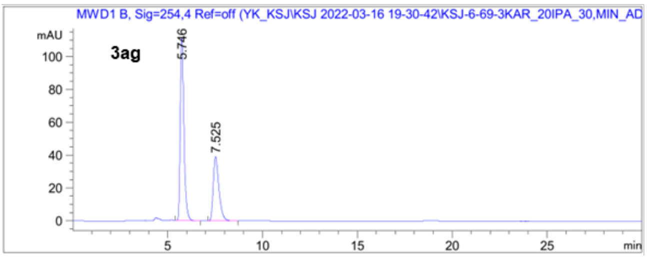

Peak #	RetTime [min]	Type	Width [min]	Area [mAU*s]	Height [mAU]	Area %
1	5.746	BB	0.2048	1501.09827	111.65788	64.7539
2	7.525	BB	0.3148	817.06201	39.25795	35.2461
Totals :				2318.16028	150.91583	

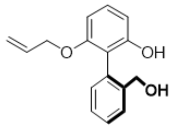

3ag

HPLC conditions:
Chiralpak AD-H, 20% ^{i}PrOH/Hex eluent 1.0 mL/min, 254 nm

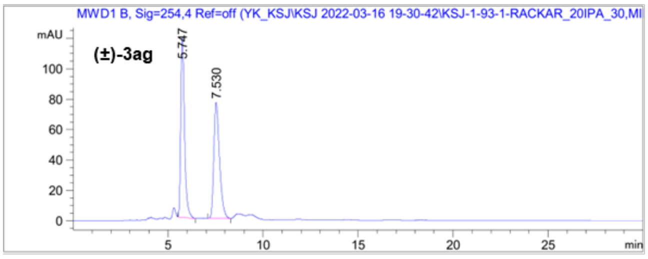

Peak #	RetTime [min]	Type	Width [min]	Area [mAU*s]	Height [mAU]	Area %
1	5.747	BB	0.2052	1620.55493	118.76287	50.4162
2	7.530	BB	0.3154	1593.79651	76.40486	49.5838
Totals :				3214.35144	195.16773	

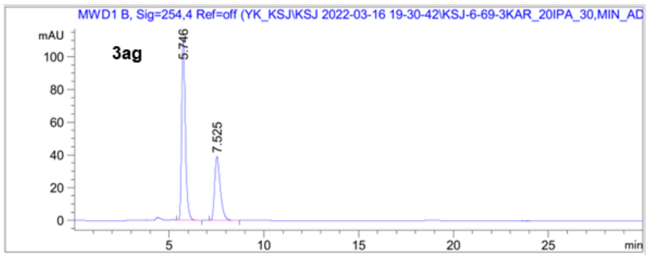

Peak #	RetTime [min]	Type	Width [min]	Area [mAU*s]	Height [mAU]	Area %
1	6.203	BB	0.2322	524.79028	33.60233	61.6378
2	8.008	BB	0.3005	326.61908	16.11167	38.3622
Totals :				851.40936	49.71401	

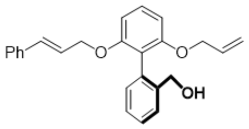

4ag’

HPLC conditions:
Chiralpak AD-H, 20% ^{i}PrOH/Hex eluent 1.0 mL/min, 254 nm

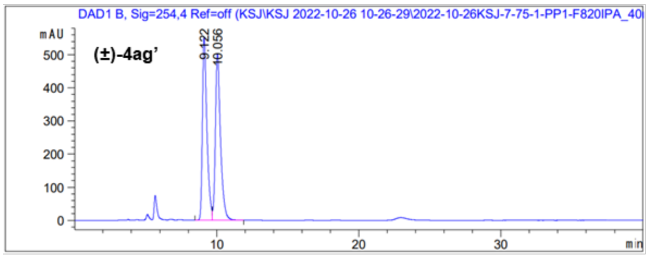

Peak #	RetTime [min]	Type	Width [min]	Area [mAU*s]	Height [mAU]	Area %
1	9.122	BV	0.3284	1.22375e4	552.59009	49.2104
2	10.056	VB	0.3707	1.26302e4	503.42493	50.7896
Totals :				2.48677e4	1056.01501	

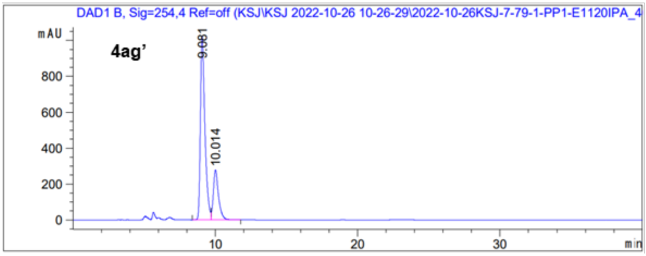

Peak #	RetTime [min]	Type	Width [min]	Area [mAU*s]	Height [mAU]	Area %
1	9.081	BV	0.3319	2.27985e4	1023.35883	76.3367
2	10.014	VB	0.3725	7067.18799	278.03677	23.6633
Totals :				2.98656e4	1301.39560	

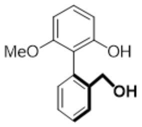

3s

HPLC conditions:
Chiralpak AD-H, 20% ^{i}PrOH/Hex eluent 1.0 mL/min, 254 nm

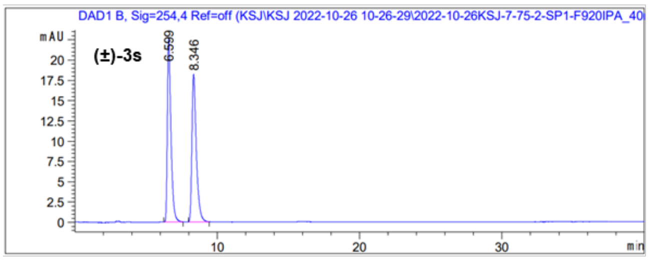

Peak #	RetTime [min]	Type	Width [min]	Area [mAU*s]	Height [mAU]	Area %
1	6.599	BB	0.2555	389.35138	22.54174	49.9677
2	8.346	BB	0.3163	389.85461	18.17473	50.0323
Totals :				779.20599	40.71647	

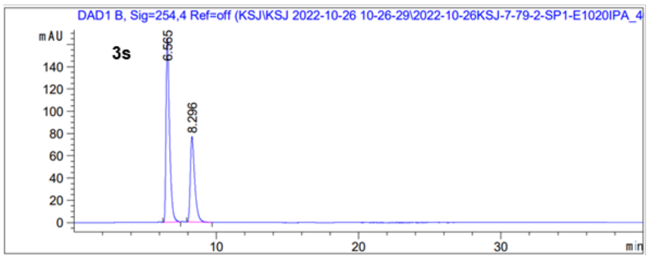

Peak #	RetTime [min]	Type	Width [min]	Area [mAU*s]	Height [mAU]	Area %
1	6.565	BB	0.2498	2791.90967	164.68750	63.0308
2	8.296	BB	0.3155	1637.52686	76.58215	36.9692
Totals :				4429.43652	241.26965	

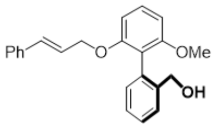

4sa

HPLC conditions:
Chiralpak AD-H, 5% ^{i}PrOH/Hex eluent 1.0 mL/min, 254 nm

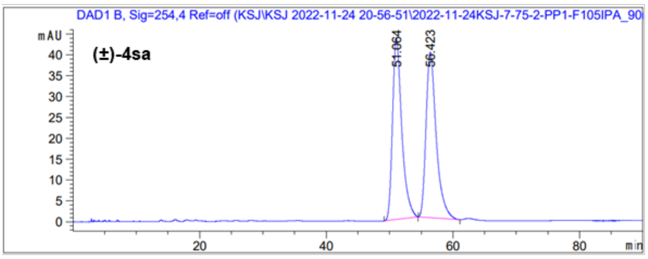

Peak #	RetTime [min]	Type	Width [min]	Area [mAU*s]	Height [mAU]	Area %
1	51.064	BB	1.5126	4412.77881	43.38794	49.7731
2	56.423	BB	1.5582	4453.01953	39.65257	50.2269
Totals :				8865.79834	83.04051	

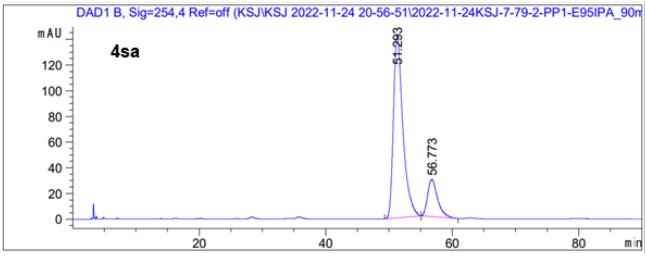

Peak #	RetTime [min]	Type	Width [min]	Area [mAU*s]	Height [mAU]	Area %
1	51.293	BB	1.5497	1.47536e4	141.58809	82.3617
2	56.773	BB	1.5139	3159.56494	29.02782	17.6383
Totals :				1.79131e4	170.61591	

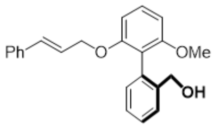

4sa

HPLC conditions:
Chiralpak AD-H, 5% ^{i}PrOH/Hex eluent 1.0 mL/min, 254 nm

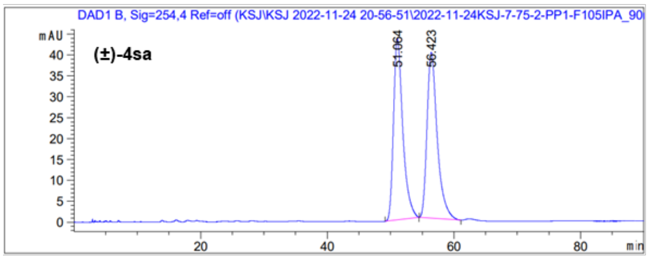

Peak #	RetTime [min]	Type	Width [min]	Area [mAU*s]	Height [mAU]	Area %
1	51.064	BB	1.5126	4412.77881	43.38794	49.7731
2	56.423	BB	1.5582	4453.01953	39.65257	50.2269
Totals :				8865.79834	83.04051	

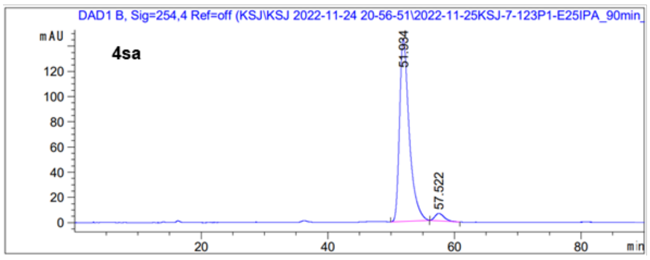

Peak #	RetTime [min]	Type	Width [min]	Area [mAU*s]	Height [mAU]	Area %
1	51.934	BB	1.5428	1.53792e4	144.83749	96.3282
2	57.522	BB	1.1723	586.22162	5.92504	3.6718
Totals :				1.59655e4	150.76253	

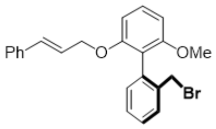

5

HPLC conditions:
Chiralpak OD-H, 5% ^{i}PrOH/Hex eluent 1.0 mL/min, 254 nm

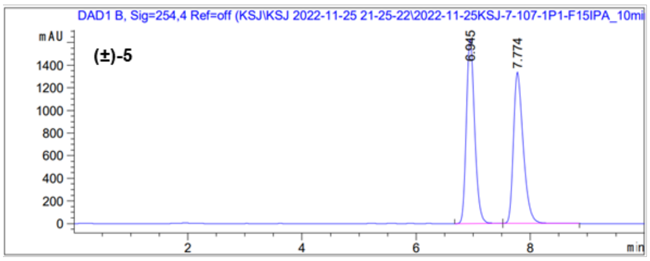

Peak #	RetTime [min]	Type	Width [min]	Area [mAU*s]	Height [mAU]	Area %
1	6.945	BB	0.1541	1.62295e4	1630.68469	49.9912
2	7.774	BB	0.1857	1.62352e4	1336.91589	50.0088
Totals :				3.24647e4	2967.60059	

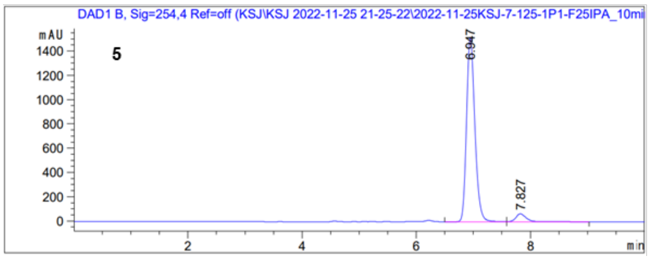

Peak #	RetTime [min]	Type	Width [min]	Area [mAU*s]	Height [mAU]	Area %
1	6.947	BV	0.1526	1.51776e4	1518.26184	94.8060
2	7.827	VB	0.1939	831.51813	64.72313	5.1940
Totals :				1.60091e4	1582.98497	

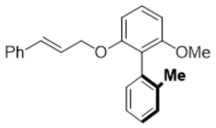

6

HPLC conditions:
Chiralpak AD-H, 1% ^{i}PrOH/Hex eluent 1.0 mL/min, 254 nm

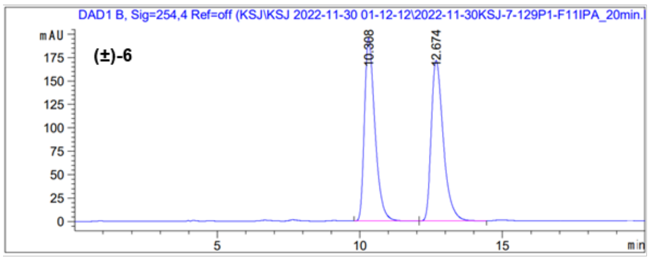

Peak #	RetTime [min]	Type	Width [min]	Area [mAU*s]	Height [mAU]	Area %
1	10.308	BB	0.3934	5009.02295	195.15477	49.9197
2	12.674	BB	0.4478	5025.13623	171.54791	50.0803
Totals :				1.00342e4	366.70268	

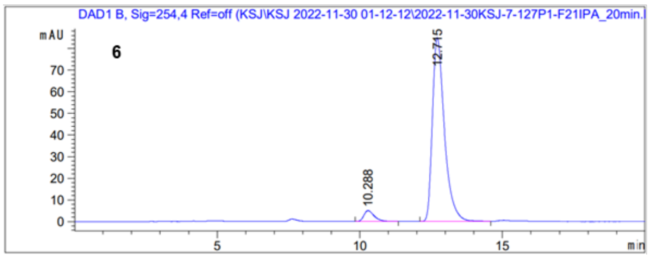

Peak #	RetTime [min]	Type	Width [min]	Area [mAU*s]	Height [mAU]	Area %
1	10.288	BB	0.3835	127.93231	5.05025	4.8916
2	12.715	BB	0.4436	2487.39673	84.94601	95.1084
Totals :				2615.32904	89.99625	

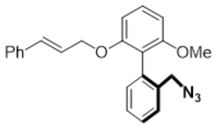

7

HPLC conditions:
Chiralpak AD-H, 1% ^{i}PrOH/Hex eluent 1.0 mL/min, 254 nm

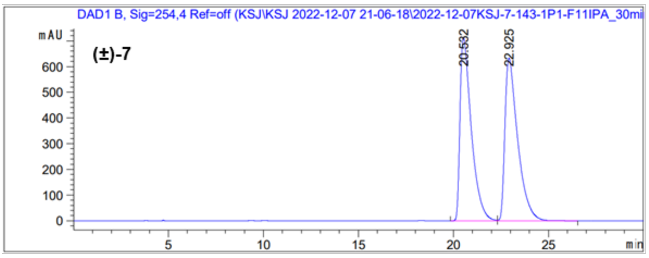

Peak #	RetTime [min]	Type	Width [min]	Area [mAU*s]	Height [mAU]	Area %
1	20.532	BV	0.6181	2.94362e4	712.32629	49.9484
2	22.925	VB	0.6894	2.94971e4	633.28076	50.0516
Totals :				5.89333e4	1345.60706	

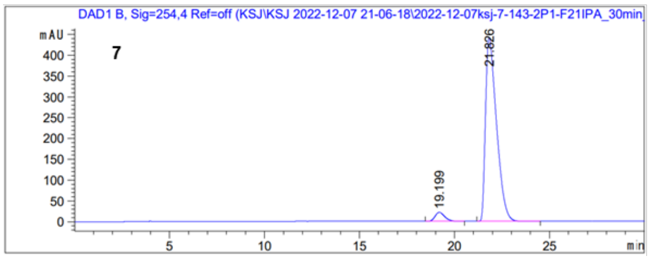

Peak #	RetTime [min]	Type	Width [min]	Area [mAU*s]	Height [mAU]	Area %
1	19.199	BB	0.5381	759.62054	21.60794	4.1176
2	21.826	BB	0.6117	1.76886e4	441.20663	95.8824
Totals :				1.84482e4	462.81457	
