# Supplementary material for: Atroposelective desymmetrization of 2-arylresorcinols via Tsuji-Trost allylation
Source: Commun Chem. 2023 Feb 25;6:42. doi: 10.1038/s42004-023-00839-z (PMC9968306; doi:10.1038/s42004-023-00839-z)
Supplement: Supplementary file 6 — Supplementary Data 4 [file 42004_2023_839_MOESM6_ESM.pdf]

Computational Chemistry Data

1 Computational Studies to Determine Rotational Barriers

1.1 General Comments

All calculations were carried out using Gaussian 16[1] and all visualization of input and output files was accomplished using GaussView 6.0.[2] All geometry optimizations and transition state optimizations were performed with B3LYP[3-5] functional using 6-31+G(d,p) basis set. The vibrational frequencies were computed at the same level of theory as for the geometry optimizations and to evaluate the zero-point vibrational energy (ZPVE) and thermal corrections at 298 K. The single-point energies were computed based on the gas-phase optimized structures, using M06-2X[6] using 6-311++G(2d,3p) basis set. Rendering of computational outputs was accomplished using CYLview 20.[7]

1.2 Calculation of Rotational Barrier for 1a

Supplementary Table 14. Calculation of rotational barrier for 1a

Structure	G^a (Ha)	Thermal correction^b	G_{corr} (Ha)	$\Delta G_{corr}^{\ddagger}$ (Ha)
**GS1**	−728.22083768	0.183298	−728.03753968	
**TS1**	−728.17076190	0.184919	−727.98584290	0.05169678
**GS2**	−728.22083945	0.183296	−728.03754345	
**TS2**	−728.17076238	0.184920	−727.98584238	0.05169730

^{a}Computed at M06-2X/6-311++G(2d,3p) level of theory. ^{b}Computed at 298 K and B3LYP/6-31+G(d,p) level of theory.

$\Delta G_{corr}^{\ddagger}$ (1) = 32.440 kcal/mol → $k_1 = 1.115 \times 10^{-11}$ s^{-1}
$\Delta G_{corr}^{\ddagger}$ (2) = 32.440 kcal/mol → $k_2 = 1.115 \times 10^{-11}$ s^{-1}

$k_{obs} = k_1 + k_2 = 2.230 \times 10^{-11}$ s^{-1} → **$\Delta G_{obs}^{\ddagger}$ = 32.029 kcal/mol**

GS1 Optimization

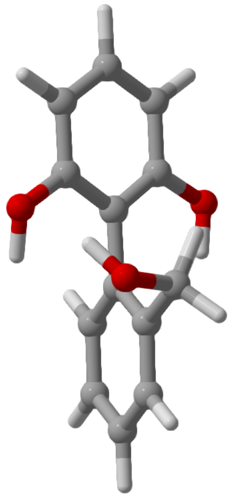

GS1 Cartesian coordinates

C	3.5125270000	-0.7473650000	0.3232550000
C	2.9492920000	0.2542630000	-0.4681910000
H	3.5948560000	0.9199290000	-1.0351070000
C	1.5602570000	0.4271340000	-0.5544280000
C	0.7174450000	-0.4348440000	0.1900330000
C	1.2979690000	-1.4424720000	0.9853420000
H	0.6475680000	-2.0966810000	1.5602390000
C	2.6831890000	-1.6028170000	1.0521950000
H	3.1089730000	-2.3866650000	1.6715540000
C	1.0076970000	1.5434510000	-1.4258030000
H	1.6789510000	1.7030380000	-2.2740360000
H	0.0233440000	1.2663170000	-1.8216900000
C	-0.7743160000	-0.3038230000	0.1512830000
C	-1.5708710000	-1.2317620000	-0.5484260000
C	-2.9617480000	-1.0990160000	-0.5984810000
H	-3.5394670000	-1.8291770000	-1.1542760000
C	-3.5687860000	-0.0310540000	0.0602510000
H	-4.6488260000	0.0746130000	0.0224800000
C	-2.8128100000	0.9005450000	0.7744520000
H	-3.2730780000	1.7275320000	1.3033530000
C	-1.4273950000	0.7497710000	0.8163680000
O	0.9466700000	2.8039200000	-0.7529530000
H	0.2512150000	2.7648960000	-0.0777100000
O	-1.0185540000	-2.2844280000	-1.2244930000
H	-0.0553630000	-2.2711190000	-1.1205380000
O	-0.7060870000	1.6994600000	1.5121240000
H	4.5916560000	-0.8613690000	0.3686450000
H	0.1692330000	1.3450000000	1.7325630000

TS1 Optimization

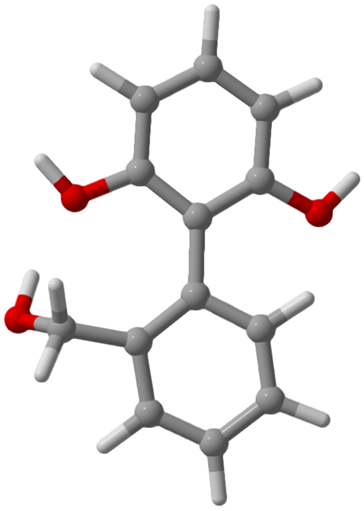

TS1 Cartesian coordinates

C	3.3859420000	-1.4471200000	0.0250580000
C	3.0913110000	-0.0916090000	-0.0244570000
H	3.9120190000	0.6192520000	0.0061250000
C	1.7916850000	0.4377730000	-0.1556710000
C	0.6660530000	-0.4677870000	-0.1213620000
C	1.0254420000	-1.8413350000	-0.1980220000
H	0.2574520000	-2.5803560000	-0.3147730000
C	2.3253690000	-2.3299320000	-0.1277060000
H	2.4886360000	-3.4022950000	-0.1871730000
C	1.8613300000	1.9409410000	-0.4370650000
H	2.8314850000	2.1335320000	-0.8996830000
H	1.1002660000	2.2510360000	-1.1526750000
C	-0.8359690000	-0.1766530000	-0.0453260000
C	-1.8233000000	-1.2133380000	0.1231500000
C	-3.2036340000	-0.9992830000	0.0072850000
H	-3.8686720000	-1.8562420000	0.0866460000
C	-3.7217570000	0.2713600000	-0.1792390000
H	-4.7901620000	0.4325080000	-0.2768560000
C	-2.8324390000	1.3322420000	-0.1919640000
H	-3.1914660000	2.3563620000	-0.2636200000
C	-1.4547950000	1.1092370000	-0.0825160000
O	1.8562770000	2.7693280000	0.7278130000
H	0.9773640000	2.6812040000	1.1200510000
O	-1.4615270000	-2.4997760000	0.4454400000
H	-2.2651600000	-3.0128910000	0.6024620000
O	-0.6795850000	2.2311300000	0.0330440000
H	4.4109330000	-1.7914590000	0.1255740000
H	-1.2354360000	3.0169280000	-0.0494440000

GS2 Optimization

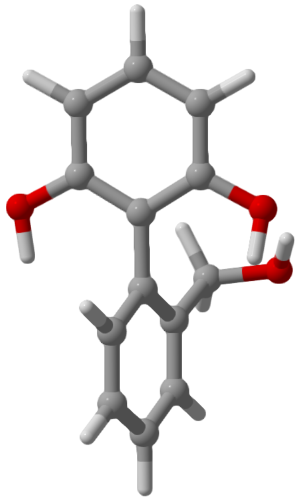

GS2 Cartesian coordinates

C	3.5127760000	-0.7466520000	0.3232390000
C	2.9492550000	0.2548310000	-0.4682910000
H	3.5947390000	0.9205220000	-1.0352740000
C	1.5602270000	0.4273970000	-0.5544590000
C	0.7176490000	-0.4345950000	0.1901190000
C	1.2983430000	-1.4420510000	0.9854620000
H	0.6481470000	-2.0963430000	1.5605000000
C	2.6836510000	-1.6021900000	1.0522460000
H	3.1094970000	-2.3859520000	1.6716710000
C	1.0071790000	1.5432270000	-1.4260480000
H	1.6786850000	1.7033560000	-2.2739610000
H	0.0232150000	1.2653110000	-1.8223730000
C	-0.7741070000	-0.3038340000	0.1514260000
C	-1.4274400000	0.7495160000	0.8165580000
C	-2.8128510000	0.9000360000	0.7745600000
H	-3.2734090000	1.7268200000	1.3035280000
C	-3.5685960000	-0.0316680000	0.0602270000
H	-4.6486520000	0.0738040000	0.0224090000
C	-2.9613020000	-1.0994840000	-0.5985160000
H	-3.5389110000	-1.8296710000	-1.1543920000
C	-1.5703900000	-1.2319550000	-0.5484340000
O	0.9448030000	2.8037820000	-0.7534050000
H	0.2495080000	2.7642690000	-0.0780480000
O	-0.7063470000	1.6992010000	1.5125620000
H	0.1688300000	1.3447830000	1.7334410000
O	-1.0178540000	-2.2842880000	-1.2247520000
H	4.5919400000	-0.8603500000	0.3684900000
H	-0.0547650000	-2.2715800000	-1.1197620000

TS2 Optimization

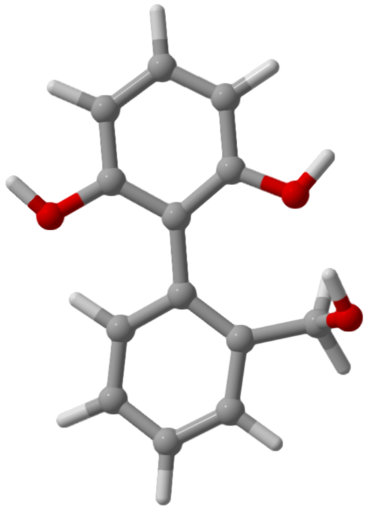

TS2 Cartesian coordinates

C	3.3859310000	-1.4471000000	0.0251140000
C	3.0913000000	-0.0915820000	-0.0242700000
H	3.9119990000	0.6192830000	0.0064660000
C	1.7916850000	0.4378030000	-0.1555640000
C	0.6660580000	-0.4677660000	-0.1214280000
C	1.0254590000	-1.8412980000	-0.1982860000
H	0.2574750000	-2.5802990000	-0.3152300000
C	2.3253820000	-2.3298980000	-0.1279090000
H	2.4886670000	-3.4022480000	-0.1875360000
C	1.8613360000	1.9409740000	-0.4369580000
H	2.8315640000	2.1335760000	-0.8994210000
H	1.1003820000	2.2510320000	-1.1527060000
C	-0.8359590000	-0.1766600000	-0.0454110000
C	-1.4548170000	1.1092060000	-0.0826550000
C	-2.8324680000	1.3321720000	-0.1920500000
H	-3.1915310000	2.3562800000	-0.2637010000
C	-3.7217500000	0.2712590000	-0.1791970000
H	-4.7901660000	0.4323670000	-0.2767580000
C	-3.2035840000	-0.9993550000	0.0074240000
H	-3.8686000000	-1.8563200000	0.0869150000
C	-1.8232380000	-1.2133620000	0.1232330000
O	1.8560810000	2.7693900000	0.7278930000
H	0.9770720000	2.6813330000	1.1199290000
O	-0.6796130000	2.2311200000	0.0329020000
H	-1.2354450000	3.0168990000	-0.0499190000
O	-1.4613870000	-2.4997480000	0.4456240000
H	4.4109120000	-1.7914470000	0.1257070000
H	-2.2649890000	-3.0129130000	0.6026430000

1.3 Calculation of Rotational Barrier for 3aa

Supplementary Table 15. Calculation of rotational barrier for 3aa

Structure	G^a (Ha)	Thermal correction^b	G_{corr} (Ha)	$\Delta G_{corr}^{\ddagger}$ (Ha)
**GS1**	−1075.91891535	0.311904	−1075.60701135	
**TS1**	−1075.86735605	0.312440	−1075.55491605	0.05209530
**GS2**	−1075.91924121	0.311551	−1075.60769021	
**TS2**	−1075.86747666	0.313154	−1075.55432266	0.05268869

^{a}Computed at M06-2X/6-311++G(2d,3p) level of theory. ^{b}Computed at 298 K and B3LYP/6-31+G(d,p) level of theory.

$\Delta G_{corr}^{\ddagger}$ (1) = 32.690 kcal/mol → $k_1 = 7.317 \times 10^{-12}$ s^{-1}
$\Delta G_{corr}^{\ddagger}$ (2) = 33.062 kcal/mol → $k_2 = 3.905 \times 10^{-12}$ s^{-1}

$k_{obs} = k_1 + k_2 = 1.122 \times 10^{-11}$ s^{-1} → $\boldsymbol{\Delta G_{obs}^{\ddagger}}$ **= 32.436 kcal/mol**

GS1 Optimization

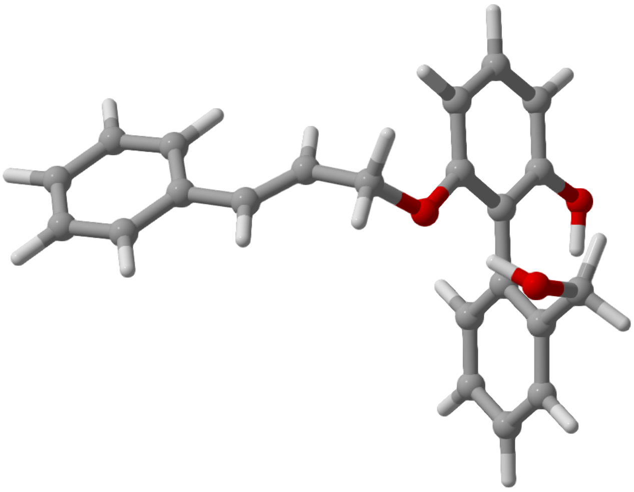

GS1 Cartesian coordinates

C	3.0756250000	-3.2840330000	-1.1780490000
C	3.5122580000	-2.6920420000	0.0074320000
H	4.1067020000	-3.2687120000	0.7114420000
C	3.1997040000	-1.3616460000	0.3204090000
C	2.4164410000	-0.6108260000	-0.5857960000
C	1.9809960000	-1.2179210000	-1.7779320000
H	1.3724870000	-0.6407240000	-2.4690160000
C	2.3054250000	-2.5424870000	-2.0764120000
H	1.9556880000	-2.9902950000	-3.0020430000
C	3.6571120000	-0.7832650000	1.6446170000
H	4.5788040000	-1.2749200000	1.9678530000
H	3.8642540000	0.2903190000	1.5417090000
C	2.0566480000	0.8244620000	-0.3427470000
C	2.7243880000	1.8394190000	-1.0525950000
C	2.3899780000	3.1893500000	-0.8901530000
H	2.9314990000	3.9389420000	-1.4562790000
C	1.3710040000	3.5285590000	-0.0087370000
H	1.1009890000	4.5721280000	0.1232860000
C	0.6843070000	2.5500930000	0.7184540000
H	-0.1029700000	2.8455860000	1.4001250000
C	1.0342680000	1.2065440000	0.5516630000
C	-0.7783100000	0.3905750000	1.9819750000
H	-0.6528030000	1.2610410000	2.6380070000
H	-0.8707520000	-0.4946900000	2.6154620000
C	-1.9757630000	0.5255630000	1.0903150000
H	-1.9786830000	1.3706020000	0.4059970000
C	-3.0039270000	-0.3382900000	1.1250490000
H	-2.9396760000	-1.1757140000	1.8210080000
C	-4.2394410000	-0.3011320000	0.3266940000
C	-4.5298550000	0.7166820000	-0.6028810000

H	-3.8277400000	1.5305810000	-0.7546350000
C	-5.7141520000	0.6972280000	-1.3358960000
H	-5.9177780000	1.4921360000	-2.0477020000
C	-6.6407270000	-0.3373290000	-1.1587700000
H	-7.5634520000	-0.3482440000	-1.7313290000
C	-6.3695370000	-1.3527870000	-0.2395600000
H	-7.0804740000	-2.1606680000	-0.0924020000
C	-5.1819910000	-1.3320600000	0.4938260000
H	-4.9769470000	-2.1265950000	1.2070500000
O	2.7088610000	-1.0114860000	2.6911610000
H	1.8512540000	-0.6835570000	2.3732850000
O	3.7324100000	1.5540610000	-1.9291630000
H	3.8624950000	0.5938650000	-1.9705000000
O	0.4469280000	0.1694790000	1.2445370000
H	3.3348090000	-4.3154530000	-1.3990410000

TS1 Optimization

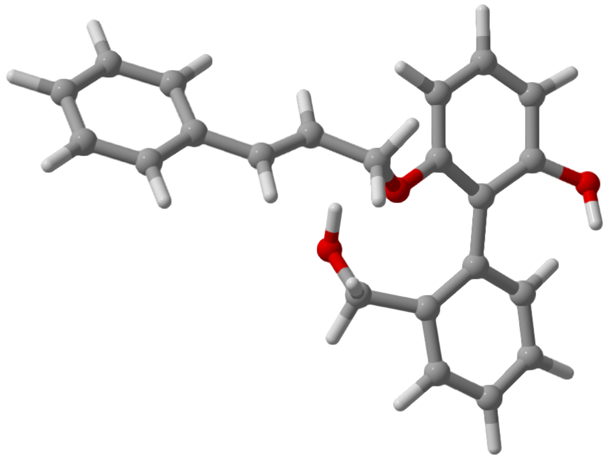

TS1 Cartesian coordinates

C	-4.3362000000	-3.1948490000	-0.1267260000
C	-2.9534000000	-3.0719910000	-0.0800100000
H	-2.3559800000	-3.9793400000	-0.1204110000
C	-2.2673470000	-1.8533830000	0.0881800000
C	-3.0272600000	-0.6280700000	0.0842770000
C	-4.4307720000	-0.8245230000	0.2472920000
H	-5.0487090000	-0.0005920000	0.5637410000
C	-5.0794420000	-2.0492910000	0.1318900000
H	-6.1567690000	-2.0936500000	0.2630980000
C	-0.7894110000	-2.0900500000	0.4288110000
H	-0.7023180000	-3.1445010000	0.7027950000
H	-0.1356810000	-1.9272220000	-0.4313190000
C	-2.5339790000	0.8051300000	0.0078060000
C	-3.4167270000	1.9396740000	0.0407880000
C	-2.9763760000	3.2500550000	0.2595610000
H	-3.7334140000	4.0228170000	0.3324780000
C	-1.6239300000	3.5365790000	0.3140050000
H	-1.2754370000	4.5477240000	0.4989380000
C	-0.7189270000	2.5144290000	0.0447100000
H	0.3402720000	2.7304670000	-0.0150450000
C	-1.1710460000	1.2096250000	-0.1741190000
C	0.8349560000	0.6327560000	-1.4470990000
H	0.5970510000	1.5886170000	-1.9274530000
H	0.8707480000	-0.1410500000	-2.2211580000
C	2.1374840000	0.6834370000	-0.7070570000
H	2.2160110000	1.4171550000	0.0927100000
C	3.1733470000	-0.1192090000	-1.0044700000
H	3.0354360000	-0.8429690000	-1.8088870000
C	4.5044330000	-0.1479250000	-0.3791040000
C	4.8746780000	0.6885030000	0.6920410000
H	4.1625660000	1.4001120000	1.0980930000

C	6.1498020000	0.6144070000	1.2473350000
H	6.4136570000	1.2669740000	2.0746000000
C	7.0888500000	-0.2956350000	0.7471690000
H	8.0819810000	-0.3504070000	1.1830410000
C	6.7380630000	-1.1323120000	-0.3141580000
H	7.4572000000	-1.8435230000	-0.7099730000
C	5.4592970000	-1.0577150000	-0.8686060000
H	5.1921290000	-1.7135660000	-1.6935180000
O	-0.3273510000	-1.3778100000	1.5678560000
H	0.0266220000	-0.5378810000	1.2494460000
O	-4.7756780000	1.8903010000	-0.1697330000
H	-4.9963640000	1.1275540000	-0.7208760000
O	-0.2719560000	0.2599210000	-0.5906020000
H	-4.8056810000	-4.1638630000	-0.2655750000

GS2 Optimization

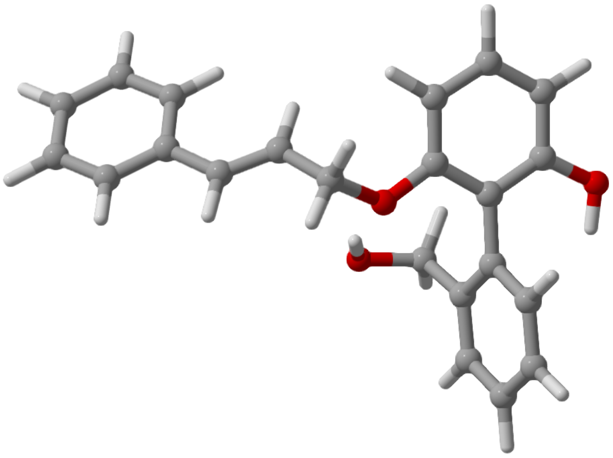

GS2 Cartesian coordinates

C	-3.9883030000	-3.1711060000	-0.2804690000
C	-3.0430610000	-2.8562330000	0.6958740000
H	-2.7036650000	-3.6242190000	1.3861950000
C	-2.5103050000	-1.5642890000	0.8108400000
C	-2.9375610000	-0.5649470000	-0.0924610000
C	-3.8929780000	-0.8928410000	-1.0723910000
H	-4.2155420000	-0.1235800000	-1.7691220000
C	-4.4169600000	-2.1829690000	-1.1696710000
H	-5.1518540000	-2.4129060000	-1.9356960000
C	-1.4542410000	-1.2907710000	1.8636830000
H	-1.5066250000	-0.2448330000	2.1936550000
H	-1.6267710000	-1.9273570000	2.7360990000
C	-2.4343230000	0.8457110000	-0.0306950000
C	-3.2643860000	1.8626860000	0.4753180000
C	-2.8526290000	3.2008840000	0.4995420000
H	-3.5242340000	3.9522390000	0.8996120000
C	-1.5932180000	3.5266090000	0.0116940000
H	-1.2624120000	4.5608520000	0.0278280000
C	-0.7336830000	2.5439870000	-0.4918540000
H	0.2485070000	2.8250380000	-0.8501980000
C	-1.1583420000	1.2114940000	-0.5099600000
C	0.7781870000	0.4392930000	-1.7888910000
H	0.5809720000	1.3316390000	-2.3948720000
H	0.8491980000	-0.4195800000	-2.4619360000
C	2.0319770000	0.5716930000	-0.9771730000
H	2.0583170000	1.3802250000	-0.2500920000
C	3.0810390000	-0.2547220000	-1.1258270000
H	2.9944010000	-1.0541610000	-1.8629510000
C	4.3678600000	-0.2170640000	-0.4140880000
C	4.6540870000	0.6975000000	0.6181280000
H	3.9042340000	1.4179300000	0.9294690000

C	5.8910130000	0.6864700000	1.2582130000
H	6.0895690000	1.3982560000	2.0543650000
C	6.8744220000	-0.2373130000	0.8844350000
H	7.8373390000	-0.2431170000	1.3865730000
C	6.6060080000	-1.1524920000	-0.1353470000
H	7.3594980000	-1.8762270000	-0.4323100000
C	5.3653040000	-1.1411160000	-0.7747390000
H	5.1617920000	-1.8585430000	-1.5658550000
O	-0.1366930000	-1.6102940000	1.4112320000
H	0.0200140000	-1.1108180000	0.5933120000
O	-4.5098470000	1.5908860000	0.9666190000
H	-4.6884280000	0.6410590000	0.8864090000
O	-0.3853330000	0.1699110000	-0.9715680000
H	-4.3887660000	-4.1787020000	-0.3457200000

TS2 Optimization

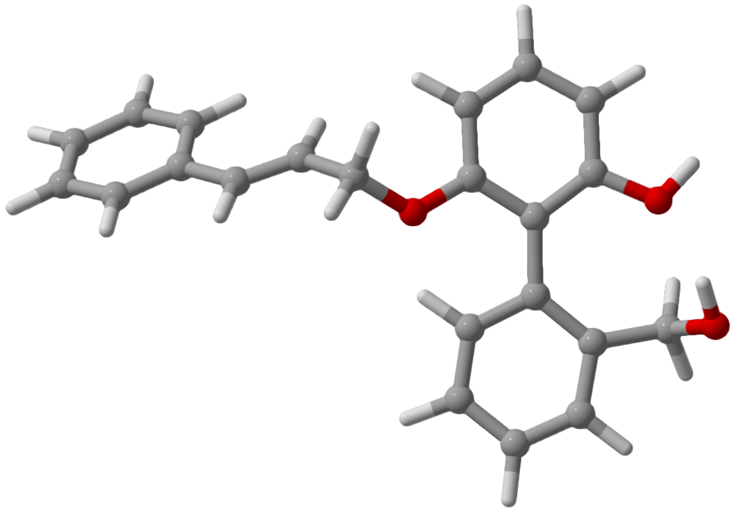

TS2 Cartesian coordinates

C	-2.9349440000	3.6087520000	-0.0361300000
C	-3.9769970000	2.7183910000	-0.2548770000
H	-4.9814250000	3.1127520000	-0.3808090000
C	-3.8188220000	1.3232330000	-0.3768440000
C	-2.5165320000	0.7465550000	-0.1345920000
C	-1.4673550000	1.7043930000	-0.0597700000
H	-0.4502080000	1.3645960000	-0.0356340000
C	-1.6498960000	3.0816350000	-0.0083850000
H	-0.7780060000	3.7265550000	0.0570650000
C	-5.1099320000	0.6892390000	-0.9005660000
H	-5.6245420000	1.4544890000	-1.4858380000
H	-4.9043860000	-0.1422180000	-1.5746400000
C	-2.0998680000	-0.7189510000	0.0002550000
C	-2.9441590000	-1.8511930000	-0.1792730000
C	-2.4925860000	-3.1725560000	-0.2913740000
H	-3.2165860000	-3.9587450000	-0.4933460000
C	-1.1528660000	-3.4677550000	-0.1230250000
H	-0.7842640000	-4.4820200000	-0.2375340000
C	-0.2961230000	-2.4402510000	0.2484160000
H	0.7414390000	-2.6670230000	0.4490730000
C	-0.7598510000	-1.1232070000	0.3824440000
C	1.2718710000	-0.5692590000	1.6454310000
H	1.0994170000	-1.5280470000	2.1493110000
H	1.3766720000	0.2008190000	2.4150270000
C	2.4922500000	-0.5957630000	0.7730280000
H	2.4739900000	-1.2799960000	-0.0723340000
C	3.5654360000	0.1784170000	1.0052800000
H	3.5226580000	0.8599830000	1.8559100000
C	4.8251340000	0.2274080000	0.2463880000
C	5.0804550000	-0.5743690000	-0.8833660000

H	4.3293600000	-1.2757090000	-1.2332990000
C	6.2907100000	-0.4809330000	-1.5665430000
H	6.4657220000	-1.1077750000	-2.4362740000
C	7.2791220000	0.4136700000	-1.1389020000
H	8.2216600000	0.4830050000	-1.6738100000
C	7.0429630000	1.2148430000	-0.0200140000
H	7.8014520000	1.9131090000	0.3218310000
C	5.8290200000	1.1208740000	0.6626320000
H	5.6517050000	1.7489040000	1.5322180000
O	-6.0534630000	0.3259970000	0.1100280000
H	-5.6490660000	-0.3947680000	0.6112840000
O	-4.3032610000	-1.6815520000	-0.2043630000
H	-4.7302370000	-2.5309740000	-0.3759040000
O	0.0833750000	-0.1908860000	0.9218280000
H	-3.1207280000	4.6755480000	0.0464570000

1.4 Calculation of Rotational Barrier for 3ka

Supplementary Table 16. Calculation of rotational barrier for 3ka

Structure	G[a] (Ha)	Thermal correction[b]	G_{corr} (Ha)	$\Delta G_{corr}^{\ddagger}$ (Ha)
**GS1**	−1115.22956282	0.337667	−1114.89189582	
**TS1**	−1115.16030517	0.338235	−1114.82207017	0.06982565
**GS2**	−1115.22961897	0.337049	−1114.89256997	
**TS2**	−1115.15558585	0.338794	−1114.81679185	0.07510397

[a]Computed at M06-2X/6-311++G(2d,3p) level of theory. [b]Computed at 298 K and B3LYP/6-31+G(d,p) level of theory.

$\Delta G_{corr}^{\ddagger}$ (1) = 43.813 kcal/mol → $k_1 = 5.282 \times 10^{-20}$ s^{-1}
$\Delta G_{corr}^{\ddagger}$ (2) = 46.608 kcal/mol → $k_2 = 4.752 \times 10^{-22}$ s^{-1}

$k_{obs} = k_1 + k_2 = 5.329 \times 10^{-20}$ s^{-1} → **$\Delta G_{obs}^{\ddagger}$ = 43.808 kcal/mol**

GS1 Optimization

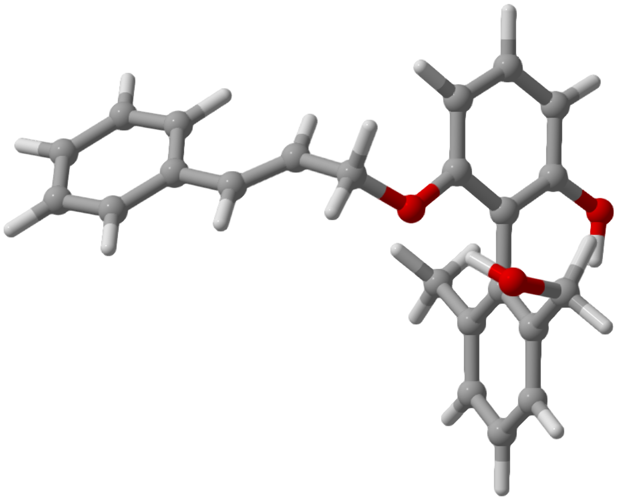

GS1 Cartesian coordinates

C	3.1449850000	-3.3160580000	-0.7217190000
C	3.5894710000	-2.5917580000	0.3818090000
H	4.2210850000	-3.0659670000	1.1281780000
C	3.2241620000	-1.2499030000	0.5518840000
C	2.3937760000	-0.6273430000	-0.4080780000
C	1.9278810000	-1.3643080000	-1.5248830000
C	2.3169520000	-2.7032710000	-1.6629820000
H	1.9605380000	-3.2709810000	-2.5185810000
C	3.6645150000	-0.5190840000	1.8048610000
H	4.5966180000	-0.9498070000	2.1805790000
H	3.8435480000	0.5428600000	1.5915570000
C	2.0115770000	0.8185900000	-0.2752170000
C	2.7024620000	1.7925170000	-1.0175450000
C	2.3548440000	3.1470480000	-0.9570830000
H	2.9140090000	3.8638190000	-1.5480870000
C	1.3014070000	3.5334750000	-0.1370570000
H	1.0207960000	4.5811350000	-0.0818740000
C	0.5963630000	2.5984700000	0.6287190000
H	-0.2129450000	2.9317050000	1.2657450000
C	0.9604240000	1.2496170000	0.5604590000
C	-0.8571880000	0.5200770000	2.0304530000
H	-0.7269530000	1.4279980000	2.6327620000
H	-0.9486360000	-0.3259130000	2.7156140000
C	-2.0592940000	0.6046320000	1.1383980000
H	-2.0711420000	1.4150750000	0.4134660000
C	-3.0818510000	-0.2629890000	1.2192370000
H	-3.0086050000	-1.0660920000	1.9536800000
C	-4.3206870000	-0.2723510000	0.4251810000
C	-4.6325990000	0.7112860000	-0.5337700000
H	-3.9470530000	1.5341520000	-0.7109440000

C	-5.8183630000	0.6477040000	-1.2619330000
H	-6.0390120000	1.4170320000	-1.9964010000
C	-6.7251940000	-0.3977490000	-1.0500660000
H	-7.6492930000	-0.4429600000	-1.6187000000
C	-6.4331520000	-1.3790220000	-0.1005960000
H	-7.1290310000	-2.1944850000	0.0740730000
C	-5.2441950000	-1.3140890000	0.6278860000
H	-5.0228700000	-2.0821510000	1.3648370000
O	2.7170330000	-0.6602910000	2.8690410000
H	1.8454940000	-0.4215590000	2.5128230000
O	3.7428370000	1.4536600000	-1.8365520000
H	3.9092580000	0.5004460000	-1.7636180000
O	0.3641150000	0.2527600000	1.3027710000
H	3.4367360000	-4.3548410000	-0.8480920000
C	1.0057050000	-0.7392620000	-2.5485510000
H	1.4716900000	0.1121030000	-3.0567210000
H	0.7228960000	-1.4707810000	-3.3103170000
H	0.0889920000	-0.3672000000	-2.0785000000

TS1 Optimization

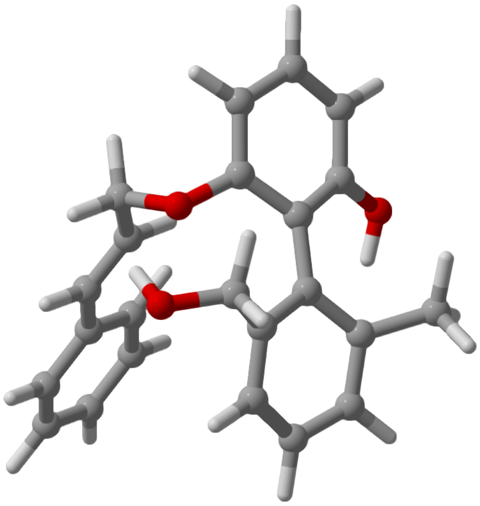

TS1 Cartesian coordinates

C	-0.2199480000	3.4660170000	0.3049310000
C	-0.5727680000	2.6585200000	1.3836840000
H	-0.2958500000	2.9364840000	2.3970410000
C	-1.3492960000	1.5090680000	1.2105600000
C	-1.6710650000	1.0543430000	-0.1133470000
C	-1.6673940000	2.0973470000	-1.1108010000
C	-0.8685290000	3.2317210000	-0.9046530000
H	-0.8313640000	3.9828760000	-1.6907530000
C	-2.0678650000	1.0193820000	2.4698280000
H	-2.6834000000	1.8632830000	2.8037510000
H	-2.7535370000	0.1968130000	2.2539510000
C	-2.0018810000	-0.3772830000	-0.4032390000
C	-2.3029720000	-0.8924250000	-1.7053900000
C	-2.8833480000	-2.1518590000	-1.9074550000
H	-3.1626210000	-2.4208240000	-2.9205200000
C	-3.0179610000	-3.0344760000	-0.8468410000
H	-3.4876680000	-4.0028320000	-0.9874580000
C	-2.4372510000	-2.6953900000	0.3759770000
H	-2.3822090000	-3.4120530000	1.1884620000
C	-1.8617770000	-1.4384610000	0.5557370000
C	0.1483070000	-2.0809120000	1.6993570000
H	-0.1247360000	-3.1276450000	1.5224670000
H	0.5233610000	-1.9939340000	2.7232000000
C	1.1773430000	-1.6196090000	0.7140360000
H	0.9168200000	-1.7265530000	-0.3367570000
C	2.3661550000	-1.1146790000	1.0829380000
H	2.5641440000	-1.0098050000	2.1504970000
C	3.4624210000	-0.6654500000	0.2104920000
C	3.4533970000	-0.8305170000	-1.1883390000
H	2.6151480000	-1.3230880000	-1.6712210000

C	4.5155950000	-0.3775480000	-1.9674590000
H	4.4886260000	-0.5165810000	-3.0445080000
C	5.6162380000	0.2485870000	-1.3703000000
H	6.4433490000	0.5986390000	-1.9808690000
C	5.6439800000	0.4142490000	0.0159760000
H	6.4932460000	0.8962680000	0.4916120000
C	4.5790080000	-0.0406780000	0.7952740000
H	4.6063220000	0.0932660000	1.8738220000
O	-1.2306780000	0.7223810000	3.5804890000
H	-0.8187750000	-0.1245750000	3.3651690000
O	-1.9767330000	-0.2504120000	-2.8655680000
H	-1.3891950000	0.4933180000	-2.6737500000
O	-1.0665630000	-1.2728120000	1.6835320000
H	0.4199020000	4.3332190000	0.4372890000
C	-2.7045030000	2.2408620000	-2.2293470000
H	-3.4843120000	1.4822940000	-2.1913650000
H	-3.1879470000	3.2125480000	-2.0821740000
H	-2.2928330000	2.2507660000	-3.2452340000

GS2 Optimization

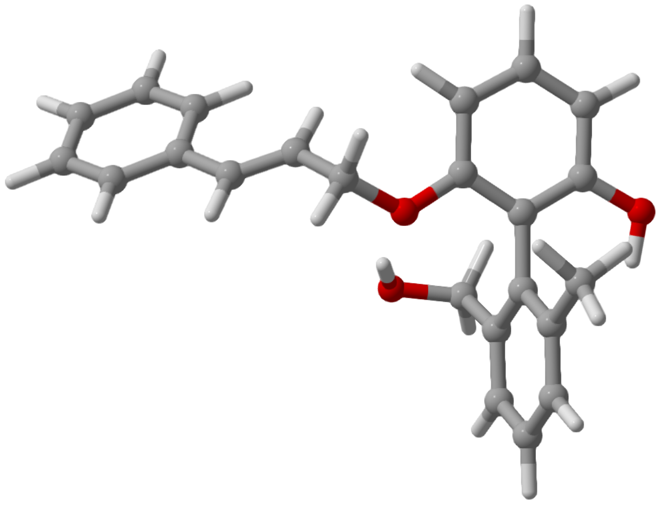

GS2 Cartesian coordinates

C	3.7394950000	-3.2125090000	0.0337350000
C	2.8049050000	-2.8598580000	-0.9366070000
H	2.4541450000	-3.5990440000	-1.6517560000
C	2.2963870000	-1.5557190000	-1.0009280000
C	2.7380720000	-0.5898640000	-0.0685040000
C	3.6806800000	-0.9469950000	0.9276370000
C	4.1683580000	-2.2599570000	0.9592270000
H	4.8908880000	-2.5368040000	1.7226320000
C	1.2282790000	-1.2330200000	-2.0275700000
H	1.3034830000	-0.1855850000	-2.3472970000
H	1.3583840000	-1.8647310000	-2.9106740000
C	2.2357830000	0.8235010000	-0.1260130000
C	3.0289170000	1.8236770000	-0.7148400000
C	2.6224740000	3.1628940000	-0.7437990000
H	3.2668860000	3.9016170000	-1.2072040000
C	1.3987430000	3.5051490000	-0.1809720000
H	1.0700000000	4.5400060000	-0.2000290000
C	0.5698830000	2.5378060000	0.3980050000
H	-0.3881150000	2.8313010000	0.8080430000
C	0.9920740000	1.2040820000	0.4197960000
C	-0.9036920000	0.4577190000	1.7727780000
H	-0.6987280000	1.3610680000	2.3600690000
H	-0.9652160000	-0.3884930000	2.4623960000
C	-2.1690650000	0.5753070000	0.9766610000
H	-2.2028510000	1.3652970000	0.2298400000
C	-3.2197740000	-0.2412760000	1.1627690000
H	-3.1272100000	-1.0202190000	1.9208540000
C	-4.5167540000	-0.2147430000	0.4691090000
C	-4.8018000000	0.6526140000	-0.6033560000

H	-4.0423450000	1.3394940000	-0.9633940000
C	-6.0489210000	0.6343910000	-1.2231990000
H	-6.2462600000	1.3087780000	-2.0515530000
C	-7.0436290000	-0.2499390000	-0.7888430000
H	-8.0143710000	-0.2617430000	-1.2755850000
C	-6.7760500000	-1.1191480000	0.2706320000
H	-7.5381230000	-1.8126010000	0.6143090000
C	-5.5250760000	-1.1009220000	0.8895490000
H	-5.3223170000	-1.7825490000	1.7119190000
O	-0.0886730000	-1.5162180000	-1.5459880000
H	-0.1994740000	-1.0588340000	-0.6967800000
O	4.2393020000	1.5290610000	-1.2776880000
H	4.3882250000	0.5713380000	-1.2316590000
O	0.2478300000	0.1718860000	0.9453720000
H	4.1313950000	-4.2249270000	0.0738080000
C	4.1484680000	0.0543390000	1.9610980000
H	3.3033870000	0.4747820000	2.5168360000
H	4.8249230000	-0.4188500000	2.6780260000
H	4.6790680000	0.8976940000	1.5054380000

TS2 Optimization

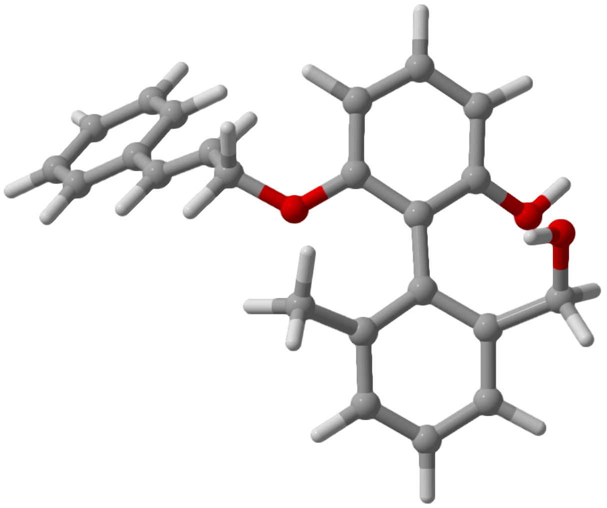

TS2 Cartesian coordinates

C	3.5856950000	-3.0520230000	-1.2664340000
C	4.2707760000	-1.8573680000	-1.4489920000
H	5.1790690000	-1.8431450000	-2.0480640000
C	3.8695330000	-0.6695620000	-0.8232000000
C	2.6343830000	-0.6304240000	-0.0777920000
C	2.2262730000	-1.9229290000	0.4225810000
C	2.6405440000	-3.0812530000	-0.2457770000
H	2.2600960000	-4.0371620000	0.1075390000
C	4.9638720000	0.4041620000	-0.8421280000
H	5.9171350000	-0.1423520000	-0.8044100000
H	4.9615720000	0.9888370000	-1.7612930000
C	1.8845060000	0.6456790000	0.1716920000
C	2.2188570000	1.8743230000	-0.4765490000
C	1.7496190000	3.1210560000	-0.0409430000
H	2.1368990000	4.0228800000	-0.5089240000
C	0.7710580000	3.1884890000	0.9365590000
H	0.4107380000	4.1474880000	1.2956760000
C	0.1788480000	2.0059580000	1.3735050000
H	-0.6918790000	2.0545270000	2.0129790000
C	0.6599450000	0.7686190000	0.9232510000
C	-1.3048690000	-0.3598140000	1.8378440000
H	-1.1953110000	0.2480070000	2.7450400000
H	-1.4403730000	-1.3980130000	2.1543800000
C	-2.4709710000	0.0788570000	1.0002510000
H	-2.4068270000	1.0721530000	0.5634220000
C	-3.5458240000	-0.6995310000	0.7936410000
H	-3.5469970000	-1.6936010000	1.2431120000
C	-4.7572050000	-0.3772740000	0.0225720000
C	-4.9501220000	0.8519530000	-0.6378910000

H	-4.1838560000	1.6194570000	-0.5916100000
C	-6.1162950000	1.1003570000	-1.3580650000
H	-6.2429670000	2.0547830000	-1.8611820000
C	-7.1220350000	0.1295310000	-1.4365860000
H	-8.0299700000	0.3273750000	-1.9986500000
C	-6.9473790000	-1.0940280000	-0.7869730000
H	-7.7195230000	-1.8561260000	-0.8405480000
C	-5.7774870000	-1.3417560000	-0.0668080000
H	-5.6478430000	-2.2976010000	0.4348770000
O	4.9302850000	1.3510700000	0.2201820000
H	4.9168290000	0.8719780000	1.0595910000
O	2.9502410000	1.8404980000	-1.6265050000
H	3.1338670000	2.7472960000	-1.9053200000
O	-0.0683940000	-0.3662450000	1.1043130000
H	3.8758130000	-3.9560090000	-1.7932530000
C	1.5851250000	-2.1942710000	1.7772440000
H	0.5561580000	-2.5555150000	1.7096040000
H	2.1768550000	-2.9818370000	2.2589790000
H	1.6023810000	-1.3285360000	2.4381080000

1.5 Calculation of Rotational Barrier for 3na

Supplementary Table 17. Calculation of rotational barrier for 3na

Structure	G^a (Ha)	Thermal correction^b	G_{corr} (Ha)	$\Delta G_{corr}^{\ddagger}$ (Ha)
**GS1**	−1000.70030147	0.305954	−1000.39434747	
**TS1**	−1000.65184406	0.309708	−1000.34213606	0.05221141
**GS2**	−1000.70091316	0.306247	−1000.39466616	
**TS2**	−1000.64718765	0.308723	−1000.33846465	0.05588282

[a]Computed at M06-2X/6-311++G(2d,3p) level of theory. [b]Computed at 298 K and B3LYP/6-31+G(d,p) level of theory.

$\Delta G_{corr}^{\ddagger}$ (1) = 32.763 kcal/mol → $k_1 = 6.471 \times 10^{-12}$ s^{-1}
$\Delta G_{corr}^{\ddagger}$ (2) = 35.067 kcal/mol → $k_2 = 1.332 \times 10^{-13}$ s^{-1}

$k_{obs} = k_1 + k_2 = 6.605 \times 10^{-12}$ s^{-1} → **$\Delta G_{obs}^{\ddagger}$ = 32.751 kcal/mol**

GS1 Optimization

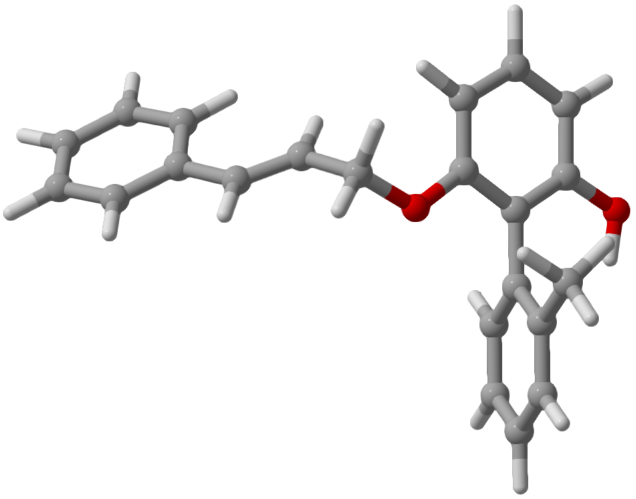

GS1 Cartesian coordinates

C	3.5066950000	-3.3360960000	-0.7236760000
C	3.7789160000	-2.6712170000	0.4745830000
H	4.3157920000	-3.1907780000	1.2644580000
C	3.3802000000	-1.3442860000	0.6862380000
C	2.6812020000	-0.6760640000	-0.3460710000
C	2.4143600000	-1.3521270000	-1.5482660000
H	1.8685630000	-0.8322170000	-2.3312790000
C	2.8215170000	-2.6742980000	-1.7428420000
H	2.6007570000	-3.1793180000	-2.6786350000
C	3.6851200000	-0.6565660000	1.9959880000
H	4.1960570000	0.2995910000	1.8403610000
H	2.7625970000	-0.4404390000	2.5461420000
C	2.2449770000	0.7486770000	-0.1985530000
C	2.9789620000	1.7750690000	-0.8165210000
C	2.5979780000	3.1186300000	-0.7062480000
H	3.1936340000	3.8797460000	-1.1977470000
C	1.4635150000	3.4347380000	0.0308490000
H	1.1547730000	4.4722180000	0.1209080000
C	0.7026280000	2.4417150000	0.6588440000
H	-0.1810650000	2.7222070000	1.2177280000
C	1.0981800000	1.1032450000	0.5450180000
C	-0.7625930000	0.2752000000	1.8782810000
H	-0.6258180000	1.1428570000	2.5376720000
H	-0.8606670000	-0.6116190000	2.5096860000
C	-1.9723910000	0.4266650000	1.0028300000
H	-1.9512910000	1.2440930000	0.2857790000
C	-3.0355790000	-0.3898610000	1.0855230000
H	-2.9938520000	-1.2052740000	1.8089680000
C	-4.2830360000	-0.3271840000	0.3070090000
C	-4.5511290000	0.6749480000	-0.6460610000

H	-3.8212640000	1.4566990000	-0.8320170000
C	-5.7478930000	0.6810970000	-1.3589210000
H	-5.9330550000	1.4634240000	-2.0895320000
C	-6.7104870000	-0.3111410000	-1.1375640000
H	-7.6428330000	-0.3020930000	-1.6943960000
C	-6.4621380000	-1.3102420000	-0.1942630000
H	-7.2007970000	-2.0856290000	-0.0124510000
C	-5.2618810000	-1.3153300000	0.5185110000
H	-5.0750510000	-2.0973310000	1.2504200000
O	4.1050860000	1.5078130000	-1.5447840000
H	4.2497340000	0.5489260000	-1.5690130000
O	0.4437920000	0.0508910000	1.1278400000
H	4.3197180000	-1.2837170000	2.6283670000
H	3.8303180000	-4.3644180000	-0.8579960000

TS1 Optimization

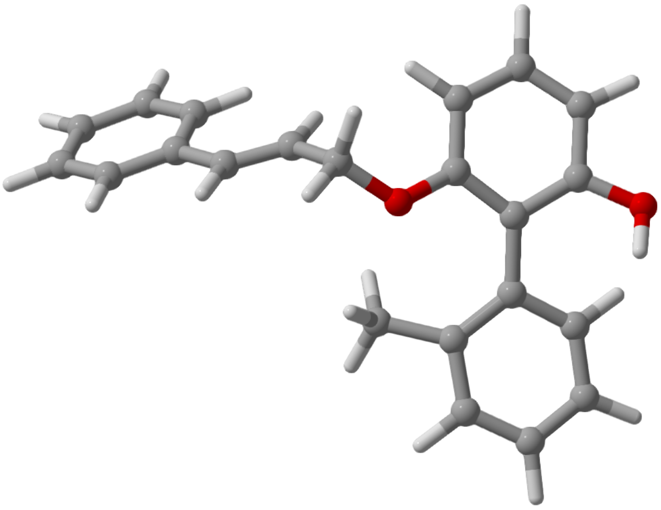

TS1 Cartesian coordinates

C	-4.0158900000	3.3735380000	-0.2815710000
C	-2.7006940000	3.1757320000	0.1189800000
H	-2.1064960000	4.0430010000	0.3955590000
C	-2.0524250000	1.9239750000	0.1284110000
C	-2.8289640000	0.7456900000	-0.1550170000
C	-4.0998890000	1.0185460000	-0.7452210000
H	-4.6252800000	0.2282710000	-1.2585990000
C	-4.6929600000	2.2748610000	-0.8009320000
H	-5.6697770000	2.3816830000	-1.2639400000
C	-0.5448040000	2.0513390000	0.2798740000
H	-0.2006940000	1.9261410000	1.3084930000
H	-0.0025550000	1.3373280000	-0.3381670000
C	-2.4420200000	-0.7126280000	-0.0085440000
C	-3.3630170000	-1.7761670000	-0.2896350000
C	-2.9612440000	-3.0998990000	-0.5056800000
H	-3.7239110000	-3.8176650000	-0.7861330000
C	-1.6417190000	-3.4636500000	-0.3056010000
H	-1.3133360000	-4.4819760000	-0.4894700000
C	-0.7578890000	-2.5292240000	0.2311950000
H	0.2376210000	-2.8440240000	0.5118970000
C	-1.1774800000	-1.2128120000	0.4612920000
C	0.8175860000	-0.7830340000	1.7990140000
H	0.6952230000	-1.7864130000	2.2275530000
H	0.9753060000	-0.0857860000	2.6264460000
C	1.9739140000	-0.7191130000	0.8423310000
H	1.8836950000	-1.3041220000	-0.0699950000
C	3.0779160000	0.0068480000	1.0844630000
H	3.1103950000	0.5890840000	2.0065140000
C	4.2829800000	0.1225150000	0.2476050000
C	4.4498500000	-0.5673280000	-0.9692640000

H	3.6690080000	-1.2294240000	-1.3303560000
C	5.6106440000	-0.4138730000	-1.7237760000
H	5.7175880000	-0.9549720000	-2.6596050000
C	6.6369060000	0.4301200000	-1.2826250000
H	7.5408060000	0.5461220000	-1.8732560000
C	6.4888160000	1.1195510000	-0.0774920000
H	7.2776930000	1.7770850000	0.2762390000
C	5.3244120000	0.9654450000	0.6768460000
H	5.2157590000	1.5062980000	1.6137510000
O	-4.7291880000	-1.6353100000	-0.3487230000
H	-4.9996240000	-0.8294240000	0.1122890000
O	-0.4294730000	-0.3649600000	1.2182370000
H	-0.2559560000	3.0543360000	-0.0489050000
H	-4.4603600000	4.3640160000	-0.2643540000

GS2 Optimization

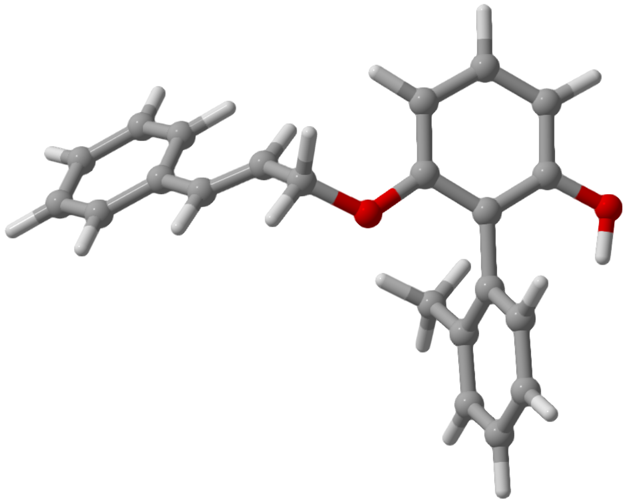

GS2 Cartesian coordinates

C	3.8217230000	-3.3031980000	0.0039550000
C	2.7792190000	-2.9307700000	-0.8481080000
H	2.3409020000	-3.6717300000	-1.5121160000
C	2.2818210000	-1.6204310000	-0.8744060000
C	2.8572010000	-0.6626370000	-0.0067360000
C	3.9077400000	-1.0461050000	0.8443480000
H	4.3367690000	-0.3044340000	1.5132190000
C	4.3919560000	-2.3564350000	0.8553310000
H	5.2020070000	-2.6315330000	1.5244360000
C	1.1474160000	-1.2563540000	-1.8023310000
H	0.2335690000	-1.0485950000	-1.2347550000
H	1.3755290000	-0.3575860000	-2.3848710000
C	2.3948080000	0.7611960000	0.0071420000
C	3.1744940000	1.7592620000	-0.6018370000
C	2.7833730000	3.1044490000	-0.5918920000
H	3.4135950000	3.8420460000	-1.0760950000
C	1.5946450000	3.4522110000	0.0373440000
H	1.2794630000	4.4915630000	0.0510360000
C	0.7875780000	2.4883820000	0.6530500000
H	-0.1350830000	2.7928920000	1.1304700000
C	1.1917760000	1.1478300000	0.6378350000
C	-0.7173130000	0.3930490000	1.9466960000
H	-0.5755640000	1.2904140000	2.5633520000
H	-0.8276070000	-0.4612400000	2.6197670000
C	-1.9201010000	0.5126200000	1.0562940000
H	-1.8930690000	1.3025910000	0.3090810000
C	-2.9847210000	-0.3000610000	1.1598190000
H	-2.9503860000	-1.0860850000	1.9155030000
C	-4.2248020000	-0.2678050000	0.3677280000
C	-4.4783900000	0.6887580000	-0.6349090000

H	-3.7423670000	1.4567250000	-0.8515820000
C	-5.6686450000	0.6670630000	-1.3582810000
H	-5.8426830000	1.4142730000	-2.1273460000
C	-6.6388910000	-0.3082600000	-1.0983980000
H	-7.5661150000	-0.3209810000	-1.6636190000
C	-6.4048470000	-1.2622680000	-0.1060430000
H	-7.1496680000	-2.0239350000	0.1060070000
C	-5.2110120000	-1.2396790000	0.6171060000
H	-5.0353620000	-1.9864940000	1.3875120000
O	4.3469340000	1.4604700000	-1.2388550000
H	4.5188310000	0.5089060000	-1.1623610000
O	0.4912160000	0.1228700000	1.2152170000
H	0.9360290000	-2.0716760000	-2.4998250000
H	4.1858480000	-4.3267390000	-0.0009440000

TS2 Optimization

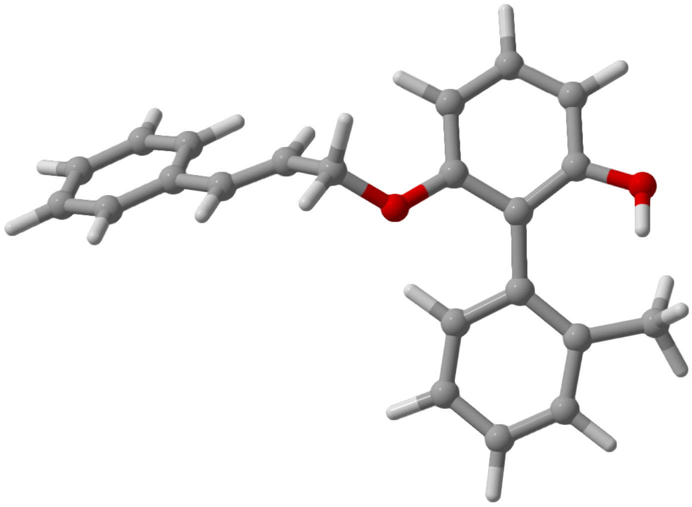

TS2 Cartesian coordinates

C	-2.9515260000	3.6858540000	-0.0831410000
C	-4.0589230000	2.8649950000	-0.2463990000
H	-5.0301670000	3.3320160000	-0.3845270000
C	-4.0040870000	1.4569300000	-0.2983710000
C	-2.7409400000	0.7984590000	-0.0625370000
C	-1.6268970000	1.6824070000	-0.0300840000
H	-0.6368790000	1.2687240000	0.0030130000
C	-1.7078050000	3.0693790000	-0.0323350000
H	-0.7915030000	3.6516500000	0.0047520000
C	-5.3445920000	0.8670470000	-0.7321570000
H	-5.2495380000	0.0917790000	-1.4912760000
H	-5.9575770000	0.4761150000	0.0943880000
C	-2.4104100000	-0.6910140000	0.0511140000
C	-3.2993410000	-1.7818090000	-0.1840540000
C	-2.8756310000	-3.0909370000	-0.4538840000
H	-3.6384350000	-3.8230210000	-0.6944450000
C	-1.5393010000	-3.4211030000	-0.3489660000
H	-1.1936400000	-4.4259620000	-0.5709200000
C	-0.6501010000	-2.4617530000	0.1318430000
H	0.3776350000	-2.7413500000	0.3172650000
C	-1.0826100000	-1.1589360000	0.4022670000
C	0.9654030000	-0.7590090000	1.6920490000
H	0.8006090000	-1.7660610000	2.0948050000
H	1.1052180000	-0.0780450000	2.5364150000
C	2.1565130000	-0.7009640000	0.7805620000
H	2.0980360000	-1.2848140000	-0.1352810000
C	3.2494570000	0.0283550000	1.0607530000
H	3.2472930000	0.6090780000	1.9842470000
C	4.4832240000	0.1475290000	0.2674830000
C	4.6874240000	-0.5259260000	-0.9528710000

H	3.9140960000	-1.1752460000	-1.3513520000
C	5.8750180000	-0.3709650000	-1.6641400000
H	6.0103690000	-0.8990300000	-2.6037430000
C	6.8914650000	0.4584960000	-1.1752820000
H	7.8162750000	0.5758700000	-1.7323290000
C	6.7061150000	1.1322940000	0.0335430000
H	7.4867800000	1.7788450000	0.4238700000
C	5.5148030000	0.9769040000	0.7443400000
H	5.3772370000	1.5056420000	1.6843490000
O	-4.6613590000	-1.6888900000	-0.1417930000
H	-4.9312670000	-0.8879010000	0.3173280000
O	-0.2412240000	-0.2959660000	1.0573290000
H	-5.9473360000	1.6673090000	-1.1666970000
H	-3.0600960000	4.7658620000	-0.0525500000

2 Computational Studies to Investigate the Origin of Enantioselectivity

2.1 General Comments

All calculations were carried out using Gaussian 16[1] and all visualization of input and output files was accomplished using GaussView 6.0.[2] Based on the previously reported X-ray crystallographic structure (CCDC 701796),[8] initial guess structure for π-allylpalldium complex which is coordinated to **L4** and THF was generated. Because of the bulky chiral ligand, the two-layer quantum-mechanical (QM)/semiempirical (SE) ONIOM model[9-11] was applied to this palladium complexes. The QM layer was treated with B3LYP[3-5]/6-31G(d) (C, H, O, N, P)/LANL2DZ (Pd) and the SE layer with PM6.[12] The vibrational frequencies were computed at the same level of theory as for the geometry optimizations to evaluate the zero-point vibrational energy (ZPVE) and thermal corrections at 298 K. All optimized species were verified as either minima or transition structures by the presence of zero or a single imaginary vibrational frequency. The single-point energies of these optimized structures were calculated with M06-2X[6]/6-311++G(2d,3p) (C, H, O, N, P)/SDD (Pd) for the QM layer and PM6[12] for the SE layer with the inclusion of solvation energy corrections using a self-consistent reaction field based on SMD implicit solvent model[13] with tetrahydrofuran as a solvent. The 3D diagrams of computed palladium complex and transition sates were generated using CYLView.[7]

2.2 Computed Structures, Energy and Imaginary Vibrational Frequency of Calculated Structures

Supplementary Table 18. Computed energies and imaginary vibrational Frequencies

Structure	G^a (Ha)	Thermal correction^b	G_{corr} (Ha)	Imaginary frequency^b
[**1a**−H]	−727.75734206	0.171027	−727.58631506	0
AllylPd(**L4**)(THF)	−1101.30786467	0.708428	−1100.59943667	0
**TS1**	−1829.07893533	0.904508	−1828.17442733	1 (−169.9041)
**TS2**	−1829.07162905	0.901226	−1828.17040305	1 (−227.9223)
**TS3**	−1829.06790908	0.902643	−1828.16526608	1 (−233.9887)
**INT1**	−1829.11132933	0.904945	−1828.20638433	0

^{a}Computed using the QM/SE ONIOM model (M06-2X/6-311++G(2d,3p) (C, H, O, N, P)/SDD (Pd):PM6) level of theory with the inclusion of solvation energy corrections (SMD, tetrahydrofuran). ^{b}Computed at 298 K using the QM/SE ONIOM model (B3LYP/6-31G(d) (C, H, O, N, P)/LANL2DZ (Pd):PM6) level of theory.

Based on the energy difference between **TS1** and **TS2** (2.5 kcal/mol), the expected enantioselectivity at −20 °C is 99% ee.

[**1a**−H] Optimization

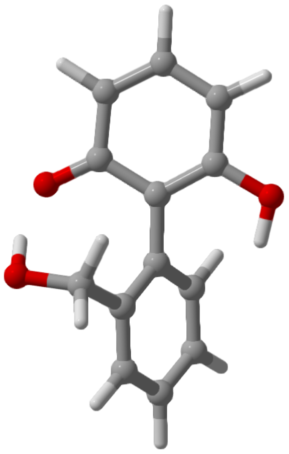

[**1a**−H] Cartesian coordinates

C	1.6091270000	-1.2377780000	0.4588470000
C	0.7963920000	-0.2254070000	-0.0836450000
C	1.3968390000	0.9668940000	-0.6556960000
C	2.8333750000	1.0124900000	-0.6317290000
C	3.5934310000	-0.0077090000	-0.0853530000
C	3.0021850000	-1.1479910000	0.4771710000
H	3.2972580000	1.8962970000	-1.0634050000
H	4.6812420000	0.0769760000	-0.0828770000
H	3.5835220000	-1.9479410000	0.9251880000
C	-0.6780610000	-0.4199980000	-0.1443090000
C	-1.6054580000	0.4535580000	0.4755940000
C	-1.1899170000	-1.5373850000	-0.8381710000
C	-2.9760500000	0.1976100000	0.3550810000
C	-2.5584240000	-1.7928090000	-0.9285280000
H	-0.4830020000	-2.2011440000	-1.3306810000
C	-3.4635040000	-0.9140590000	-0.3326100000
H	-3.6744170000	0.8905340000	0.8221500000
H	-2.9132670000	-2.6650680000	-1.4746690000
H	-4.5351560000	-1.0915680000	-0.4014530000
O	0.7076870000	1.9130630000	-1.1763490000
O	1.0624090000	-2.3783140000	1.0274170000
H	0.0964730000	-2.2783640000	0.9897220000
C	-1.1605240000	1.6907310000	1.2406730000
H	-1.8653740000	1.8710700000	2.0657460000
H	-0.1708460000	1.4994300000	1.6858170000
O	-1.1606680000	2.8515550000	0.4358120000
H	-0.4883220000	2.6304650000	-0.2745410000

AllylPd(**L4**)(THF) Optimization

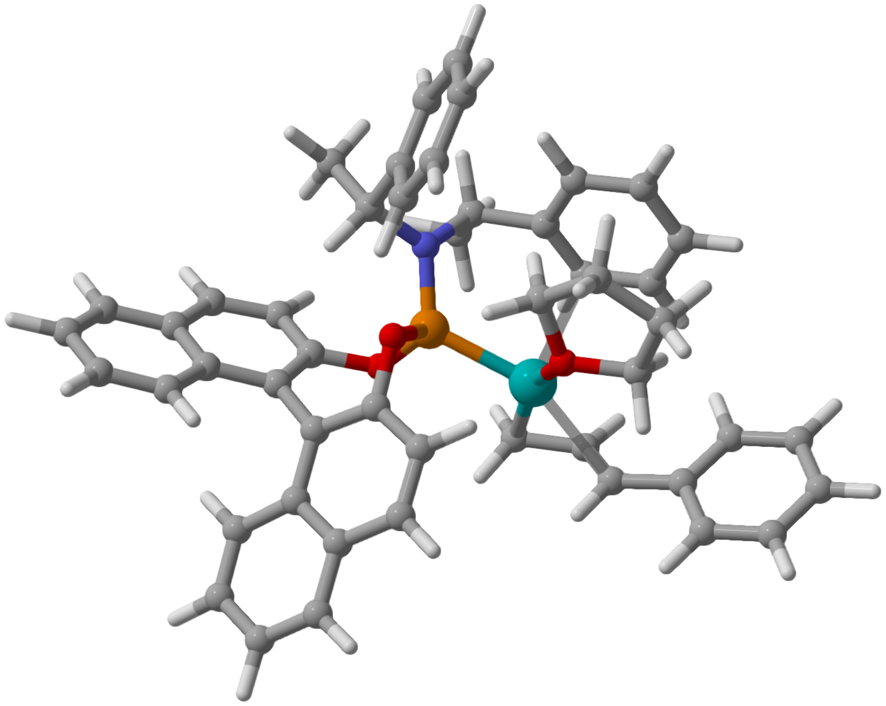

AllylPd(**L4**)(THF) Cartesian coordinates

Pd	1.5591780000	-0.6461680000	-0.6957160000
P	-0.4883430000	0.4192890000	-0.7140830000
O	-1.1901020000	0.2589980000	0.7710720000
O	-1.4859370000	-0.3340340000	-1.7849890000
N	-0.5625050000	2.0589140000	-0.8159400000
C	-1.5190460000	-1.1000690000	0.9436510000
C	-2.7135410000	-1.5613580000	0.4100230000
C	-3.4869530000	-0.6588230000	-0.4631620000
C	-2.8466240000	-0.0749740000	-1.5476660000
C	-0.6482050000	-1.8962360000	1.7338760000
C	-1.0257580000	-3.1865290000	2.0082870000
C	-2.2441880000	-3.7252120000	1.4835470000
C	-3.0812960000	-2.9273150000	0.6541910000
C	-2.6239990000	-5.0771090000	1.7642560000
C	-3.7645890000	-5.6087980000	1.2189660000
C	-4.5812210000	-4.8224870000	0.3541950000
C	-4.2487740000	-3.5212690000	0.0759800000
C	-4.8631590000	-0.3244460000	-0.2302910000
C	-5.5477020000	0.4792460000	-1.1835480000
C	-4.8584630000	0.9628730000	-2.3410130000
C	-3.5226850000	0.7066410000	-2.5218570000
C	-5.5622580000	-0.7543380000	0.9426050000
C	-6.8803400000	-0.4234250000	1.1291550000
C	-7.5751860000	0.3567180000	0.1588480000
C	-6.9261440000	0.7992330000	-0.9654970000

C	1.2207520000	-1.7724420000	-2.4576840000
C	3.1281890000	-2.5929270000	-1.1487510000
C	2.6270690000	-1.9538950000	-2.2780800000
C	0.3592570000	2.7295990000	-1.7618110000
C	0.0739310000	2.3637560000	-3.2263430000
C	-1.6555590000	2.8064960000	-0.1338650000
C	-2.4386450000	3.7114340000	-1.0865910000
C	1.8126240000	2.4982220000	-1.3589580000
C	2.6072370000	1.4991670000	-1.9460580000
C	3.9410640000	1.3421290000	-1.5481490000
C	4.4950890000	2.1859910000	-0.5810720000
C	3.7117030000	3.1906760000	-0.0050860000
C	2.3769060000	3.3429890000	-0.3899920000
C	-1.0400890000	3.5687490000	1.0343610000
C	-0.9719760000	2.9278250000	2.2813310000
C	-0.3777390000	3.5688750000	3.3702230000
C	0.1487070000	4.8565570000	3.2239190000
C	-0.5245370000	4.8632740000	0.8949360000
C	0.0687920000	5.5035830000	1.9875670000
H	0.2977660000	-1.4751170000	2.0897720000
H	-0.3949710000	-3.8278540000	2.6266510000
H	-1.9886280000	-5.6736540000	2.4192710000
H	-4.0620370000	-6.6367610000	1.4312180000
H	-5.4718440000	-5.2771110000	-0.0826460000
H	-4.8667480000	-2.9230700000	-0.5969150000
H	-5.4164620000	1.5458810000	-3.0767800000
H	-2.9726340000	1.0592450000	-3.3925300000
H	-5.0254510000	-1.3429870000	1.6890240000
H	-7.4169380000	-0.7486240000	2.0219970000
H	-8.6256430000	0.5953820000	0.3310620000
H	-7.4476420000	1.4017590000	-1.7099210000
H	4.5574370000	0.5616060000	-2.0051520000
H	5.5374970000	2.0675100000	-0.2860020000
H	4.1413830000	3.8593200000	0.7400690000
H	1.7725650000	4.1275710000	0.0705650000
H	-1.3906140000	1.9251760000	2.4014060000
H	-0.3323110000	3.0686710000	4.3369460000
H	0.6077540000	5.3592230000	4.0747130000
H	0.4627450000	6.5137140000	1.8754780000
H	0.6960420000	2.9729230000	-3.8984300000
H	-0.9717230000	2.5681030000	-3.4924800000
H	0.2707160000	1.3178760000	-3.4847640000
H	-3.2094590000	4.2707360000	-0.5357250000
H	-2.9563690000	3.1329180000	-1.8610770000
H	-1.8132200000	4.4532320000	-1.5961970000
H	0.1692880000	3.8450680000	-1.6728930000
H	-2.3997530000	2.0621190000	0.2919310000
H	0.5303590000	-2.5292710000	-2.0861140000
H	2.4432710000	-3.2422020000	-0.6033790000
H	3.3079790000	-1.4277690000	-2.9416200000

H	2.2194750000	0.8912270000	-2.7691530000
H	-0.5928900000	5.3851640000	-0.0583140000
O	2.1121910000	-0.2379850000	1.3691750000
C	3.5395970000	-0.4088080000	1.5674020000
C	1.6570860000	0.9828470000	2.0127790000
C	3.9668160000	0.5405520000	2.6917230000
H	3.6435230000	-1.4808410000	1.8135460000
H	4.0280410000	-0.1869400000	0.5992230000
C	2.7139090000	1.3599020000	3.0549460000
H	1.5467020000	1.7554420000	1.2328590000
H	0.6761000000	0.7115910000	2.4418860000
H	4.7918110000	1.1991450000	2.3657870000
H	4.3462000000	-0.0141570000	3.5685200000
H	2.9208870000	2.4450170000	3.0412330000
H	2.3719350000	1.1252040000	4.0789680000
H	0.8637340000	-1.2500740000	-3.3380960000
C	4.5138350000	-2.6169030000	-0.6944120000
C	4.8782920000	-3.5339870000	0.3128540000
C	5.4978880000	-1.7360470000	-1.1921440000
C	6.1851130000	-3.5851550000	0.7908670000
H	4.1301750000	-4.2170160000	0.7065830000
C	6.8005360000	-1.7873520000	-0.7100270000
H	5.2354650000	-0.9921440000	-1.9428180000
C	7.1495300000	-2.7125800000	0.2808840000
H	6.4515730000	-4.3044720000	1.5584950000
H	7.5469450000	-1.1040810000	-1.1021830000
H	8.1677490000	-2.7496490000	0.6538070000

TS1 Optimization

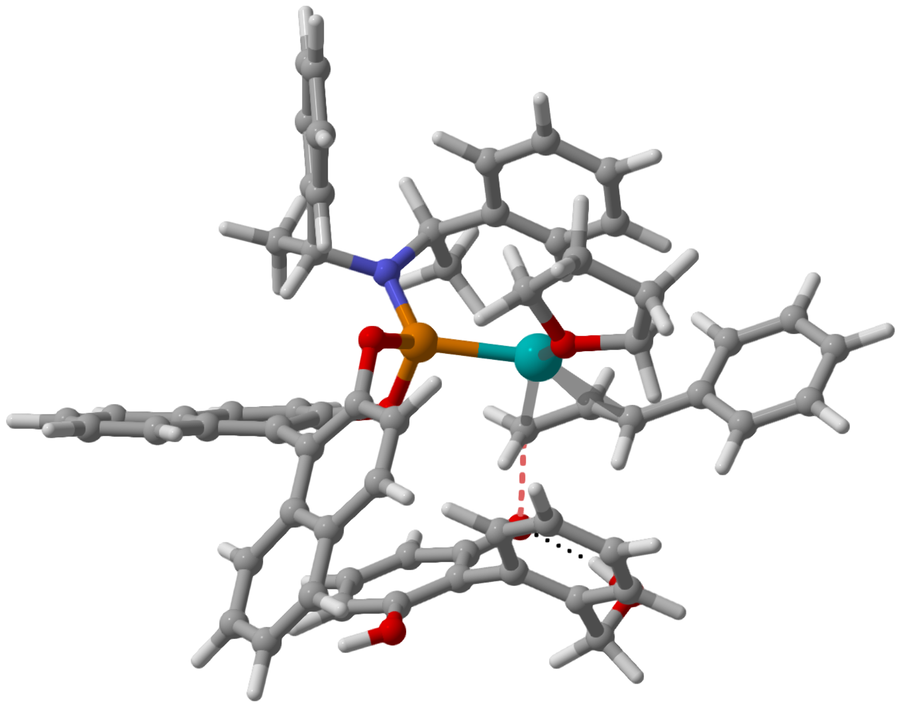

TS1 Cartesian coordinates

Pd	1.8666940000	0.3916720000	-0.3027440000
P	-0.4106570000	1.0670920000	-0.3690450000
O	-1.2809630000	1.4030080000	1.0222760000
O	-1.1816580000	-0.2467090000	-1.0237570000
N	-0.8200580000	2.4637310000	-1.1621220000
C	-1.7264220000	0.2607770000	1.6947850000
C	-2.8250790000	-0.4499870000	1.2373450000
C	-3.4041460000	-0.1485040000	-0.0820290000
C	-2.5630040000	-0.1439370000	-1.1838660000
C	-1.0834870000	-0.0178830000	2.9334190000
C	-1.5502950000	-1.0487920000	3.7044520000
C	-2.6610570000	-1.8411210000	3.2682480000
C	-3.2923810000	-1.5594430000	2.0264950000
C	-3.1296020000	-2.9365400000	4.0620320000
C	-4.1707610000	-3.7180420000	3.6298190000
C	-4.7884620000	-3.4543950000	2.3732690000
C	-4.3576710000	-2.4128690000	1.5911330000
C	-4.7999010000	0.1073410000	-0.3011290000
C	-5.2884570000	0.1748210000	-1.6339840000
C	-4.3893150000	-0.0006510000	-2.7366930000
C	-3.0427780000	-0.1359940000	-2.5251870000
C	-5.7105200000	0.3209080000	0.7824670000
C	-7.0391230000	0.5623730000	0.5403190000
C	-7.5339890000	0.6037430000	-0.7952770000

C	-6.6812620000	0.4166280000	-1.8540740000
C	1.3891630000	-1.1788370000	-2.0265010000
C	3.5168820000	-1.1885480000	-0.8318180000
C	2.7719790000	-0.9193060000	-1.9878750000
C	0.2170630000	3.0384840000	-2.0493110000
C	0.4010860000	2.2028810000	-3.3270050000
C	-2.1323830000	3.1386730000	-0.9839530000
C	-2.8563600000	3.3380140000	-2.3196340000
C	1.5088150000	3.2789340000	-1.2707570000
C	2.6604130000	2.4858450000	-1.4373770000
C	3.8047650000	2.7500710000	-0.6673320000
C	3.8191590000	3.8128820000	0.2393230000
C	2.6886070000	4.6243270000	0.3804700000
C	1.5379940000	4.3490760000	-0.3626930000
C	-1.9247180000	4.4422270000	-0.2181720000
C	-1.8082160000	4.3782680000	1.1803310000
C	-1.5914500000	5.5394880000	1.9235410000
C	-1.4902840000	6.7772940000	1.2787640000
C	-1.8243210000	5.6835710000	-0.8586380000
C	-1.6090920000	6.8464930000	-0.1115380000
H	-0.2192000000	0.5911380000	3.2229490000
H	-1.0719420000	-1.2957000000	4.6526210000
H	-2.6335520000	-3.1422610000	5.0099640000
H	-4.5316790000	-4.5530020000	4.2284310000
H	-5.6029660000	-4.0985030000	2.0432700000
H	-4.8086500000	-2.2351050000	0.6117820000
H	-4.7982040000	-0.0204490000	-3.7470440000
H	-2.3261080000	-0.2854000000	-3.3333290000
H	-5.3231790000	0.2923810000	1.8022260000
H	-7.7345920000	0.7268330000	1.3624530000
H	-8.5948980000	0.7874320000	-0.9563160000
H	-7.0488530000	0.4494180000	-2.8789040000
H	4.6914140000	2.1274500000	-0.7862460000
H	4.7104470000	4.0090820000	0.8321380000
H	2.7011050000	5.4672050000	1.0672420000
H	0.6524230000	4.9742960000	-0.2298730000
H	-1.8817540000	3.4118080000	1.6888470000
H	-1.5044070000	5.4815370000	3.0070110000
H	-1.3238630000	7.6826700000	1.8587460000
H	-1.5356840000	7.8077590000	-0.6170830000
H	1.0069110000	2.7486310000	-4.0604550000
H	-0.5660370000	1.9860330000	-3.8001400000
H	0.8929480000	1.2354630000	-3.1632320000
H	-3.8479790000	3.7798970000	-2.1572150000
H	-3.0126560000	2.3798510000	-2.8319010000
H	-2.3132610000	3.9883540000	-3.0124300000
H	-0.1489590000	4.0561420000	-2.3873600000
H	-2.7948770000	2.4749990000	-0.3458000000

H	0.8884890000	-1.6757290000	-1.2192070000
H	3.0938730000	-1.9088850000	-0.1371040000
H	3.2375250000	-0.4423100000	-2.8461110000
H	2.7156090000	1.7407940000	-2.2418350000
H	-1.9161570000	5.7545580000	-1.9410150000
O	2.3757550000	0.7802340000	1.8478970000
C	3.7823940000	0.6521300000	2.1642100000
C	1.8262600000	1.9760130000	2.4514250000
C	4.0798660000	1.5882320000	3.3418940000
H	3.9024410000	-0.4214410000	2.3917050000
H	4.3405050000	0.9170400000	1.2475390000
C	2.8391090000	2.4909430000	3.4823610000
H	1.6375590000	2.6934970000	1.6350210000
H	0.8678160000	1.6357870000	2.8970200000
H	4.9910990000	2.1827500000	3.1613170000
H	4.2590940000	1.0179960000	4.2698170000
H	3.0933160000	3.5483040000	3.2932050000
H	2.4312140000	2.4465800000	4.5061100000
H	0.7739610000	-0.8211730000	-2.8382570000
C	4.9415810000	-0.9029260000	-0.6415390000
C	5.6040370000	-1.4948910000	0.4528840000
C	5.6818720000	-0.0498540000	-1.4836820000
C	6.9491240000	-1.2331060000	0.7044300000
H	5.0492390000	-2.1790010000	1.0893120000
C	7.0266630000	0.2070140000	-1.2327470000
H	5.2029810000	0.4140940000	-2.3407960000
C	7.6664560000	-0.3767210000	-0.1342910000
H	7.4409190000	-1.7056360000	1.5506250000
H	7.5802250000	0.8655100000	-1.8968360000
H	8.7158440000	-0.1728070000	0.0584400000
C	-0.1844740000	-3.5099320000	-2.3313680000
C	-0.3871700000	-3.7797560000	-0.9323030000
C	-1.6925240000	-4.0375900000	-0.4730350000
C	-2.8001540000	-4.0198560000	-1.3262410000
C	-2.6033570000	-3.7293480000	-2.6805390000
C	-1.3324370000	-3.4820680000	-3.1744950000
H	-3.7933120000	-4.2321490000	-0.9341540000
H	-3.4610810000	-3.7040150000	-3.3496070000
H	-1.1638560000	-3.2594520000	-4.2229970000
C	0.7095510000	-3.6675740000	0.0778510000
C	1.8873220000	-4.4441460000	0.0605890000
C	0.5548170000	-2.7188060000	1.1107300000
C	2.8456640000	-4.2571240000	1.0698310000
C	1.5124250000	-2.5392920000	2.1039270000
H	-0.3497550000	-2.1153170000	1.1154530000
C	2.6697570000	-3.3244770000	2.0910230000
H	3.7495860000	-4.8614000000	1.0407580000
H	1.3627900000	-1.7835420000	2.8706670000

H	3.4231290000	-3.2116060000	2.8672180000
O	-1.8399660000	-4.3329770000	0.8643820000
H	-2.7876090000	-4.4237740000	1.0521700000
O	0.9866900000	-3.2196020000	-2.8387480000
C	2.1988230000	-5.4262650000	-1.0549840000
H	2.7808210000	-6.2632360000	-0.6500580000
H	1.2617320000	-5.8365140000	-1.4602740000
O	2.9892620000	-4.8250020000	-2.0648740000
H	2.3774330000	-4.1611150000	-2.4729290000

TS2 Optimization

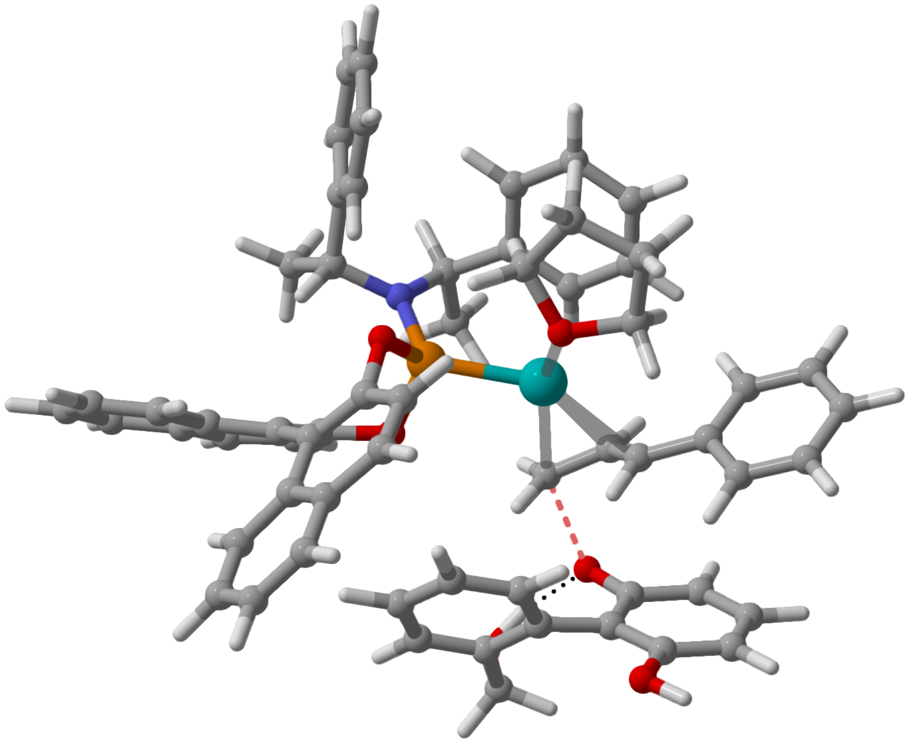

TS2 Cartesian coordinates

Pd	1.1420570000	1.0748870000	-0.1528700000
P	-1.1357390000	0.6597030000	-0.5118270000
O	-2.1056580000	0.6691470000	0.8555260000
O	-1.2708210000	-0.8840670000	-1.1058340000
N	-2.0896880000	1.7205230000	-1.3662820000
C	-2.0112590000	-0.5030880000	1.6104300000
C	-2.6984870000	-1.6403010000	1.2163940000
C	-3.3349260000	-1.6803170000	-0.1112270000
C	-2.5779360000	-1.3606310000	-1.2293360000
C	-1.3255830000	-0.3949460000	2.8524160000
C	-1.3432120000	-1.4644310000	3.7074220000
C	-2.0171250000	-2.6763900000	3.3478470000
C	-2.6756910000	-2.7818900000	2.0921740000
C	-2.0042970000	-3.8028770000	4.2310630000
C	-2.5991950000	-4.9835290000	3.8661590000
C	-3.2311470000	-5.1023430000	2.5946190000
C	-3.2666440000	-4.0361700000	1.7323550000
C	-4.7120920000	-2.0379020000	-0.3104270000
C	-5.1954270000	-2.2231730000	-1.6337600000
C	-4.3172770000	-2.0307750000	-2.7494640000
C	-3.0358320000	-1.5853040000	-2.5599600000
C	-5.6228210000	-2.1866420000	0.7844160000
C	-6.9316160000	-2.5327190000	0.5629140000

C	-7.4074080000	-2.7496140000	-0.7627390000
C	-6.5624120000	-2.5964460000	-1.8327920000
C	1.9169260000	-0.5229860000	-1.8704960000
C	3.4203090000	0.3560730000	-0.1571550000
C	2.8928360000	0.3997520000	-1.4501970000
C	-1.4035090000	2.5961080000	-2.3429330000
C	-0.8700730000	1.8040340000	-3.5481750000
C	-3.5601550000	1.8194340000	-1.1684780000
C	-4.3270710000	1.6365490000	-2.4824410000
C	-0.3435620000	3.4461290000	-1.6467250000
C	1.0289390000	3.1525590000	-1.7279410000
C	1.9569330000	3.9601470000	-1.0553070000
C	1.5296550000	5.0630260000	-0.3118830000
C	0.1669340000	5.3714650000	-0.2484680000
C	-0.7629670000	4.5631470000	-0.9080960000
C	-3.8774000000	3.1402890000	-0.4711290000
C	-3.6795000000	3.2225340000	0.9173360000
C	-3.9179070000	4.4189850000	1.5949880000
C	-4.3653890000	5.5451200000	0.8952660000
C	-4.3299150000	4.2690570000	-1.1658590000
C	-4.5746400000	5.4657370000	-0.4837290000
H	-0.7926070000	0.5348470000	3.0783110000
H	-0.8302630000	-1.4162010000	4.6683560000
H	-1.5029660000	-3.7046910000	5.1931260000
H	-2.5879410000	-5.8465560000	4.5302450000
H	-3.6750720000	-6.0579480000	2.3166650000
H	-3.7310160000	-4.1378660000	0.7493920000
H	-4.6914810000	-2.2384850000	-3.7524210000
H	-2.3401560000	-1.4232380000	-3.3831610000
H	-5.2550390000	-2.0122090000	1.7972190000
H	-7.6266030000	-2.6453380000	1.3943680000
H	-8.4484180000	-3.0343400000	-0.9068870000
H	-6.9165340000	-2.7546230000	-2.8507580000
H	3.0236060000	3.7211200000	-1.1216890000
H	2.2554010000	5.6813660000	0.2123680000
H	-0.1723760000	6.2389250000	0.3127380000
H	-1.8254050000	4.8043220000	-0.8405510000
H	-3.3337810000	2.3442760000	1.4710030000
H	-3.7595160000	4.4739120000	2.6704100000
H	-4.5543820000	6.4769600000	1.4240650000
H	-4.9298920000	6.3368400000	-1.0315420000
H	-0.5308490000	2.4870720000	-4.3372340000
H	-1.6538840000	1.1695960000	-3.9823840000
H	-0.0237730000	1.1456640000	-3.3168390000
H	-5.4095580000	1.6525650000	-2.3020970000
H	-4.0933610000	0.6697650000	-2.9459700000
H	-4.1023130000	2.4097230000	-3.2242570000
H	-2.1750830000	3.3159590000	-2.7556850000

H	-3.8898580000	0.9842740000	-0.4754700000
H	1.5846850000	-1.3386390000	-1.2464140000
H	3.1779040000	-0.5236720000	0.4302880000
H	3.2785920000	1.1141000000	-2.1722680000
H	1.3884090000	2.3563580000	-2.3897280000
H	-4.4968300000	4.2255080000	-2.2407110000
O	1.0786990000	1.9423010000	1.9519840000
C	2.3211430000	2.6108670000	2.2764450000
C	-0.0347140000	2.8363960000	2.1995290000
C	1.9837550000	3.7475070000	3.2511420000
H	2.9499040000	1.8100080000	2.7002560000
H	2.7522420000	2.9758410000	1.3255130000
C	0.4540700000	3.9193100000	3.1719720000
H	-0.3509570000	3.2521780000	1.2267760000
H	-0.8178370000	2.1736690000	2.6151850000
H	2.5084040000	4.6780320000	2.9776520000
H	2.3005550000	3.5010150000	4.2788970000
H	0.1829870000	4.9263840000	2.8116750000
H	-0.0083470000	3.8069530000	4.1666600000
H	1.4445400000	-0.4254570000	-2.8374950000
C	4.5379780000	1.1490490000	0.3568330000
C	5.2778580000	0.6367870000	1.4406550000
C	4.9139690000	2.3976520000	-0.1751830000
C	6.3554490000	1.3449970000	1.9687500000
H	5.0177670000	-0.3395050000	1.8401650000
C	5.9883540000	3.1049530000	0.3554560000
H	4.3422270000	2.8245620000	-1.0000480000
C	6.7146010000	2.5831390000	1.4315040000
H	6.9200380000	0.9263120000	2.7975920000
H	6.2603950000	4.0679130000	-0.0684210000
H	7.5529990000	3.1369230000	1.8442800000
C	4.3277780000	-2.1651340000	-1.8795720000
C	4.1122050000	-2.8995370000	-0.6579350000
C	5.1753660000	-3.0249440000	0.2503600000
C	6.4349060000	-2.4669000000	0.0023330000
C	6.6399360000	-1.7482030000	-1.1792260000
C	5.6122760000	-1.5889880000	-2.0945450000
H	7.2368360000	-2.5902680000	0.7276370000
H	7.6127220000	-1.3009690000	-1.3688560000
H	5.7497420000	-1.0232000000	-3.0103940000
C	2.7472000000	-3.3910930000	-0.3170070000
C	1.9934470000	-4.2173550000	-1.1781720000
C	2.1435690000	-2.9396540000	0.8737410000
C	0.6535410000	-4.4864730000	-0.8665360000
C	0.8173530000	-3.2306650000	1.1790040000
H	2.7337260000	-2.3490530000	1.5672110000
C	0.0574570000	-3.9949870000	0.2906770000
H	0.0757620000	-5.0948460000	-1.5583440000

H	0.3782010000	-2.8580450000	2.1027820000
H	-0.9863460000	-4.2152410000	0.5066490000
O	4.9361580000	-3.7118460000	1.4234500000
H	5.7757320000	-3.7849350000	1.9019100000
O	3.3777600000	-1.9530610000	-2.7482080000
C	2.5573770000	-4.8194590000	-2.4550640000
H	3.6559410000	-4.8378480000	-2.4030980000
H	2.2151010000	-5.8586190000	-2.5364860000
O	2.0972650000	-4.1522580000	-3.6179230000
H	2.4705070000	-3.2464930000	-3.5122100000

TS3 Optimization

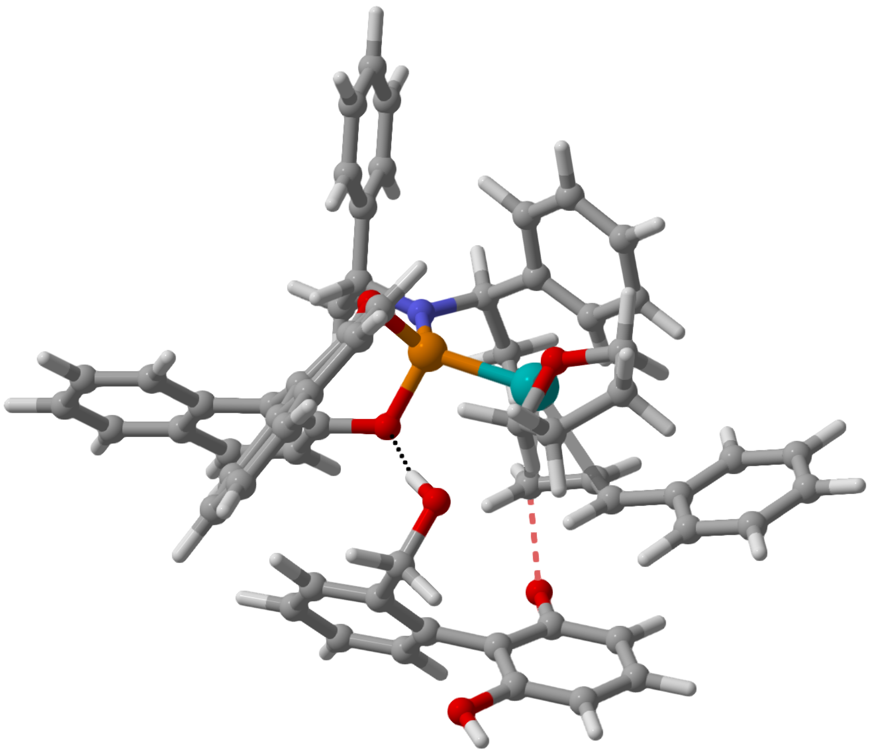

TS3 Cartesian coordinates

Pd	1.1759910000	1.4920550000	-0.0834230000
P	-1.1344710000	1.1223320000	-0.3247430000
O	-2.2785070000	1.0842170000	0.9034180000
O	-1.1587340000	-0.4763030000	-0.8835100000
N	-2.0250690000	2.0497520000	-1.3744100000
C	-1.9583020000	0.0799400000	1.8235640000
C	-2.3325230000	-1.2291030000	1.5564600000
C	-2.9905780000	-1.5380630000	0.2753830000
C	-2.3793060000	-1.1467470000	-0.9061480000
C	-1.3660160000	0.4863690000	3.0518240000
C	-1.1105880000	-0.4681800000	4.0023010000
C	-1.3974220000	-1.8485440000	3.7498970000
C	-1.9910160000	-2.2394350000	2.5190100000
C	-1.0656730000	-2.8522950000	4.7154550000
C	-1.2904820000	-4.1790210000	4.4493930000
C	-1.8538130000	-4.5748780000	3.2013420000
C	-2.1942810000	-3.6338620000	2.2638780000
C	-4.2595980000	-2.2061540000	0.1910580000
C	-4.7796300000	-2.5435230000	-1.0878710000
C	-4.0530350000	-2.1958880000	-2.2734330000
C	-2.8779430000	-1.4980110000	-2.1933170000
C	-5.0299420000	-2.5260650000	1.3545900000
C	-6.2326710000	-3.1763210000	1.2408950000

C	-6.7386790000	-3.5410870000	-0.0399690000
C	-6.0324520000	-3.2291900000	-1.1743470000
C	1.6123280000	0.2175820000	-2.1176000000
C	3.5713460000	0.4029170000	-0.6829620000
C	2.8005190000	0.8745040000	-1.7342620000
C	-1.2783260000	3.1723350000	-1.9948320000
C	-0.6315650000	2.7263680000	-3.3148470000
C	-3.5088280000	1.9930870000	-1.4260460000
C	-3.9924230000	1.7583670000	-2.8621050000
C	-0.3065480000	3.7598100000	-0.9751840000
C	1.0938760000	3.5999880000	-1.0843270000
C	1.9310400000	4.0406880000	-0.0387780000
C	1.3780220000	4.6751980000	1.0783310000
C	-0.0004840000	4.9011490000	1.1506560000
C	-0.8375680000	4.4436180000	0.1297410000
C	-4.1096120000	3.2443790000	-0.7929540000
C	-4.2772760000	3.2711070000	0.6009670000
C	-4.7975470000	4.4049980000	1.2271890000
C	-5.1553920000	5.5242550000	0.4678090000
C	-4.4739340000	4.3656080000	-1.5489980000
C	-4.9939310000	5.5012310000	-0.9199040000
H	-1.1140310000	1.5378360000	3.1960600000
H	-0.6628070000	-0.1918690000	4.9570920000
H	-0.6249850000	-2.5394800000	5.6608270000
H	-1.0370160000	-4.9479510000	5.1775800000
H	-2.0006220000	-5.6364820000	3.0040510000
H	-2.6072210000	-3.9349060000	1.2966050000
H	-4.4532690000	-2.5002840000	-3.2407110000
H	-2.2830450000	-1.2359860000	-3.0689760000
H	-4.6446640000	-2.2359520000	2.3334150000
H	-6.8192690000	-3.4210620000	2.1254850000
H	-7.6912380000	-4.0646170000	-0.0989610000
H	-6.4126250000	-3.4969790000	-2.1593630000
H	3.0083060000	3.8897060000	-0.1079820000
H	2.0245070000	5.0033510000	1.8898050000
H	-0.4238220000	5.4246580000	2.0040220000
H	-1.9152700000	4.6057200000	0.2051020000
H	-3.9896100000	2.4022160000	1.2019280000
H	-4.9246380000	4.4165660000	2.3083390000
H	-5.5601180000	6.4082980000	0.9562680000
H	-5.2742790000	6.3684710000	-1.5149680000
H	-0.1310040000	3.5653600000	-3.8102870000
H	-1.3898980000	2.3378850000	-4.0070820000
H	0.1084310000	1.9180420000	-3.1989460000
H	-5.0825950000	1.6397170000	-2.8876160000
H	-3.5521300000	0.8422490000	-3.2771900000
H	-3.7316930000	2.5764720000	-3.5410570000
H	-2.0139400000	3.9910080000	-2.2585180000

H	-3.8546870000	1.1032610000	-0.8137910000
H	1.2761780000	-0.6809720000	-1.6275840000
H	3.2432430000	-0.5232940000	-0.2135720000
H	3.1446720000	1.7140850000	-2.3319600000
H	1.5406160000	3.2431390000	-2.0200530000
H	-4.3587950000	4.3598720000	-2.6318760000
O	1.8739870000	1.5077210000	2.1906710000
C	2.0240620000	0.0765020000	2.3513940000
C	3.0542000000	2.1649180000	2.7131470000
C	2.9689810000	-0.1394940000	3.5416190000
H	0.9967090000	-0.2968530000	2.4938550000
H	2.4340260000	-0.3359490000	1.4016890000
C	3.7374620000	1.1882030000	3.6844900000
H	3.6907070000	2.4202330000	1.8486950000
H	2.6678390000	3.0766810000	3.1912610000
H	3.6484330000	-0.9892030000	3.3655050000
H	2.4054360000	-0.3735110000	4.4598180000
H	4.8037340000	1.0601110000	3.4277280000
H	3.7045550000	1.5579720000	4.7213370000
H	1.0159000000	0.5813440000	-2.9471130000
C	4.7954510000	0.9886290000	-0.1519610000
C	5.5433560000	0.2472160000	0.7855240000
C	5.2547570000	2.2743830000	-0.5032900000
C	6.7050640000	0.7696090000	1.3495190000
H	5.2081440000	-0.7530620000	1.0475150000
C	6.4137520000	2.7950240000	0.0638890000
H	4.6943620000	2.8706560000	-1.2166990000
C	7.1441260000	2.0477830000	0.9948110000
H	7.2697720000	0.1784410000	2.0655190000
H	6.7501440000	3.7891730000	-0.2176850000
H	8.0468260000	2.4591670000	1.4374060000
C	3.8652030000	-3.7183750000	-1.0653140000
C	2.9450490000	-3.0284730000	-1.8602800000
C	3.4187380000	-1.9279890000	-2.6779810000
C	4.7881680000	-1.5359470000	-2.5310580000
C	5.6602230000	-2.2296900000	-1.7023820000
C	5.2101470000	-3.3216540000	-0.9606780000
H	5.1236630000	-0.6949160000	-3.1284520000
H	6.6975160000	-1.9153260000	-1.6177620000
H	5.8918080000	-3.8854770000	-0.3263100000
C	1.5213500000	-3.4496180000	-1.9669750000
C	0.6547510000	-3.5741560000	-0.8549430000
C	1.0021400000	-3.7152270000	-3.2460250000
C	-0.6655700000	-3.9995140000	-1.0578750000
C	-0.3158610000	-4.1275420000	-3.4320990000
H	1.6542620000	-3.5842060000	-4.1011400000
C	-1.1575610000	-4.2852030000	-2.3307650000
H	-1.3251800000	-4.0922080000	-0.1933020000

H	-0.6801650000	-4.3320430000	-4.4352400000
H	-2.1870090000	-4.6116450000	-2.4582470000
O	2.6332260000	-1.2741070000	-3.4605960000
O	3.4197930000	-4.8250460000	-0.3709790000
H	4.1798110000	-5.2242340000	0.0787240000
C	1.0398760000	-3.1251610000	0.5376720000
H	0.3559870000	-3.5855160000	1.2701160000
H	2.0529490000	-3.4180660000	0.8030880000
O	0.9894700000	-1.6931070000	0.6568250000
H	0.1844930000	-1.3925500000	0.1926710000

INT1 Optimization

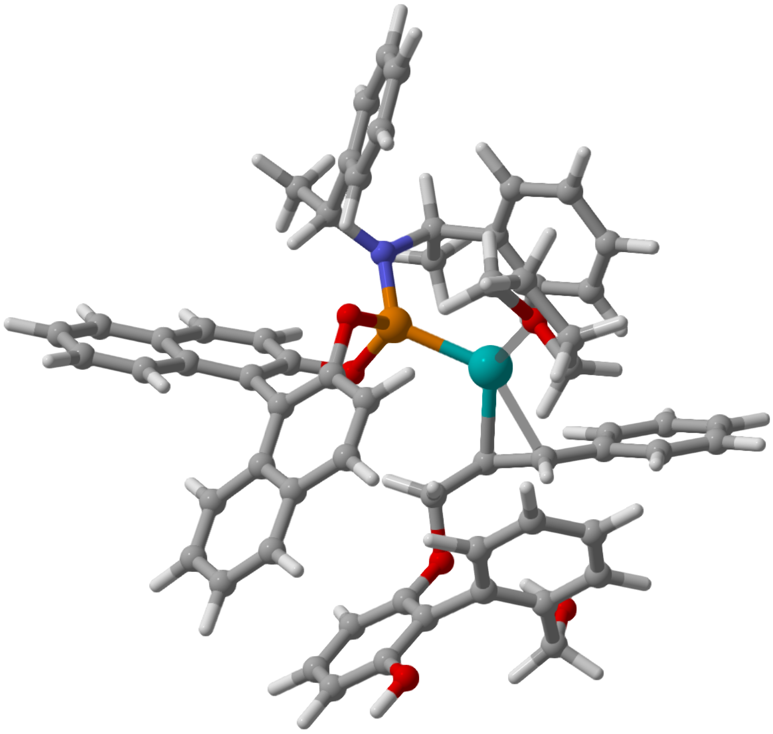

INT1 Cartesian coordinates

Pd	0.2380470000	1.6805430000	-0.4746460000
P	-1.7000990000	0.4249410000	-0.6269000000
O	-2.0635610000	-0.4195150000	0.7848200000
O	-1.5125730000	-0.8024950000	-1.7646180000
N	-3.2162110000	1.0872010000	-0.8037830000
C	-1.1104490000	-1.4225610000	0.9764340000
C	-1.2678690000	-2.6422740000	0.3322570000
C	-2.3502190000	-2.7795300000	-0.6575550000
C	-2.4266510000	-1.8483250000	-1.6863860000
C	-0.0744840000	-1.1722300000	1.9202930000
C	0.7992750000	-2.1830310000	2.2188230000
C	0.6926630000	-3.4590110000	1.5784150000
C	-0.3170320000	-3.6835190000	0.6032690000
C	1.6044170000	-4.5148250000	1.8982820000
C	1.5252670000	-5.7235940000	1.2547090000
C	0.5490300000	-5.9316100000	0.2374690000
C	-0.3415560000	-4.9396720000	-0.0844060000
C	-3.3522630000	-3.8036550000	-0.5880730000
C	-4.3049430000	-3.9121730000	-1.6373750000
C	-4.2738610000	-2.9866050000	-2.7298910000
C	-3.3641750000	-1.9627630000	-2.7540640000
C	-3.4412120000	-4.7084390000	0.5190530000
C	-4.4057000000	-5.6828930000	0.5510900000
C	-5.3407270000	-5.8104170000	-0.5170660000

C	-5.2936260000	-4.9454470000	-1.5807580000
C	1.8512640000	-0.7823520000	-1.1185020000
C	2.4592670000	1.6420890000	-1.0154850000
C	1.7449610000	0.6169940000	-1.6467400000
C	-3.2416360000	2.3819220000	-1.5253300000
C	-3.0173050000	2.1926640000	-3.0378400000
C	-4.4499590000	0.5065210000	-0.2195920000
C	-5.5547270000	0.3279120000	-1.2678130000
C	-2.2664690000	3.3671610000	-0.8920470000
C	-0.9481030000	3.5496580000	-1.3652410000
C	-0.0912230000	4.4436180000	-0.6906690000
C	-0.5482400000	5.1587780000	0.4177890000
C	-1.8703120000	5.0101550000	0.8582970000
C	-2.7144490000	4.1038060000	0.2154380000
C	-4.8792360000	1.3734220000	0.9627900000
C	-4.2199060000	1.1924360000	2.1901660000
C	-4.5320450000	1.9994990000	3.2848910000
C	-5.5133130000	2.9905100000	3.1695080000
C	-5.8630180000	2.3637750000	0.8537380000
C	-6.1797580000	3.1670900000	1.9545440000
H	-0.0031640000	-0.1684860000	2.3633410000
H	1.6133840000	-2.0235310000	2.9323890000
H	2.3663980000	-4.3332800000	2.6551790000
H	2.2112740000	-6.5326720000	1.4963660000
H	0.5283160000	-6.8885290000	-0.2813300000
H	-1.0796880000	-5.0885210000	-0.8747370000
H	-4.9952600000	-3.1084170000	-3.5376910000
H	-3.3182470000	-1.2345850000	-3.5620370000
H	-2.7313220000	-4.5988150000	1.3407190000
H	-4.4751620000	-6.3705490000	1.3928550000
H	-6.0895580000	-6.5985270000	-0.4677220000
H	-6.0083310000	-5.0273420000	-2.3982930000
H	0.9349510000	4.5712770000	-1.0399460000
H	0.1272920000	5.8282850000	0.9449940000
H	-2.2304290000	5.5849120000	1.7052110000
H	-3.7304660000	3.9616640000	0.5843280000
H	-3.4493670000	0.4212750000	2.2843740000
H	-4.0098620000	1.8578890000	4.2289220000
H	-5.7559000000	3.6199540000	4.0224770000
H	-6.9457480000	3.9342440000	1.8605270000
H	-3.1650430000	3.1429330000	-3.5636320000
H	-3.7217540000	1.4636830000	-3.4528070000
H	-2.0093850000	1.8423150000	-3.2919170000
H	-6.4488210000	-0.1168600000	-0.8152610000
H	-5.2247660000	-0.3431780000	-2.0702150000
H	-5.8522950000	1.2703020000	-1.7388430000
H	-4.2787820000	2.8181910000	-1.4110620000
H	-4.2062170000	-0.5257690000	0.1811120000

H	1.9120760000	-0.7917350000	-0.0243160000
H	2.9847870000	1.3814230000	-0.0989460000
H	1.4997180000	0.6853410000	-2.7039760000
H	-0.6282660000	3.1204900000	-2.3272530000
H	-6.3863070000	2.5191290000	-0.0876180000
O	0.2788030000	2.7765450000	1.5854820000
C	1.6011170000	2.7908580000	2.1677540000
C	-0.6657080000	2.3274880000	2.5824310000
C	1.4225890000	2.9469870000	3.6886150000
H	2.0927000000	1.8422880000	1.8822410000
H	2.0991210000	3.6396760000	1.6727810000
C	-0.0833200000	2.7319210000	3.9469980000
H	-1.6096210000	2.8251060000	2.3070340000
H	-0.7646690000	1.2271490000	2.4816270000
H	1.7412960000	3.9459300000	4.0278860000
H	2.0350560000	2.2174550000	4.2413410000
H	-0.5530090000	3.6594450000	4.3155440000
H	-0.2609900000	1.9613630000	4.7122250000
H	1.0098560000	-1.4083590000	-1.4362370000
C	2.8564960000	2.9263080000	-1.6052830000
C	3.6738080000	3.7973360000	-0.8579480000
C	2.4446080000	3.3515670000	-2.8845870000
C	4.0526140000	5.0410450000	-1.3567040000
H	4.0215150000	3.4753540000	0.1211300000
C	2.8251580000	4.5947320000	-3.3831130000
H	1.8232140000	2.7007030000	-3.4918600000
C	3.6261220000	5.4518480000	-2.6223780000
H	4.6878240000	5.6906710000	-0.7586660000
H	2.4957360000	4.8973590000	-4.3743980000
H	3.9212210000	6.4213300000	-3.0149880000
C	3.5156820000	-2.5170640000	-1.0304770000
C	4.2299820000	-2.4362070000	0.1809640000
C	4.6831550000	-3.6425390000	0.7459700000
C	4.4547170000	-4.8702320000	0.1162310000
C	3.7415740000	-4.9140020000	-1.0817110000
C	3.2656770000	-3.7391080000	-1.6581600000
H	4.8260970000	-5.7862020000	0.5704060000
H	3.5537500000	-5.8707520000	-1.5603240000
H	2.7035760000	-3.7412980000	-2.5859360000
C	4.4955450000	-1.1299740000	0.8554990000
C	5.3232830000	-0.1434950000	0.2839800000
C	3.8954770000	-0.8794310000	2.1006370000
C	5.4943160000	1.0732320000	0.9595030000
C	4.0792860000	0.3309120000	2.7642120000
H	3.2737370000	-1.6541290000	2.5465460000
C	4.8808540000	1.3199750000	2.1858060000
H	6.1129230000	1.8385960000	0.4979520000
H	3.6082900000	0.4965350000	3.7304070000

H	5.0337060000	2.2696740000	2.6926730000
O	5.3653630000	-3.5593320000	1.9325850000
H	5.6708490000	-4.4460160000	2.1773120000
O	3.0881130000	-1.3638950000	-1.6525060000
C	5.9936420000	-0.3175560000	-1.0682060000
H	6.9977920000	0.1192840000	-1.0314540000
H	6.1086490000	-1.3872350000	-1.2970320000
O	5.3077060000	0.3702170000	-2.1026110000
H	4.4211970000	-0.0342900000	-2.1476210000

2.3 Non-covalent interaction (NCI) analysis

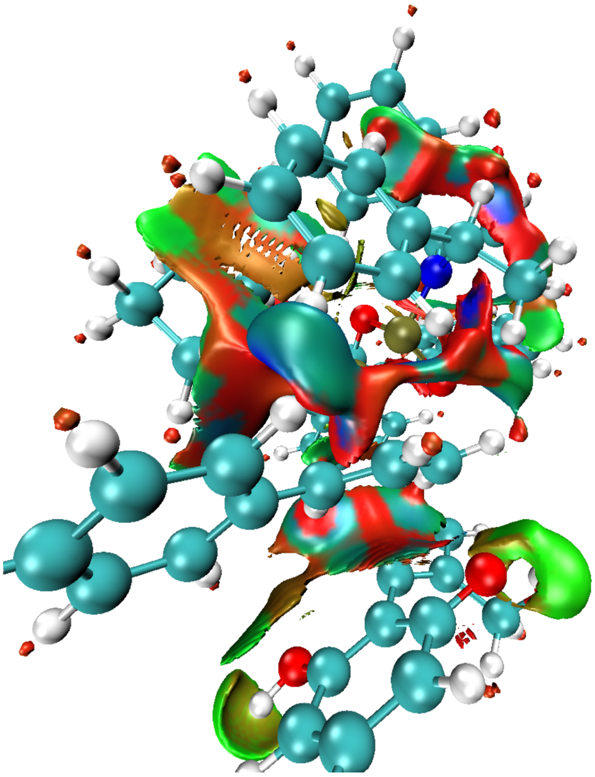

Supplementary Figure 2. Non-covalent interaction (NCI) analysis

Non covalent interaction (NCI) plots have been performed by Multiwfn 3.8[14] and visualized by VMD 1.9.[15] Compared to **TS1**, we found unfavorable steric interactions between the phenyl ring of **L4** and cinnamyl group in **TS2**.

3 Supplementary References

1. Gaussian 16, Revision C.01, Frisch, M. J., *et al.* Wallingford CT, 2016.

2. Dennington, R., Keith, T. A., Millam, J. M. GaussView, Version 6.). Semichem Inc. (2016).

3. Lee, C., Yang, W., Parr, R. G. Development of the Colle-Salvetti correlation-energy formula into a functional of the electron density. *Phy. Rev. B* **37**, 785−789 (1988).

4. Becke, A. D. A new mixing of Hartree–Fock and local density-functional theories. *J. Chem. Phys.* **98**, 1372−1377 (1993).

5. Becke, A. D. Density-functional thermochemistry. III. The role of exact exchange. *J. Chem. Phys.* **98**, 5648−5652 (1993).

6. Zhao, Y., Truhlar, D. G. The M06 suite of density functionals for main group thermochemistry, thermochemical kinetics, noncovalent interactions, excited states, and transition elements: two new functionals and systematic testing of four M06-class functionals and 12 other functionals. *Theor. Chem. Acc.* **120**, 215−241 (2008).

7. CYLview20, Legault, C. Y. Université de Sherbrooke, 2020.

8. Shintani, R., Park, S., Shirozu, F., Murakami, M., Hayashi, T. Palladium-Catalyzed Asymmetric Decarboxylative Lactamization of γ-Methylidene-δ-valerolactones with Isocyanates: Conversion of Racemic Lactones to Enantioenriched Lactams. *J. Am. Chem. Soc.* **130**, 16174−16175 (2008).

9. Li, Z., Liu, L., Fu, Y., Guo, Q.-X. Assessing performance of diverse ONIOM methods in calculation of structures of organonickel and organopalladium compounds. *THEOCHEM* **757**, 69−76 (2005).

10. Ananikov, V. P., Musaev, D. G., Morokuma, K. Real size of ligands, reactants and catalysts: Studies of structure, reactivity and selectivity by ONIOM and other hybrid computational approaches. *J. Mol. Catal. A: Chem.* **324**, 104−119 (2010).

11. Karami, K., Abedanzadeh, S., Farrokhpour, H., Lipkowski, J. Synthesis and characterization of the P,C-palladacycles with bridging and chelating dinitrogen ligands and ONIOM calculations on the pyrazine-bridged organometallic polymers (n = 1 to n = 10). *J. Organomet. Chem.* **805**, 68−76 (2016).

12. Stewart, J. J. P. Optimization of parameters for semiempirical methods V: Modification of NDDO approximations and application to 70 elements. *J. Mol. Model.* **13**, 1173−1213 (2007).

13. Marenich, A. V., Cramer, C. J., Truhlar, D. G. Universal Solvation Model Based on Solute Electron Density and on a Continuum Model of the

Solvent Defined by the Bulk Dielectric Constant and Atomic Surface Tensions. *J. Phys. Chem. B* **113**, 6378−6396 (2009).

14. Lu, T., Chen, F. Multiwfn: A multifunctional wavefunction analyzer. *J. Comput. Chem.* **33**, 580−592 (2012).

15. Humphrey, W., Dalke, A., Schulten, K. VMD: Visual molecular dynamics. *J. Mol. Graph.* **14**, 33−38 (1996).
